# NMR and EPR Study of Homolysis of Diastereomeric Alkoxyamines

**DOI:** 10.3390/molecules25215080

**Published:** 2020-11-01

**Authors:** Sergey Cherkasov, Dmitriy Parkhomenko, Alexander Genaev, Georgii Salnikov, Mariya Edeleva, Denis Morozov, Tatyana Rybalova, Igor Kirilyuk, Sylvain R. A. Marque, Elena Bagryanskaya

**Affiliations:** 1 N. N. Vorozhtsov Novosibirsk Institute of Organic Chemistry SB RAS, Pr. Lavrentjeva 9, 630090 Novosibirsk, Russia; scher@nioch.nsc.ru (S.C.); parkhomenko@nioch.nsc.ru (D.P.); genaev@nioch.nsc.ru (A.G.); sge@nioch.nsc.ru (G.S.); edeleva@nioch.nsc.ru (M.E.); m_falcon@nioch.nsc.ru (D.M.); rybalova@nioch.nsc.ru (T.R.); kirilyuk@nioch.nsc.ru (I.K.); 2 National Research University—Novosibirsk State University, 630090 Novosibirsk, Russia; 3 Aix Marseille Univ, CNRS, ICR, UMR 7273, case 551, Avenue Escadrille Normandie-Niemen, 13397 Marseille CEDEX 20, France; Sylvain.marque@univ-amu.fr

**Keywords:** nitroxide, alkoxyamine, nitroxide mediated polymerization, kinetics, scavenger, imidazoline radical, homolysis, nitrogen inversion, chirality, stereoisomerization

## Abstract

Three alkoxyamines based on imidazoline radicals with a pyridine functional group—potential initiators of nitroxide-mediated, controlled radical polymerization—were synthesized. Electron Paramagnetic Resonance (EPR) measurements reveal biexponential kinetics for the thermolysis for diastereomeric alkoxyamines and monoexponential kinetics for an achiral alkoxyamine. For comparison, the thermolysis of all three alkoxyamines was studied by NMR in the presence of three different scavengers, namely tetramethylpiperidine-N-oxyl (TEMPO), thiophenol (PhSH), and β-mercaptoethanol (BME), and detailed analysis of products was performed. NMR differentiates between *N*-inversion, epimerization, and homolysis reactions. The choice of scavenger is crucial for making a reliable and accurate estimate of the true homolysis rate constant.

## 1. Introduction

**Scheme 1:**
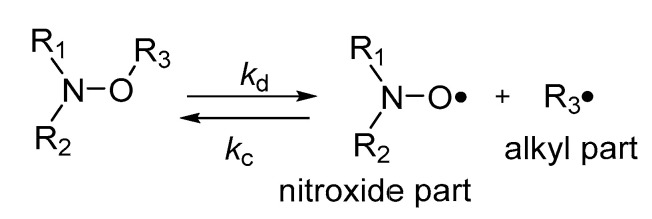
C–ON bond homolysis in alkoxyamines.

Alkoxyamines have been used for three decades [1] as initiators in Nitroxide-Mediated Polymerization (NMP) [2,3,4,5,6,7,8,9,10,11,12,13,14,15]. The main parameters determining the applicability of alkoxyamines for NMP are the rate constants of their homolysis, *k_d_*, the rate of recombination of the corresponding alkyl and nitroxide radicals, *k_c_* (Scheme 1), and the absence of substantial side reactions. Each of the *k_d_* and *k_c_* rate constants depend on the structure of both the nitroxide and the alkyl radicals. The equilibrium constants vary over a wide range depending on the steric and/or electronic effects of substituents in both the nitroxyl [16,17,18,19] and the alkyl parts [19,20,21,22]. In addition, the alkoxyamine homolysis parameters can be varied via the introduction of functional groups capable of reversible protonation [23,24,25], hydrogen bonding [26] or coordination with metals [21,27,28,29] (for example, pyridine or OH groups) in so-called “switchable” alkoxyamines [30,31,32]; another such factor is light in photo-cleavable alkoxyamines [33]. For example, the introduction of a pyridine group into the alkoxyamine produced up to a 30-fold increase or decrease of *k_d_* upon complexation [21,28,29,30].

**Chart 1:**
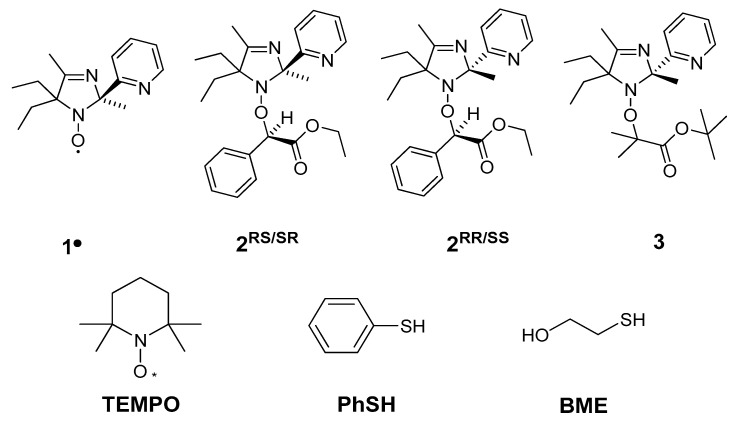
Structures of the nitroxide (**1**^•^), alkoxyamines (**2^RR/SS^**, **2^RS/RS^**, **3**), and scavengers (TEMPO, thiophenol (PhSH), β-mercaptoethanol (BME)).

In the present work, we investigate the homolysis of alkoxyamines composed of imidazoline radicals with a pyridine functional group capable of complexation with metals (Chart 1). Sterically hindered imidazoline radicals have shown their applicability to NMPs [34]. However, the introduction of bulky groups often leads to chiral alkoxyamines, which raises the question of whether the rate *k_d_* and *k_c_* constants can be different for different stereoisomers. In particular, Ananchenko et al. studied tetramethylpiperidine-N-oxyl (TEMPO) and *N*-(2-methylpropan-2-yl)-*N*-(1-diethylphosphono-2,2-dimethylpropyl)-aminoxyl)-based alkoxyamines by ^1^H and ^31^P NMR spectroscopy [35,36,37]. They showed that the diastereomeric excess upon homolysis and reformation of the diastereomeric alkoxyamines depends strongly on the structure of both the nitroxyl and the released alkyl part.

**Scheme 2:**
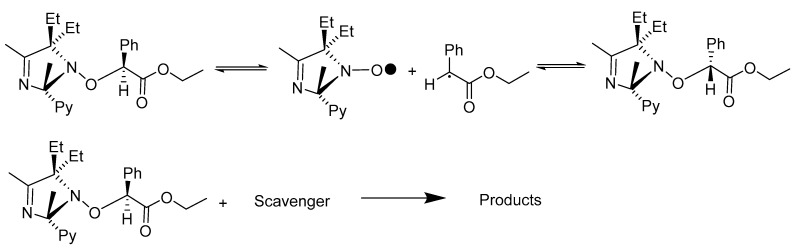
Epimerization and thermolysis in the presence of a scavenger.

The coupling of a chiral nitroxyl radical with a prochiral alkyl radical produces an alkoxyamine exhibiting two chiral centers: one on the alkyl fragment and one on the nitroxyl fragment. In addition, the *N*-atom could be considered as a third pseudo-chiral center because of its three different substituent groups, with its electron lone pair as the fourth group. The chiral nitrogen implies the possibility of configuration inversion at the *N*-atom through a flip-flop or inversion process. These three chiral centers could undergo two independent processes: (a) nitrogen inversion and (b) stereoisomerization of the alkyl part following alkoxyamine homolysis and recombination (Scheme 2). Hereafter, we will refer to this second process as epimerization.

The homolysis of alkoxyamines commonly has a monoexponential time dependence, and consequently, EPR is often used to study the kinetics of the homolysis reaction. In such experiments, the oxygen dissolved in the sample is used as a scavenger for the alkyl radicals, so that the increasing nitroxyl radical concentration reveals the kinetics of the homolysis reaction [38]. However, this approach is not useful for the more complicated, non-monoexponential kinetics caused by side reactions. In such cases, NMR spectroscopy is used to detect and monitor the reaction intermediates and final products [39,40,41]. Here, we used three different scavengers, namely TEMPO, thiophenol (PhSH), and β-mercaptoethanol (BME) (Chart 1), to show the importance of the selection of a radical trap for NMR measurements of different alkoxyamines. We examine how various processes can manifest themselves and show that only a careful analysis by the NMR method allows us to draw conclusions about the processes that take place.

## 2. Results and Discussion

### 2.1. Nitrogen Inversion

**Figure 1:**
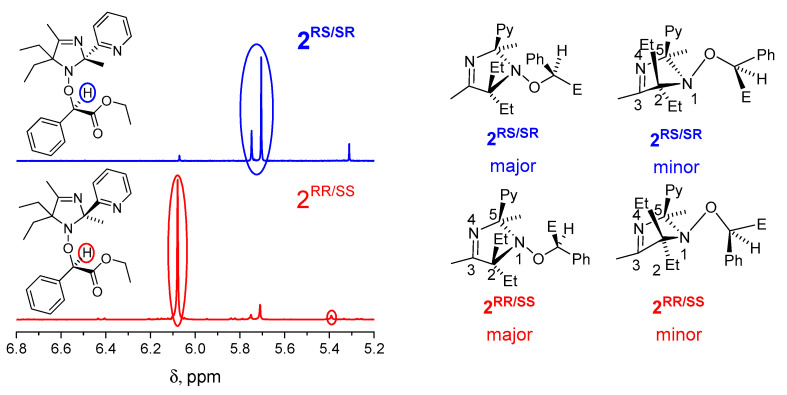
^1^H-NMR spectra in the region of the proton at the tertiary chiral carbon center for the two diastereomers **2^RS/SR^** and **2^RR/SS^**. For each diastereomer, the highlighted regions correspond to the respective invertomers.

Two diastereomers **2^RS/SR^** and **2^RR/SS^** were studied by NMR in the 265–331 K temperature range. Both diastereomers were found to undergo *N*-inversion: for the **2^RS/SR^** diastereomer, the invertomer ratio is 1:3; and for **2^RR/SS^**, the invertomer ratio is 1:30 at room temperature (Figure 1, SI p. S3–S23).

**Figure 2:**
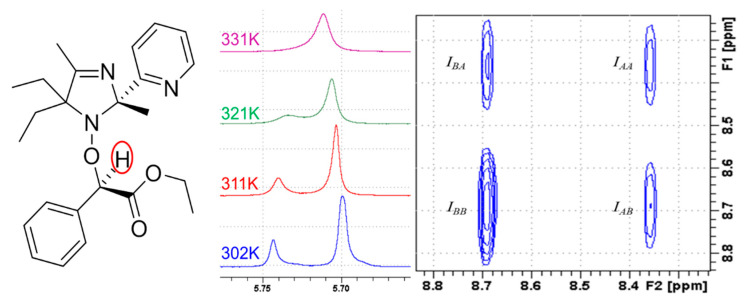
Center—^1^H DNMR spectra of **2^RS/SR^** at different temperatures. Right—fragment of the 2D NOESY/EXSY spectrum of **2^RS/SR^** at 290 K showing the signals from the proton highlighted on the Left.

The nitrogen inversion rate constants were determined in the temperature range 265–311 K by 2D NOESY/Exchange Spectroscopy (EXSY) and at 302-331 K by Dynamic Nuclear Magnetic Resonance (DNMR) for the **2^RS/SR^** diastereomer, which was possible because of the substantial signal from its minor invertomer. Under such conditions, the nitrogen inversion is slow on the NMR time scale; plus, the alkoxyamine epimerization process is negligible and does not affect nitrogen inversion. The signals of protons at tertiary chiral carbon centers from DNMR and cross-peaks of the α-pyridine protons from NOESY/EXSY (Figure 2; SI p. S180) were used.

In the case of NOESY/EXSY, the nitrogen inversion rate constant *k_AB_* is [42]:

(1) $$k_{AB}=\frac{K}{(K+1)\tau }\ln\frac{I_{AA}+KI_{BB}+(K+1)I_{cross}}{I_{AA}+KI_{BB}-(K+1)I_{cross}}$$

where *K* is the equilibrium constant; τ—the mixing time (0.1 s); *I_AA_* and *I_BB_*—the integral intensity of the diagonal peaks; *I_cross_* = (*I_AB_* + *I_BA_*)/2; and *I_AB_* and *I_BA_*—the integral intensity of the cross peaks (Figure 2). For the DNMR line shape analysis, we used the DNMR5 software package [43].

**Figure 3:**
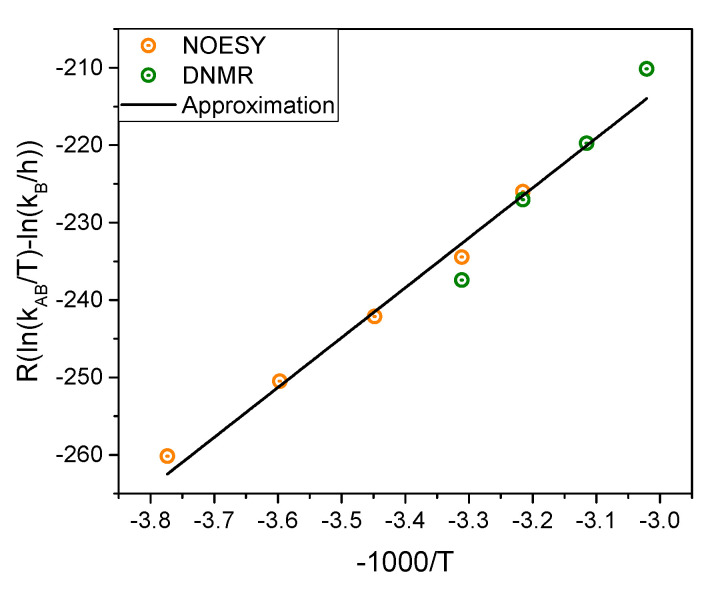
Eyring plot for the nitrogen inversion of the **2^RS/SR^** diastereomer.

Enthalpy and entropy of activation were calculated using the Eyring equation:

(2) $$\Delta G^{\neq }=\Delta H^{\neq }-T\Delta S^{\neq }=-RT(\ln(k_{AB}/T)-\ln(k_{B}/h))$$

where Δ*G^≠^*, Δ*H^≠^*, and Δ*S^≠^* are the Gibbs’ free energy, enthalpy, and entropy of activation, respectively; *k_AB_*—the nitrogen inversion rate constant; *k_B_*—the Boltzmann constant; *h*—Planck’s constant; and R—the universal gas constant (Figure 3, SI p. S180). The transmission coefficient was taken as 1.

Determination of the nitrogen inversion rate constants for alkoxyamine **3** was performed in the temperature range 342–376 K by 2D NOESY/EXSY. The nitrogen inversion is much slower in **3** than in **2^RS/SR^** (0.086/0.36 s^−1^ vs. 48/115 s^–1^ at 342 K in toluene-d_8_ for the rates of the more populated invertomer to/from the less populated invertomer). The ratio of isomers for nitrogen inversion in **3** is 4:1.

**Scheme 3:**
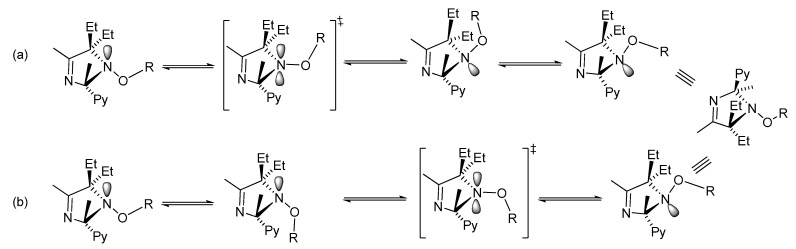
Process involving either (**a**) nitrogen inversion and N–O bond rotation or (**b**) N–O bond rotation and nitrogen inversion and expected transition state (TS) for the nitrogen inversion event.

**Figure 4:**
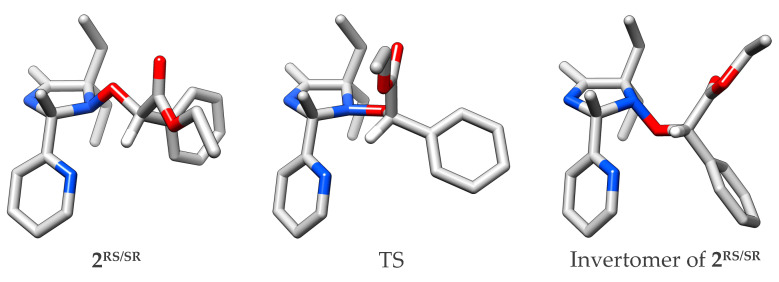
Geometries of the **2^RS/SR^** invertomers and the transition state of nitrogen inversion calculated by Density Functional Theory/Perdew-Birke-Ernzerhof (DFT/PBE)/Λ01.

Due to the specific steric hindrances in alkoxyamines **2** and **3**, nitrogen inversion is always accompanied by a rotation around the N–O bond. The sequence of these two events could follow two alternative processes shown in Scheme 3a,b. However, according to DFT calculations (Figure 4, SI p. S205–S213), planarizing the *N*-atom coordination center and the rotation of the N—O bond do not occur sequentially. The two events are concerted, and the reaction path of nitrogen inversion contains no additional intermediates. The N-O-C fragment turns into the plane of the imidazoline ring in the transition state (TS). An introduction of bulky substituents to the carbon atom of this fragment would make TS less favorable and raise the energy barrier. The steric factor of a methyl group is greater than that of phenyl and especially of a hydrogen atom; therefore, nitrogen inversion in **3**, which contains two methyl groups, should be slower than that in **2^RS/SR^**.

### 2.2. EPR Study of Alkoxyamines Homolysis

**Figure 5:**
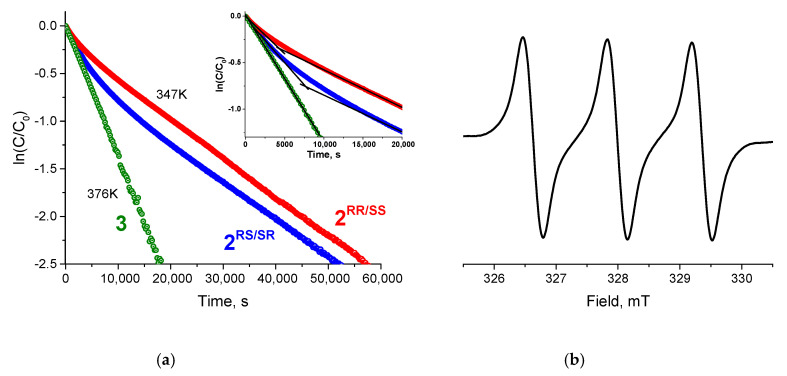
(**a**) Kinetic curves of alkoxyamine decomposition at semi-log scale of **3** at 376 K (green); **2^RS/SR^** (blue) and **2^RR/SS^**(red) at 347K in toluene (left) measured using the second integral of EPR spectrum of formed radical **1**^•^ (right); (**b**) EPR spectrum of formed radical **1**^•^.

Homolysis kinetics were investigated by EPR with oxygen as a scavenger for both diastereomers **2^RS/SR^** and **2^RR/SS^**, as well as for alkoxyamine **3**. As expected, alkoxyamine **3** exhibited monoexponential kinetics; the homolysis rate constant was *k_d_* = 2.5 × 10^−4^ s^−1^ (Figure 5, green). In contrast, for alkoxyamines ***2^RS/SR^*** and ***2^RR/SS^***, bi-exponential kinetics were observed (Figure 5, blue and red), as evident in the upward deviation in concentration of nitroxide at short times. In general, such a dependence is typical of a mixture of diastereomers, such as a mixture of ***2^RS/SR^*** and ***2^RR/SS^***. However, the samples studied here contain only minor impurities of the corresponding diastereomers, which does not account for the deviations from monoexponential decay.

### 2.3. NMR Study of Alkoxyamine Homolysis

As mentioned above, the NMR experiments are affected by two dynamic processes in these alkoxyamines: (*i*) *nitrogen inversion* and (*ii*) *homolysis* of the C–ON bond followed by recombination of the nitroxyl and alkyl radicals giving either of the two diastereomers (*epimerization*). The kinetics of homolysis can be observed by the addition of a radical scavenger to convert the generated radicals (or at least only the alkyl radicals) into diamagnetic products. The epimerization reaction relies on homolysis of the C–ON bond to generate two radicals with a loss of chirality at both N- and C-atoms, but chirality regenerates upon coupling. Thus, the Δ*G*^≠^ of epimerization should be close to the Δ*G*^≠^ of homolysis and larger than 110 kJ/mol. From the little data available in the literature concerning labile alkoxyamines [44,45], the Δ*G*^≠^ of nitrogen inversion is around 60–75 kJ/mol for non-sterically hindered alkoxyamines and close to Δ*G*^≠^ of homolysis when sterically hindered [46].

In ^1^H-NMR spectra, the signals of the α-protons of pyridine moieties are easily recognized by the reduced spin–spin coupling constant with the neighboring proton (≈5 Hz) and are in the low-field region of the spectrum, not overlapping with the signals of other protons. Therefore, in reactions where the pyridine ring is not affected, the total integral of the entire region of α-pyridine protons of the starting materials and products can be taken as unity and used as an “internal standard” in the processing of kinetic data. In addition, in reactions in which the nitroxide radical **1**^•^ is the starting material or the product, the ^1^H spectrum contains two relatively narrow, precisely integrable signals, δ = 7.7 and 8.9 ppm, with a half-width of about 20 Hz and each with an intensity of 1H, which is presumably related to the pyridine fragment. The large chemical shifts difference of the ethyl group protons is noteworthy and is due to the specific spatial structure of this alkoxyamine (SI, p. S10).

### 2.4. Homolysis of Alkoxyamines in the Absence of Scavengers (Epimerization)

The main reaction upon heating of the alkoxyamine solution is the homolysis of the C–ON bond with the formation of a nitroxyl and a planar alkyl radical with a subsequent recombination of these radicals to form one of the diastereomers. In the absence of any alkyl radical traps, including oxygen, the only one side reaction is a recombination of two alkyl radicals. It leads to the accumulation of an excess of the stable nitroxyl radical, forcing the recombination of nitroxyl and alkyl radical to be the main reaction occurring in our experimental conditions [36,37,38,47]. Toluene was chosen as the solvent after careful consideration of the NMR and EPR experiments, taking into account the advantage of using non-polar solvents in EPR experiments on the one hand and the typical activation energy of this reaction (and temperature range) on the other hand.

**Figure 6:**
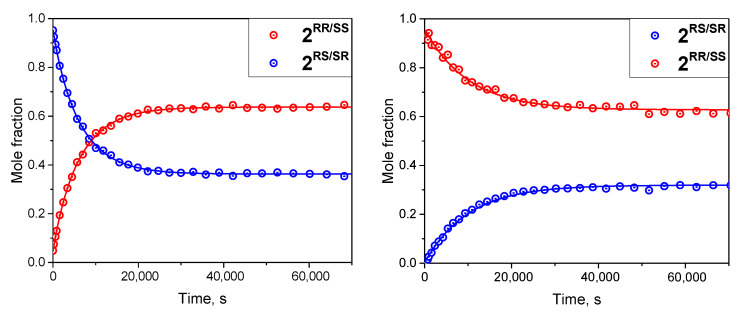
Kinetic curves for the epimerization reaction of alkoxyamines **2^RS/SR^** and **2^RR/SS^**.

Kinetic data were collected for the proton located at the stereogenic carbon center (Figure 1). The kinetic scheme was the conventional reversible conversion **2^RS/SR^** ⇄ **2^RR/SS^** (Figure 6, Table 1).

For the calculation of the energetic parameters of the reaction, a number of experiments at different temperatures were performed (SI, p. S181–S186). The enthalpy and entropy of the reaction were obtained using:

(3) $$\Delta G_{0}=\Delta H_{0}-T\Delta S_{0}=-RT\ln K_{eq}$$

where *K_eq_* is the equilibrium constant of the reaction. The calculated values are Δ*H*_0_ = 0.021 ± 0.046 kJ/mol and Δ*S*_0_ = 2.33 ± 0.33 J/mol/K. Since the equilibrium constant is practically independent of temperature, the enthalpy of the reaction is close to zero.

**Figure 7:**
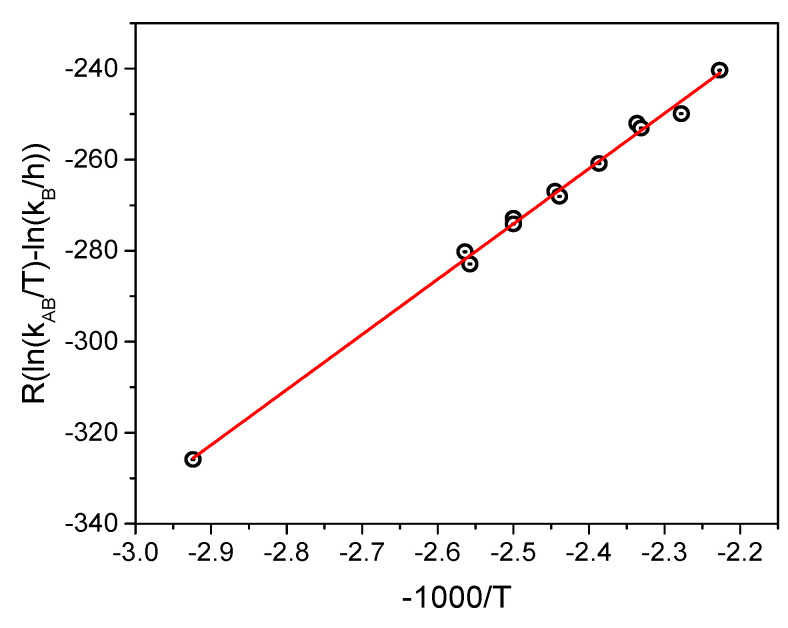
Eyring plot for epimerization reaction of **2^RS/SR^** diastereomer.

In addition, the enthalpy and entropy of activation were calculated for the epimerization reaction using the Eyring equation, in the same way as for nitrogen inversion. Energy parameters of Δ*H*^≠^ = 121.50 ± 2.5 kJ/mol and Δ*S*^≠^ = 29.6 ± 6.2 J/mol/K (Figure 7) were obtained.

### 2.5. Homolysis of Alkoxyamines in the Presence of Scavengers

The decomposition of alkoxyamines **2^RS/SR^**, **2^RR/SS^**, and **3** was studied in the presence of three different scavengers—TEMPO, PhSH, and BME.

**Scheme 4:**
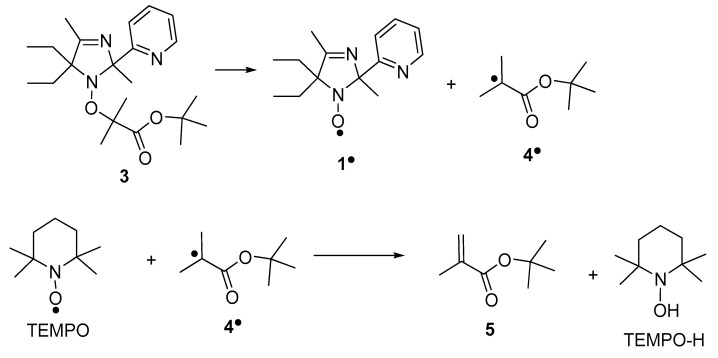
The mechanism of decomposition of alkoxyamine 3 in the presence of TEMPO.

Heating of **3** with TEMPO as an alkyl radical scavenger leads to a complete decomposition of the alkoxyamine (Scheme 4, SI p. S187, S188). TEMPO is not the best scavenger to use in such a case, as TEMPO-based alkoxyamines carrying a tertiary alkyl fragment with a functional group in general exhibit an *E*_a_ lower than 120 kJ/mol[20]. Here, TEMPO is efficient due to the H-abstraction from the tertiary alkyl radical converting it to an olefin [39]. The signals of the reaction products nitroxide **1**^•^ and tert-butylmetacrylate **5** in stoichiometric amounts are present in the NMR spectra (SI p. S39–S43). The absence of signals of the hydroxylamine TEMPO-H in NMR is likely due to the fast exchange—TEMPO + TEMPO-H ⇄ TEMPO-H + TEMPO—on the NMR time scale.

**Scheme 5:**
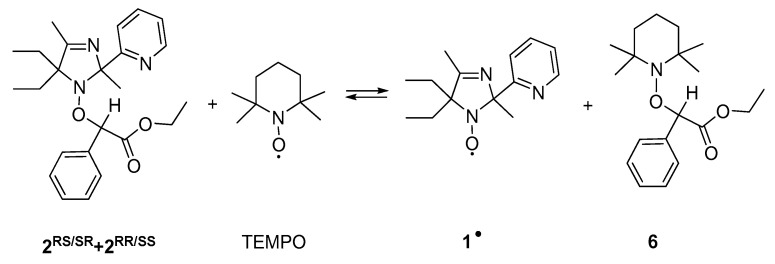
The equilibrium between alkoxyamines **2** and TEMPO.

In sharp contrast to the results observed for **3**, the reaction of **2^RS/SR^** with an excess of TEMPO at 347 K does not reach completion. Even when the experiment is performed at 367 K in the presence of TEMPO for 8 h, decomposition is not complete, and an equilibrium is observed with the epimerization product **2^RR/SS^**. Moreover, a steady-state is also observed between **2^RS/SR^** + **2^RR/SS^** and the TEMPO-based alkoxyamine **6** (SI p. S44–S61). This additional equilibrium (Scheme 5) is established substantially faster than the epimerization of alkoxyamine **2**, its equilibrium constant being 0.3.

Thus, when the *E*_a_ of TEMPO-based alkoxyamines is lower than that of the starting material, the use of TEMPO must comply with the requirement: the presence of a hydrogen at the carbon atom in the α-position to the radical center in the released alkyl radical to favor the conversion of the latter into an olefin.

Unexpectedly, least-squares fitting of the kinetics data for the reversible reaction of **2^RS/SR^** with TEMPO revealed that the rate constants for the direct epimerization **2^RS/SR^** ⇄ **2^RR/SS^** became significantly lower (SI, p. S189). In the presence of TEMPO, the diastereomers of **2** are transformed into each other via the intermediate formation of **6** only. Such unusual behavior could be explained by an unfavorable recombination of chiral **1**^•^ and prochiral **7**^•^ in the opposite diastereomeric configuration, while still in the solvent cavity. Let us assume that the opposite diastereomer can be formed either in the cavity after reorientation of the radicals or after leaving the cavity. Although steric hindrances could prohibit reorientation in the cavity, free radical **7**^•^ out of the cavity would be rapidly scavenged by the more active TEMPO.

**Scheme 6:**
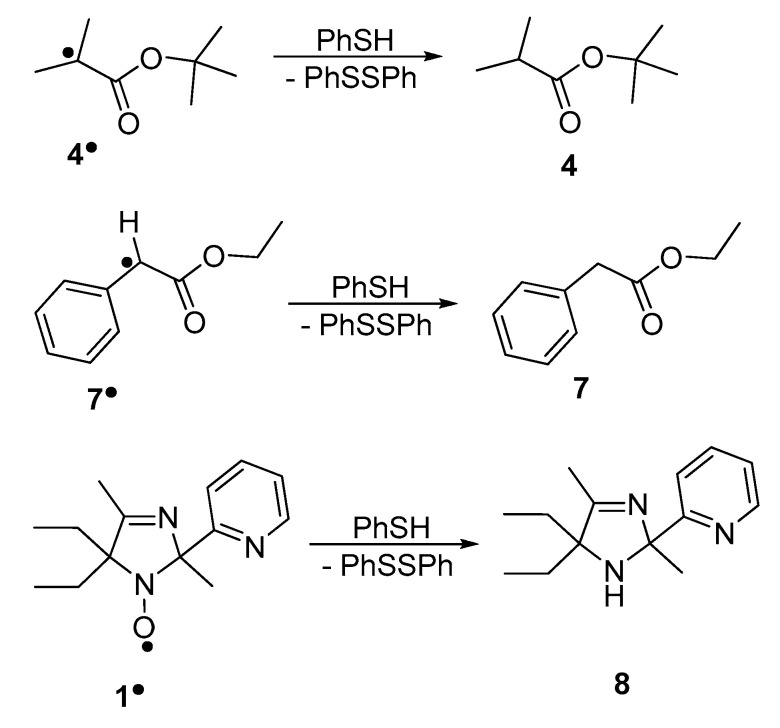
Decomposition of alkoxyamines **2^RS/SR^** and **3** in the presence of PhSH.

**Figure 8:**
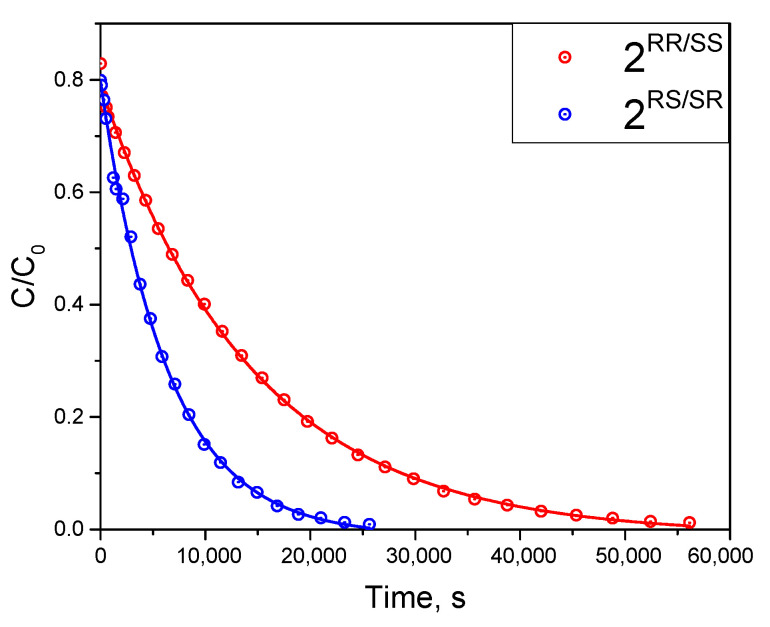
Kinetic curves of alkoxyamines decomposition of **2^RS/SR^** and **2^RR/SS^** at 347 K in toluene-d_8_ in the presence of spin trap PhSH.

In the case of PhSH as a scavenger, monoexponential kinetics were observed (Figure 8) for **2** and **3** (SI p. S190–S193). In contrast to TEMPO, PhSH reduces the radicals **1**^•^, **4**^•^, and **7**^•^ from the homolysis reaction, converting itself into diphenyldisulfide. As the result, alkyl radicals **4**^•^ and **7**^•^ would obtain an additional H-atom from the scavenger, while nitroxide **1**^•^ would be reduced mainly to the corresponding amine **8** (Scheme 6, SI p. S62–S91). The same amine is formed in the reduction of pure nitroxide **1**^•^ with PhSH, with a much faster rate (Table 1, SI p. S92–S113, S194). Additional minor products were also detected by NMR in the reaction mixtures, such as adducts of the nitroxide **1**^•^ with the scavenger.

**Figure 9:**
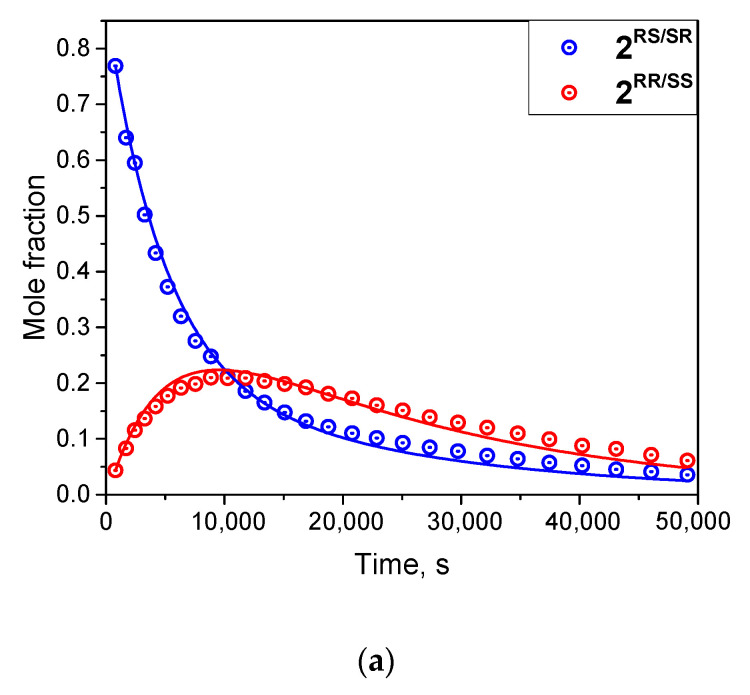

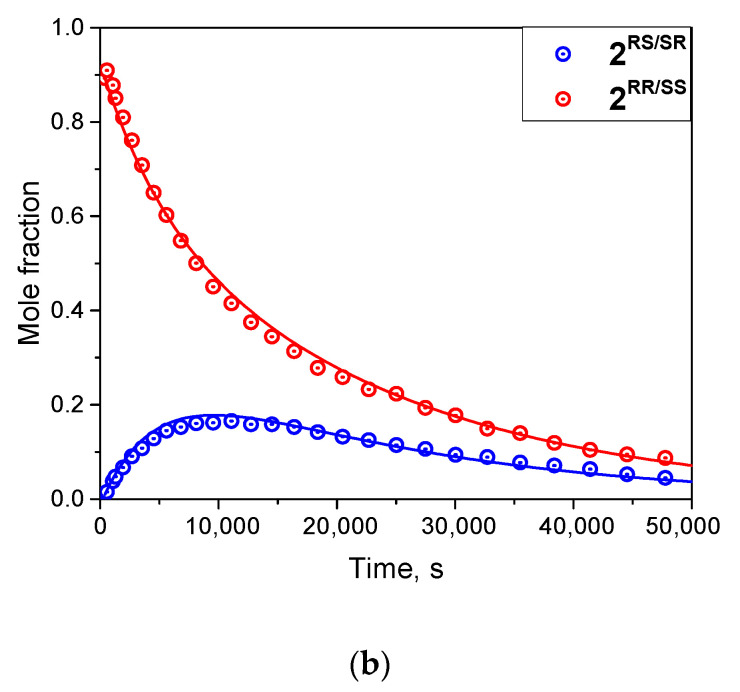
Kinetic curves for decomposition of **2^RS/SR^** and **2^RR/SS^** at 347 K in toluene-d_8_ in the presence of spin trap BME. (**a**) Initial mole fraction of **2^RS/SR^** is equal to 1 while of **2^RR/SS^** is equal to zero. (**b**) Initial mole fraction of **2^RR/SS^** is equal to 1 while of **2^RS/SR^** is equal to zero.

When BME was used as the radical scavenger, bi-exponential kinetics were observed for alkoxyamines **2^RS/SR^** and **2^RR/SS^** (Figure 9), and **3** (SI p. S195–S197). For **2^RS/SR^** and **2^RR/SS^**, the decay was fit with a 4-reaction kinetic scheme:

**2^RS/SR^** → **2^RR/SS^** k_AB_

**2^RR/SS^** → **2^RS/SR^**  k_BA_

**2^RS/SR^** → Products k_AP_

**2^RR/SS^** → Products k_BP_

where k_AB_, k_BA_ are the epimerization rate constants for the two diastereomers; and k_AP_, k_BP_ are the rate constants of the reactions of each diastereomer with BME. To solve the kinetic equations, we assume relationships K = k_AB_/k_BA_ and k_P_ = k_AP_/k_BP_ and obtained the constants (at 347 K): k_AB_ = (8.29 ± 0.58) × 10^−5^ s^−1^, K = 1.33 ± 0.2, k_AP_ = (8.27 ± 6.1) × 10^−5^ s^−1^, and k_P_ = 3.25 ± 0.75.

Our numerous experiments on the destruction kinetics of the alkoxyamines in the presence of BME showed several contradicting but systematically reproducible features which were not observed either with PhSH or with TEMPO (Table 1).

- The rate constants for the reaction of **2^RS/SR^** and **2^RR/SS^** with PhSH are slightly higher than the rate constants for their epimerization. This is not surprising and can be explained by the influence of the different medium with a large excess of the spin trap. In contrast, the rate constants for the reaction of **2^RS/SR^** and especially of **2^RR/SS^** with BME are substantially lower than the epimerization rate constants.
- In contrast to the behavior of **2^RS/SR^** and **2^RR/SS^** (see item above), the rate constant for the reaction of **3** with BME is significantly higher than rate constant for its reaction with PhSH.
- Quite unexpected is the much lower reactivity of **2^RR/SS^** with BME in comparison with its diastereomer **2^RS/SR^**. It can hardly be explained by steric differences in the substrates: the starting diastereomers have very similar energies, and the transition states should be close to the corresponding alkyl and nitroxide radicals, which are identical for both diastereomers.
- The decay rates of the alkoxyamines monotonically increase with increasing BME concentration but do not reach a plateau even at a 100-fold excess of BME.

These unexpected results clearly point out that the reactions of alkoxyamines with PhSH and BME have different mechanisms. It may even be possible that BME reacts with alkoxyamines without a preceding homolysis step. This supposition forced us to perform a thorough NMR study on the products formed in the decomposition of **2^RS/SR^**, **3**, and **1**^•^ in the presence of BME. The structures and the complete NMR signal assignments for all the main components of the reaction mixtures, including assignments for nuclei that differ from each other only in their spatial configuration, were unambiguously determined by 2D NMR (HSQC, HMBC, ^1^H-^15^N HMBC, COSY, NOESY, and DOSY); see SI.

**Chart 2:**
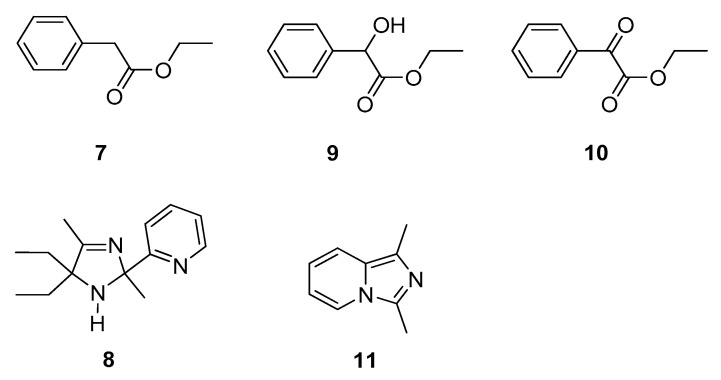
Structures of **7**, **8**, **9**, **10**, **11**.

Decay of **2^RS/SR^** and **2^RR/SS^** with an excess of BME afforded products analogous to those of the reaction with PhSH. However, the reaction with BME proceeds much less selectively (SI p. S114–S138). The alkyl radical **7**^•^ formed by decomposition of the alkoxyamine tends to disproportionate, giving the expected ethyl-2-phenylacetate **7** along with oxidized forms **9** and **10** (**7**:**9**:**10** = 1:0.7:0.9) (Chart 2). The nitroxide radical **1**^•^, as in the case of PhSH, was mainly reduced to the corresponding amine **8** along with minor adducts with BME. Similar adducts of nitroxide radicals with thiols were reported earlier [48]. Surprisingly, a substantial additional product 1,3-dimethylimidazo [1,5-a]pyridine **11** was detected in the reaction mixture (**8**:adducts:**11** = 1:0.6:0.5). The heterocyclic skeleton of **11** is completely different from that of the original alkoxyamine (Chart 2), and even traces of this heterocycle were not observed after the homolysis of **2** with PhSH.

**Chart 3:**
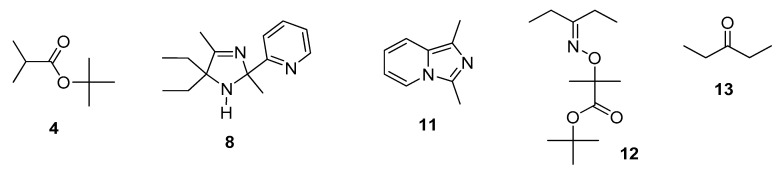
Structures of **4**, **8**, **11**, **12**, **13**.

For the case of decay of alkoxyamine **3** in the presence of BME, tert-butylisobutyrate **4** and amine **8** were also detected in the reaction mixture, as expected. However, the main products of alkoxyamine decay were now the same heterocycle **11**, which was already observed in the case of **2^RS/SR^** and **2^RR/SS^**, and tert-butyl-2-methyl-2-(pentan-3-ylideneaminooxy)propanoate **12** (**11**,**12**:**4**,**8** = 1:0.6) (Chart 3, SI p. S139–S157). It is important to note that the structure of product **12** precludes every mechanism proceeding through homolysis of the substrate to alkyl and nitroxide radicals. Oxime ether **12** can be formed only through rearrangement of the original alkoxyamine **3**. A minor amount of diethylketone **13** was additionally observed, which originated apparently from oxime ether **12**.

A small amount of compound **11** and diethylketone **13** (10%) were detected even in the reaction of nitroxide **1**^•^ with BME, along with the expected major reduction products (SI p. S158–S179, S198). An analogous product mixture was also observed after the thermolysis of alkoxyamines at 150 °C in DMSO or o-dichlorobenzene in the absence of scavengers (SI, p. S199).

Apparently, the essential side reaction of either alkoxyamine **2** or **3**, or nitroxide **1**^•^ with BME breaks the imidazoline ring followed by cyclization with the nitrogen of the pyridine residue. The mechanism of this unexpected rearrangement will be the subject of a more detailed study in the future. However, it is clear that this side reaction complicates the reaction mixtures and significantly shifts the numeric results of kinetics measurements. Instead of the expected alkoxyamine homolysis kinetics, the data reflect some aggregate of parallel homolysis and heterocyclic rearrangement under the influence of BME.

Hence, the choice of scavenger is crucial for a reliable and accurate estimate of the true homolysis rate constant.

Thus, the upward deviation in EPR kinetics obtained with a less efficient scavenger is ascribed to the time needed to reach a pseudo-equilibrium in the reaction. Then, after this pseudo-equilibrium is reached, a mono exponential decay is observed. When the experiment is performed in the presence of a really efficient radical scavenger, free of side processes, such as PhSH (vide infra), a mono-exponential decay in ^1^H NMR and a mono-exponential nitroxide growth in EPR provide the same rate constant as expected.

## 3. Experimental

Routine ^1^H and ^13^C-NMR spectra were recorded on Bruker AV-300, AV-400, and DRX-500 instruments. The structures, including spatial structure, and the complete NMR signal assignments of alkoxyamines and their decomposition products were determined by 2D NMR (COSY, HSQC, HMBC, ^1^H-^15^N HMBC, NOESY, ROESY, and DOSY) on a Bruker Avance-III 600 instrument at 600.30 MHz, 150.96 MHz and 60.85 MHz for ^1^H, ^13^C and ^15^N, respectively. Chemical shifts were measured for ^1^H and ^13^C relative to the residual signals of solvents (CDCl_3_: δ_H_ 7.24 ppm, δ_C_ 76.9 ppm; toluene-d8: δ_H_ 2.13 ppm, δ_C_ 20.1 ppm; DMSO: δ_H_ 2.51 ppm), and for ^15^N relative to an external NH_3_ standard (δ_N_ = 0 ppm).

IR spectra were acquired on an FTIR spectrometer in KBr or neat and are reported in wavenumbers (cm^−1^). Reactions were monitored by TLC using UV light (254 nm) and/or aqueous permanganate for visualization. Column chromatography was performed on silica gel 60, (70–230 Mesh).

### Synthesis (Scheme 7)

*5,5-Diethyl-2,4-dimethyl-2-(pyridin-2-yl)-2,5-dihydro-1H-imidazol 1-oxyl* (**1**•). Synthesis was in analogy to the literature procedures [49]. To a stirred solution of 3-hydroxylamino-3-ethylpentan-2-one hydrochloride (**14**; 2.50 g, 13.8 mmol) in MeOH (20 mL), ammonia acetate (3.18 g, 41.3 mmol) and 2-acetylpyridine (1.87 g, 15.1 mmol) were added. The reaction mixture was stirred at +60 °C for 4 h; then, it was cooled to room temperature and allowed to stand overnight. Then, the mixture was diluted with 20 mL of H_2_O and kept at −10 °C overnight. A crystalline precipitate of *5,5-diethyl-2,4-dimethyl-2-(pyridin-2-yl)-2,5-dihydro-1H-imidazol 1-ol* (**15**) was filtered off and washed with cold H_2_O. To a stirred suspension of **15** in CHCl_3_ (25 mL), MnO_2_ (7 g, 80.5 mmol) was added portion-wise. After the reaction was complete (monitored by TLC analysis; CHCl_3_ + CH_3_OH 20:1), the manganese oxides were filtered off, and the solvent was removed in vacuum to yield radical **1•** as a yellow crystals.

*5,5-diethyl-2,4-dimethyl-2-(pyridin-2-yl)-2,5-dihydro-1H-imidazol 1-ol* (**15**): Yield 60%, colorless crystals, m.p. 187 °C (decomp.). ^1^H-NMR (400 MHz, CDCl_3_ + CD_3_OD): δ = 0.61 (t, 3H, *J* = 7.32 Hz, CH_3_), 1.01 (t, 3H, J = 7.44 Hz, CH_3_), 1.50 (m, 1H, CH_2_), 1.59 (m, 1H, CH_2_), 1.71 (m, 1H, CH_2_), 1.73 (s, 3H, CH_3_), 1.99 (m, 1H, CH_2_), 2.02 (s, 3H, CH_3_), 4.35 (s, 1H, OH), 7.23 (m, 1H, Py), 7.60 (m, 1H, Py), 7.73 (m, 1H, Py), 8.51 (m, 1H, Py). ^13^C-NMR (100 MHz, CDCl_3_ + CD_3_OD): δ = 8.85 (CH_3_CH_2_), 10.74 (CH_3_CH_2_), 26.64 (CH_3_CH_2_), 30.26 (CH_3_CH_2_), 80.86 (CEt_2_), 95.11 (NCN), 121.43, 122.88 (CH, 3-Py and 5-Py), 137.66 (CH, 4-Py), 148.54 (CH, 6-Py), 165.50 (CH, 2-Py), 177.85 (C=N). UV (EtOH): λ_max_ (log ε) = 260 (3.99). IR (neat): 756, 795, 991, 1043, 1095, 1352, 1385, 1431, 1462, 1568, 1581, 1651, 2877, 2904, 2966, 2995, 3047, 3144 cm^−1^. Anal. calcd. for C_14_H_21_N_3_O: C, 67.98; H, 8.56; N, 16.99; found: C, 68.26; H, 8.45; N, 16.93.

*5,5-Diethyl-2,4-dimethyl-2-(pyridin-2-yl)-2,5-dihydro-1H-imidazol 1-oxyl* (**1**•): Yield 90%, yellow crystals, m.p. 51–52 °C (hexane). UV (EtOH): λmax (log ε) = 260 (3.57). IR (neat): 754, 787, 876, 924, 997, 1049, 1095, 1146, 1178, 1257, 1300, 1358, 1387, 1433, 1462, 1572, 1587, 1641, 2879, 2937, 2964, 2978, 3059 cm^−1^. Anal. calcd. for C_14_H_20_N_3_O: C, 68.26; H, 8.18; N, 17.06; found: C, 68.46; H, 8.23; N, 17.25. ^1^H-NMR spectrum (600 MHz): SI, p S24. EPR spectrum: SI, p S217; hyperfine coupling 1.351 mT, g = 2.00608.

**Scheme 7:**
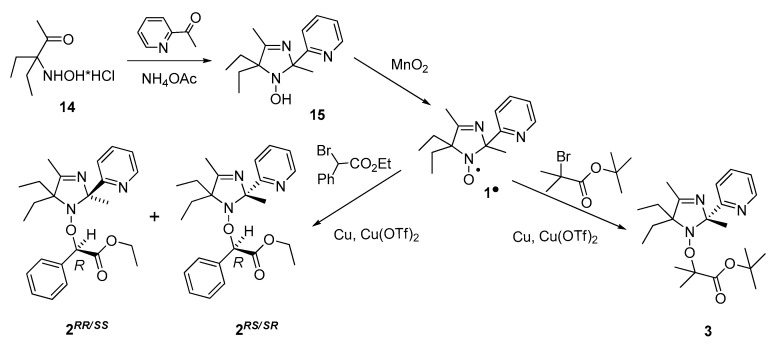
Synthesis of alkoxyamines **2** and **3**.

Synthesis of alkoxyamines 2 and 3 (General procedure). The alkoxyamines were prepared using the method developed by Matyjaszewski et al. [50] (Scheme 7). A mixture of the nitroxide **1**• (2.2 mmol), 2-bromo-2-methylpropionic acid *tert*-butyl ester (2.25 mmol) or 2-bromo-2-phenylacetatic acid ethyl ester (2.25 mmol), Cu powder (140 mg, 2.25 mmol), 4,4’-di-*tert*-butyl-2,2’-bipyridine (24 mg, 0.09 mmol), Cu(OTf)_2_ (8 mg, 0.023 mmol), and benzene (5 mL) was placed into a Schlenk flask and degassed by three freeze–pump–thaw cycles. The solution was stirred for 24 h at 50 °C. Benzene was removed in vacuum, and the residue was separated by column chromatography (silica gel, hexane-diethyl ether 1:6 for 2 and hexane–ethyl acetate 2:1 for 3).

*(R,R)-Ethyl-2-(5,5-diethyl-2,4-dimethyl-2-(pyridin-2-yl)-2,5-dihydro-1H-imidazol-1-yloxy)-2-phenylacetate* (**2^RR/SS^**): Yield 30%, colorless oil. ^1^H-NMR (400 MHz, CDCl_3_): δ = 0.37 (t, 3H, *J* = 7.3 Hz, CH_3_), 1.04 (t, 3H, *J* = 7.3 Hz CH_3_), 1.12 (t, 3H, *J* = 7.3 Hz, CH_3_), 1.48 (ABq, 1H, *J* = 7.3, 14 Hz, CH_2_), 1.67 (ABq, 1H, *J* = 7.3, 14 Hz, CH_2_), 1.73 (s, 3H, CH_3_), 1.81 (ABq, 1H, *J* = 7.3, 14 Hz, CH_2_), 1.98 (s, 3H, CH_3_), 2.47 (ABq, 1H, *J* = 7.3, 14 Hz, CH_2_), 3.97 (ABq, 1H, *J* = 7.0, 17.9 Hz, CH_2_-O), 4.10 (ABq, 1H, *J* = 7.0, 17.9 Hz, CH_2_-O), 6.04 (s, 1H, CH-Ph), 7.11–7.15 (m, 1H, Py), 7.30–7.37 (m, 3H, Ph), 7.38–7.41 (m, 1H, Py), 7.49–7.54 (m, 2H, Ph), 7.55–7.59 (m, 1H, Py), 8.64–8.68 (m, 1H, Py). 13C NMR (CDCl_3_, 125 MHz): δ = 8.75, 10.79 (2*CH_3_, Et), 13.88 (CH_3_, EtO), 16.63 (CH_3_-CNN), 23.59 (CH_3_-C=N), 27.17, 29.71 (2*CH_2_, Et), 60.29 (CH_2_, EtO), 82.01 (CEt_2_), 83.32 (CH-O-N), 96.87 (Py-C-CH_3_), 119.91, 121.55, 127.45, 128.19, 128.29 (Ar), 135.47 (CH, 4-Py), 135.60 (*^i^*Ph), 148.58 (CH, 2-Py), 164.82 (*^i^*Py), 172.51 (C=O), 174.25 (C=N). UV (EtOH): λ_max_ (log ε) = 256 (4.28). IR(neat): 623, 696, 750, 789, 1063, 1095, 1180, 1205, 1273, 1375, 1431, 1454, 1468, 1587, 1664, 1747, 2875, 2935, 2962. Anal. calcd. for C_24_H_31_N_3_O_3_: C, 70.39; H, 7.63; N, 10.26; found: C, 70.49; H, 7.58; N, 10.18. NMR assignments (600 MHz): SI, p S3–S8.

*(R,S)-Ethyl-2-(5,5-diethyl-2,4-dimethyl-2-(pyridin-2-yl)-2,5-dihydro-1H-imidazol-1-yloxy)-2-phenylacetate* (**2^RS/SR^**): Yield 45%, colorless crystals, *m.p.* 81–83 °C (hexane). ^1^H-NMR (400 MHz, CDCl_3_): δ = 0.38 (t, 3H, *J* = 7 Hz, CH_3_), 1.02 (t, 3H, *J* = 7 Hz CH_3_), 1.19 (t, 3H, *J* = 7 Hz, CH_3_), 1.25–1.33 (m, 2H, CH_2_), 1.53 (ABq, 1H, *J* = 7, 14 Hz, CH_2_), 1.82 (ABq, 1H, *J* = 7, 14 Hz, CH_2_), 1.95 (s, 3H, CH_3_), 2.00 (s, 3H, CH_3_), 4.04 (ABq, 1H, *J* = 7, 17.9 Hz, CH_2_-O), 4.13 (ABq, 1H, *J* = 7, 17.9 Hz, CH_2_-O), 5.74 (s, 1H, CH-Ph), 7.18-7.22 (m, 1H, Py), 7.30–7.35 (m, 3H, Ph), 7.36–7.41 (m, 2H, Ph), 7.48–7.53 (m, 1H, Py), 7.61–7.67 (m, 1H, Py), 8.69–8.73 (m, 1H, Py). ^13^C-NMR (100 MHz, CDCl_3_): δ = 8.68, 10.50 (2*CH_3_, Et), 13.80 (CH_3_, EtO), 16.74 (CH_3_-CNN), 23.30 (CH_3_-C=N), 27.42, 29.86 (2*CH_2_, Et), 60.59 (CH_2_, EtO), 81.20 (CEt_2_), 84.81 (CH-O-N), 96.34 (Py-C-CH_3_), 119.84, 121.62, 128.01, 128.07, 128.29 (Ar), 135.62 (CH, 4-Py), 137.37 (*^i^*Ph), 148.53 (CH, 2-Py), 164.99 (*^i^*Py), 170.81 (C=O), 173.47 (C=N). UV (EtOH): λ_max_ (log ε) = 255 (4.27). IR (neat): 623, 696, 752, 791, 1066, 1086, 1176, 1201, 1331, 1371, 1433, 1468, 1585, 1659, 1741, 2874, 2933, 2978 cm^−1^. Anal. calcd. for C_24_H_31_N_3_O_3_: C, 70.39; H, 7.63; N, 10.26; found: C,70.55; H, 7.84; N, 10.29. NMR assignments (600 MHz): SI, p. S9–S23.

*(tert-Butyl-2-((5,5-diethyl-2,4-dimethyl-2-(pyridin-2-yl)-2,5-dihydro-1H-imidazol-1-yl)oxy)-2-methylpropanoate)* (**3**). Yield 84%. Colorless oil. ^1^H NMR (500 MHz, CDCl_3_): δ = 0.71 (t, 3H, *J* = 7.3 Hz, ^Et^CH_3_), 0.98 (t, 3H, *J* = 7.3 Hz ^Et^CH_3_), 0.99 (s, 3H, CH_3_), 1.23 (s, 3H, CH_3_), 1.32 (s, 9H, 3*CH_3_), 1.60 (ABq, 1H, *J* = 7.3, 14 Hz, CH_2_), 1.66-1.77 (m, 2H, CH_2_), 1.86 (s, 3H, CH_3_), 1.93 (s, 3H, CH_3_), 2.00 (ABq, 1H, *J* = 7.3, 14 Hz, CH_2_), 7.04–7.08 (m, 1H, Py), 7.40–7.43 (m, 1H, Py), 7.49–7.54 (m, 2H, Ph), 7.52–7.57 (m, 1H, Py), 8.49–8.52 (m, 1H, Py). ^13^C-NMR (CDCl_3_, 125 MHz): δ = 9.05, 10.80 (2*CH_3_, Et), 17.26 (CH_3_-CNN), 21.73, 24.15, 24.70 (3*CH_3_), 27.35, 28.97 (2*CH_2_, Et), 27.67 (3*CH_3_, tBu), 80.53, 81.12, 81.85 (CMe_2_, CEt_2_, C^t^Bu), 95.70 (Py-C-CH_3_), 121.33, 121.66 (2*CH, Py), 135.45 (CH, 4-Py), 147.74 (CH, 2-Py), 163.94 (*^i^*Py), 173.36, 173.53 (C=O, C=N). NMR assignments (600 MHz): SI, p S25–S38.

UV (EtOH): λ_max_ (log ε) = 256 (3.51).

IR(neat): 750, 788, 848, 1099, 1138, 1160, 1254, 1292, 1367, 1431, 1468, 1572, 1587, 1662, 1728, 2877, 2933, 2978.

Anal. calcd. for C_22_H_35_N_3_O_3_: C, 67.83; H, 9.06; N, 10.79; found: C, 67.69; H, 8.89; N, 10.51.

**Figure 10:**
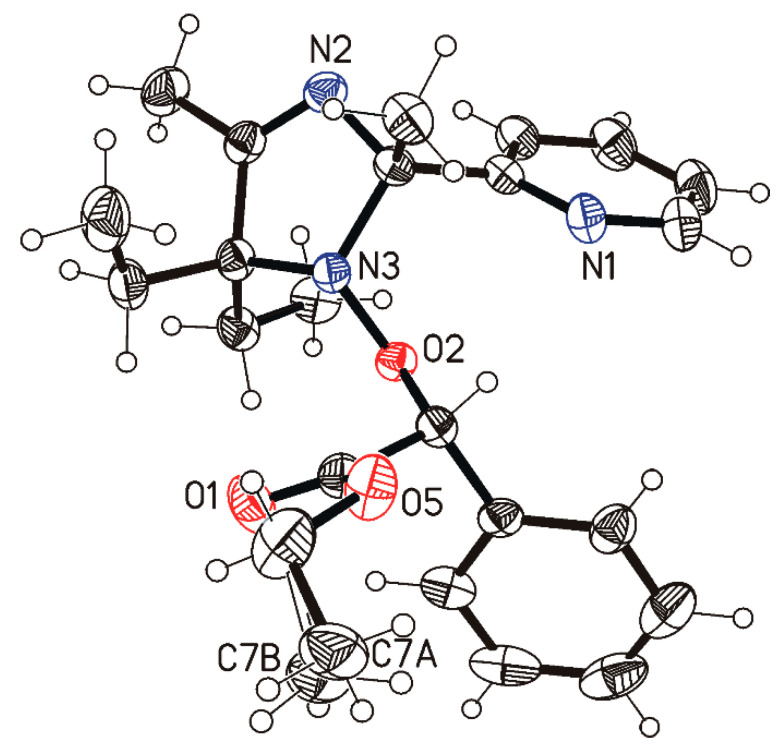
XRD structure of ***2^RS/SR^***. (Displacement ellipsoids are drawn at the 30% probability level).

*XRD analysis of alkoxyamine 2^RS/SR^.* XRD data were obtained at room teperature on a Bruker Kappa Apex II CCD diffractometer using *φ, ω* scans of narrow (0.5°) frames with Mo Kα radiation (λ = 0.71073 Å), and a graphite monochromator. Absorption corrections were applied empirically using *SADABS* programs [51]. The structures were solved by direct methods and refined by full-matrix least-squares method against all *F*^2^ in anisotropic approximation (beside the atoms H) using the *SHELX-97* programs set [52]. The H atoms positions were calculated with the riding model (Figure 10).

Crystallographic data for ***2^RS/SR^***: *T* 296(2) *K*, C_24_H_31_N_3_O_3_, *FW* = 409.52, monoclinic, *P*2_1_/c, *a* = 9.9606(4), *b* = 20.3503(8), *c* = 11.7648(5)Ǻ, *β* = 108.819(2)°, *V* = 2257.3(2)Å^3^, *Z* = 4, *D*_calc_ = 1.205 g cm^−3^, *μ*(Mo-*Kα*) = 0.080 mm^−1^, *F*(000) = 880, 72,240 measured reflections (*θ*_max_ = 27.19°, completeness 99.4%), 5005 independent (*R*_int_ = 0.0388), 285 parameters, *R*_1_ = 0.0616(for 3991 observed *I* > 2σ(*I*)), *wR*_2_ = 0.2000 (all data), *GooF* = 1.03, largest diff. peak and hole 0.61 and −0.47 e.A^−3^. The C7-methyl group is disordered by two positions C7a and C7b with 0.55:0.45(3) occupation ratio.

CCDC 1,829,288 contain the supplementary crystallographic data for this paper. These data can be obtained free of charge via http://www.ccdc.cam.ac.uk/cgi-bin/catreq.cgi, or from the Cambridge Crystallographic Data Centre, 12 Union Road, Cambridge CB2 1EZ, UK; fax: (+44) 1223 336 033; or e-mail: deposit@ccdc.cam.ac.uk.

Molecules ***2^RS/SR^*** adopt conformations that are stabilized by two weak intramolecular hydrogen bonds: one between the nitrogen of the pyridine moiety and the methynic proton (*d*_H····N_ = 2.59, *d*_C····N_ = 3.172(3) Å and <NHC> = 118°) [26,53,54,55,56] and the other one between the *ortho* proton of the pyridyl moiety and the imino function (*d*_H····N_ = 2.44, *d*_C····N_ = 2.777(3) Å and <NHC> = 102°). The intermolecular interactions governing the crystal packing include the π… π interaction of phenyl groups (the separation of molecular planes within the stacks is 3.453(1) and the distance between ring centers Cg…Cg 4.148 (2) Å), which leads to the formation of the centrosymmetric dimers.

*EPR experiments.* All the EPR experiments were performed on the Bruker Elexys E540 X-band spectrometer, using toluene (0.2 mL) as solvent. The concentration of species in all the experiments was 2 × 10^−4^ M. These solutions have been prepared by the dilution method, from the stock solution of 10^−2^ M (1 mL of toluene, 4 mg of alkoxyamine or 2.5 mg of radical **1•**). EPR of radical **1**•: g-factor of radical **1•** was calculated from the reference spectrum of TEMPO, g(TEMPO) = 2.00623 [57]. The EPR spectrum of **1•** was recording at the following parameters: microwave power 2 mW, resolution—1024 points, number of scans 24, conversion time 20.74 ms, modulation amplitude 0.5 G, time constant 20.48 ms. 

*Kinetics of alkoxyamine homolysis by EPR:* to perform the experiments, the oxygen that is in the solvent was used as an alkyl radical trap. The kinetic data were collected as a growing second intergral EPR signal of forming nitroxyl radical. The EPR spectra of all the alkoxyamines were recorded at the following parameters: microwave power 2 mW, resolution—1024 points, conversion time 19.55 ms, modulation amplitude 1 G, time constant 20.48 ms.

*Homolysis of alkoxyamines in the absence of scavengers (Epimerization).* The epimerization reaction was investigated by NMR. The experiments were performed as follows: 0.01 mmol (≈5 mg) of alkoxyamine **2** was solved in 0.58 mL of toluene-d_8_ in NMR tube. The solution was blown with Ar, the tube was sealed with a PTFE cap, and a heat-shrinkable tube. The process of epimerization was carried out at 345 K directly in the probe head of the NMR spectrometer.

*Homolysis of alkoxyamines in the presence of scavengers.* Experiments were performed by the above procedure with 0.50 mL toluene-d_8_ and 1 mmol of scavenger (0.080 mL BME or 0.115 mL PhSH) as the reaction medium.

The experimental kinetic data were evaluated by numerical integration of the differential equations followed by a nonlinear least square fitting of the involved rate constants. The complete processing algorithm was implemented in the form of SciLab[58] script [59] (SI p. S200–S204).

*Quantum chemical calculations*. These were performed using a fast DFT code implemented in the PRIRODA program [60] and employing a PBE functional [61] with full-electron basis Λ01 [62] (similar to the cc-pVDZ basis) as a gas-phase model. The transition states were connected with corresponding minima using the intrinsic reaction coordinate (IRC) procedure. Conformational analysis was performed with the computer program scan from Tinker package (MMFF94 force field) [63] and with the CREST program (GFN2-xTB method) [64]. Then, the geometries of all the conformers were optimized on the DFT/PBE/Λ01 level.

## 4. Conclusions

We synthesized three alkoxyamines based on the imidazoline radical with a pyridine functional group capable of complexation with metals. EPR and NMR were used to investigate the thermolysis of chiral alkoxyamines **2^RR/SS^**, **2^RS/RS^**, and the results were compared with those for non-diastereomeric alkoxyamine **3**. For NMR experiments, we used three different scavengers, namely β-mercaptoethanol, thiophenol, and TEMPO, to show the importance of choosing an appropriate radical trap. For EPR measurements, we used oxygen as a radical scavenger possessing different rate constants with alkyl radicals formed during the decay of alkoxyamines. That revealed that oxygen can produce incorrect results due to its low recombination rate constant with alkyl radicals. The biexponential growth of nitroxide can be obtained if additional processes such as isomerization occur with comparable rates. It is shown that only a careful analysis by NMR provides valid conclusions about the processes that take place. Careful analysis of the thermolysis products of the investigated alkoxyamines differentiated between *N*-inversion, epimerization, and reactions with radical scavengers. Kinetic measurements at different temperatures determined the activation energy of *N*-inversion and the rate constants for homolysis. Additional side reactions of alkoxyamines with BME were detected and identified.

The alkoxyamine chirality affected our experiments with more than a two-fold difference in the homolysis rates for the two diastereomers (Table 1). The influence of alkoxyamine chirality on polymerization needs further study.

## Figures and Tables

**Table 1:** Rate constants (×10^−5^ s^−1^) for the reactions of alkoxyamines (≈0.02 mol/L) with BME (2 mol/L), PhSH (2 mol/L) and TEMPO (3 eqv. ^a^) in toluene-d_8_ at 347 K by NMR.

	None	TEMPO	BME	PhSH
**2^RS/SR^**	10.5 ^b^	5.0 ^c^	8.3	16.3
**2^RR/SS^**	6.0 ^b^	2.2 ^c^	2.5	7.0
**3**		0.33 ^c^	0.81 ^d^	0.36 ^e^
**1** **^•^**			21	6400 ^f^

^a^ A lower concentration of TEMPO was essential to get resolved NMR spectra. ^b^ Epimerization. ^c^ To simplify comparison of rate constants, the initial concentration of the substrate was taken as 1. To obtain the formally correct value (as for bimolecular reaction with TEMPO), the rate constant must be divided by the initial concentration of **2** or **3** (0.033 mol/L), see SI p. S189. ^d^ Interpolated to 347 K from the rates at 342 K and 376 K (the reaction was carried out at a different temperature so that broadening due to nitrogen inversion would be less disturbing). At *T* = 342 K, *k* = 4.1 10^−6^ s^−1^. *T* = 376 K, *k* = 3.1 10^−4^ s^−1^. ^e^ Extrapolated to 347 K from rate at T = 376K, *k*_d_ = 1.9 10^−4^ s^−1^. ^f^ Extrapolated to 347 K from the rates at 300 K by the Eyring equation with Δ*S*^#^ = 0 (the reaction is very fast even at room temperature, incl. at increased speed is not measured).

## Supplementary Materials

The following are available online. NMR and EPR study of Diastereomeric Alkoxyamine’s Homolysis.
