# Supplementary material for: NMR and EPR Study of Homolysis of Diastereomeric Alkoxyamines
Source: Molecules. 2020 Nov 1;25(21):5080. doi: 10.3390/molecules25215080 (PMC7663419; doi:10.3390/molecules25215080)
Supplement: Supplementary file 1 [file molecules-25-05080-s001.pdf]

NMR and EPR study of Diastereomeric Alkoxyamine's Homolysis

Sergey Cherkasov[a,b], Dmitriy Parkhomenko[a], Alexander Genaev[a], Georgii Salnikov[a], Mariya Edeleva[a], Denis Morozov[a], Tatyana Rybalova[a], Igor Kirilyuk[a], Sylvain R. A. Marque[c], Elena Bagryanskaya[a]*

[a] N. N. Vorozhtsov Novosibirsk Institute of Organic Chemistry SB RAS, Pr. Lavrentjeva 9, 630090 Novosibirsk, Russia.
[b]National Research University – Novosibirsk State University, Novosibirsk 630090, Russia.
[c] Aix Marseille Univ, CNRS, ICR, UMR 7273, case 551, Avenue Escadrille Normandie-Niemen, 13397 Marseille Cedex 20 France.

Supporting Information

Table of contents

NMR spectra of diastereomer $2^{RR/SS}$ 3

Signal assignments 3
^{1}H NMR spectrum (600 MHz) 4
^{13}C{^{1}H} NMR spectrum (150 MHz) 7

NMR spectra of diastereomer $2^{RS/SR}$ 9

Signal assignments 9
Influence of spatial structure on ^{1}H chemical shifts of ethyl groups 10
^{1}H NMR spectrum (600 MHz) 11
^{13}C{^{1}H} NMR spectrum (150 MHz) 17

^{1}H NMR spectrum of radical $1^{\bullet}$ in toluene-d_8 at 108 °C 24
NMR spectra of alkoxyamine 3 25

Signal assignments 25
^{1}H NMR spectrum (600 MHz) 27
^{13}C{^{1}H} NMR spectrum (150 MHz) 32

Structures and NMR signal assignments for products in the reaction mixture 3 + TEMPO 39

Signal assignments 39
^{13}C{^{1}H} NMR spectrum (150 MHz) 40
^{1}H NMR spectrum (600 MHz) 42

Structures and NMR signal assignments for products in the reaction mixture $2^{RS/SR}$ + TEMPO 44

Signal assignments 44
^{13}C{^{1}H} NMR spectrum (150 MHz) 46
^{1}H NMR spectrum (600 MHz) 58

Structures and NMR signal assignments for products in the reaction mixture $2^{RS/SR}$ +PhSH 62

Signal assignments 62
^{13}C{^{1}H} NMR spectrum (150 MHz) 64
^{1}H NMR spectrum (600 MHz) 70

Structures and NMR signal assignments for products in the reaction mixture 3 + PhSH........ 77

Signal assignments 77
^{13}C{^{1}H} NMR spectrum (150 MHz) 79
^{1}H NMR spectrum (600 MHz) 86

Structures and NMR signal assignments for products in the reaction mixture $1^{\bullet}$ + PhSH 92

Signal assignments 92
^{13}C{^{1}H} NMR spectrum (150 MHz) 96
^{1}H NMR spectrum (600 MHz) 105
^{15}N NMR spectrum........ 112

Structures and NMR signal assignments for products in the reaction mixture $2^{RS/SR}$ + BME 114

Signal assignments 114
^{13}C{^{1}H} NMR spectrum (150 MHz) 119
^{1}H NMR spectrum (600 MHz) 130

Structures and NMR signal assignments for products in the reaction mixture 3 + BME 139

Signal assignments 139

$^{13}C\{^1H\}$ NMR spectrum (150 MHz) 142
^1H NMR spectrum (600 MHz) 151

Structures and NMR signal assignments for products in the reaction mixture $1^\bullet$ + BME 158

Signal assignments 158
$^{13}C\{^1H\}$ NMR spectrum (150 MHz) 162
^1H NMR spectrum (600 MHz) 170
^{15}N NMR spectrum 178

Inversion of nitrogen, $2^{RS/SR}i \rightleftarrows 2^{RS/SR}$ (A⇄B) in $CDCl_3$ 180
Epimerization of diastereomers $2^{RS/SR}$ (A) and $2^{RR/SS}$ (B) in DMSO-d_6 181

Rate constants from NOE at 117÷176 °C 181
Kinetics A□B ($2^{RS/SR}$□$2^{RR/SS}$) at 69 °C in DMSO-d_6 182
Kinetics A□B ($2^{RS/SR}$□$2^{RR/SS}$) at 69 °C in toluene-d_8 183
Choice of fitted parameters for A□B kinetics 184
Thermodynamic and activation parameters of A→B ($2^{RS/SR} \rightarrow 2^{RR/SS}$) reaction in DMSO-$d_6$ 185
Kinetics A□B ($2^{RS/SR}$□$2^{RR/SS}$) at 74 °C in toluene-d_8 186

Kinetics 3 + TEMPO at 74 °C in toluene-d_8 187
Kinetics 3 + TEMPO at 103 °C in toluene-d_8 188
Kinetics $2^{RS/SR}$ + TEMPO at 74 °C in toluene-d_8 189
Kinetics $2^{RS/SR}$ + PhSH at 74 °C in toluene-d_8 190
Kinetics $2^{RR/SS}$ + PhSH at 74 °C in toluene-d_8 191
Kinetics 3 + PhSH at 74 °C in toluene-d_8 192
Kinetics 3 + PhSH at 103 °C in toluene-d_8 193
Kinetics $1^\bullet$ + PhSH at 27 °C in toluene-d_8 194
Kinetics 3 + BME at 69 °C in toluene-d_8 195
Kinetics 3 + BME at 103 °C in toluene-d_8 196
Kinetics $2^{RS/SR}$ (RS)+BME and $2^{RR/SS}$ (RR)+BME at 74 °C in toluene-d_8 197
Kinetics $1^\bullet$ + BME at 74 °C in toluene-d_8 198
Kinetics of decomposition of alkoxyamine 2 in DMSO-d_6 199
SciLab script for A□B kinetics 200
Quantum chemical calculations 205

The most stable conformers $2^{RS/SR}$, $2^{RS/SR}i$, $2^{RR/SS}$, $2^{RR/SS}i$ 205
Transition state of $2^{RS/SR}$-$2^{RS/SR}i$ NO inversion 210
The most stable conformers 3, 3i 211
Transition state of 3-3i NO inversion 213
DFT “transition state” of $2^{RS/SR}$-$2^{RR/SS}$ epimerization 214

EPR spectrum of $1^\bullet$ 205

NMR spectra of diastereomer $2^{RR/SS}$

in $CDCl_3$ at 25 °C

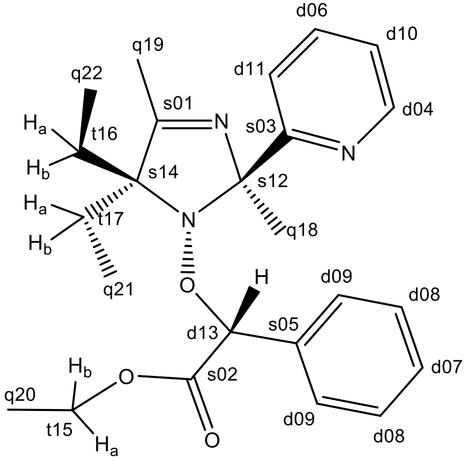

Signal assignments

Experiment Bruker_2, 1D 13C

s01 174.3
s02 172.5
s03 164.8
d04 148.6
s05 135.6
d06 135.5
d07 128.3
d08 128.2 (*2)
d09 127.5 (*2)
d10 121.6
d11 119.9
s12 96.9
d13 83.4
s14 82.0
t15 60.3
t16 29.7
t17 27.2
q18 23.6
q19 16.6
q20 13.9
q21 10.8
q22 8.7

Experiment Bruker_1, 1D 1H

d04-H 8.65
d06-H 7.56
d07-H 7.31
d08-H 7.33 (*2)
d09-H 7.51 (*2)
d10-H 7.12
d11-H 7.39
d13-H 6.03
t15-a 3.95
t15-b 4.09
t16-a 1.46
t16-b 1.80
t17-a 1.66
t17-b 2.46
q18-H 1.71
q19-H 1.97
q20-H 1.10
q21-H 1.02
q22-H 0.36

Experiment Bruker_5, 2D 13C-1H via onebond (HSQC)

d04-H - d04
d06-H - d06
d07-H - d07
d08-H - d08
d09-H - d09
d10-H - d10
d11-H - d11
d13-H - d13
q18-H - q18
q19-H - q19
q20-H - q20
q21-H - q21
q22-H - q22
t15-a - t15
t15-b - t15
t16-a - t16
t16-b - t16
t17-a - t17
t17-b - t17

Experiment Bruker_4, 2D 1H-1H via Jcoupling (COSY)

d04-H - d10-H
d06-H - d10-H d11-H
d08-H - d09-H
d09-H - d08-H
d10-H - d04-H d06-H
d11-H - d06-H
q20-H - t15-a t15-b
q21-H - t17-a t17-b
q22-H - t16-a t16-b
t15-a - q20-H t15-b
t15-b - q20-H t15-a
t16-a - q22-H t16-b
t16-b - q22-H t16-a
t17-a - q21-H t17-b
t17-b - q21-H t17-a

Experiment Bruker_6, 2D 13C-1H via Jcoupling (HMBC)

d04-H - d06 d10(weak) s03
d06-H - d04 s03
d07-H - d09
d08-H - d08 s05
d09-H - d07 d13
d10-H - d04(weak) d11
d11-H - d06(weak) d10 s12
d13-H - d09 s02 s05
q18-H - s03 s12
q19-H - s01 s14
q20-H - t15
q21-H - s14 t17
q22-H - s14 t16
t15-a - q20 s02
t15-b - q20 s02
t16-a - q22 s14
t16-b - q22 s01 s14 t17
t17-a - q21 s01(weak) s14 t16
t17-b - q21 s01 s14 t16

Experiment Bruker_7, 2D 1H-1H via through-space (NOESY)

d09-H - d13-H
d11-H - q22-H?
d13-H - d09-H q18-H
q18-H - d13-H q21-H?
q19-H - q21-H q22-H t16-a? t17-a?
q21-H - q19-H
q22-H - q19-H

^{1}H NMR spectrum (600 MHz)

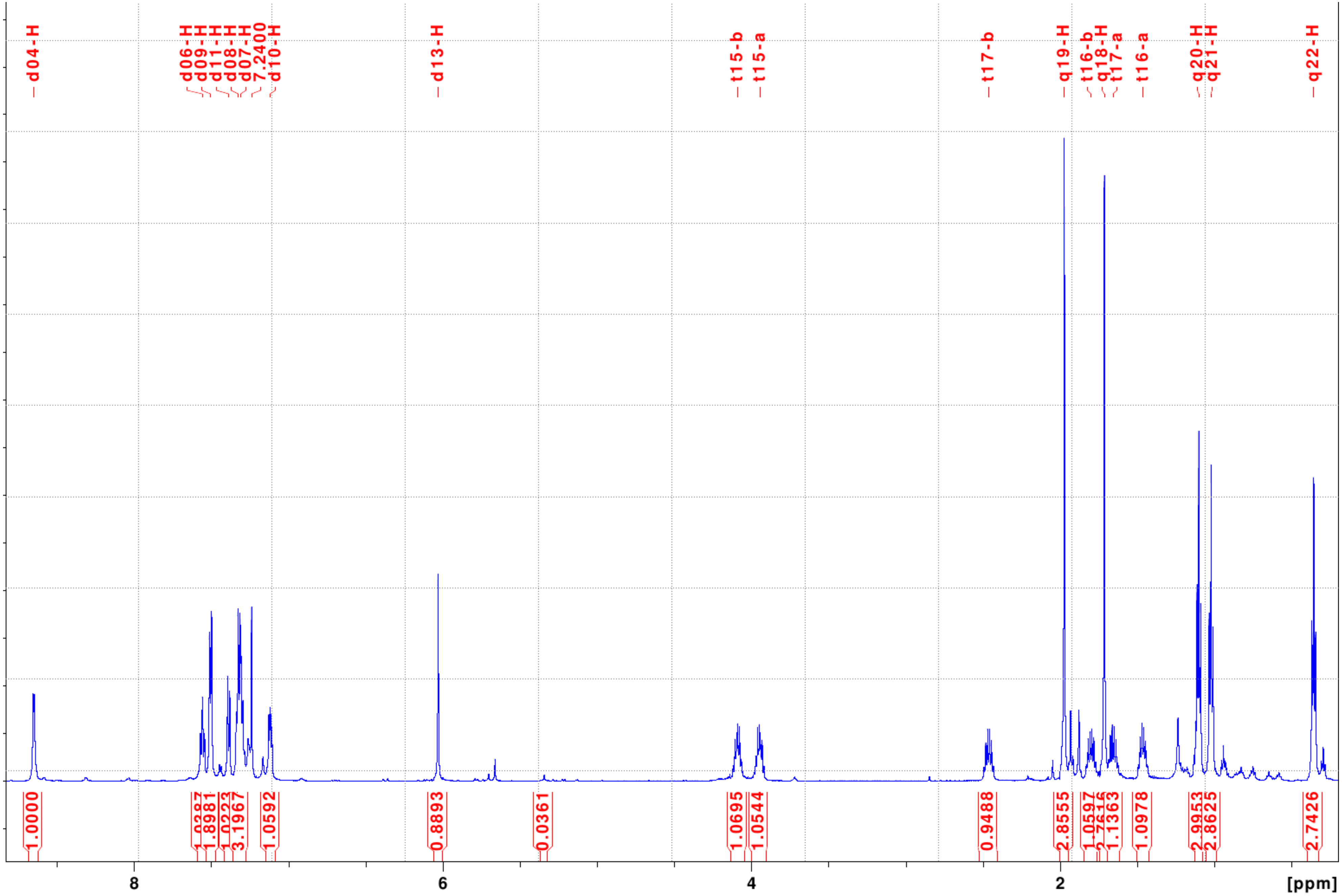

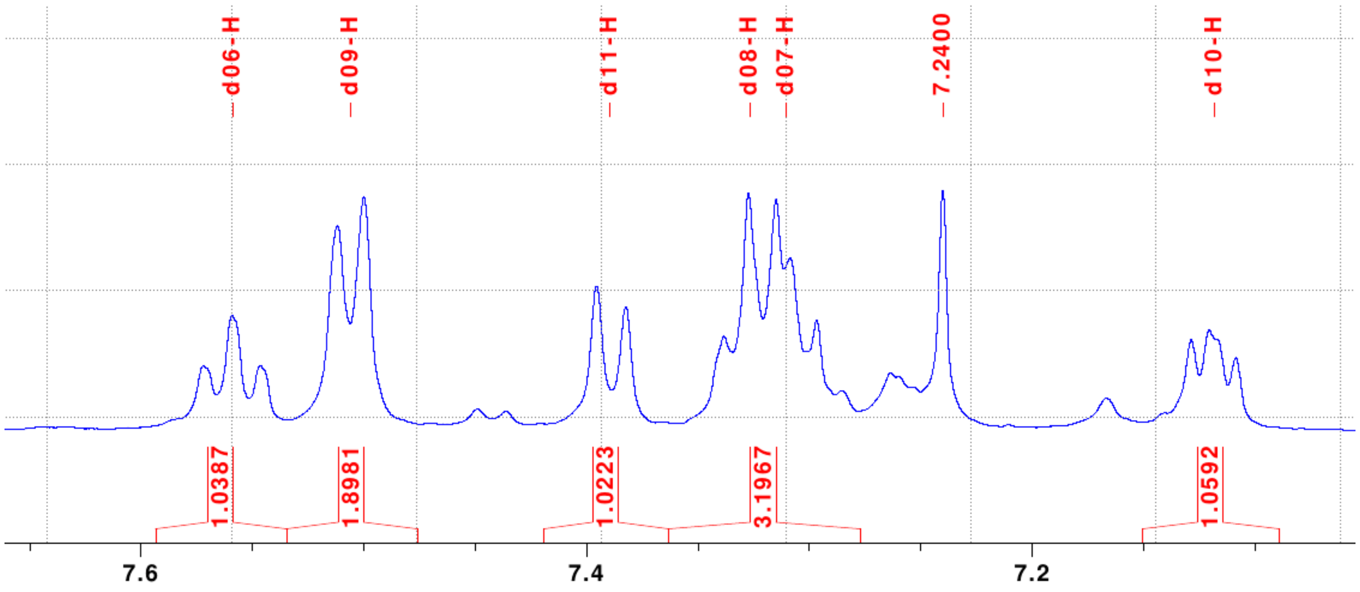
d06-H
d09-H
d11-H
d08-H
d07-H
7.2400
d10-H
1.0387
1.8981
1.0223
3.1967
1.0592
7.6
7.4
7.2

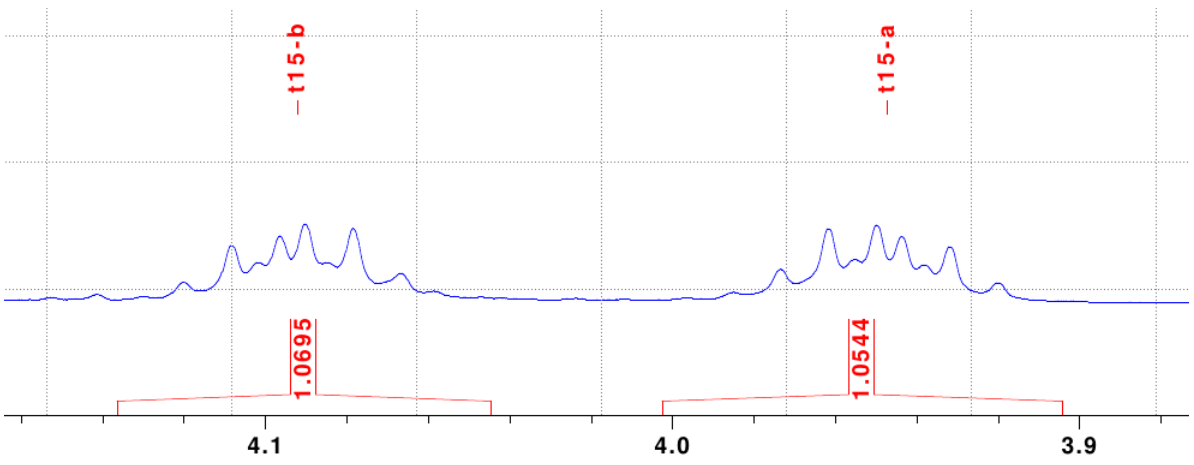
t15-b
t15-a
1.0695
1.0544
4.1
4.0
3.9

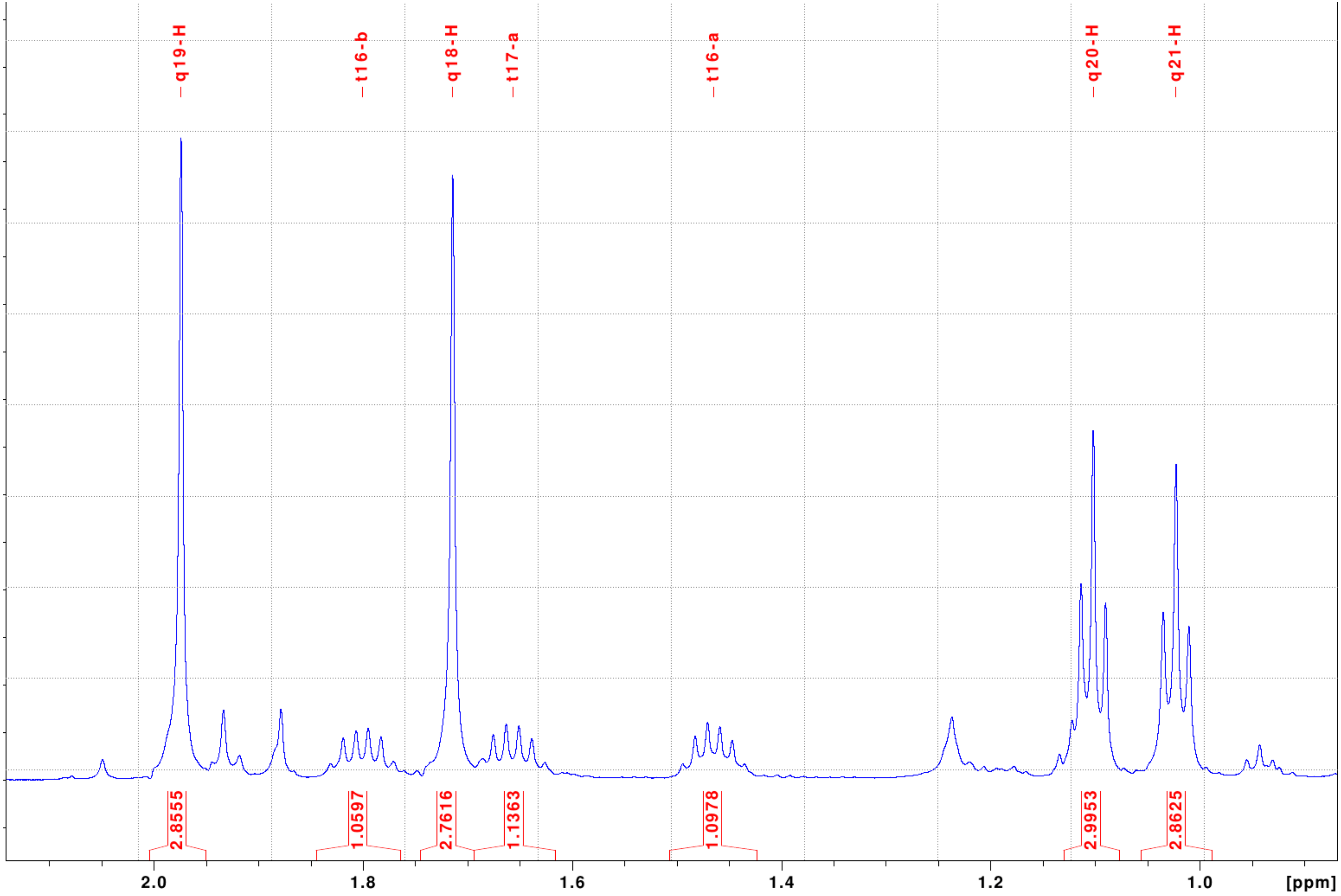
q19-H
t16-b
q18-H
t17-a
t16-a
q20-H
q21-H
2.8555
1.0597
2.7616
1.1363
1.0978
2.9953
2.8625
2.0
1.8
1.6
1.4
1.2
1.0
[ppm]

$^{13}C\{^1H\}$ NMR spectrum (150 MHz)

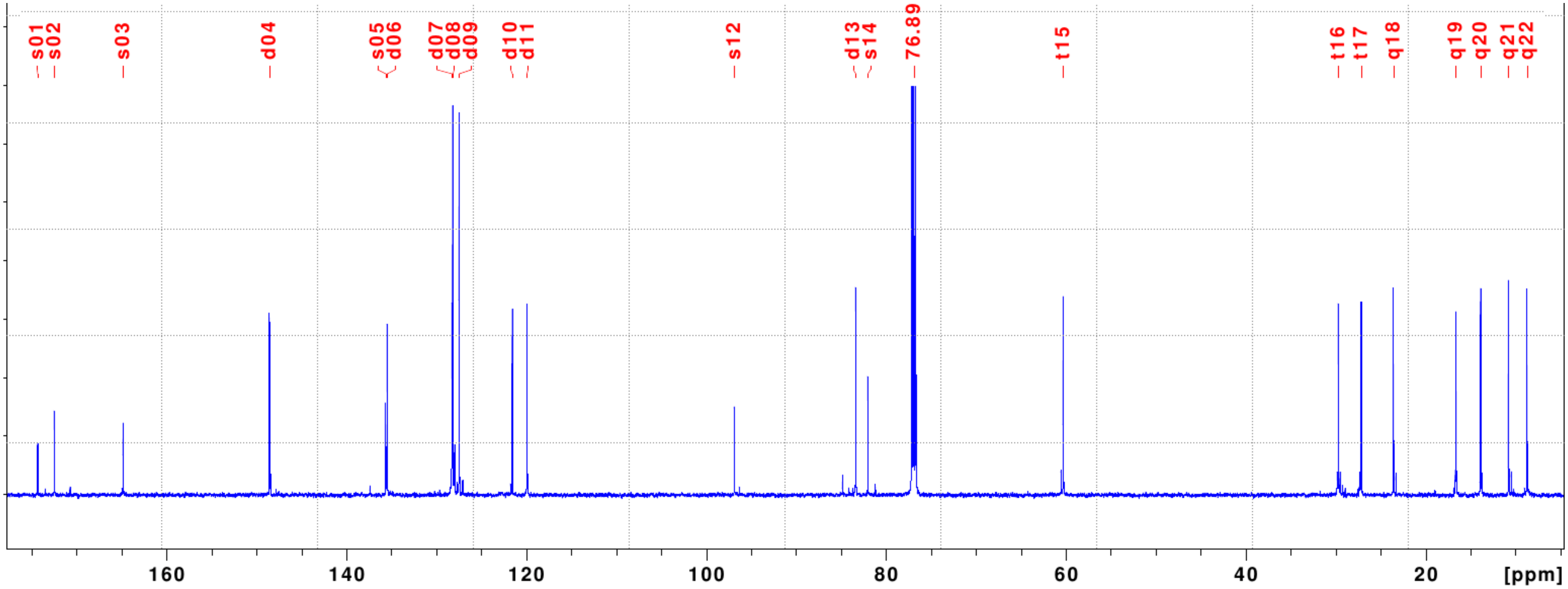

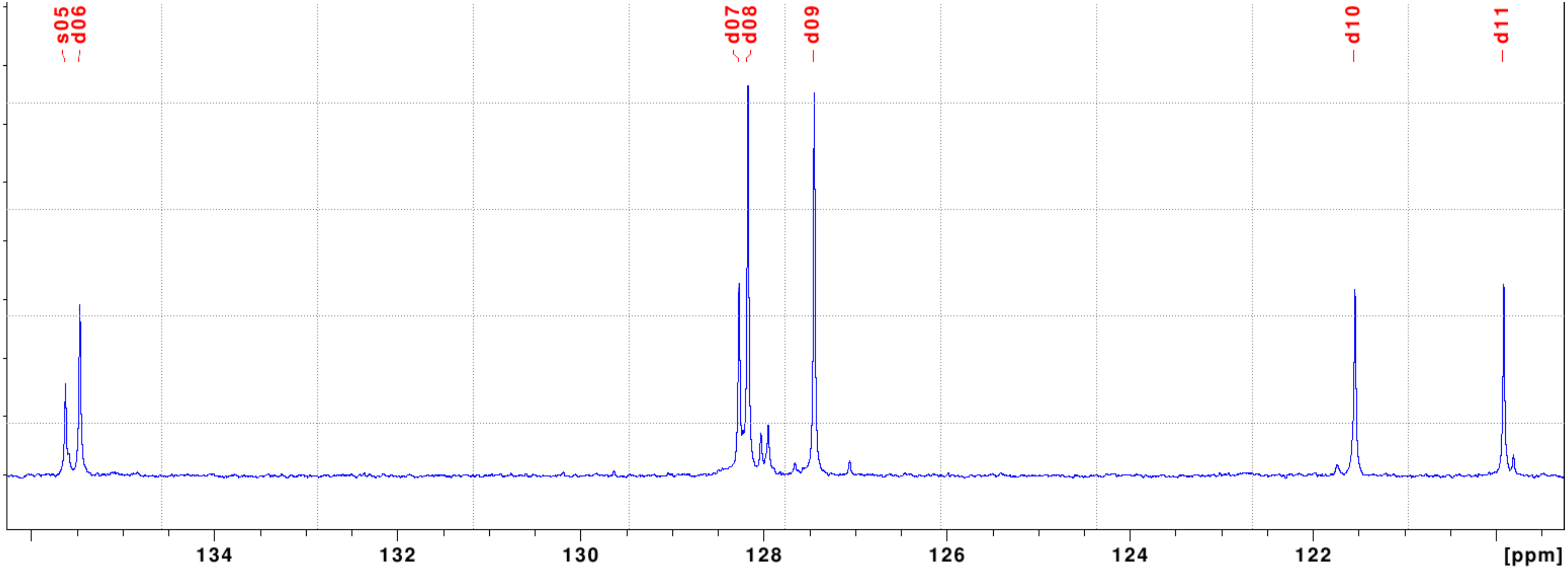

s05
d06
d07
d08
d09
d10
d11
134
132
130
128
126
124
122
[ppm]

NMR spectra of diastereomer 2RS/SR

(equilibrium mixture of nitrogen inversion conformers) in $CDCl_3$ at 25 °C

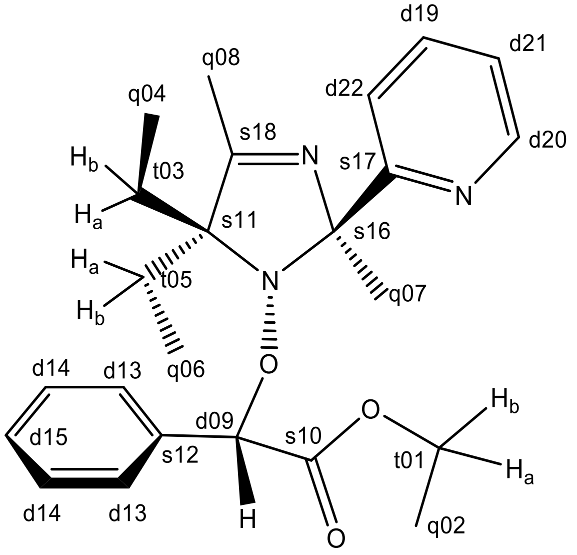

RS (**A** on spectra), 75%

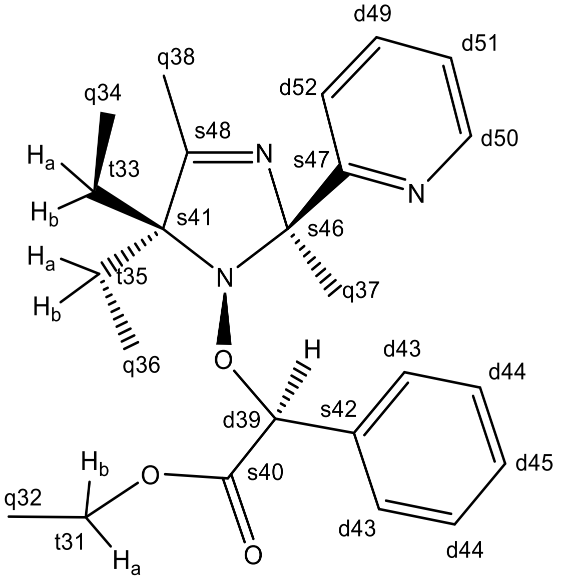

RSi (**B** on spectra), 25%

Signal assignments

Experiment Bruker_1, 1D 13C

A:t01 60.6	B:t31 60.6
A:q02 13.9	B:q32 14.1
A:t03 29.9	B:t33 27.5
A:q04 8.7	B:q34 10.9
A:t05 27.4	B:t35 29.0
A:q06 10.5	B:q36 9.1
A:q07 23.4	B:q37 29.8
A:q08 16.8	B:q38 16.8
A:d09 84.9	B:d39 83.8
A:s10 170.8	B:s40 172.5
A:s11 81.3	B:s41 82.1
A:s12 137.4	B:s42 135.2
A:d13 128.1 (*2)	B:d43 127.2 (*2)
A:d14 128.1 (*2)	B:d44 127.8 (*2)
A:d15 128.3	B:d45 128.0
A:s16 96.4	B:s46 96.7
A:s17 165.0	B:s47 159.7
A:s18 173.5	B:s48 175.9
A:d19 135.7	B:d49 134.9
A:d20 148.5	B:d50 147.9
A:d21 121.7	B:d51 121.8
A:d22 119.9	B:d52 121.8

Experiment Bruker_10, 1D 1H

A:t01-a 3.97	B:t31-a 4.04
A:t01-b 4.07	B:t31-b 4.21
A:q02-H 1.12	B:q32-H 1.24
A:t03-a 0.75	B:t33-b 2.19
A:t03-b 1.21	B:t33-a 1.60
A:q04-H 0.30	B:q34-H 0.75
A:t05-a 1.46	B:t35-a 1.53
A:t05-b 1.75	B:t35-b 1.93
A:q06-H 0.94	B:q36-H 0.82
A:q07-H 1.93	B:q37-H 1.92
A:q08-H 1.88	B:q38-H 2.05
A:d09-H 5.66	B:d39-H 5.70
A:d13-H 7.31 (*2)	B:d43-H 7.17
A:d14-H 7.26 (*2)	B:d44-H 7.17
A:d15-H 7.26	B:d45-H 7.17
A:d19-H 7.58	B:d49-H 7.44
A:d20-H 8.64	B:d50-H 8.31
A:d21-H 7.14	B:d51-H 6.92
A:d22-H 7.44	B:d52-H 7.24

Experiment Bruker_5, 2D 13C-1H via onebond (HSQC)

A:d09-H - d09	B:d39-H - d39
A:d13-H - d13	B:d44-H - d44
A:d14-H - d14?	B:d49-H - d49
A:d15-H - d15?	B:d50-H - d50
A:d19-H - d19	B:d51-H - d51
A:d20-H - d20	B:d52-H - d52
A:d21-H - d21	B:q32-H - q32
A:d22-H - d22	B:q34-H - q34
A:q02-H - q02	B:q36-H - q36
A:q04-H - q04	B:q37-H - q37
A:q06-H - q06	B:q38-H - q38
A:q07-H - q07	B:t31-a - t31
A:q08-H - q08	B:t31-b - t31
A:t01-a - t01	B:t33-a - t33
A:t01-b - t01	B:t33-b - t33
A:t03-a - t03	B:t35-a - t35
A:t03-b - t03	B:t35-b - t35
A:t05-a - t05	
A:t05-b - t05	

Experiment Bruker_9, 2D 1H-13C via onebond (H-C correlation)

A:d09 - d09-H	B:d39 - d39-H
A:d13 - d13-H	B:d43 - d43-H
A:d14 - d14-H	B:d44 - d44-H
A:d15 - d15-H	B:d45 - d45-H
A:d19 - d19-H	B:d49 - d49-H
A:d20 - d20-H	B:d50 - d50-H
A:d21 - d21-H	B:d51 - d51-H
A:d22 - d22-H	B:d52 - d52-H
A:q02 - q02-H	B:q32 - q32-H
A:q04 - q04-H	B:q34 - q34-H
A:q06 - q06-H	B:q36 - q36-H
A:q07 - q07-H	B:q37 - q37-H
A:q08 - q08-H	B:q38 - q38-H
A:t01 - t01-a t01-b	B:t33 - t33-a t33-b
A:t03 - t03-a t03-b	B:t35 - t35-b
A:t05 - t05-a t05-b	

Experiment Bruker_4, 2D 1H-1H via Jcoupling (COSY)

A:d13-H - d14-H	B:d49-H - d51-H d52-H
A:d14-H - d13-H	B:d50-H - d51-H
A:d19-H - d21-H d22-H	B:d51-H - d49-H d50-H
A:d20-H - d21-H	B:d52-H - d49-H

A:d21-H - d19-H d20-H   B:q32-H - t31-a t31-b
A:d22-H - d19-H   B:q34-H - t33-a t33-b
A:q02-H - t01-a t01-b   B:q36-H - t35-a t35-b
A:q04-H - t03-a t03-b   B:t31-a - q32-H t31-b
A:q06-H - t05-a t05-b   B:t31-b - q32-H t31-a
A:t01-a - q02-H t01-b   B:t33-a - q34-H t33-b
A:t01-b - q02-H t01-a   B:t33-b - q34-H t33-a
A:t03-a - q04-H t03-b   B:t35-a - q36-H t35-b
A:t03-b - q04-H t03-a   B:t35-b - q36-H t35-a
A:t05-a - q06-H t05-b
A:t05-b - q06-H t05-a

Experiment Bruker_6, 2D 13C-1H via Jcoupling (HMBC)
A:d09-H - d13 s10 s12   B:d39-H - d43 s40 s42
A:d13-H - d09 d15   B:d43-H - d39 d43 d45
A:d14-H - d13? d14? s12   B:d44-H - s42
A:d15-H - d13? d14?   B:d45-H - d43
A:d19-H - d20 d22 s17   B:d49-H - d50
A:d20-H - d19 d21 s17   B:d50-H - d49
A:d21-H - d20 d22   B:d52-H - d51
A:d22-H - d19 d21   B:q32-H - t31
A:q02-H - t01   B:q34-H - s41 t33
A:q04-H - s11 t03   B:q36-H - s41 t35
A:q06-H - s11 t05   B:q37-H - s46 s47
A:q07-H - s16 s17   B:q38-H - s41 s48
A:q08-H - s11 s18   B:t31-b - q32 s40
A:t01-a - q02 s10   B:t33-a - q34
A:t01-b - q02 s10   B:t33-b - q34 s41
A:t03-a - q04 s11 s18 t05   B:t35-a - q36
A:t03-b - q04 s11(weak) t05(weak)   B:t35-b - q36 s41 s48
A:t05-a - q06 s11 t03
A:t05-b - q06 s11 s18 t03(weak)

Experiment Bruker_7, 2D 1H-1H via through-space (NOESY)
A:d09-H - d13-H q04-H? q07-H t03-a?   B:d39-H - d43-H? q37-H? t35-b?
A:d13-H - d09-H   B:q36-H - q37-H
A:d22-H - q04-H   B:q37-H - q36-H
A:q04-H - d22-H
A:q06-H - q07-H
A:q07-H - d09-H q06-H t05-a
A:q08-H - q04-H? q06-H? t03-a? t03-b? t05-a?
A:t05-a - q07-H

A:d13-H - B:d43-H?(exch)   B:d39-H - A:d09-H?(exch)
A:d14-H - B:d44-H?(exch)   B:d44-H - A:d14-H?(exch)
A:d15-H - B:d45-H?(exch)   B:d45-H - A:d15-H?(exch)
A:d19-H - B:d49-H?(exch)   B:d49-H - A:d19-H?(exch)
A:d20-H - B:d50-H?(exch)   B:d50-H - A:d20-H?(exch)
A:d21-H - B:d51-H?(exch)   B:d51-H - A:d21-H?(exch)
A:d22-H - B:d52-H?(exch)   B:d52-H - A:d22-H?(exch)
A:q02-H - B:q32-H?(exch)   B:q32-H - A:q02-H?(exch)
A:q04-H - B:q34-H?(exch)   B:q34-H - A:q04-H?(exch)
A:q06-H - B:q36-H?(exch)   B:q36-H - A:q06-H?(exch)
A:q08-H - B:q38-H?(exch)   B:q38-H - A:q08-H?(exch)
A:t01-a - B:t31-a?(exch)   B:t31-a - A:t01-a?(exch)
A:t01-b - B:t31-b?(exch)   B:t31-b - A:t01-b?(exch)
A:t03-a - B:t33-b?(exch)   B:t33-a - A:t03-b?(exch)
A:t03-b - B:t33-a?(exch)   B:t33-b - A:t03-a?(exch)
A:t05-a - B:t35-a?(exch)   B:t35-a - A:t05-a?(exch)
A:t05-b - B:t35-b?(exch)   B:t35-b - A:t05-b?(exch)

Influence of spatial structure on ^{1}H chemical shifts of ethyl groups

(on the example of **RS** stereoisomer)

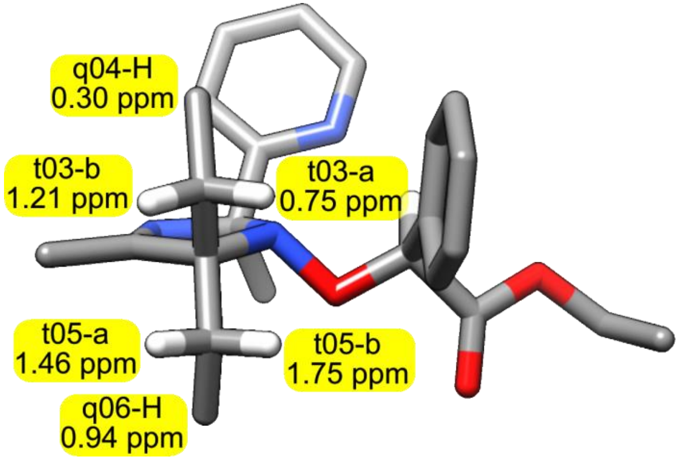

The most stable calculated **RS** conformer (RS.03, see http://limor1.nioch.nsc.ru/quant/NO-inversion/) and experimental chemical shifts for **A** (see above) are shown. Only relevant hydrogen atoms are depicted.

t03-a proton and q04-H methyl protons fall into shielding cones of aromatic rings while t05-b proton undergoes deshielding effect of lone pairs of oxygen atom of NO fragment

^{1}H NMR spectrum (600 MHz)

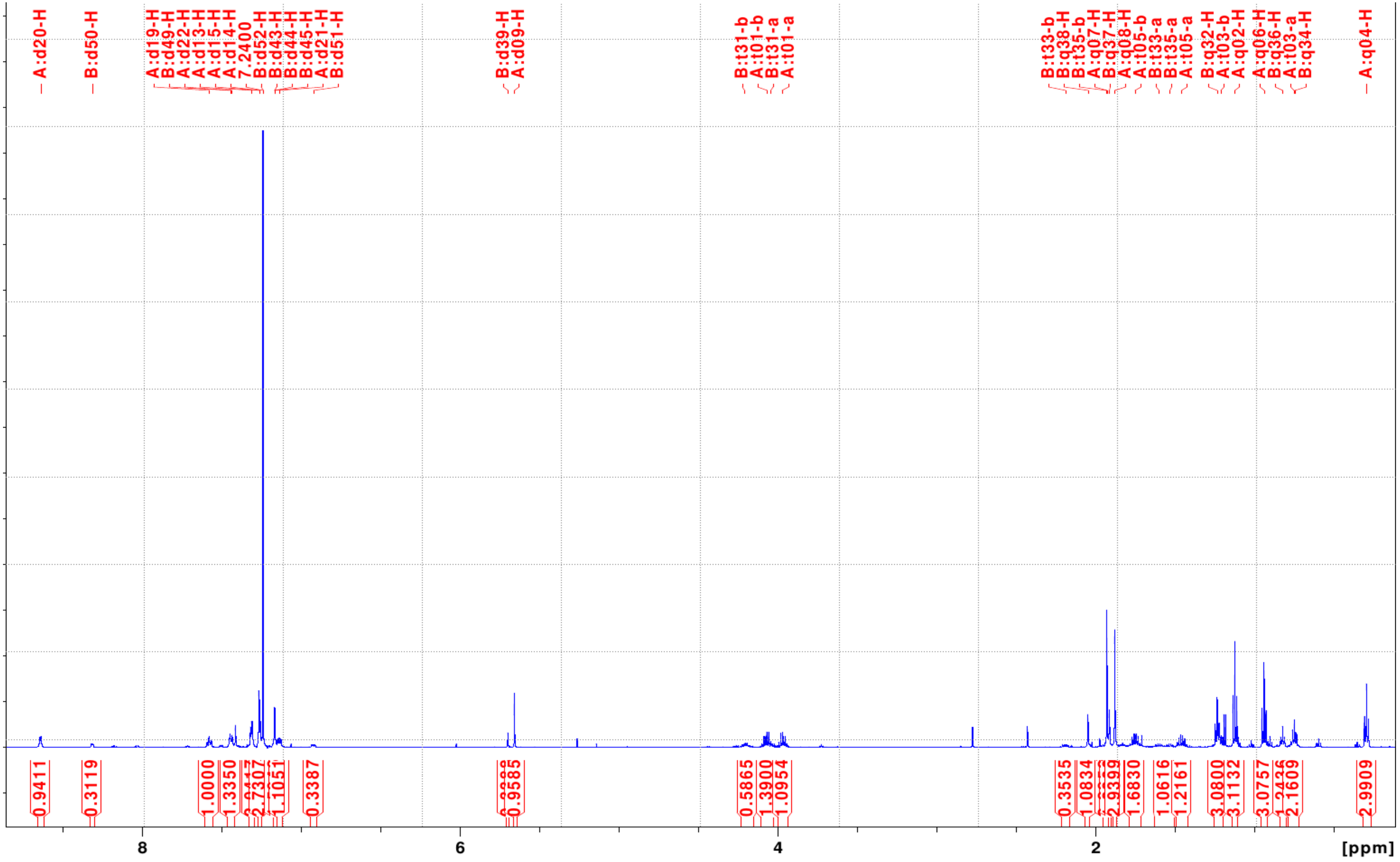

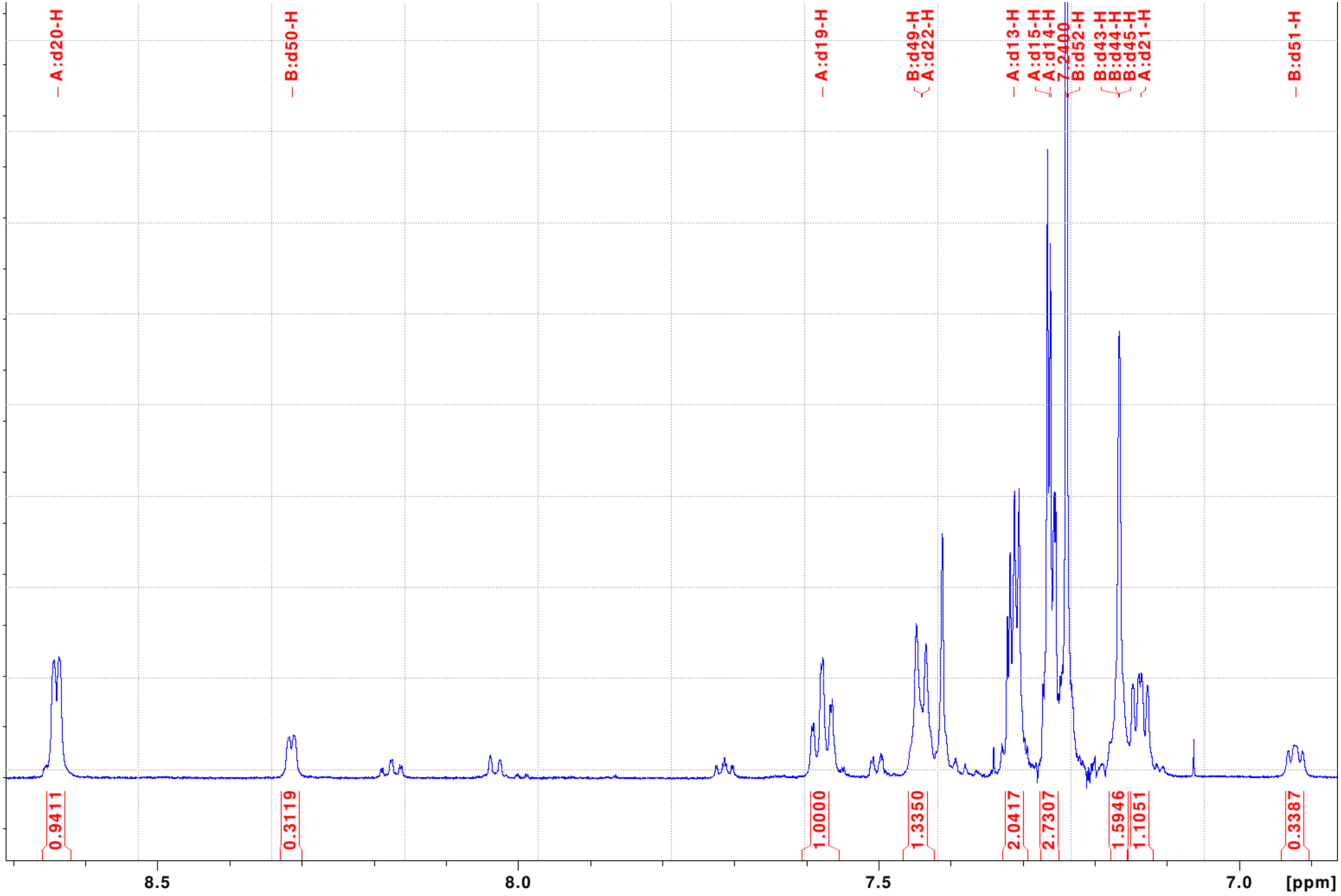
A:d20-H
B:d50-H
A:d19-H
B:d49-H
A:d22-H
A:d13-H
A:d15-H
A:d14-H
7.2400
B:d52-H
B:d43-H
B:d44-H
B:d45-H
A:d21-H
B:d51-H
0.9411
0.3119
1.0000
1.3350
2.0417
2.7307
1.5946
1.1051
0.3387
8.5
8.0
7.5
7.0
[ppm]

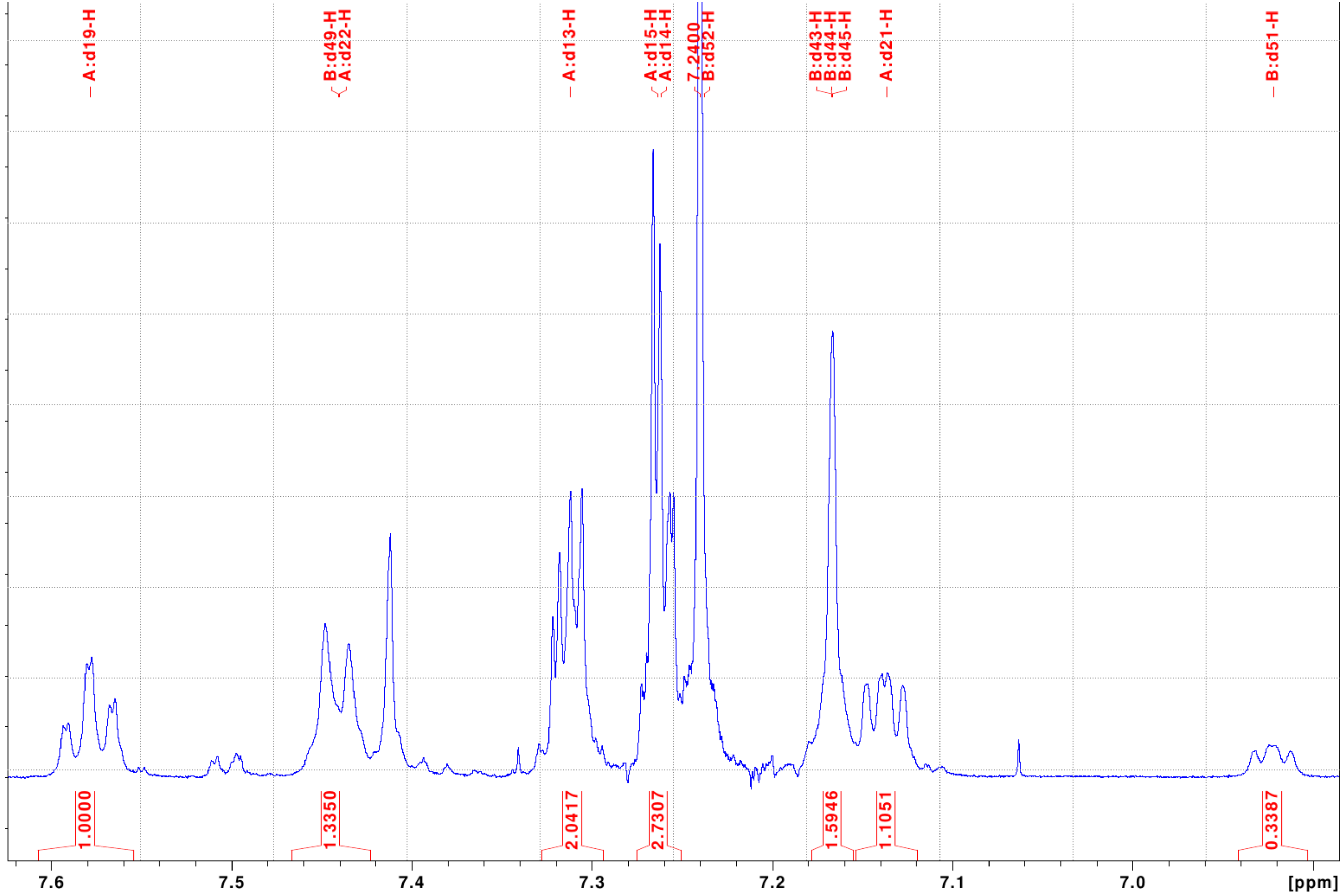
A:d19-H
B:d49-H
A:d22-H
A:d13-H
A:d15-H
A:d14-H
7.2400
B:d52-H
B:d43-H
B:d44-H
B:d45-H
A:d21-H
B:d51-H
1.0000
1.3350
2.0417
2.7307
1.5946
1.1051
0.3387
7.6
7.5
7.4
7.3
7.2
7.1
7.0
[ppm]

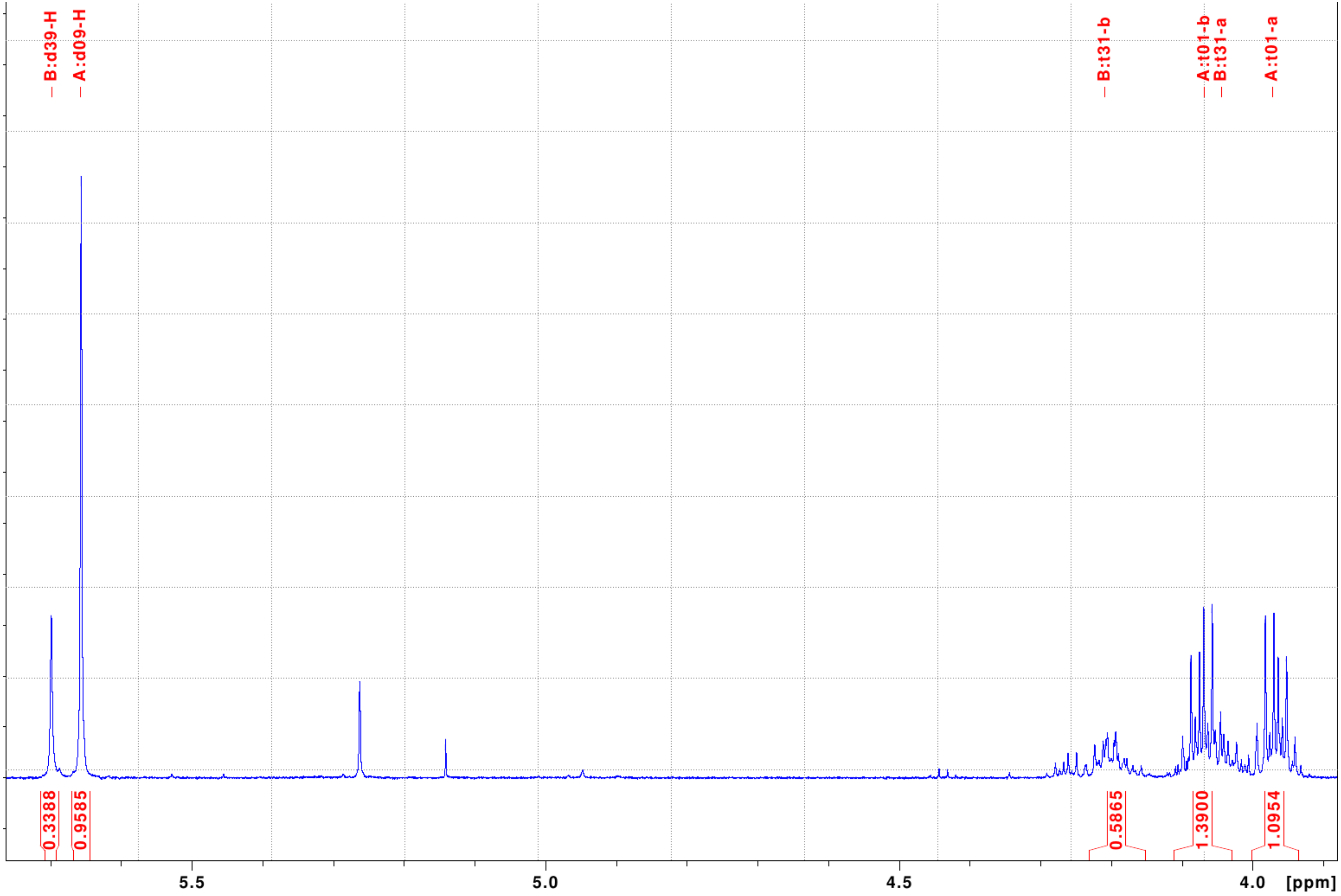

B:d39-H
A:d09-H
B:t31-b
A:t01-b
B:t31-a
A:t01-a
0.3388
0.9585
0.5865
1.3900
1.0954
5.5
5.0
4.5
4.0
[ppm]

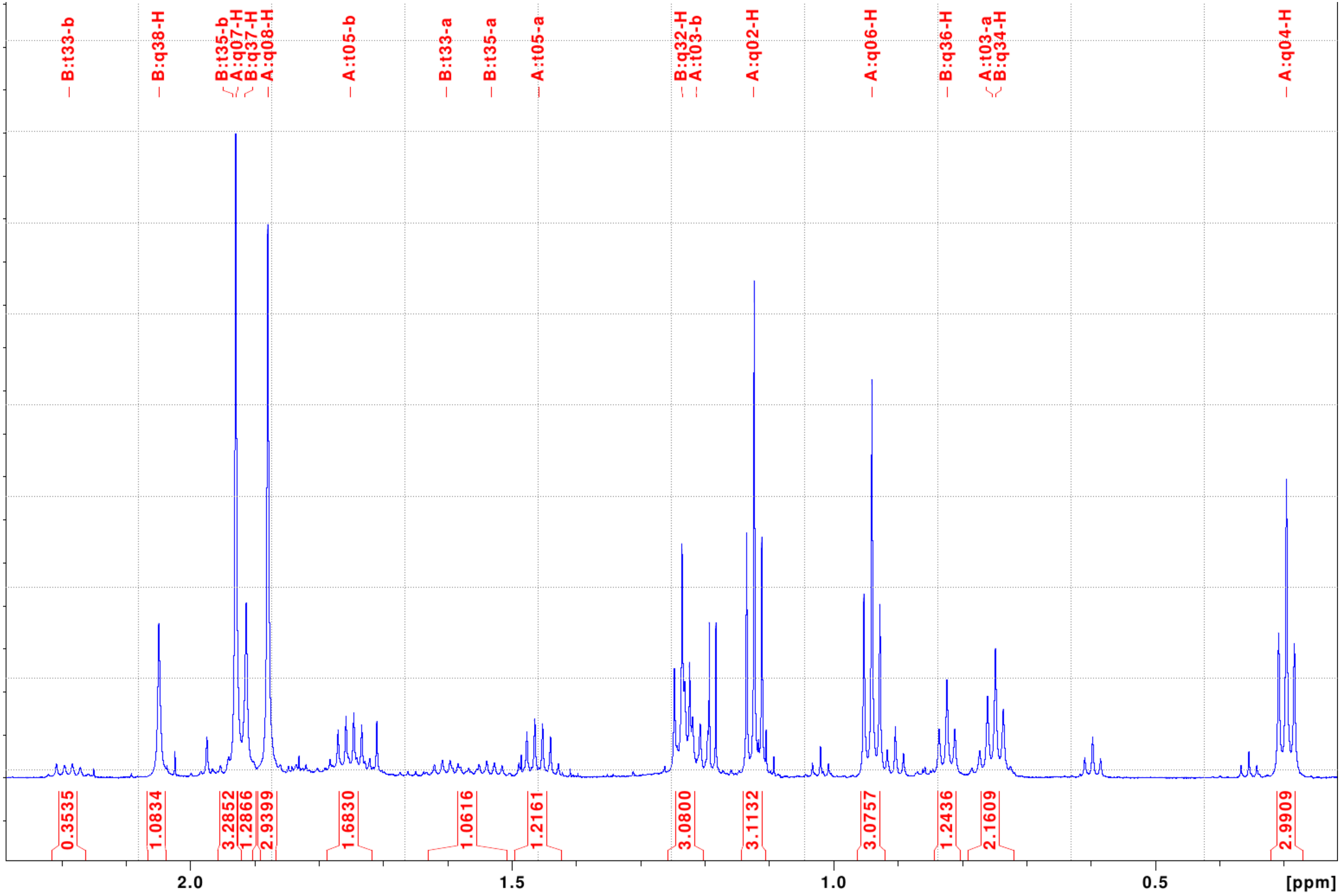
B:t33-b
B:q38-H
B:t35-b
A:q07-H
B:q37-H
A:q08-H
A:t05-b
B:t33-a
B:t35-a
A:t05-a
B:q32-H
A:t03-b
A:q02-H
A:q06-H
B:q36-H
A:t03-a
B:q34-H
A:q04-H
0.3535
1.0834
3.2852
1.2866
2.9399
1.6830
1.0616
1.2161
3.0800
3.1132
3.0757
1.2436
2.1609
2.9909
2.0
1.5
1.0
0.5
[ppm]

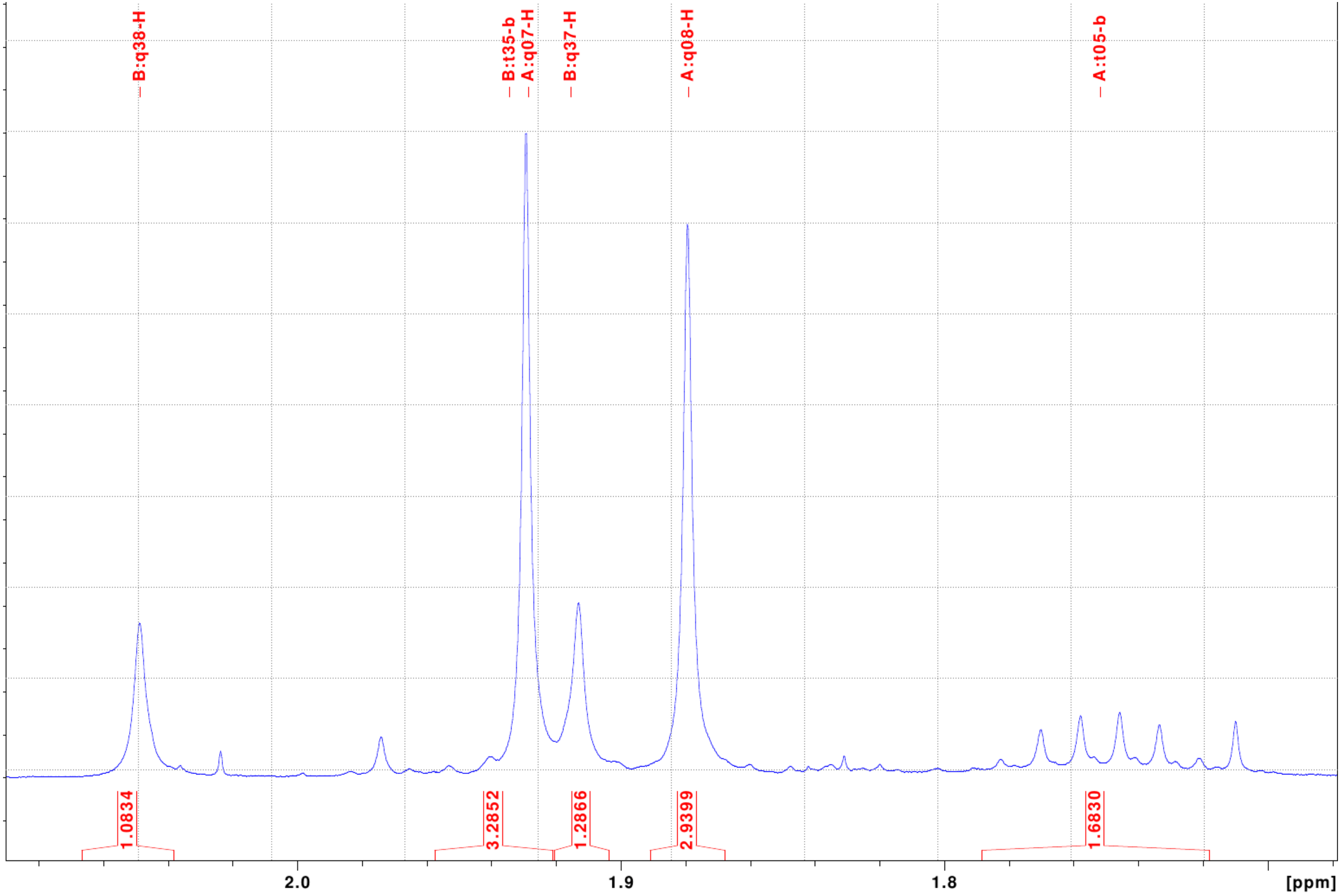
B:q38-H
B:t35-b
A:q07-H
B:q37-H
A:q08-H
A:t05-b
1.0834
3.2852
1.2866
2.9399
1.6830
2.0
1.9
1.8
[ppm]

$^{13}C\{^{1}H\}$ NMR spectrum (150 MHz)

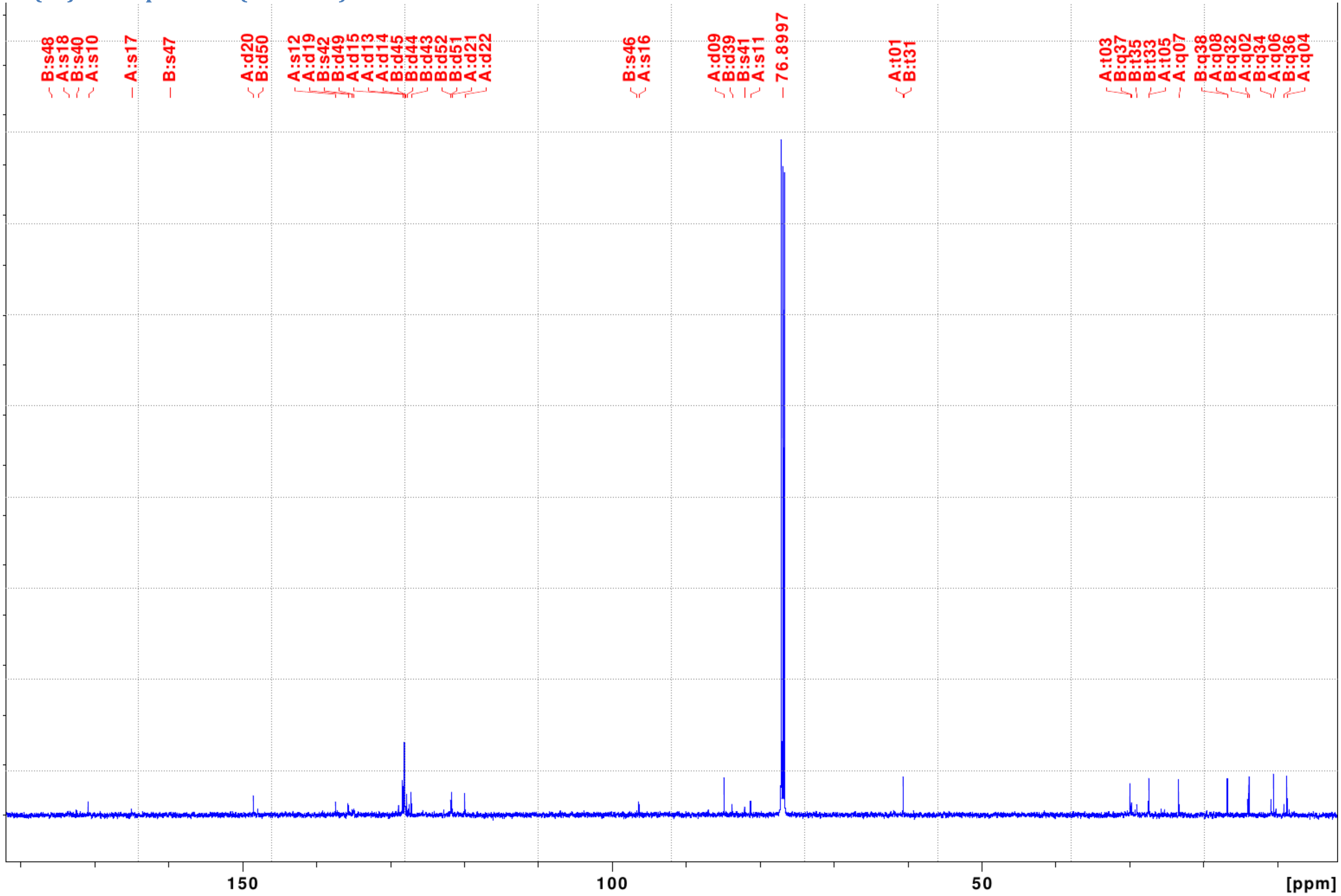

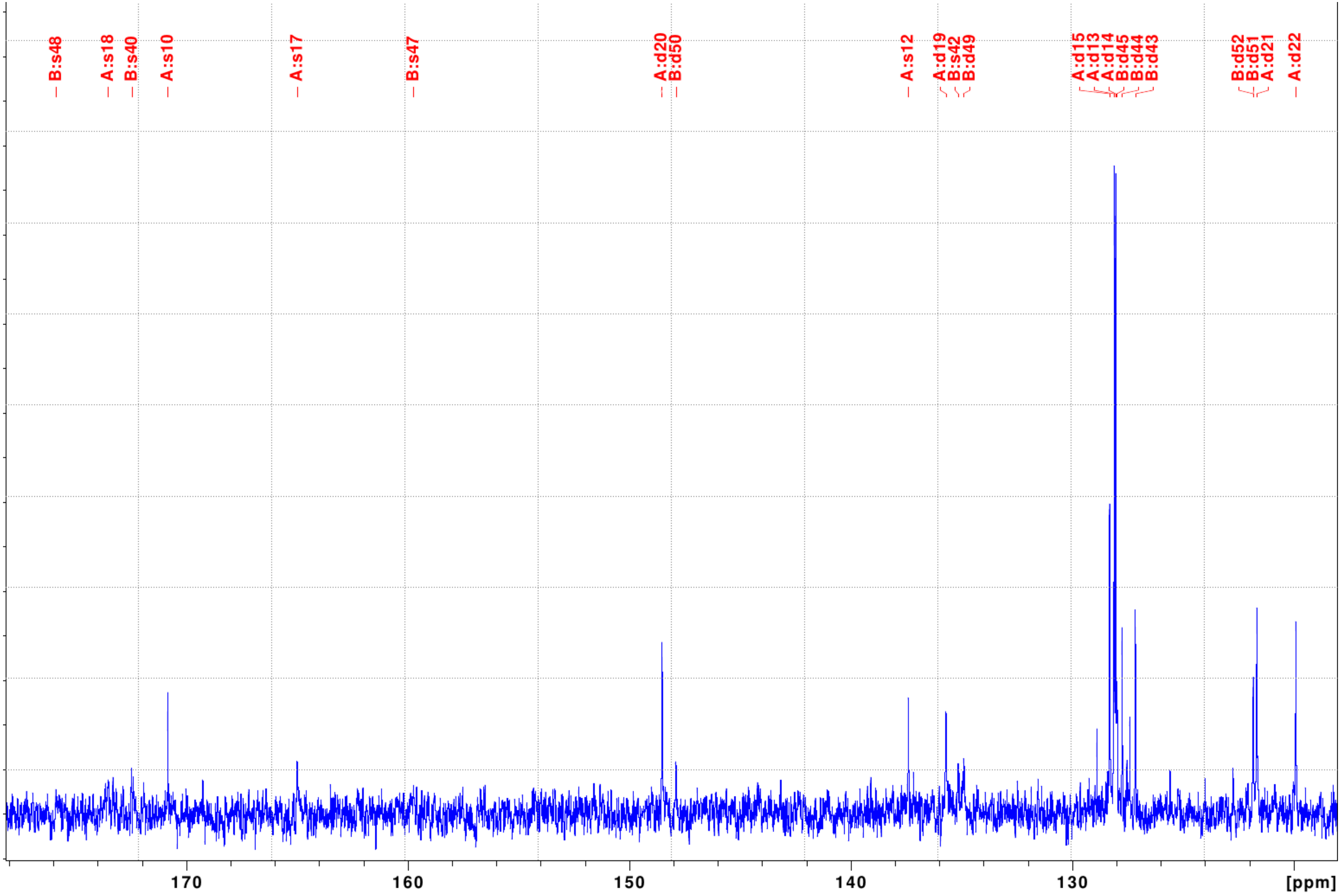
B:s48
A:s18
B:s40
A:s10
A:s17
B:s47
A:d20
B:d50
A:s12
A:d19
B:s42
B:d49
A:d15
A:d13
A:d14
B:d45
B:d44
B:d43
B:d52
B:d51
A:d21
A:d22
170
160
150
140
130
[ppm]

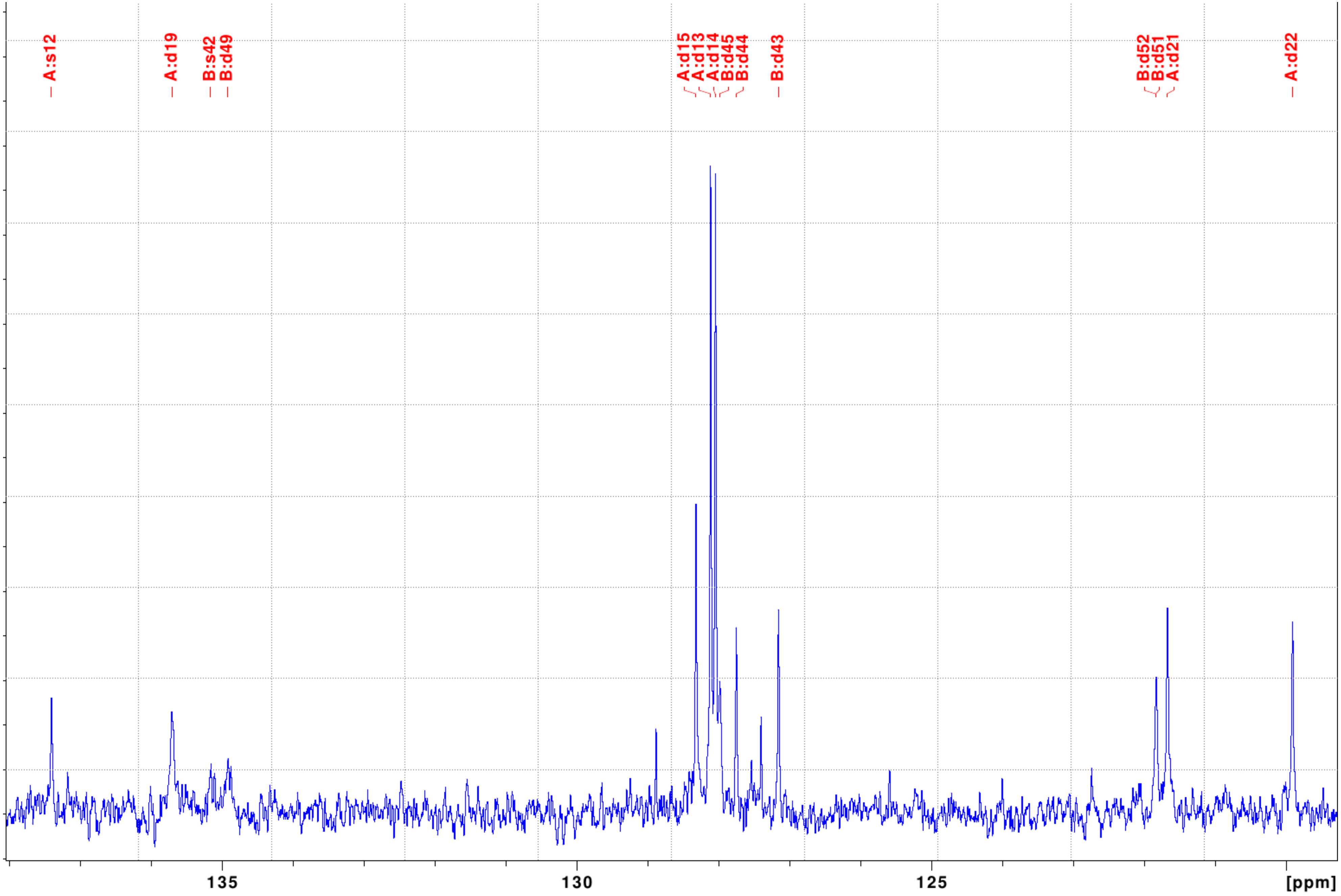
A:s12
A:d19
B:s42
B:d49
A:d15
A:d13
A:d14
B:d45
B:d44
B:d43
B:d52
B:d51
A:d21
A:d22
135
130
125
[ppm]

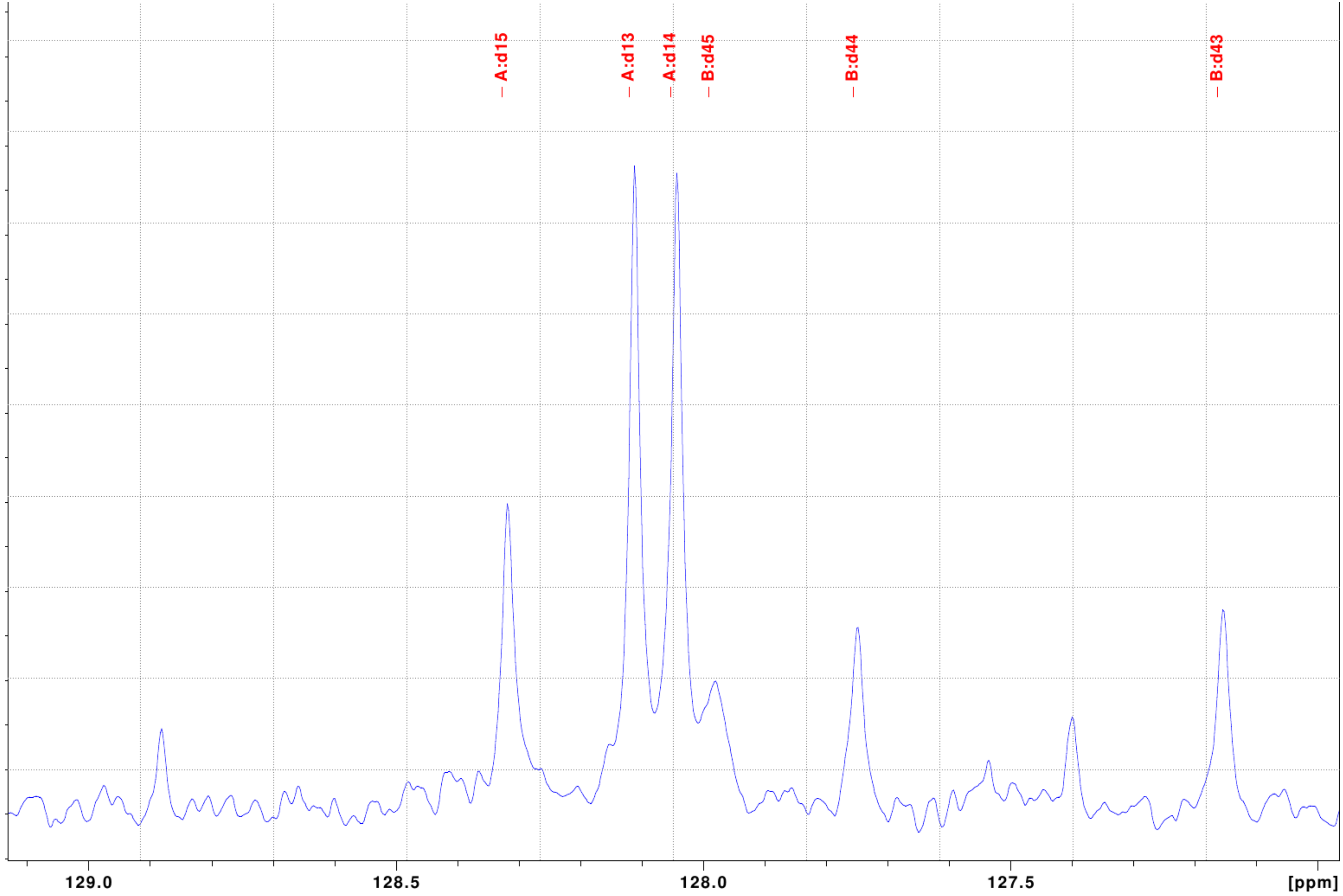
A:d15
A:d13
A:d14
B:d45
B:d44
B:d43
129.0
128.5
128.0
127.5
[ppm]

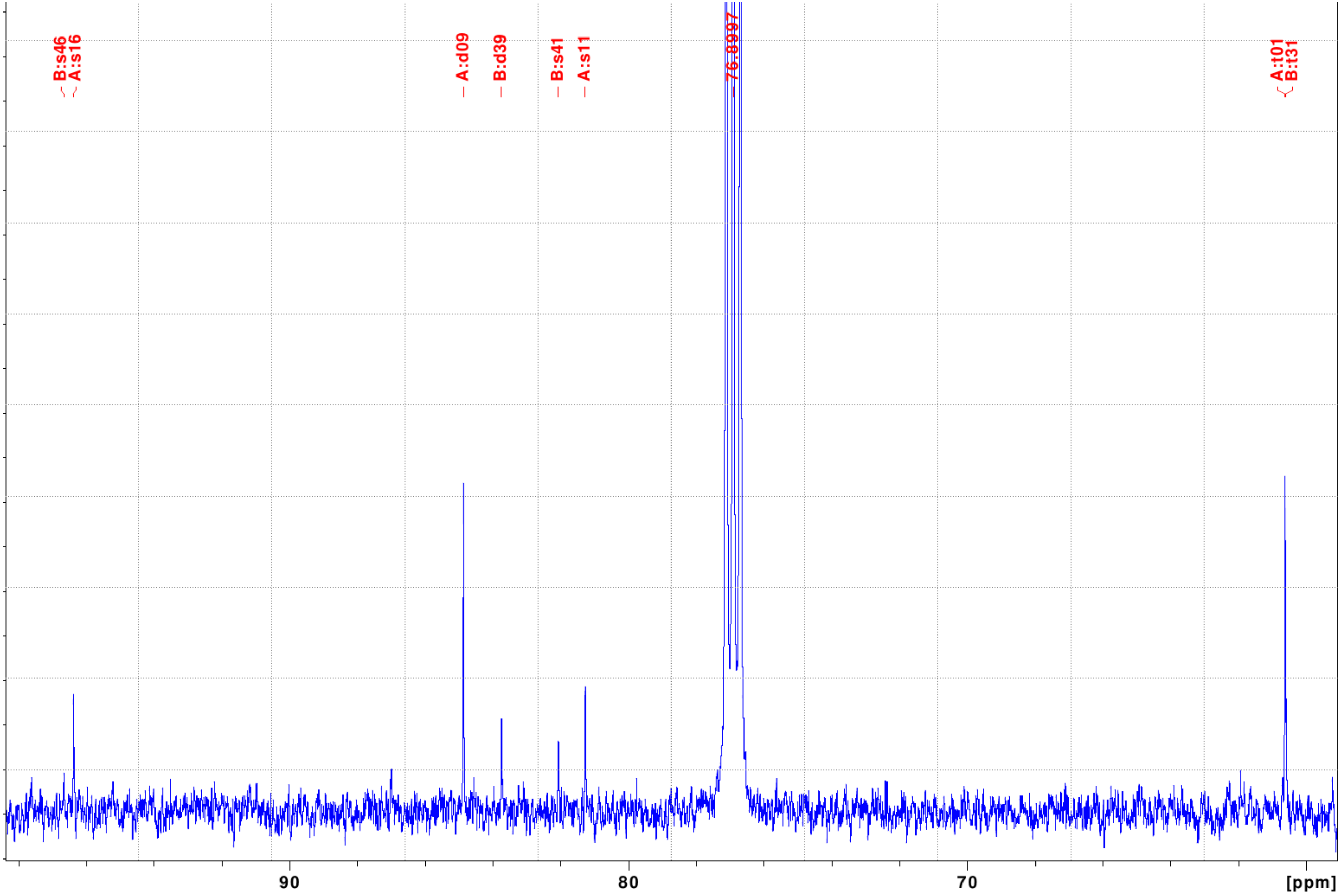
B:s46
A:s16
A:d09
B:d39
B:s41
A:s11
76.8997
A:t01
B:t31
90
80
70
[ppm]

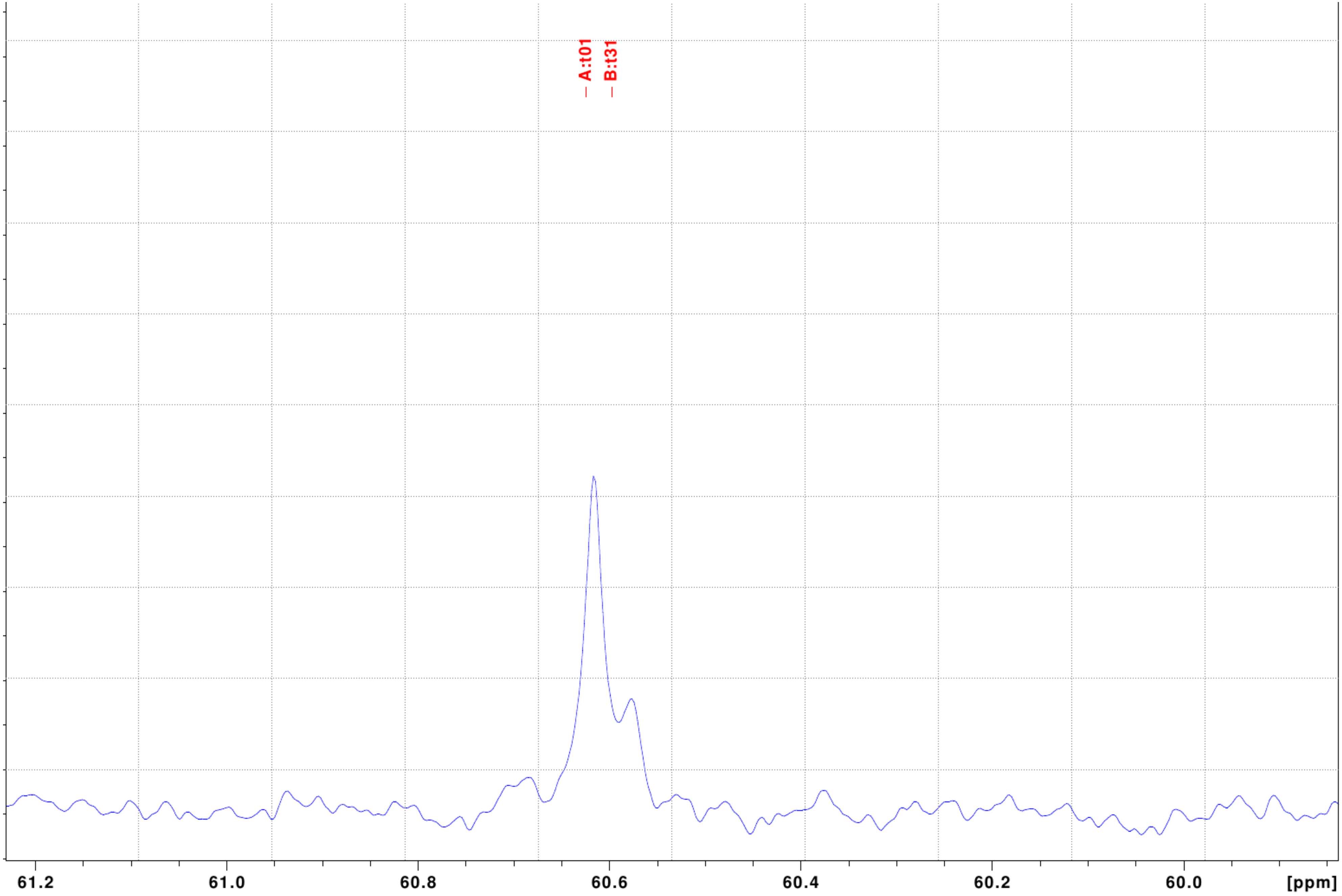
A:t01
B:t31
61.2
61.0
60.8
60.6
60.4
60.2
60.0
[ppm]

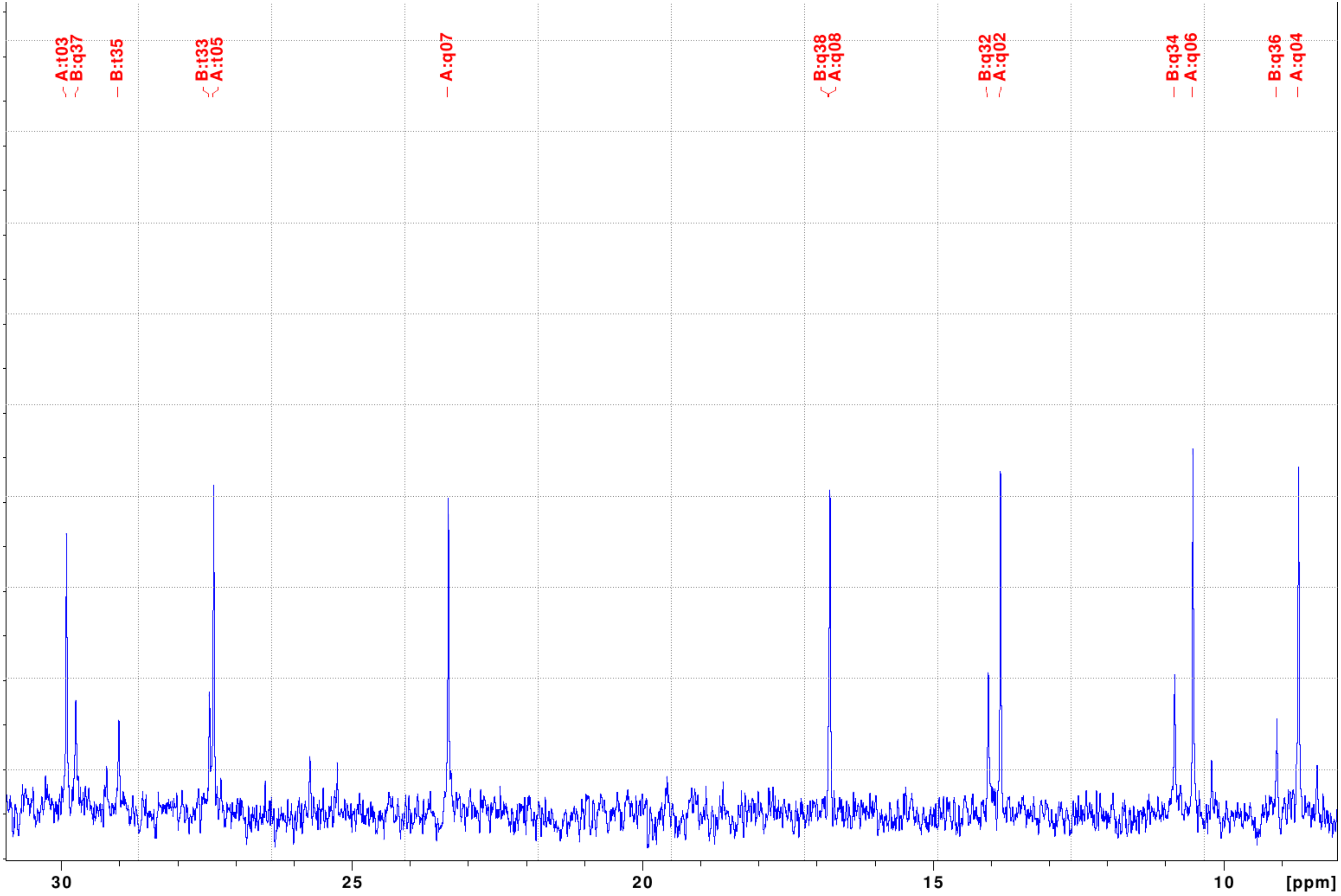

A:t03
B:q37
B:t35
B:t33
A:t05
A:q07
B:q38
A:q08
B:q32
A:q02
B:q34
A:q06
B:q36
A:q04
30
25
20
15
10
[ppm]

^{1}H NMR spectrum of radical 1˙ in toluene-d_8 at 108 °C

3.4 mg 1˙ in 0.5 mL toluene-d_8

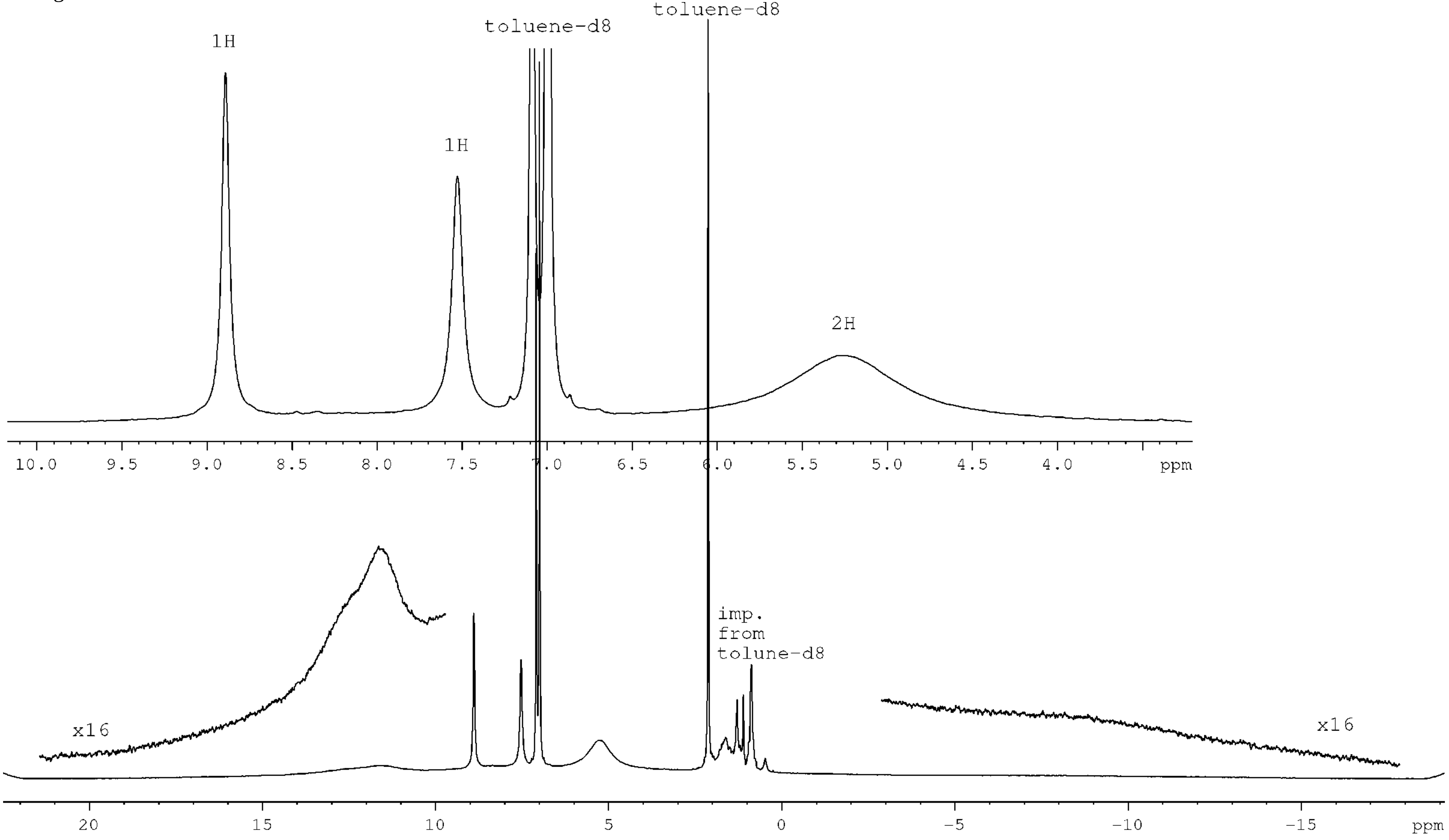

NMR spectra of alkoxyamine 3

5.8 mg **3** in toluene-d_8 at 25 °C

Signal assignments

Alcox D-604 Tol orig, alk1_2, major product A

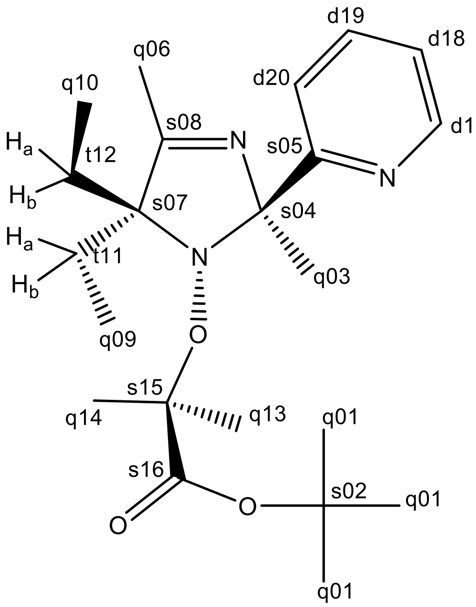

A

Experiment Bruker_1, 1D 13C: 20 peaks
q01 27.6
s02 79.5
q03 22.6
s04 96.1
s05 164.9
q06 16.6
s07 81.1
s08 172.4
q09 11.0
q10 9.3
t11 27.4
t12 29.4
q13 24.8
q14 24.3
s15 82.4
s16 173.1
d17 147.6
d18 121.4
d19 135.0
d20 121.0

Experiment Bruker_10, 1D 1H: 15 peaks
q01-H 1.39
q03-H 2.18
q06-H 1.79
q09-H 1.06
q10-H 0.70
t11-a 1.62
t11-b 2.15
t12-a 1.42
t12-b 1.73
q13-H 1.53
q14-H 1.26
d17-H 8.50
d18-H 6.68
d19-H 7.18
d20-H 7.49

Experiment Bruker_6, 2D 13C-1H via onebond (HSQC): 15 peaks
d17-H - d17(177 Hz)
d18-H - d18(163 Hz)
d19-H - d19
d20-H - d20(163 Hz)
q01-H - q01(127 Hz)
q03-H - q03(129 Hz)
q06-H - q06(127 Hz)
q09-H - q09(125 Hz)
q10-H - q10(125 Hz)
q13-H - q13(128 Hz)
q14-H - q14(128 Hz)
t11-a - t11
t11-b - t11
t12-a - t12
t12-b - t12

Experiment Bruker_5, 2D 1H-1H via Jcoupling (COSY): 19 peaks
d17-H - d18-H
d18-H - d17-H d19-H
d19-H - d17-H?(weak) d18-H d20-H
d20-H - d19-H
q09-H - t11-a t11-b
q10-H - t12-a t12-b
t11-a - q09-H t11-b
t11-b - q09-H t11-a
t12-a - q10-H t12-b
t12-b - q10-H t12-a

Experiment Bruker_7, 2D 13C-1H via Jcoupling (HMBC): 36 peaks
d17-H - d18(weak) d19
d18-H - d17 d20
d19-H - s05
d20-H - d18
q01-H - q01 s02
q03-H - s04 s05
q06-H - s07 s08
q09-H - s07 t11
q10-H - s07 t12
q13-H - q14 s15 s16
q14-H - q13 s15 s16
t11-a - q09 s07 s08 t12
t11-b - q09 s07 s08 t12
t12-a - q10 s07
t12-b - q10 s07 s08 t11

Experiment Bruker_9, 2D 1H-13C via onebond (H-C correlation): 15 peaks
d17 - d17-H
d18 - d18-H
d19 - d19-H
d20 - d20-H
q01 - q01-H
q03 - q03-H
q06 - q06-H
q09 - q09-H
q10 - q10-H
q13 - q13-H
q14 - q14-H
t11 - t11-a t11-b
t12 - t12-a t12-b

Experiment Bruker_8, 2D 1H-1H via through-space (NOESY): 15 peaks
d20-H - q10-H
q03-H - q09-H q13-H
q06-H - q09-H q10-H t12-a?
q09-H - q03-H q06-H
q10-H - d20-H q06-H
q13-H - q03-H q14-H
q14-H - q10-H? q13-H t11-b?

Alcox D-604 Tol orig, alk1_2, minor product B

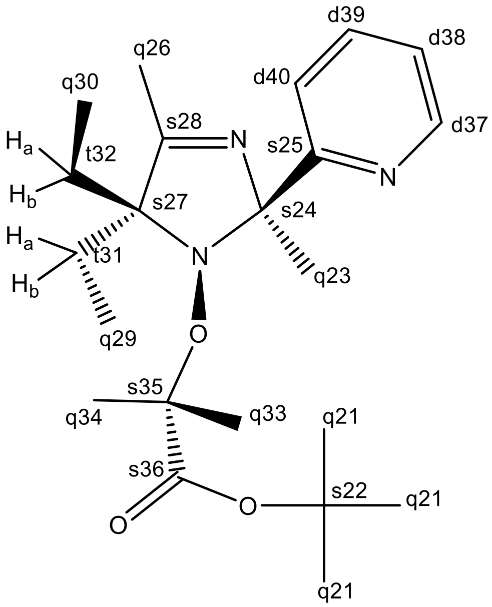

B

Experiment Bruker_1, 1D 13C: 20 peaks
q21   27.5
s22   79.4
q23   28.0
s24   96.9
s25 160.7
q26   16.5
s27   82.2
s28 174.1
q29   9.5
q30   11.4
t31   30.1
t32   27.5
q33   23.4
q34   25.0
s35   82.0
s36 173.4
d37 147.5
d38 121.4
d39 134.1
d40 122.8

Experiment Bruker_10, 1D 1H: 15 peaks
q21-H   1.33
q23-H   2.14
q26-H   1.86
q29-H   0.95
q30-H   0.80
t31-a   1.44
t31-b   1.80
t32-a   1.44
t32-b   2.50
q33-H   1.88
q34-H   1.29
d37-H   8.54
d38-H   6.69
d39-H   7.15
d40-H   7.44

Experiment Bruker_6, 2D 13C-1H via onebond (HSQC): 15 peaks
d37-H - d37
d38-H - d38
d39-H - d39
d40-H - d40
q21-H - q21
q23-H - q23
q26-H - q26
q29-H - q29
q30-H - q30
q33-H - q33
q34-H - q34
t31-a - t31
t31-b - t31
t32-a - t32
t32-b - t32

Experiment Bruker_5, 2D 1H-1H via Jcoupling (COSY): 19 peaks
d37-H - d38-H d39-H(weak)
d38-H - d37-H d39-H
d39-H - d37-H(weak) d38-H d40-H
d40-H - d39-H
q29-H - t31-a t31-b
q30-H - t32-a t32-b
t31-a - q29-H
t31-b - q29-H t31-a?
t32-a - q30-H t32-b
t32-b - q30-H t32-a

Experiment Bruker_7, 2D 13C-1H via Jcoupling (HMBC): 22 peaks
d38-H - d37
d40-H - d38
q21-H - q21 s22
q23-H - s24 s25
q26-H - s27 s28
q29-H - s27 t31
q30-H - s27 t32
q33-H - q34 s35 s36
q34-H - q33 s35 s36
t31-a - t32
t31-b - s27 s28
t32-b - s27

Experiment Bruker_9, 2D 1H-13C via onebond (H-C correlation): 6 peaks
d39 - d39-H
d40 - d40-H
q21 - q21-H
q23 - q23-H
q29 - q29-H
q34 - q34-H

Experiment Bruker_8, 2D 1H-1H via through-space (NOESY): 15 peaks
d40-H - q30-H
q23-H - q29-H q33-H
q26-H - q29-H q30-H t31-a? t32-a?
q29-H - q23-H q26-H
q30-H - d40-H q26-H
q33-H - d40-H? q23-H q34-H
q34-H - q33-H

¹H NMR spectrum (600 MHz)

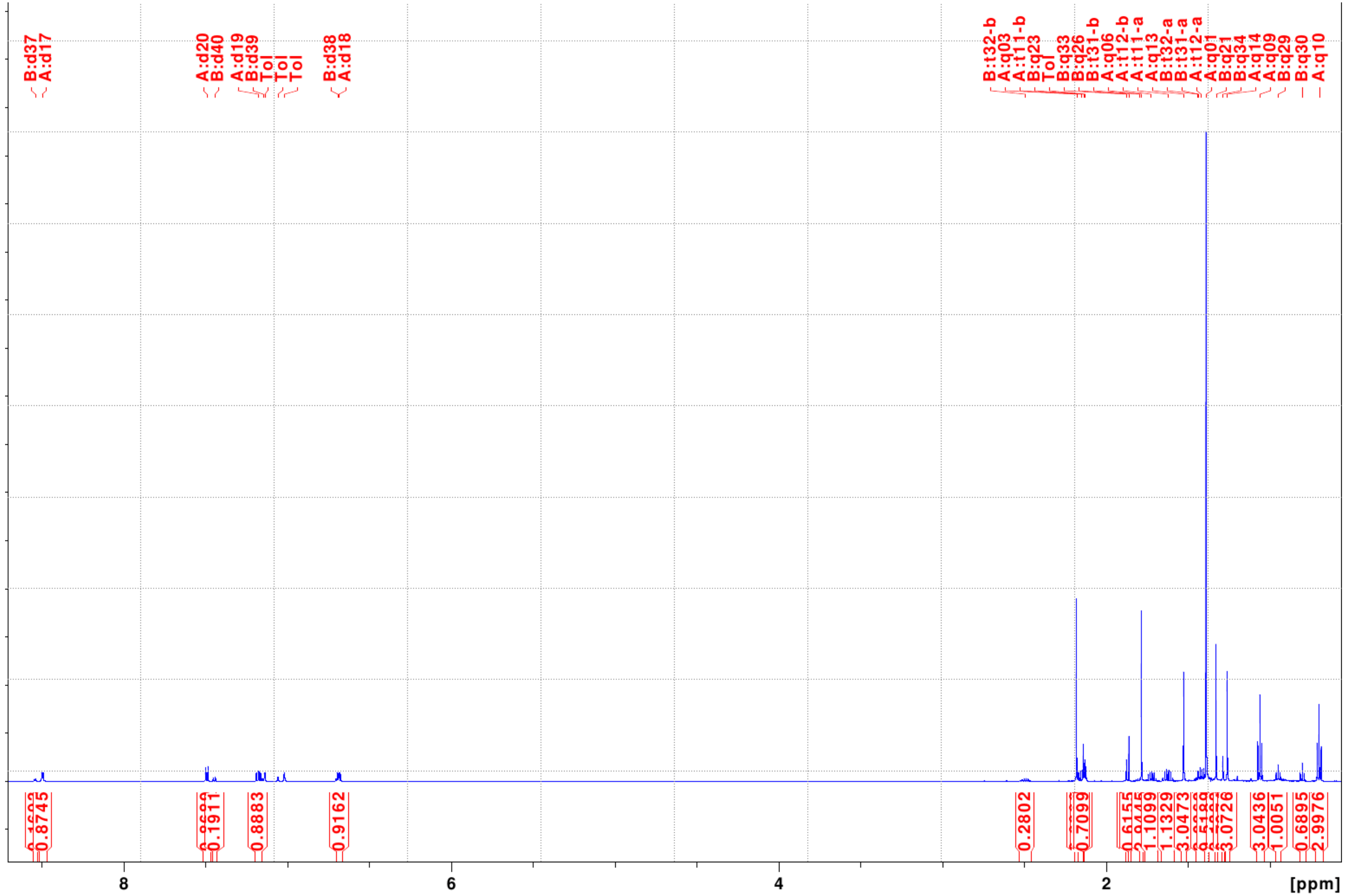

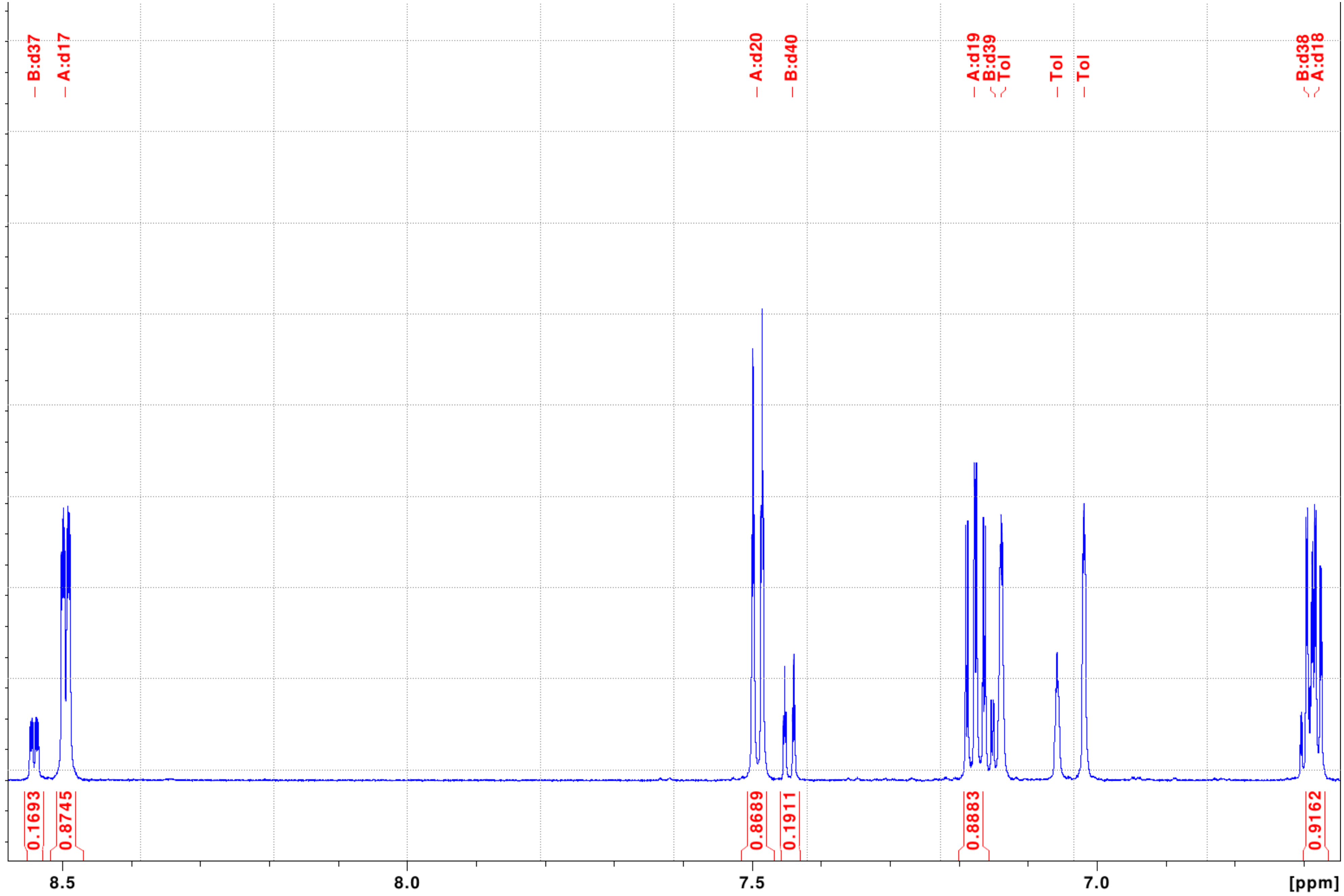
B:d37
A:d17
A:d20
B:d40
A:d19
B:d39
Tol
Tol
Tol
B:d38
A:d18
0.1693
0.8745
0.8689
0.1911
0.8883
0.9162
8.5
8.0
7.5
7.0
[ppm]

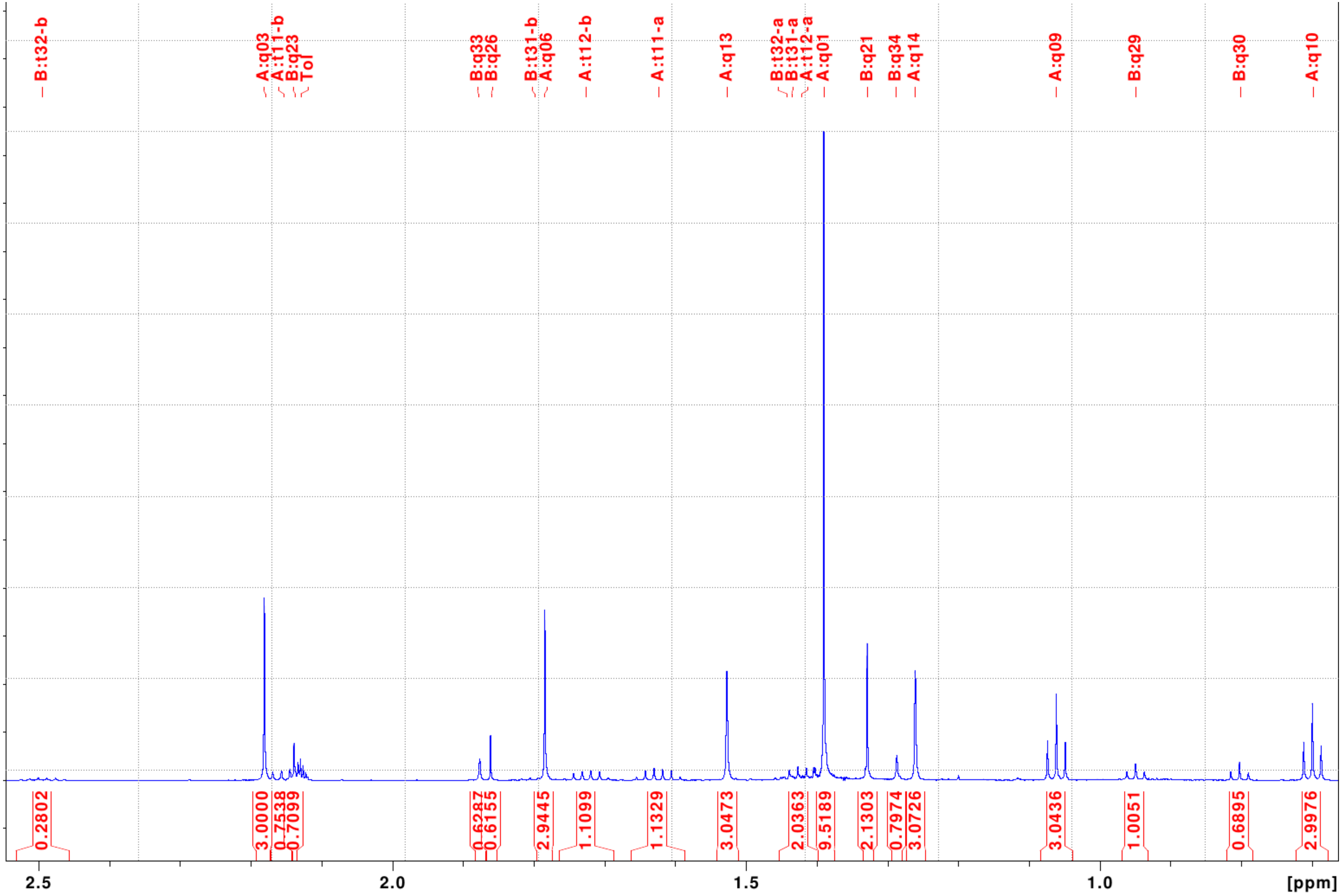
B:t32-b
A:q03
A:t11-b
B:q23
Tol
B:q33
B:q26
B:t31-b
A:q06
A:t12-b
A:t11-a
A:q13
B:t32-a
B:t31-a
A:t12-a
A:q01
B:q21
B:q34
A:q14
A:q09
B:q29
B:q30
A:q10
0.2802
3.0000
0.7538
0.7099
0.6287
0.6155
2.9445
1.1099
1.1329
3.0473
2.0363
9.5189
2.1303
0.7974
3.0726
3.0436
1.0051
0.6895
2.9976
2.5
2.0
1.5
1.0
[ppm]

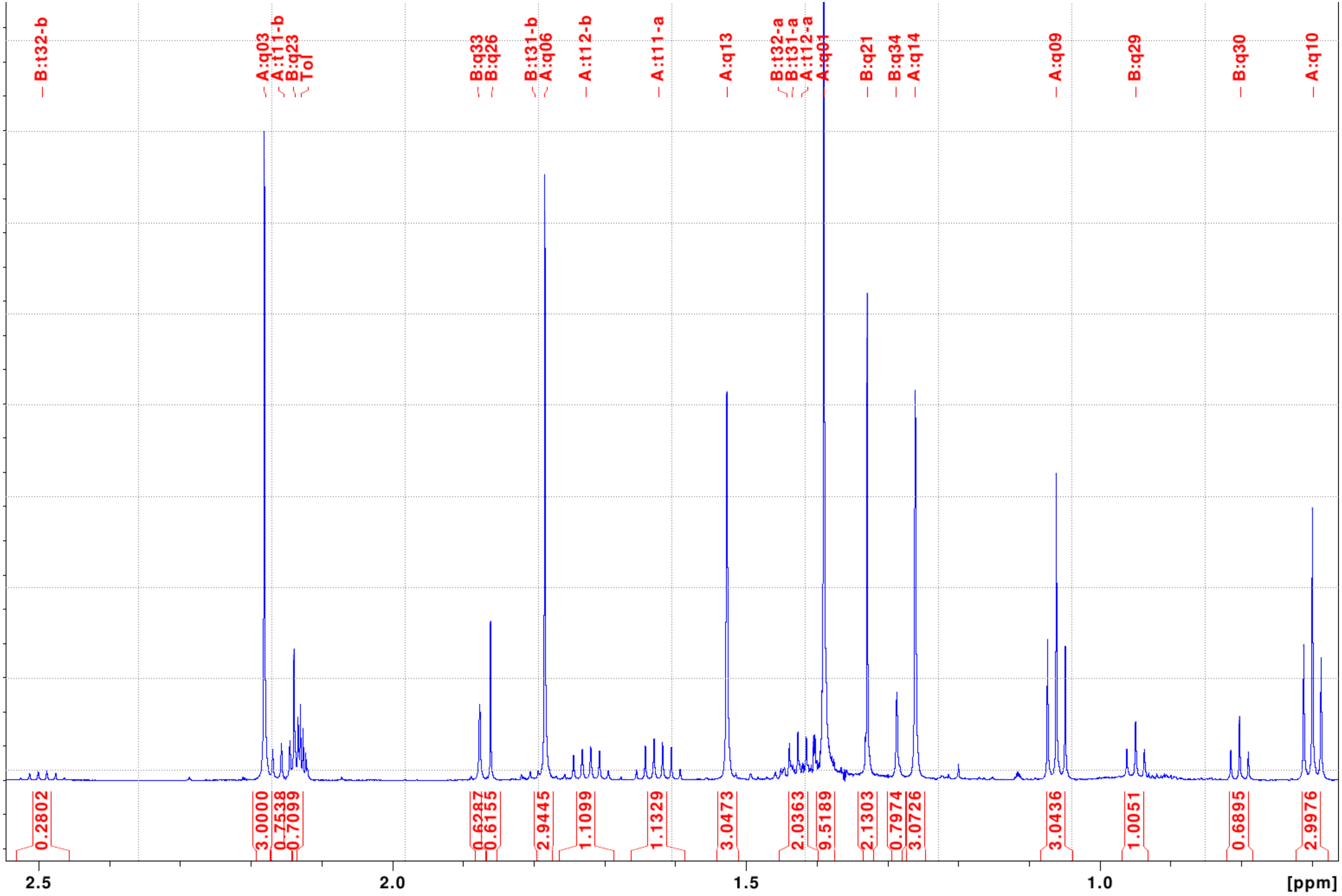
B:t32-b
A:q03
A:t11-b
B:q23
Tol
B:q33
B:q26
B:t31-b
A:q06
A:t12-b
A:t11-a
A:q13
B:t32-a
B:t31-a
A:t12-a
A:q01
B:q21
B:q34
A:q14
A:q09
B:q29
B:q30
A:q10
0.2802
3.0000
0.7538
0.7099
0.6287
0.6155
2.9445
1.1099
1.1329
3.0473
2.0363
9.5189
2.1303
0.7974
3.0726
3.0436
1.0051
0.6895
2.9976
2.5
2.0
1.5
1.0
[ppm]

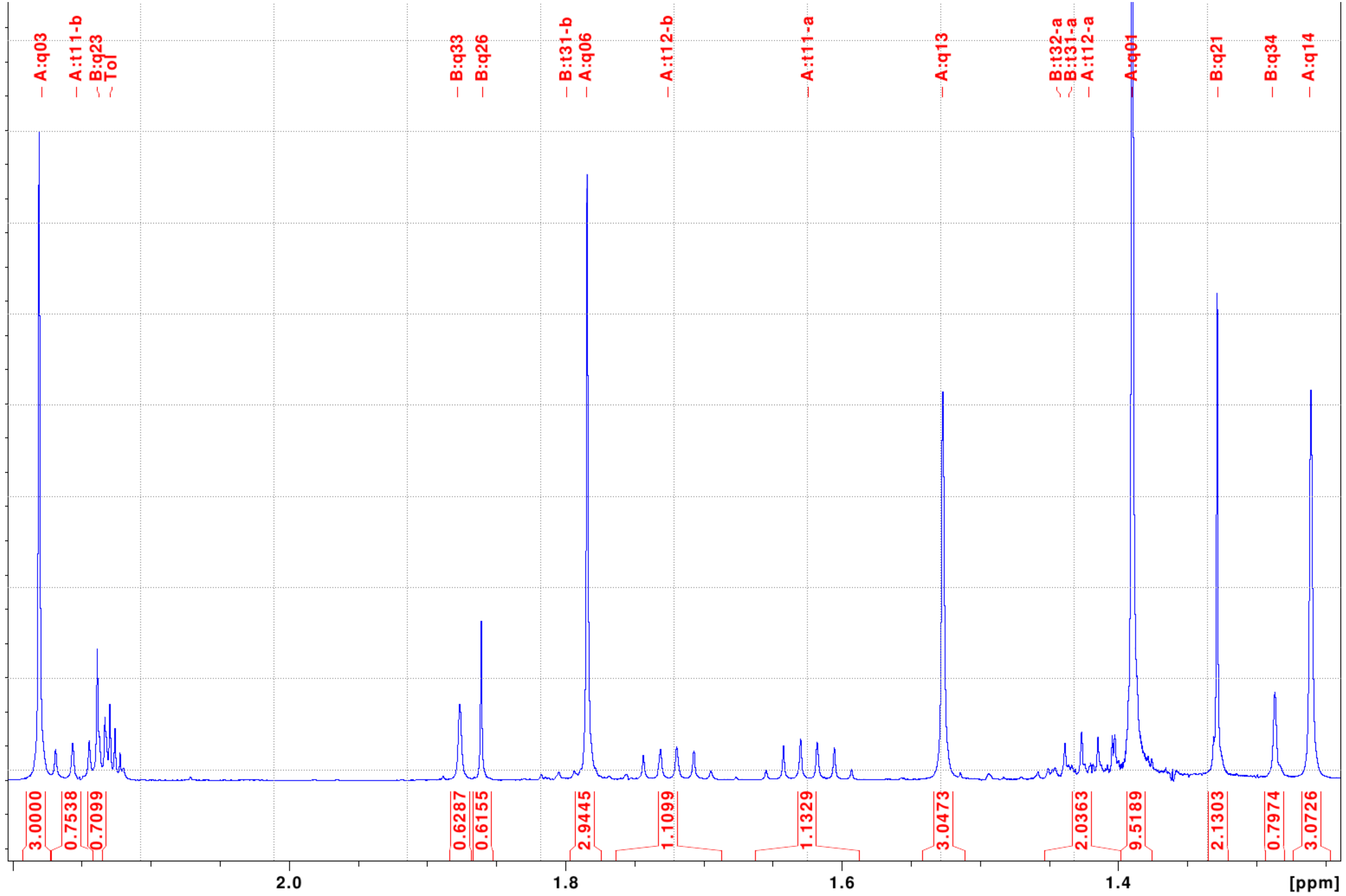
A:q03
A:t11-b
B:q23
Tol
B:q33
B:q26
B:t31-b
A:q06
A:t12-b
A:t11-a
A:q13
B:t32-a
B:t31-a
A:t12-a
A:q01
B:q21
B:q34
A:q14
3.0000
0.7538
0.7099
0.6287
0.6155
2.9445
1.1099
1.1329
3.0473
2.0363
9.5189
2.1303
0.7974
3.0726
2.0
1.8
1.6
1.4
[ppm]

$^{13}C\{^{1}H\}$ NMR spectrum (150 MHz)

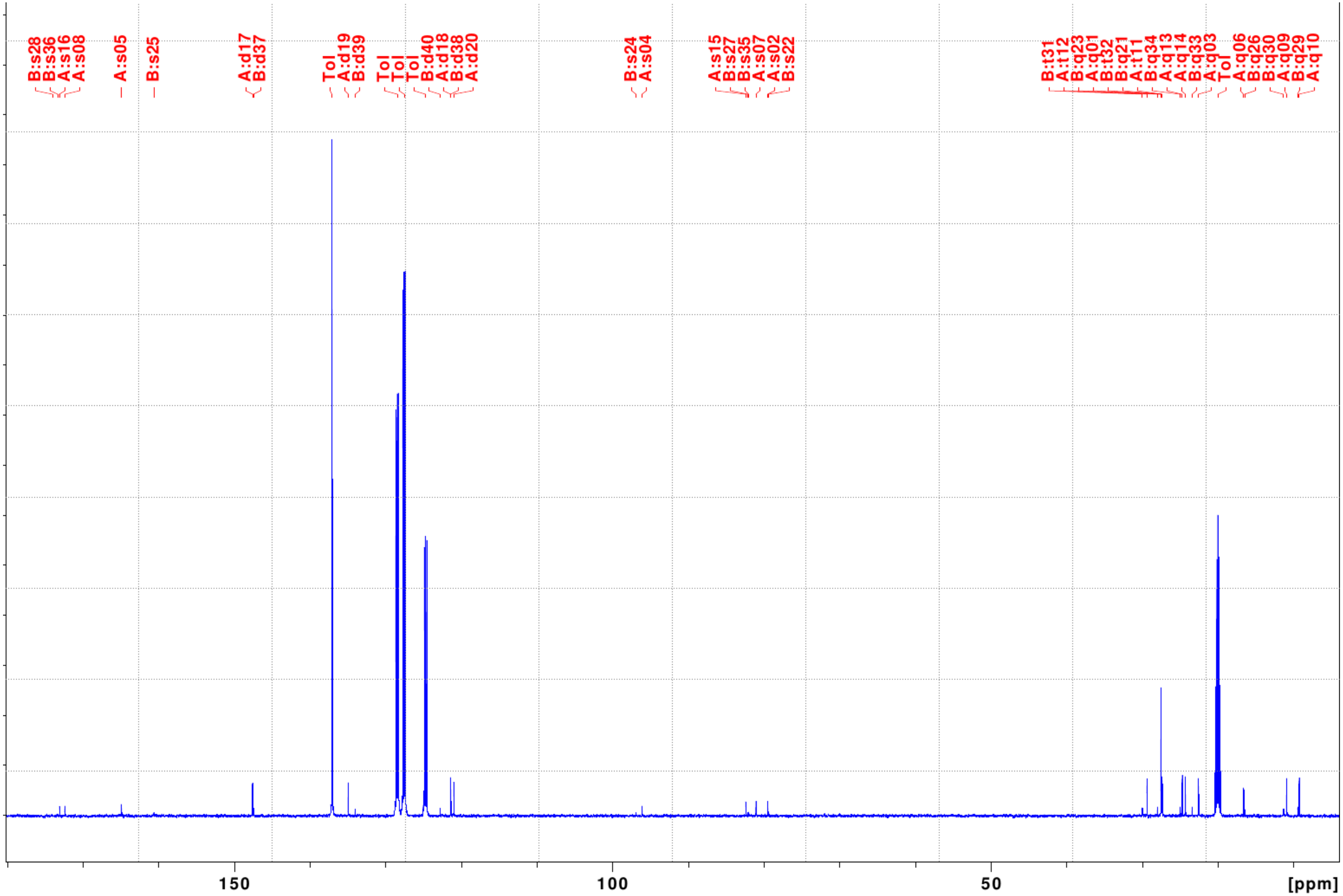

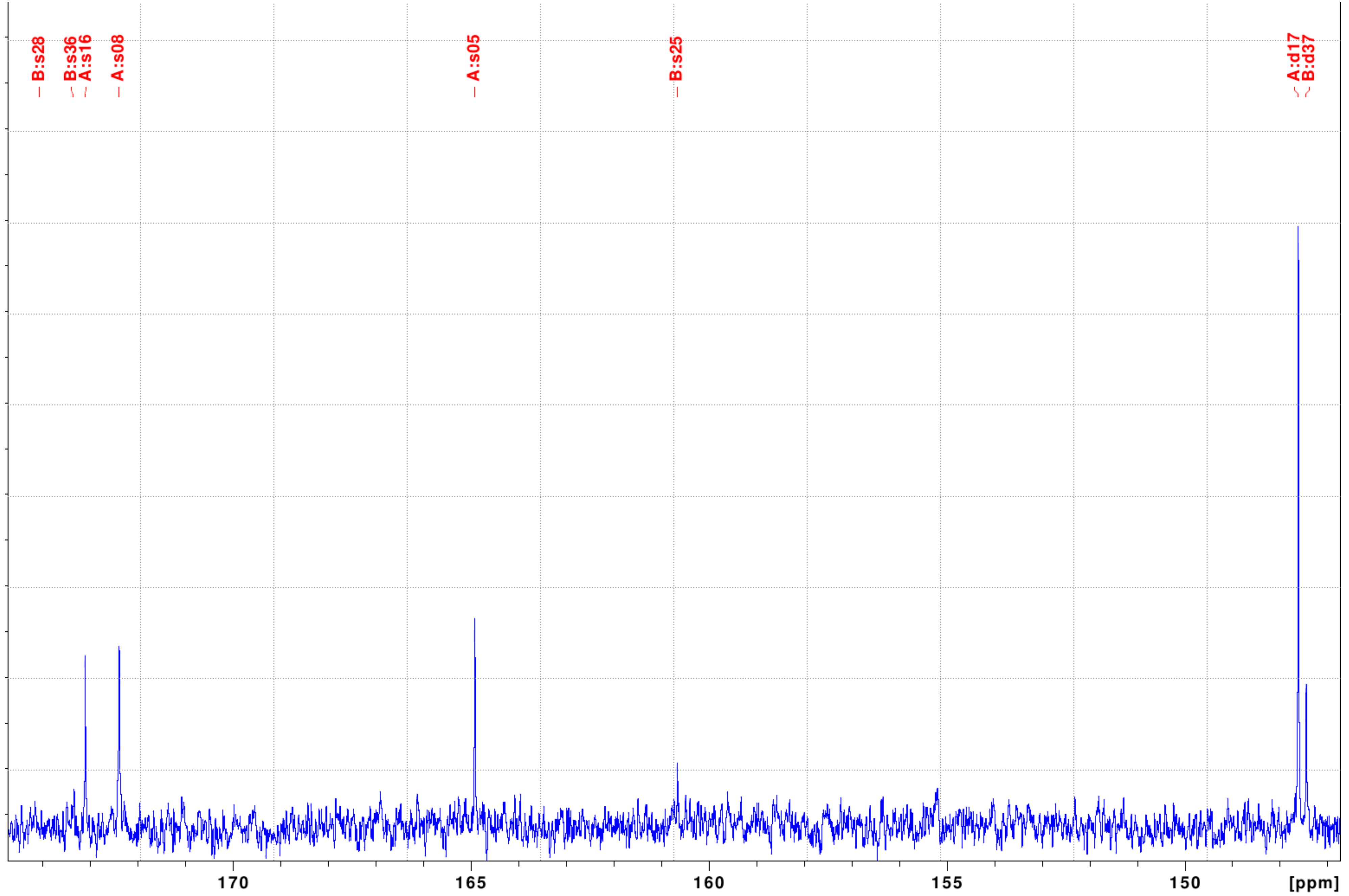
B:s28
B:s36
A:s16
A:s08
A:s05
B:s25
A:d17
B:d37
170
165
160
155
150
[ppm]

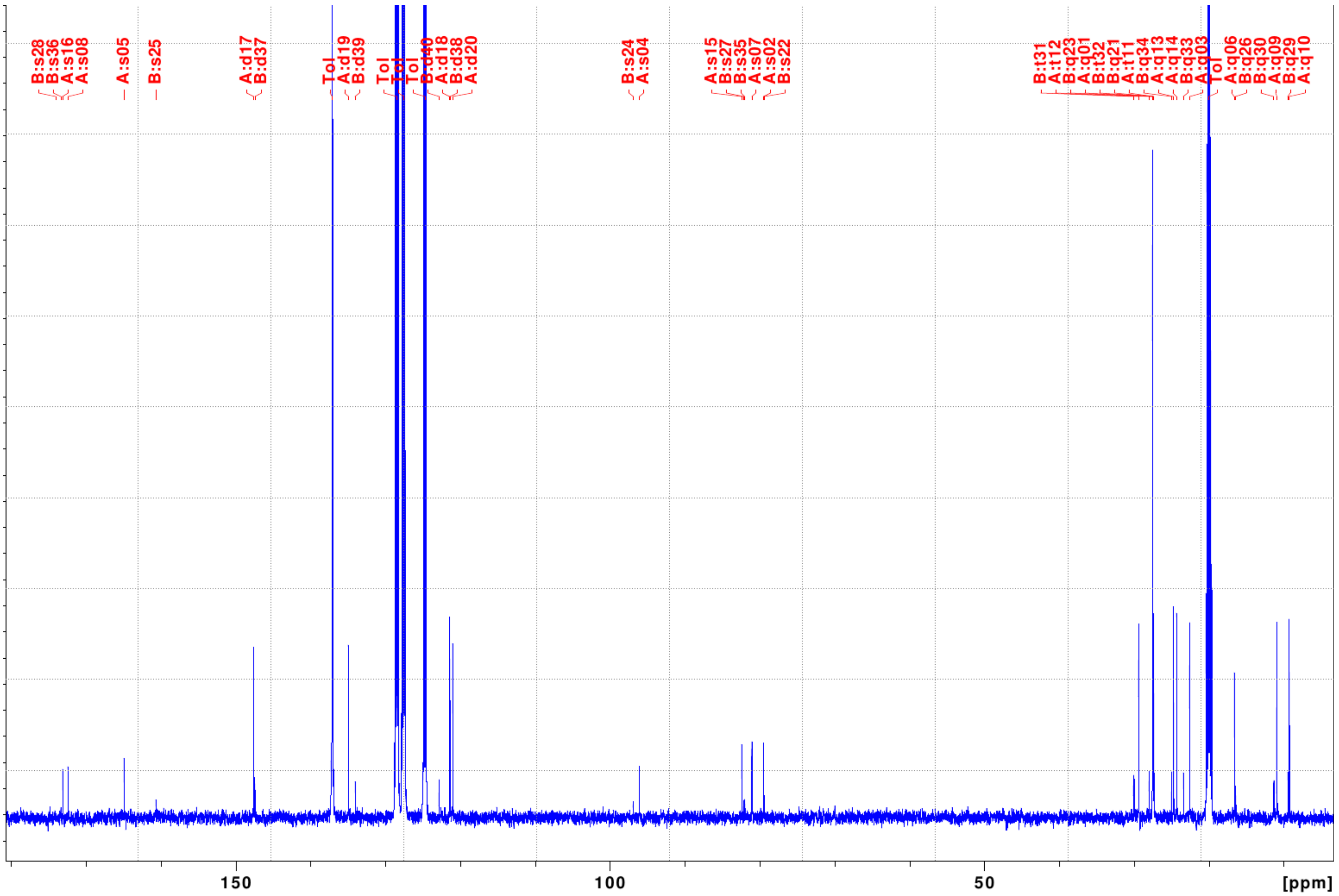
B:s28
B:s36
A:s16
A:s08
A:s05
B:s25
A:d17
B:d37
Tol
A:d19
B:d39
Tol
Tol
Tol
B:d40
A:d18
B:d38
A:d20
B:s24
A:s04
A:s15
B:s27
B:s35
A:s07
A:s02
B:s22
B:t31
A:t12
B:q23
A:q01
B:t32
B:q21
A:t11
B:q34
A:q13
A:q14
B:q33
A:q03
Tol
A:q06
B:q26
B:q30
A:q09
B:q29
A:q10
150
100
50
[ppm]

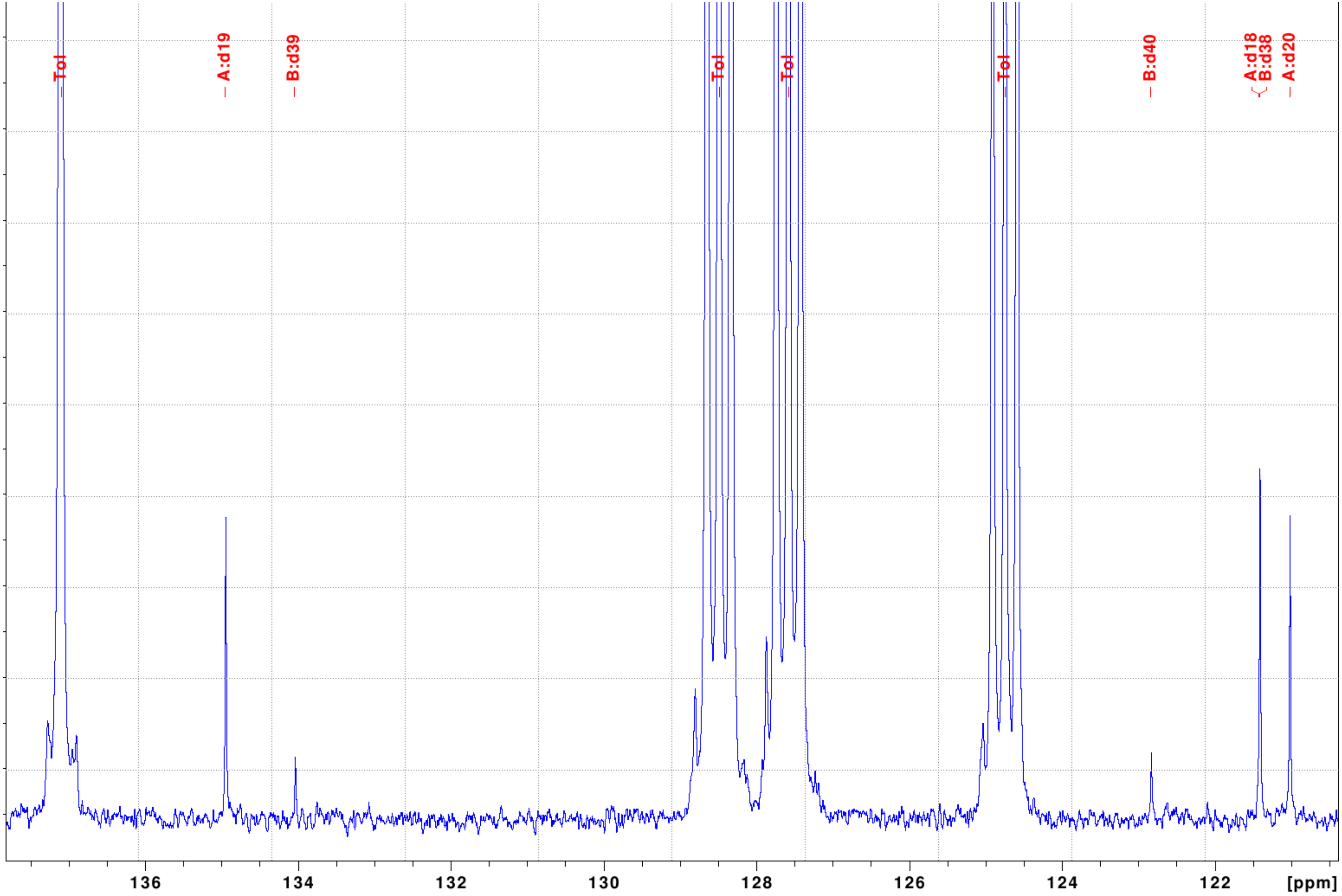

Tol
A:d19
B:d39
Tol
Tol
Tol
B:d40
A:d18
B:d38
A:d20
136
134
132
130
128
126
124
122
[ppm]

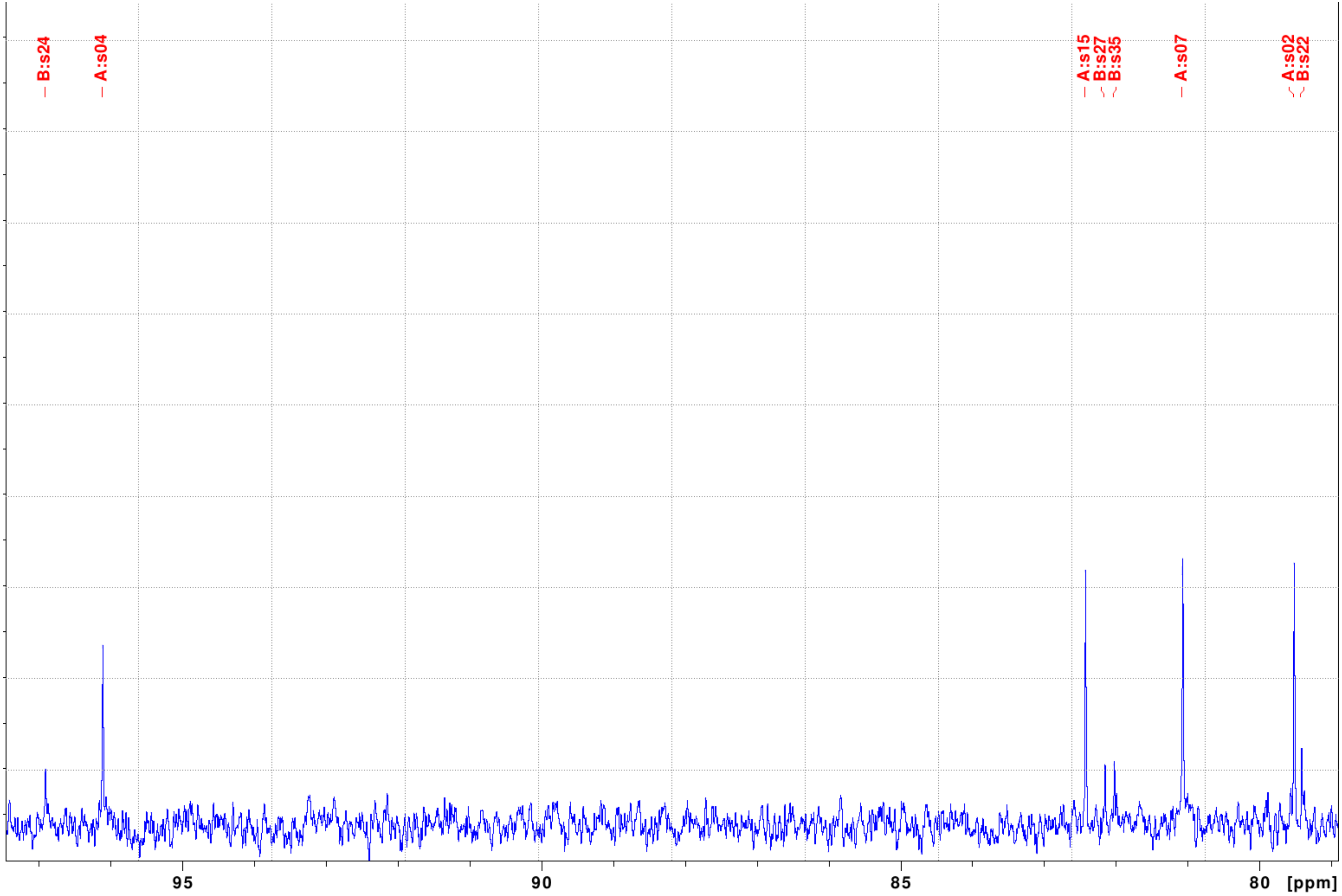

B:s24
A:s04
A:s15
B:s27
B:s35
A:s07
A:s02
B:s22
95
90
85
80
[ppm]

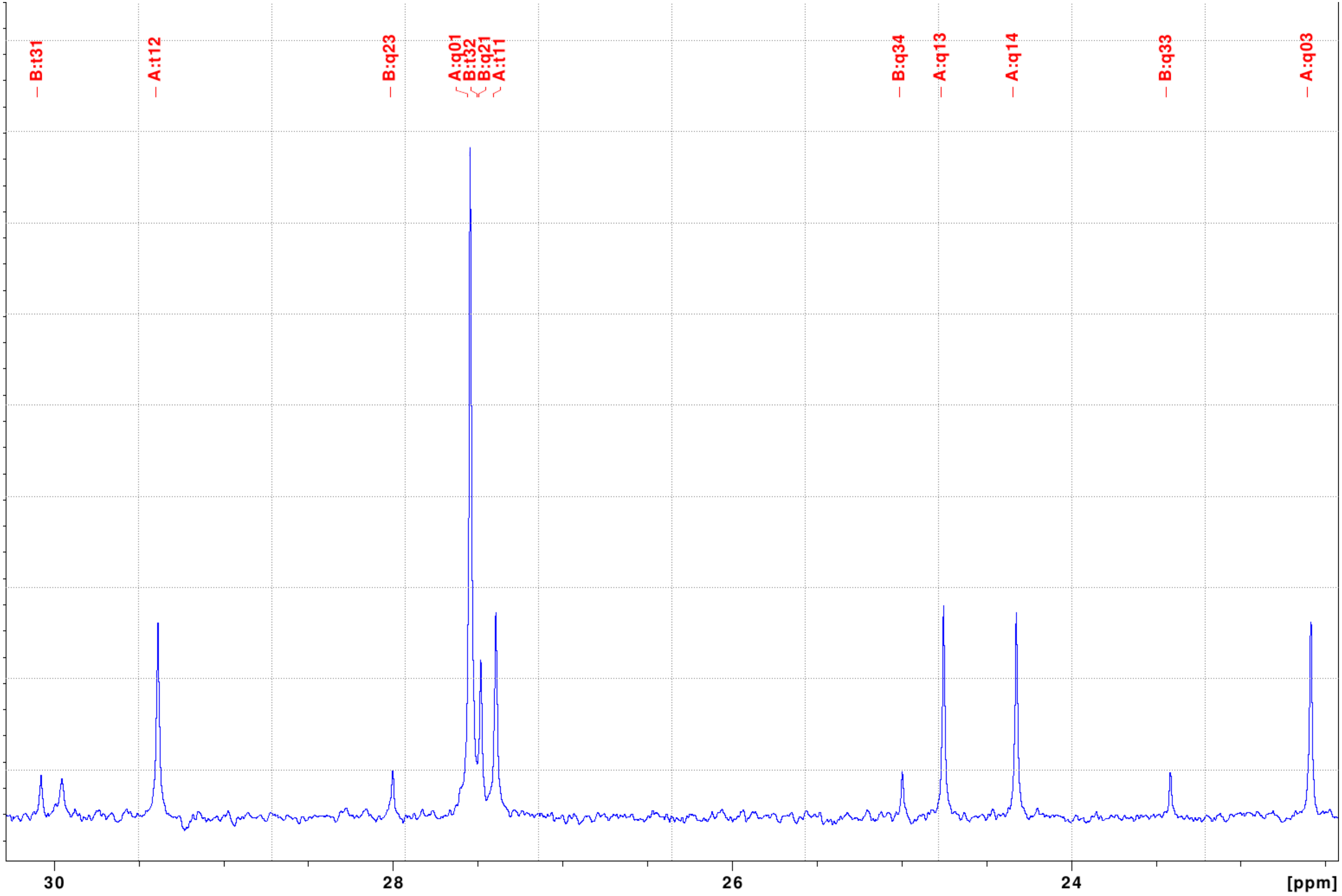

B:t31
A:t12
B:q23
A:q01
B:t32
B:q21
A:t11
B:q34
A:q13
A:q14
B:q33
A:q03
30
28
26
24
[ppm]

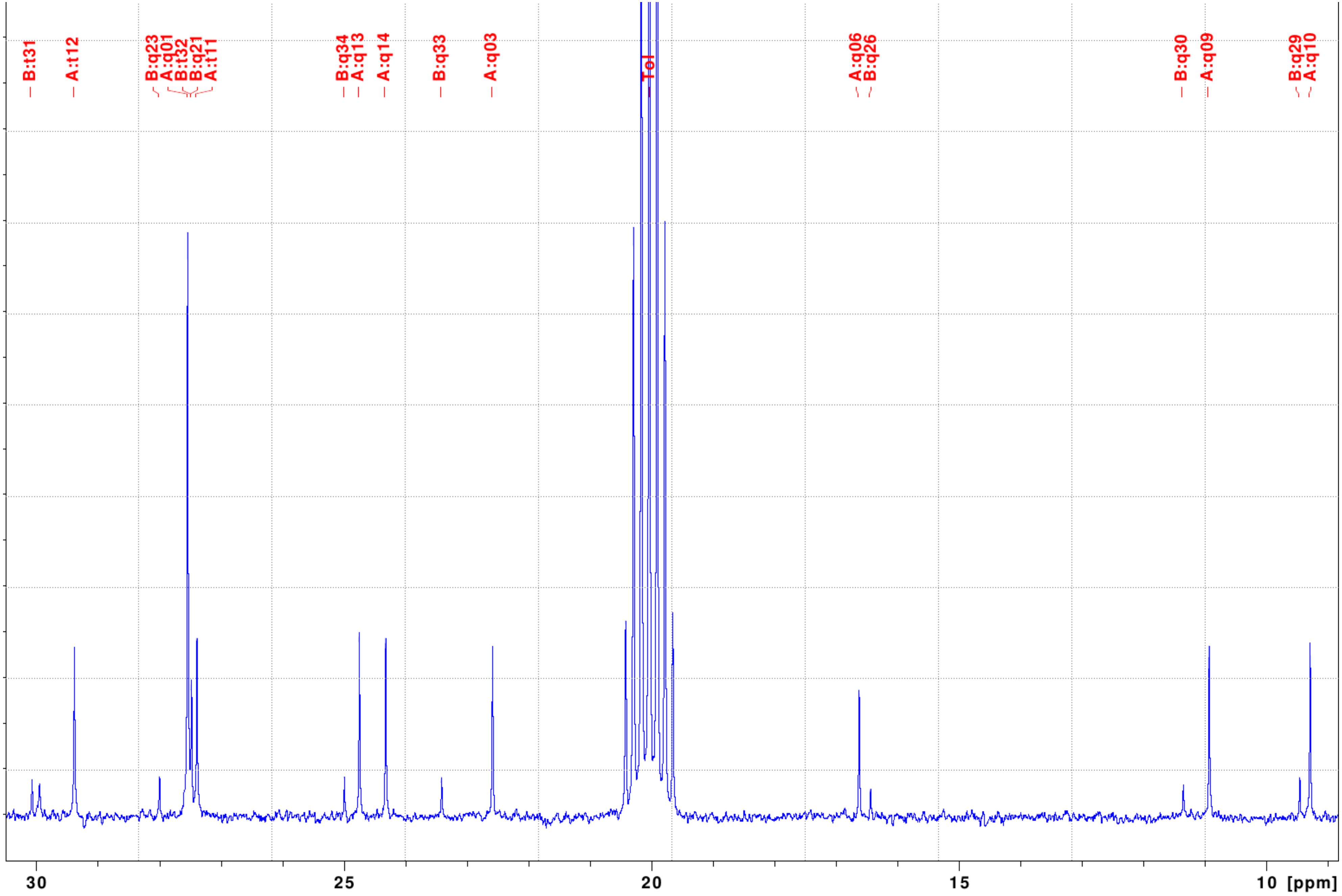
B:t31
A:t12
B:q23
A:q01
B:t32
B:q21
A:t11
B:q34
A:q13
A:q14
B:q33
A:q03
Tol
A:q06
B:q26
B:q30
A:q09
B:q29
A:q10
30
25
20
15
10 [ppm]

Structures and NMR signal assignments for products in the reaction mixture 3 + TEMPO

in toluene-d_8 at 25 °C

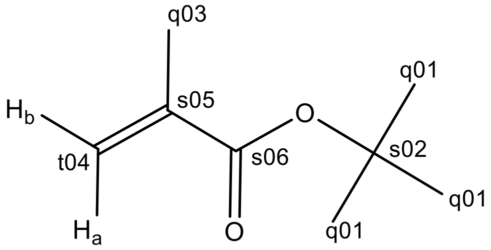

Signal assignments

Some peak labels in NMR spectra could not be assigned to structures because of low product content.

Alcox **3** + TEMPO Tol after strong heating, alkt_1

Experiment Bruker_248, 1D 13C: 8 peaks
q01 28.0
s02 79.3
q03 18.3
t04 123.5
s05 138.4
s06 165.8
d07 119.2
t08 29.9

Experiment Bruker_247, 1D 1H: 6 peaks
q01-H 1.40
q03-H 1.84
t04-a 5.21
t04-b 6.03
d07-H 7.62
t08-a 1.66
t08-b 1.66

Experiment Bruker_252, 2D 13C-1H via onebond (HSQC): 6 peaks
d07-H - d07
q01-H - q01(127 Hz)
q03-H - q03(128 Hz)
t04-a - t04(159 Hz)
t04-b - t04(161 Hz)
t08-a - t08
t08-b - t08

Experiment Bruker_253, 2D 13C-1H via Jcoupling (HMBC): 10 peaks
q01-H - q01 s02
q03-H - s05 s06 t04
t04-a - q03
t04-b - q03 s05 s06
t08-a - q09
t08-b - q09

Experiment Bruker_250, 2D 1H-1H via Jcoupling (COSY): 6 peaks
q03-H - t04-a t04-b
t04-a - q03-H t04-b
t04-b - q03-H t04-a

The system has 4 distinct fragment(s)

Fragment 1:
q01
s02

Fragment 2:
q03
t04
s05
s06

Fragment 3:
d07

Fragment 4:
t08

$^{13}C\{^1H\}$ NMR spectrum (150 MHz)

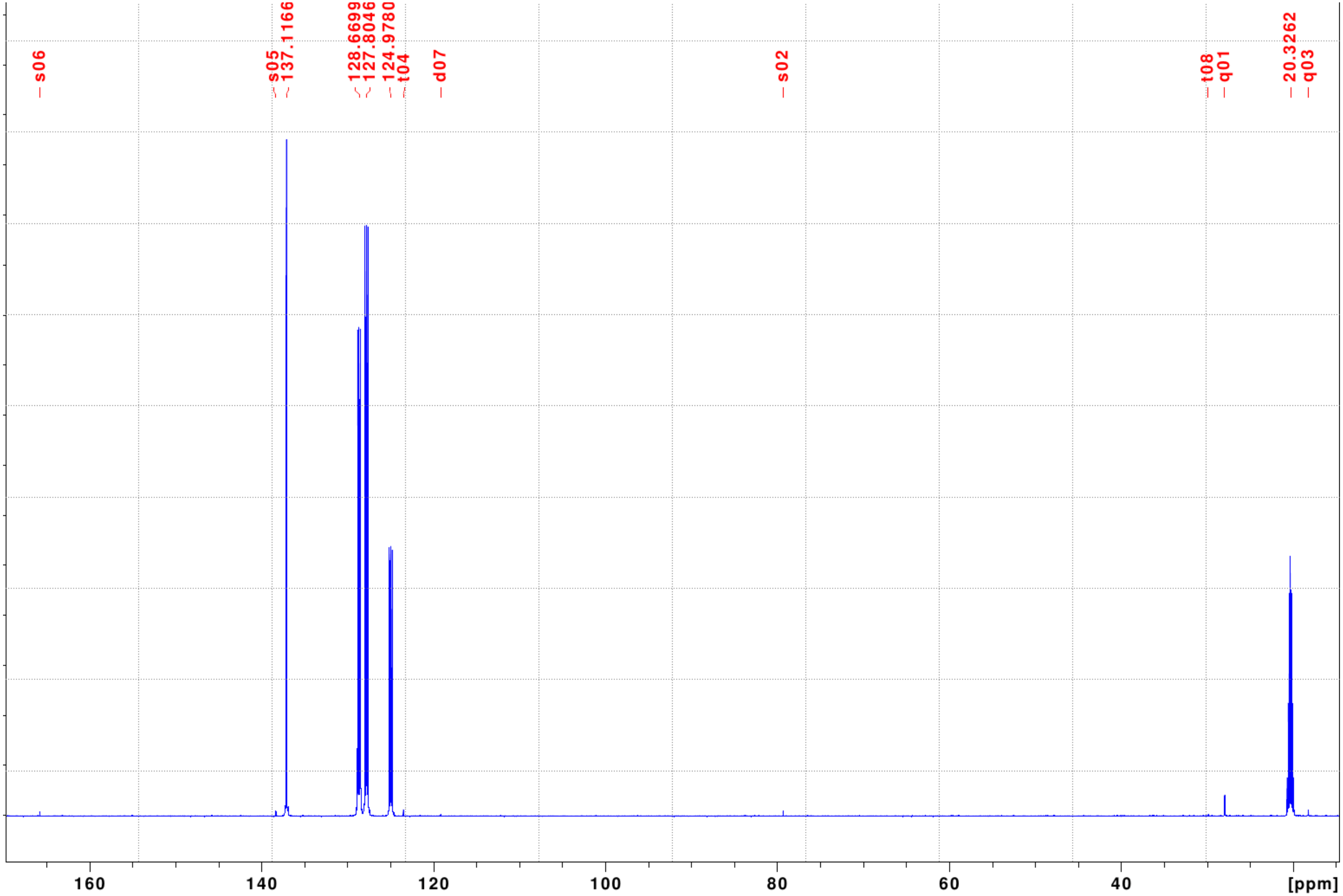

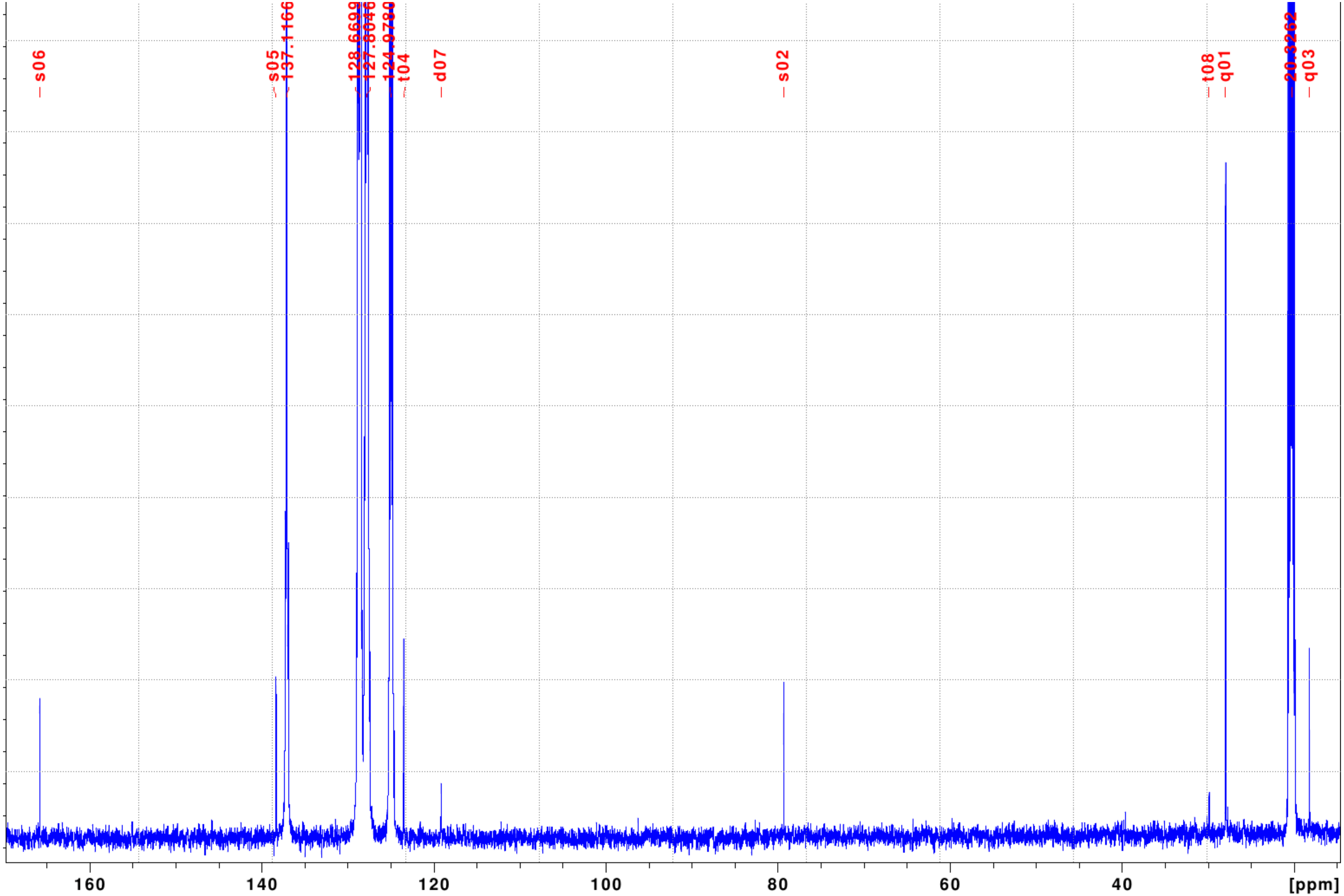

s06
s05
137.1166
128.6699
127.8046
124.9780
t04
d07
s02
t08
q01
20.3262
q03
160
140
120
100
80
60
40
[ppm]

^{1}H NMR spectrum (600 MHz)

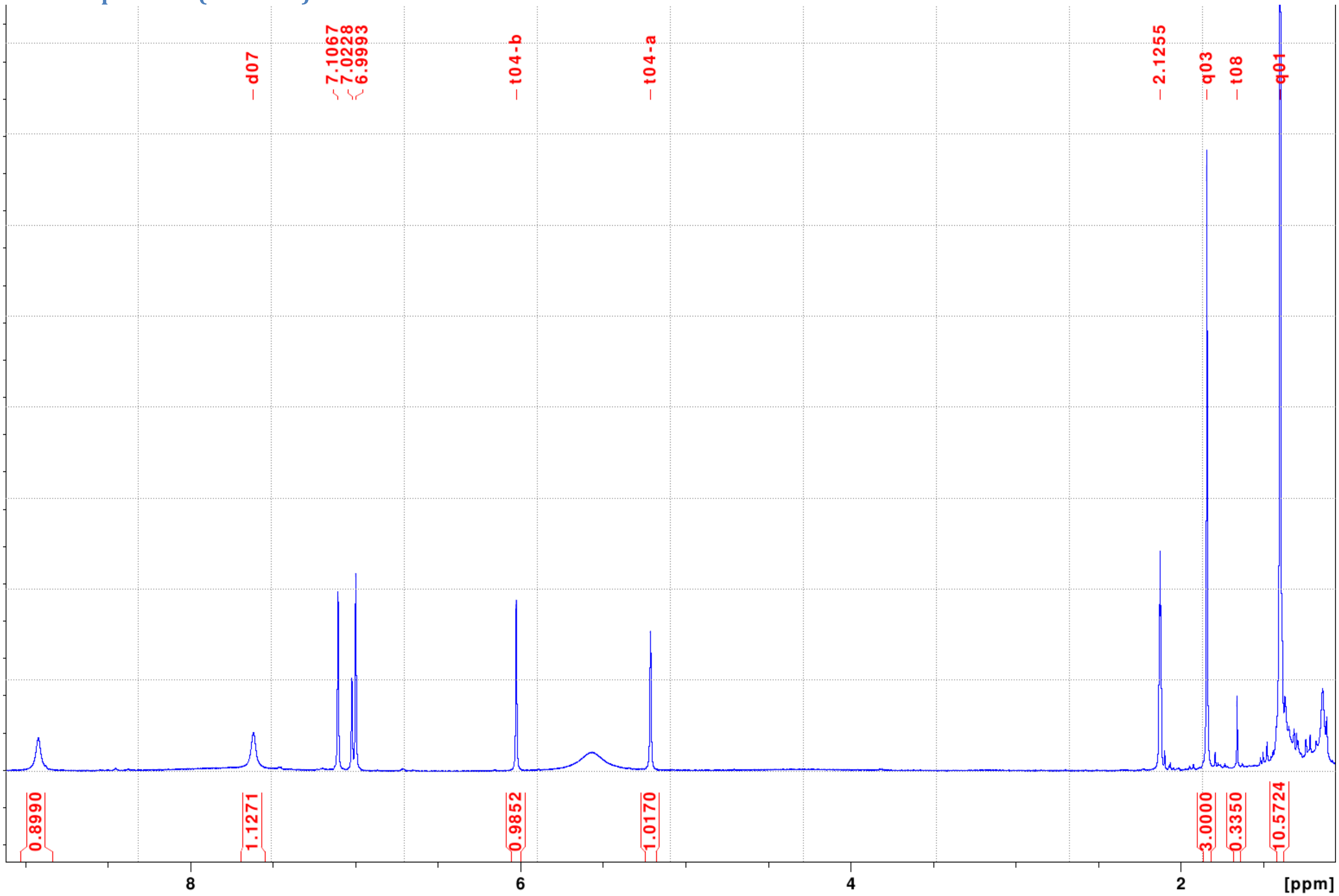

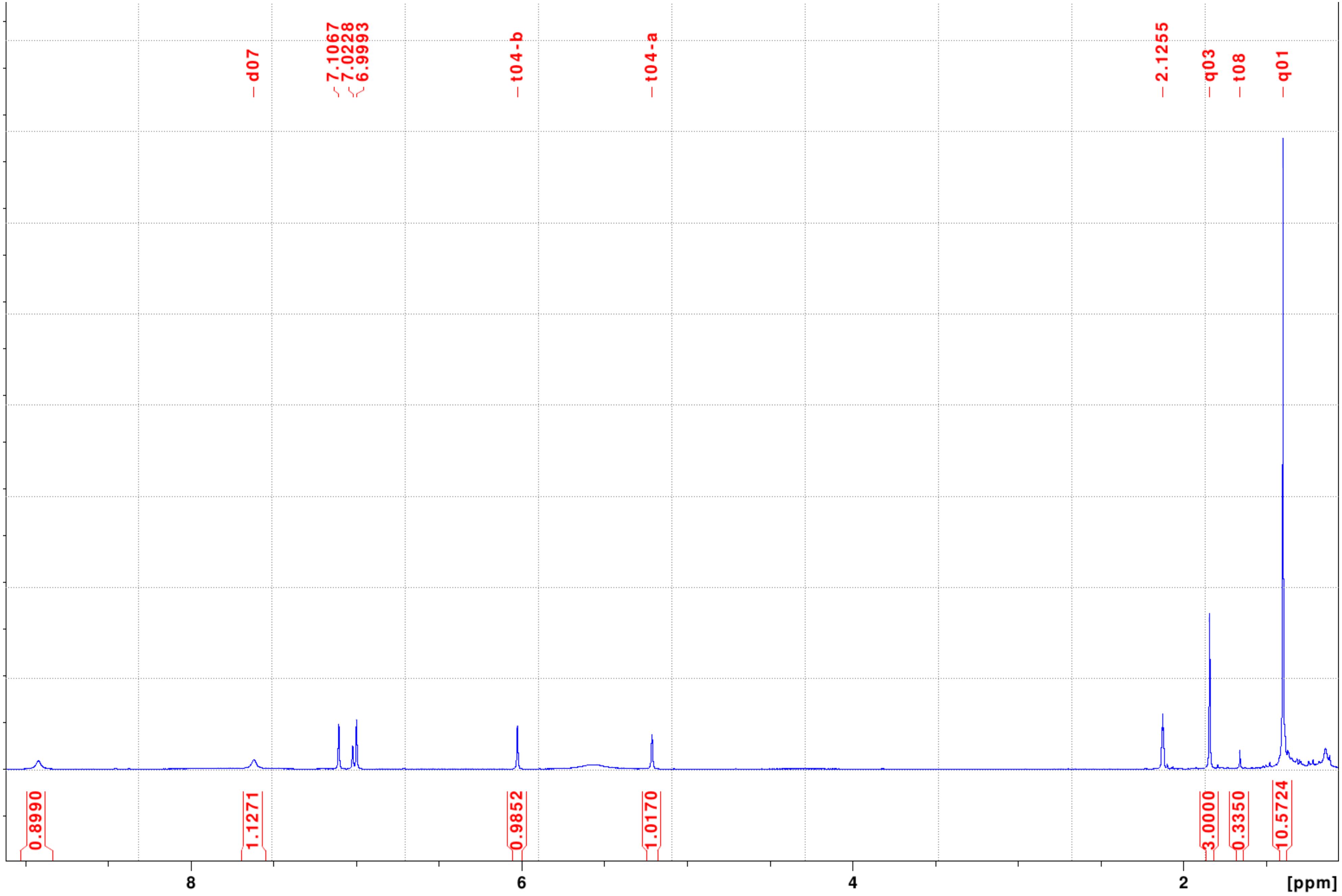
d07
7.1067
7.0228
6.9993
t04-b
t04-a
2.1255
q03
t08
q01
0.8990
1.1271
0.9852
1.0170
3.0000
0.3350
10.5724
8
6
4
2
[ppm]

Structures and NMR signal assignments for products in the reaction mixture $2^{RS/SR}$ + TEMPO

in toluene-d_8 at 25 °C

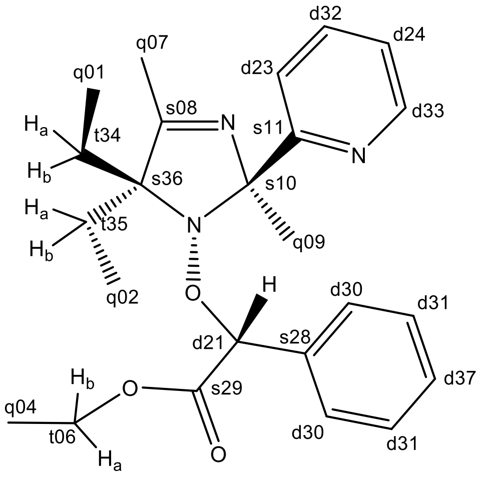

$2^{RR/SS}$

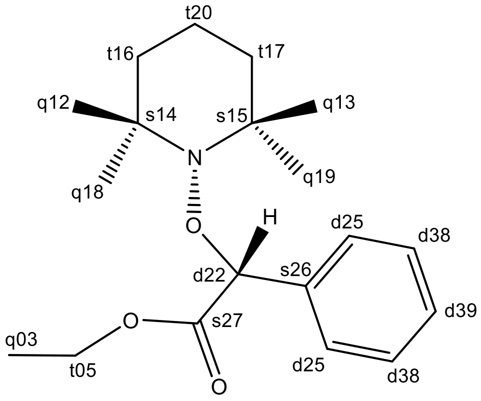

6

Signal assignments

Some peak labels in NMR spectra could not be assigned to structures because of low product content.

Experiment Bruker_592, 1D 13C: 39 peaks

d21 83.7
d22 89.2
d23 120.4
d24 121.9
d25 127.3
d30 128.2
d31 128.7
d32 135.8
d33 149.1
d37 128.7
d38 128.3
d39 128.2
q01 9.0
q02 11.3
q03 14.2
q04 14.2
q07 16.7
q09 24.8
q12 33.1
q13 33.8
q18 20.3
q19 20.1
s08 173.0
s10 97.9
s11 166.1
s14 59.8
s15 59.7
s26 138.9
s27 171.0
s28 136.6
s29 172.5
s36 82.4
t05 60.6
t06 60.3
t16 40.5
t17 40.3
t20 17.4
t34 30.3
t35 27.6

Experiment Bruker_590, 1D 1H: 36 peaks

d21-H 6.77
d22-H 5.37
d23-H 7.55
d24-H 6.69
d25-H 7.55
d30-H 7.81
d31-H 7.19
d32-H 7.15
d33-H 8.61
d37-H 7.10
d38-H 7.14
d39-H 7.05
q01-H 0.44
q02-H 1.10
q03-H 0.93
q04-H 0.88
q07-H 1.78
q09-H 1.94
q12-H 1.36
q13-H 0.83
q18-H 1.36
q19-H 1.13
t05-a 3.85
t05-b 3.94
t06-a 3.77
t06-b 3.97
t16-a 1.37
t16-b 1.47
t17-a 1.27
t17-b 1.37
t20-a 1.16
t20-b 1.47
t34-a 1.38
t34-b 2.10
t35-a 1.55
t35-b 2.85

Experiment Bruker_595, 2D 13C-1H via onebond (HSQC): 38 peaks

d21-H - d21
d22-H - d22(150 Hz)
d23-H - d23
d24-H - d24
d25-H - d25
d30-H - d30
d31-H - d31
d32-H - d32
d33-H - d33
d37-H - d37
d38-H - d38
d39-H - d39
q01-H - q01
q02-H - q02
q03-H - q03
q04-H - q04
q07-H - q07
q09-H - q09(130 Hz)
q12-H - q12
q13-H - q13
q18-H - q18
q19-H - q19
t05-a - t05
t05-b - t05
t06-a - t06
t06-b - t06
t16-a - t16? t17?
t16-b - t16
t17-a - t17

t17-b - t16? t17?
t20-a - t20
t20-b - t20
t34-a - t34
t34-b - t34
t35-a - t35
t35-b - t35

Experiment Bruker_598, 2D 1H-13C via onebond (H-C correlation): 25 peaks
d21 - d21-H
d22 - d22-H
d23 - d23-H
d24 - d24-H
d25 - d25-H
d30 - d30-H
d31 - d31-H
d32 - d32-H
d33 - d33-H
d37 - d37-H
d38 - d38-H
d39 - d39-H
q01 - q01-H
q02 - q02-H
q03 - q03-H? q04-H?
q04 - q03-H? q04-H?
q07 - q07-H
q09 - q09-H
q12 - q12-H
q13 - q13-H
q18 - q18-H
q19 - q19-H
t05 - t05-b

Experiment Bruker_594, 2D 1H-1H via Jcoupling (COSY): 56 peaks
d24-H - d32-H d33-H
d25-H - d38-H
d30-H - d31-H
d31-H - d30-H d37-H
d32-H - d24-H
d33-H - d24-H
d37-H - d31-H
d38-H - d25-H d39-H
d39-H - d38-H
q01-H - t34-a t34-b
q02-H - t35-a t35-b
q03-H - t05-a t05-b
q04-H - t06-a t06-b
t05-a - q03-H t05-b
t05-b - q03-H t05-a
t06-a - q04-H t06-b
t06-b - q04-H t06-a
t16-a - t16-b? t20-a t20-b?
t16-b - t16-a? t17-b? t20-a
t17-a - t17-b t20-a
t17-b - t16-b? t17-a t20-a t20-b?
t20-a - t16-a t16-b t17-a t17-b t20-b
t20-b - t16-a? t17-b? t20-a
t34-a - q01-H t34-b
t34-b - q01-H t34-a
t35-a - q02-H t35-b
t35-b - q02-H t35-a

Experiment Bruker_596, 2D 13C-1H via Jcoupling (HMBC): 31 peaks
d21-H - d30 s28 s29
d22-H - d25 s26 s27
d24-H - d33
d25-H - d25
d37-H - d30
d38-H - d38
d39-H - d25
q01-H - t34
q02-H - t35
q03-H - t05
q04-H - t06
q07-H - s08
q09-H - s10 s11
q12-H - q18 s14 t16
q13-H - q19 s15 t17
q18-H - q12 s14 t16
q19-H - q13 s15 t17
t34-a - q01

The system has 12 distinct fragment(s)

Fragment 1: **2**$^{\mathbf{RR/SS}}$, D ~ 0.85e-9
d21
d23
d24
d30
d31
d32
d33
d37
q01
q02
q04
q07
q09
s08
s10
s11
s28
s29
s36
t06
t34
t35

Fragment 2: **6**, D ~ 0.99e-9
d22
d25
d38
d39
q03
q12
q13
q18
q19
s14
s15
s26
s27
t05
t16
t17
t20

$^{13}C\{^{1}H\}$ NMR spectrum (150 MHz)

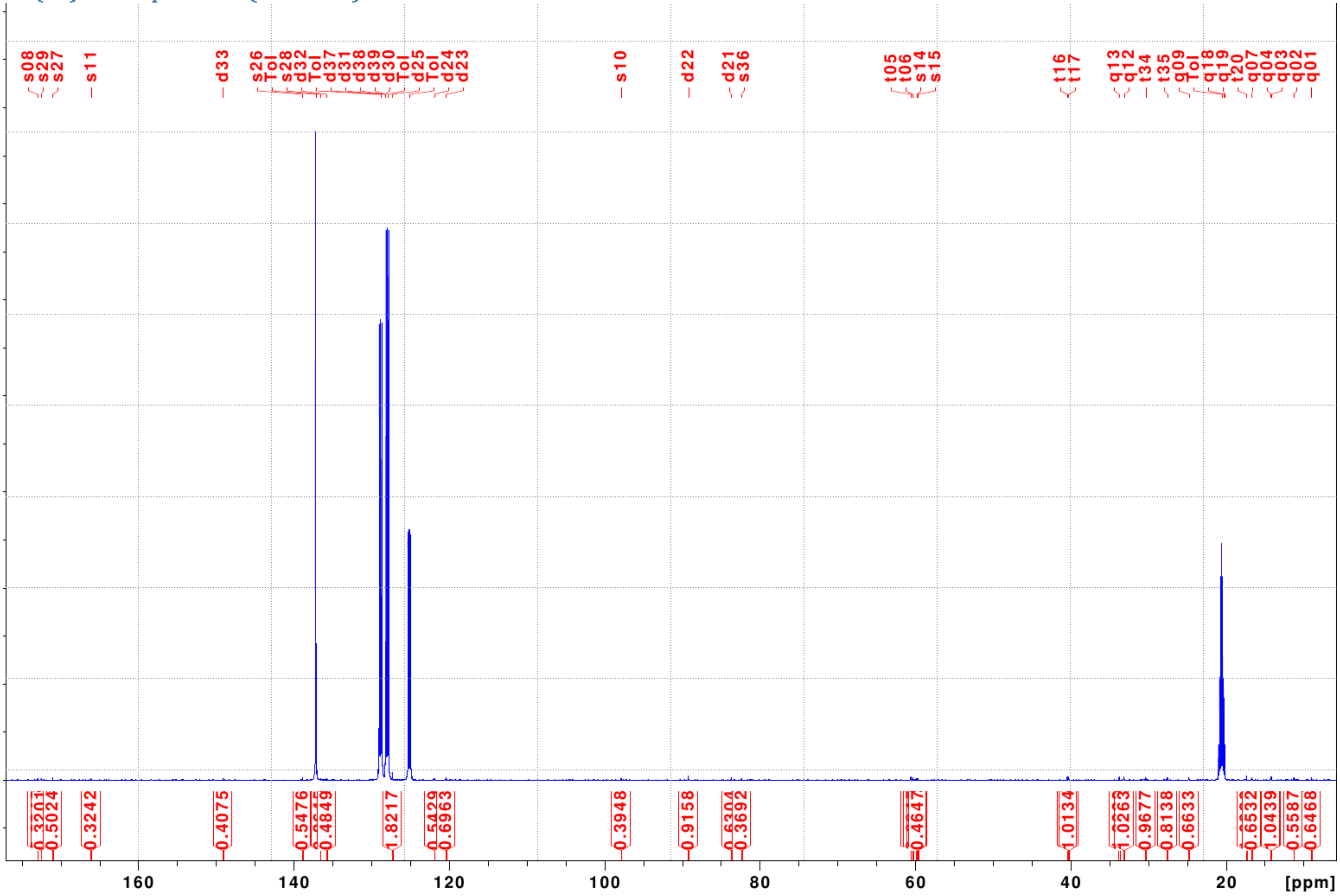

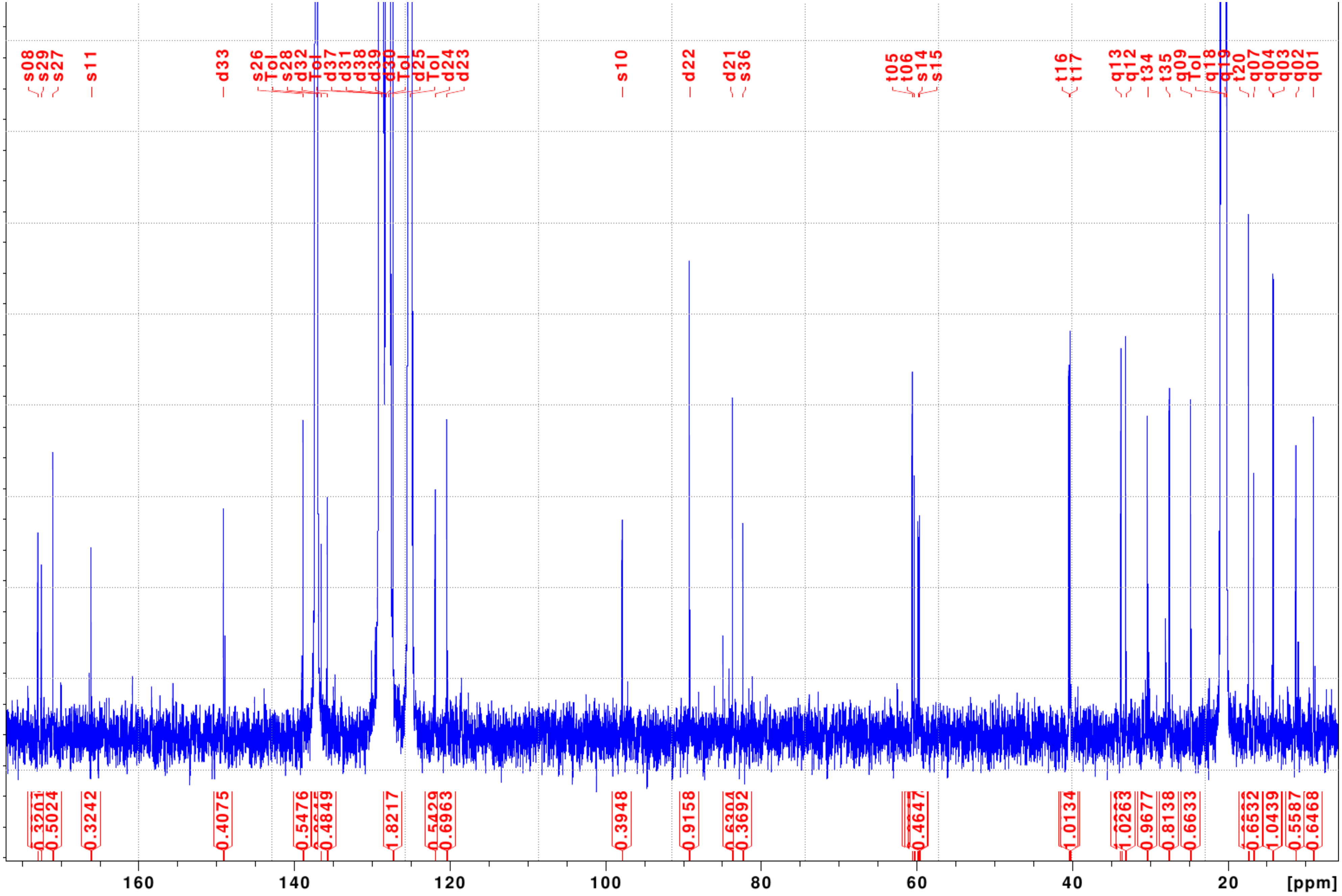

s08
s29
s27
s11
d33
s26
Tol
s28
d32
Tol
d37
d31
d38
d39
d30
Tol
d25
Tol
d24
d23
s10
d22
d21
s36
t05
t06
s14
s15
t16
t17
q13
q12
t34
t35
q09
Tol
q18
q19
t20
q07
q04
q03
q02
q01
0.5024
0.3242
0.4075
0.5476
0.4849
1.8217
0.5429
0.6963
0.3948
0.9158
0.3692
0.4647
1.0134
1.0263
0.9677
0.8138
0.6633
0.6532
1.0439
0.5587
0.6468
160
140
120
100
80
60
40
20
[ppm]

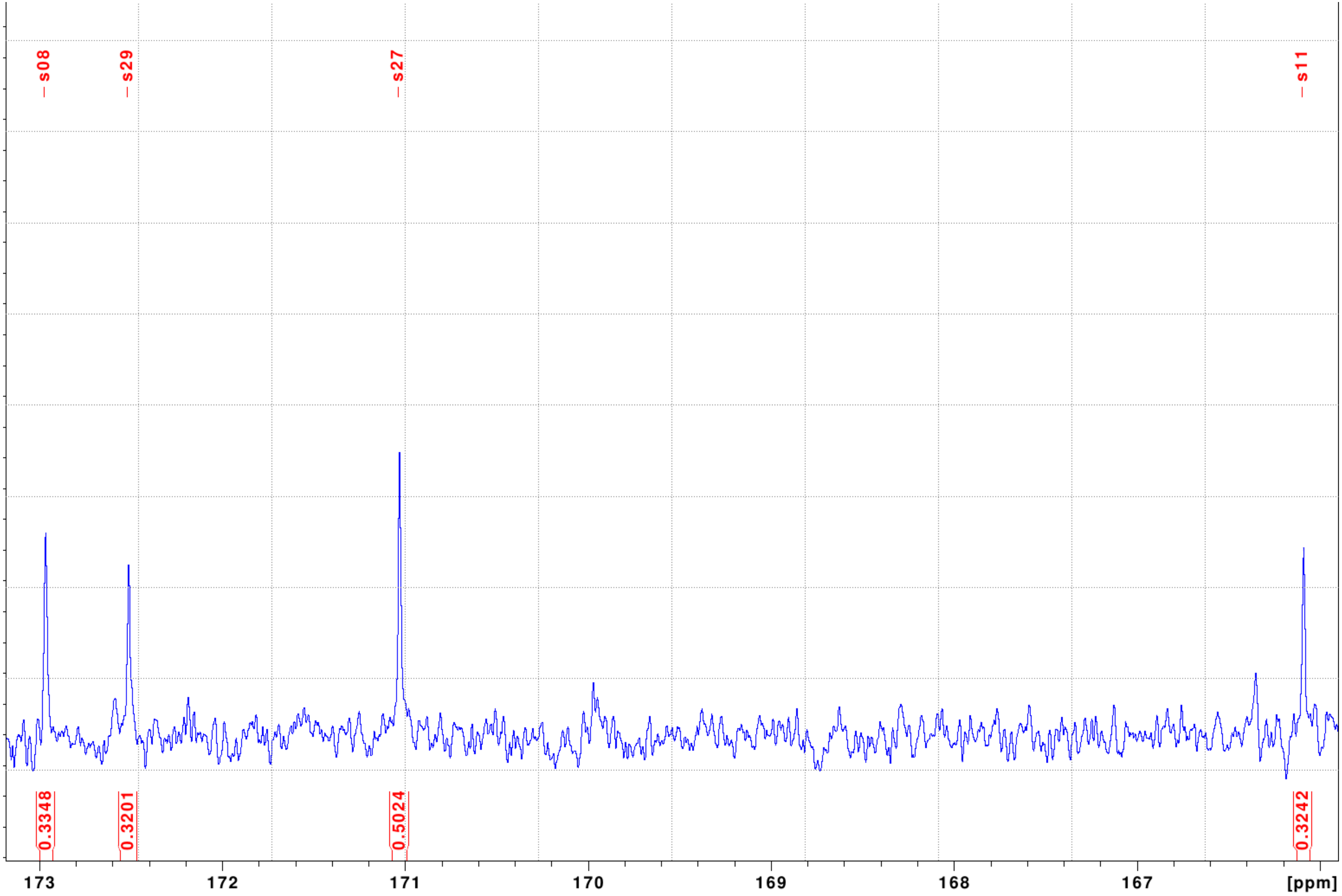
s08
s29
s27
s11
0.3348
0.3201
0.5024
0.3242
173
172
171
170
169
168
167
[ppm]

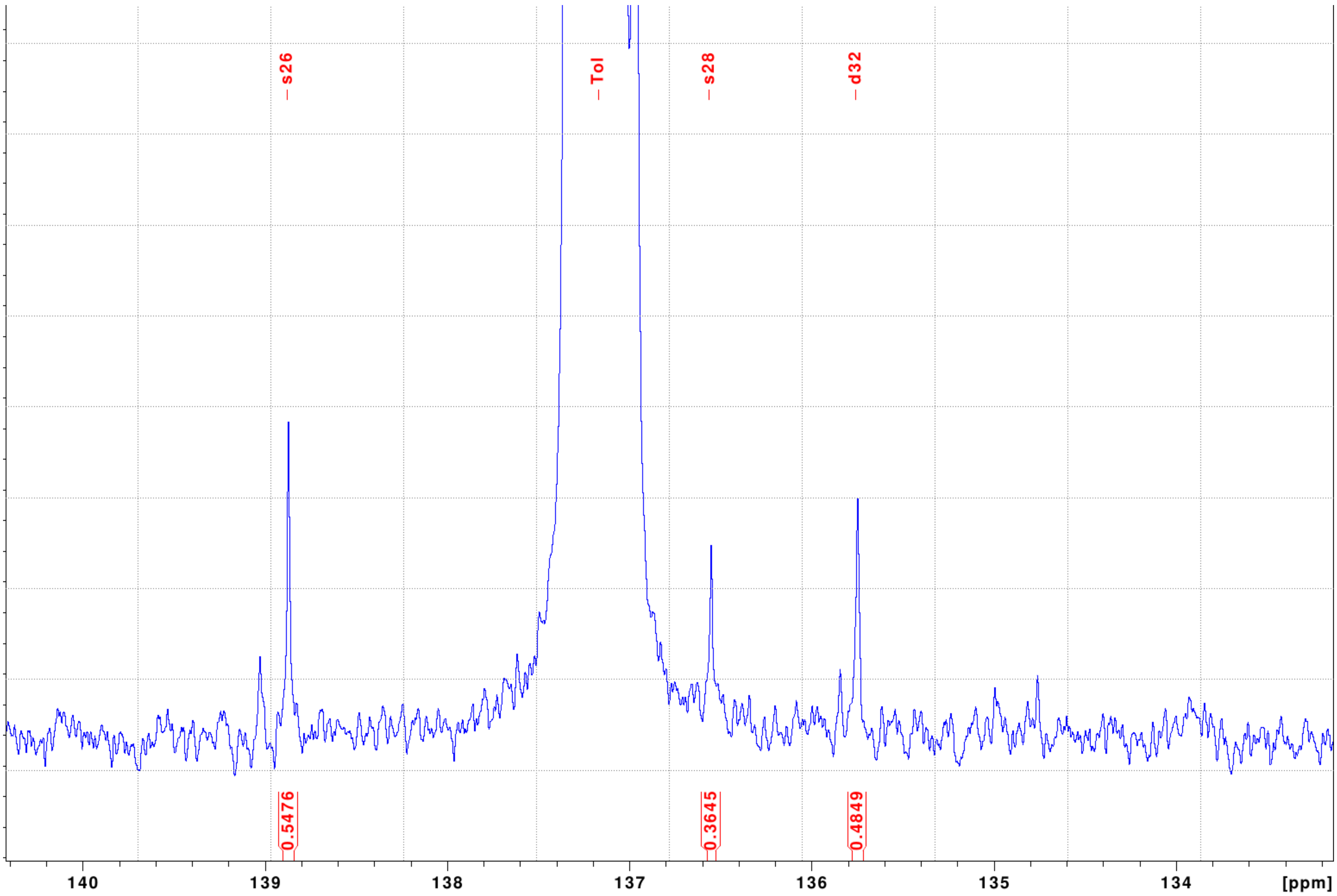
s26
Tol
s28
d32
0.5476
0.3645
0.4849
140
139
138
137
136
135
134
[ppm]

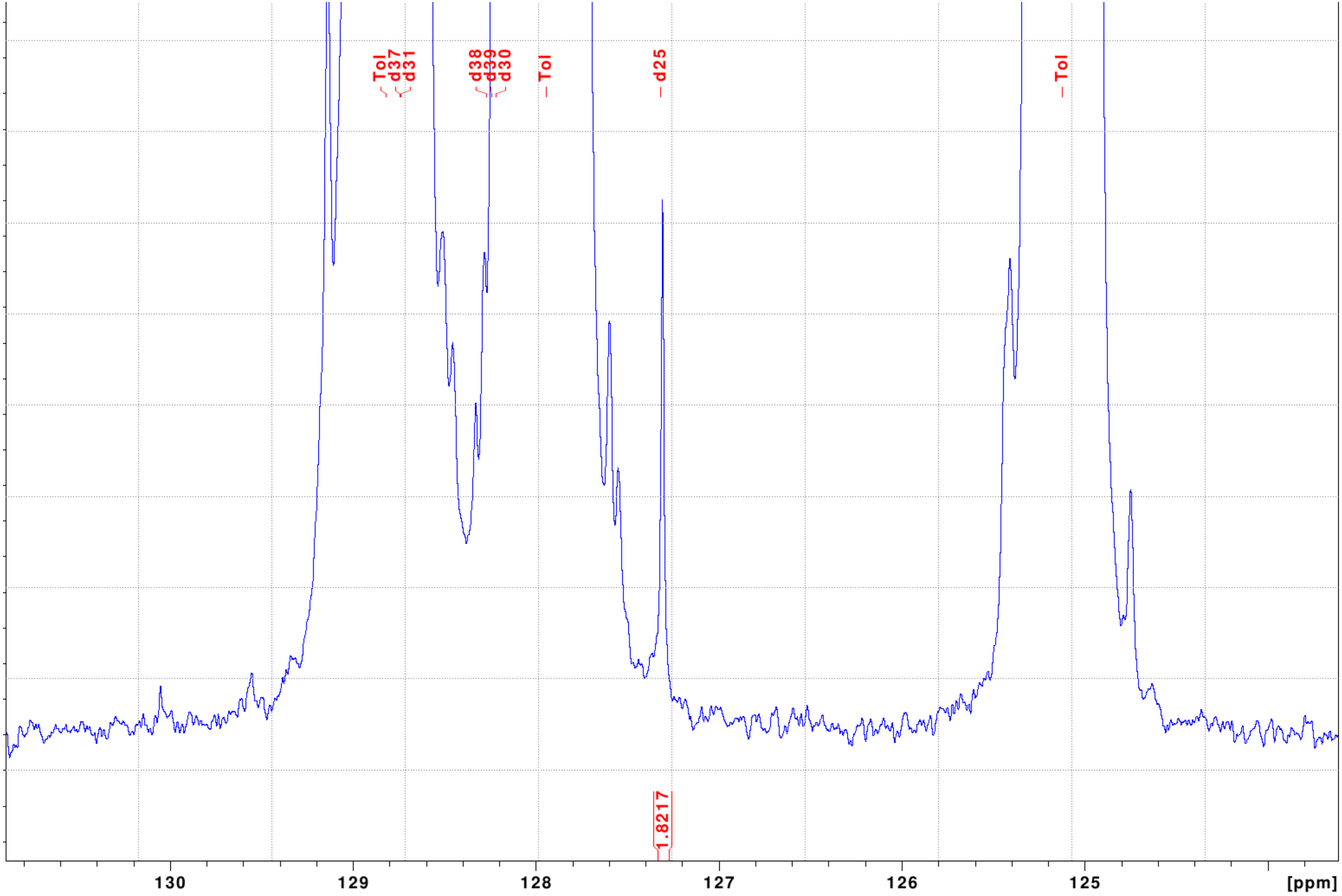

Tol
d37
d31
d38
d39
d30
Tol
d25
Tol
1.8217
130
129
128
127
126
125
[ppm]

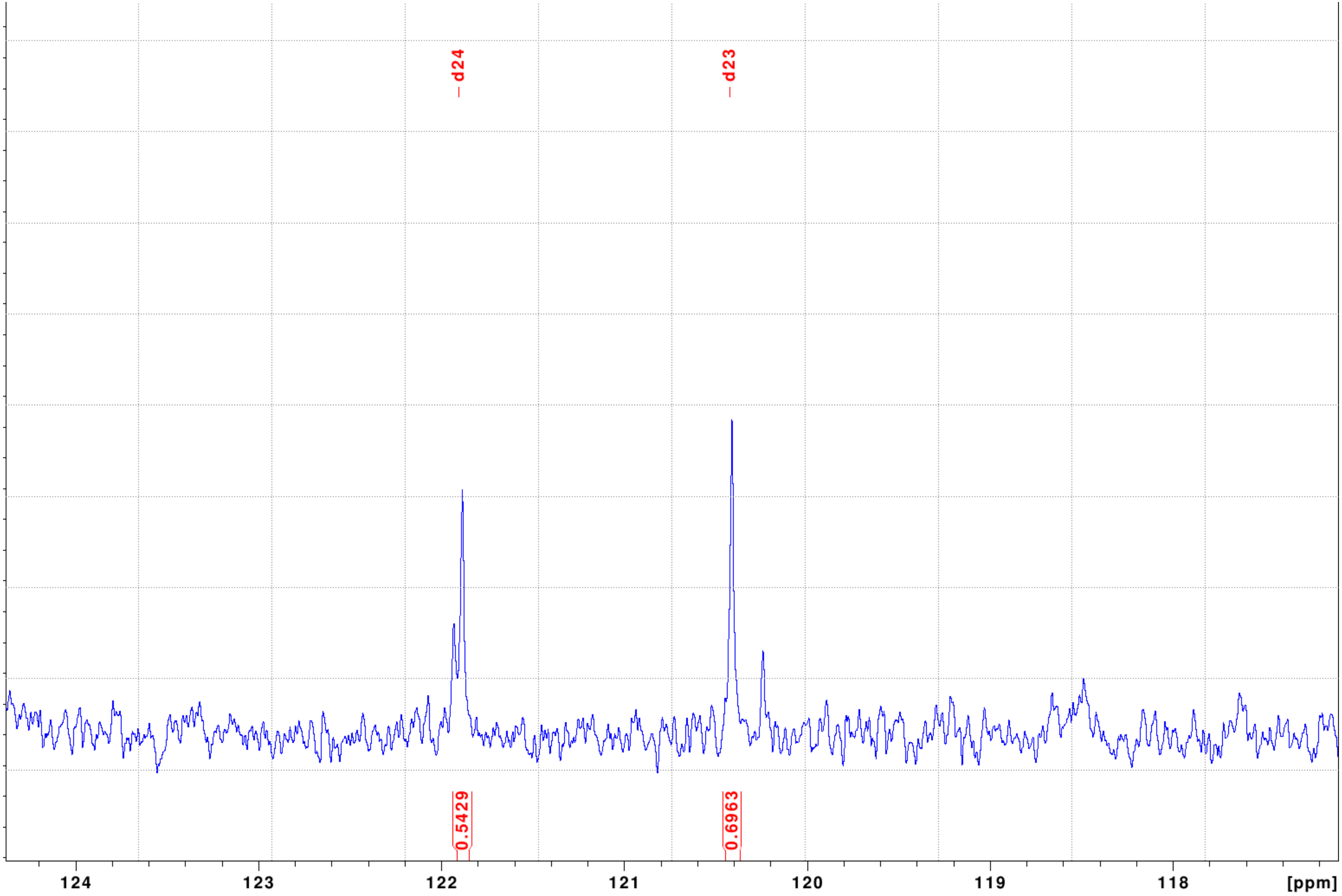
d24
d23
0.5429
0.6963
124
123
122
121
120
119
118
[ppm]

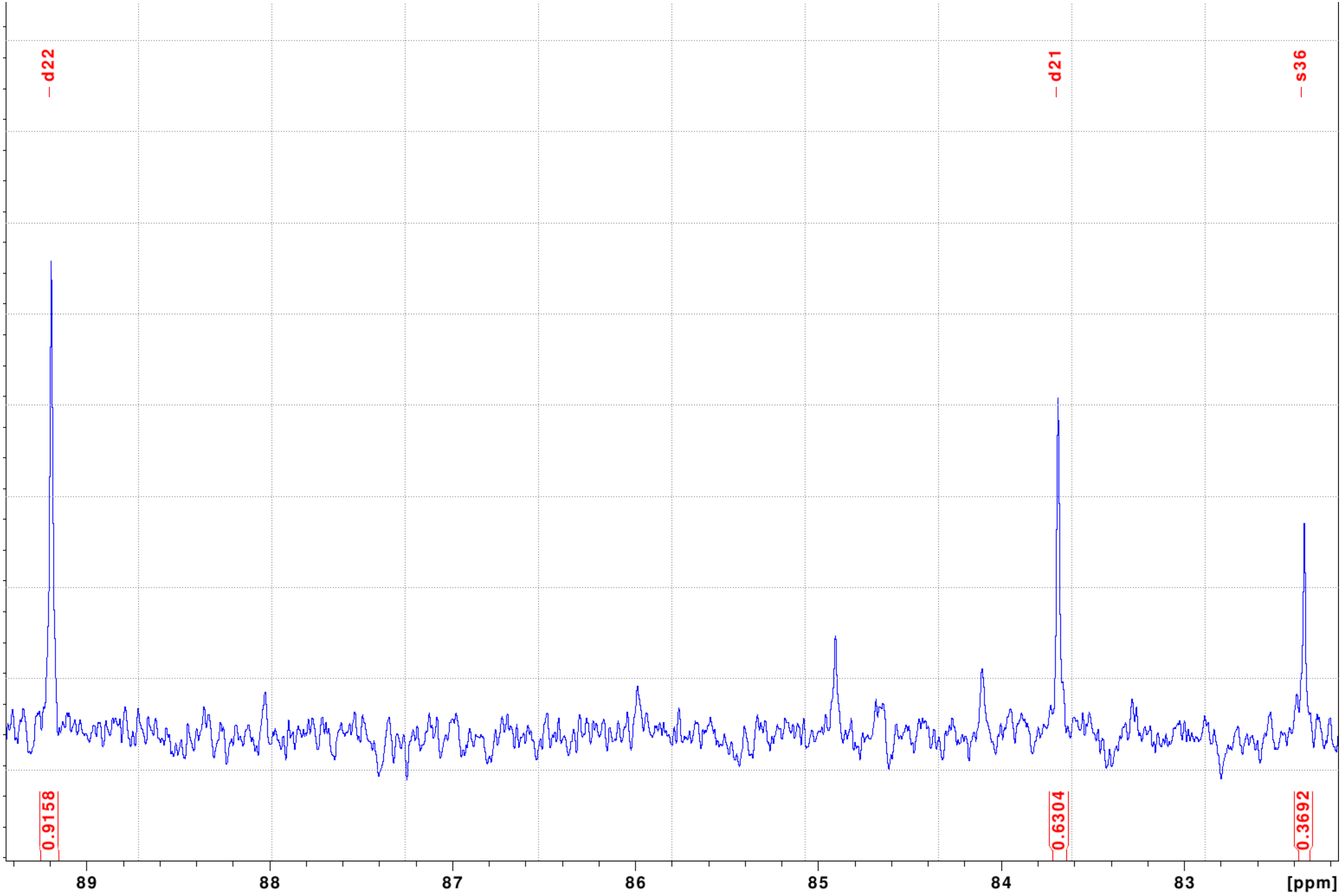

d22
d21
s36
0.9158
0.6304
0.3692
89
88
87
86
85
84
83
[ppm]

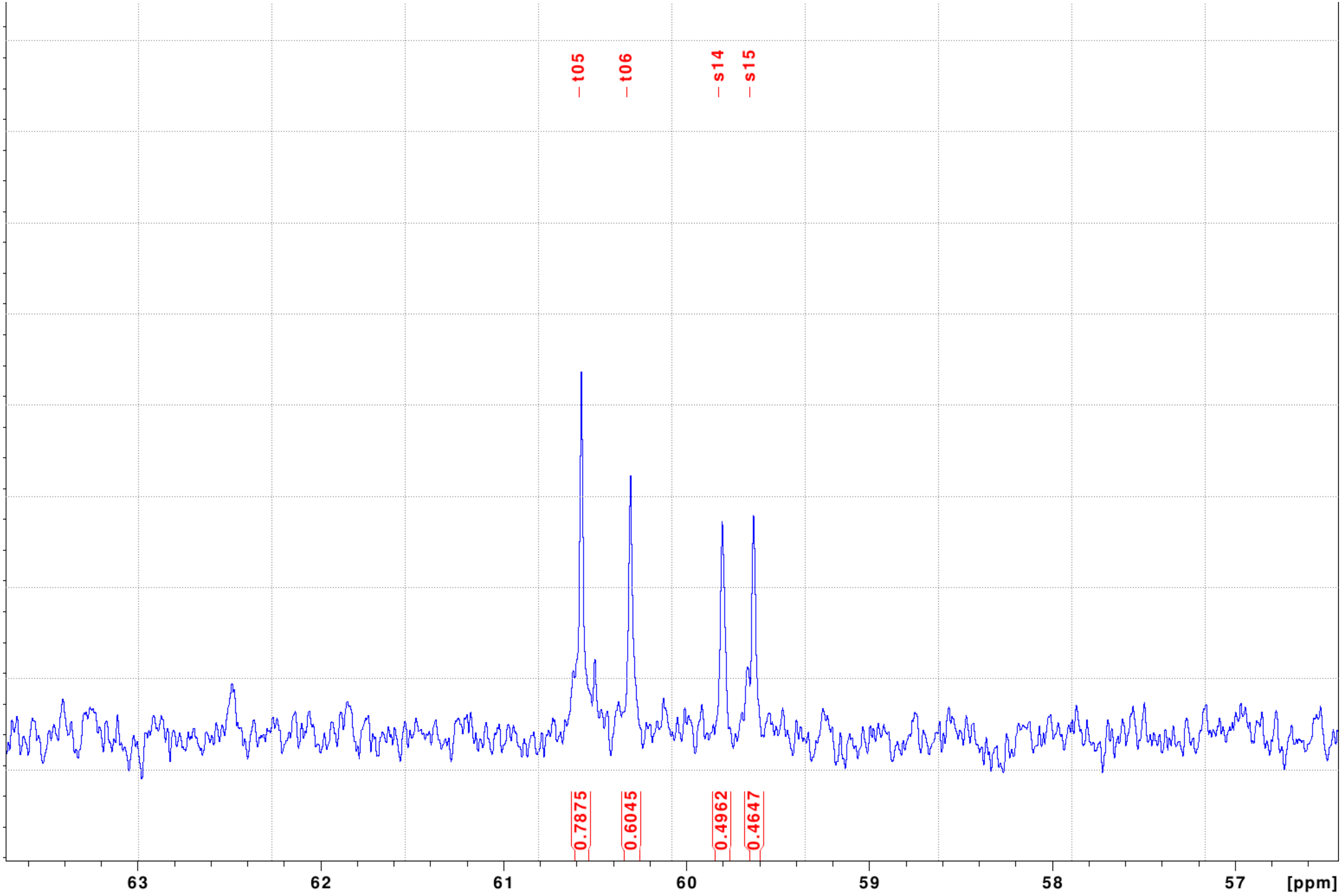

t05
t06
s14
s15
0.7875
0.6045
0.4962
0.4647
63
62
61
60
59
58
57
[ppm]

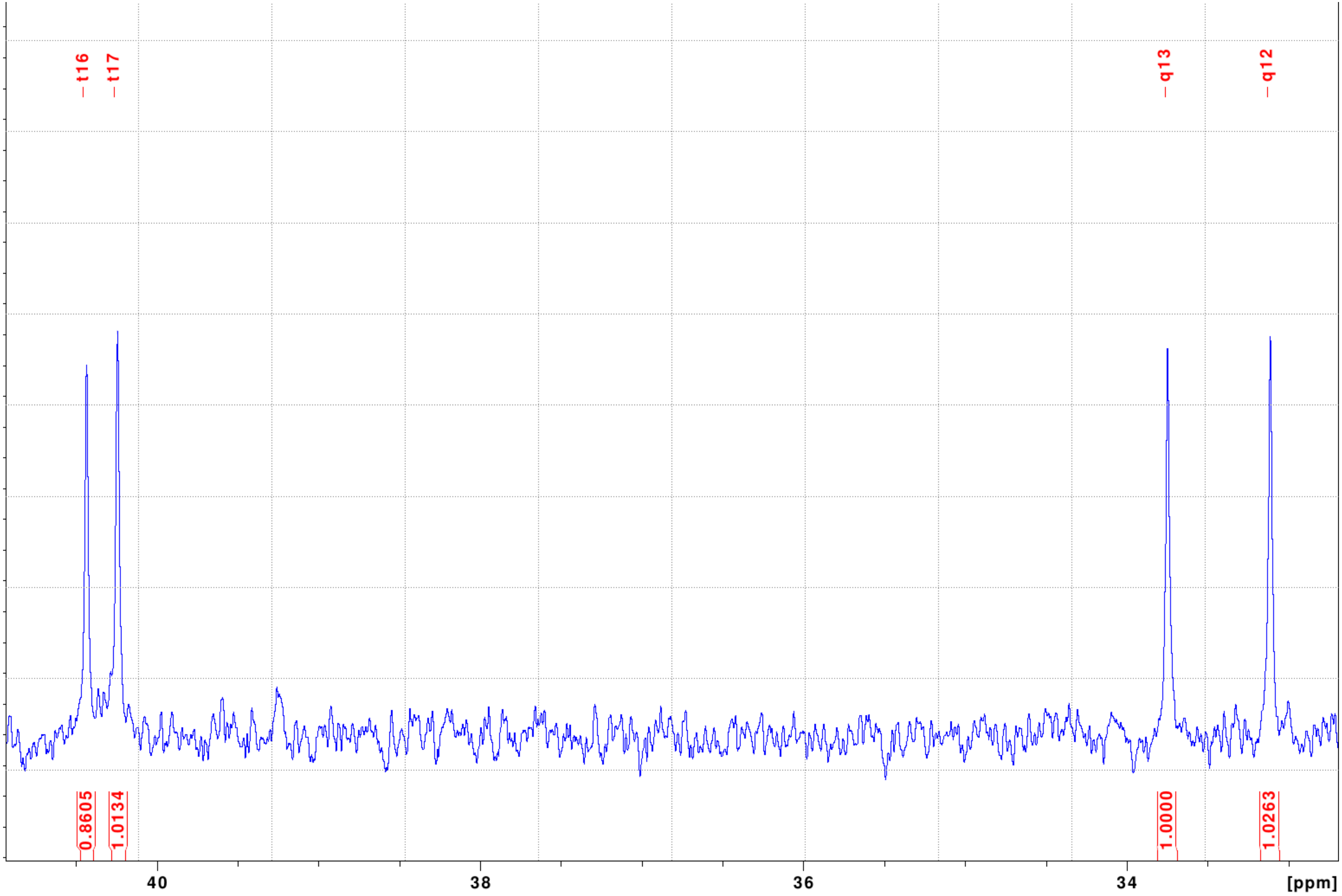
t16
t17
q13
q12
0.8605
1.0134
1.0000
1.0263
40
38
36
34
[ppm]

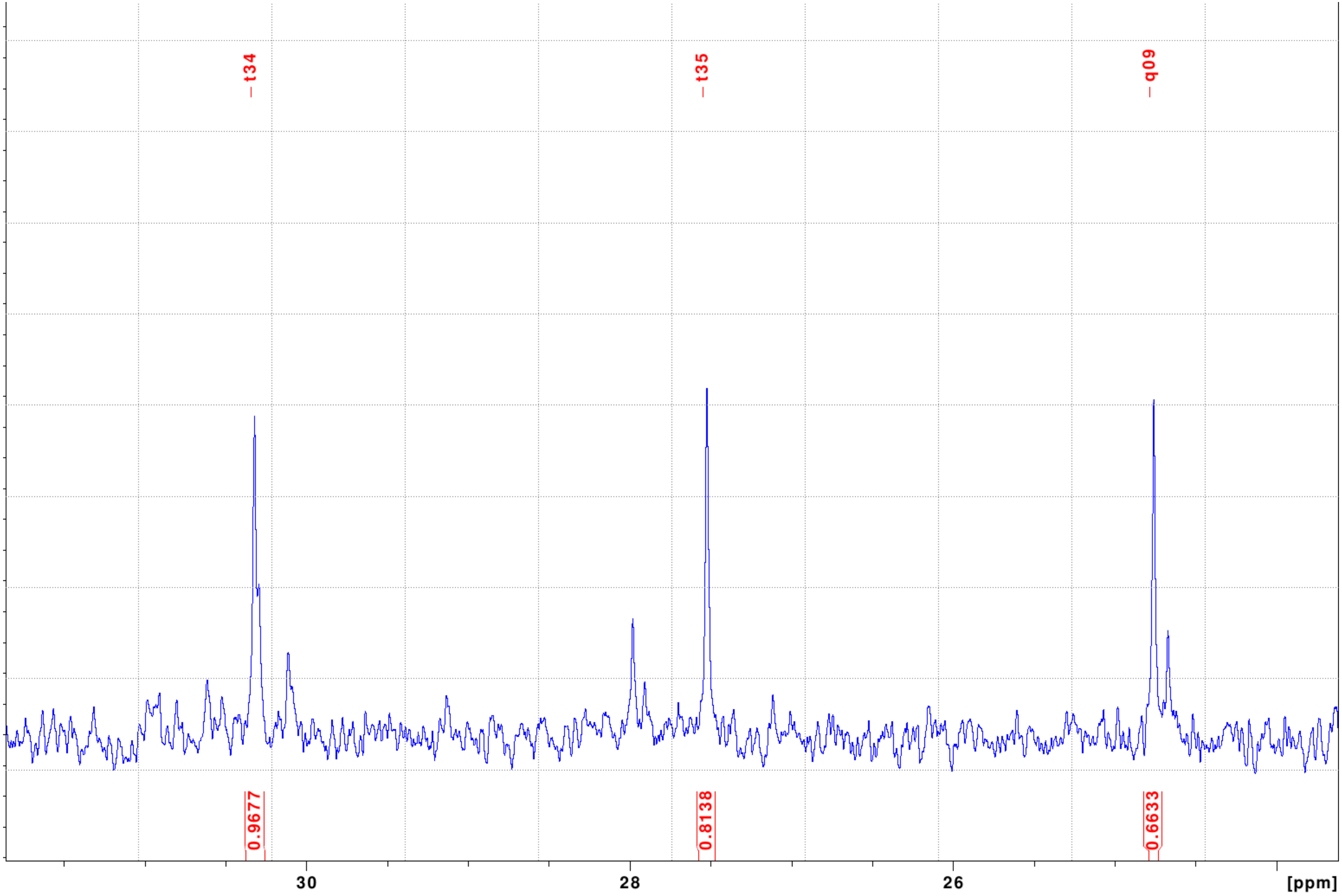

t34
t35
q09
0.9677
0.8138
0.6633
30
28
26
[ppm]

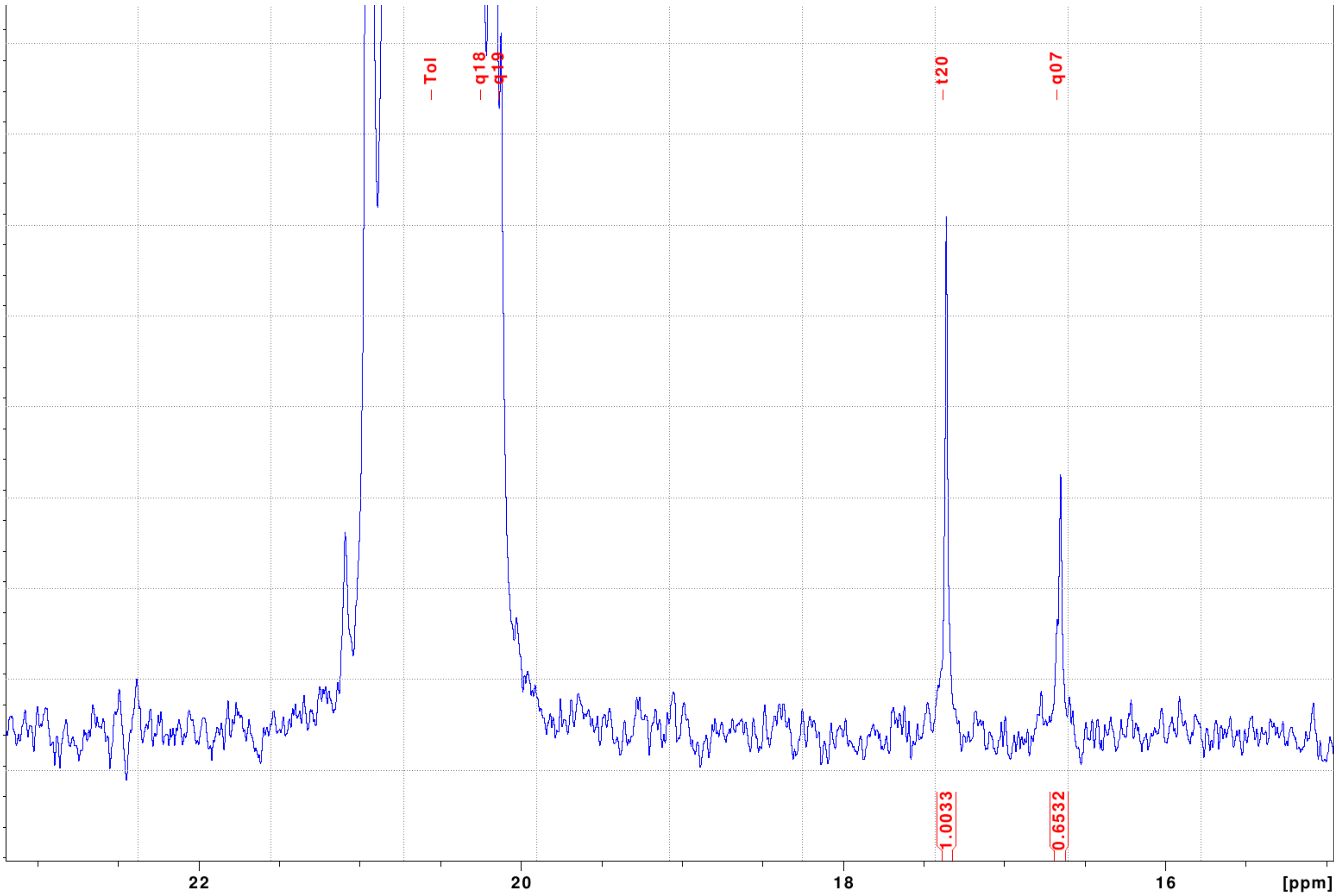

Tol
q18
q19
t20
q07
1.0033
0.6532
22
20
18
16
[ppm]

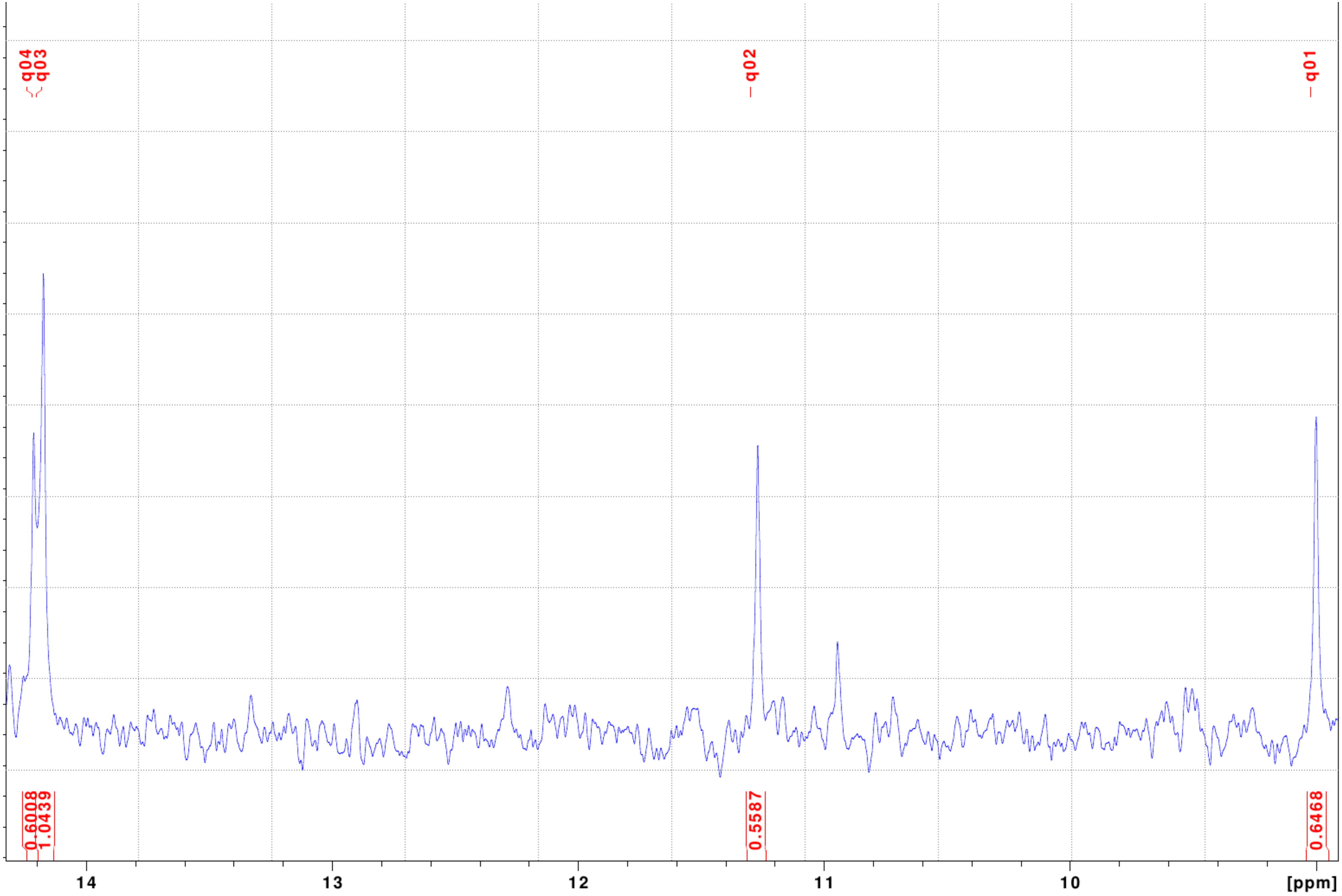
q04
q03
q02
q01
0.6008
1.0439
0.5587
0.6468
14
13
12
11
10
[ppm]

^{1}H NMR spectrum (600 MHz)

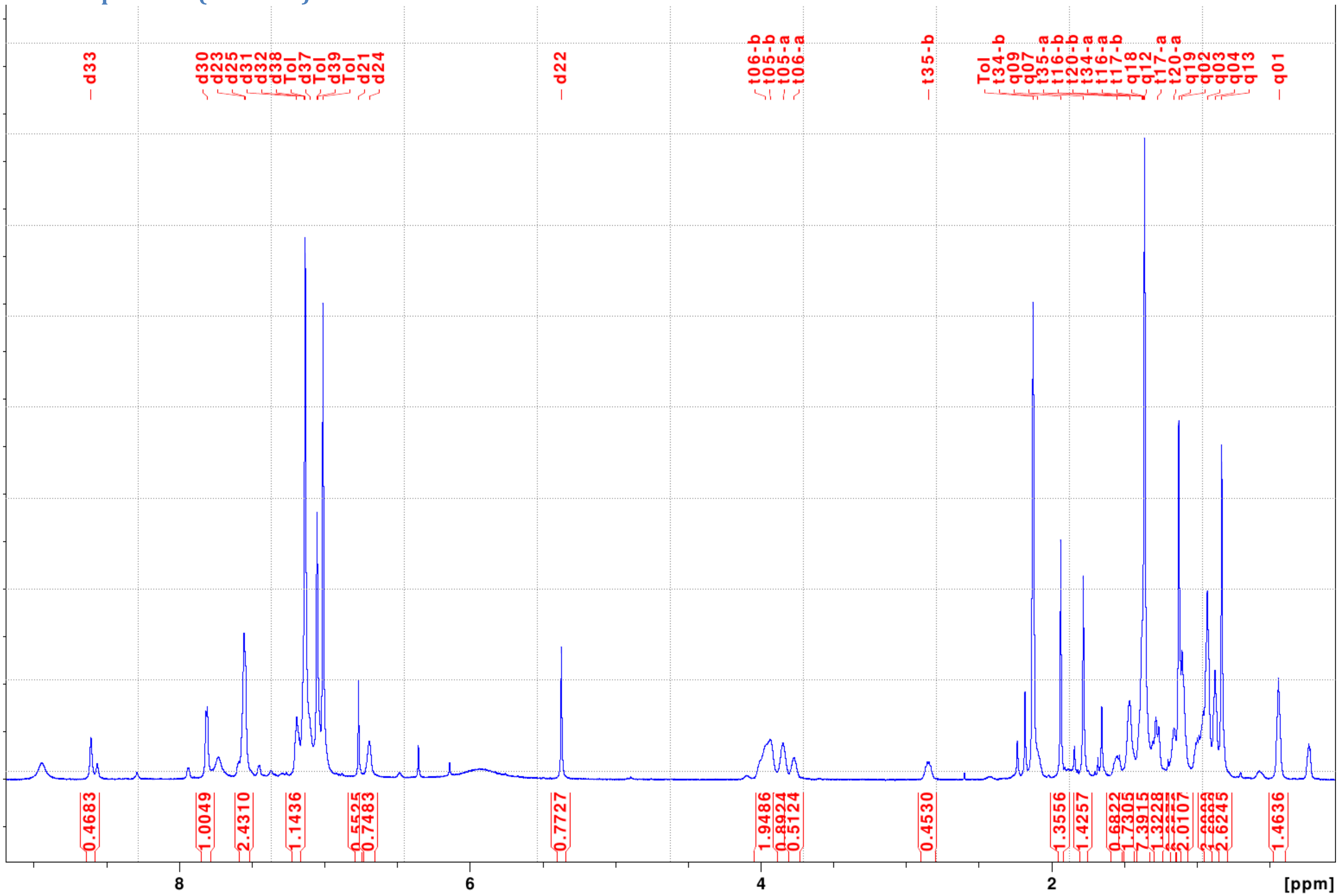

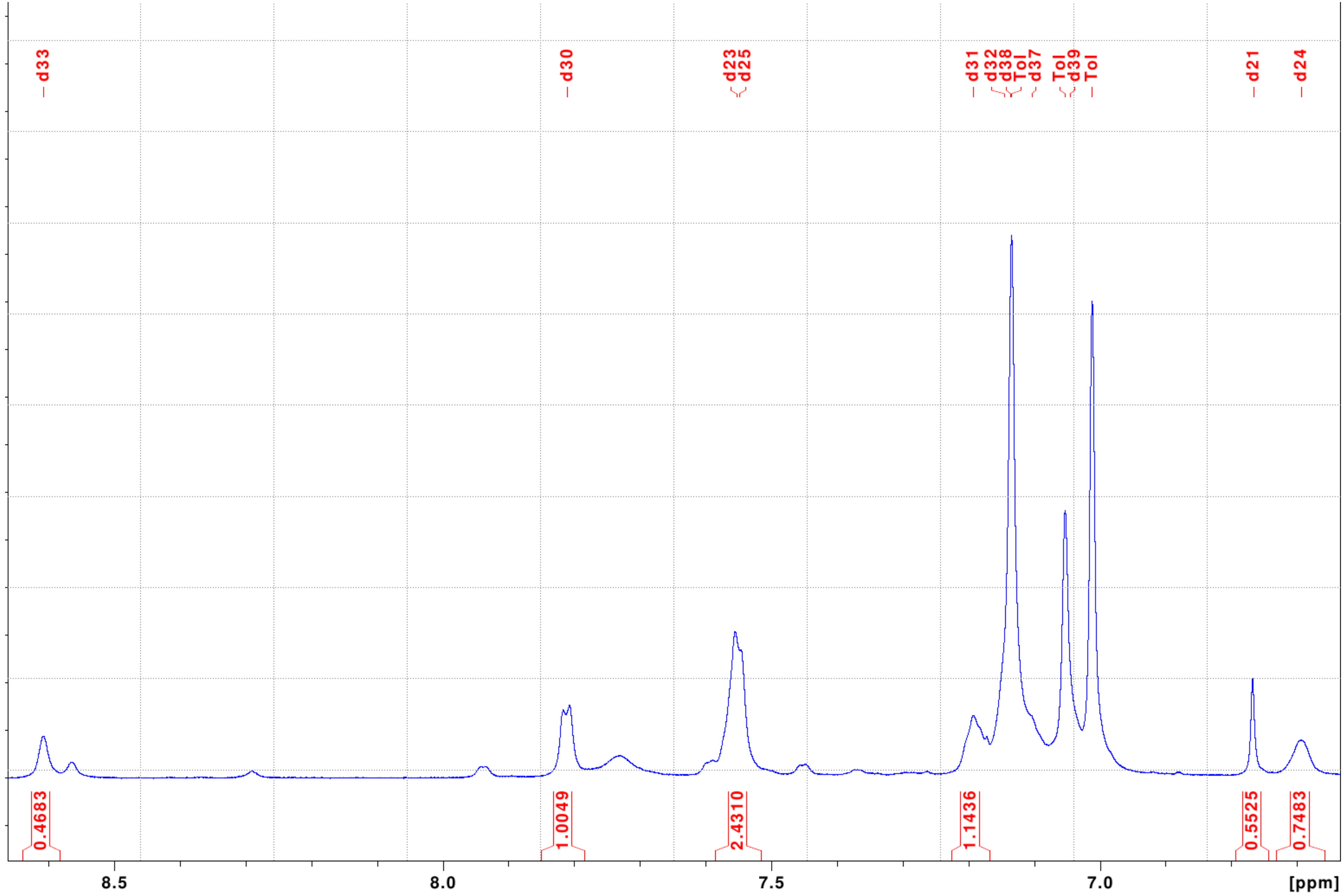
d33
d30
d23
d25
d31
d32
d38
Tol
d37
Tol
d39
Tol
d21
d24
0.4683
1.0049
2.4310
1.1436
0.5525
0.7483
8.5
8.0
7.5
7.0
[ppm]

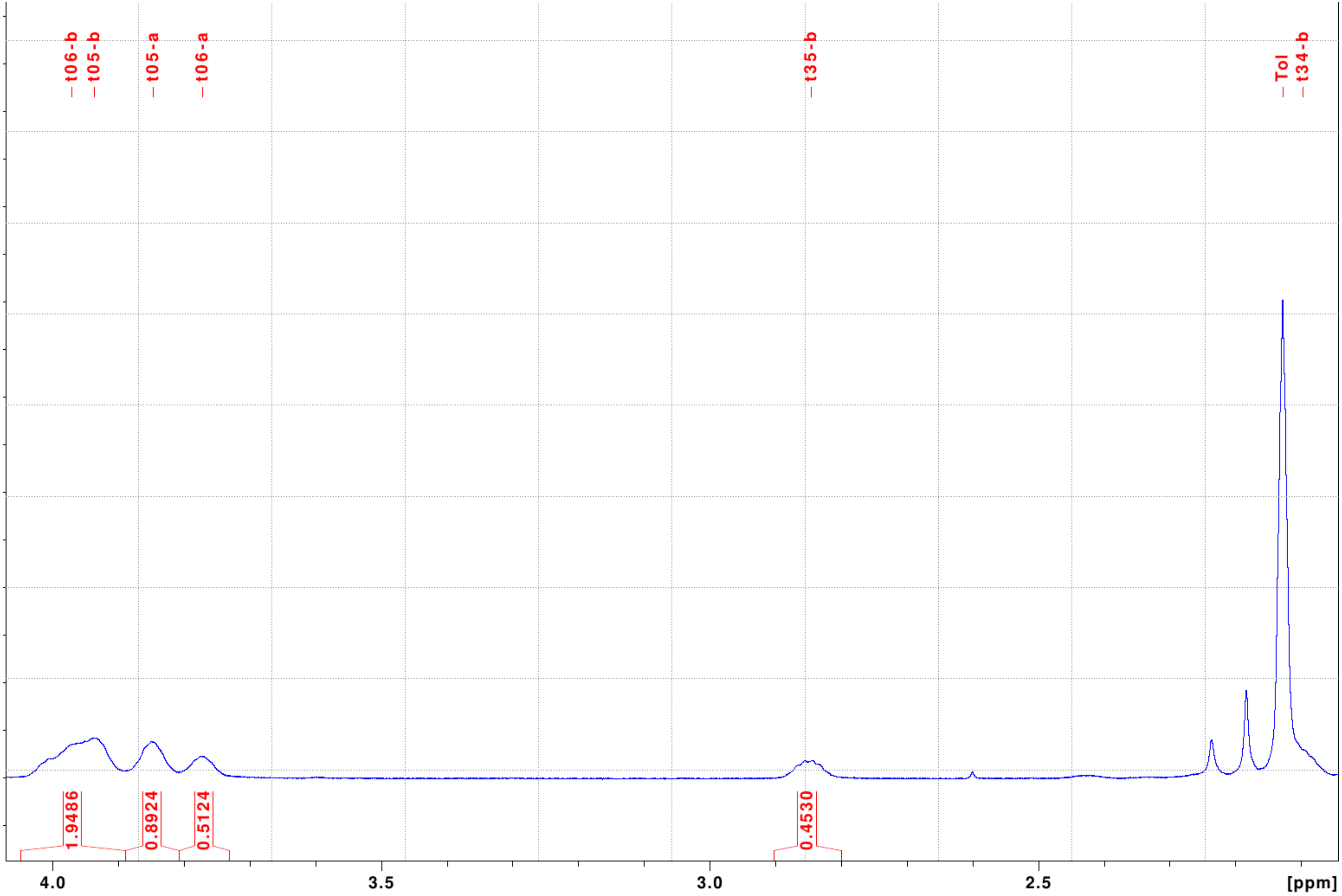
t06-b
t05-b
t05-a
t06-a
t35-b
Tol
t34-b
1.9486
0.8924
0.5124
0.4530
4.0
3.5
3.0
2.5
[ppm]

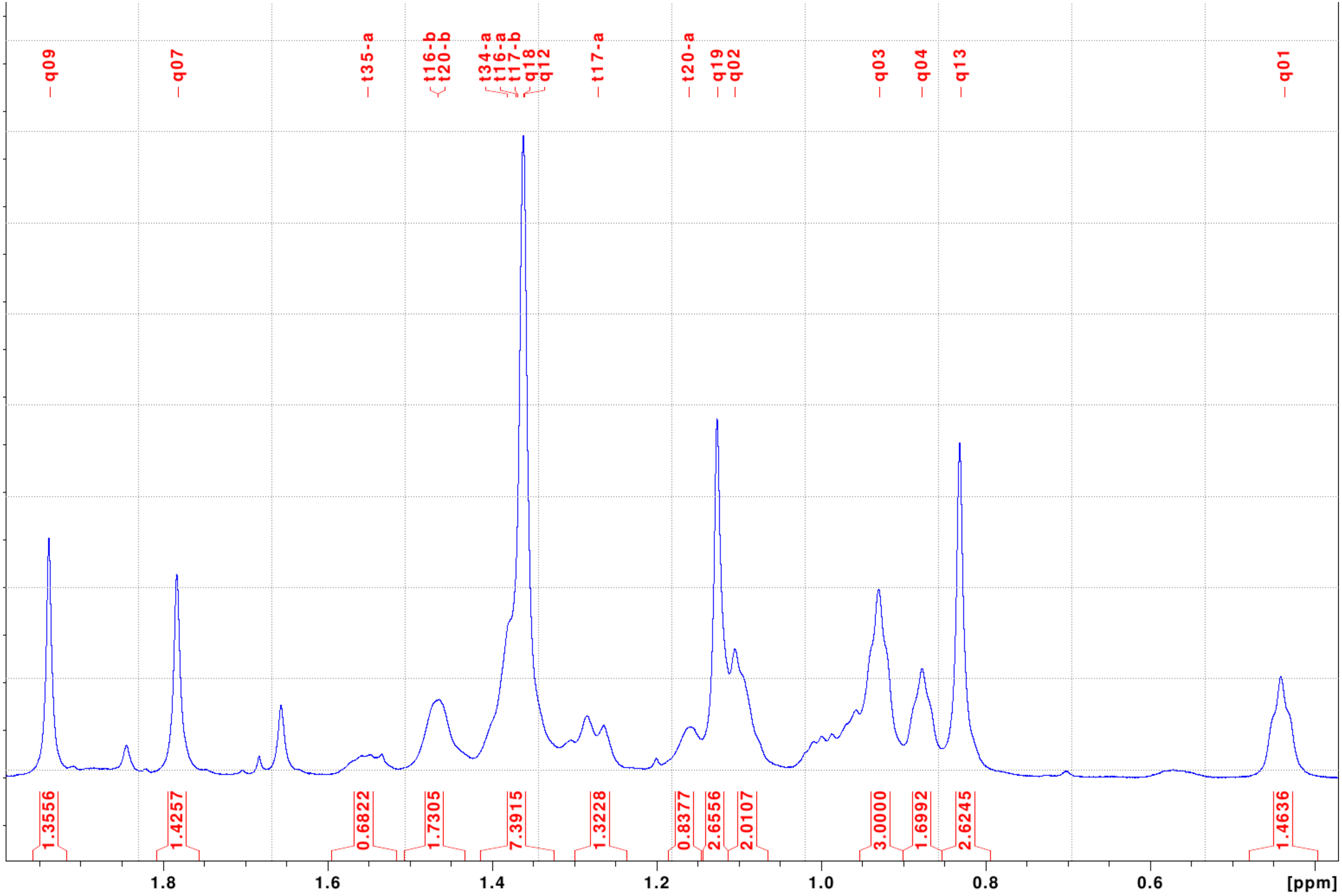
q09
q07
t35-a
t16-b
t20-b
t34-a
t16-a
t17-b
q18
q12
t17-a
t20-a
q19
q02
q03
q04
q13
q01
1.3556
1.4257
0.6822
1.7305
7.3915
1.3228
0.8377
2.6556
2.0107
3.0000
1.6992
2.6245
1.4636
1.8
1.6
1.4
1.2
1.0
0.8
0.6
[ppm]

Structures and NMR signal assignments for products in the reaction mixture 2RS/SR +PhSH

in toluene-d_8 at 25 °C

Signal assignments

Some peak labels in NMR spectra could not be assigned to structures because of low product content.

Structure A: PhSH
D ~ 1.90e-9 (V ~ 1.0)

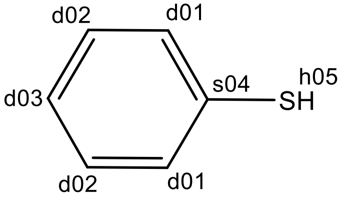

Experiment Bruker_500, 1D 13C: 37 peaks
d01 129.2
d02 128.8
d03 125.2
s04 131.2

Experiment Bruker_493, 1D 1H: 30 peaks
d01-H 6.98
d02-H 6.92
d03-H 6.87
h05-H 3.09

Experiment Bruker_496, 2D 13C-1H via onebond (HSQC): 29 peaks
d01-H - d01
d02-H - d02
d03-H - d03

Experiment Bruker_498, 2D 1H-13C via onebond (H-C correlation): 26 peaks
d01 - d01-H
d02 - d02-H
d03 - d03-H

Experiment Bruker_494, 2D 1H-1H via Jcoupling (COSY): 48 peaks
d01-H - d02-H
d02-H - d01-H d03-H
d03-H - d02-H

Experiment Bruker_497, 2D 13C-1H via Jcoupling (HMBC): 62 peaks
d01-H - d01 d03
d02-H - d02 s04
d03-H - d01

Experiment Bruker_495, 2D 1H-1H via through-space (NOESY): 19 peaks
d01-H - h05-H
h05-H - d01-H

Structure B: Ph-S-S-Ph
D ~ 1.24e-9 (V ~ 3.6)

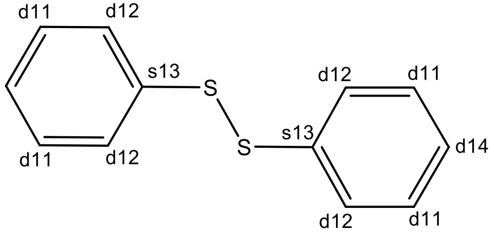

Experiment Bruker_500, 1D 13C: 37 peaks
d11 129.0
d12 127.4
s13 137.2
d14 126.9

Experiment Bruker_493, 1D 1H: 30 peaks
d11-H 6.96
d12-H 7.40
d14-H 6.90

Experiment Bruker_496, 2D 13C-1H via onebond (HSQC): 29 peaks
d11-H - d11
d12-H - d12
d14-H - d14

Experiment Bruker_498, 2D 1H-13C via onebond (H-C correlation): 26 peaks
d11 - d11-H
d12 - d12-H
d14 - d14-H

Experiment Bruker_494, 2D 1H-1H via Jcoupling (COSY): 48 peaks
d11-H - d12-H d14-H
d12-H - d11-H d14-H(weak)
d14-H - d11-H d12-H(weak)

Experiment Bruker_497, 2D 13C-1H via Jcoupling (HMBC): 62 peaks
d11-H - d11 d12(weak) s13
d12-H - d12 s13(weak)
d14-H - d12

Structure C: amine
D ~ 1.04e-9 (V ~ 6.1)

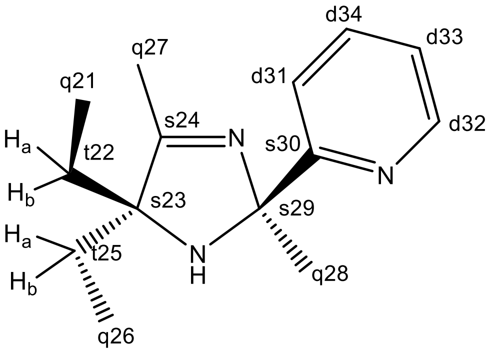

Experiment Bruker_500, 1D 13C: 37 peaks
q21 8.3
t22 30.2
s23 77.0
s24 173.5
t25 31.7
q26 8.9
q27 14.7
q28 33.5
s29 91.9
s30 165.8
d31 121.0
d32 147.8
d33 121.5
d34 135.9

Experiment Bruker_493, 1D 1H: 30 peaks
q21-H 0.42
t22-a 1.16
t22-b 1.31
t25-a 1.36
t25-b 1.48
q26-H 1.02
q27-H 1.71
q28-H 1.81
d31-H 7.73
d32-H 8.38
d33-H 6.67
d34-H 7.17

Experiment Bruker_496, 2D 13C-1H via onebond (HSQC): 29 peaks
d31-H - d31(164 Hz)
d32-H - d32(178 Hz)
d33-H - d33(163 Hz)
d34-H - d34(162 Hz)
q21-H - q21(125 Hz)
q26-H - q26(125 Hz)
q27-H - q27(127 Hz)
q28-H - q28(127 Hz)

t22-a - t22
t22-b - t22
t25-a - t25
t25-b - t25

Experiment Bruker_498, 2D 1H-13C via onebond (H-C correlation): 26 peaks
d31 - d31-H
d32 - d32-H
d33 - d33-H
d34 - d34-H
q21 - q21-H
q26 - q26-H
q27 - q27-H
q28 - q28-H
t22 - t22-a t22-b
t25 - t25-a t25-b

Experiment Bruker_494, 2D 1H-1H via Jcoupling (COSY): 48 peaks
d31-H - d32-H(weak) d33-H(weak) d34-H
d32-H - d31-H(weak) d33-H d34-H(weak)
d33-H - d31-H(weak) d32-H d34-H
d34-H - d31-H d32-H(weak) d33-H
q21-H - t22-a t22-b
q26-H - t25-a t25-b
t22-a - q21-H t22-b
t22-b - q21-H t22-a
t25-a - q26-H t25-b
t25-b - q26-H t25-a

Experiment Bruker_497, 2D 13C-1H via Jcoupling (HMBC): 62 peaks
d31-H - d33
d32-H - d33 d34 s30
d33-H - d31 d32
d34-H - d32 s30
q21-H - s23 t22
q26-H - s23 t25
q27-H - s23 s24 s29(weak) s30(weak)
q28-H - s29 s30
t22-a - q21 s23 t25
t22-b - q21 s23 s24
t25-a - q26 s23 t22
t25-b - q26 s23 s24 t22

Experiment Bruker_495, 2D 1H-1H via through-space (NOESY): 19 peaks
d31-H - q28-H?
q21-H - q27-H t25-b
q26-H - q27-H q28-H
q27-H - q21-H q26-H t22-a t25-a
q28-H - q26-H
t22-a - q27-H
t25-a - q27-H
t25-b - q21-H

Structure D: alkane
D ~ 1.40e-9 (V ~ 2.5)

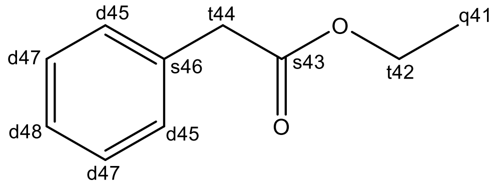

Experiment Bruker_500, 1D 13C: 37 peaks
q41   13.9
t42   60.2
s43 170.5
t44   41.2
d45 129.2
s46 134.5
d47 128.4
d48 126.8

Experiment Bruker_493, 1D 1H: 30 peaks
q41-H   0.95
t42-a   3.91
t42-b   3.91
t44-a   3.37
t44-b   3.37
d45-H   7.17
d47-H   7.12
d48-H   7.05

Experiment Bruker_496, 2D 13C-1H via onebond (HSQC): 29 peaks
d45-H - d45
d47-H - d47
d48-H - d48
q41-H - q41(128 Hz)
t42-a - t42(147 Hz)
t42-b - t42(147 Hz)
t44-a - t44(129 Hz)
t44-b - t44(129 Hz)
Experiment Bruker_498, 2D 1H-13C via onebond (H-C correlation): 26 peaks
d45 - d45-H
d47 - d47-H
d48 - d48-H
q41 - q41-H
t42 - t42-a t42-b
t44 - t44-a t44-b

Experiment Bruker_494, 2D 1H-1H via Jcoupling (COSY): 48 peaks
d45-H - d47-H t44-a(weak) t44-b(weak)
d47-H - d45-H d48-H
d48-H - d47-H
q41-H - t42-a t42-b
t42-a - q41-H
t42-b - q41-H
t44-a - d45-H(weak)
t44-b - d45-H(weak)

Experiment Bruker_497, 2D 13C-1H via Jcoupling (HMBC): 62 peaks
d45-H - d45 d48 t44
d47-H - d47 s46
q41-H - t42
t42-a - q41 s43
t42-b - q41 s43
t44-a - d45 s43 s46
t44-b - d45 s43 s46

Experiment Bruker_495, 2D 1H-1H via through-space (NOESY): 19 peaks
d45-H - t44-a t44-b
t44-a - d45-H
t44-b - d45-H

Structure E: D ~ 0.64e-9 (V ~ 26)

Structure F: D ~ 1.68e-9 (V ~ 1.4)

$^{13}C\{^{1}H\}$ NMR spectrum (150 MHz)

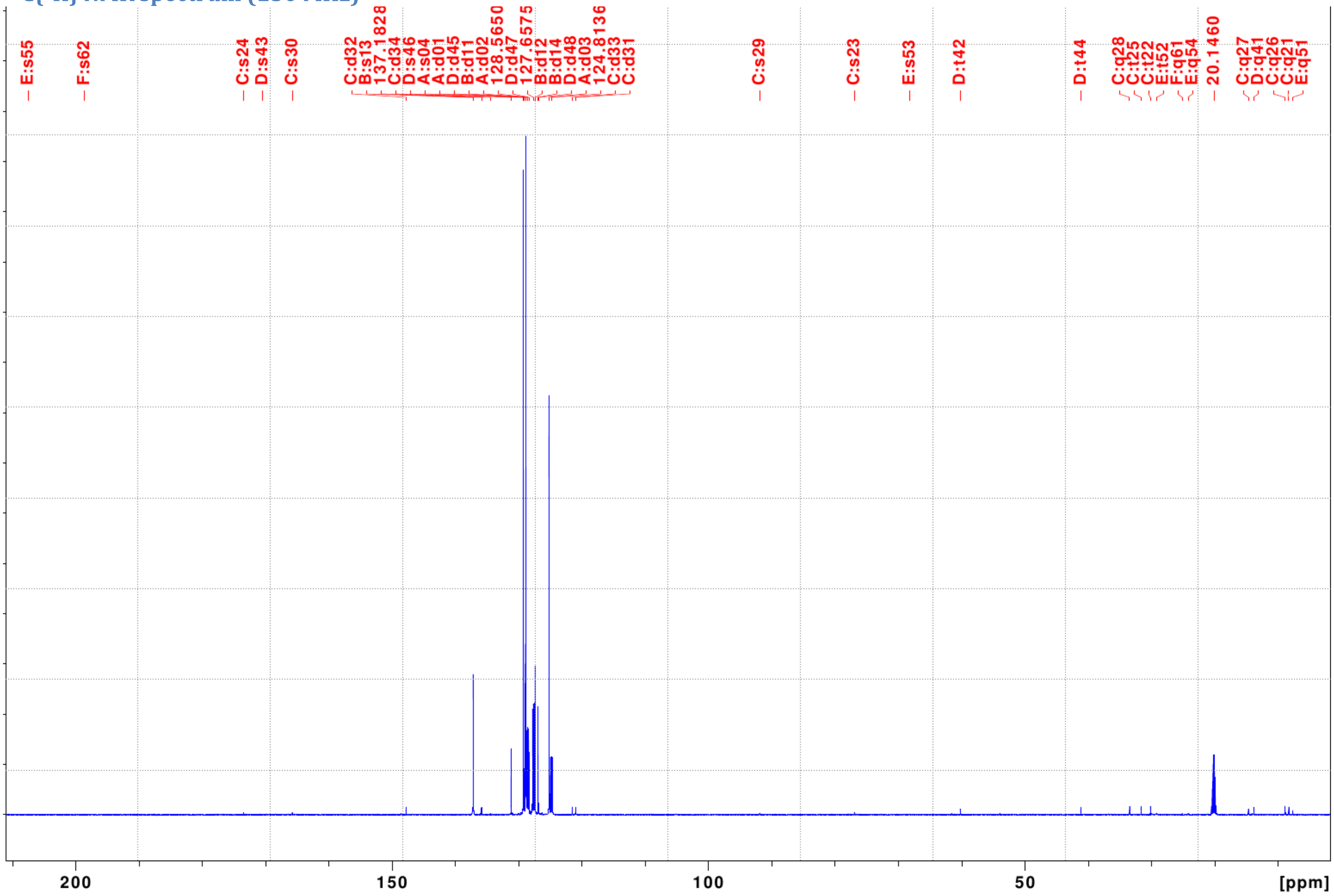

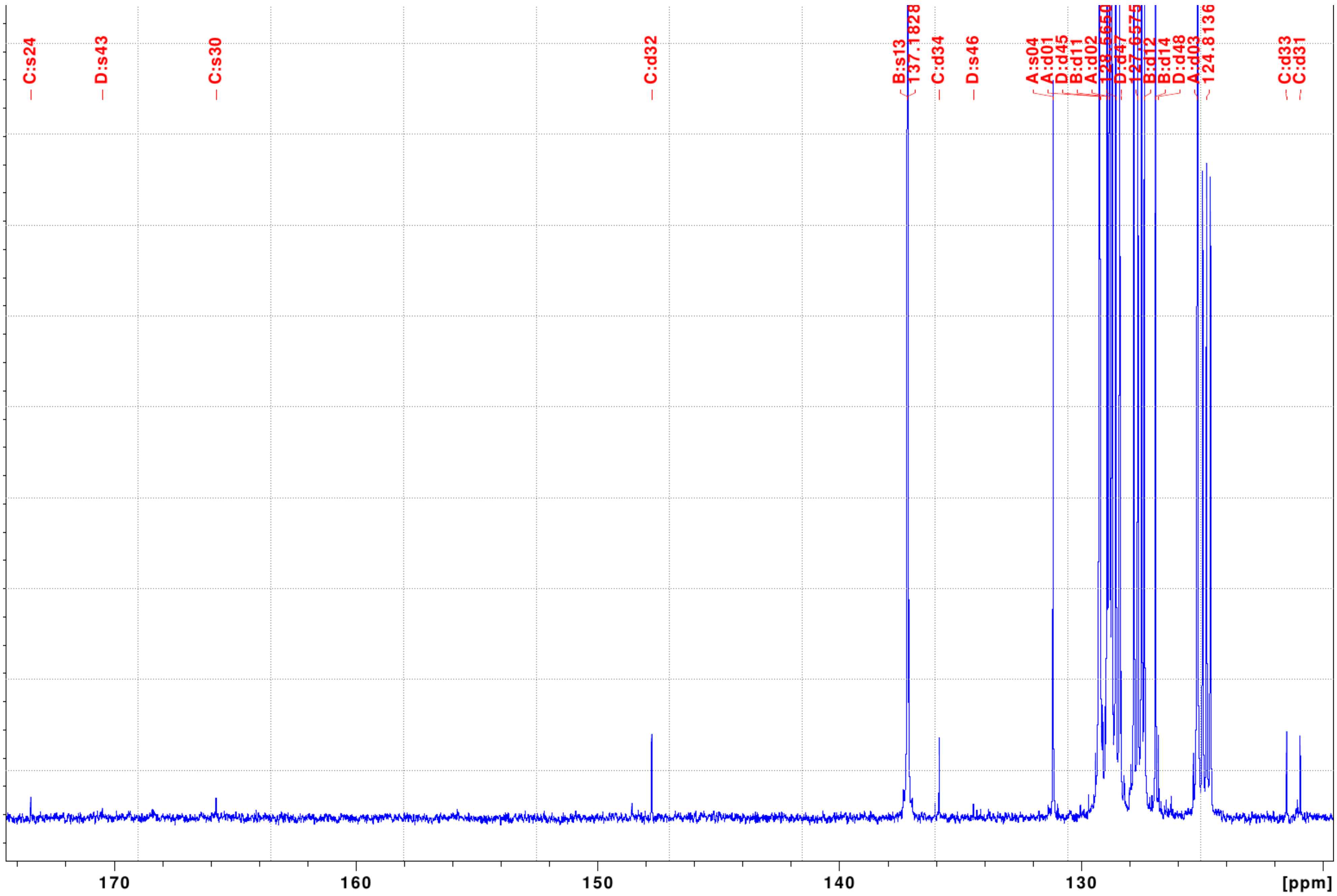
C:s24
D:s43
C:s30
C:d32
B:s13
137.1828
C:d34
D:s46
A:s04
A:d01
D:d45
B:d11
A:d02
128.5650
D:d47
127.6575
B:d12
B:d14
D:d48
A:d03
124.8136
C:d33
C:d31
170
160
150
140
130
[ppm]

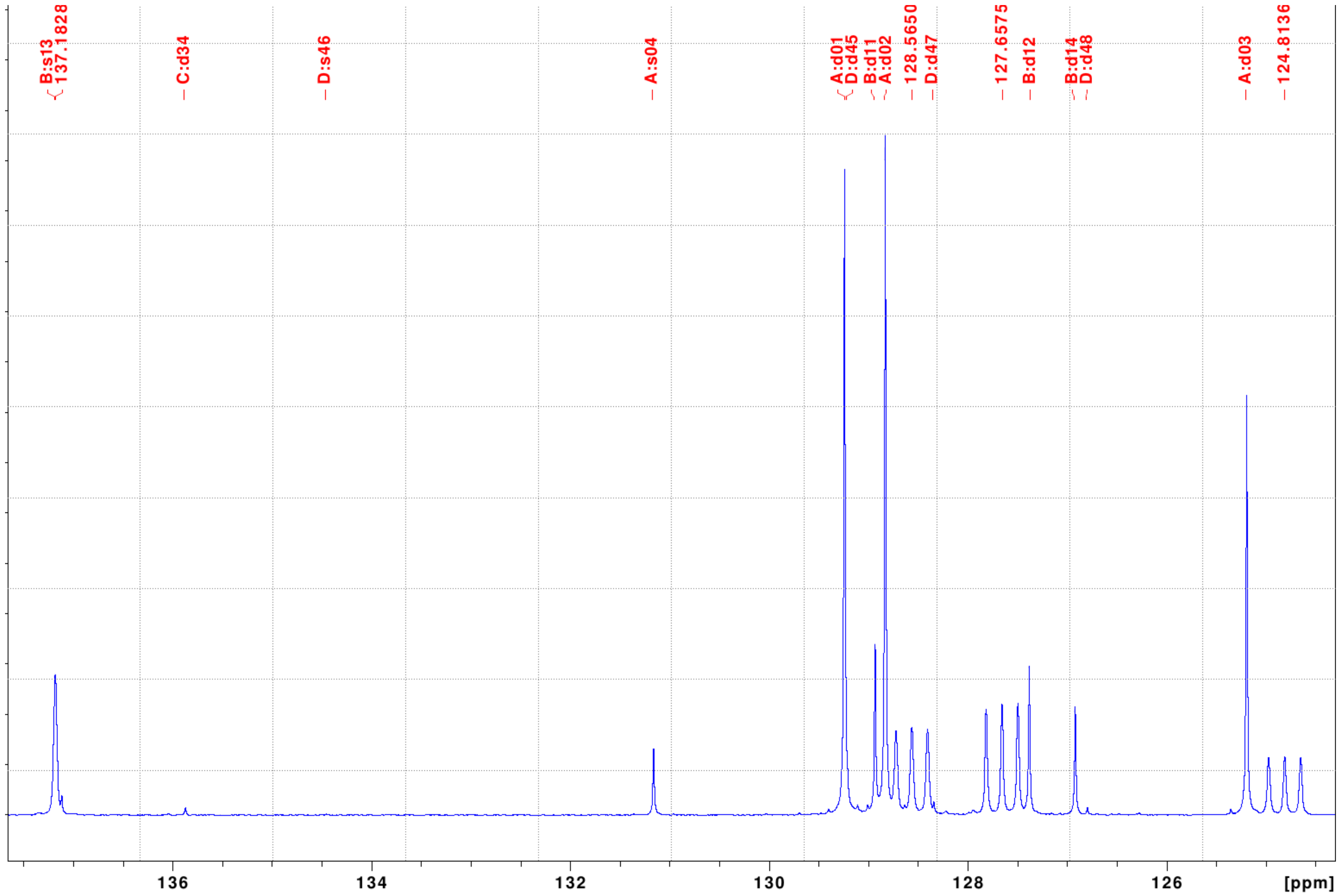
B:s13
137.1828
C:d34
D:s46
A:s04
A:d01
D:d45
B:d11
A:d02
128.5650
D:d47
127.6575
B:d12
B:d14
D:d48
A:d03
124.8136
136
134
132
130
128
126
[ppm]

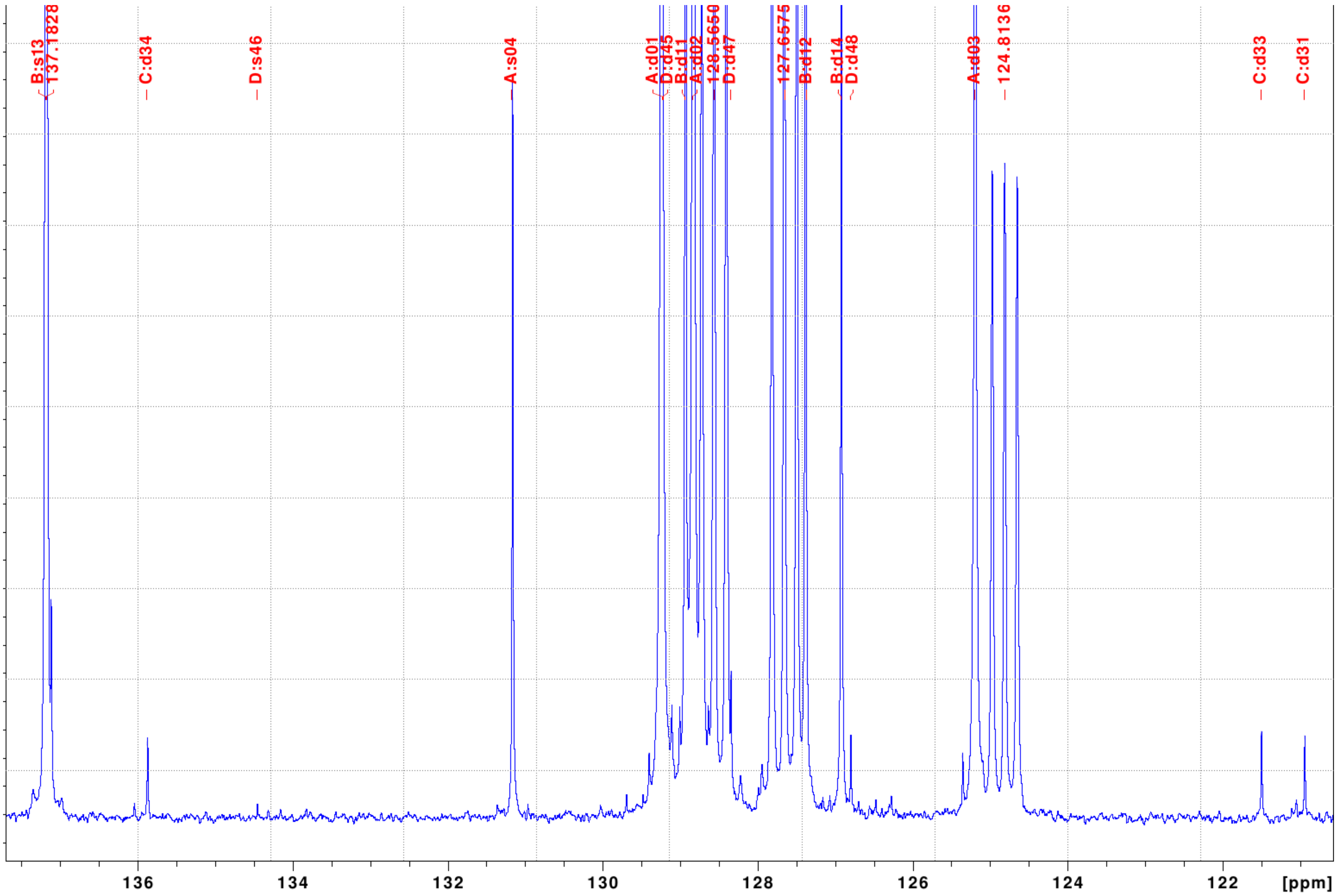
B:s13
137.1828
C:d34
D:s46
A:s04
A:d01
D:d45
B:d11
A:d02
128.5650
D:d47
127.6575
B:d12
B:d14
D:d48
A:d03
124.8136
C:d33
C:d31
136
134
132
130
128
126
124
122
[ppm]

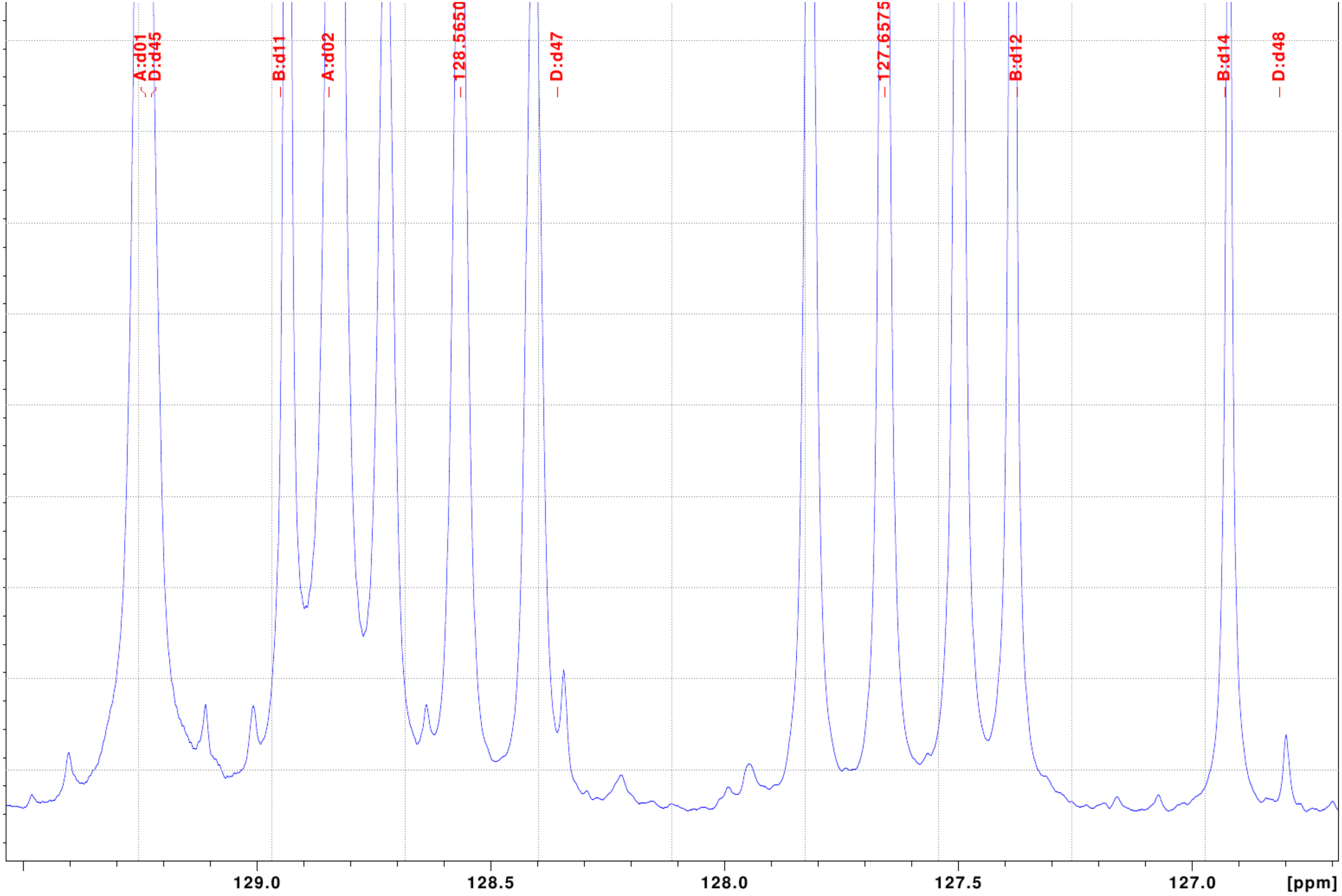
A:d01
D:d45
B:d11
A:d02
128.5650
D:d47
127.6575
B:d12
B:d14
D:d48
129.0
128.5
128.0
127.5
127.0
[ppm]

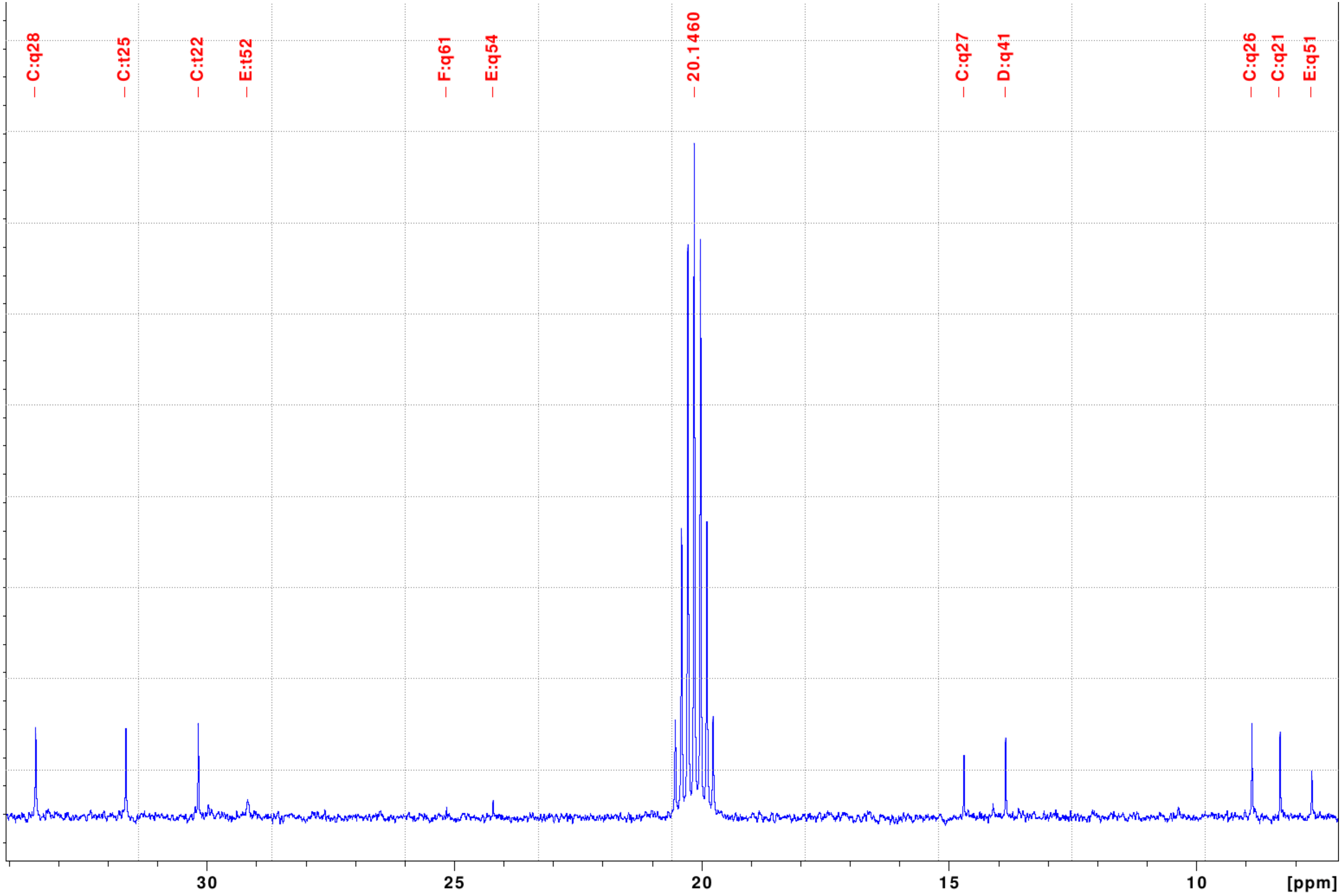

C:q28
C:t25
C:t22
E:t52
F:q61
E:q54
20.1460
C:q27
D:q41
C:q26
C:q21
E:q51
30
25
20
15
10
[ppm]

^{1}H NMR spectrum (600 MHz)

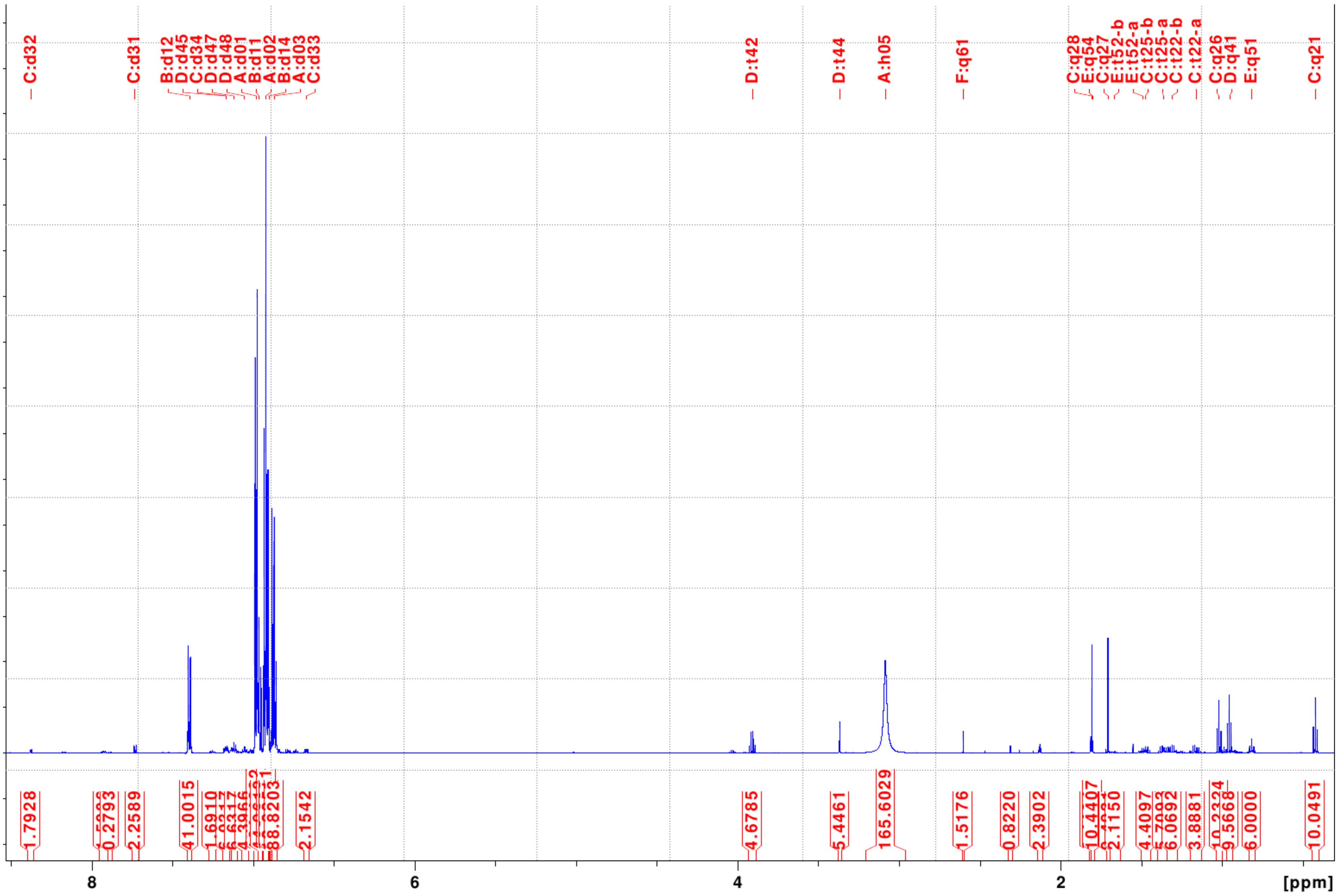

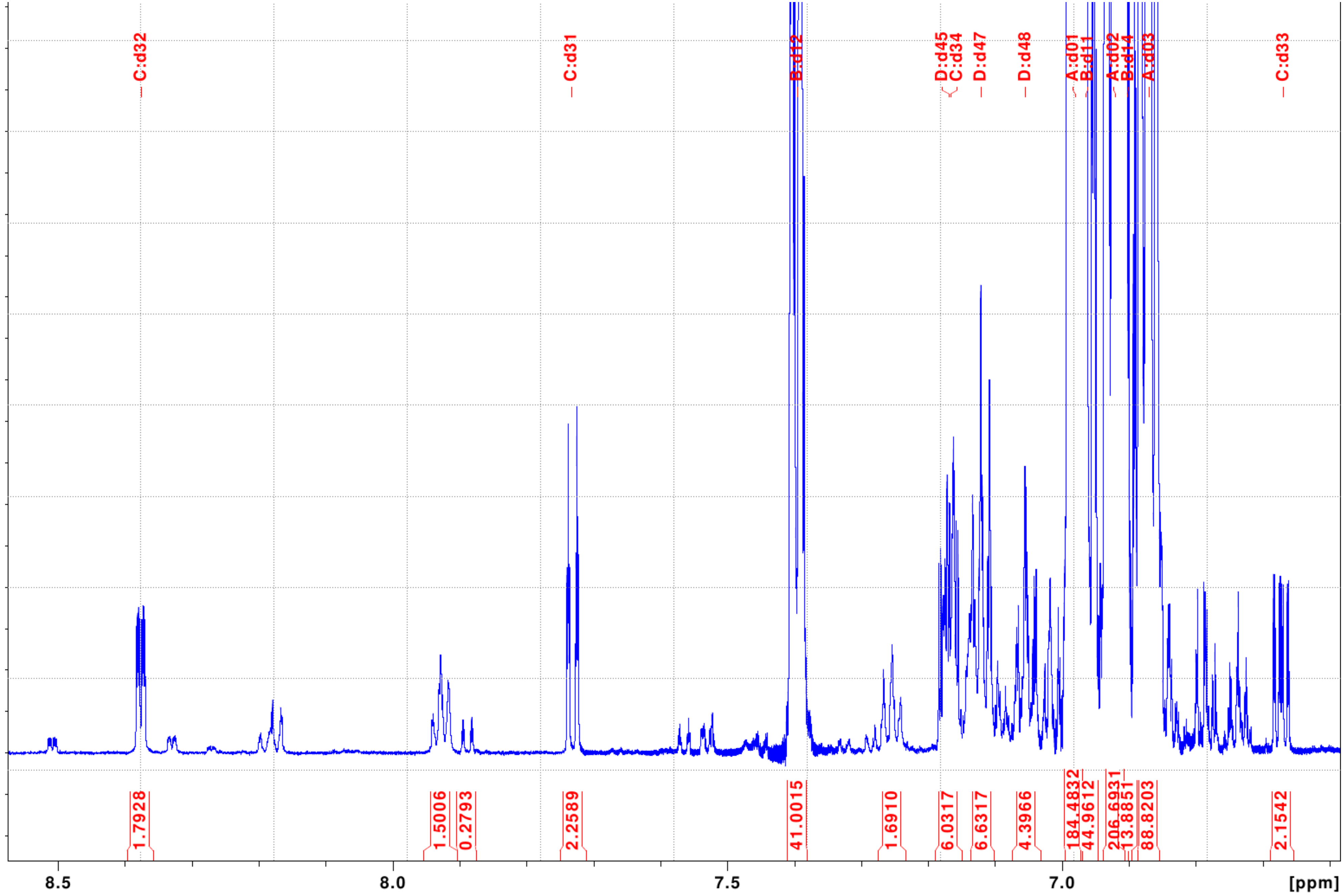
C:d32
C:d31
B:d12
D:d45
C:d34
D:d47
D:d48
A:d01
B:d11
A:d02
B:d14
A:d03
C:d33
1.7928
1.5006
0.2793
2.2589
41.0015
1.6910
6.0317
6.6317
4.3966
184.4832
44.9612
206.6931
13.8851
88.8203
2.1542
8.5
8.0
7.5
7.0
[ppm]

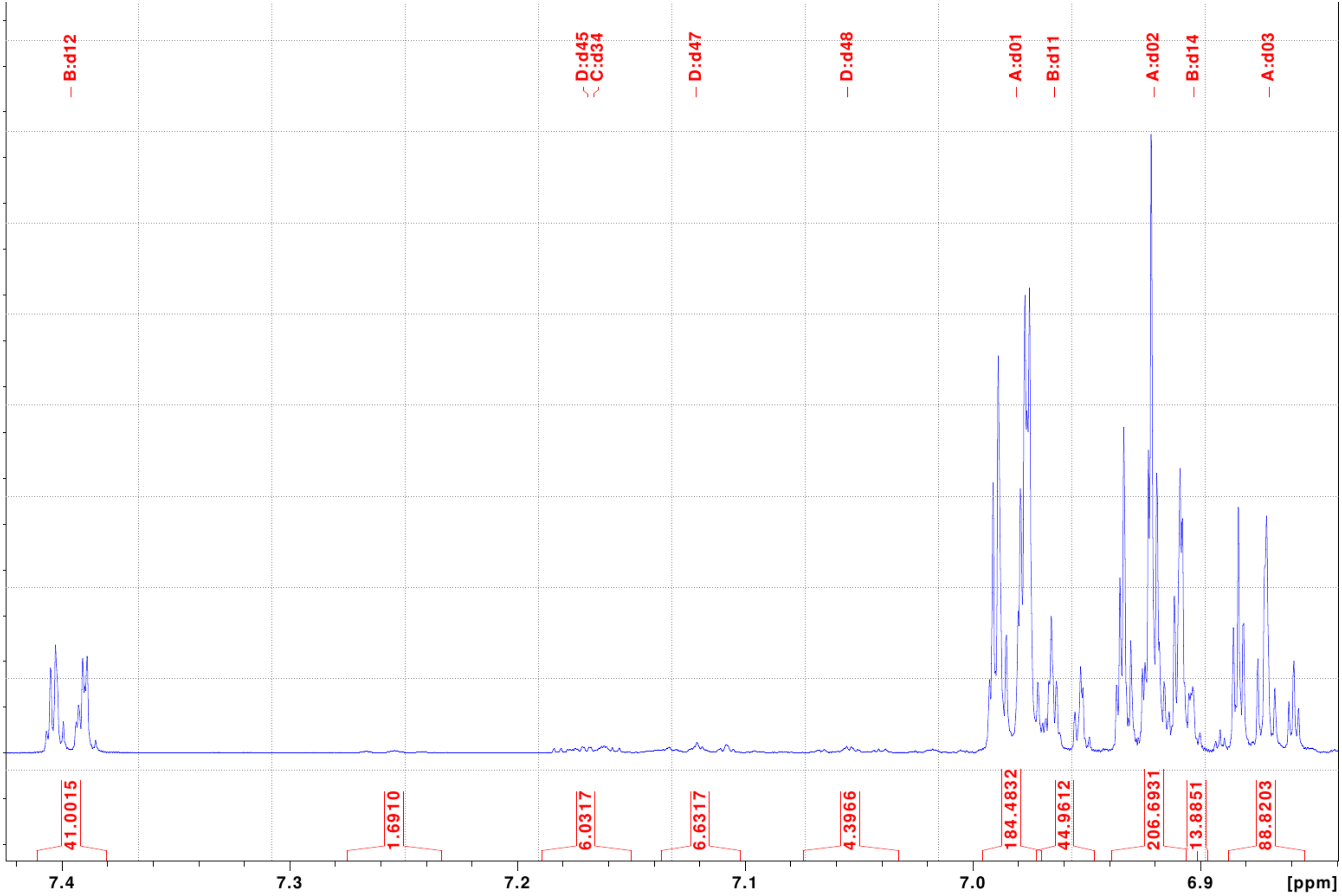
B:d12
D:d45
C:d34
D:d47
D:d48
A:d01
B:d11
A:d02
B:d14
A:d03
41.0015
1.6910
6.0317
6.6317
4.3966
184.4832
44.9612
206.6931
13.8851
88.8203
7.4
7.3
7.2
7.1
7.0
6.9
[ppm]

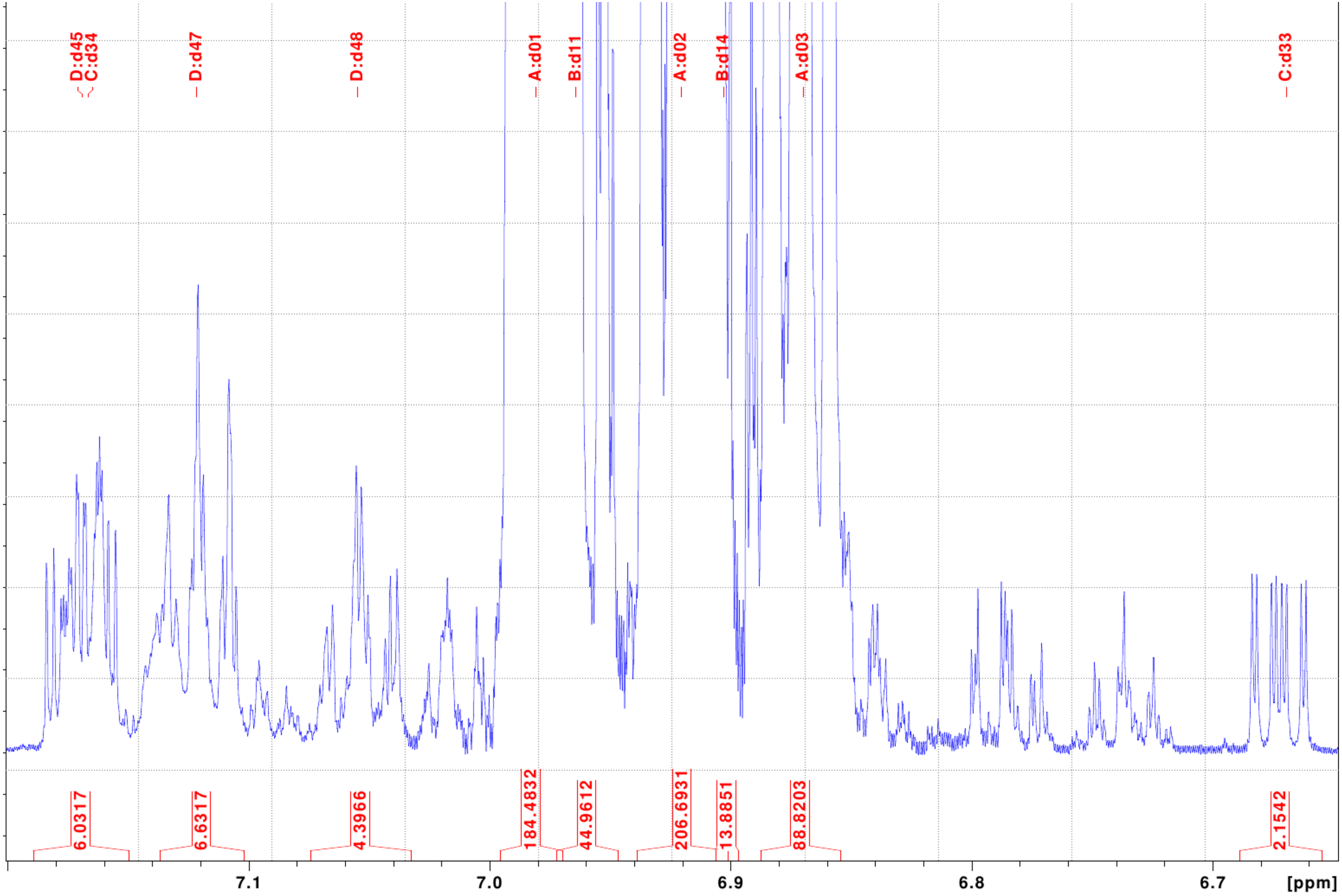

D:d45
C:d34
D:d47
D:d48
A:d01
B:d11
A:d02
B:d14
A:d03
C:d33
6.0317
6.6317
4.3966
184.4832
44.9612
206.6931
13.8851
88.8203
2.1542
7.1
7.0
6.9
6.8
6.7
[ppm]

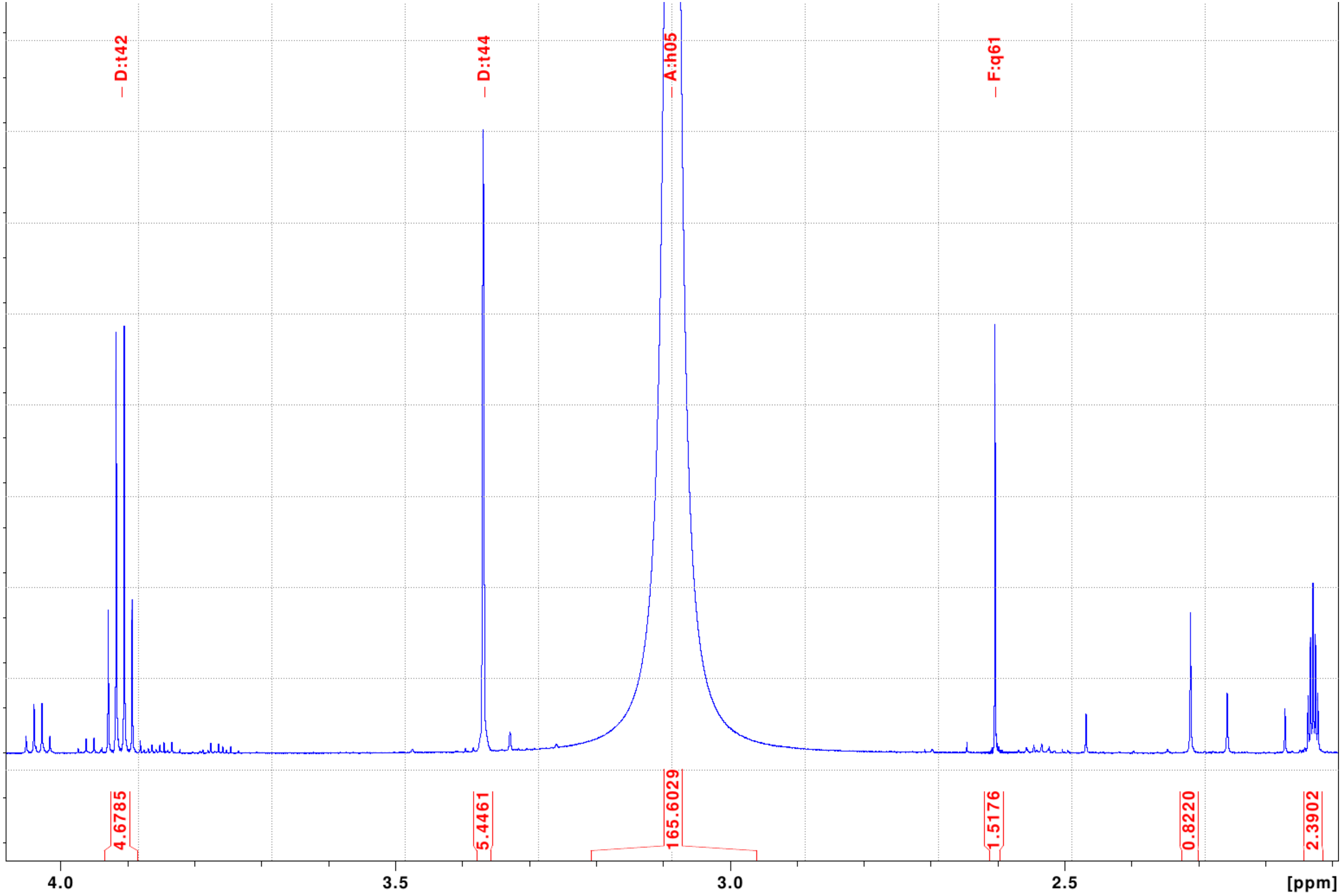
D:t42
D:t44
A:h05
F:q61
4.6785
5.4461
165.6029
1.5176
0.8220
2.3902
4.0
3.5
3.0
2.5
[ppm]

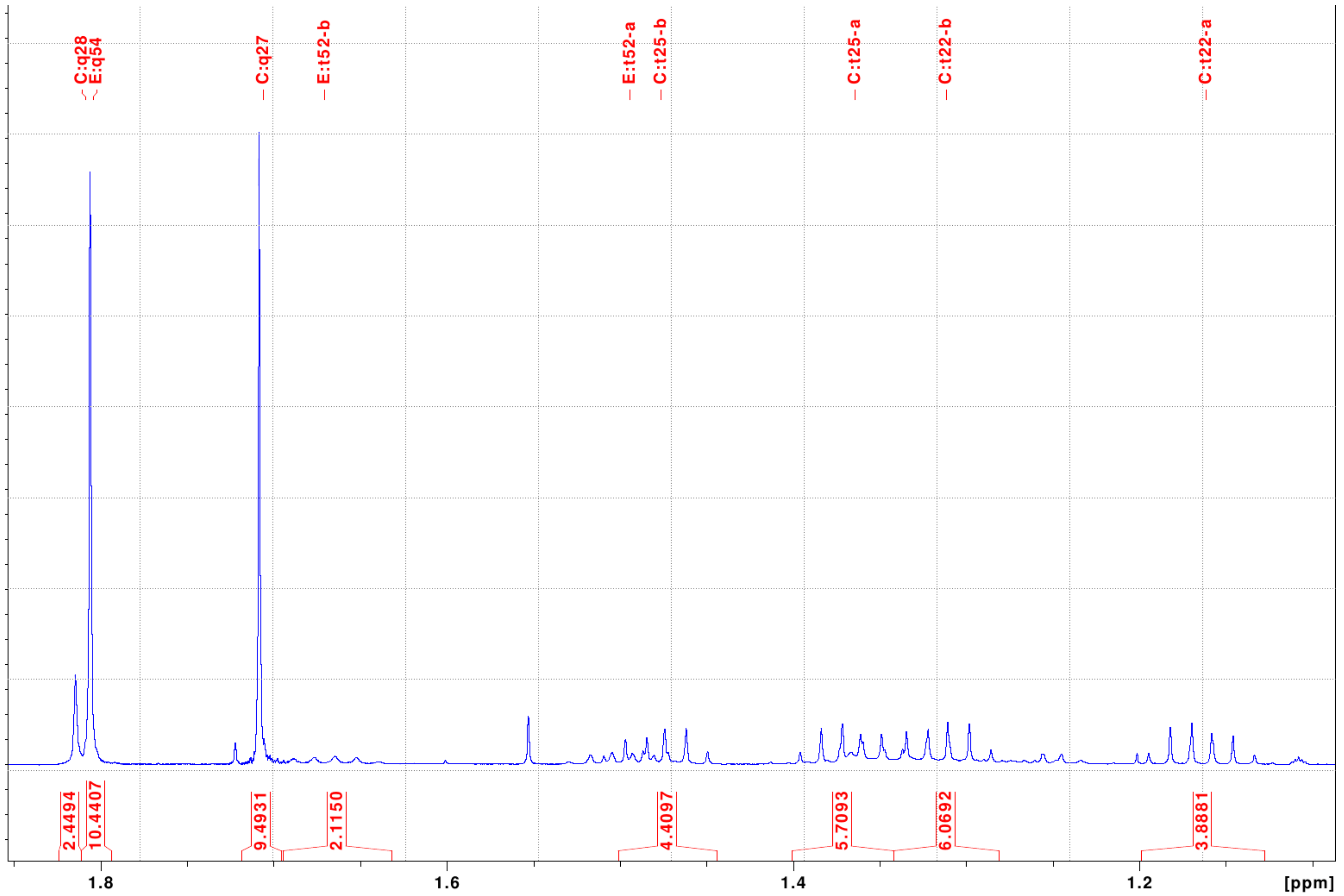

C:q28
E:q54
C:q27
E:t52-b
E:t52-a
C:t25-b
C:t25-a
C:t22-b
C:t22-a
2.4494
10.4407
9.4931
2.1150
4.4097
5.7093
6.0692
3.8881
1.8
1.6
1.4
1.2
[ppm]

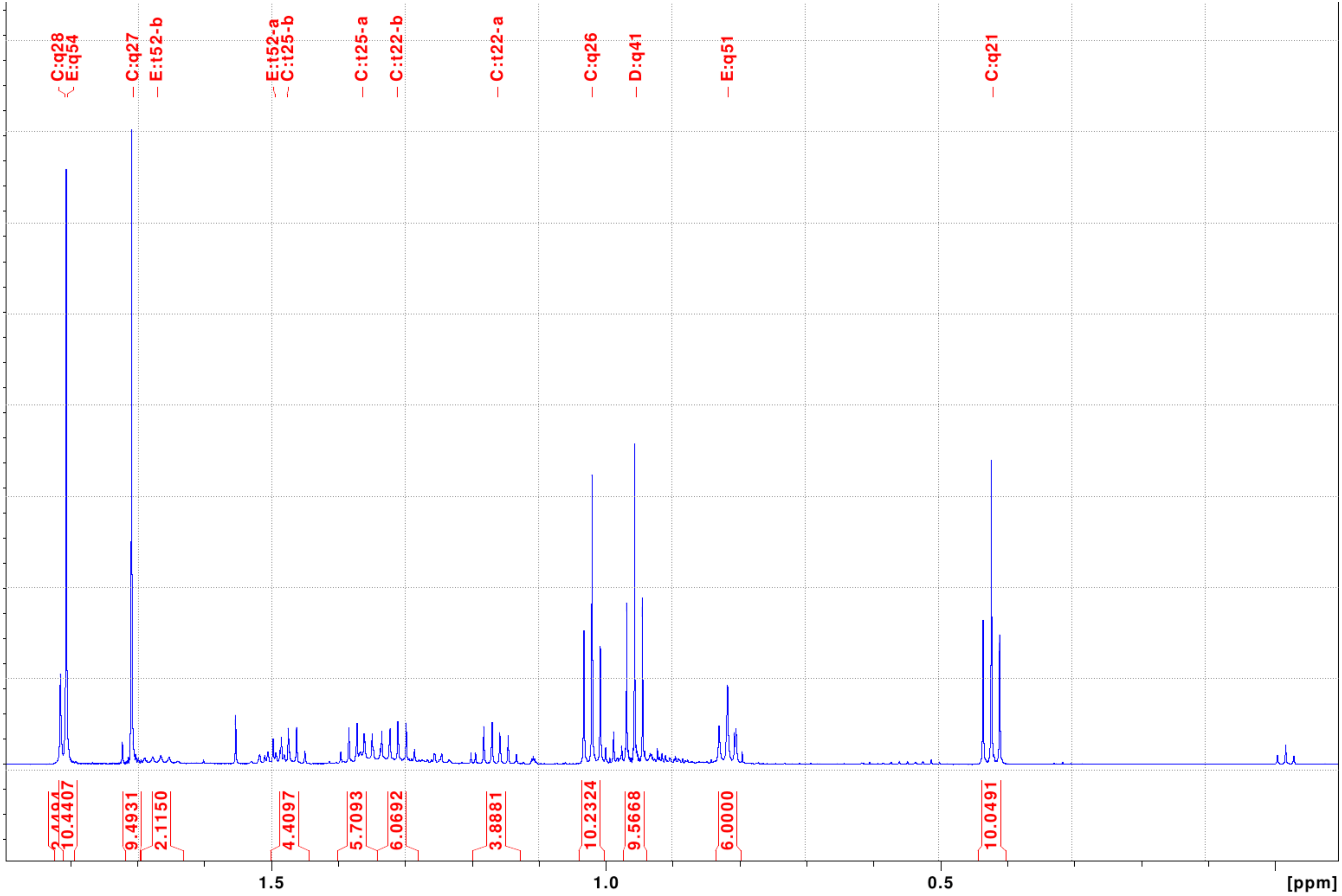

C:q28
E:q54
C:q27
E:t52-b
E:t52-a
C:t25-b
C:t25-a
C:t22-b
C:t22-a
C:q26
D:q41
E:q51
C:q21
10.4407
9.4931
2.1150
4.4097
5.7093
6.0692
3.8881
10.2324
9.5668
6.0000
10.0491
1.5
1.0
0.5
[ppm]

Structures and NMR signal assignments for products in the reaction mixture 3 + PhSH

in toluene-d_8 at 25 °C

Signal assignments

Some peak labels in NMR spectra could not be assigned to structures because of low product content.

Structure A: PhSH
D ~ 1.91e-9 (V ~ 1)

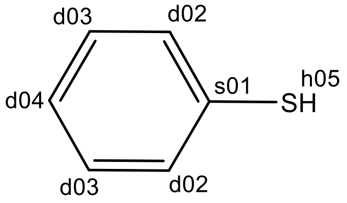

Experiment Bruker_323, 1D 13C: 33 peaks
s01 131.2
d02 129.2
d03 128.8
d04 125.2

Experiment Bruker_333, 1D 1H: 27 peaks
d02-H 6.98
d03-H 6.92
d04-H 6.87
h05-H 3.08

Experiment Bruker_326, 2D 13C-1H via onebond (HSQC): 26 peaks
d02-H - d02
d03-H - d03
d04-H - d04

Experiment Bruker_328, 2D 1H-13C via onebond (H-C correlation): 22 peaks
d02 - d02-H
d03 - d03-H
d04 - d04-H

Experiment Bruker_324, 2D 1H-1H via Jcoupling (COSY): 39 peaks
d02-H - d03-H
d03-H - d02-H d04-H
d04-H - d03-H

Experiment Bruker_327, 2D 13C-1H via Jcoupling (HMBC): 64 peaks
d02-H - d02 d04
d03-H - d03 s01
d04-H - d02
h05-H - d02

Experiment Bruker_325, 2D 1H-1H via through-space (NOESY): 17 peaks
d02-H - h05-H
h05-H - d02-H

Structure B: Ph-S-S-Ph
D ~ 1.25e-9 (V ~ 3.6)

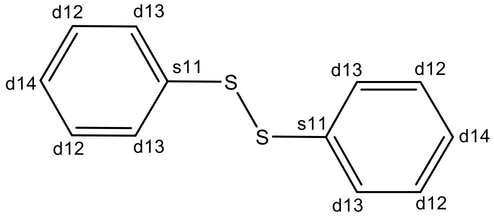

Experiment Bruker_323, 1D 13C: 33 peaks
s11 137.2
d12 128.9
d13 127.4
d14 126.9

Experiment Bruker_333, 1D 1H: 27 peaks
d12-H 6.96
d13-H 7.39
d14-H 6.90

Experiment Bruker_326, 2D 13C-1H via onebond (HSQC): 26 peaks
d12-H - d12
d13-H - d13
d14-H - d14

Experiment Bruker_328, 2D 1H-13C via onebond (H-C correlation): 22 peaks
d12 - d12-H
d13 - d13-H
d14 - d14-H

Experiment Bruker_324, 2D 1H-1H via Jcoupling (COSY): 39 peaks
d12-H - d13-H
d13-H - d12-H
d14-H - d12-H?

Experiment Bruker_327, 2D 13C-1H via Jcoupling (HMBC): 64 peaks
d12-H - d12 d13(weak) s11
d13-H - d13 d14 s11(weak)
d14-H - d13 s11(weak)

Structure C: tBu-isobutyrate
D ~ 1.43e-9 (V ~ 2.4)

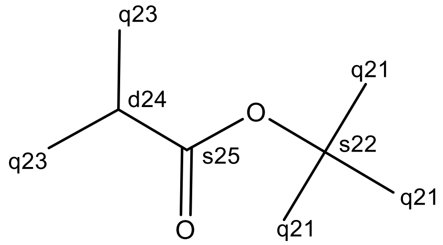

Experiment Bruker_323, 1D 13C: 33 peaks
q21 27.7
s22 78.8
q23 18.9
d24 34.8
s25 175.4

Experiment Bruker_333, 1D 1H: 27 peaks
q21-H 1.39
q23-H 1.08
d24-H 2.34

Experiment Bruker_326, 2D 13C-1H via onebond (HSQC): 26 peaks
d24-H - d24
q21-H - q21(127 Hz)
q23-H - q23(127 Hz)

Experiment Bruker_328, 2D 1H-13C via onebond (H-C correlation): 22 peaks
d24 - d24-H
q21 - q21-H
q23 - q23-H

Experiment Bruker_324, 2D 1H-1H via Jcoupling (COSY): 39 peaks
d24-H - q23-H
q23-H - d24-H

Experiment Bruker_327, 2D 13C-1H via Jcoupling (HMBC): 64 peaks
d24-H - q23 s25
q21-H - q21 s22
q23-H - d24 q23 s25

Structure D: amine
D ~ 1.08e-9 (V ~ 5.5)

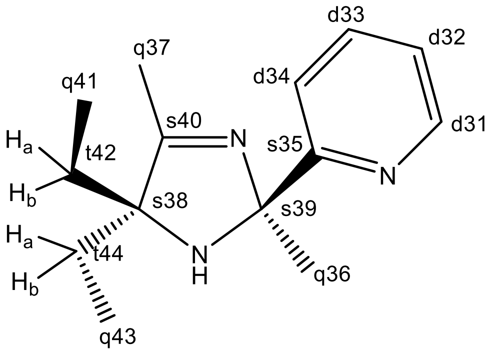

Experiment Bruker_323, 1D 13C: 33 peaks
d31 147.8
d32 121.5
d33 135.9
d34 121.0
s35 165.8
q36   33.5
q37   14.7
s38   77.0
s39   91.9
s40 173.4
q41   8.3
t42   30.2
q43   8.9
t44   31.7

Experiment Bruker_333, 1D 1H: 27 peaks
d31-H   8.37
d32-H   6.67
d33-H   7.16
d34-H   7.73
q36-H   1.81
q37-H   1.70
q41-H   0.42
t42-a   1.16
t42-b   1.31
q43-H   1.02
t44-a   1.37
t44-b   1.47
h45-H   3.08

Experiment Bruker_326, 2D 13C-1H via onebond (HSQC): 26 peaks
d31-H - d31(178 Hz)
d32-H - d32(163 Hz)
d33-H - d33(161 Hz)
d34-H - d34(165 Hz)
q36-H - q36(128 Hz)
q37-H - q37(127 Hz)
q41-H - q41(126 Hz)
q43-H - q43(125 Hz)
t42-a - t42
t42-b - t42
t44-a - t44
t44-b - t44

Experiment Bruker_328, 2D 1H-13C via onebond (H-C correlation): 22 peaks
d31 - d31-H
d32 - d32-H
d33 - d33-H
d34 - d34-H
q36 - q36-H
q37 - q37-H
q41 - q41-H
q43 - q43-H
t42 - t42-a t42-b
t44 - t44-a t44-b

Experiment Bruker_324, 2D 1H-1H via Jcoupling (COSY): 39 peaks
d31-H - d32-H d33-H(weak) d34-H(weak)
d32-H - d31-H d33-H d34-H(weak)
d33-H - d31-H(weak) d32-H d34-H
d34-H - d31-H(weak) d32-H(weak) d33-H
q41-H - t42-a t42-b
q43-H - t44-a t44-b
t42-a - q41-H t42-b
t42-b - q41-H t42-a
t44-a - q43-H t44-b
t44-b - q43-H t44-a

Experiment Bruker_327, 2D 13C-1H via Jcoupling (HMBC): 64 peaks
d31-H - d32 d33 s35
d32-H - d31 d34
d33-H - d31 s35
d34-H - d32 s35(weak)
q36-H - s35 s39
q37-H - s35(weak) s38 s39(weak) s40
q41-H - s38 t42
q43-H - s38 t44
t42-a - q41 s38 t44
t42-b - q41 s38 s40 t44(weak)
t44-a - q43 s38 t42
t44-b - q43 s38 s40 t42(weak)

Experiment Bruker_325, 2D 1H-1H via through-space (NOESY): 17 peaks
d34-H - q36-H?
h45-H - q36-H q41-H? q43-H? t44-a?
q36-H - h45-H q43-H t44-a?
q37-H - q41-H q43-H t42-a? t44-a?
q41-H - q37-H
q43-H - q36-H q37-H

Structures E: undefined

Experiment Bruker_323, 1D 13C: 33 peaks
q51   7.6
t52   30.0
s53   67.4
q54   24.0
s55 208.0
q56   27.7
s57   79.5

Experiment Bruker_333, 1D 1H: 27 peaks
q51-H   0.76
t52-a   1.43
t52-b   1.55
q54-H   1.74
q56-H   1.40

Experiment Bruker_326, 2D 13C-1H via onebond (HSQC): 26 peaks
q51-H - q51
q54-H - q54
q56-H - q56
t52-a - t52
t52-b - t52

Experiment Bruker_328, 2D 1H-13C via onebond (H-C correlation): 22 peaks
q51 - q51-H

Experiment Bruker_324, 2D 1H-1H via Jcoupling (COSY): 39 peaks
q51-H - t52-a t52-b
t52-a - q51-H t52-b
t52-b - q51-H t52-a

Experiment Bruker_327, 2D 13C-1H via Jcoupling (HMBC): 64 peaks
q51-H - s53 t52
q54-H - s53 s55
q56-H - s57
t52-a - q51 s53
t52-b - q51 s53 s55

The system has 2 distinct fragment(s)
Fragment 1:
q51
t52
s53
q54
s55
Fragment 2:
q56
s57

$^{13}C\{^1H\}$ NMR spectrum (150 MHz)

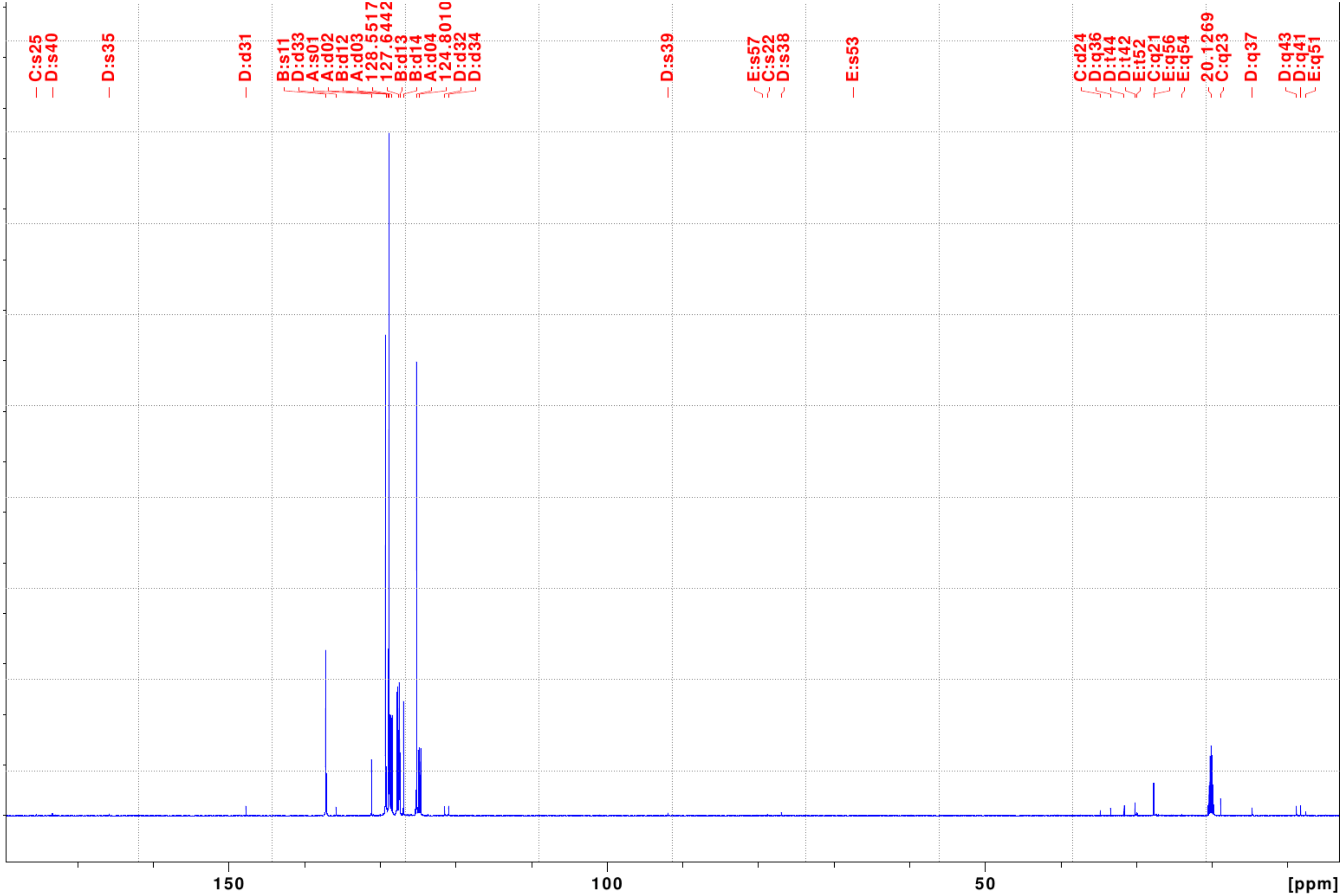

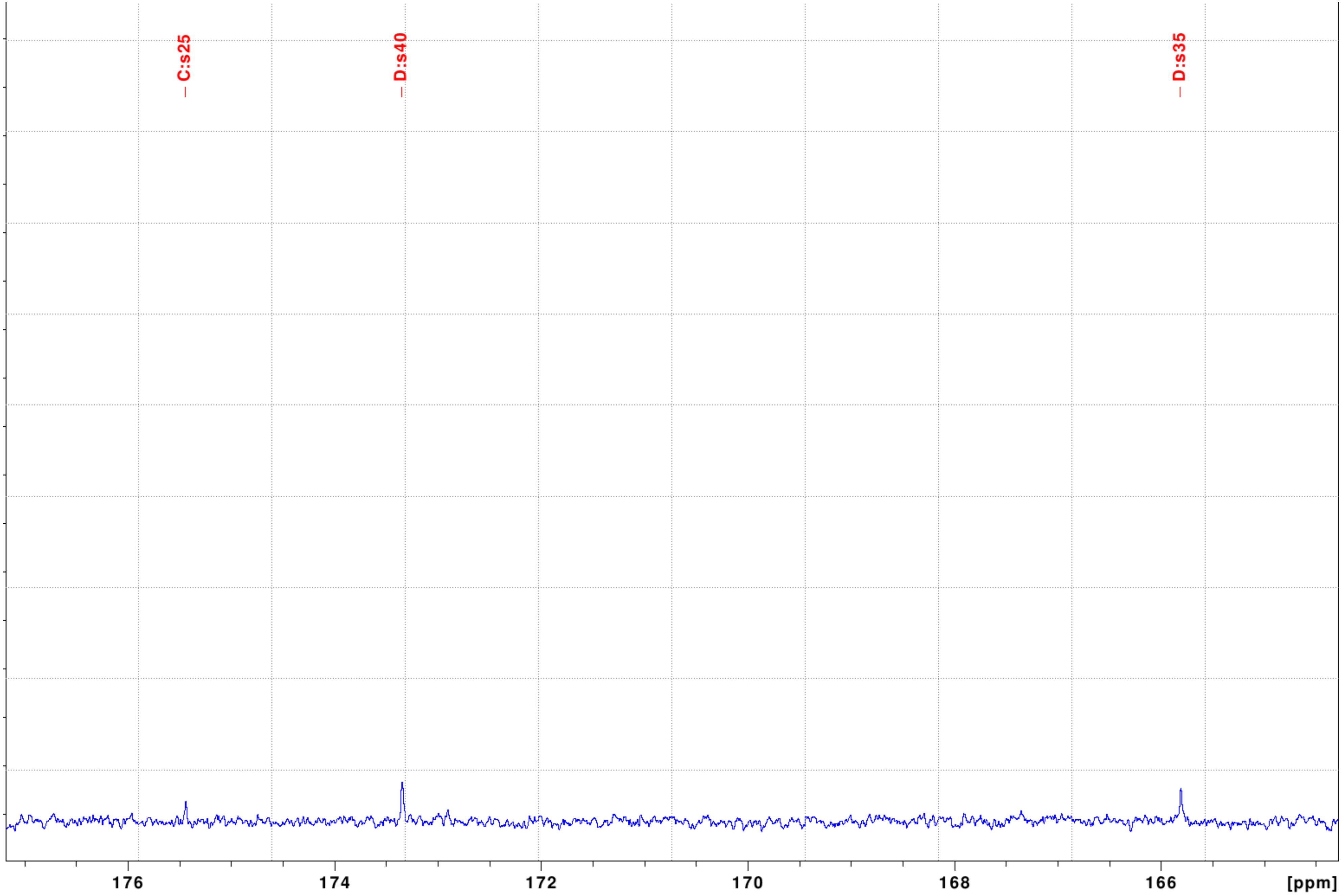
C:s25
D:s40
D:s35
176
174
172
170
168
166
[ppm]

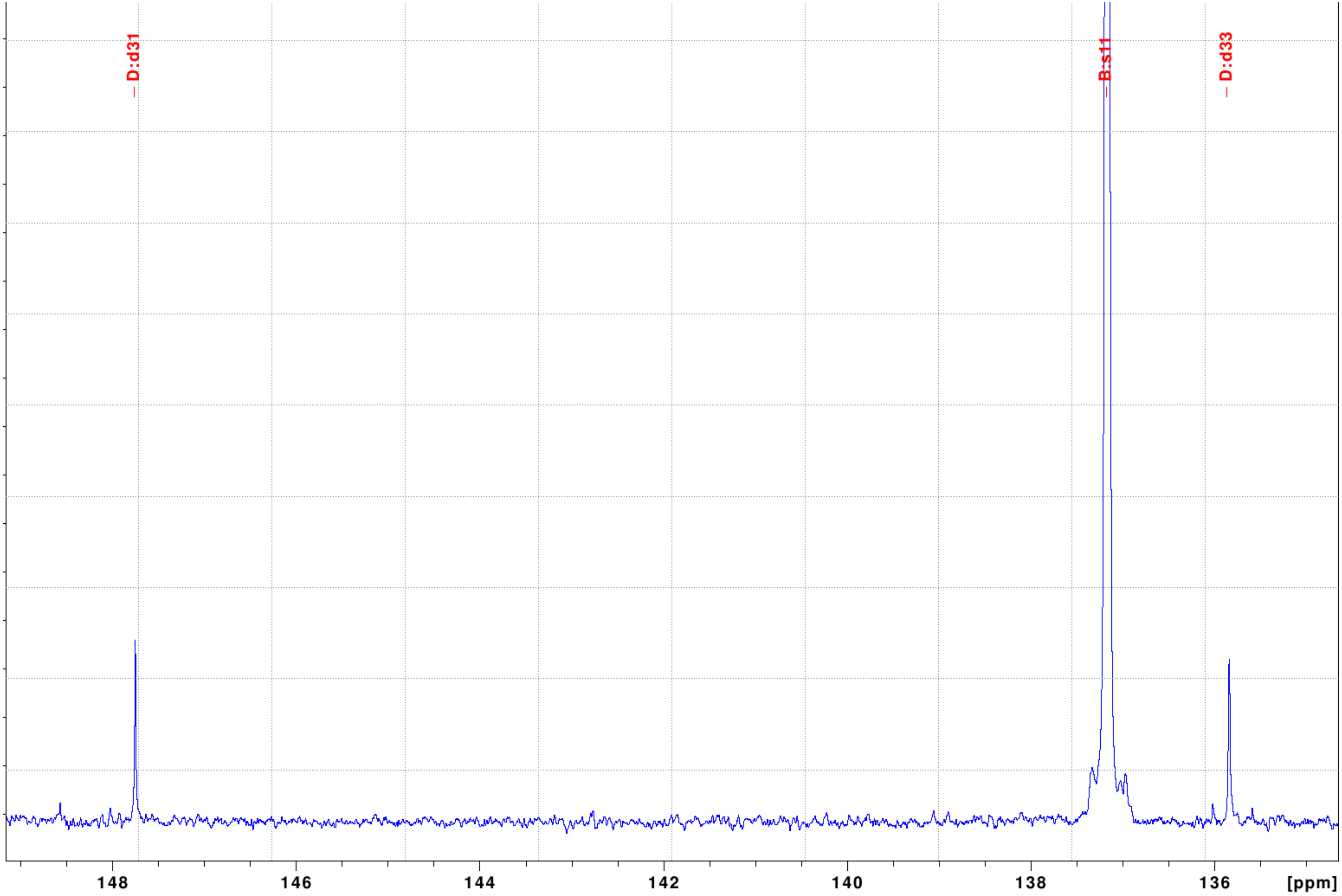
D:d31
B:s11
D:d33
148
146
144
142
140
138
136
[ppm]

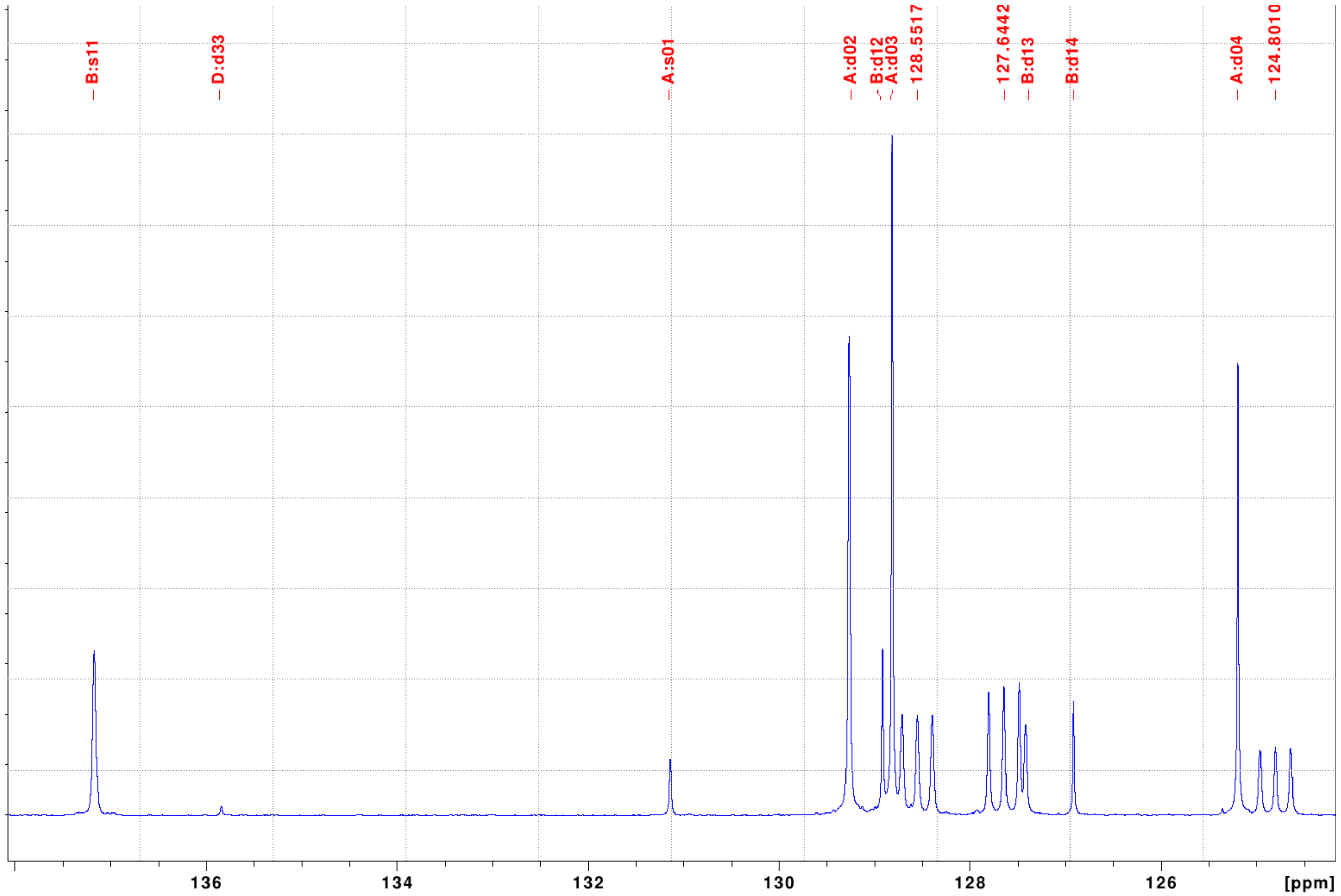
B:s11
D:d33
A:s01
A:d02
B:d12
A:d03
128.5517
127.6442
B:d13
B:d14
A:d04
124.8010
136
134
132
130
128
126
[ppm]

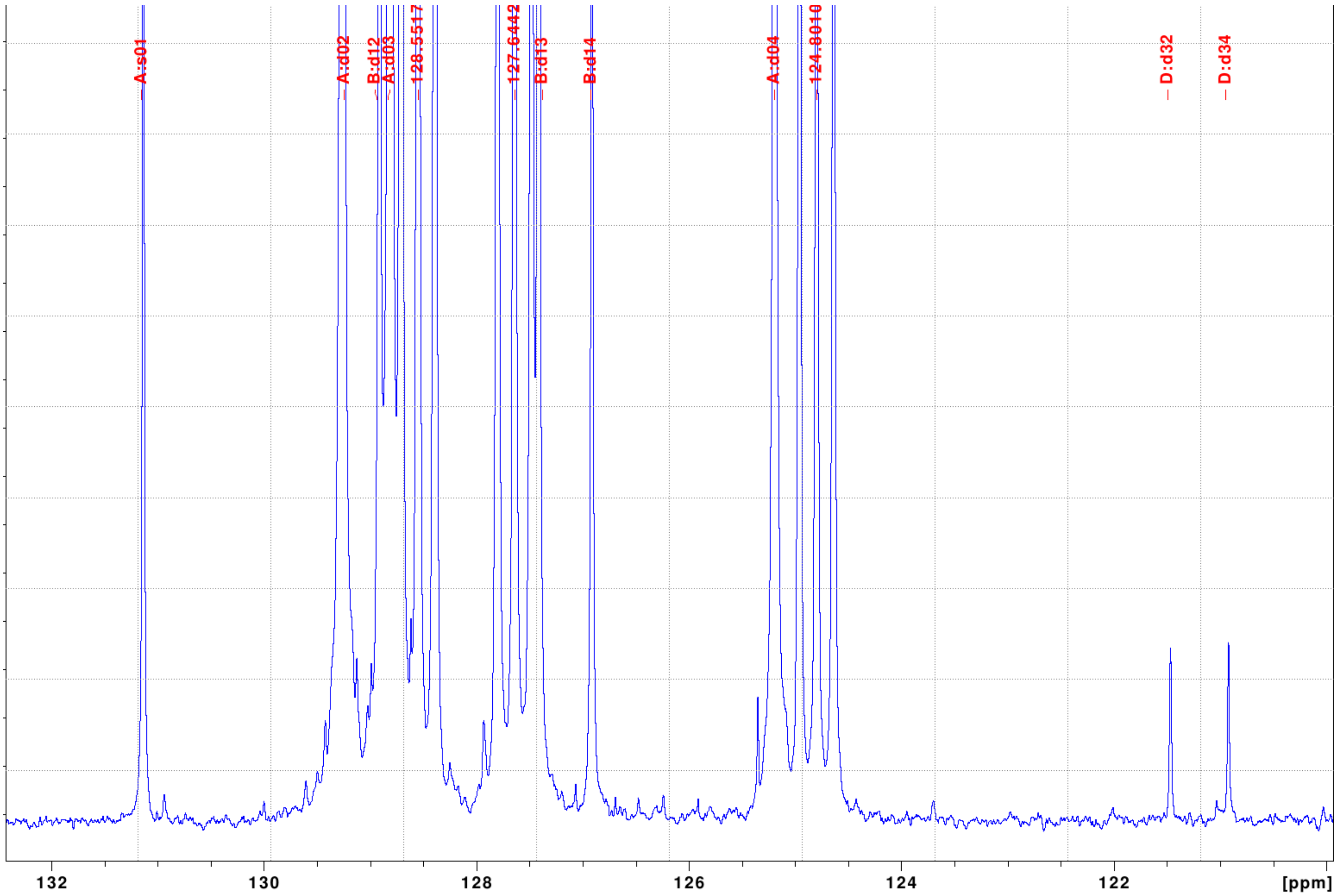
A:s01
A:d02
B:d12
A:d03
128.5517
127.6442
B:d13
B:d14
A:d04
124.8010
D:d32
D:d34
132
130
128
126
124
122
[ppm]

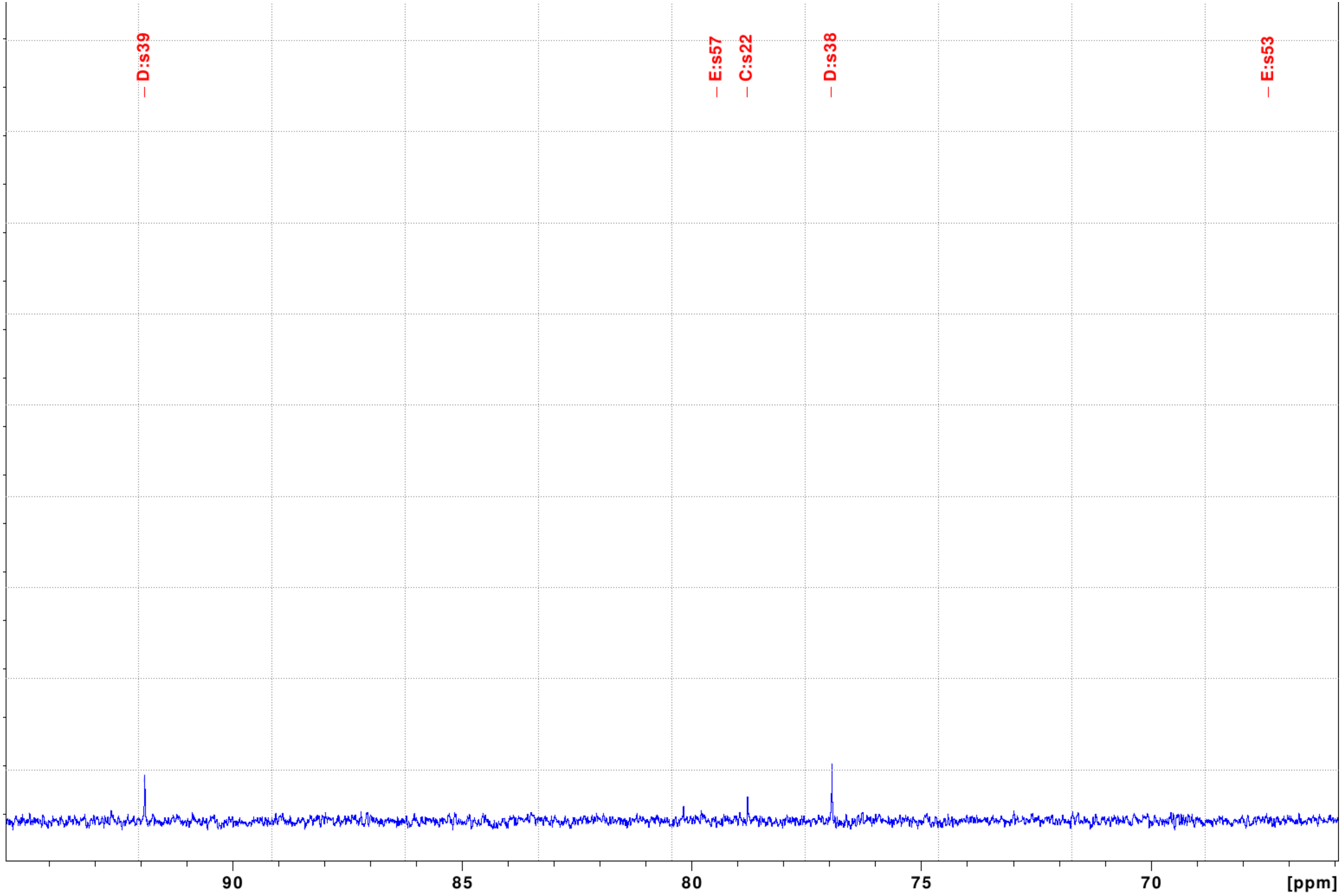
D:s39
E:s57
C:s22
D:s38
E:s53
90
85
80
75
70
[ppm]

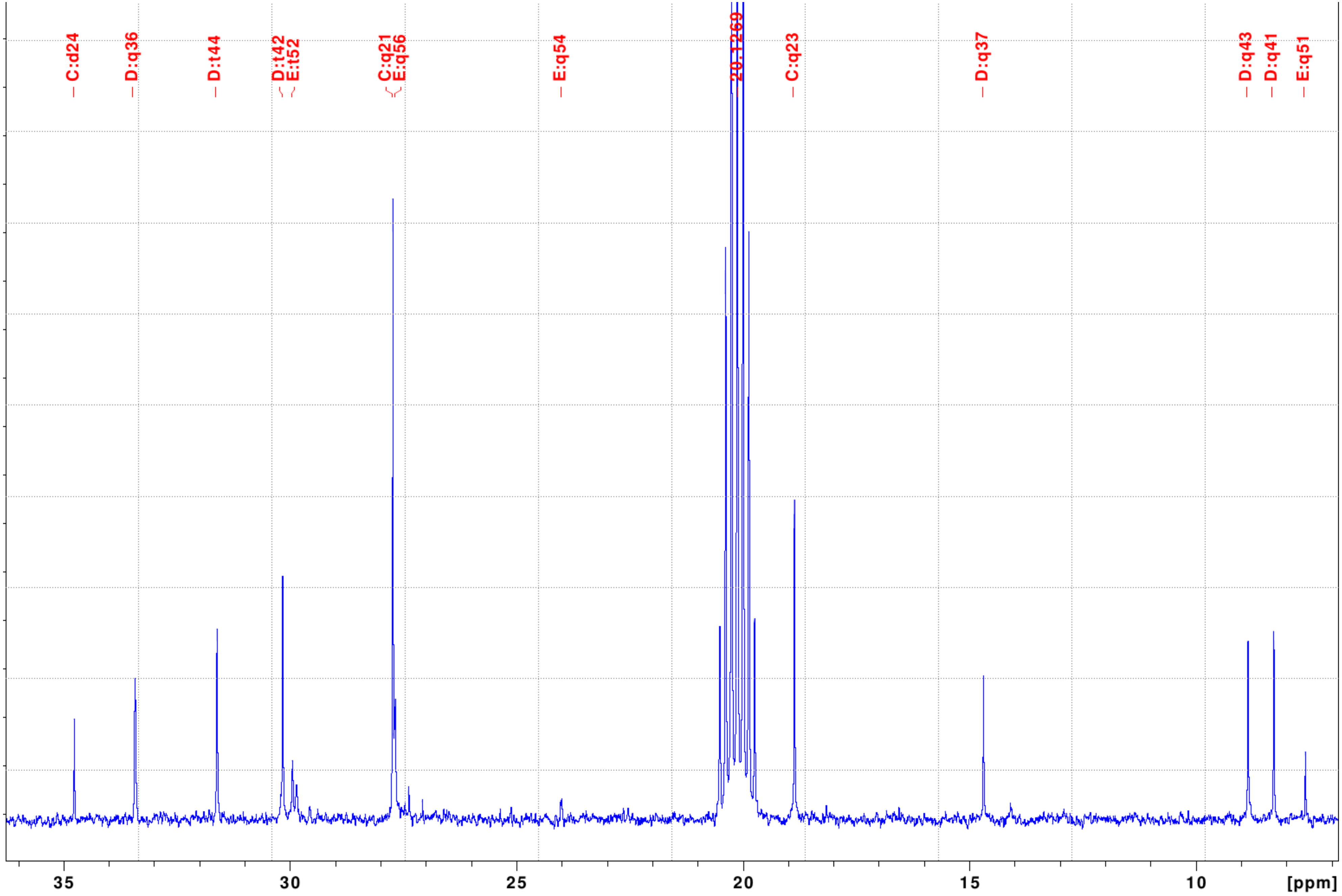

C:d24
D:q36
D:t44
D:t42
E:t52
C:q21
E:q56
E:q54
20.1269
C:q23
D:q37
D:q43
D:q41
E:q51
35
30
25
20
15
10
[ppm]

^{1}H NMR spectrum (600 MHz)

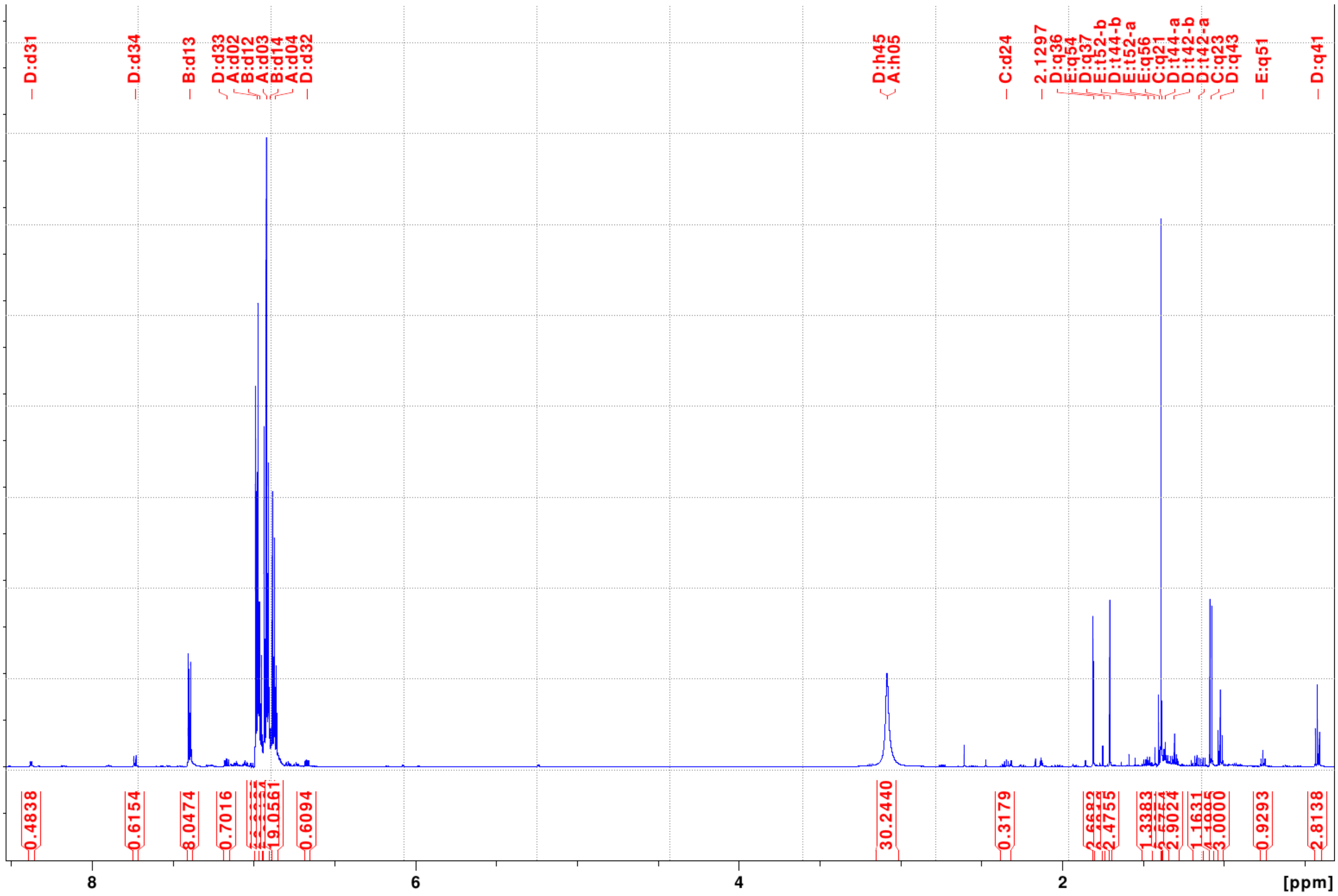

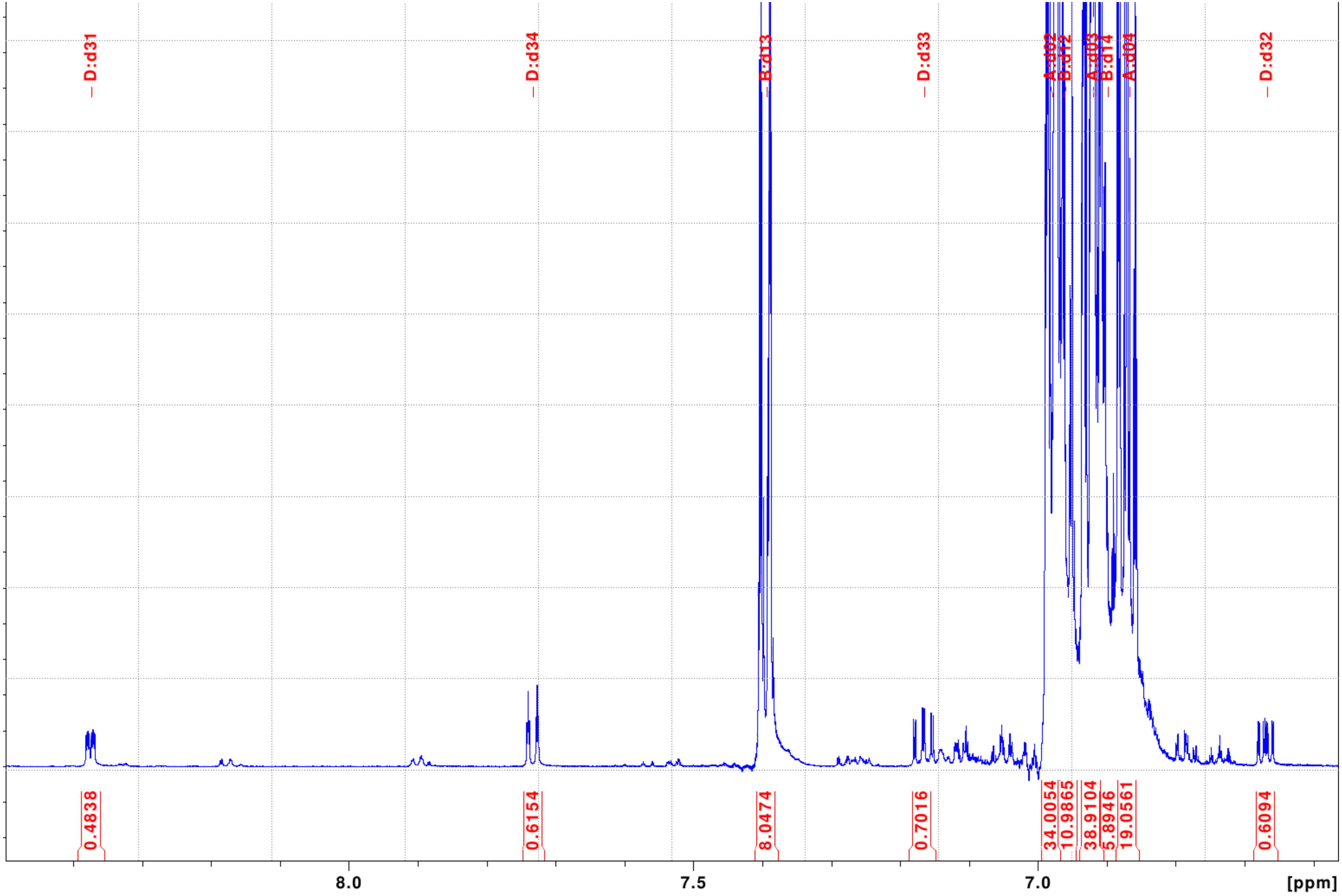
D:d31
D:d34
B:d13
D:d33
A:d02
B:d12
A:d03
B:d14
A:d04
D:d32
0.4838
0.6154
8.0474
0.7016
34.0054
10.9865
38.9104
5.8946
19.0561
0.6094
8.0
7.5
7.0
[ppm]

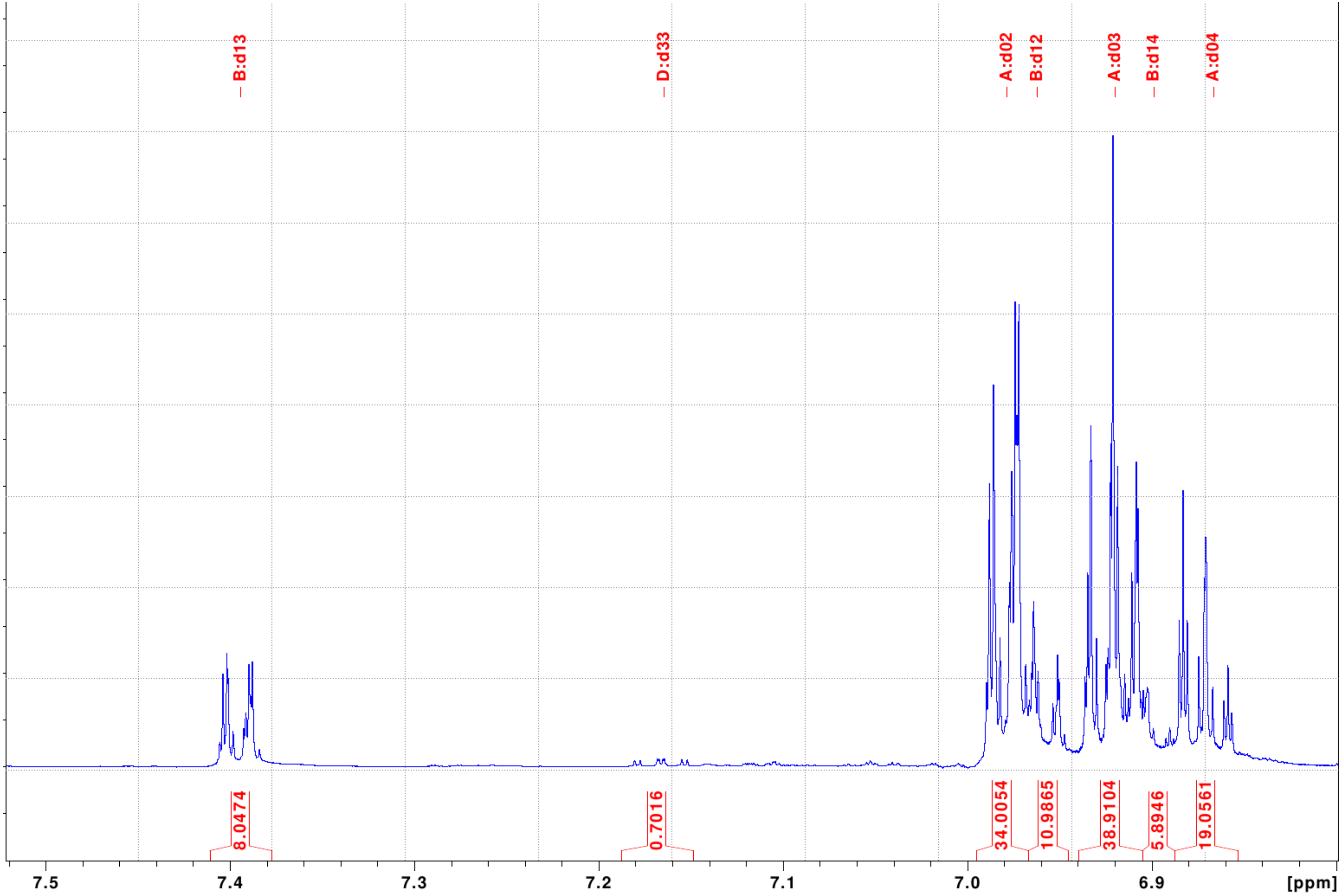
B:d13
D:d33
A:d02
B:d12
A:d03
B:d14
A:d04
8.0474
0.7016
34.0054
10.9865
38.9104
5.8946
19.0561
7.5
7.4
7.3
7.2
7.1
7.0
6.9
[ppm]

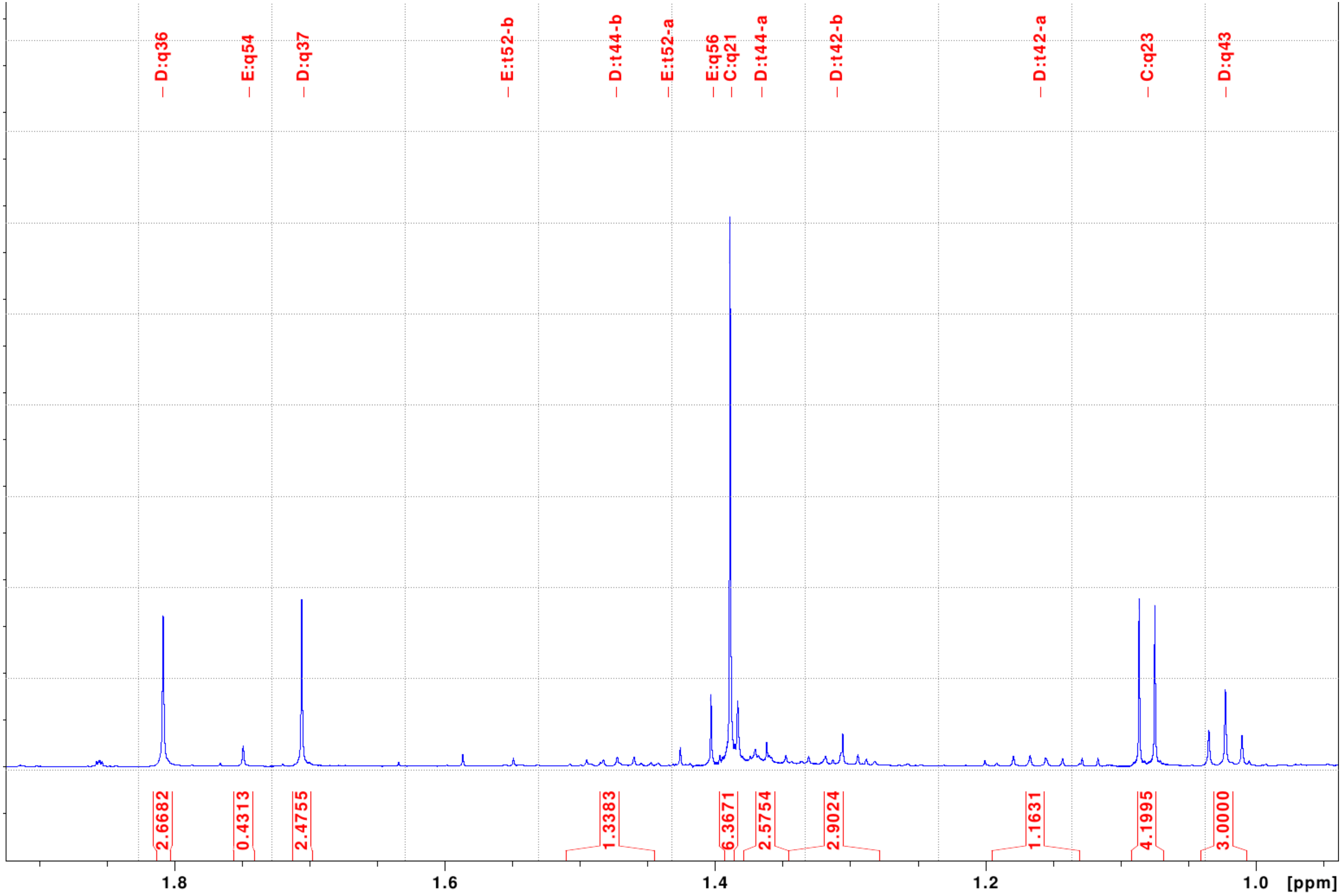

D:q36
E:q54
D:q37
E:t52-b
D:t44-b
E:t52-a
E:q56
C:q21
D:t44-a
D:t42-b
D:t42-a
C:q23
D:q43
2.6682
0.4313
2.4755
1.3383
6.3671
2.5754
2.9024
1.1631
4.1995
3.0000
1.8
1.6
1.4
1.2
1.0
[ppm]

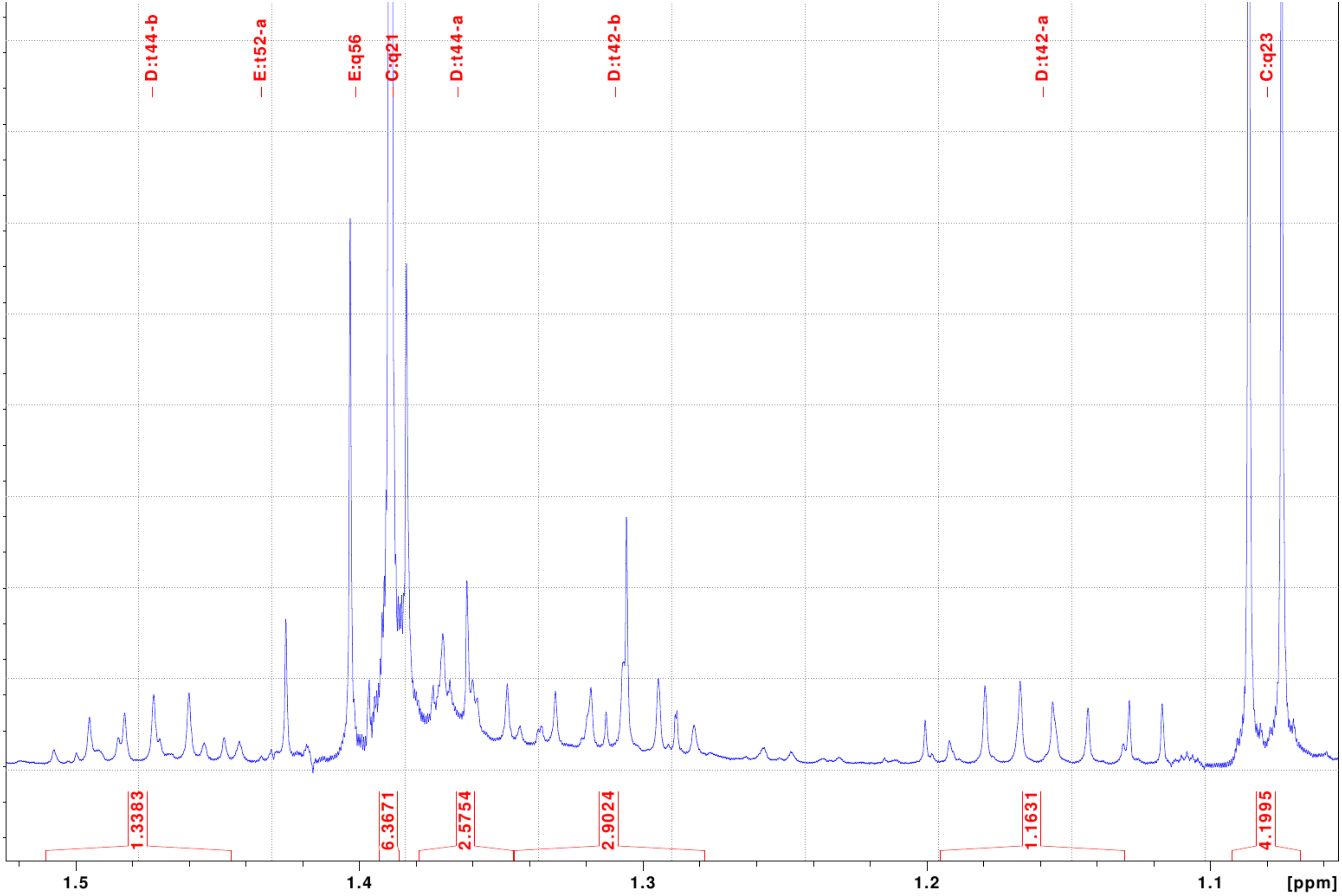

D:t44-b
E:t52-a
E:q56
C:q21
D:t44-a
D:t42-b
D:t42-a
C:q23
1.3383
6.3671
2.5754
2.9024
1.1631
4.1995
1.5
1.4
1.3
1.2
1.1
[ppm]

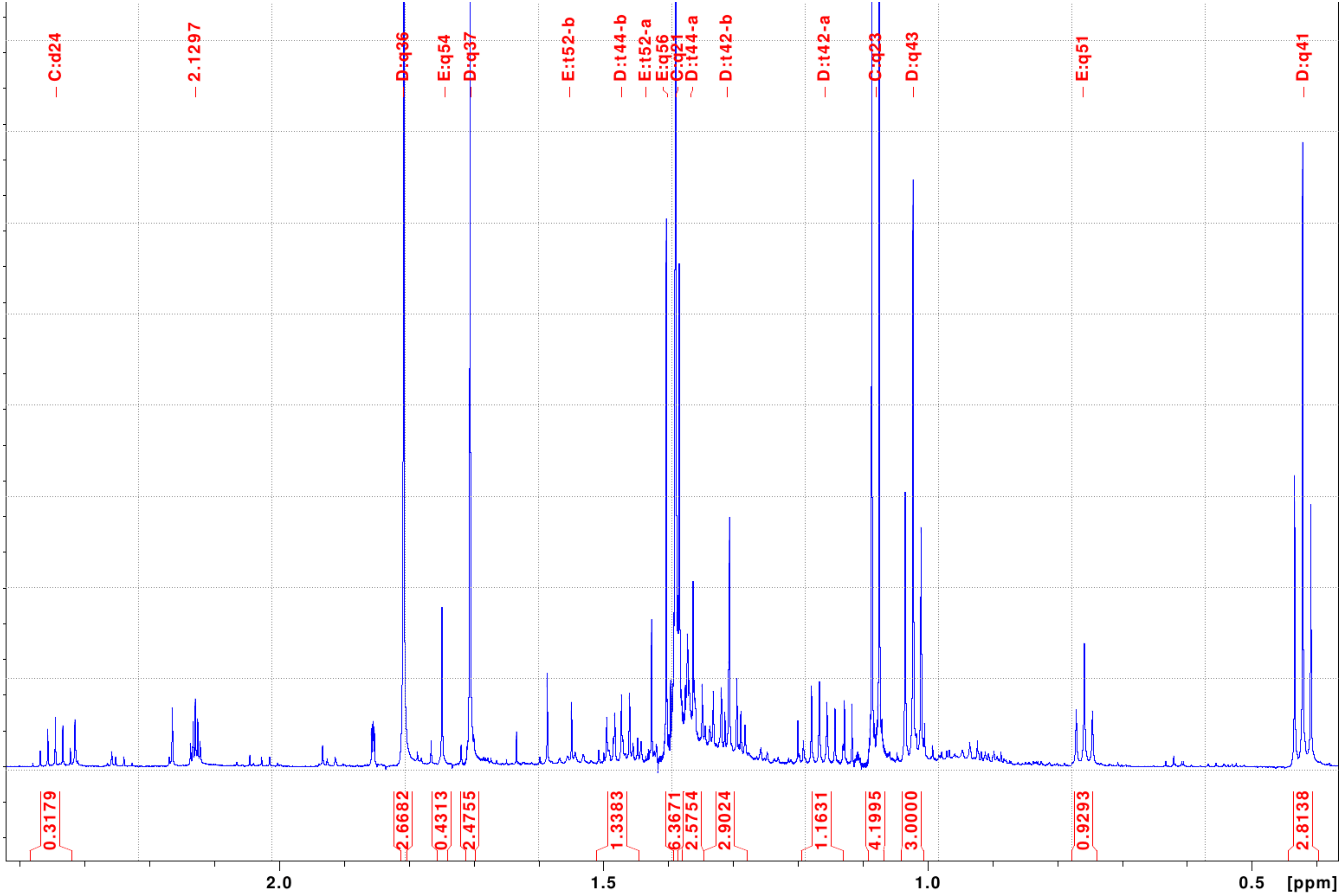
C:d24
2.1297
D:q36
E:q54
D:q37
E:t52-b
D:t44-b
E:t52-a
E:q56
C:q21
D:t44-a
D:t42-b
D:t42-a
C:q23
D:q43
E:q51
D:q41
0.3179
2.6682
0.4313
2.4755
1.3383
6.3671
2.5754
2.9024
1.1631
4.1995
3.0000
0.9293
2.8138
2.0
1.5
1.0
0.5
[ppm]

Structures and NMR signal assignments for products in the reaction mixture 1• + PhSH

in toluene-d$_8$ at 25 °C

Signal assignments

Some peak labels in NMR spectra could not be assigned to structures because of low product content.

Molecule A: PhSH, D ~ 1.85e-9

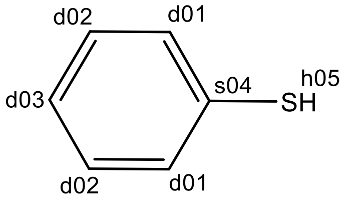

Experiment Bruker_142, 1D 13C: 4 peaks
d01 129.2
d02 128.8
d03 125.2
s04 131.2

Experiment Bruker_141, 1D 1H: 4 peaks
d01-H 6.99
d02-H 6.92
d03-H 6.87
h05-H 3.13

Experiment Bruker_145, 2D 13C-1H via onebond (HSQC): 3 peaks
d01-H - d01
d02-H - d02
d03-H - d03

Experiment Bruker_148, 2D 1H-13C via onebond (H-C correlation): 3 peaks
d01 - d01-H
d02 - d02-H
d03 - d03-H

Experiment Bruker_144, 2D 1H-1H via Jcoupling (COSY): 4 peaks
d01-H - d02-H
d02-H - d01-H d03-H
d03-H - d02-H

Experiment Bruker_146, 2D 13C-1H via Jcoupling (HMBC): 5 peaks
d01-H - d01 d03
d02-H - d02 s04
d03-H - d01

Experiment Bruker_147, 2D 1H-1H via through-space (NOESY): 2 peaks
d01-H - h05-H
h05-H - d01-H

Molecule B: PhSSPh, D ~ 1.22e-9

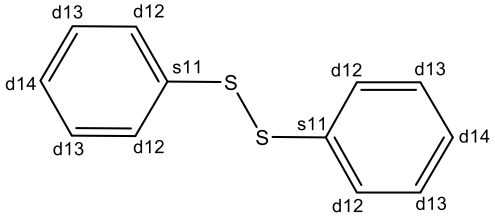

Experiment Bruker_142, 1D 13C: 4 peaks
s11 137.2
d12 127.4
d13 128.9
d14 126.9

Experiment Bruker_141, 1D 1H: 3 peaks
d12-H 7.39
d13-H 6.97
d14-H 6.91

Experiment Bruker_145, 2D 13C-1H via onebond (HSQC): 3 peaks
d12-H - d12
d13-H - d13
d14-H - d14

Experiment Bruker_148, 2D 1H-13C via onebond (H-C correlation): 3 peaks
d12 - d12-H
d13 - d13-H
d14 - d14-H

Experiment Bruker_144, 2D 1H-1H via Jcoupling (COSY): 5 peaks
d12-H - d13-H d14-H(weak)
d13-H - d12-H
d14-H - d12-H(weak) d13-H?

Experiment Bruker_146, 2D 13C-1H via Jcoupling (HMBC): 6 peaks
d12-H - d12 d14 s11(weak)
d13-H - d13 s11
d14-H - d12

Molecule C: amine, D ~ 0.99e-9

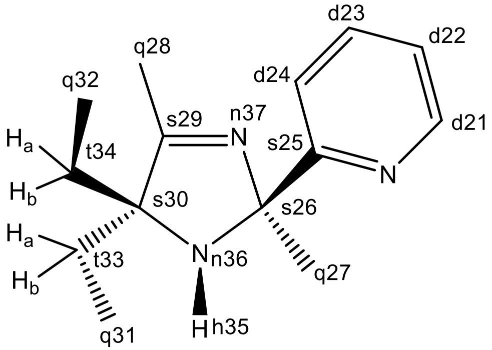

Experiment Bruker_142, 1D 13C: 14 peaks
d21 147.7
d22 121.7
d23 136.0
d24 120.9
s25 165.1
s26 92.1
q27 33.0
q28 14.7
s29 173.5
s30 77.3
q31 8.9
q32 8.3
t33 31.3
t34 30.1

Experiment Bruker_141, 1D 1H: 13 peaks
d21-H 8.36
d22-H 6.67
d23-H 7.17
d24-H 7.71
q27-H 1.81
q28-H 1.68
q31-H 1.01
q32-H 0.42
t33-a 1.35
t33-b 1.52
t34-a 1.16
t34-b 1.34
h35-H 3.13

Experiment Bruker_156, 1D 15N: 2 peaks
n36-N1 61.55
n37-N1 327.21

Experiment Bruker_145, 2D 13C-1H via onebond (HSQC): 12 peaks
d21-H - d21(178 Hz)
d22-H - d22(164 Hz)
d23-H - d23(161 Hz)
d24-H - d24(165 Hz)
q27-H - q27(128 Hz)
q28-H - q28(127 Hz)
q31-H - q31(126 Hz)
q32-H - q32(126 Hz)
t33-a - t33
t33-b - t33
t34-a - t34

t34-b - t34

Experiment Bruker_148, 2D 1H-13C via onebond (H-C correlation): 12 peaks
d21 - d21-H
d22 - d22-H
d23 - d23-H
d24 - d24-H
q27 - q27-H
q28 - q28-H
q31 - q31-H
q32 - q32-H
t33 - t33-a t33-b
t34 - t34-a t34-b

Experiment Bruker_144, 2D 1H-1H via Jcoupling (COSY): 18 peaks
d21-H - d22-H
d22-H - d21-H d23-H
d23-H - d22-H d24-H
d24-H - d23-H
q31-H - t33-a t33-b
q32-H - t34-a t34-b
t33-a - q31-H t33-b
t33-b - q31-H t33-a
t34-a - q32-H t34-b
t34-b - q32-H t34-a

Experiment Bruker_146, 2D 13C-1H via Jcoupling (HMBC): 35 peaks
d21-H - d22 d23 s25
d22-H - d21 d24
d23-H - d21 s25
d24-H - d22 s25(weak) s26
q27-H - s25 s26
q28-H - s25(weak) s26(weak) s29 s30
q31-H - s30 t33
q32-H - s30 t34
t33-a - q31 s30 t34
t33-b - q31 s29 s30 t34(weak)
t34-a - q32 s29(weak) s30 t33
t34-b - q32 s29 s30 t33

Experiment Bruker_147, 2D 1H-1H via through-space (NOESY): 17 peaks
d24-H - q27-H
h35-H - q32-H t33-a?
q27-H - d24-H q31-H
q28-H - q31-H q32-H t34-a
q31-H - q27-H q28-H
q32-H - h35-H q28-H t33-b
t33-b - q32-H
t34-a - q28-H t33-b?
t34-b - h35-H?

Experiment Bruker_153, 2D 15N-1H via Jcoupling: 7 peaks
q27-H - n36-N1 n37-N1
q28-H - n37-N1
t33-a - n36-N1
t33-b - n36-N1
t34-a - n36-N1
t34-b - n36-N1

Experiment Bruker_154, 2D 15N-1H via Jcoupling: 7 peaks
q27-H - n36-N1 n37-N1
q28-H - n37-N1
t33-a - n36-N1
t33-b - n36-N1
t34-a - n36-N1
t34-b - n36-N1

Molecule E: D ~ 0.84e-9

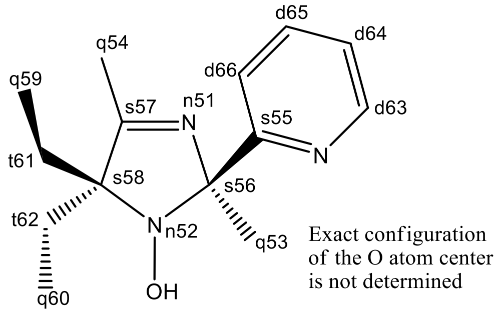

Exact configuration of the O atom center is not determined

Experiment Bruker_142, 1D 13C: 14 peaks
q53 24.7
q54 16.6
s55 164.7
s56 94.6
s57 174.8
s58 80.1
q59 8.5
q60 10.5
t61 29.5
t62 26.2
d63 146.4
d64 122.1
d65 137.8
d66 121.2

Experiment Bruker_141, 1D 1H: 12 peaks
q53-H 1.87
q54-H 1.73
q59-H 0.58
q60-H 1.00
t61-a 1.27
t61-b 1.53
t62-a 1.51
t62-b 1.95
d63-H 8.57
d64-H 6.63
d65-H 7.10
d66-H 7.59

Experiment Bruker_156, 1D 15N: 2 peaks
n51-N1 324.10
n52-N1 148.83

Experiment Bruker_145, 2D 13C-1H via onebond (HSQC): 12 peaks
d63-H - d63
d64-H - d64
d65-H - d65
d66-H - d66
q53-H - q53
q54-H - q54
q59-H - q59
q60-H - q60
t61-a - t61
t61-b - t61
t62-a - t62
t62-b - t62

Experiment Bruker_148, 2D 1H-13C via onebond (H-C correlation): 10 peaks
d63 - d63-H
d64 - d64-H
d65 - d65-H
d66 - d66-H
q53 - q53-H
q54 - q54-H
q59 - q59-H
q60 - q60-H
t61 - t61-a
t62 - t62-b

Experiment Bruker_144, 2D 1H-1H via Jcoupling (COSY): 23 peaks
d63-H - d64-H d65-H(weak)
d64-H - d63-H d65-H d66-H(weak)
d65-H - d63-H(weak) d64-H d66-H
d66-H - d63-H?(weak) d64-H(weak) d65-H
q59-H - t61-a t61-b
q60-H - t62-a t62-b

t61-a - q59-H t61-b
t61-b - q59-H t61-a
t62-a - q60-H t62-b
t62-b - q60-H t62-a

Experiment Bruker_146, 2D 13C-1H via Jcoupling (HMBC): 27 peaks
d63-H - d64 d65 s55
d64-H - d63 d66
d65-H - d63 s55
d66-H - d64
q53-H - s55 s56
q54-H - s55(weak) s57 s58
q59-H - s58 t61
q60-H - s58 t62
t61-a - q59 s58
t61-b - s57 s58
t62-a - q60 s57 s58
t62-b - q60 s57 s58

Experiment Bruker_147, 2D 1H-1H via through-space (NOESY): 8 peaks
d66-H - q53-H
q53-H - d66-H q60-H? t62-a
q54-H - q59-H t61-b?
q59-H - q54-H
t62-a - q53-H

Experiment Bruker_153, 2D 15N-1H via Jcoupling: 5 peaks
q53-H - n51-N1 n52-N1
q54-H - n51-N1
t61-a - n52-N1
t61-b - n52-N1

Experiment Bruker_154, 2D 15N-1H via Jcoupling: 4 peaks
q53-H - n51-N1 n52-N1
q54-H - n51-N1
t61-b - n52-N1

Molecule F: D ~ 0.84e-9

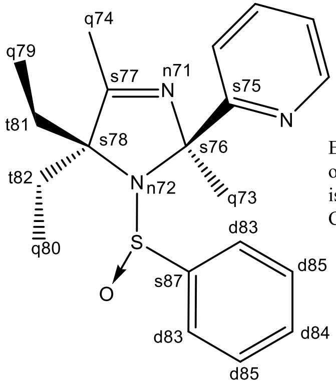

Exact configuration of the S atom center is not determined, CH_{Py} not assigned

Experiment Bruker_142, 1D 13C: 14 peaks
q73   30.2
q74   14.1
s75 164.4
s76   94.5
s77 172.6
s78   84.5
q79   8.8
q80   10.4
t81   29.4
t82   31.1
d83 126.5
d84 130.1
d85 127.9
s87 144.9

Experiment Bruker_141, 1D 1H: 11 peaks
q73-H   2.31
q74-H   1.55
q79-H -0.02
q80-H   1.24
t81-a   0.54
t81-b   0.82
t82-a   1.28
t82-b   2.54
d83-H   8.17
d84-H   7.09
d85-H   7.14

Experiment Bruker_156, 1D 15N: 2 peaks
n71-N1 324.89
n72-N1 111.52

Experiment Bruker_145, 2D 13C-1H via onebond (HSQC): 11 peaks
d83-H - d83
d84-H - d84
d85-H - d85
q73-H - q73
q74-H - q74
q79-H - q79
q80-H - q80
t81-a - t81
t81-b - t81
t82-a - t82
t82-b - t82

Experiment Bruker_148, 2D 1H-13C via onebond (H-C correlation): 7 peaks
d83 - d83-H
d84 - d84-H
d85 - d85-H
q73 - q73-H
q74 - q74-H
q79 - q79-H
q80 - q80-H

Experiment Bruker_144, 2D 1H-1H via Jcoupling (COSY): 14 peaks
d83-H - d84-H(weak) d85-H
d84-H - d83-H(weak)
d85-H - d83-H
q79-H - t81-a t81-b
q80-H - t82-b
t81-a - q79-H t81-b
t81-b - q79-H t81-a
t82-a - t82-b
t82-b - q80-H t82-a

Experiment Bruker_146, 2D 13C-1H via Jcoupling (HMBC): 21 peaks
d83-H - d83 d84
d85-H - s87
q73-H - s75 s76
q74-H - s75(weak) s77 s78
q79-H - s78 t81
q80-H - s78 t82
t81-a - q79 s77 s78
t81-b - q79
t82-a - q80 s78
t82-b - q80 s77 s78

Experiment Bruker_147, 2D 1H-1H via through-space (NOESY): 8 peaks
d83-H - q79-H? t82-b?
q73-H - q80-H
q74-H - q79-H? t81-b?
q80-H - q73-H t81-b
t81-b - q80-H

Experiment Bruker_153, 2D 15N-1H via Jcoupling: 4 peaks
q73-H - n71-N1 n72-N1
q74-H - n71-N1
t82-a - n72-N1

Experiment Bruker_154, 2D 15N-1H via Jcoupling: 4 peaks
q73-H - n71-N1 n72-N1
q74-H - n71-N1
t82-a - n72-N1

Molecule G: D ~ 0.84e-9

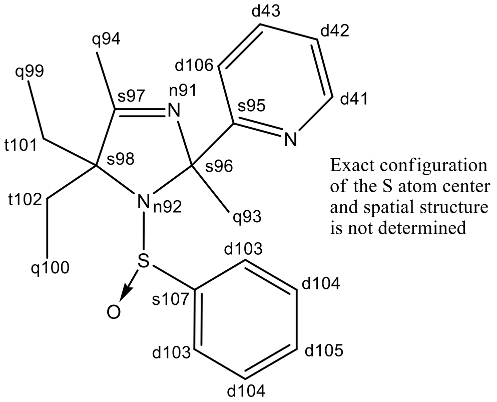

Experiment Bruker_142, 1D 13C: 18 peaks

d41   148.2
d42   121.6
d43   134.9
q93   27.9
q94   15.3
s95   161.5
s96   94.9
s97   171.7
s98   81.9
q99   9.3
q100   9.3
t101   28.4
t102   32.8
d103 126.3
d104 127.8
d105 129.2
d106 120.3
s107 143.2

Experiment Bruker_141, 1D 1H: 15 peaks

d41-H   8.27
d42-H   6.53
d43-H   6.85
q93-H   2.25
q94-H   1.72
q99-H   0.96
q100-H   0.96
t101-a   1.36
t101-b   1.73
t102-a   1.91
t102-b   2.05
d103-H   7.46
d104-H   6.84
d105-H   6.88
d106-H   6.80

Experiment Bruker_156, 1D 15N: 2 peaks

n91-N1 326.96
n92-N1 119.39

Experiment Bruker_145, 2D 13C-1H via onebond (HSQC): 17 peaks

d103-H - d103
d104-H - d104
d105-H - d105
d106-H - d106
d41-H - d41
d42-H - d42
d43-H - d43
q100-H - q100? q99?
q93-H - q93
q94-H - q94
q99-H - q100? q99?
t101-a - t101
t101-b - t101
t102-a - t102
t102-b - t102

Experiment Bruker_148, 2D 1H-13C via onebond (H-C correlation): 12 peaks

d103 - d103-H
d104 - d104-H
d105 - d105-H
d106 - d106-H
d41 - d41-H
d42 - d42-H
d43 - d43-H
q100 - q100-H
q93 - q93-H
q94 - q94-H
q99 - q99-H
t102 - t102-b

Experiment Bruker_144, 2D 1H-1H via Jcoupling (COSY): 15 peaks

d103-H - d104-H
d104-H - d103-H
d41-H - d42-H d43-H(weak)
d42-H - d41-H d43-H
d43-H - d41-H(weak) d42-H
q100-H - t102-a t102-b
q99-H - t101-a? t101-b
t101-b - q99-H
t102-a - q100-H
t102-b - q100-H

Experiment Bruker_146, 2D 13C-1H via Jcoupling (HMBC): 22 peaks

d103-H - d105
d104-H - s107
d106-H - d42
d41-H - d43
d42-H - d106 d41
d43-H - d41 s95
q100-H - s98 t102
q93-H - s95 s96
q94-H - s95(weak) s97 s98
q99-H - s98 t101
t102-a - q100 s98
t102-b - q100 s97 s98

Experiment Bruker_147, 2D 1H-1H via through-space (NOESY): 10 peaks

d103-H - q100-H q93-H
d106-H - q93-H
q100-H - d103-H q93-H
q93-H - d103-H d106-H q100-H
t101-a - t102-a
t102-a - t101-a

Experiment Bruker_153, 2D 15N-1H via Jcoupling: 6 peaks

q93-H - n91-N1 n92-N1
q94-H - n91-N1
t101-a - n92-N1
t102-a - n92-N1
t102-b - n92-N1

Experiment Bruker_154, 2D 15N-1H via Jcoupling: 5 peaks

q93-H - n91-N1 n92-N1
q94-H - n91-N1
t102-a - n92-N1
t102-b - n92-N1

^{13}C{^{1}H} NMR spectrum (150 MHz)

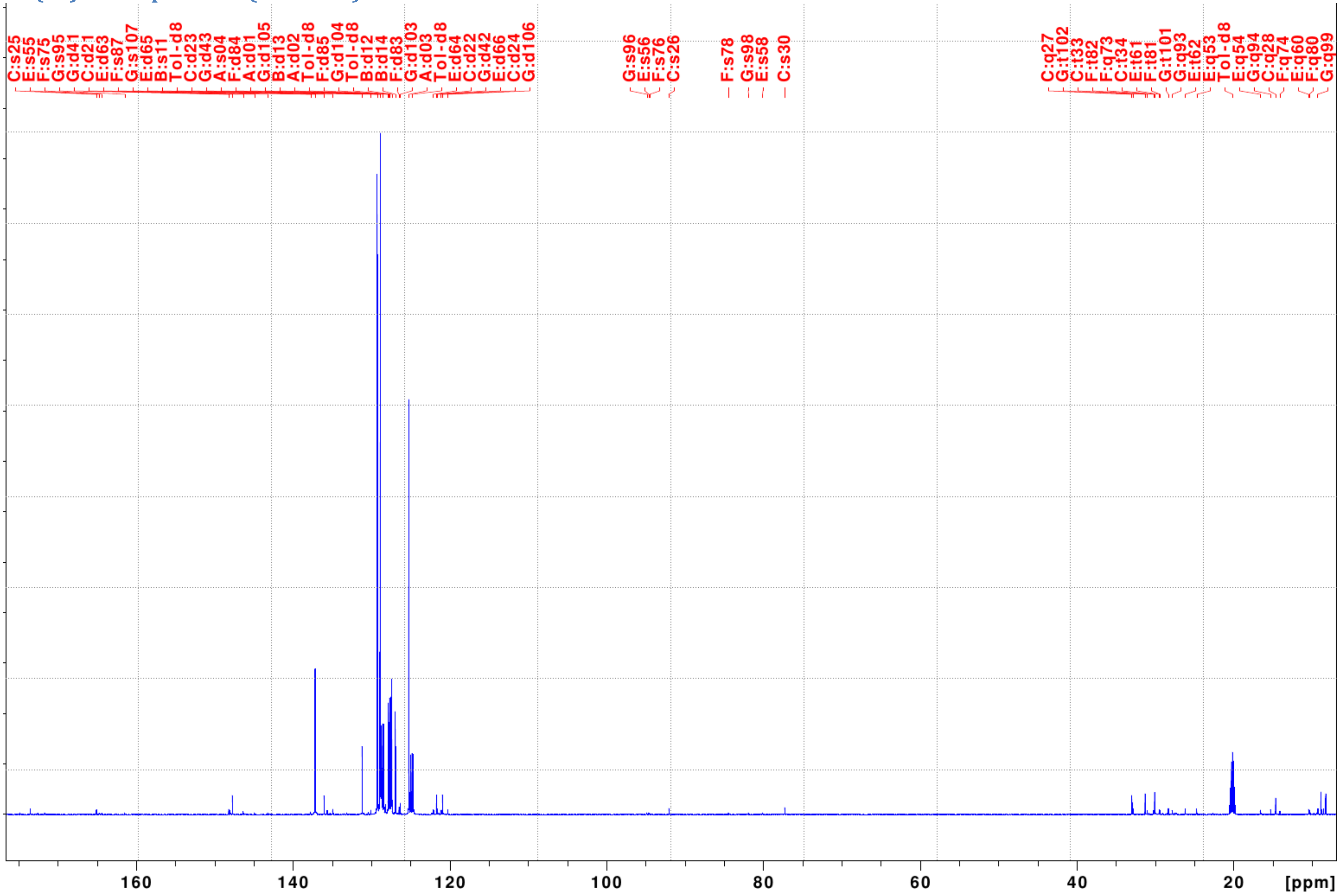

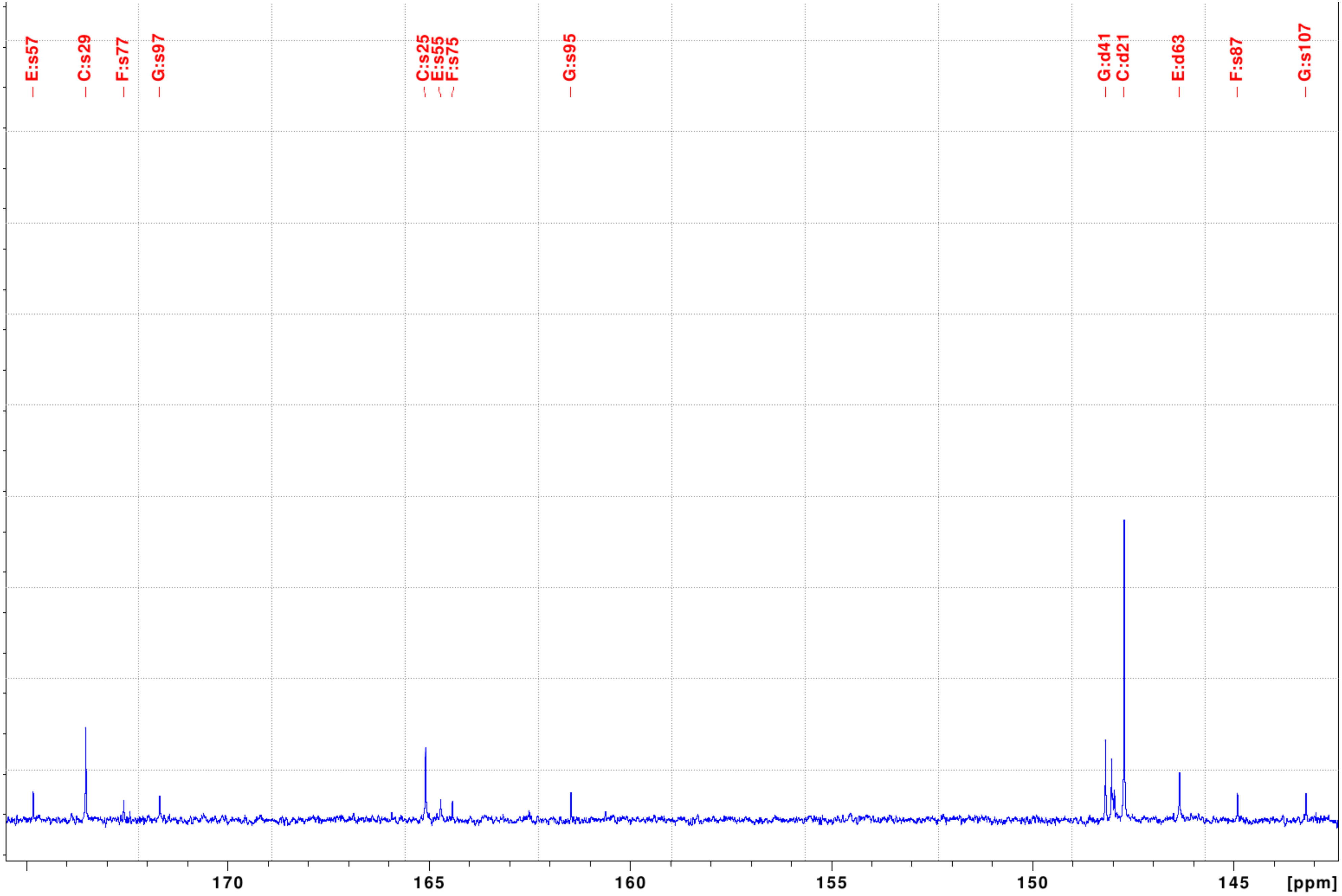
E:s57
C:s29
F:s77
G:s97
C:s25
E:s55
F:s75
G:s95
G:d41
C:d21
E:d63
F:s87
G:s107
170
165
160
155
150
145
[ppm]

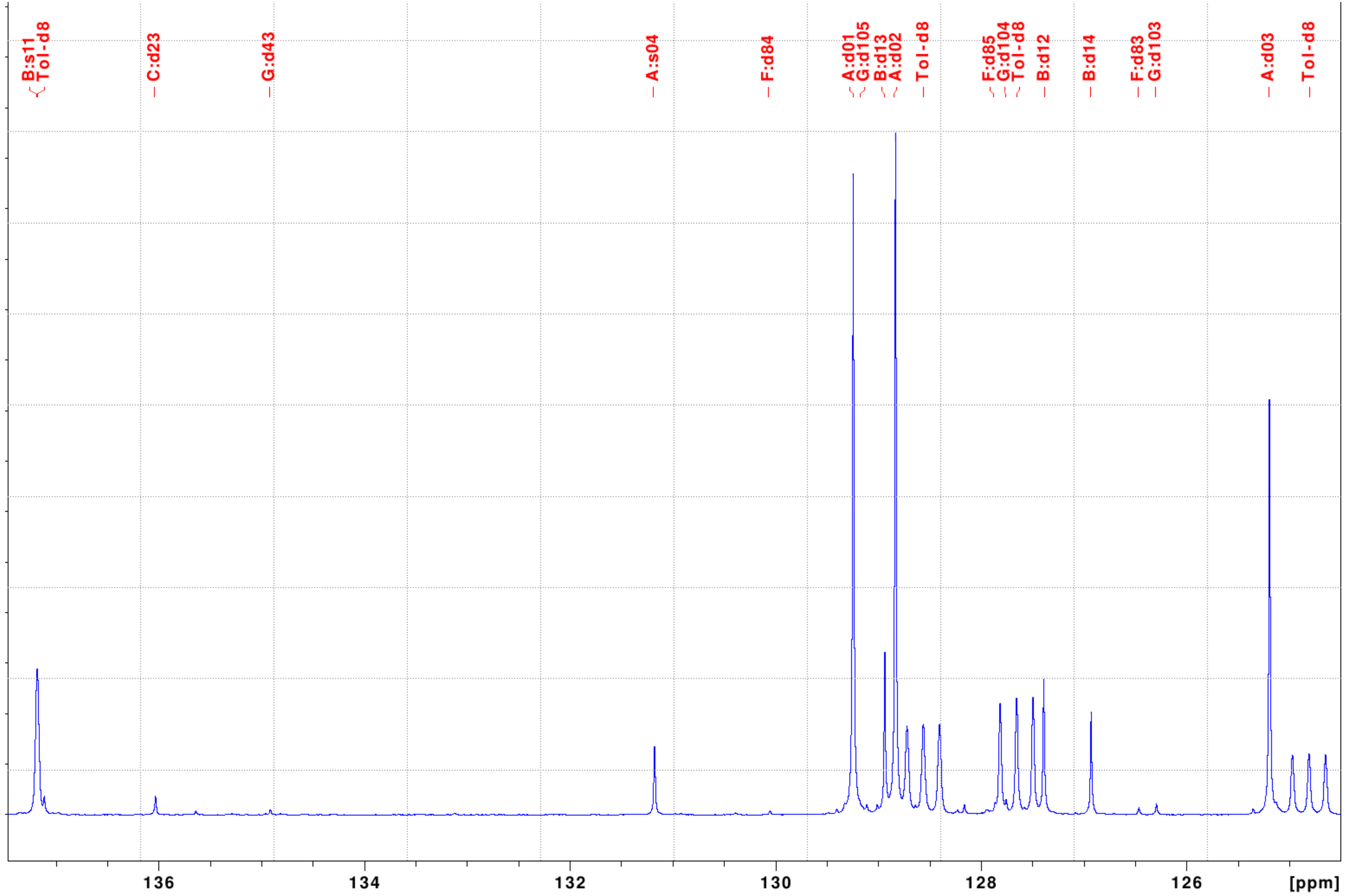

B:s11
Tol-d8
C:d23
G:d43
A:s04
F:d84
A:d01
G:d105
B:d13
A:d02
Tol-d8
F:d85
G:d104
Tol-d8
B:d12
B:d14
F:d83
G:d103
A:d03
Tol-d8
136
134
132
130
128
126
[ppm]

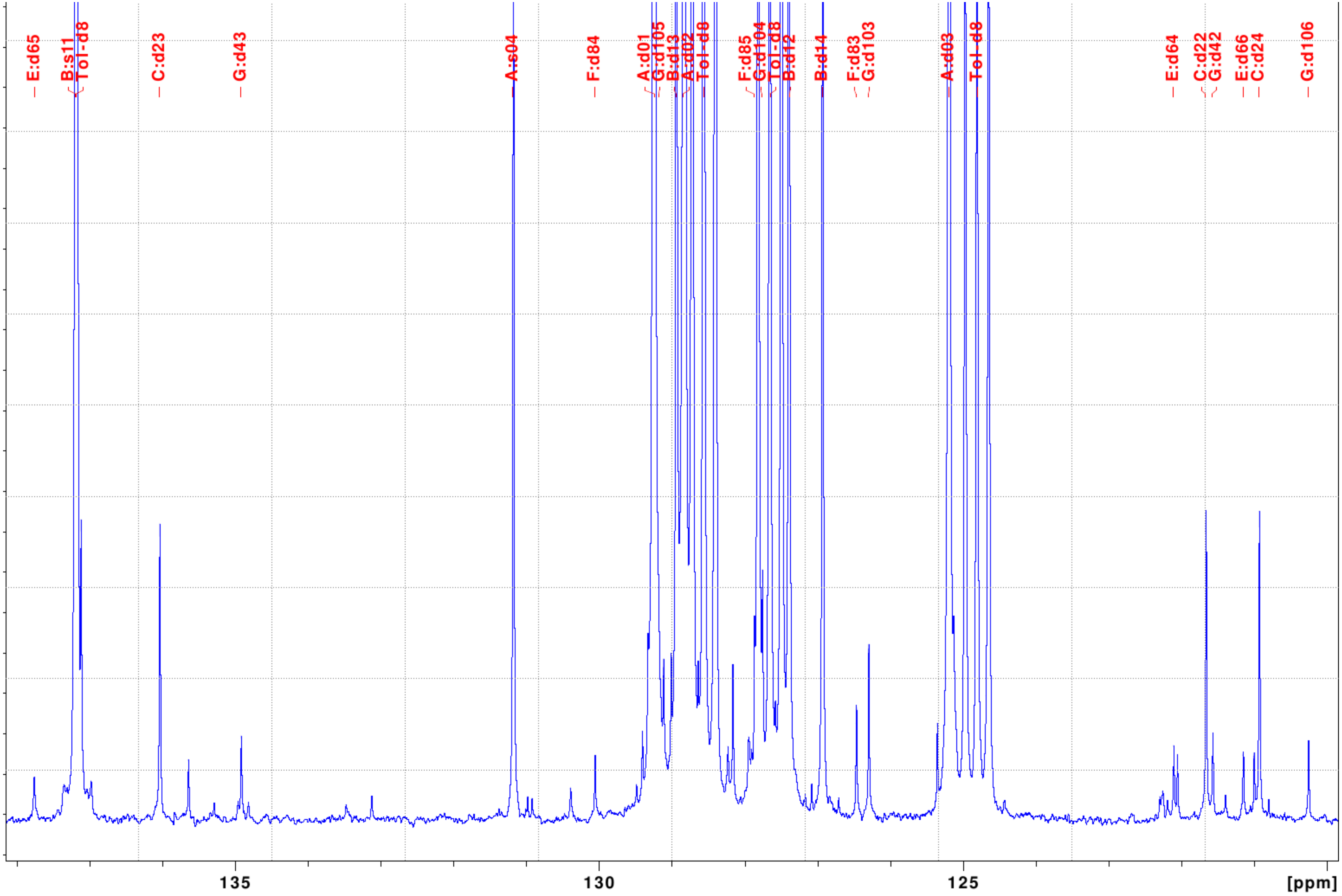

E:d65
B:s11
Tol-d8
C:d23
G:d43
A:s04
F:d84
A:d01
G:d105
B:d13
A:d02
Tol-d8
F:d85
G:d104
Tol-d8
B:d12
B:d14
F:d83
G:d103
A:d03
Tol-d8
E:d64
C:d22
G:d42
E:d66
C:d24
G:d106
135
130
125
[ppm]

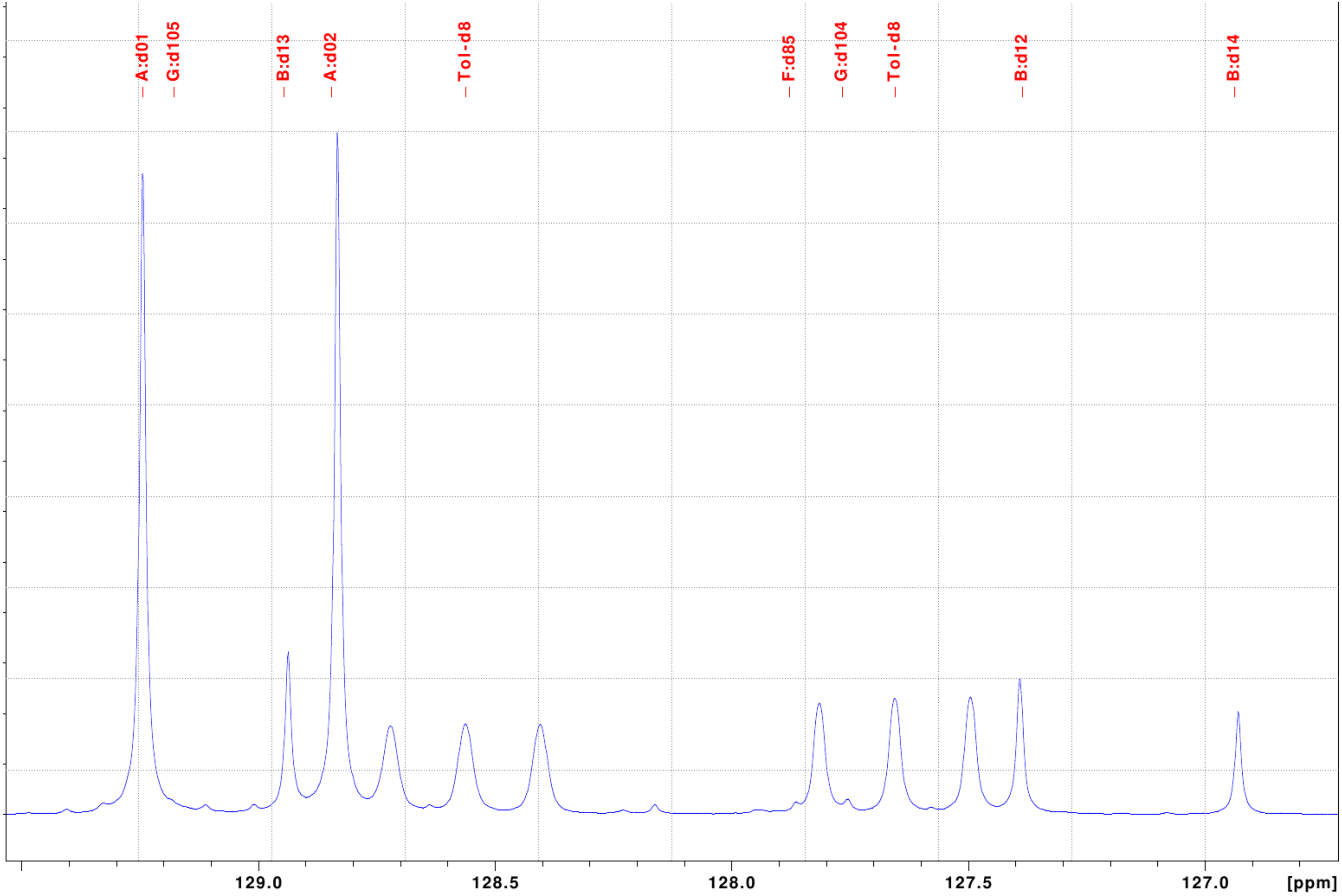
A:d01
G:d105
B:d13
A:d02
Tol-d8
F:d85
G:d104
Tol-d8
B:d12
B:d14
129.0
128.5
128.0
127.5
127.0
[ppm]

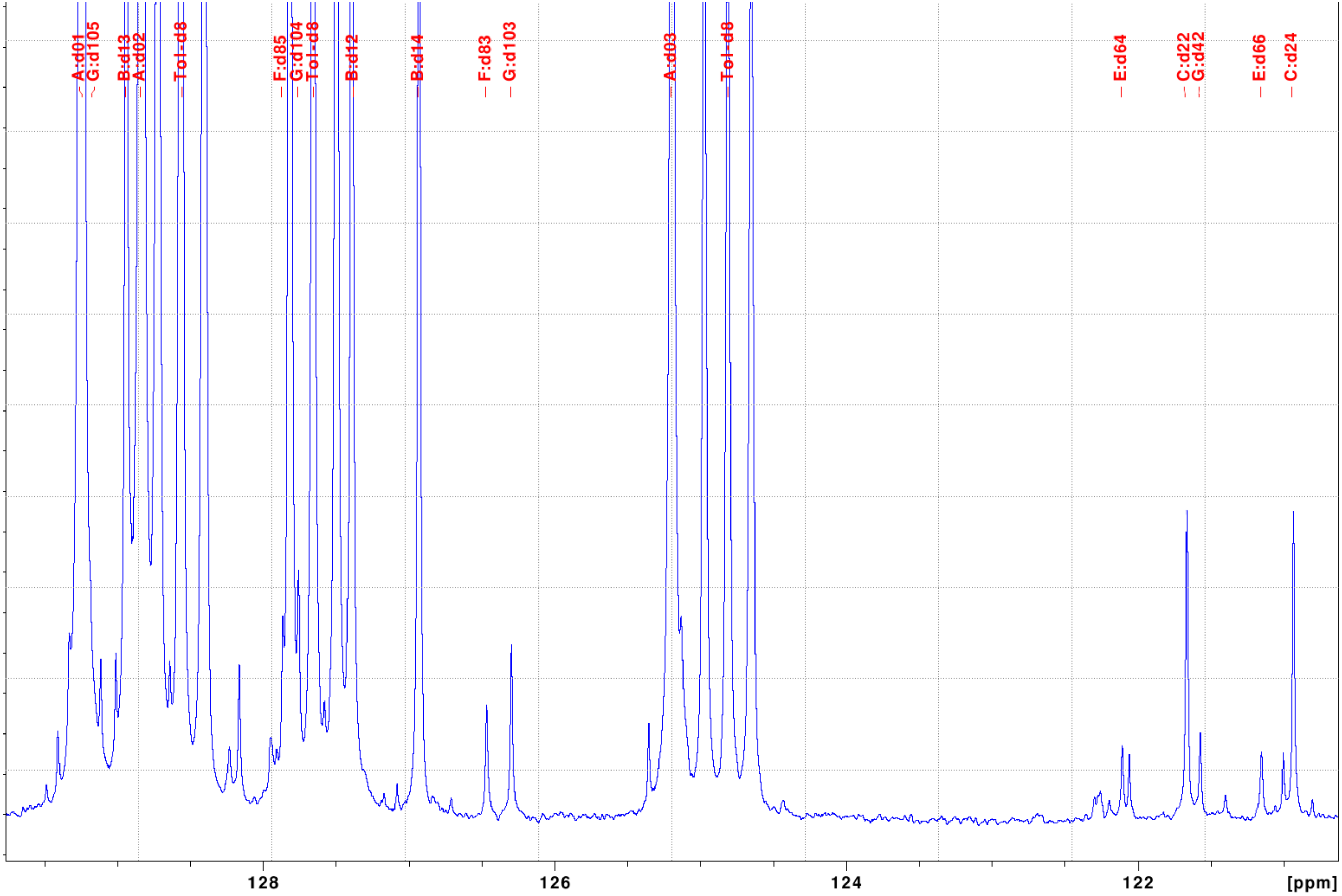

A:d01
G:d105
B:d13
A:d02
Tol-d8
F:d85
G:d104
Tol-d8
B:d12
B:d14
F:d83
G:d103
A:d03
Tol-d8
E:d64
C:d22
G:d42
E:d66
C:d24
128
126
124
122
[ppm]

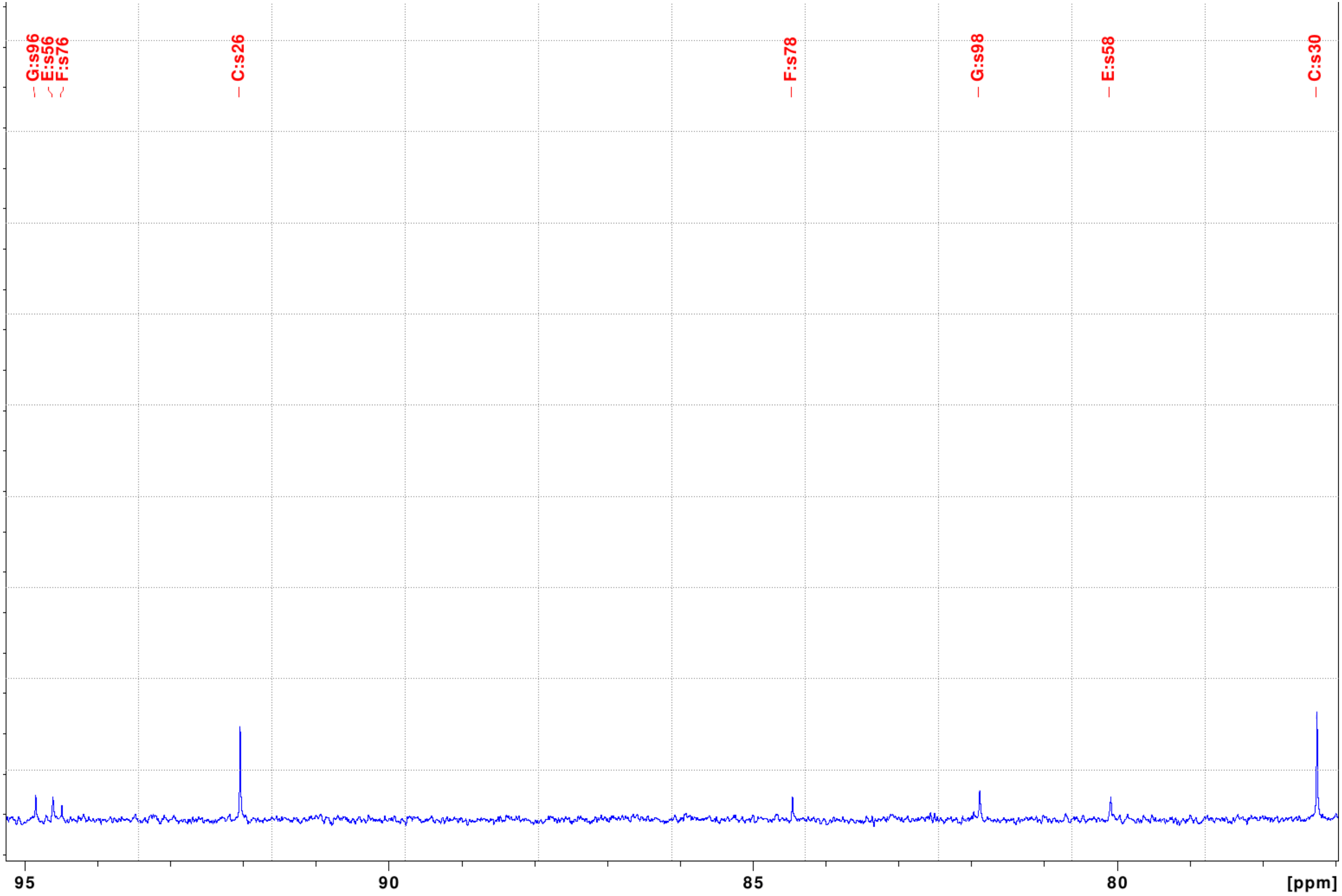
G:s96
E:s56
F:s76
C:s26
F:s78
G:s98
E:s58
C:s30
95
90
85
80
[ppm]

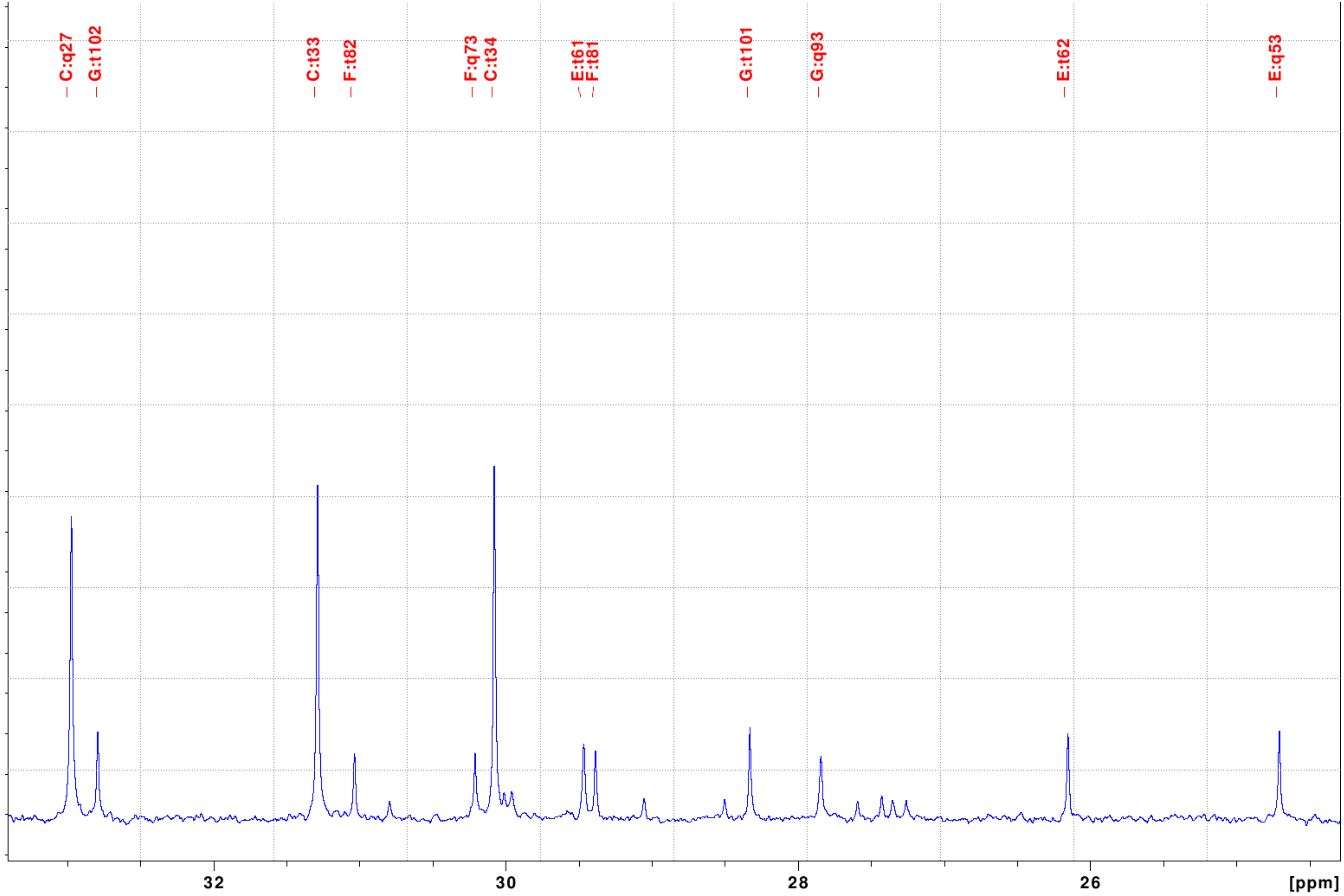

C:q27
G:t102
C:t33
F:t82
F:q73
C:t34
E:t61
F:t81
G:t101
G:q93
E:t62
E:q53
32
30
28
26
[ppm]

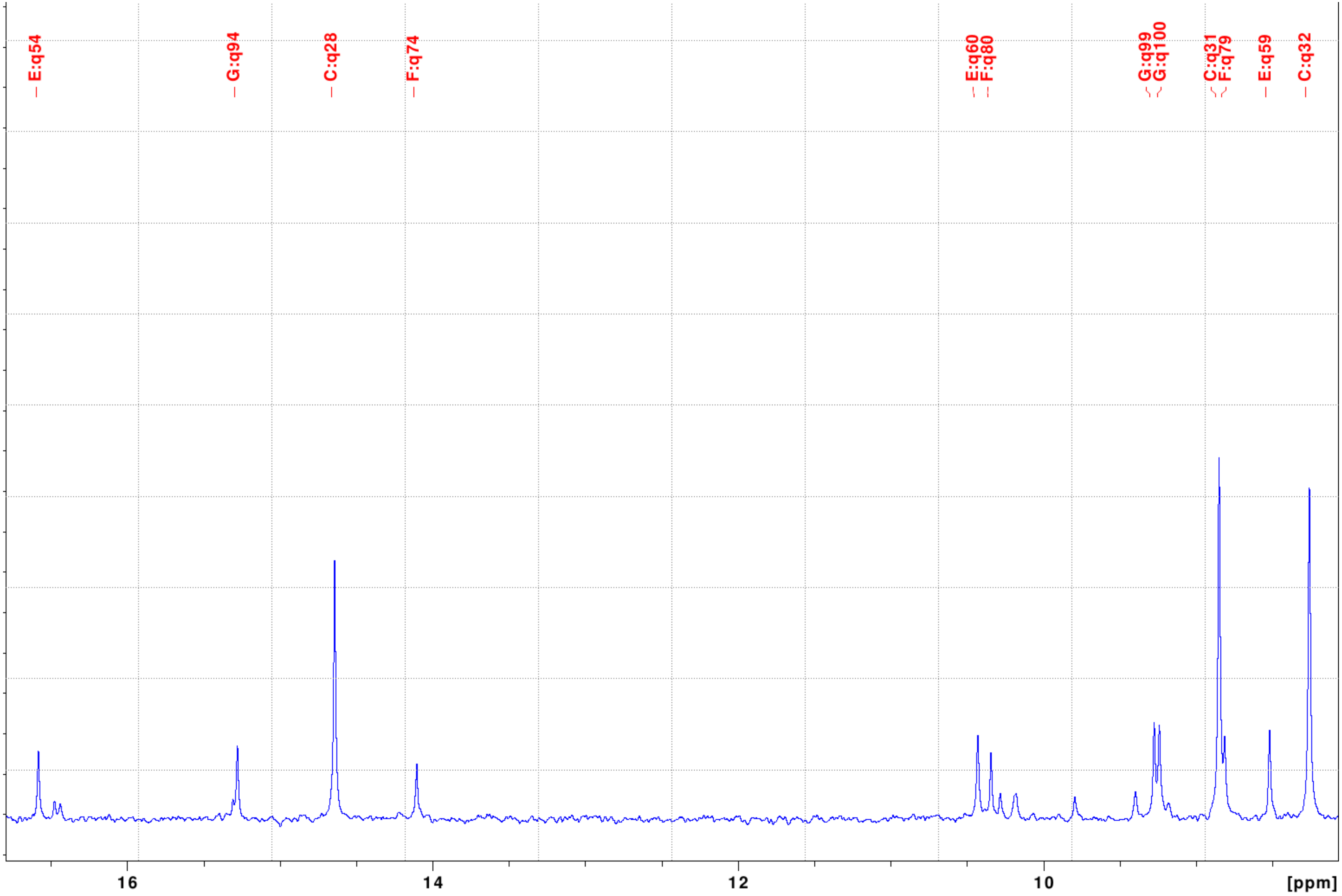
E:q54
G:q94
C:q28
F:q74
E:q60
F:q80
G:q99
G:q100
C:q31
F:q79
E:q59
C:q32
16
14
12
10
[ppm]

^{1}H NMR spectrum (600 MHz)

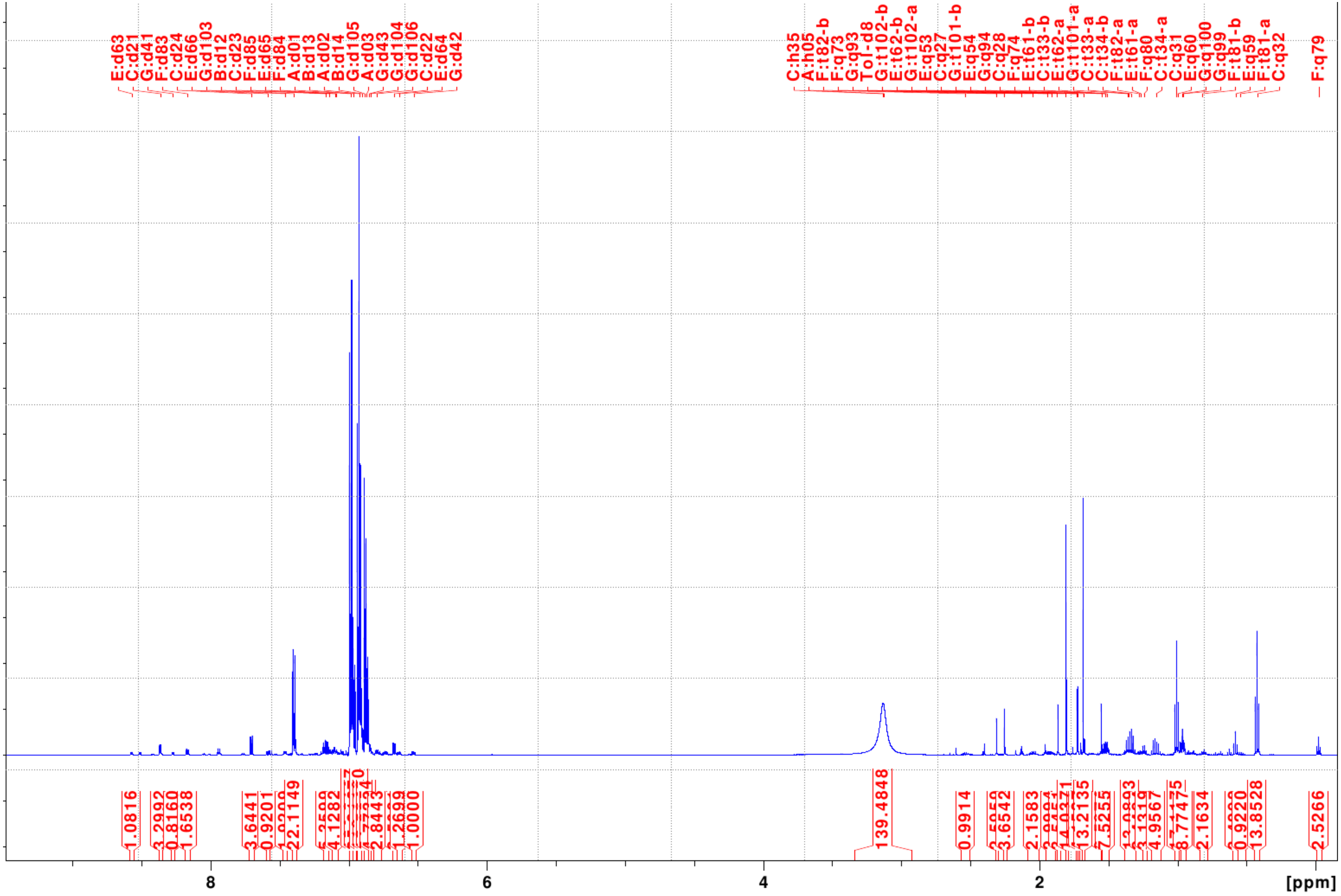

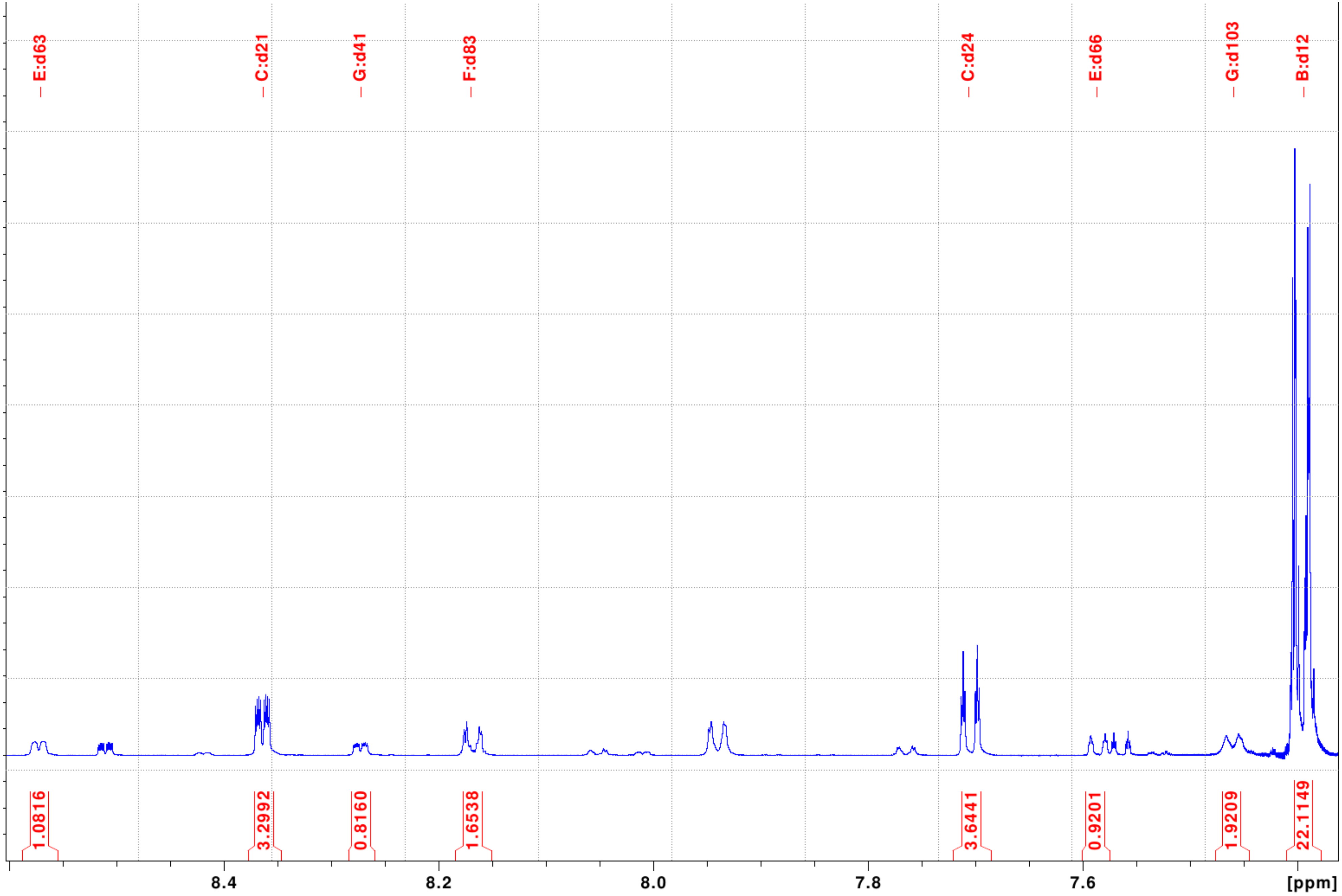

E:d63
C:d21
G:d41
F:d83
C:d24
E:d66
G:d103
B:d12
1.0816
3.2992
0.8160
1.6538
3.6441
0.9201
1.9209
22.1149
8.4
8.2
8.0
7.8
7.6
[ppm]

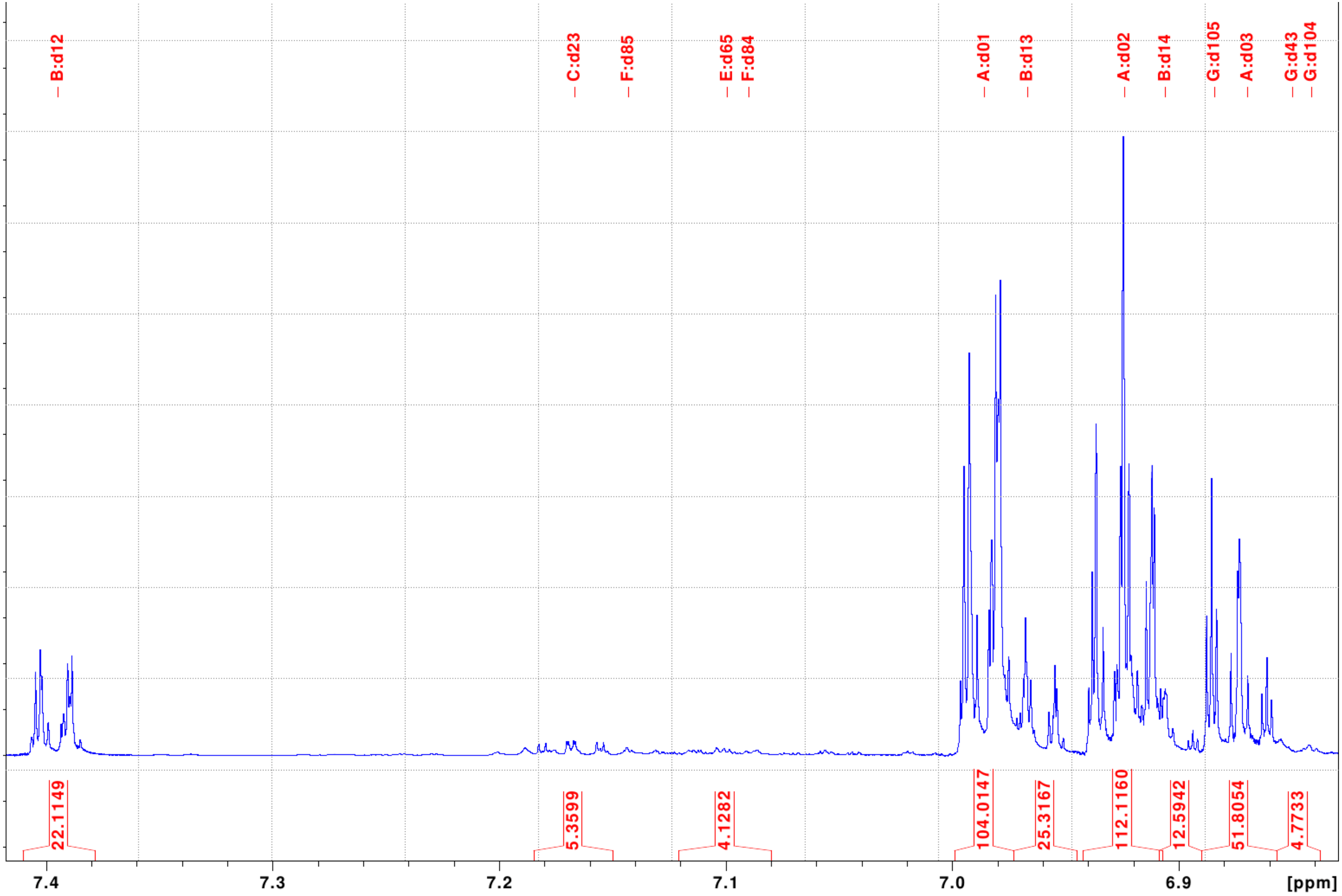
B:d12
C:d23
F:d85
E:d65
F:d84
A:d01
B:d13
A:d02
B:d14
G:d105
A:d03
G:d43
G:d104
22.1149
5.3599
4.1282
104.0147
25.3167
112.1160
12.5942
51.8054
4.7733
7.4
7.3
7.2
7.1
7.0
6.9
[ppm]

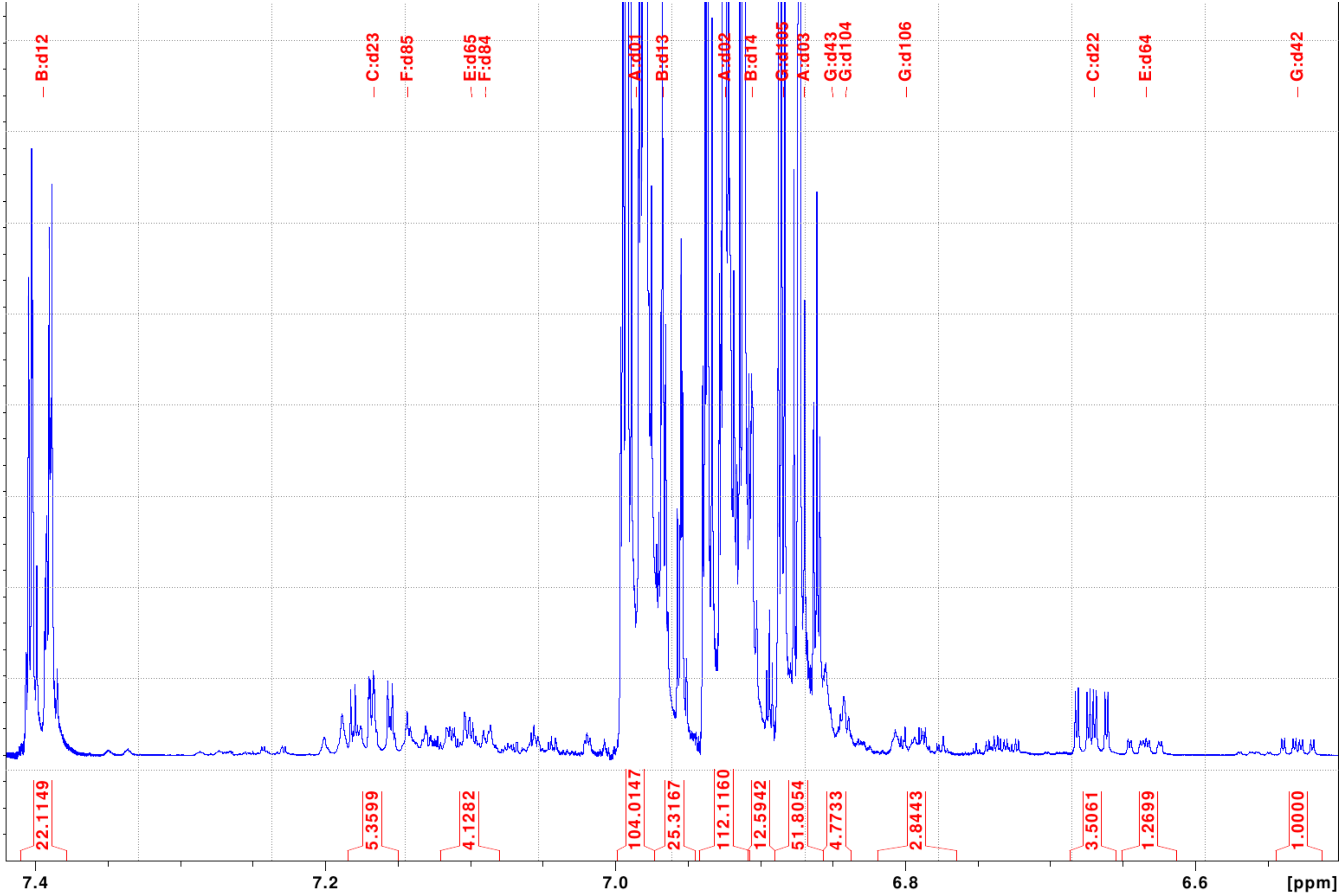

B:d12
C:d23
F:d85
E:d65
F:d84
A:d01
B:d13
A:d02
B:d14
G:d105
A:d03
G:d43
G:d104
G:d106
C:d22
E:d64
G:d42
22.1149
5.3599
4.1282
104.0147
25.3167
112.1160
12.5942
51.8054
4.7733
2.8443
3.5061
1.2699
1.0000
7.4
7.2
7.0
6.8
6.6
[ppm]

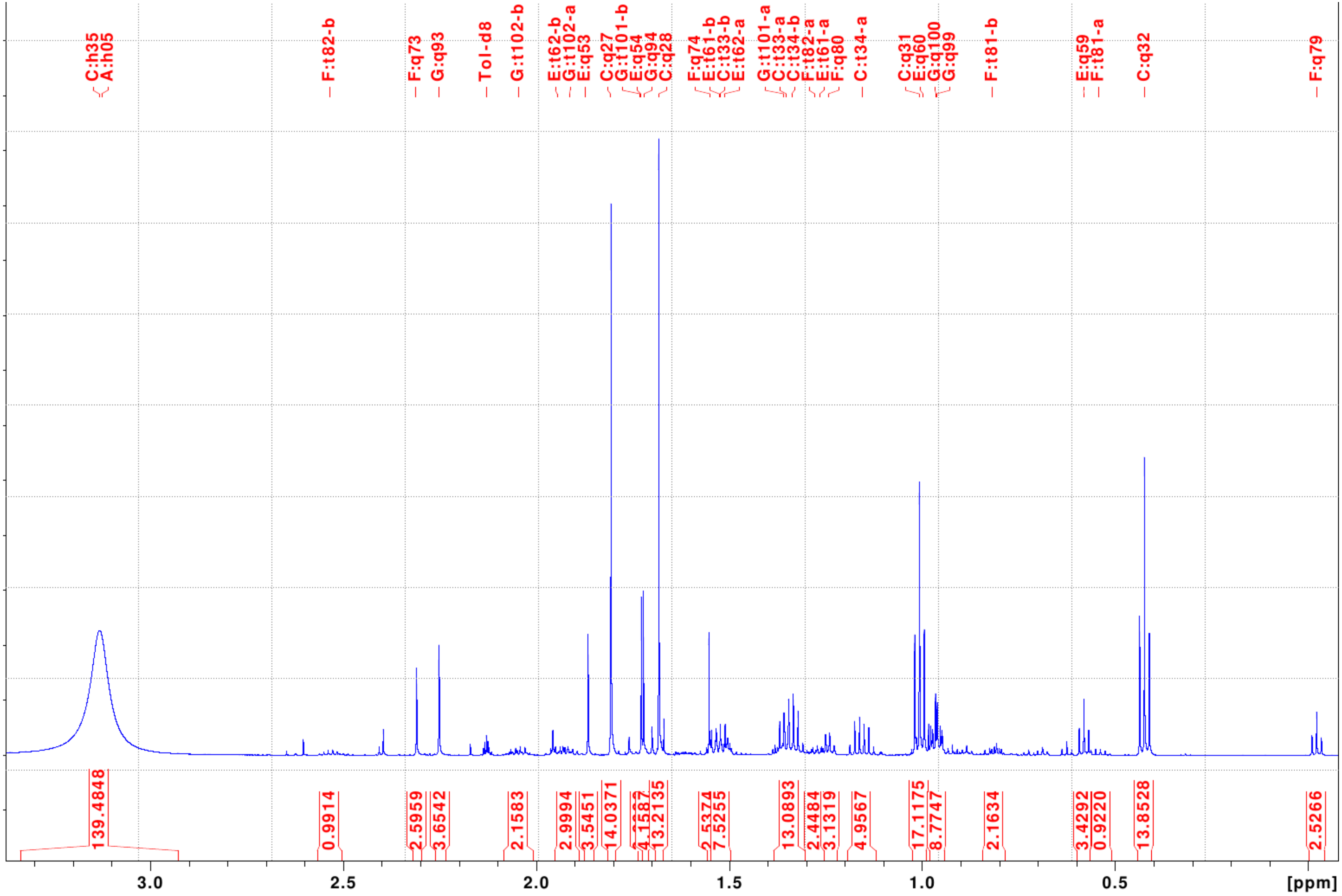

C:h35
A:h05
F:t82-b
F:q73
G:q93
Tol-d8
G:t102-b
E:t62-b
G:t102-a
E:q53
C:q27
G:t101-b
E:q54
G:q94
C:q28
F:q74
E:t61-b
C:t33-b
E:t62-a
G:t101-a
C:t33-a
C:t34-b
F:t82-a
E:t61-a
F:q80
C:t34-a
C:q31
E:q60
G:q100
G:q99
F:t81-b
E:q59
F:t81-a
C:q32
F:q79
139.4848
0.9914
2.5959
3.6542
2.1583
2.9994
3.5451
14.0371
4.1587
13.2135
2.5374
7.5255
13.0893
2.4484
3.1319
4.9567
17.1175
8.7747
2.1634
3.4292
0.9220
13.8528
2.5266
3.0
2.5
2.0
1.5
1.0
0.5
[ppm]

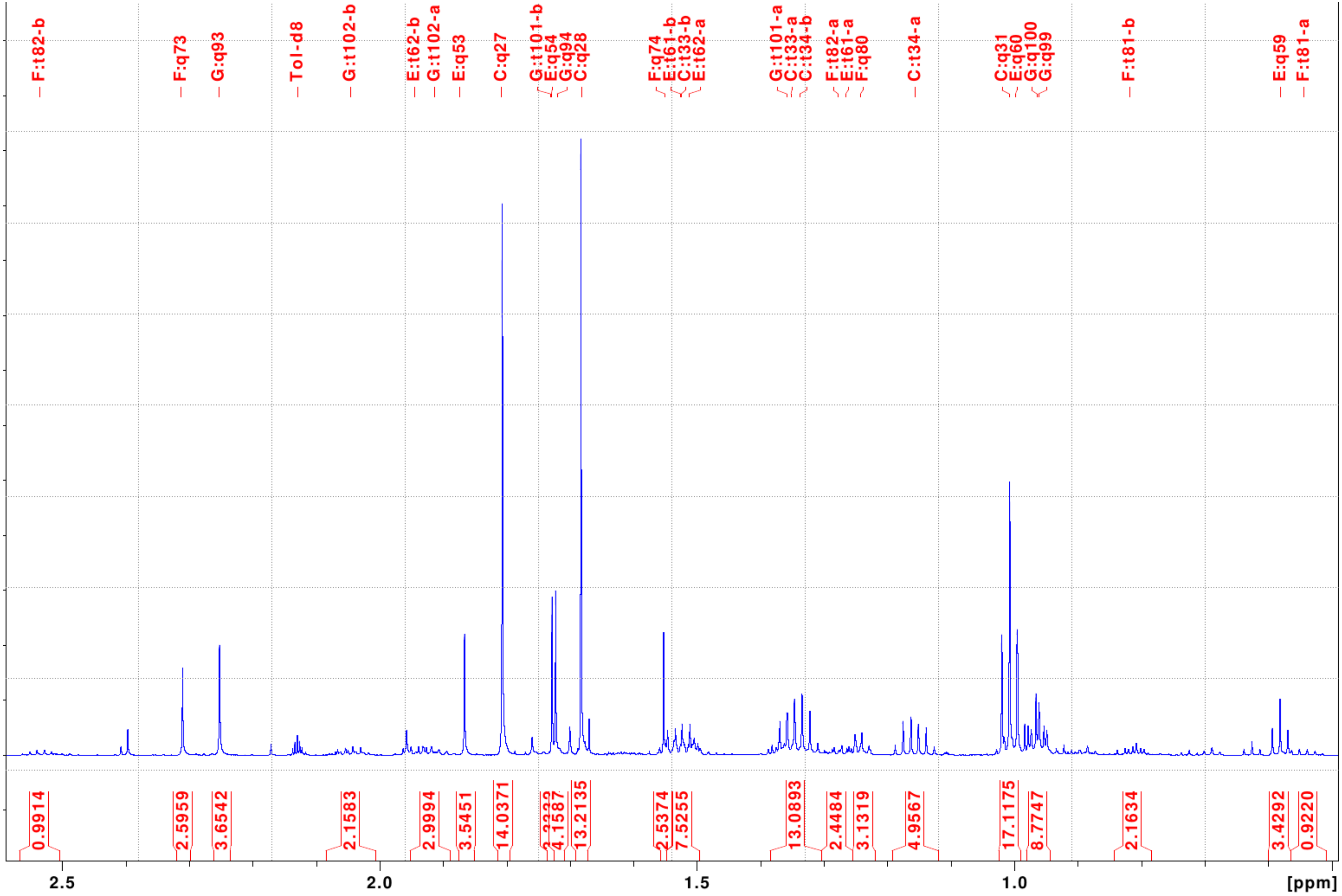

F:t82-b
F:q73
G:q93
Tol-d8
G:t102-b
E:t62-b
G:t102-a
E:q53
C:q27
G:t101-b
E:q54
G:q94
C:q28
F:q74
E:t61-b
C:t33-b
E:t62-a
G:t101-a
C:t33-a
C:t34-b
F:t82-a
E:t61-a
F:q80
C:t34-a
C:q31
E:q60
G:q100
G:q99
F:t81-b
E:q59
F:t81-a
0.9914
2.5959
3.6542
2.1583
2.9994
3.5451
14.0371
4.1587
13.2135
2.5374
7.5255
13.0893
2.4484
3.1319
4.9567
17.1175
8.7747
2.1634
3.4292
0.9220
2.5
2.0
1.5
1.0
[ppm]

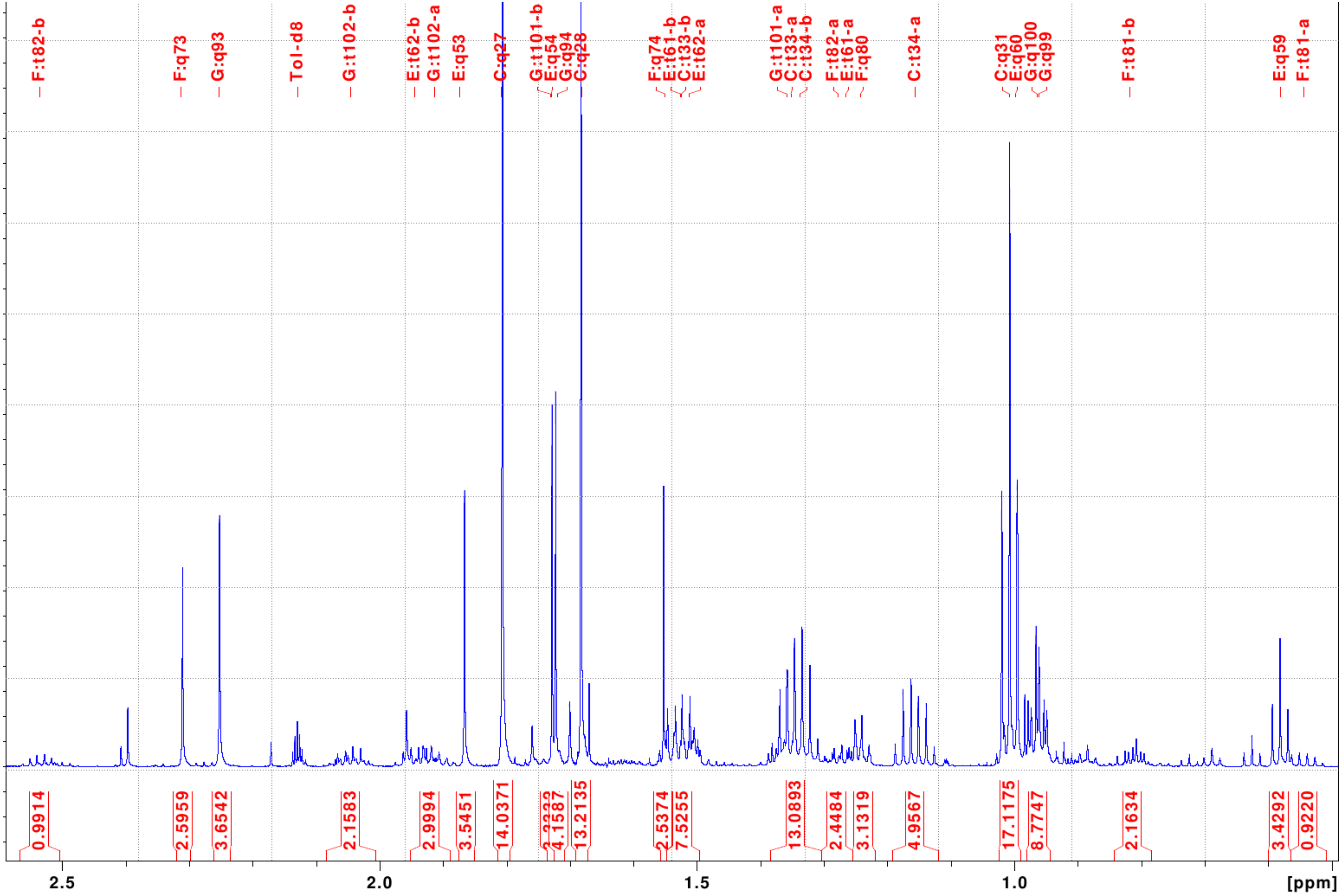
F:t82-b
F:q73
G:q93
Tol-d8
G:t102-b
E:t62-b
G:t102-a
E:q53
C:q27
G:t101-b
E:q54
G:q94
C:q28
F:q74
E:t61-b
C:t33-b
E:t62-a
G:t101-a
C:t33-a
C:t34-b
F:t82-a
E:t61-a
F:q80
C:t34-a
C:q31
E:q60
G:q100
G:q99
F:t81-b
E:q59
F:t81-a
0.9914
2.5959
3.6542
2.1583
2.9994
3.5451
14.0371
4.1587
13.2135
2.5374
7.5255
13.0893
2.4484
3.1319
4.9567
17.1175
8.7747
2.1634
3.4292
0.9220
2.5
2.0
1.5
1.0
[ppm]

^{15}N NMR spectrum

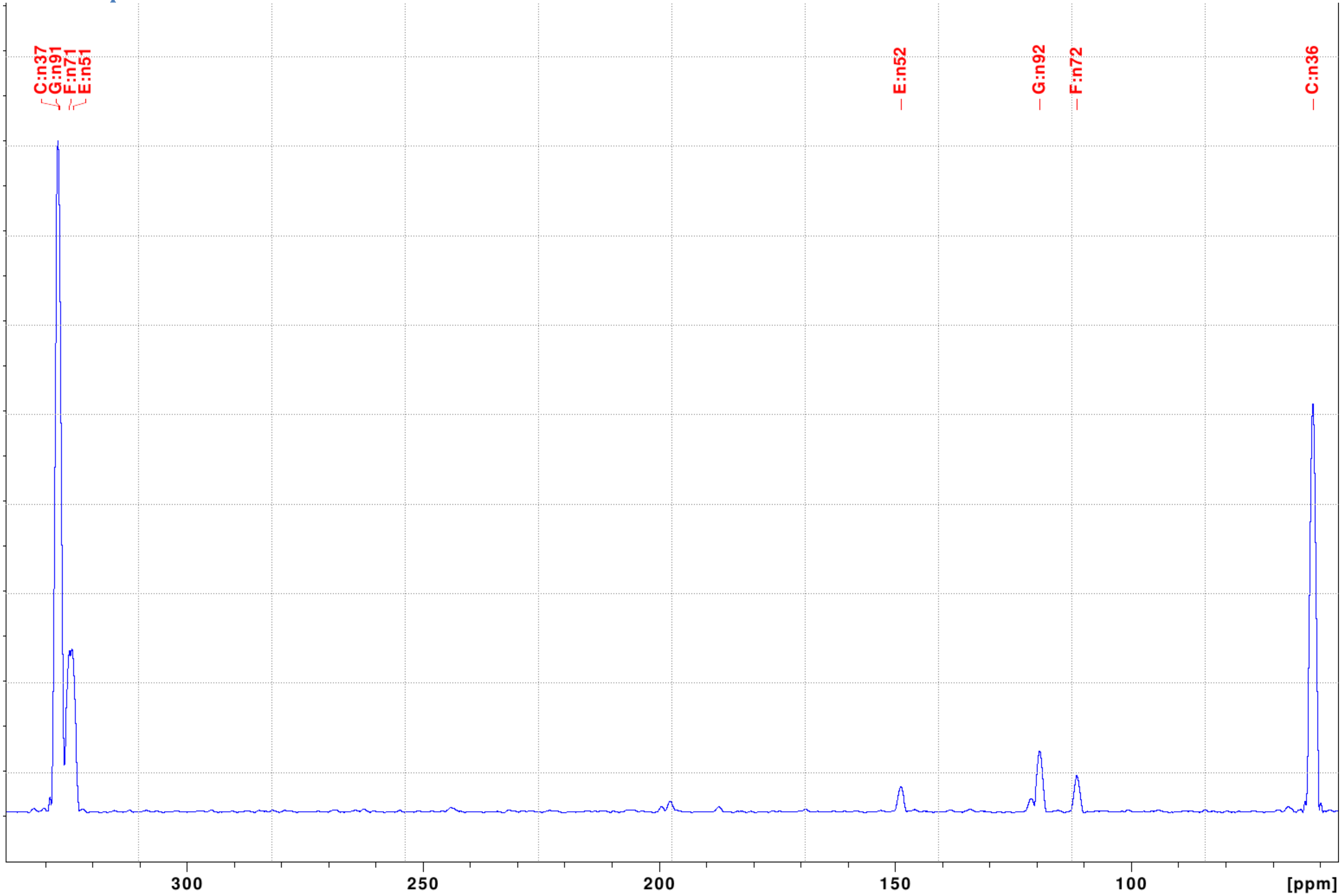

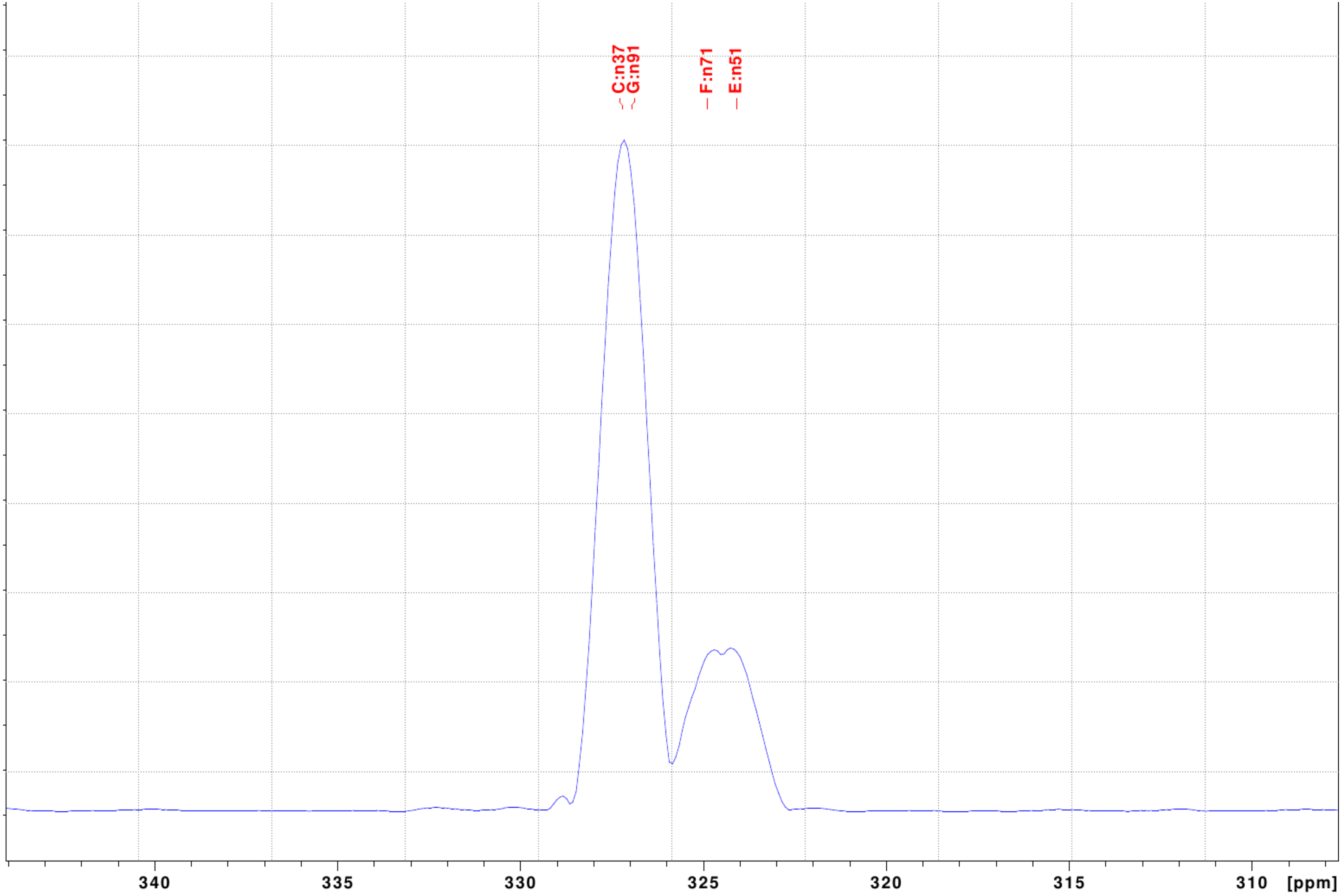
C:n37
G:n91
F:n71
E:n51
340
335
330
325
320
315
310
[ppm]

Structures and NMR signal assignments for products in the reaction mixture 2$^{RS/SR}$ + BME

in toluene-d_8 at 25 °C

Signal assignments

Some peak labels in NMR spectra could not be assigned to structures because of low product content.

Product A - trap dimer
HOCH2CH2SSCH2CH2OH
D = 1.135e-9 (V ~ 1.0)

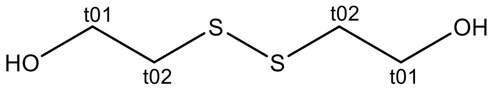

Experiment Bruker_290, 1D 13C
t01   60.2
t02   41.1

Experiment Bruker_291, 1D 1H
t01-a   3.87
t01-b   3.87
t02-a   2.85
t02-b   2.85

Experiment Bruker_298, 2D 13C-1H via onebond (HSQC)
t01-a - t01
t01-b - t01
t02-a - t02
t02-b - t02

Experiment Bruker_299, 2D 1H-13C via onebond (H-C correlation)
t01 - t01-a t01-b
t02 - t02-a t02-b

Experiment Bruker_293, 2D 1H-1H via Jcoupling (COSY)
t01-a - t02-a? t02-b?
t01-b - t02-a? t02-b?
t02-a - t01-a? t01-b?
t02-b - t01-a? t01-b?

Experiment Bruker_297, 2D 13C-1H via Jcoupling (HMBC)
t01-a - t02
t01-b - t02
t02-a - t01 t02(weak)
t02-b - t01 t02(weak)

Product B - trap trimer
HOCH2CH2SSCH2CH2SCH2CH2OH
D = 0.982e-9 (V ~ 1.5)

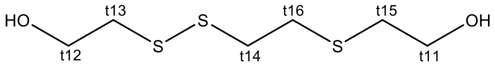

Experiment Bruker_290, 1D 13C
t11   60.7
t12   60.1
t13   41.4
t14   38.3
t15   35.1
t16   31.1

Experiment Bruker_291, 1D 1H
t11-a   3.73
t11-b   3.73
t12-a   3.85
t12-b   3.85
t13-a   2.85
t13-b   2.85
t14-a   2.86
t14-b   2.86
t15-a   2.73
t15-b   2.73
t16-a   2.86
t16-b   2.86

Experiment Bruker_298, 2D 13C-1H via onebond (HSQC)
t11-a - t11
t11-b - t11
t12-a - t12
t12-b - t12
t14-a - t14
t14-b - t14
t15-a - t15
t15-b - t15
t16-a - t16
t16-b - t16

Experiment Bruker_299, 2D 1H-13C via onebond (H-C correlation)
t11 - t11-a t11-b
t12 - t12-a t12-b
t13 - t13-a t13-b
t14 - t14-a t14-b
t15 - t15-a t15-b
t16 - t16-a t16-b

Experiment Bruker_293, 2D 1H-1H via Jcoupling (COSY)
t11-a - t15-a? t15-b?
t11-b - t15-a? t15-b?
t15-a - t11-a? t11-b?
t15-b - t11-a? t11-b?

Experiment Bruker_297, 2D 13C-1H via Jcoupling (HMBC)
t11-a - t15
t11-b - t15
t12-a - t13
t12-b - t13
t14-a - t16
t14-b - t16
t15-a - t11 t16
t15-b - t11 t16
t16-a - t14 t15
t16-b - t14 t15

The system has 2 distinct fragment(s)

Fragment 1:
t11
t14
t15
t16

Fragment 2:
t12
t13

Product C - amine
D = 0.885e-9 (V ~ 2.1)

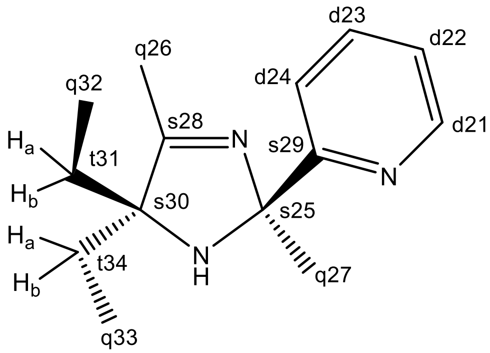

Experiment Bruker_290, 1D 13C
d21 147.8
d22 122.6
d23 137.1
d24 120.7
q26   15.2
q27   30.9
q32   8.1
q33   8.8
s25   92.4
s28 174.2
s29 162.1
s30   78.6
t31   29.8
t34   30.0

Experiment Bruker_291, 1D 1H
d21-H   8.49
d22-H   7.19
d23-H   7.69
d24-H   7.61
q26-H   1.91
q27-H   1.74
q32-H   0.56
q33-H   0.99
t31-a   1.45
t31-b   1.66
t34-a   1.62
t34-b   1.85

Experiment Bruker_298, 2D 13C-1H via onebond (HSQC)
d21-H - d21
d22-H - d22
d23-H - d23
d24-H - d24
q26-H - q26
q27-H - q27
q32-H - q32
q33-H - q33
t31-a - t31
t31-b - t31
t34-a - t34
t34-b - t34

Experiment Bruker_299, 2D 1H-13C via onebond (H-C correlation)
d21 - d21-H
d22 - d22-H
d23 - d23-H
d24 - d24-H
q26 - q26-H
q32 - q32-H
q33 - q33-H
t31 - t31-a t31-b

Experiment Bruker_293, 2D 1H-1H via Jcoupling (COSY)
d21-H - d22-H
d22-H - d21-H d23-H
d23-H - d22-H d24-H
d24-H - d23-H
q32-H - t31-a t31-b
q33-H - t34-a t34-b?
t31-a - q32-H t31-b
t31-b - q32-H t31-a
t34-a - q33-H t34-b?

Experiment Bruker_297, 2D 13C-1H via Jcoupling (HMBC)
d21-H - d22 d23 d24(weak) s29
d22-H - d21 d24
d23-H - d21 d24(weak) s29
d24-H - d21(weak) d22 s25
q26-H - q27(weak) s25(weak) s28 s29(weak) s30
q27-H - s25 s29
q32-H - s30 t31
q33-H - s30
t31-a - q32 s28 s30 t34
t34-a - q33 s30 t31

Experiment Bruker_296, 2D 1H-1H via through-space (NOESY)
d24-H - q27-H
q26-H - q32-H q33-H? t31-a t31-b? t34-a?
q27-H - d24-H q33-H
q32-H - q26-H
q33-H - q27-H
t31-a - q26-H
t31-b - t34-b?

Product D - trapped nitroxyl-OCH2CH2OH
D = 0.849e-9 (V ~ 2.4)

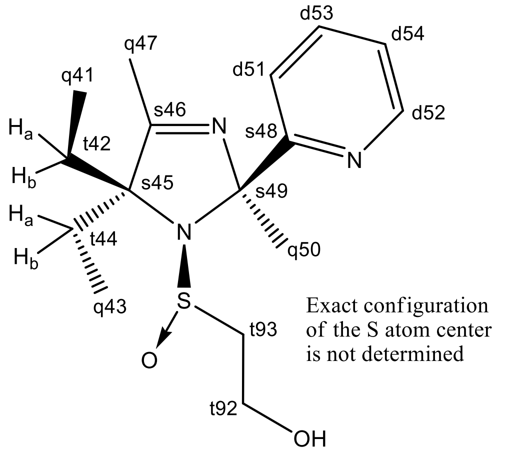

Exact configuration of the S atom center is not determined

Experiment Bruker_290, 1D 13C
d51 122.2
d52 148.5
d53 136.5
d54 123.4
q41   9.5
q43   9.7
q47   16.1
q50   27.7
s45   83.1
s46 171.3
s48 158.8
s49   94.6
t42   29.2
t44   30.3
t92   57.0
t93   57.8

Experiment Bruker_291, 1D 1H
d51-H   7.57
d52-H   8.60
d53-H   7.70
d54-H   7.23
q41-H   0.90
q43-H   0.95
q47-H   2.02
q50-H   2.10
t42-a   1.84
t42-b   2.15
t44-a   1.68
t44-b   2.24
t92-a   3.79
t92-b   3.79
t93-a   2.28
t93-b   2.73

Experiment Bruker_298, 2D 13C-1H via onebond (HSQC)
d51-H - d51
d52-H - d52
d53-H - d53
d54-H - d54
q41-H - q41
q43-H - q43
q47-H - q47
q50-H - q50
t42-a - t42
t42-b - t42
t44-b - t44
t92-a - t92
t92-b - t92
t93-a - t93
t93-b - t93

Experiment Bruker_299, 2D 1H-13C via onebond (H-C correlation)
d51 - d51-H
d52 - d52-H
d53 - d53-H
d54 - d54-H
q41 - q41-H
q43 - q43-H
q47 - q47-H
q50 - q50-H
t42 - t42-a t42-b
t44 - t44-a t44-b
t92 - t92-a t92-b
t93 - t93-a t93-b

Experiment Bruker_293, 2D 1H-1H via Jcoupling (COSY)

d51-H - d53-H
d52-H - d54-H
d53-H - d51-H d54-H
d54-H - d52-H d53-H
q41-H - t42-a t42-b
q43-H - t44-a t44-b
t42-a - q41-H t42-b
t42-b - q41-H t42-a
t44-a - q43-H t44-b
t44-b - q43-H t44-a
t92-a - t93-a t93-b
t92-b - t93-a t93-b
t93-a - t92-a t92-b t93-b
t93-b - t92-a t92-b t93-a

Experiment Bruker_297, 2D 13C-1H via Jcoupling (HMBC)
d51-H - d53 d54 s48 s49
d52-H - d53 d54 s48
d53-H - d51 d52 s48
d54-H - d51 d52 d53
q41-H - s45 t42
q43-H - s45 t44
q47-H - q50(weak) s45 s46 s48(weak) s49(weak)
q50-H - d51(weak) s48 s49
t42-a - q41 s45 s46 t44
t42-b - q41 s45 s46 t44
t44-a - q43 s46
t44-b - q43 s45 s46 t42
t92-a - t93
t92-b - t93
t93-a - t92
t93-b - t92

Experiment Bruker_296, 2D 1H-1H via through-space (NOESY)
d51-H - q41-H? q50-H
q41-H - q47-H t44-a? t93-b
q43-H - q50-H
q47-H - q41-H t42-a? t44-a
q50-H - d51-H q43-H
t42-b - t93-b
t44-a - q47-H t42-a?
t93-b - q41-H t42-b

Product E - heterocycle dimethylbenzimidazole
D = 1.072e-9 (V ~ 1.2)

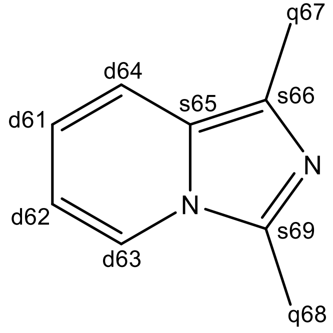

Experiment Bruker_290, 1D 13C
d61 116.9
d62 113.2
d63 120.2
d64 118.1
q67   11.4
q68   11.6
s65 126.2
s66 124.6
s69 132.6

Experiment Bruker_291, 1D 1H
d61-H   6.61
d62-H   6.55
d63-H   7.58
d64-H   7.31
q67-H   2.48
q68-H   2.65

Experiment Bruker_298, 2D 13C-1H via onebond (HSQC)
d61-H - d61
d62-H - d62
d63-H - d63
d64-H - d64
q67-H - q67
q68-H - q68

Experiment Bruker_299, 2D 1H-13C via onebond (H-C correlation)
d61 - d61-H
d62 - d62-H
d63 - d63-H
d64 - d64-H
q67 - q67-H
q68 - q68-H

Experiment Bruker_293, 2D 1H-1H via Jcoupling (COSY)
d61-H - d64-H
d62-H - d63-H
d63-H - d62-H
d64-H - d61-H

Experiment Bruker_297, 2D 13C-1H via Jcoupling (HMBC)
d61-H - d63 s65
d62-H - d63 d64
d63-H - d61 d62 d64(weak) s65 s69
d64-H - d62 d63(weak) s65
q67-H - d61(weak) d64(weak) s65 s66
q68-H - s66(weak) s69

Experiment Bruker_296, 2D 1H-1H via through-space (NOESY)
d63-H - q68-H
d64-H - q67-H
q67-H - d64-H
q68-H - d63-H

Product F - alkyl=O
D = 1.343e-9 (V ~ 0.6)

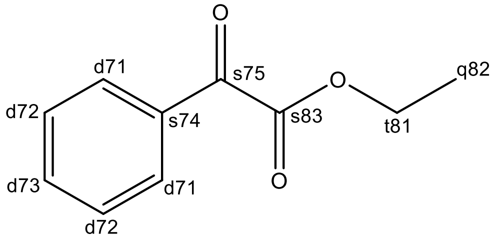

Experiment Bruker_290, 1D 13C
d71 129.9
d72 128.8
d73 134.8
q82   14.0
s74 132.3
s75 186.3
s83 163.7
t81   62.2

Experiment Bruker_291, 1D 1H
d71-H   7.97
d72-H   7.48
d73-H   7.63
q82-H   1.39
t81-a   4.42
t81-b   4.42

Experiment Bruker_298, 2D 13C-1H via onebond (HSQC)
d71-H - d71
d72-H - d72
d73-H - d73
q82-H - q82
t81-a - t81
t81-b - t81

Experiment Bruker_299, 2D 1H-13C via onebond (H-C correlation)
d71 - d71-H
d72 - d72-H
d73 - d73-H
q82 - q82-H
t81 - t81-a t81-b

Experiment Bruker_293, 2D 1H-1H via Jcoupling (COSY)
d71-H - d72-H
d72-H - d71-H d73-H
d73-H - d72-H
q82-H - t81-a t81-b
t81-a - q82-H
t81-b - q82-H

Experiment Bruker_297, 2D 13C-1H via Jcoupling (HMBC)
d71-H - d71 d73 s75
d72-H - d71(weak) d72 d73(weak) s74
d73-H - d71 d72
q82-H - t81
t81-a - q82 s83
t81-b - q82 s83

The system has 2 distinct fragment(s)

Fragment 1:
d71
d72
d73
s74
s75

Fragment 2:
t81
q82
s83

Product G - alkyl-OH (initially present in reagents)
D = 1.280e-9 (V ~ 0.7)

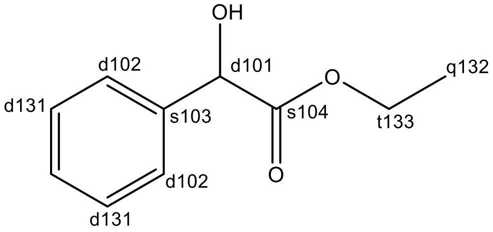

C_p, H_p not assigned

Experiment Bruker_290, 1D 13C
d101   72.8
d102 126.4
d131 128.4
q132   13.9
s103 138.3
s104 173.5
t133   62.1

Experiment Bruker_291, 1D 1H
d101-H   5.13
d102-H   7.39
d131-H   7.30
q132-H   1.19
t133-a   4.14
t133-b   4.14

Experiment Bruker_298, 2D 13C-1H via onebond (HSQC)
d101-H - d101
d102-H - d102
d131-H - d131
q132-H - q132
t133-a - t133
t133-b - t133

Experiment Bruker_299, 2D 1H-13C via onebond (H-C correlation)
d101 - d101-H
d102 - d102-H
d131 - d131-H
q132 - q132-H

Experiment Bruker_293, 2D 1H-1H via Jcoupling (COSY)
q132-H - t133-a t133-b
t133-a - q132-H
t133-b - q132-H

Experiment Bruker_297, 2D 13C-1H via Jcoupling (HMBC)
d101-H - d102 s103 s104
d102-H - d101 d102
q132-H - t133
t133-a - q132
t133-b - q132

The system has 3 distinct fragment(s)

Fragment 1:
d101
d102
s103
s104

Fragment 2:
d131

Fragment 3:
q132
t133

Product H - alkyl-H
D = 1.303e-9 (V ~ 0.66)

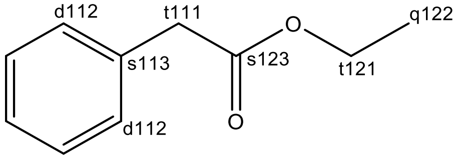

C_p, H_p, C_m, H_m not assigned

Experiment Bruker_290, 1D 13C
d112 129.1
q122   14.0
s113 134.0
s123 171.6
t111   41.3
t121   60.7

Experiment Bruker_291, 1D 1H
d112-H   7.25
q122-H   1.21
t111-a   3.58
t111-b   3.58
t121-a   4.11
t121-b   4.11

Experiment Bruker_298, 2D 13C-1H via onebond (HSQC)
d112-H - d112
q122-H - q122
t111-a - t111
t111-b - t111
t121-a - t121
t121-b - t121

Experiment Bruker_299, 2D 1H-13C via onebond (H-C correlation)
d112 - d112-H
q122 - q122-H
t111 - t111-a t111-b
t121 - t121-a t121-b

Experiment Bruker_293, 2D 1H-1H via Jcoupling (COSY)
q122-H - t121-a t121-b
t121-a - q122-H
t121-b - q122-H

Experiment Bruker_297, 2D 13C-1H via Jcoupling (HMBC)
q122-H - t121
t111-a - d112 s113 s123
t111-b - d112 s113 s123
t121-a - q122 s123
t121-b - q122 s123

Product K - nitroxyl-Ox
D = 0.849e-9 (V ~ 2.4)

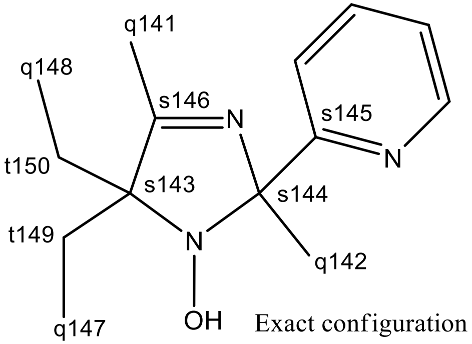

Exact configuration of the O atom center and spatial structure is not determined, C_{Py}, H_{Py} not assigned

Experiment Bruker_290, 1D 13C
q141 17.0
q142 24.7
q147 10.3
q148 8.5
s143 80.6
s144 94.1
s145 164.0
s146 176.5
t149 26.1
t150 29.5

Experiment Bruker_291, 1D 1H
q141-H 1.95
q142-H 1.70
q147-H 0.96
q148-H 0.53
t149-a 1.61
t149-b 1.98
t150-a 1.44
t150-b 1.97

Experiment Bruker_298, 2D 13C-1H via onebond (HSQC)
q141-H - q141
q142-H - q142
t149-a - t149
t149-b - t149
t150-a - t150
t150-b - t150

Experiment Bruker_299, 2D 1H-13C via onebond (H-C correlation)
q141 - q141-H
q142 - q142-H
q147 - q147-H
q148 - q148-H

Experiment Bruker_293, 2D 1H-1H via Jcoupling (COSY)
q147-H - t149-b?
q148-H - t150-a?
t149-a - t149-b
t149-b - t149-a

Experiment Bruker_297, 2D 13C-1H via Jcoupling (HMBC)
q141-H - q142 s143 s145 s146
q142-H - s144 s145
q147-H - s143 t149
q148-H - s143 t150
t149-a - q147 s143 s146
t149-b - q147
t150-a - s143

Experiment Bruker_296, 2D 1H-1H via through-space (NOESY)
q141-H - q147-H
q147-H - q141-H

$^{13}C\{^1H\}$ NMR spectrum (150 MHz)

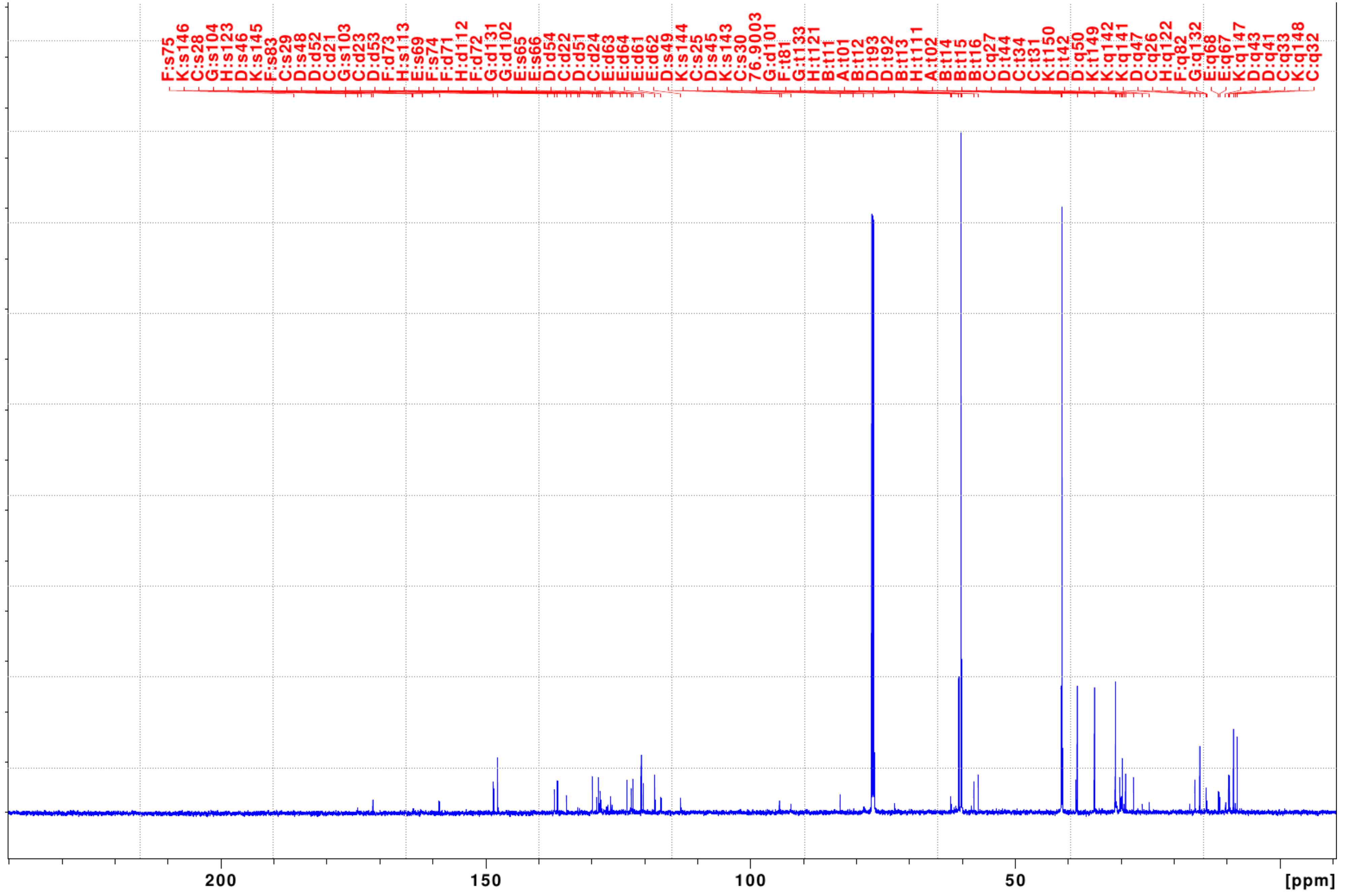

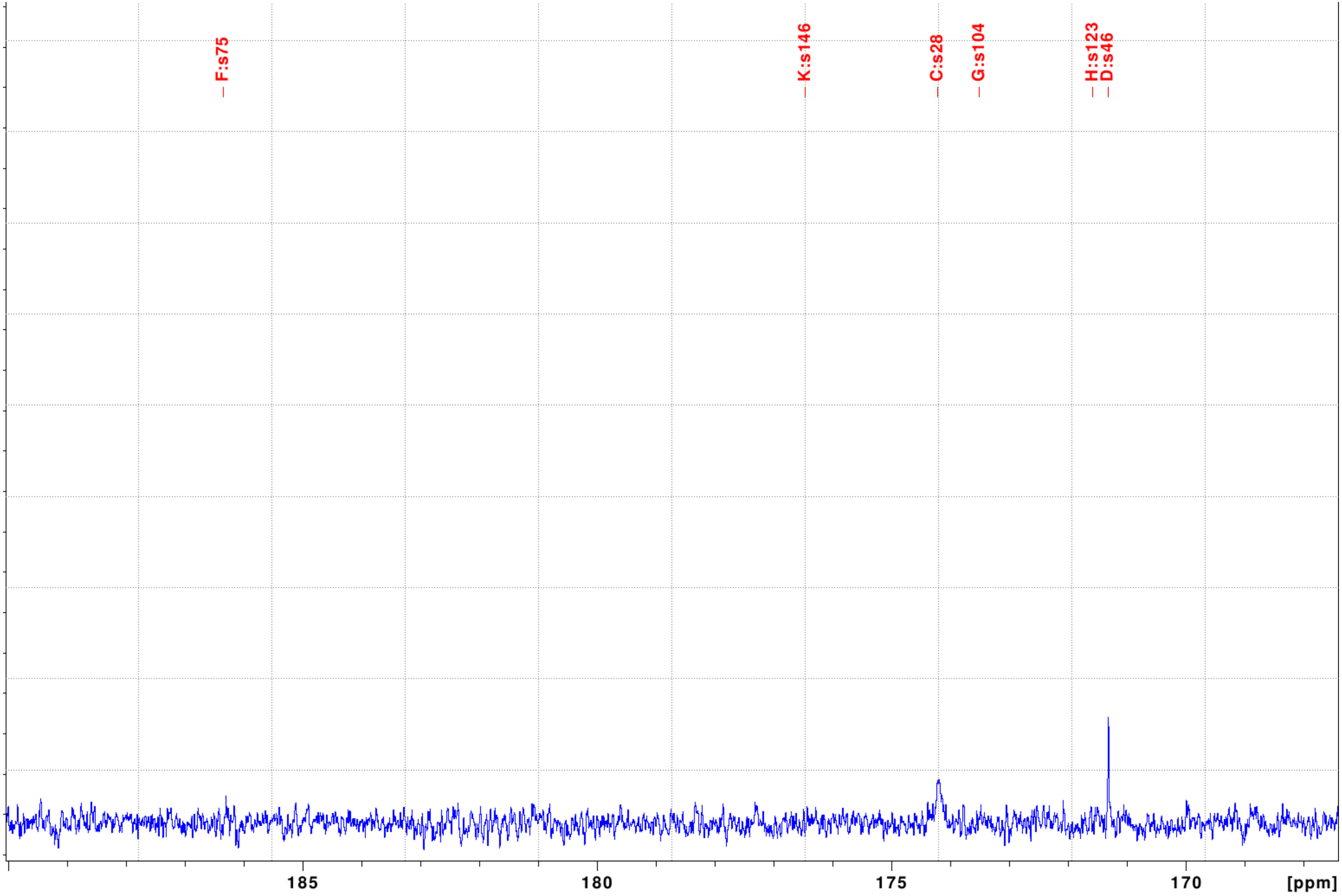
F:s75
K:s146
C:s28
G:s104
H:s123
D:s46
185
180
175
170
[ppm]

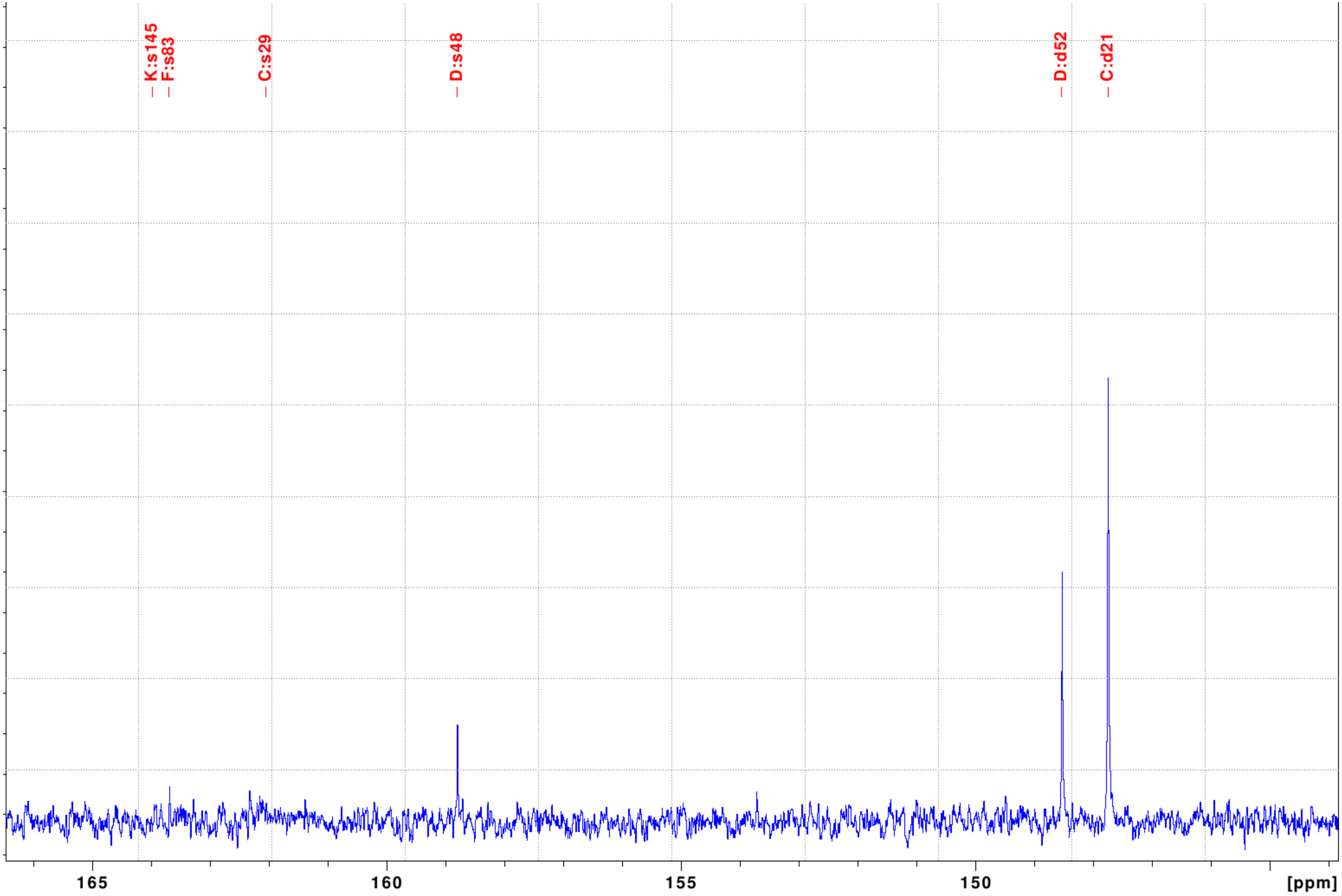
K:s145
F:s83
C:s29
D:s48
D:d52
C:d21
165
160
155
150
[ppm]

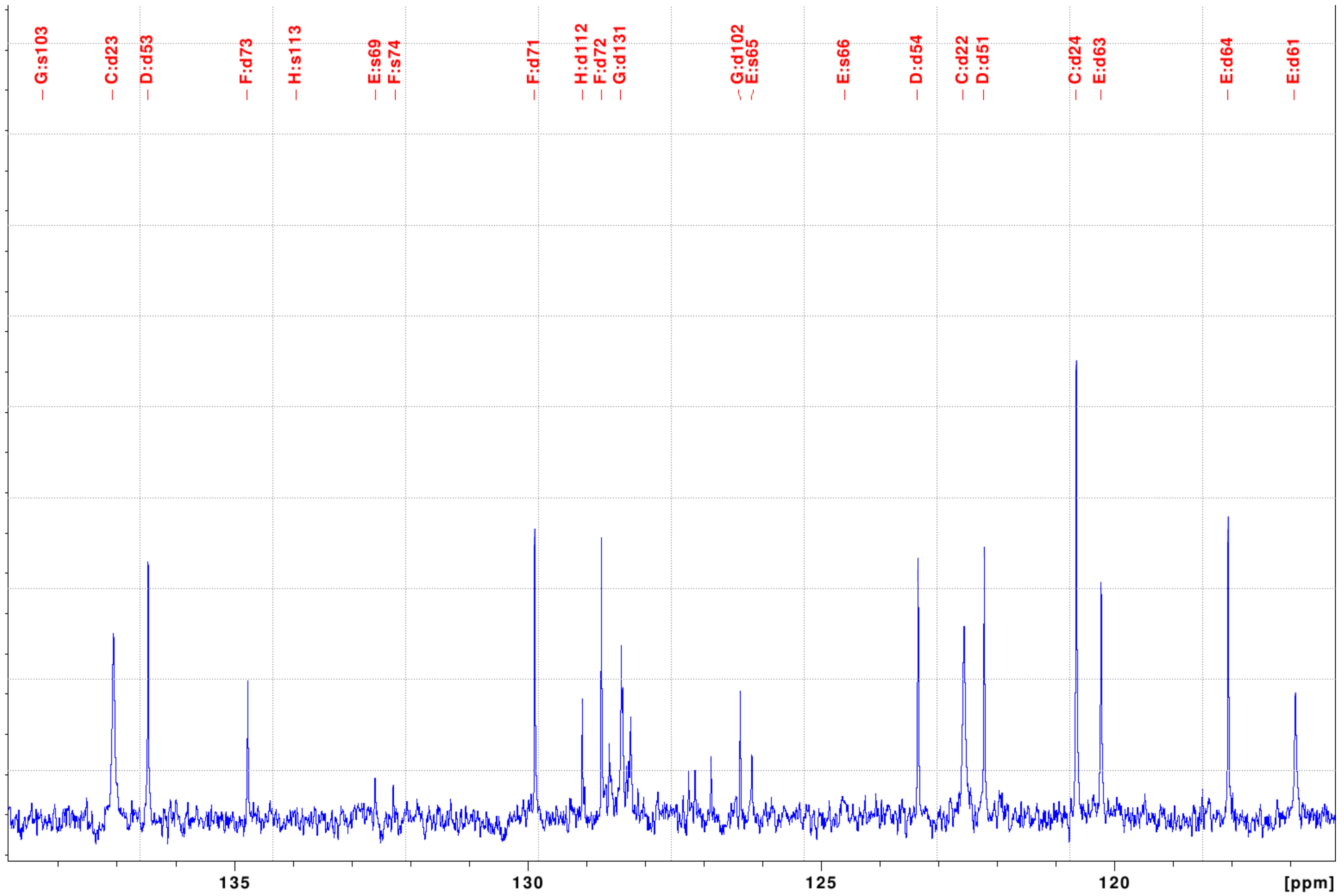
G:s103
C:d23
D:d53
F:d73
H:s113
E:s69
F:s74
F:d71
H:d112
F:d72
G:d131
G:d102
E:s65
E:s66
D:d54
C:d22
D:d51
C:d24
E:d63
E:d64
E:d61
135
130
125
120
[ppm]

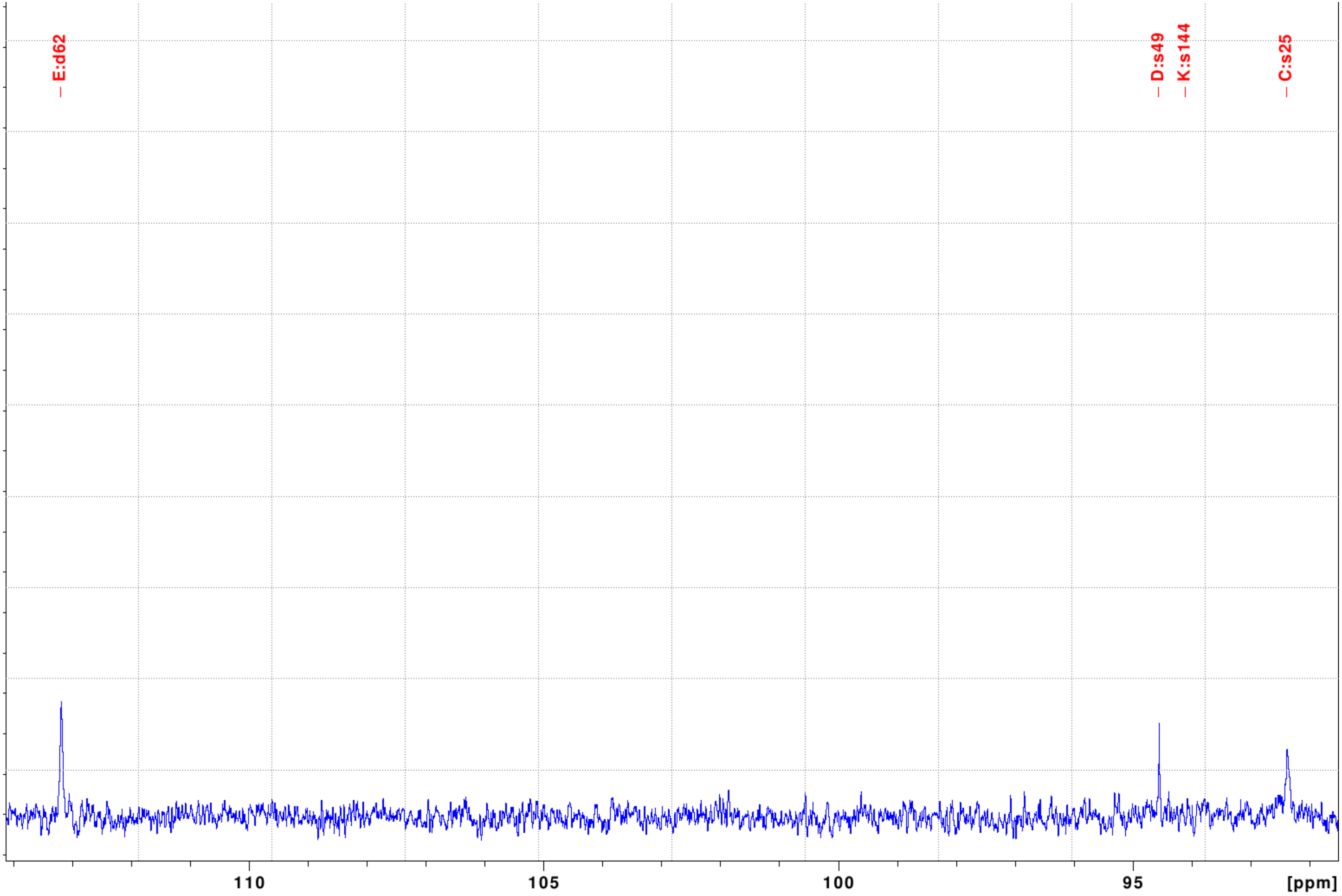

E:d62
D:s49
K:s144
C:s25
110
105
100
95
[ppm]

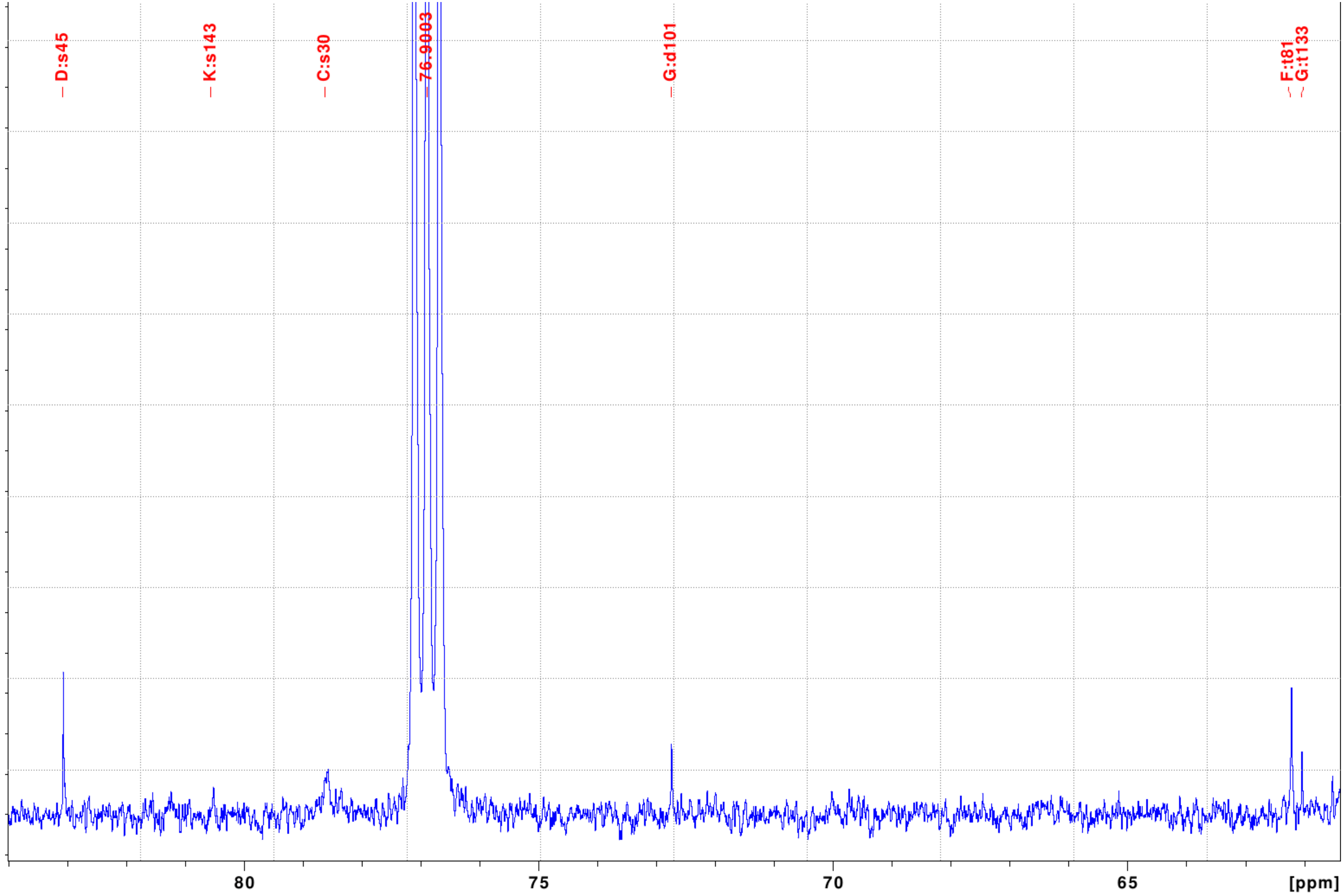
D:s45
K:s143
C:s30
76.9003
G:d101
F:t81
G:t133
80
75
70
65
[ppm]

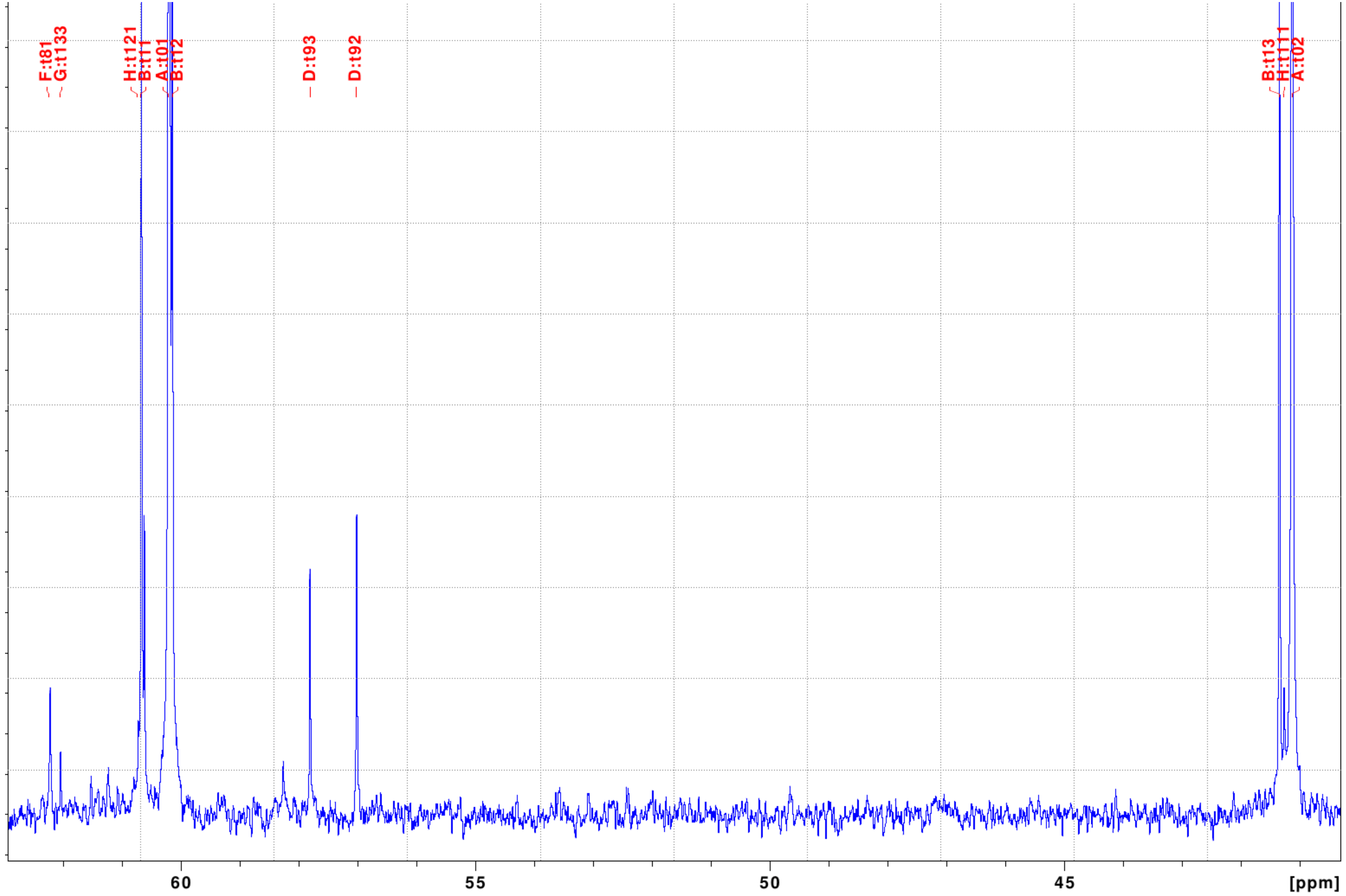
F:t81
G:t133
H:t121
B:t11
A:t01
B:t12
D:t93
D:t92
B:t13
H:t111
A:t02
60
55
50
45
[ppm]

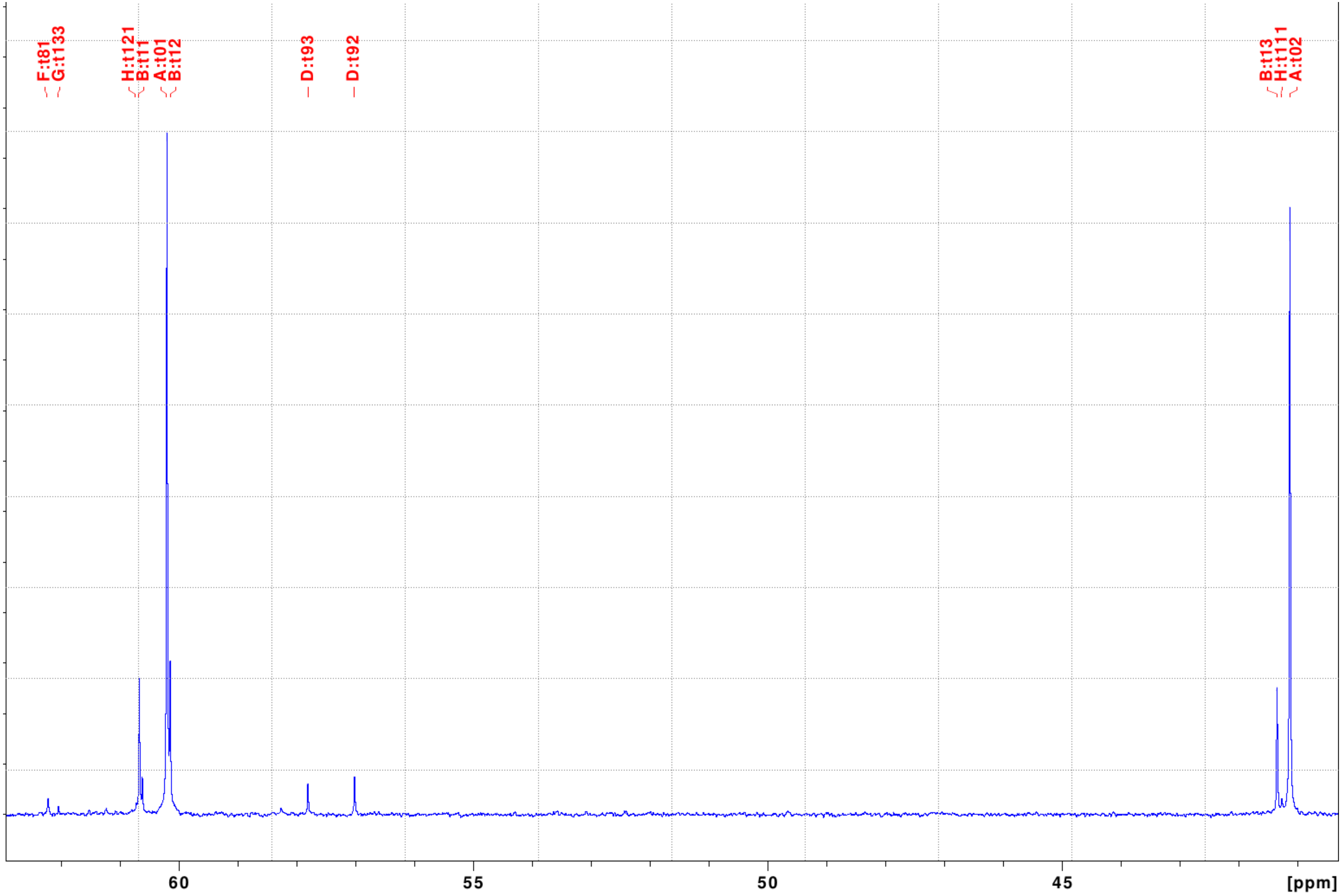

F:t81
G:t133
H:t121
B:t11
A:t01
B:t12
D:t93
D:t92
B:t13
H:t111
A:t02
60
55
50
45
[ppm]

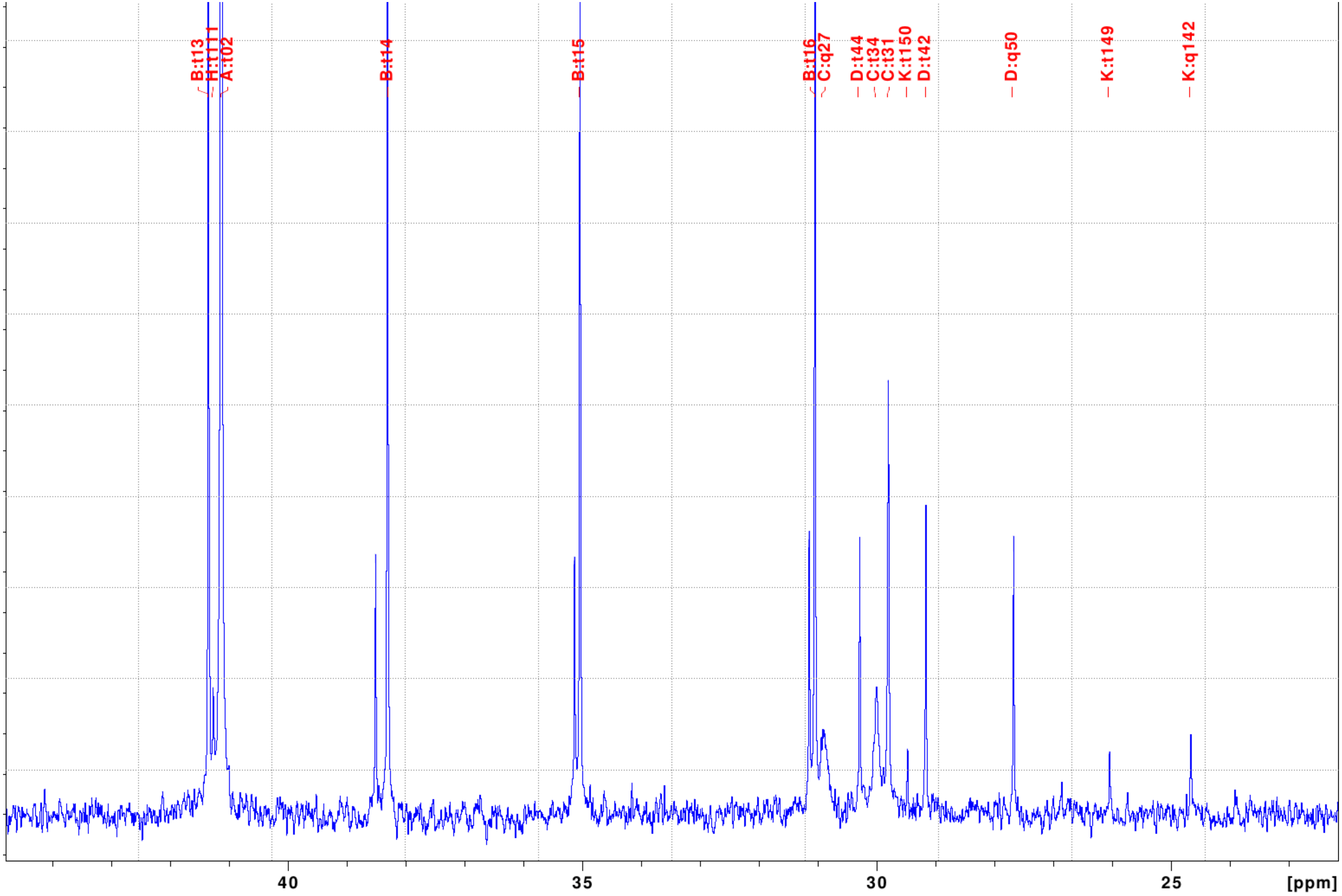
B:t13
H:t111
A:t102
B:t14
B:t15
B:t16
C:q27
D:t44
C:t34
C:t31
K:t150
D:t42
D:q50
K:t149
K:q142
40
35
30
25
[ppm]

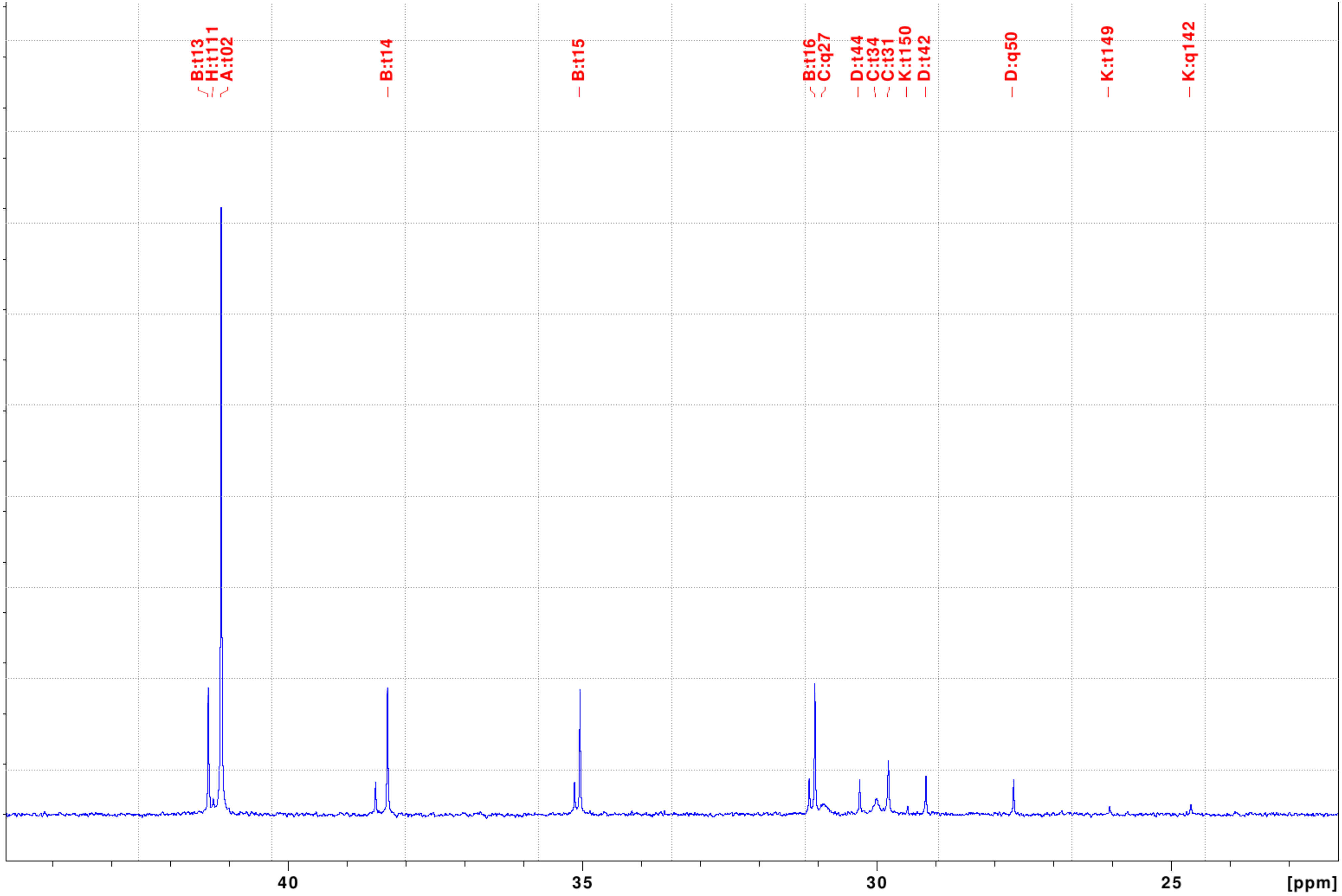
B:t13
H:t111
A:t02
B:t14
B:t15
B:t16
C:q27
D:t44
C:t34
C:t31
K:t150
D:t42
D:q50
K:t149
K:q142
40
35
30
25
[ppm]

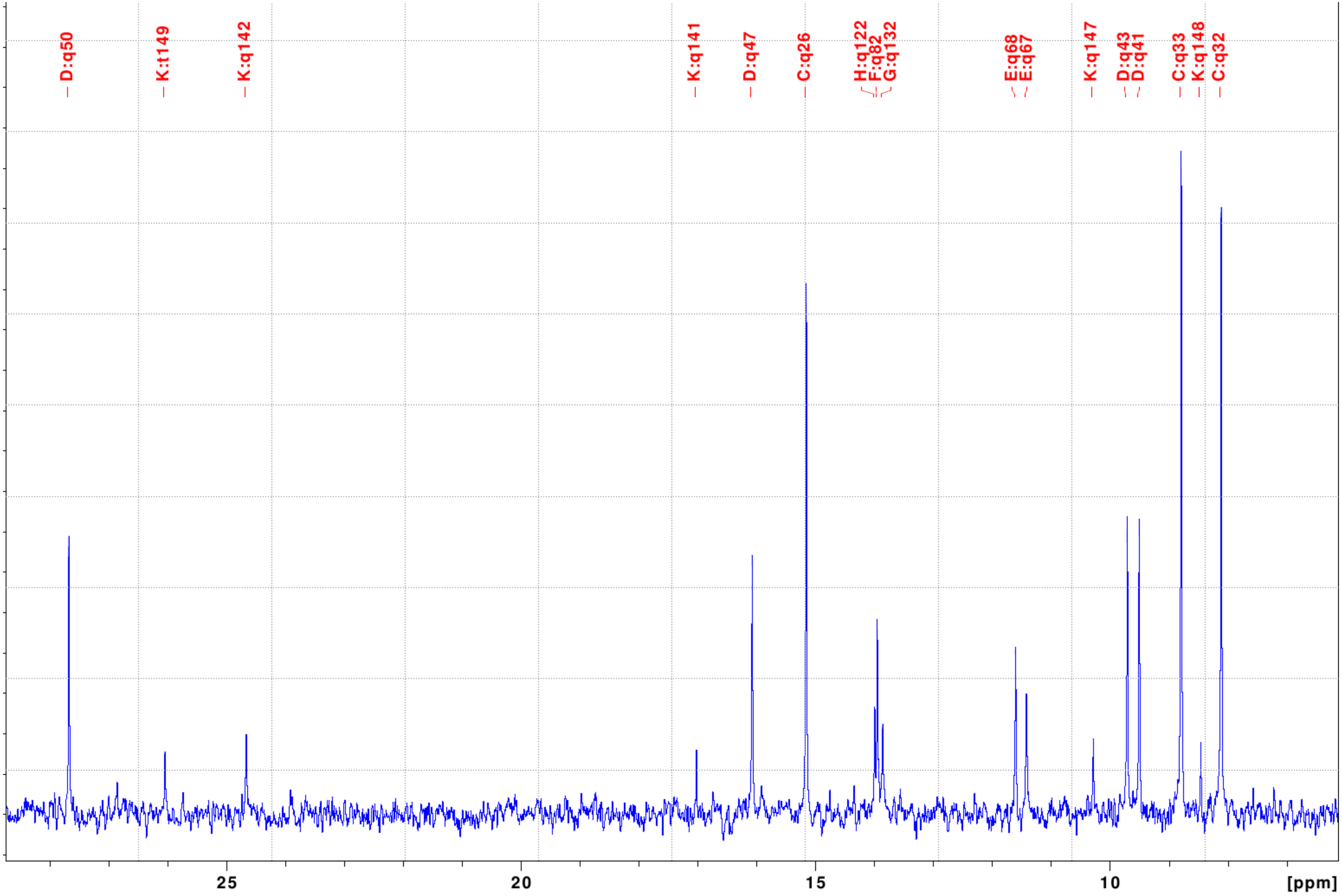
D:q50
K:t149
K:q142
K:q141
D:q47
C:q26
H:q122
F:q82
G:q132
E:q68
E:q67
K:q147
D:q43
D:q41
C:q33
K:q148
C:q32
25
20
15
10
[ppm]

^{1}H NMR spectrum (600 MHz)

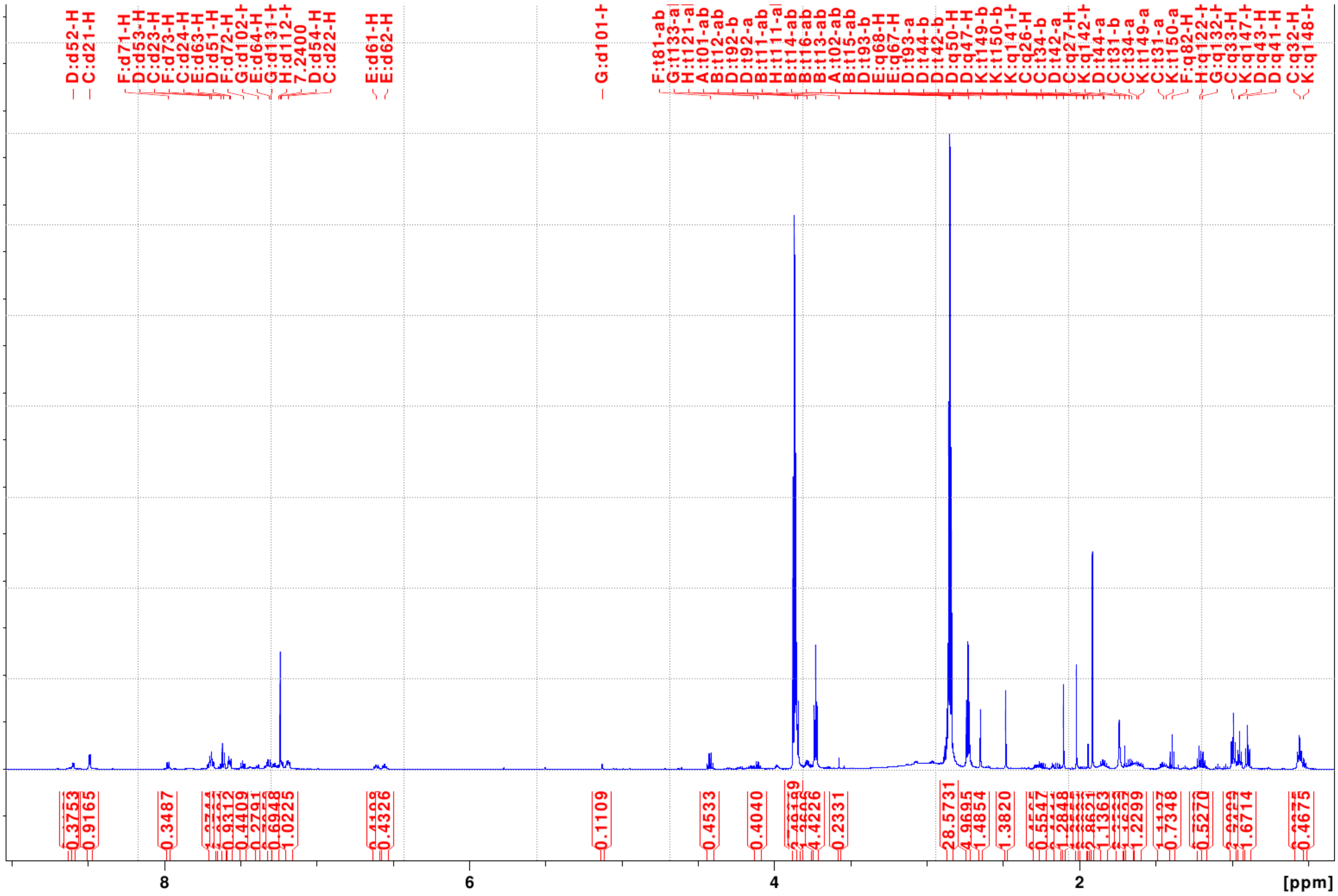

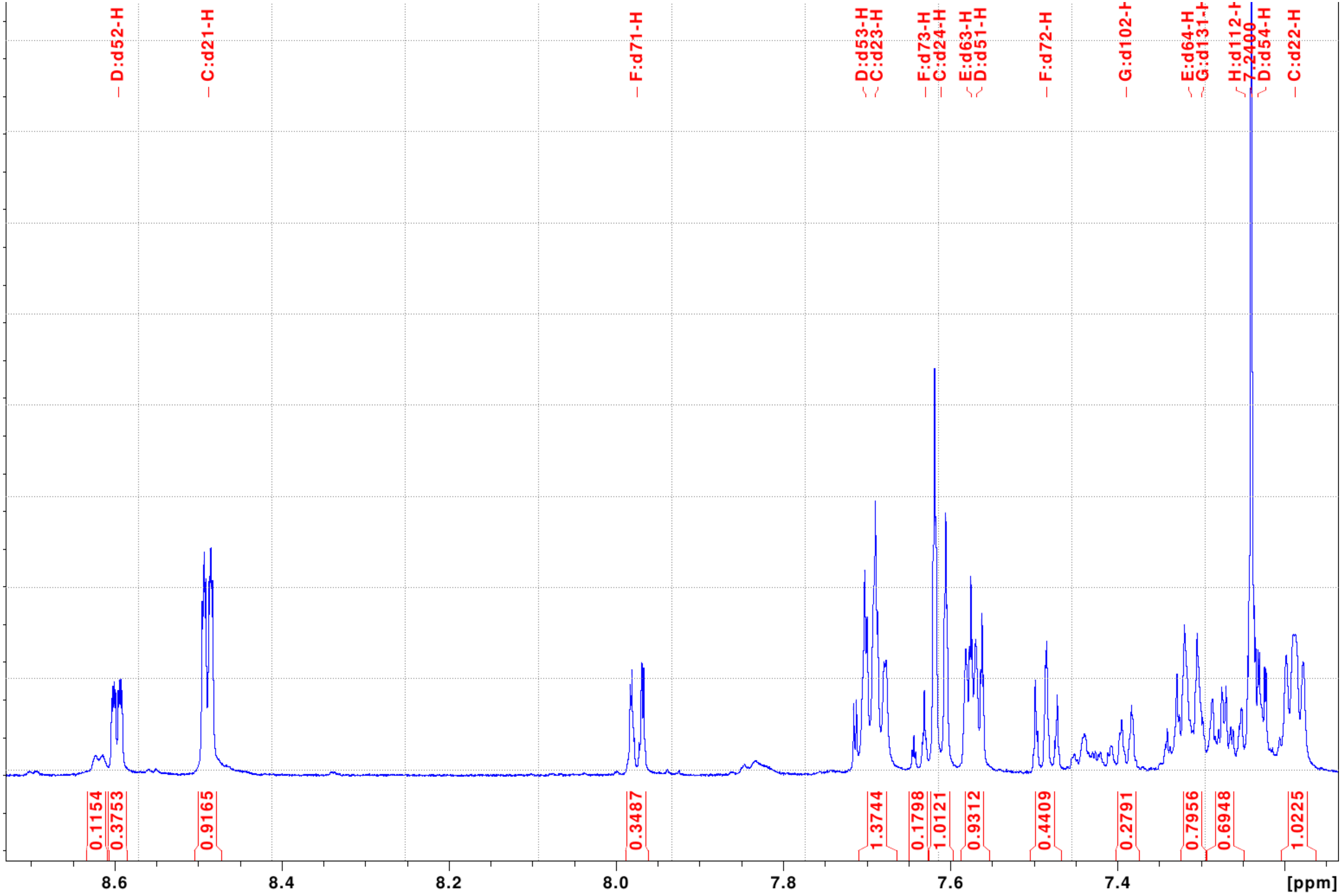

D:d52-H
C:d21-H
F:d71-H
D:d53-H
C:d23-H
F:d73-H
C:d24-H
E:d63-H
D:d51-H
F:d72-H
E:d64-H
D:d54-H
C:d22-H
7.2400
0.1154
0.3753
0.9165
0.3487
1.3744
0.1798
1.0121
0.9312
0.4409
0.2791
0.7956
0.6948
1.0225
8.6
8.4
8.2
8.0
7.8
7.6
7.4
[ppm]

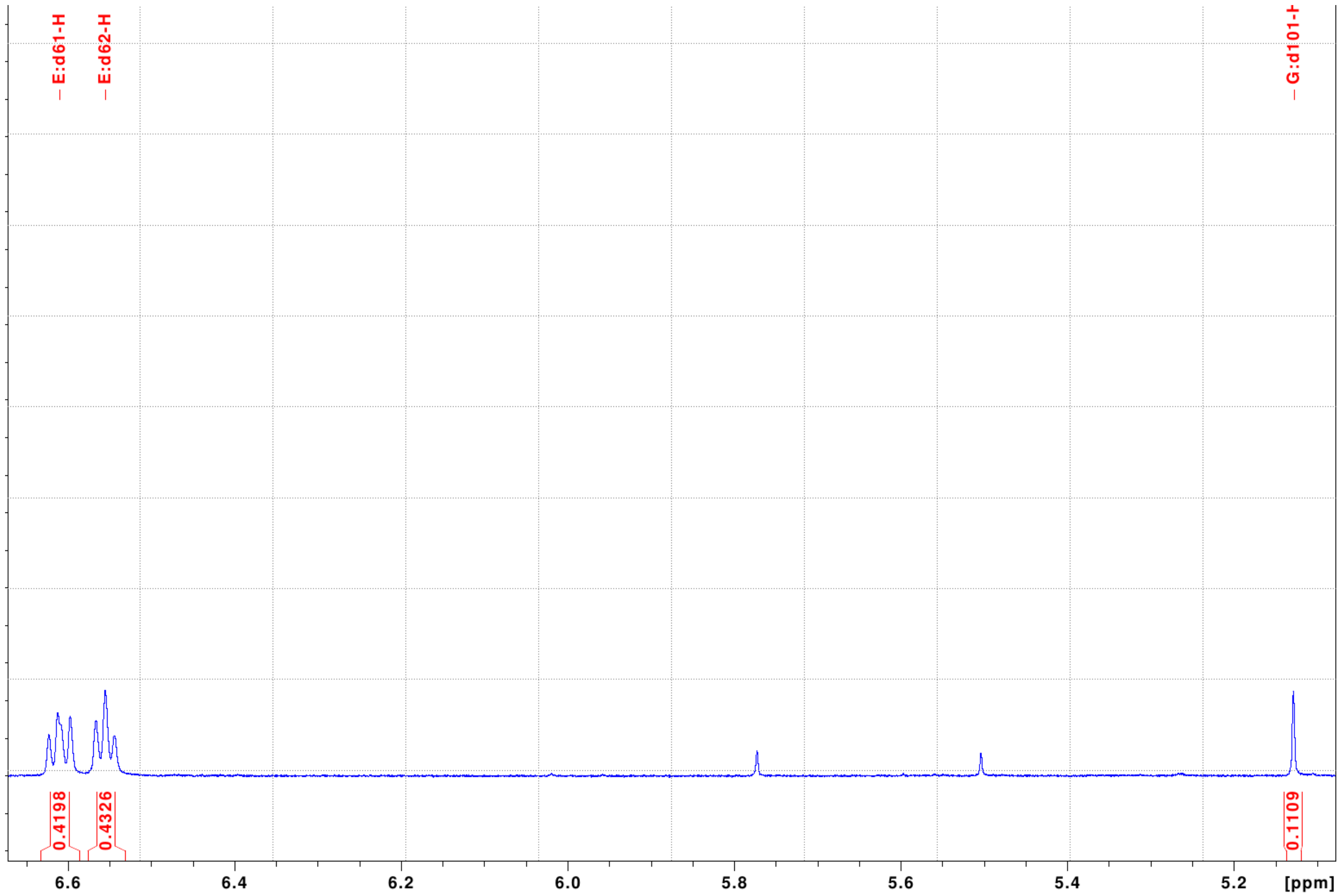

E:d61-H
E:d62-H
G:d101-H
0.4198
0.4326
0.1109
6.6
6.4
6.2
6.0
5.8
5.6
5.4
5.2
[ppm]

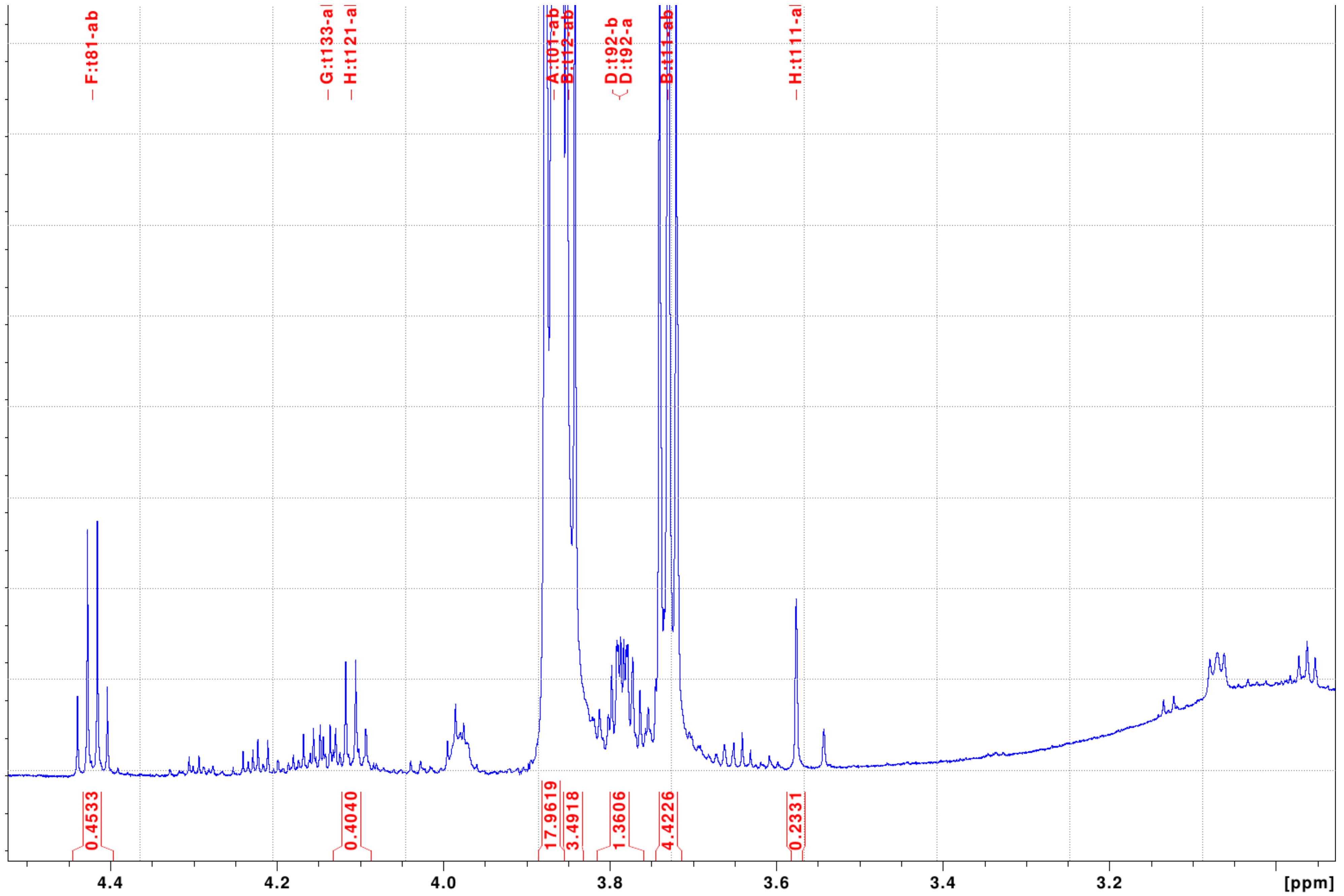
F:t81-ab
G:t133-al
H:t121-al
A:t01-ab
B:t12-ab
D:t92-b
D:t92-a
B:t11-ab
H:t111-al
0.4533
0.4040
17.9619
3.4918
1.3606
4.4226
0.2331
4.4
4.2
4.0
3.8
3.6
3.4
3.2
[ppm]

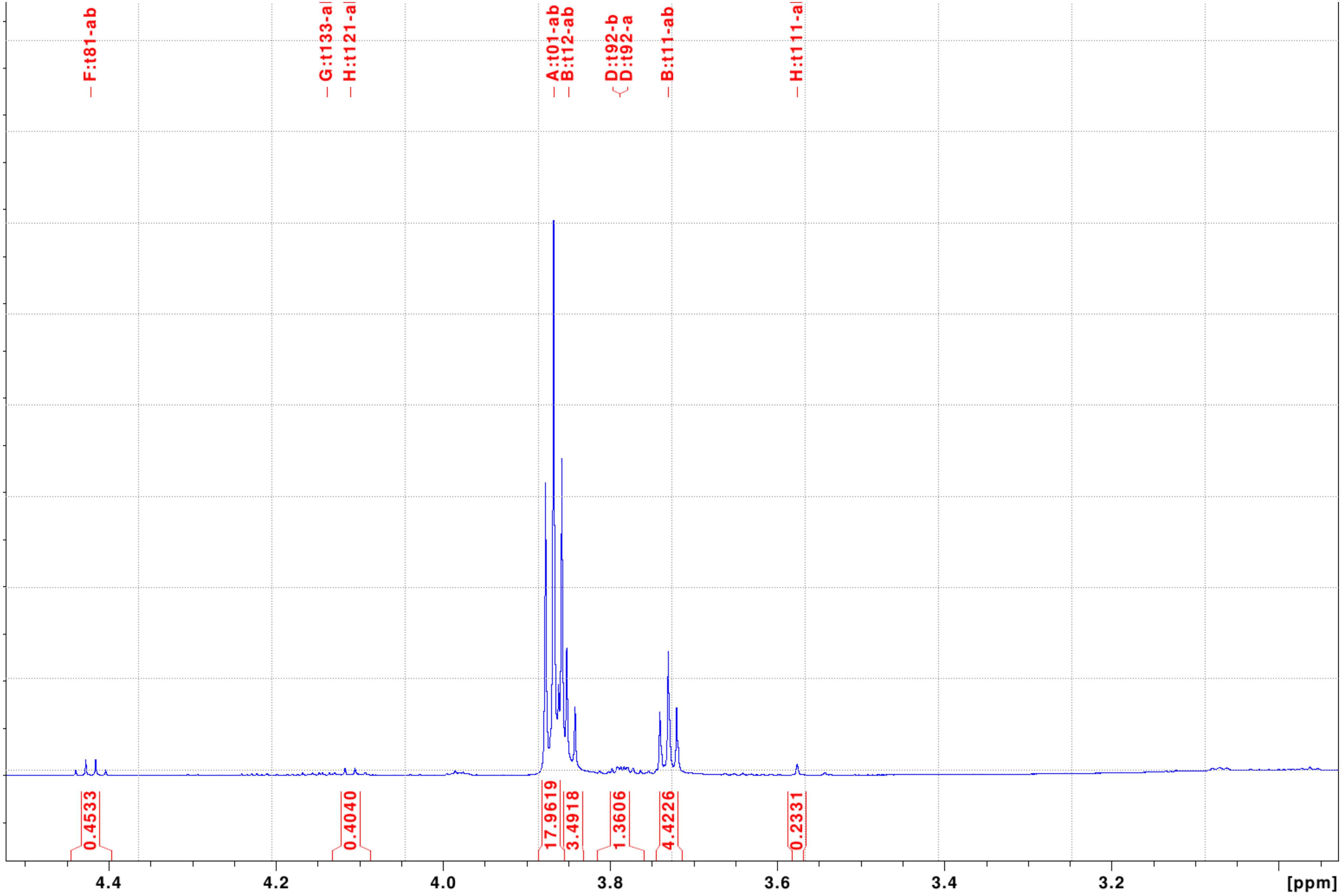
F:t81-ab
G:t133-al
H:t121-al
A:t01-ab
B:t12-ab
D:t92-b
D:t92-a
B:t11-ab
H:t111-al
0.4533
0.4040
17.9619
3.4918
1.3606
4.4226
0.2331
4.4
4.2
4.0
3.8
3.6
3.4
3.2
[ppm]

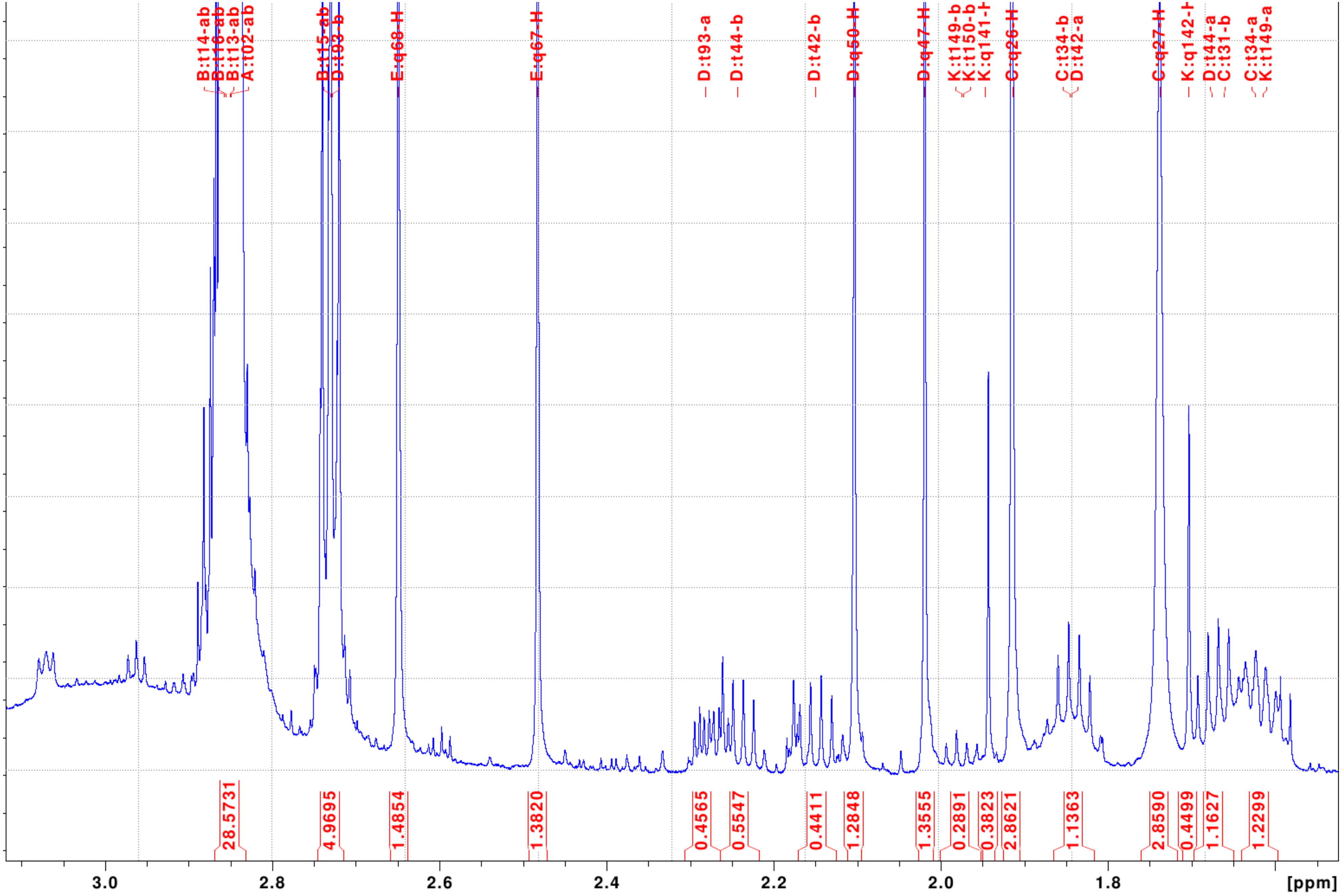
B:t14-ab
B:t16-ab
B:t13-ab
A:t02-ab
B:t15-ab
D:t93-b
E:q68-H
E:q67-H
D:t93-a
D:t44-b
D:t42-b
D:q50-H
D:q47-H
K:t149-b
K:t150-b
K:q141-H
C:q26-H
C:t34-b
D:t42-a
C:q27-H
K:q142-H
D:t44-a
C:t31-b
C:t34-a
K:t149-a
28.5731
4.9695
1.4854
1.3820
0.4565
0.5547
0.4411
1.2848
1.3555
0.2891
0.3823
2.8621
1.1363
2.8590
0.4499
1.1627
1.2299
3.0
2.8
2.6
2.4
2.2
2.0
1.8
[ppm]

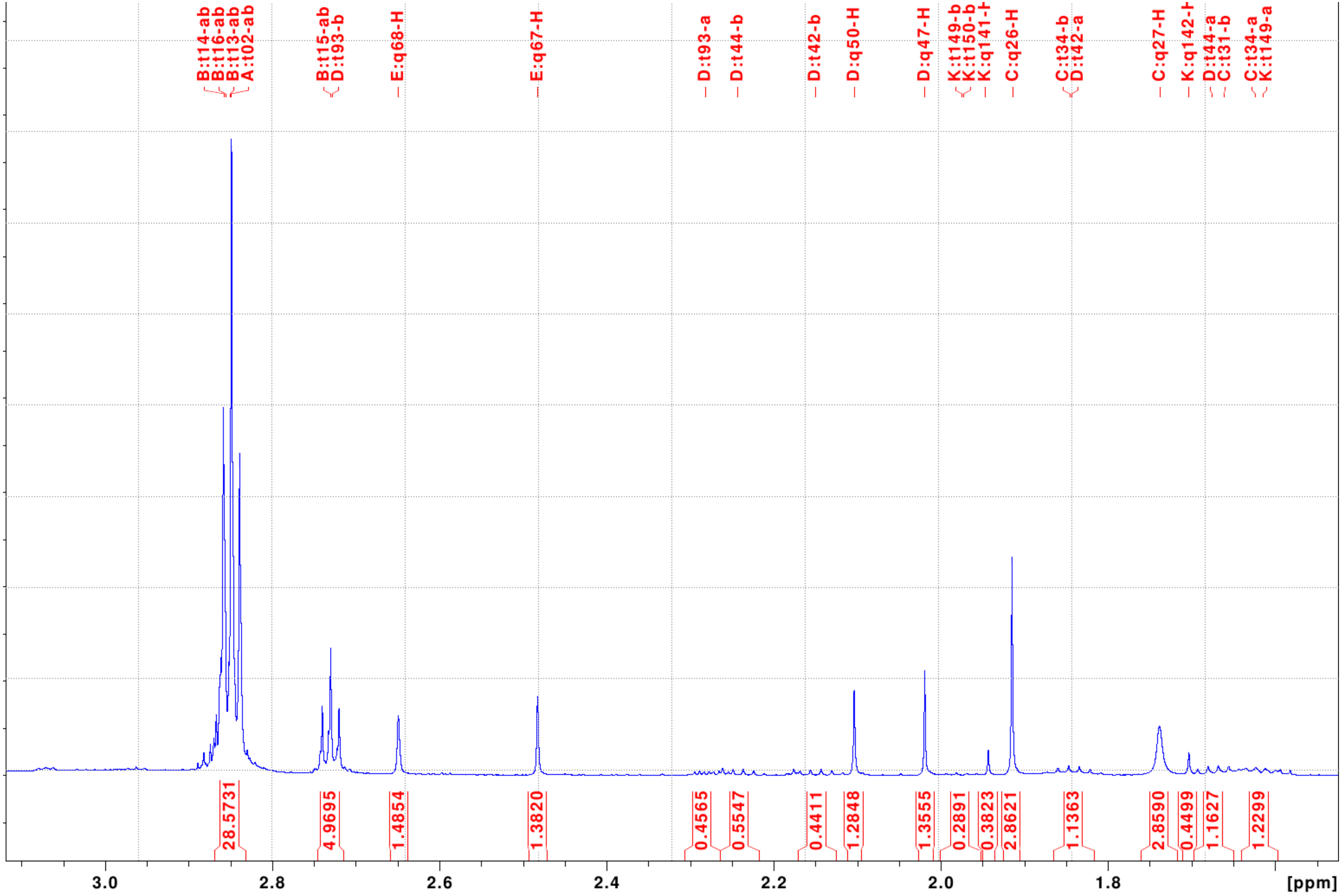

B:t14-ab
B:t16-ab
B:t13-ab
A:t02-ab
B:t15-ab
D:t93-b
E:q68-H
E:q67-H
D:t93-a
D:t44-b
D:t42-b
D:q50-H
D:q47-H
K:t149-b
K:t150-b
K:q141-H
C:q26-H
C:t34-b
D:t42-a
C:q27-H
K:q142-H
D:t44-a
C:t31-b
C:t34-a
K:t149-a
28.5731
4.9695
1.4854
1.3820
0.4565
0.5547
0.4411
1.2848
1.3555
0.2891
0.3823
2.8621
1.1363
2.8590
0.4499
1.1627
1.2299
3.0
2.8
2.6
2.4
2.2
2.0
1.8
[ppm]

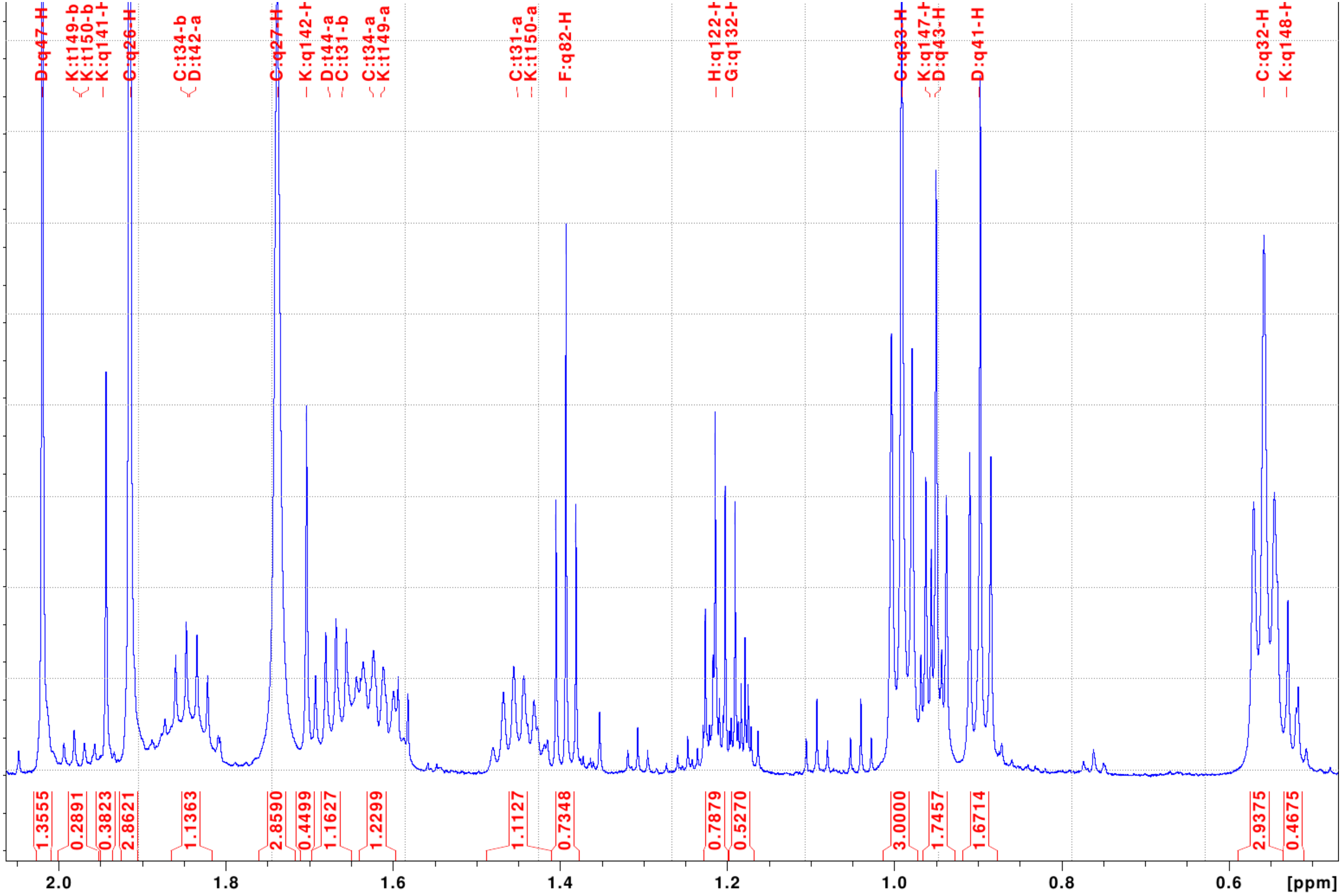
D:q47-H
K:t149-b
K:t150-b
K:q141-F
C:q26-H
C:t34-b
D:t42-a
C:q27-H
K:q142-F
D:t44-a
C:t31-b
C:t34-a
K:t149-a
C:t31-a
K:t150-a
F:q82-H
H:q122-F
G:q132-F
C:q33-H
K:q147-F
D:q43-H
D:q41-H
C:q32-H
K:q148-F
1.3555
0.2891
0.3823
2.8621
1.1363
2.8590
0.4499
1.1627
1.2299
1.1127
0.7348
0.7879
0.5270
3.0000
1.7457
1.6714
2.9375
0.4675
2.0
1.8
1.6
1.4
1.2
1.0
0.8
0.6
[ppm]

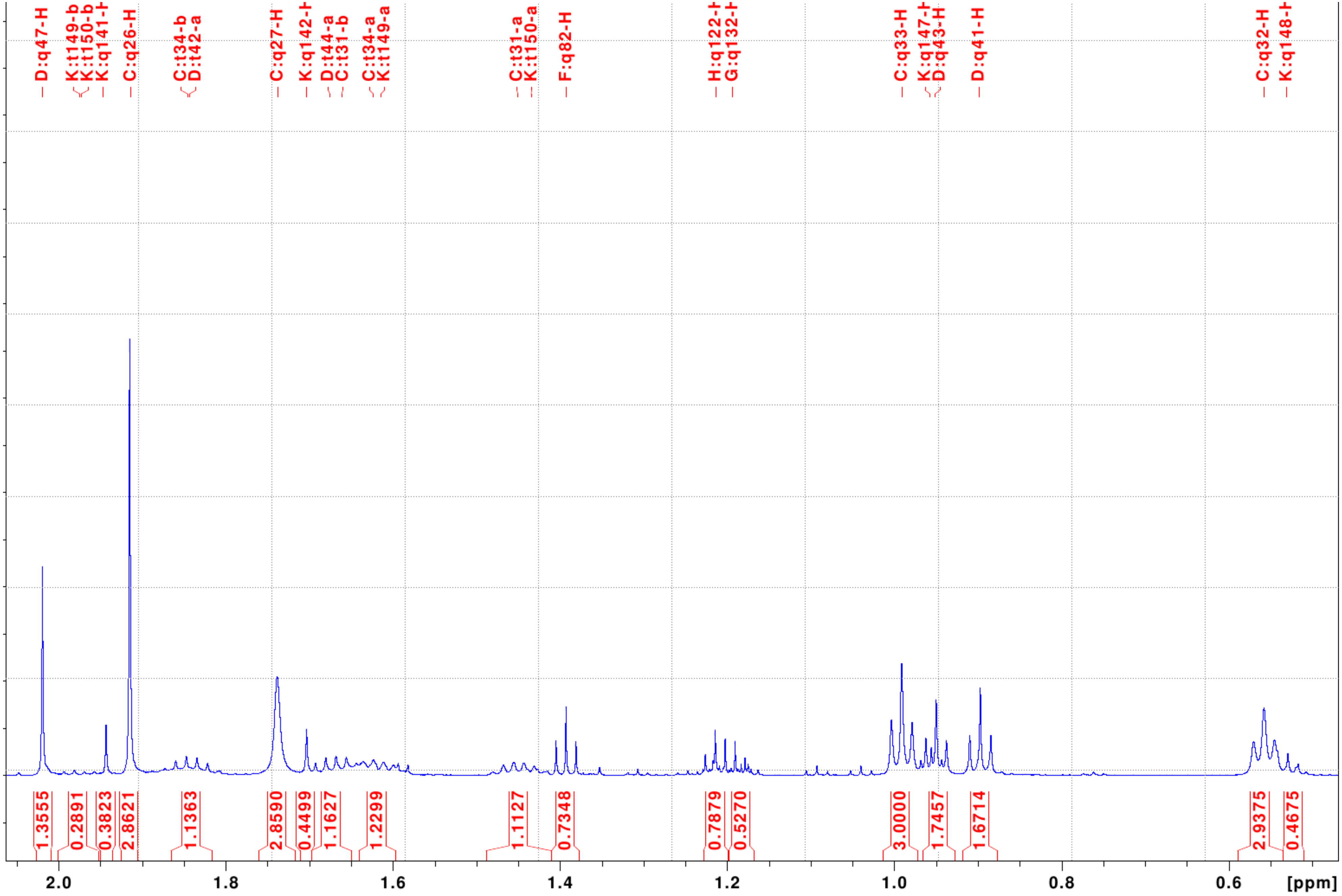
D:q47-H
K:t149-b
K:t150-b
K:q141-F
C:q26-H
C:t34-b
D:t42-a
C:q27-H
K:q142-F
D:t44-a
C:t31-b
C:t34-a
K:t149-a
C:t31-a
K:t150-a
F:q82-H
H:q122-F
G:q132-F
C:q33-H
K:q147-F
D:q43-H
D:q41-H
C:q32-H
K:q148-F
1.3555
0.2891
0.3823
2.8621
1.1363
2.8590
0.4499
1.1627
1.2299
1.1127
0.7348
0.7879
0.5270
3.0000
1.7457
1.6714
2.9375
0.4675
2.0
1.8
1.6
1.4
1.2
1.0
0.8
0.6
[ppm]

Structures and NMR signal assignments for products in the reaction mixture 3 + BME

in toluene-d_8 at 25 °C

Signal assignments

Some peak labels in NMR spectra could not be assigned to structures because of low product content.

Structure A: heterocycle
D ~ 0.91e-9 (V ~ 8.1)

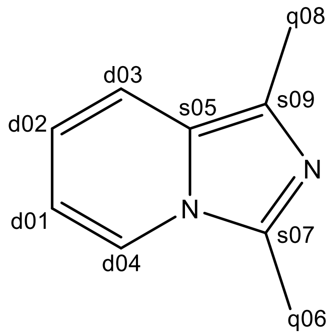

Experiment Bruker_156, 1D 13C
d01 111.9
d02 115.9
d03 117.8
d04 120.2
s05 126.2
q06 11.2
s07 132.9
q08 11.7
s09 125.4

Experiment Bruker_158, 1D 1H
d01-H 6.03
d02-H 6.22
d03-H 6.93
d04-H 6.85
q06-H 2.19
q08-H 2.38

Experiment Bruker_152, 2D 13C-1H via onebond (HSQC)
d01-H - d01(165 Hz)
d02-H - d02(166 Hz)
d03-H - d03(165 Hz)
d04-H - d04(183 Hz)
q06-H - q06(129 Hz)
q08-H - q08(127 Hz)

Experiment Bruker_155, 2D 1H-13C via onebond (H-C correlation)
d01 - d01-H
d02 - d02-H
d03 - d03-H
d04 - d04-H
q06 - q06-H
q08 - q08-H

Experiment Bruker_151, 2D 1H-1H via Jcoupling (COSY)
d01-H - d02-H d03-H(weak) d04-H
d02-H - d01-H d03-H d04-H?(weak) q08-H?(weak)
d03-H - d01-H(weak) d02-H d04-H(weak)
d04-H - d01-H d03-H(weak)

Experiment Bruker_153, 2D 13C-1H via Jcoupling (HMBC)
d01-H - d03 d04
d02-H - d01(weak) d04 s05
d03-H - d01 s05
d04-H - d01 d02 s05 s07(weak)
q06-H - s07
q08-H - d02(weak) d03(weak) s05 s09

Experiment Bruker_154, 2D 1H-1H via through-space (NOESY)
d03-H - q08-H?
d04-H - q06-H
q06-H - d04-H

Structure B: diethylketoxime
D ~ 1.00e-9 (V ~ 6.1)

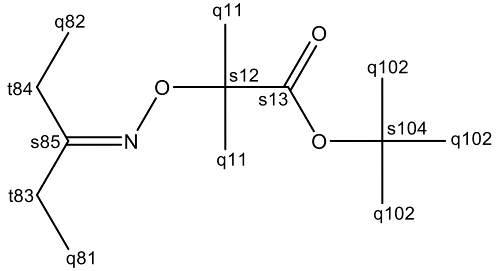

Experiment Bruker_156, 1D 13C
q11 23.9
s12 80.8
s13 173.7
q81 10.1
q82 10.6
t83 21.5
t84 27.1
s85 162.0
q102 27.6
s104 79.9

Experiment Bruker_158, 1D 1H
q11-H 1.52
q81-H 1.00
q82-H 0.98
t83-a 2.22
t83-b 2.22
t84-a 2.01
t84-b 2.01
q102-H 1.38

Experiment Bruker_152, 2D 13C-1H via onebond (HSQC)
q102-H - q102(132 Hz)
q11-H - q11(128 Hz)
q81-H - q81(128 Hz)
q82-H - q82(128 Hz)
t83-a - t83(129 Hz)
t83-b - t83(129 Hz)
t84-a - t84(127 Hz)
t84-b - t84(127 Hz)

Experiment Bruker_155, 2D 1H-13C via onebond (H-C correlation)
q102 - q102-H
q11 - q11-H
q81 - q81-H
q82 - q82-H
t83 - t83-a t83-b
t84 - t84-a t84-b

Experiment Bruker_151, 2D 1H-1H via Jcoupling (COSY)
q81-H - t83-a t83-b
q82-H - t84-a t84-b
t83-a - q81-H
t83-b - q81-H
t84-a - q82-H
t84-b - q82-H

Experiment Bruker_153, 2D 13C-1H via Jcoupling (HMBC)
q102-H - q102 s104
q11-H - q11 s12 s13
q81-H - s85 t83
q82-H - s85 t84
t83-a - q81 s85 t84
t83-b - q81 s85 t84
t84-a - q82 s85 t83
t84-b - q82 s85 t83

Experiment Bruker_154, 2D 1H-1H via through-space (NOESY)
t84 - t83

Structure C: isobutanoate
D ~ 1.34e-9 (V ~ 2.5)

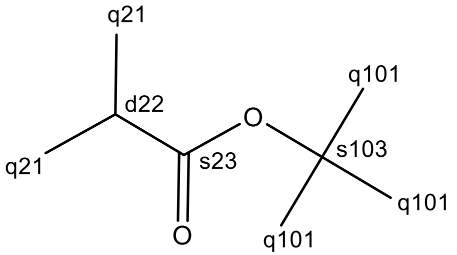

Experiment Bruker_156, 1D 13C
q21 18.8
d22 34.8
s23 176.3
q101 27.6
s103 79.3

Experiment Bruker_158, 1D 1H
q21-H 1.04
d22-H 2.31
q101-H 1.35

Experiment Bruker_152, 2D 13C-1H via onebond (HSQC)
d22-H - d22
q101-H - q101(127 Hz)
q21-H - q21(127 Hz)

Experiment Bruker_155, 2D 1H-13C via onebond (H-C correlation)
d22 - d22-H
q101 - q101-H
q21 - q21-H

Experiment Bruker_151, 2D 1H-1H via Jcoupling (COSY)
d22-H - q21-H
q21-H - d22-H

Experiment Bruker_153, 2D 13C-1H via Jcoupling (HMBC)
d22-H - q21 s23
q101-H - q101 s103
q21-H - d22 q21 s23

Structure D: BME
D ~ 1.48e-9 (V ~ 1.9)

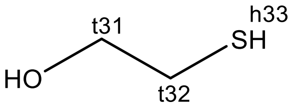

Experiment Bruker_156, 1D 13C
t31 64.0
t32 27.2

Experiment Bruker_158, 1D 1H
t31-a 3.46
t31-b 3.46
t32-a 2.38
t32-b 2.38
h33-H 1.36

Experiment Bruker_152, 2D 13C-1H via onebond (HSQC)
t31-a - t31(143 Hz)
t31-b - t31(143 Hz)
t32-a - t32(140 Hz)
t32-b - t32(140 Hz)

Experiment Bruker_155, 2D 1H-13C via onebond (H-C correlation)
t31 - t31-a t31-b
t32 - t32-a t32-b

Experiment Bruker_151, 2D 1H-1H via Jcoupling (COSY)
h33-H - t32-a t32-b
t31-a - t32-a? t32-b?
t31-b - t32-a? t32-b?
t32-a - h33-H t31-a? t31-b?
t32-b - h33-H t31-a? t31-b?

Experiment Bruker_153, 2D 13C-1H via Jcoupling (HMBC)
h33-H - t31
t31-a - t32
t31-b - t32
t32-a - t31
t32-b - t31

Structure E: BME dimer S-S
D ~ 0.83e-9 (V ~ 10.7)

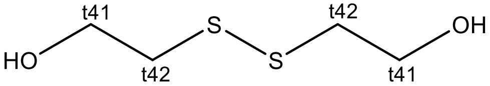

Experiment Bruker_156, 1D 13C
t41 60.4
t42 41.3

Experiment Bruker_158, 1D 1H
t41-a 3.76
t41-b 3.76
t42-a 2.73
t42-b 2.73

Experiment Bruker_152, 2D 13C-1H via onebond (HSQC)
t41-a - t41(144 Hz)
t41-b - t41(144 Hz)
t42-a - t42(139 Hz)
t42-b - t42(139 Hz)

Experiment Bruker_155, 2D 1H-13C via onebond (H-C correlation)
t41 - t41-a t41-b
t42 - t42-a t42-b

Experiment Bruker_151, 2D 1H-1H via Jcoupling (COSY)
t41-a - t42-a? t42-b?
t41-b - t42-a? t42-b?
t42-a - t41-a? t41-b?
t42-b - t41-a? t41-b?

Experiment Bruker_153, 2D 13C-1H via Jcoupling (HMBC)
t41-a - t42
t41-b - t42
t42-a - t41
t42-b - t41

Structure F: amine
D ~ 0.79e-9 (V ~ 12.4)

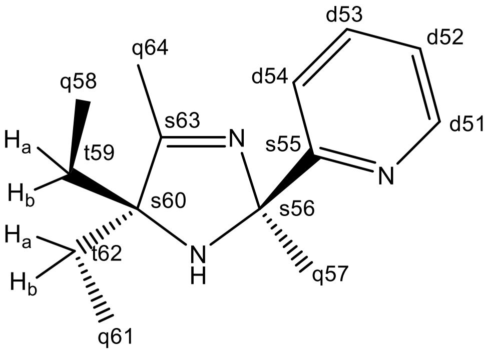

Experiment Bruker_156, 1D 13C
d51 147.9
d52 122.1
d53 136.6
d54 120.7
s55 164.4
s56 91.5
q57 32.3
q58 8.2
t59 30.2
s60 77.3
q61 8.7
t62 30.9
s63 176.1
q64 14.6

Experiment Bruker_158, 1D 1H
d51-H 8.37
d52-H 6.76
d53-H 7.33
d54-H 7.66
q57-H 1.72

q58-H   0.41
t59-a   1.13
t59-b   1.32
q61-H   0.91
t62-a   1.32
t62-b   1.47
q64-H   1.71

Experiment Bruker_152, 2D 13C-1H via onebond (HSQC)
d51-H - d51(179 Hz)
d52-H - d52(164 Hz)
d53-H - d53(163 Hz)
d54-H - d54(164 Hz)
q57-H - q57(128 Hz)
q58-H - q58
q61-H - q61
q64-H - q64(127 Hz)
t59-a - t59
t59-b - t59
t62-a - t62
t62-b - t62

Experiment Bruker_155, 2D 1H-13C via onebond (H-C correlation)
d51 - d51-H
d52 - d52-H
d53 - d53-H
d54 - d54-H
q57 - q57-H
q58 - q58-H
q61 - q61-H
q64 - q64-H
t59 - t59-a t59-b
t62 - t62-a t62-b

Experiment Bruker_151, 2D 1H-1H via Jcoupling (COSY)
d51-H - d52-H
d52-H - d51-H d53-H
d53-H - d51-H?(weak) d52-H d54-H
d54-H - d53-H

q58-H - t59-a t59-b
q61-H - t62-a t62-b
t59-a - q58-H t59-b
t59-b - q58-H t59-a
t62-a - q61-H t62-b
t62-b - q61-H t62-a

Experiment Bruker_153, 2D 13C-1H via Jcoupling (HMBC)
d51-H - d52 d53 s55
d52-H - d51 d54
d53-H - d51 s55
d54-H - d52 s56
q57-H - s55 s56
q58-H - s60 t59
q61-H - s60 t62
q64-H - s60 s63
t59-a - q58 s60 t62
t59-b - q58 s60 s63
t62-a - q61 s60 s63 t59
t62-b - q61 s60 s63

Experiment Bruker_154, 2D 1H-1H via through-space (NOESY)
d54-H - q57-H
q57-H - d54-H q61-H t59-b?
q58-H - q64-H
q61-H - q57-H
q64-H - q58-H q61-H? t59-a t59-b? t62-a? t62-b?
t59-a - q64-H

Structure G: diethylketone
D ~ 1.83e-9 (V ~ 1.0)

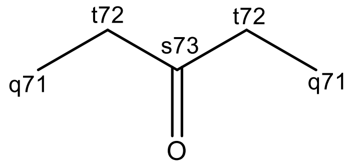

Experiment Bruker_156, 1D 13C
q71   7.6
t72   34.9
s73 211.4

Experiment Bruker_158, 1D 1H
q71-H   0.88
t72-a   1.93
t72-b   1.93

Experiment Bruker_152, 2D 13C-1H via onebond (HSQC)
q71-H - q71(127 Hz)
t72-a - t72(128 Hz)
t72-b - t72(128 Hz)

Experiment Bruker_155, 2D 1H-13C via onebond (H-C correlation)
q71 - q71-H
t72 - t72-a t72-b

Experiment Bruker_151, 2D 1H-1H via Jcoupling (COSY)
q71-H - t72-a t72-b
t72-a - q71-H
t72-b - q71-H

Experiment Bruker_153, 2D 13C-1H via Jcoupling (HMBC)
q71-H - s73 t72
t72-a - q71 s73
t72-b - q71 s73

Structure H: BME dimer
HS-CH2CH2-S-CH2CH2-OH
D unknown because of overlapping

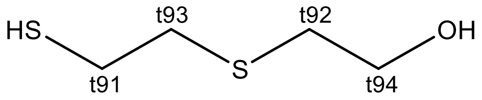

Experiment Bruker_156, 1D 13C
t91   24.7
t92   34.5
t93   36.0
t94   61.2

Experiment Bruker_158, 1D 1H
t91-a,b   2.40
t92-a,b   2.44
t93-a ,b 2.45
t94-a ,b 3.54

Experiment Bruker_152, 2D 13C-1H via onebond (HSQC)
t91-a,b - t91(140 Hz)
t92-a,b- t92(138 Hz)
t93-a,b- t93(143 Hz)
t94-a,b - t94(143 Hz)

Experiment Bruker_155, 2D 1H-13C via onebond (H-C correlation)
t91 - t91-a,b
t92 - t92-a,b
t93 - t93-a,b
t94 - t94-a,b

Experiment Bruker_151, 2D 1H-1H via Jcoupling (COSY)
t91-a,b - t93-a,b?
t92-a,b - t94-a,b?
t93-a,b - t91-a,b?
t94-a,b - t92-a,b?

Experiment Bruker_153, 2D 13C-1H via Jcoupling (HMBC)
t91-a,b - t93
t92-a,b - t91 t93 t94
t93-a,b - t91 t92
t94-a,b - t92

$^{13}C\{^{1}H\}$ NMR spectrum (150 MHz)

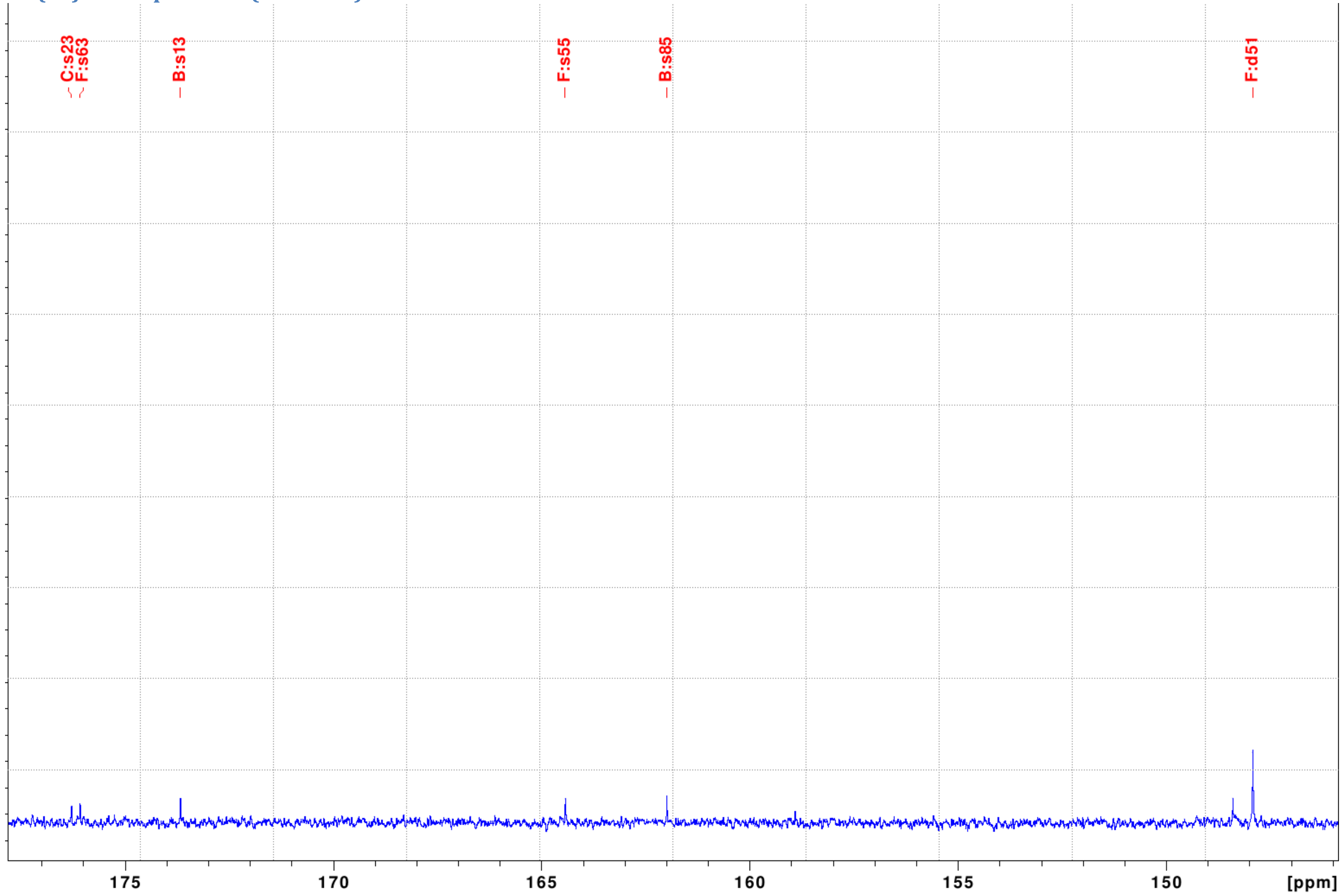

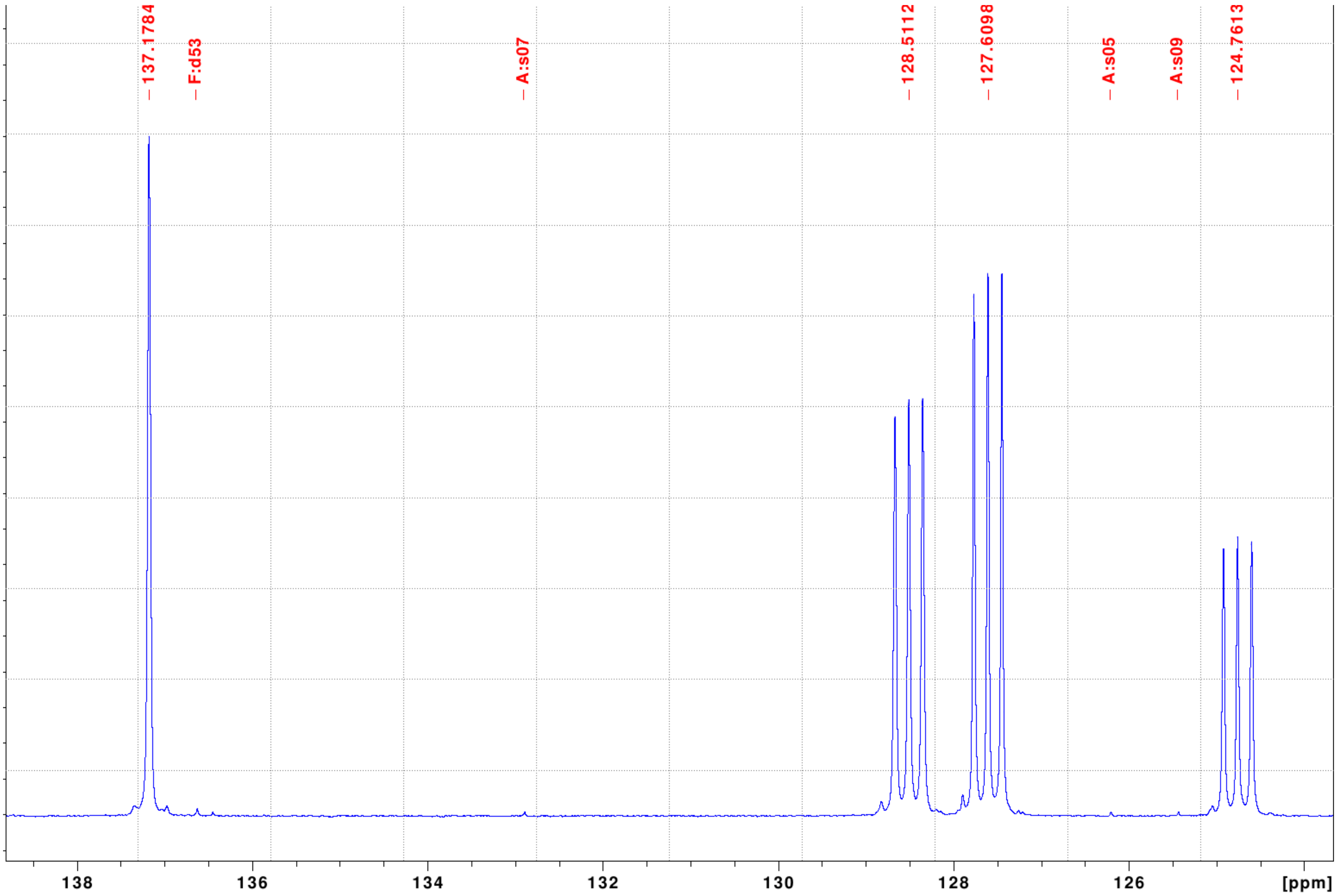
137.1784
F:d53
A:s07
128.5112
127.6098
A:s05
A:s09
124.7613
138
136
134
132
130
128
126
[ppm]

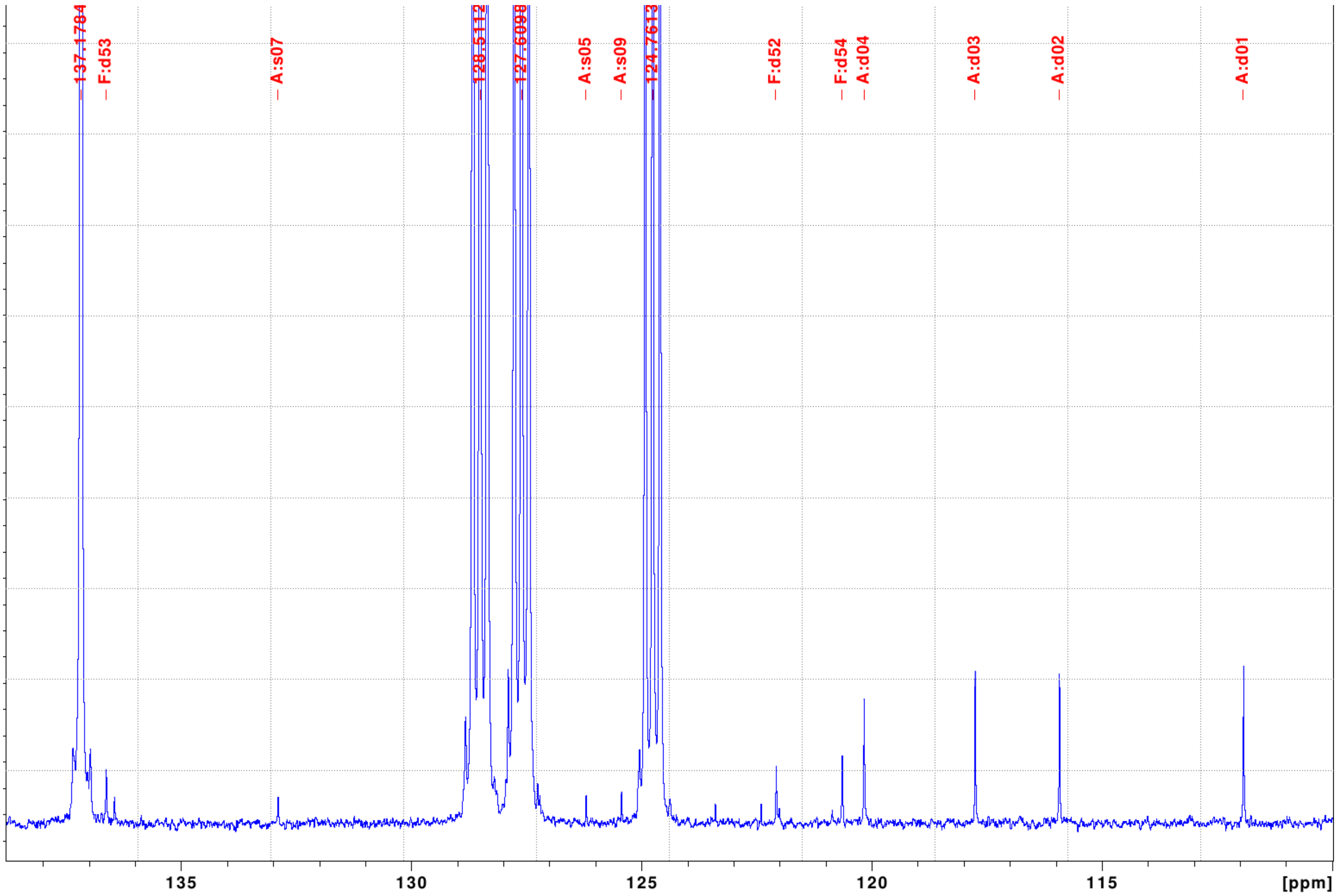
137.1784
F:d53
A:s07
128.5112
127.6098
A:s05
A:s09
124.7613
F:d52
F:d54
A:d04
A:d03
A:d02
A:d01
135
130
125
120
115
[ppm]

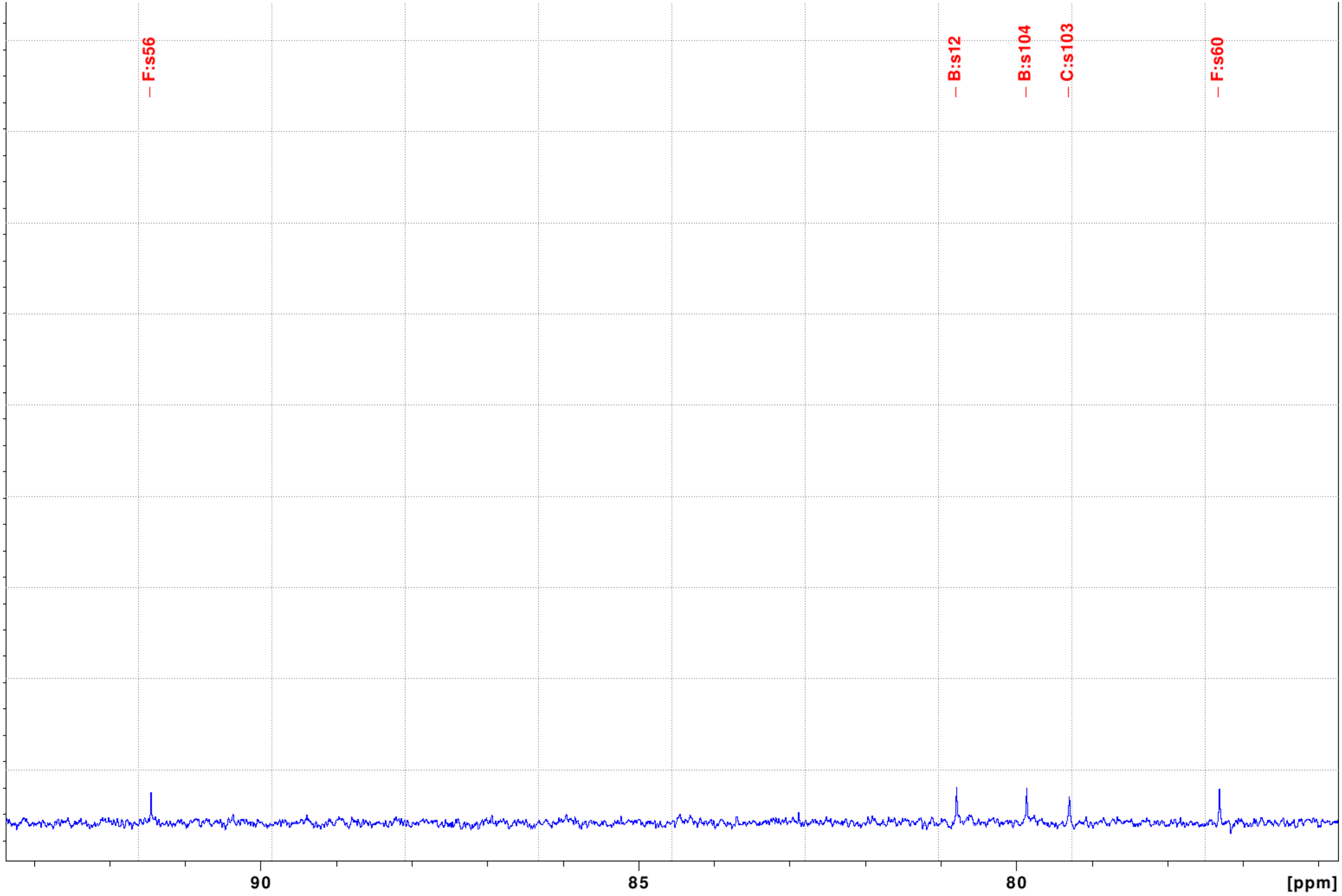

F:s56
B:s12
B:s104
C:s103
F:s60
90
85
80
[ppm]

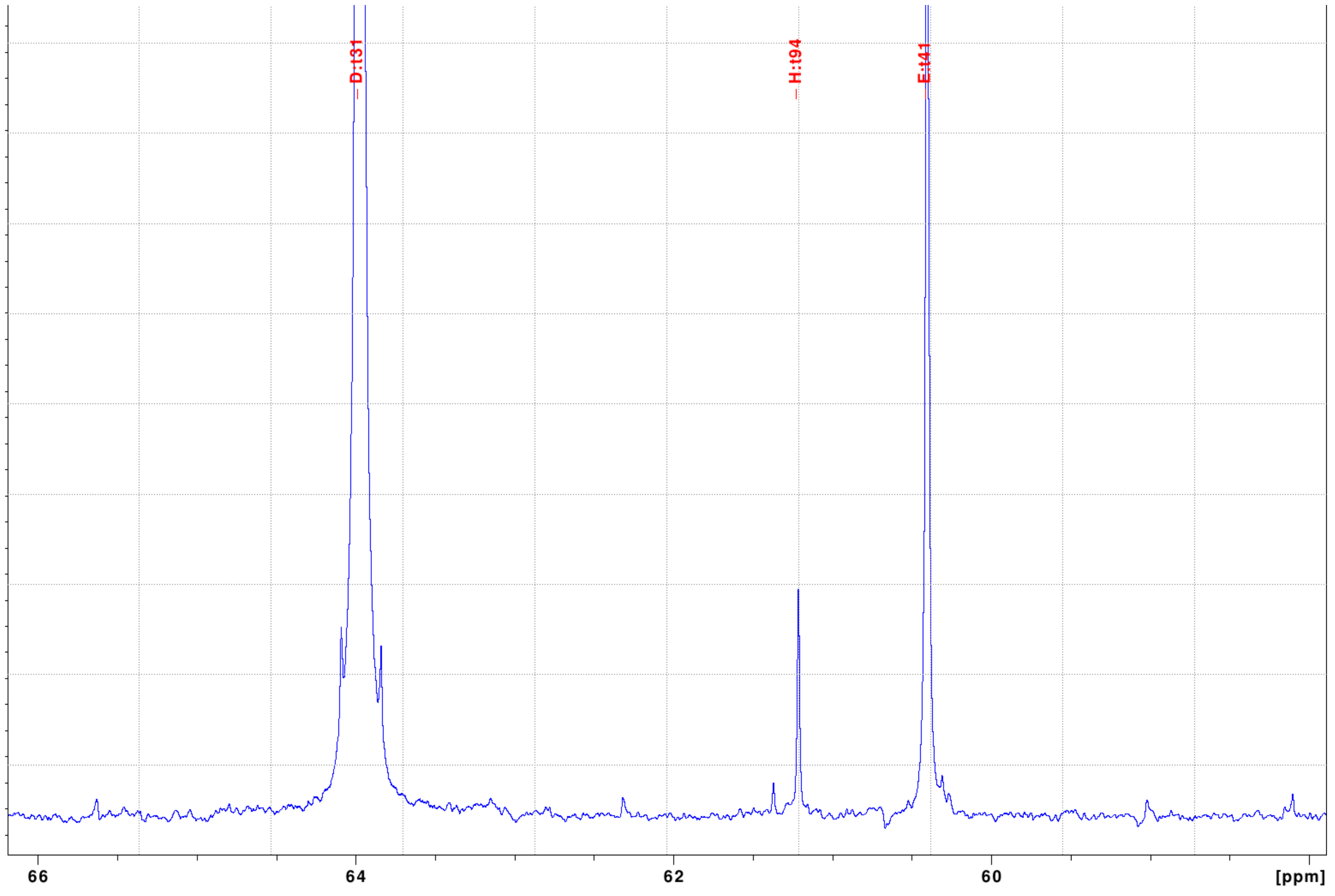
D:t31
H:t94
E:t41
66
64
62
60
[ppm]

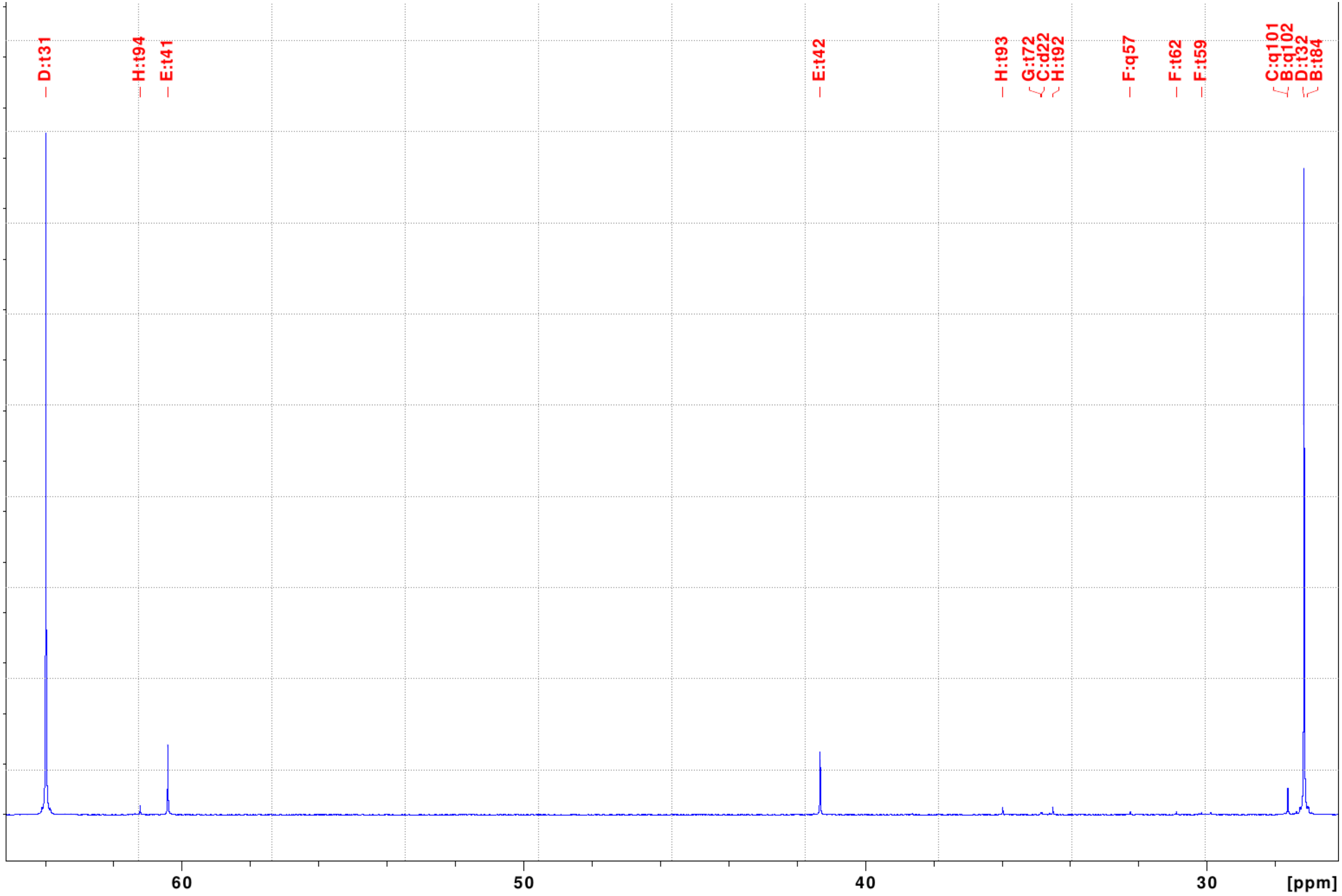
D:t31
H:t94
E:t41
E:t42
H:t93
G:t72
C:d22
H:t92
F:q57
F:t62
F:t59
C:q101
B:q102
D:t32
B:t84
60
50
40
30
[ppm]

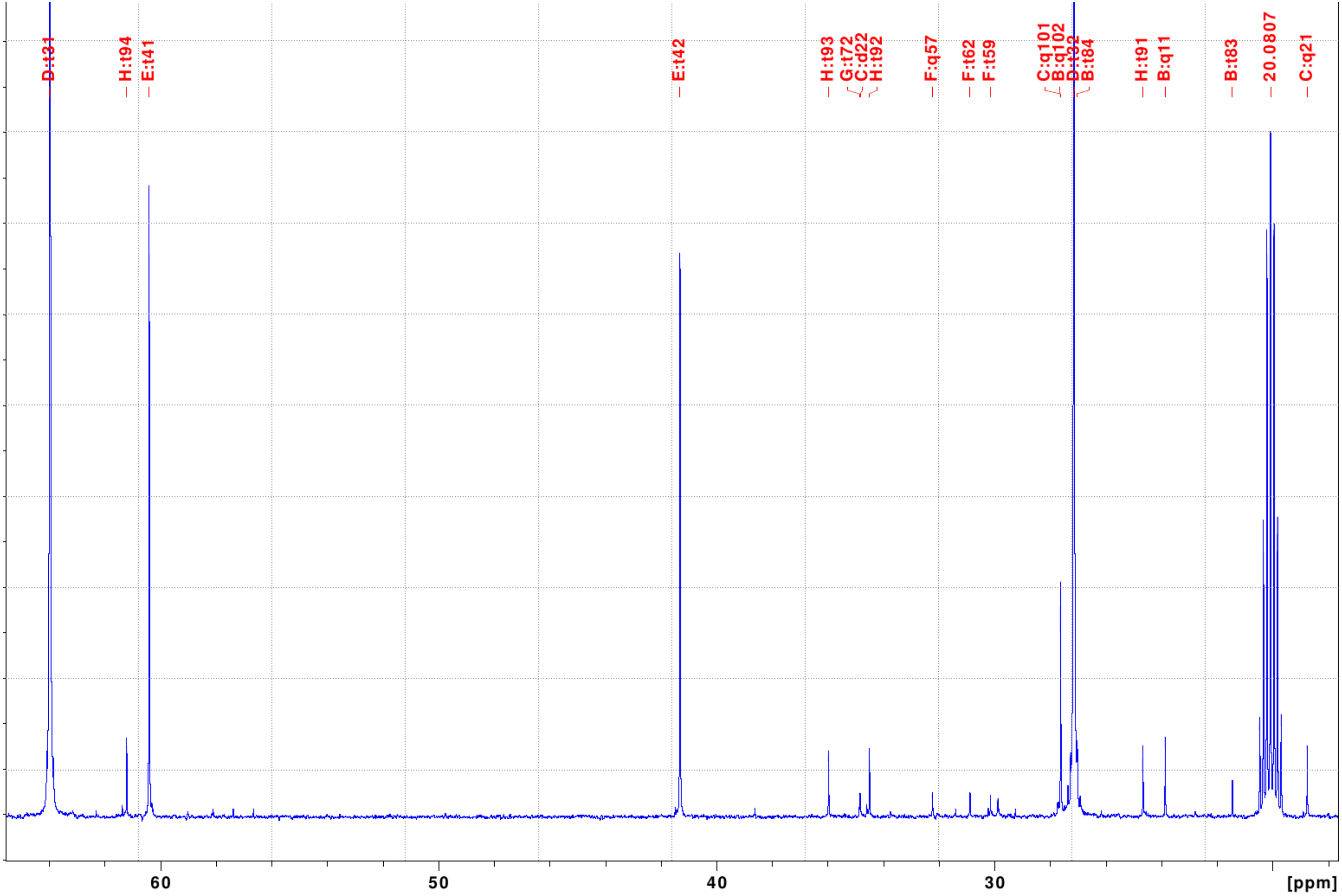

D:t31
H:t94
E:t41
E:t42
H:t93
G:t72
C:d22
H:t92
F:q57
F:t62
F:t59
C:q101
B:q102
D:t32
B:t84
H:t91
B:q11
B:t83
20.0807
C:q21
60
50
40
30
[ppm]

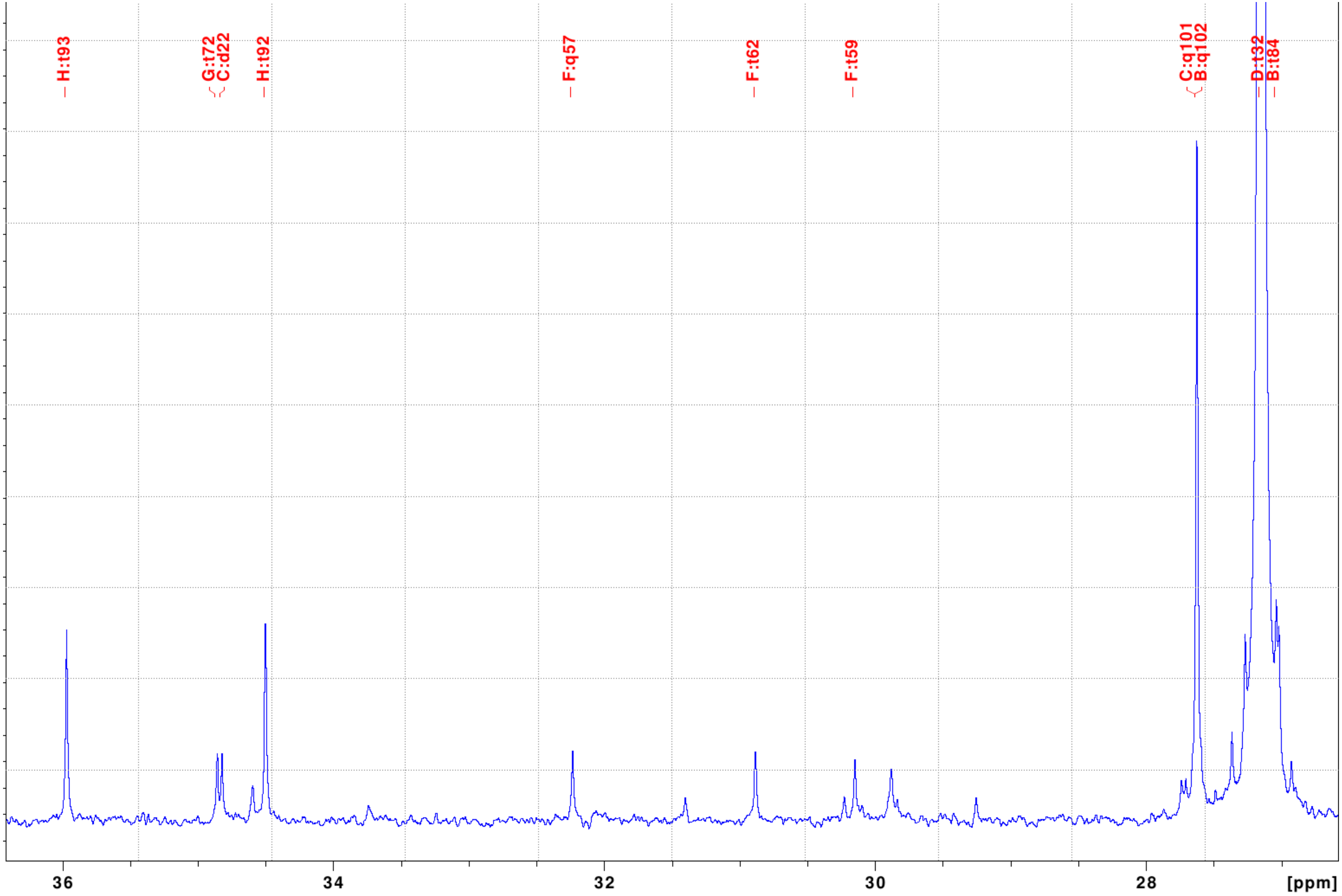

H:t93
G:t72
C:d22
H:t92
F:q57
F:t62
F:t59
C:q101
B:q102
D:t32
B:t84
36
34
32
30
28
[ppm]

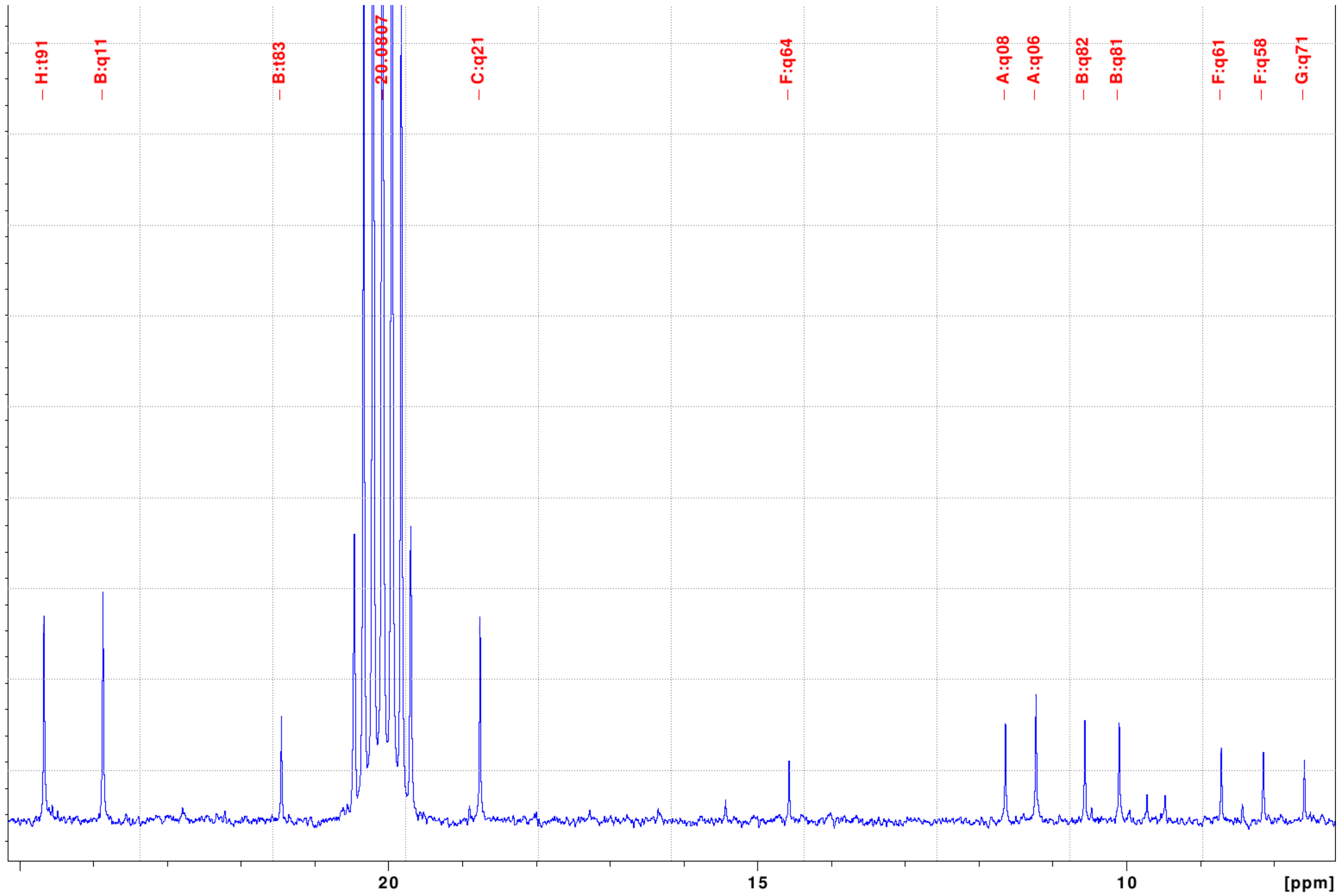

H:t91
B:q11
B:t83
20.0807
C:q21
F:q64
A:q08
A:q06
B:q82
B:q81
F:q61
F:q58
G:q71
20
15
10
[ppm]

^{1}H NMR spectrum (600 MHz)

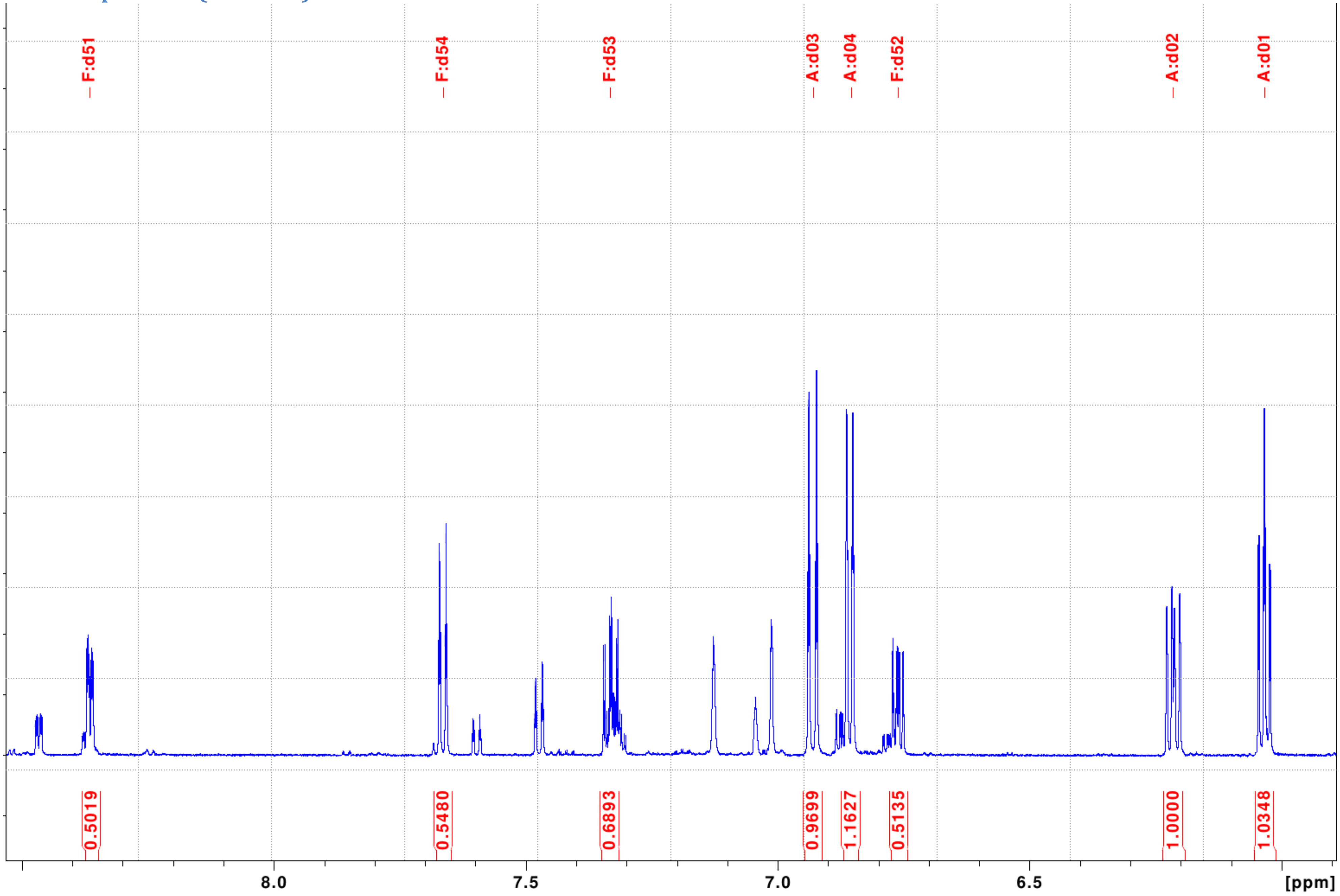

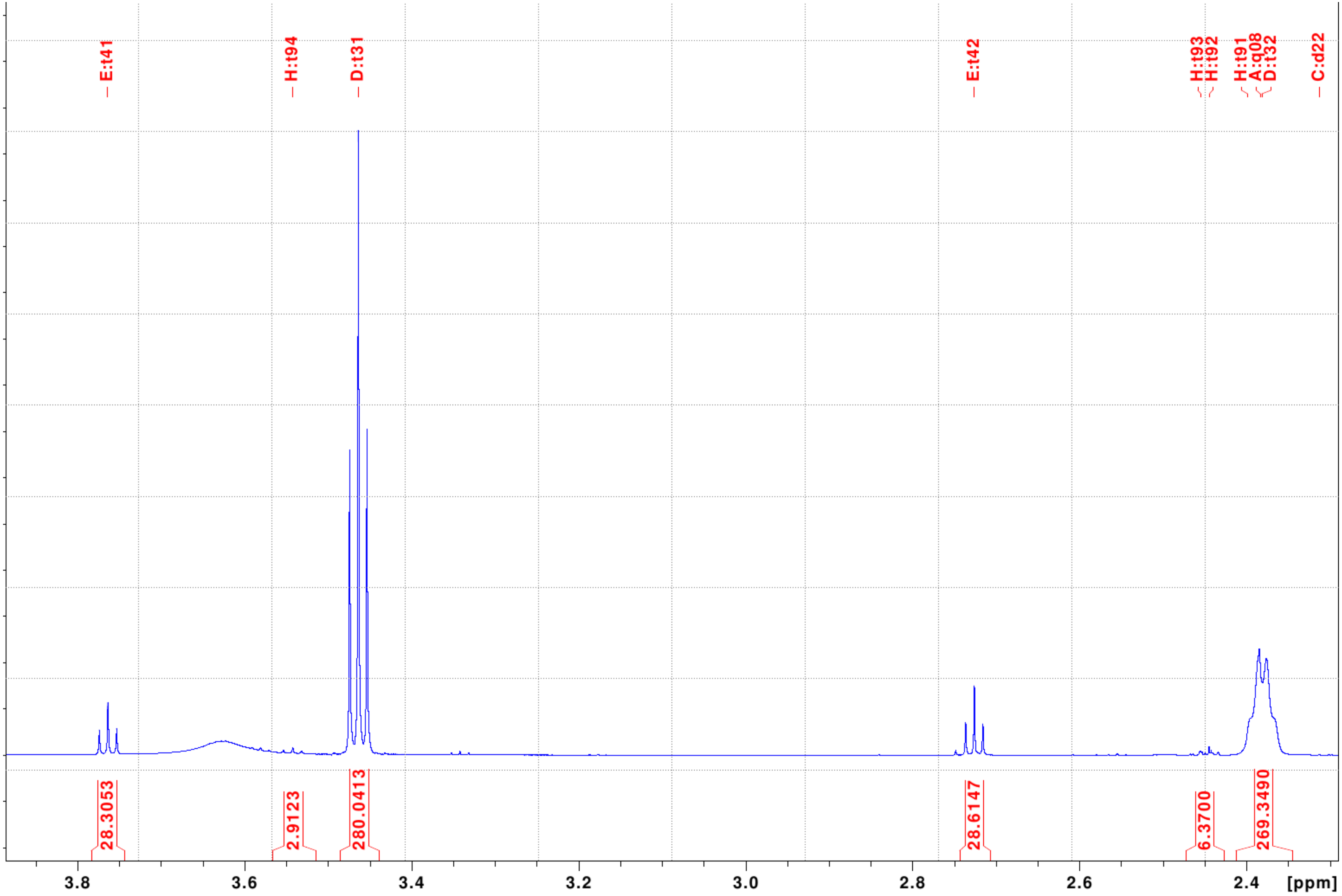

E:t41
H:t94
D:t31
E:t42
H:t93
H:t92
H:t91
A:q08
D:t32
C:d22
28.3053
2.9123
280.0413
28.6147
6.3700
269.3490
3.8
3.6
3.4
3.2
3.0
2.8
2.6
2.4
[ppm]

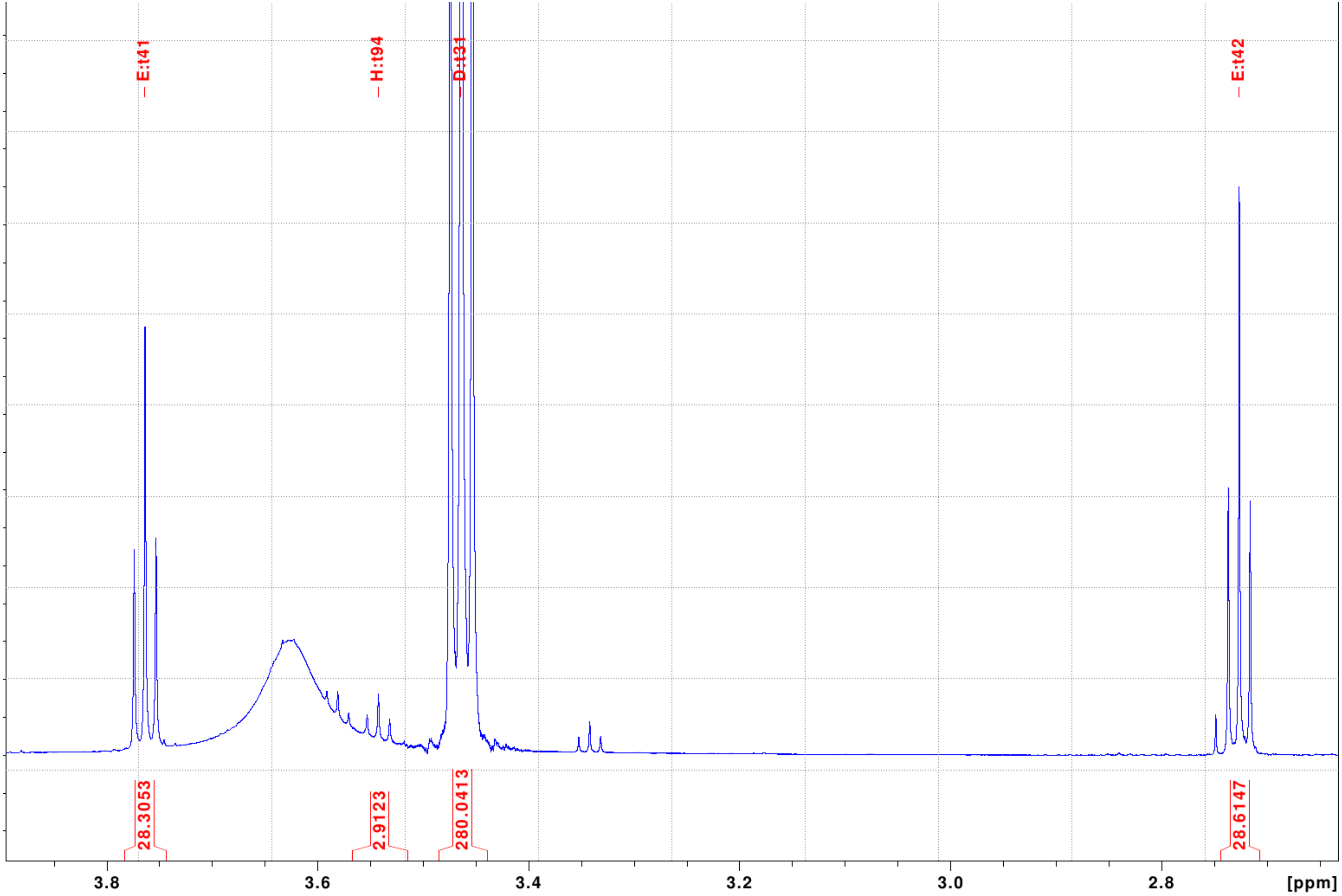
E:t41
H:t94
D:t31
E:t42
28.3053
2.9123
280.0413
28.6147
3.8
3.6
3.4
3.2
3.0
2.8
[ppm]

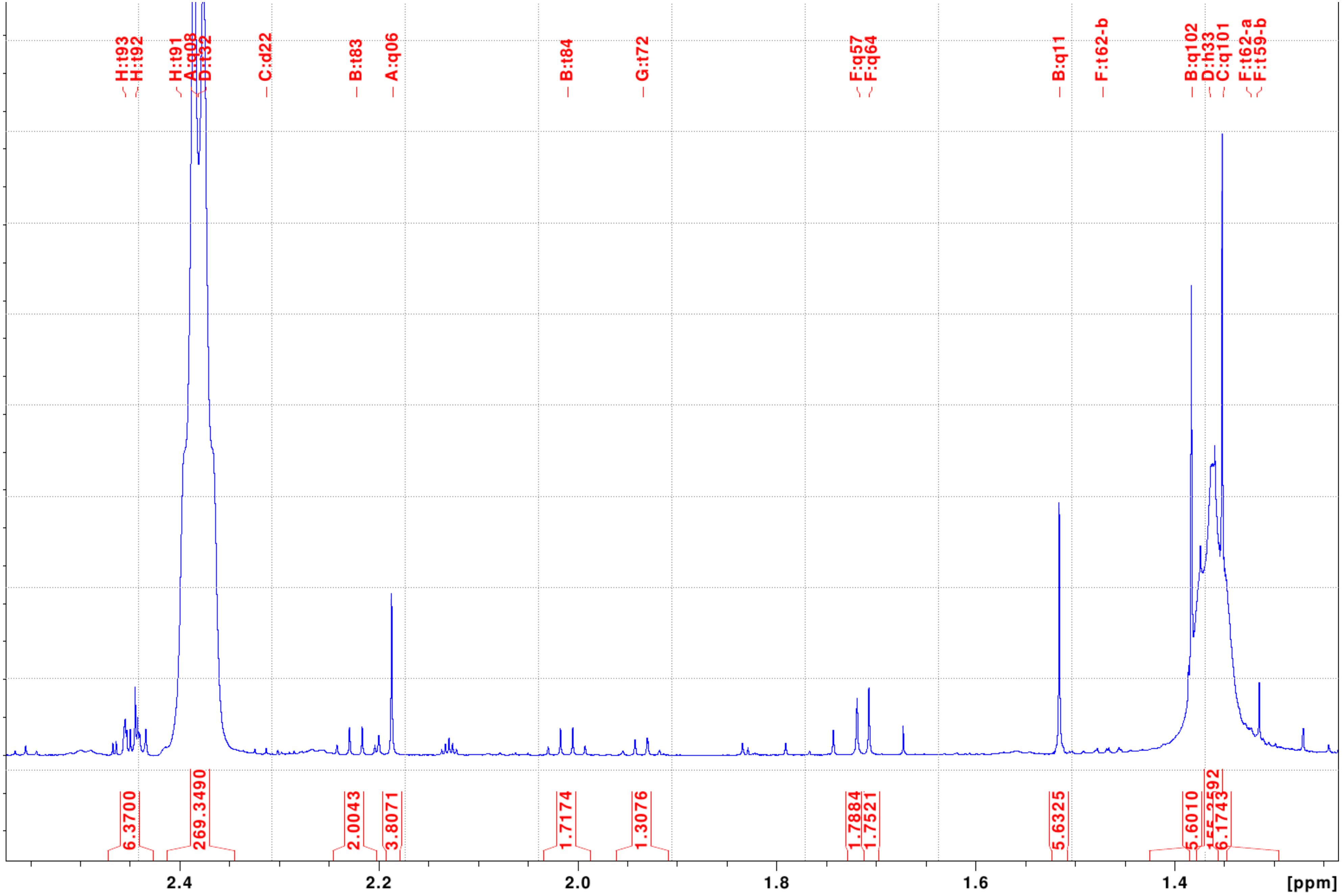
H:t93
H:t92
H:t91
A:q08
D:t32
C:d22
B:t83
A:q06
B:t84
G:t72
F:q57
F:q64
B:q11
F:t62-b
B:q102
D:h33
C:q101
F:t62-a
F:t59-b
6.3700
269.3490
2.0043
3.8071
1.7174
1.3076
1.7884
1.7521
5.6325
5.6010
15.2592
6.1743
2.4
2.2
2.0
1.8
1.6
1.4
[ppm]

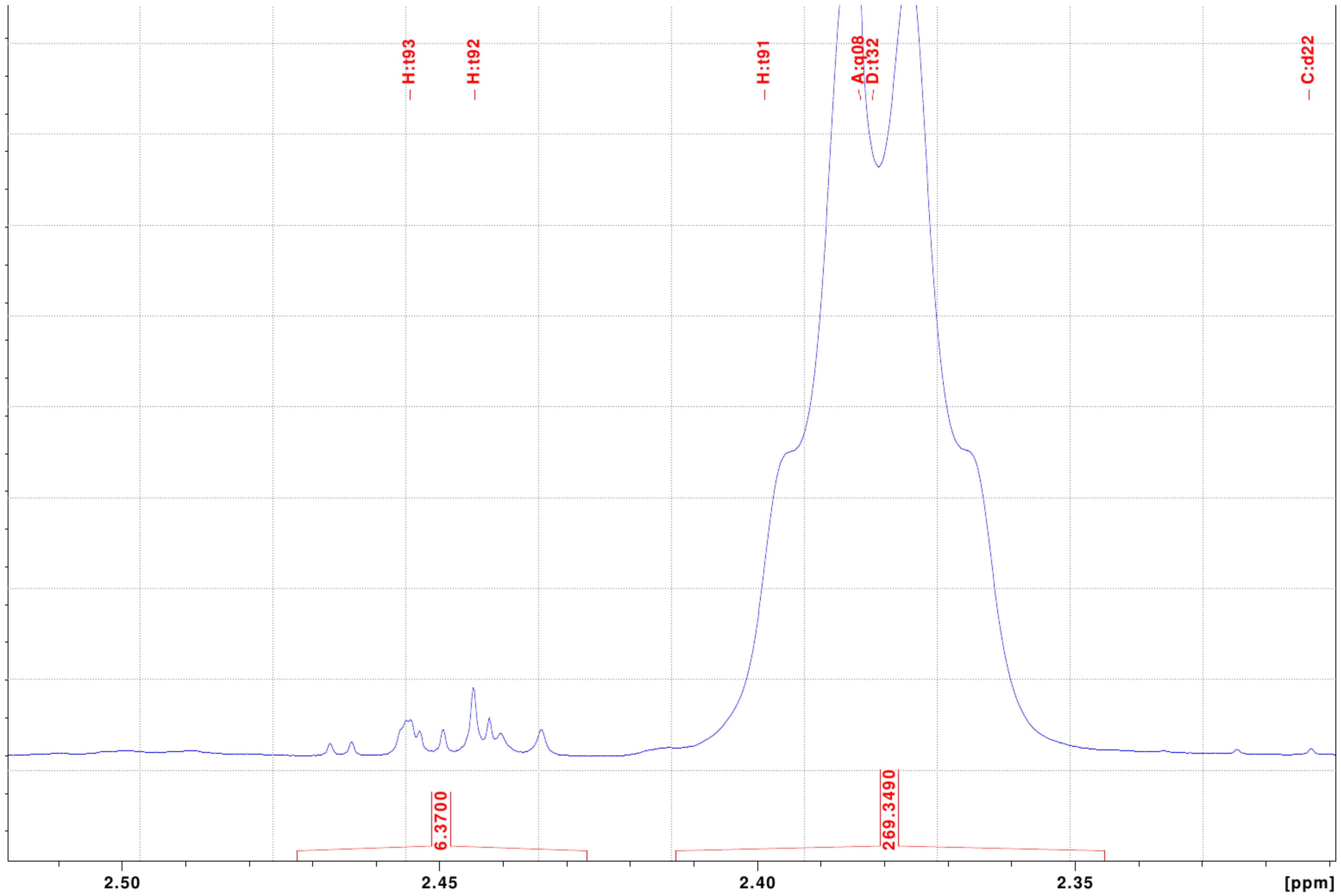

H:t93
H:t92
H:t91
A:q08
D:t32
C:d22
6.3700
269.3490
2.50
2.45
2.40
2.35
[ppm]

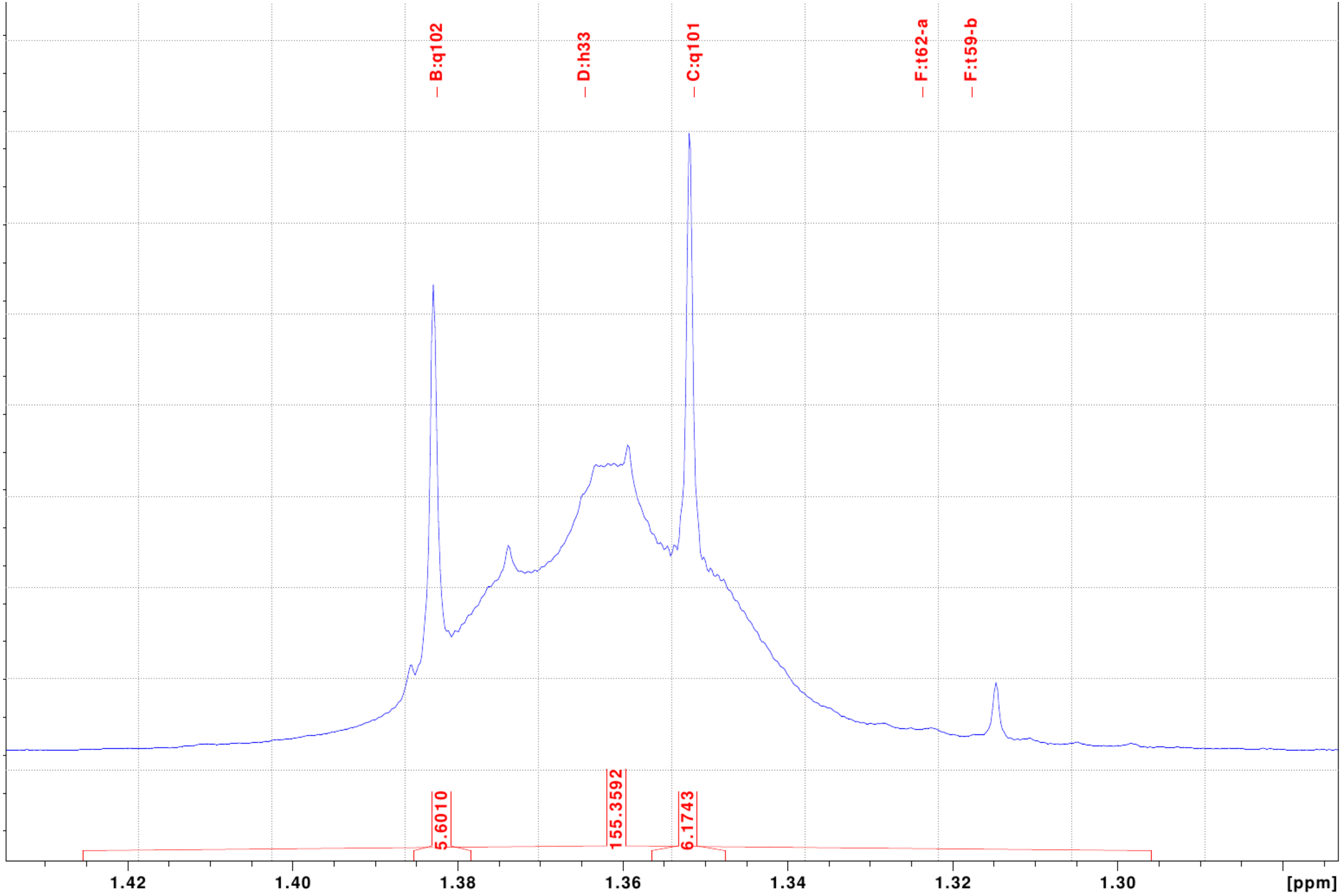
B:q102
D:h33
C:q101
F:t62-a
F:t59-b
5.6010
155.3592
6.1743
1.42
1.40
1.38
1.36
1.34
1.32
1.30
[ppm]

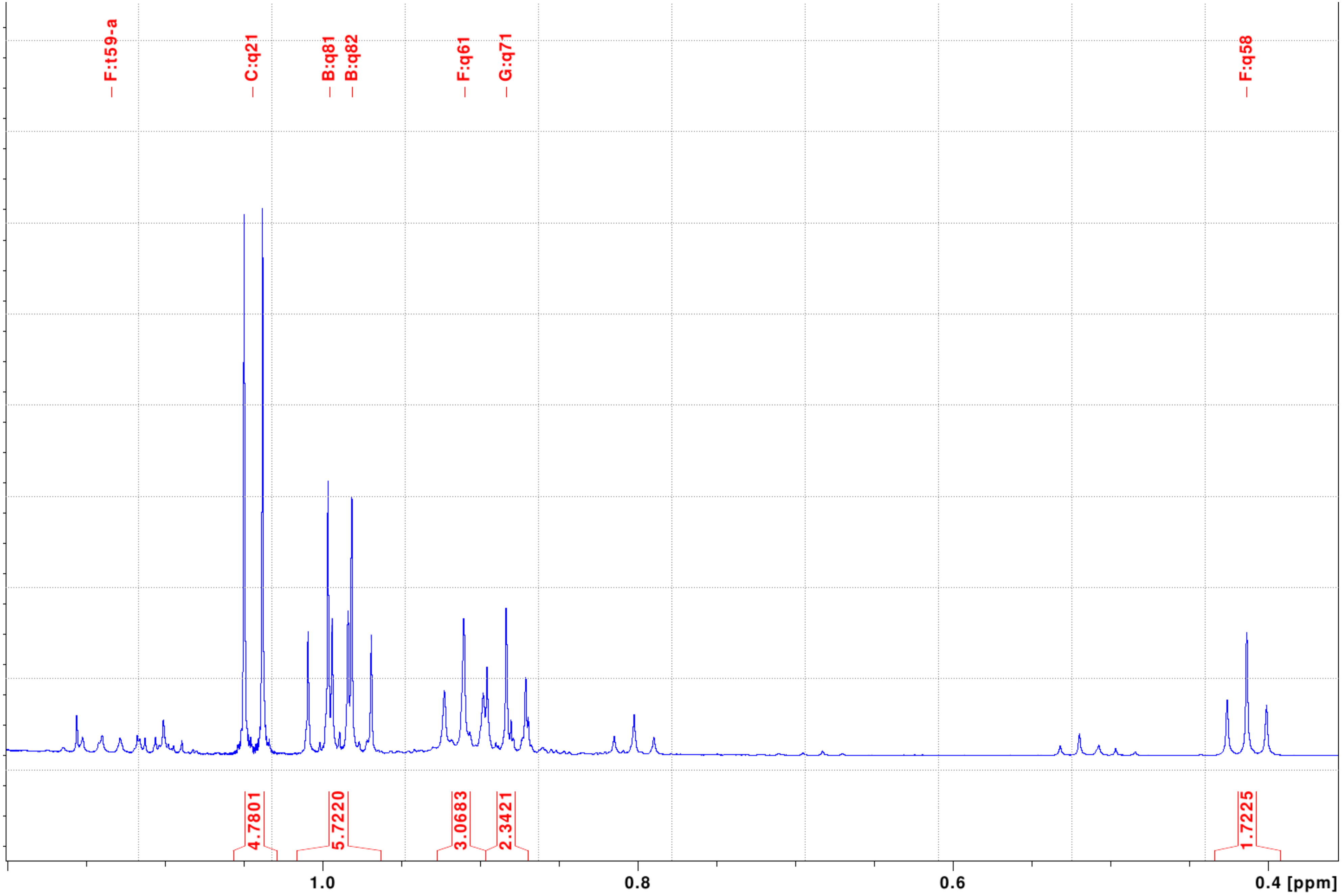
F:t59-a
C:q21
B:q81
B:q82
F:q61
G:q71
F:q58
4.7801
5.7220
3.0683
2.3421
1.7225
1.0
0.8
0.6
0.4 [ppm]

Structures and NMR signal assignments for products in the reaction mixture 1• + BME

in toluene-d_8 at 25 °C

Signal assignments

Some peak labels in NMR spectra could not be assigned to structures because of low product content.

Structure A, BME

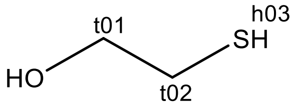

Experiment Bruker_71, 1D 13C
A:t01 63.9
A:t02 27.3

Experiment Bruker_82, 1D 1H
A:h03-H 1.27
A:t01-a 3.43
A:t01-b 3.43
A:t02-a 2.32
A:t02-b 2.32

Experiment Bruker_74, 2D 13C-1H via onebond (HSQC)
A:t01-a - t01
A:t01-b - t01
A:t02-a - t02
A:t02-b - t02

Experiment Bruker_77, 2D 1H-13C via onebond (H-C correlation)
A:t01 - t01-a t01-b
A:t02 - t02-a t02-b

Experiment Bruker_73, 2D 1H-1H via Jcoupling (COSY)
A:h03-H - t02-a t02-b
A:t01-a - t02-a? t02-b?
A:t01-b - t02-a? t02-b?
A:t02-a - h03-H t01-a? t01-b?
A:t02-b - h03-H t01-a? t01-b?

Experiment Bruker_88, 2D 13C-1H via Jcoupling (HMBC)
A:h03-H - t01 t02
A:t01-a - t02
A:t01-b - t02
A:t02-a - t01
A:t02-b - t01

Structure B, Amine

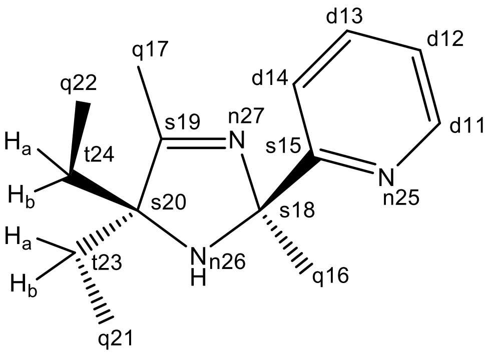

Experiment Bruker_71, 1D 13C
B:d11 147.8
B:d12 121.8
B:d13 136.2
B:d14 120.8
B:q16 32.8
B:q17 14.6
B:q21 8.7
B:q22 8.2
B:s15 165.0
B:s18 91.8
B:s19 174.6
B:s20 77.2
B:t23 31.2
B:t24 30.1

Experiment Bruker_82, 1D 1H
B:d11-H 8.36
B:d12-H 6.69
B:d13-H 7.23
B:d14-H 7.72
B:q16-H 1.77
B:q17-H 1.69
B:q21-H 0.97
B:q22-H 0.42
B:t23-a 1.33
B:t23-b 1.48
B:t24-a 1.14
B:t24-b 1.31

Experiment Bruker_91, 1D 15N
B:n25-N1 301.65
B:n26-N1 61.27
B:n27-N1 322.41

Experiment Bruker_74, 2D 13C-1H via onebond (HSQC)
B:d11-H - d11
B:d12-H - d12
B:d13-H - d13
B:d14-H - d14
B:q16-H - q16
B:q17-H - q17
B:q21-H - q21
B:q22-H - q22
B:t23-a - t23
B:t23-b - t23
B:t24-a - t24
B:t24-b - t24

Experiment Bruker_77, 2D 1H-13C via onebond (H-C correlation)
B:d11 - d11-H
B:d12 - d12-H
B:d13 - d13-H
B:d14 - d14-H
B:q16 - q16-H
B:q17 - q17-H
B:q21 - q21-H
B:q22 - q22-H
B:t23 - t23-a t23-b
B:t24 - t24-a t24-b

Experiment Bruker_73, 2D 1H-1H via Jcoupling (COSY)
B:d11-H - d12-H
B:d12-H - d11-H d13-H
B:d13-H - d12-H d14-H
B:d14-H - d13-H
B:q21-H - t23-a t23-b
B:q22-H - t24-a t24-b
B:t23-a - q21-H t23-b
B:t23-b - q21-H t23-a
B:t24-a - q22-H t24-b
B:t24-b - q22-H t24-a

Experiment Bruker_88, 2D 13C-1H via Jcoupling (HMBC)
B:d11-H - d12 d13 d14(weak) s15
B:d12-H - d11 d13(weak) d14 s15(weak)
B:d13-H - d11 s15
B:d14-H - d12 s15 s18
B:q16-H - s15 s18 s19
B:q17-H - q16(weak) s15(weak) s18(weak) s19 s20
B:q21-H - s20 t23
B:q22-H - s20 t24
B:t23-a - q21 s20 t24
B:t23-b - q21 s19 s20 t24
B:t24-a - q22 s19 s20 t23
B:t24-b - q22 s19 s20 t23

Experiment Bruker_76, 2D 1H-1H via through-space (NOESY)
B:d14-H - q16-H
B:q16-H - d14-H q21-H
B:q17-H - q21-H? q22-H? t23-a? t24-a?
B:q21-H - q16-H

Experiment Bruker_86, 2D 15N-1H via Jcoupling
B:d11-H - n25-N1
B:d12-H - n25-N1
B:d13-H - n25-N1(weak)
B:d14-H - n25-N1
B:q16-H - n26-N1 n27-N1
B:q17-H - n27-N1
B:q21-H - n26-N1(weak)
B:t23-a - n26-N1
B:t23-b - n26-N1
B:t24-a - n26-N1
B:t24-b - n26-N1

Structure C, BME dimer

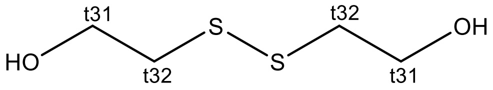

Experiment Bruker_71, 1D 13C
C:t31 60.4
C:t32 41.6

Experiment Bruker_82, 1D 1H
C:t31-a 3.74
C:t31-b 3.74
C:t32-a 2.68
C:t32-b 2.68

Experiment Bruker_74, 2D 13C-1H via onebond (HSQC)
C:t31-a - t31
C:t31-b - t31
C:t32-a - t32
C:t32-b - t32

Experiment Bruker_77, 2D 1H-13C via onebond (H-C correlation)
C:t31 - t31-a t31-b
C:t32 - t32-a t32-b

Experiment Bruker_73, 2D 1H-1H via Jcoupling (COSY)
C:t31-a - t32-a? t32-b?
C:t31-b - t32-a? t32-b?
C:t32-a - t31-a? t31-b?
C:t32-b - t31-a? t31-b?

Experiment Bruker_88, 2D 13C-1H via Jcoupling (HMBC)
C:t31-a - t32
C:t31-b - t32
C:t32-a - t31
C:t32-b - t31

Structure D, NO-BME adduct

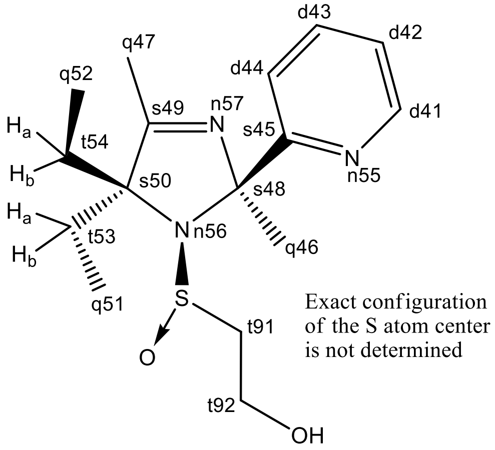

Experiment Bruker_71, 1D 13C
D:d41 148.2
D:d42 123.0
D:d43 136.0
D:d44 122.2
D:q46 27.9
D:q47 15.4
D:q51 9.7
D:q52 9.4
D:s45 159.6
D:s48 94.8
D:s49 170.7
D:s50 82.7
D:t53 30.2
D:t54 29.3
D:t91 58.3
D:t92 56.8

Experiment Bruker_82, 1D 1H
D:d41-H 8.41
D:d42-H 6.73
D:d43-H 7.19
D:d44-H 7.43
D:q46-H 2.24
D:q47-H 1.68
D:q51-H 0.94
D:q52-H 0.79
D:t53-a 1.37
D:t53-b 2.33
D:t54-a 1.58
D:t54-b 2.10
D:t91-a 2.46
D:t91-b 2.73
D:t92-a 3.77
D:t92-b 3.83

Experiment Bruker_91, 1D 15N
D:n55-N1 314.88
D:n56-N1 119.22
D:n57-N1 322.63

Experiment Bruker_74, 2D 13C-1H via onebond (HSQC)
D:d41-H - d41
D:d42-H - d42
D:d43-H - d43
D:d44-H - d44
D:q46-H - q46
D:q47-H - q47
D:q51-H - q51
D:q52-H - q52
D:t53-a - t53
D:t53-b - t53
D:t54-a - t54
D:t54-b - t54
D:t91-a - t91
D:t91-b - t91
D:t92-a - t92
D:t92-b - t92

Experiment Bruker_77, 2D 1H-13C via onebond (H-C correlation)
D:d41 - d41-H
D:d42 - d42-H
D:d43 - d43-H
D:d44 - d44-H
D:q46 - q46-H
D:q47 - q47-H
D:q51 - q51-H
D:q52 - q52-H
D:t53 - t53-a t53-b
D:t54 - t54-a t54-b
D:t91 - t91-a t91-b

Experiment Bruker_73, 2D 1H-1H via Jcoupling (COSY)
D:d41-H - d42-H
D:d42-H - d41-H d43-H
D:d43-H - d42-H d44-H
D:d44-H - d43-H
D:q51-H - t53-a t53-b
D:q52-H - t54-a t54-b
D:t53-a - q51-H t53-b
D:t53-b - q51-H t53-a
D:t54-a - q52-H t54-b
D:t54-b - q52-H t54-a
D:t91-a - t91-b t92-a t92-b
D:t91-b - t91-a t92-a t92-b
D:t92-a - t91-a t91-b t92-b
D:t92-b - t91-a t91-b t92-a

Experiment Bruker_88, 2D 13C-1H via Jcoupling (HMBC)
D:d41-H - d42 d43 s45
D:d42-H - d41 d44
D:d43-H - d41 s45
D:d44-H - d42 s45 s48
D:q46-H - d44(weak) s45 s48
D:q47-H - q46(weak) s45(weak) s48(weak) s49 s50

D:q51-H - s50 t53
D:q52-H - s50 t54
D:t53-a - q51 s49(weak)
D:t53-b - q51 s49 s50 t54
D:t54-a - q52 s49(weak) s50 t53
D:t54-b - q52 s49 s50 t53
D:t91-a - t92
D:t91-b - t92
D:t92-a - t91
D:t92-b - t91

Experiment Bruker_76, 2D 1H-1H via through-space (NOESY)
D:d44-H - q46-H
D:q46-H - d44-H q51-H
D:q47-H - q52-H? t53-a?
D:q51-H - q46-H
D:q52-H - t91-b
D:t54-b - t91-b
D:t91-b - q52-H t54-b

Experiment Bruker_86, 2D 15N-1H via Jcoupling
D:d41-H - n55-N1
D:d42-H - n55-N1
D:d44-H - n55-N1
D:q46-H - n55-N1 n56-N1 n57-N1
D:q47-H - n57-N1
D:t53-a - n56-N1
D:t54-a - n56-N1
D:t54-b - n56-N1(weak)

Structure E, diethyl ketone

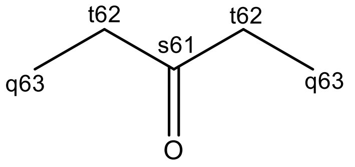

Experiment Bruker_71, 1D 13C
E:q63   7.6
E:s61 209.5
E:t62   34.8

Experiment Bruker_82, 1D 1H
E:q63-H   0.91
E:t62-a   1.91
E:t62-b   1.91

Experiment Bruker_74, 2D 13C-1H via onebond (HSQC)
E:q63-H - q63
E:t62-a - t62
E:t62-b - t62

Experiment Bruker_77, 2D 1H-13C via onebond (H-C correlation)
E:q63 - q63-H
E:t62 - t62-a t62-b

Experiment Bruker_73, 2D 1H-1H via Jcoupling (COSY)
E:q63-H - t62-a t62-b
E:t62-a - q63-H
E:t62-b - q63-H

Experiment Bruker_88, 2D 13C-1H via Jcoupling (HMBC)
E:q63-H - s61 t62
E:t62-a - q63 s61
E:t62-b - q63 s61

Structure F, Heterocycle

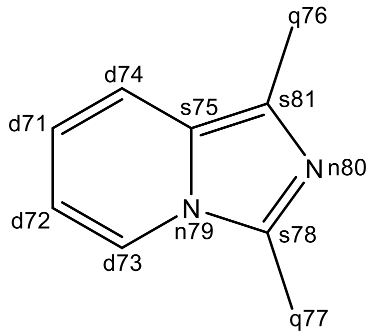

Experiment Bruker_71, 1D 13C
F:d71 115.6
F:d72 111.7
F:d73 120.1
F:d74 117.8
F:q76   11.7
F:q77   11.2
F:s75 126.2
F:s78 132.9
F:s81 126.2

Experiment Bruker_82, 1D 1H
F:d71-H   6.18
F:d72-H   5.98
F:d73-H   6.77
F:d74-H   6.90
F:q76-H   2.41
F:q77-H   2.15

Experiment Bruker_91, 1D 15N
F:n79-N1 187.57
F:n80-N1 264.29

Experiment Bruker_74, 2D 13C-1H via onebond (HSQC)
F:d71-H - d71
F:d72-H - d72
F:d73-H - d73
F:d74-H - d74
F:q76-H - q76
F:q77-H - q77

Experiment Bruker_77, 2D 1H-13C via onebond (H-C correlation)
F:d71 - d71-H
F:d72 - d72-H
F:d73 - d73-H
F:d74 - d74-H
F:q76 - q76-H
F:q77 - q77-H

Experiment Bruker_73, 2D 1H-1H via Jcoupling (COSY)
F:d71-H - d72-H? d74-H
F:d72-H - d73-H
F:d73-H - d72-H
F:d74-H - d71-H

Experiment Bruker_88, 2D 13C-1H via Jcoupling (HMBC)
F:d71-H - d72 d73 s75
F:d72-H - d73 d74
F:d73-H - d71 d72 s75
F:d74-H - d72 s81
F:q76-H - d71 d74 s75 s81
F:q77-H - s78

Experiment Bruker_76, 2D 1H-1H via through-space (NOESY)
F:d73-H - q77-H
F:d74-H - q76-H
F:q76-H - d74-H
F:q77-H - d73-H

Experiment Bruker_86, 2D 15N-1H via Jcoupling
F:d72-H - n79-N1
F:d73-H - n79-N1
F:d74-H - n79-N1
F:q76-H - n80-N1
F:q77-H - n79-N1 n80-N1

Impurity like acetone

Experiment Bruker_71, 1D 13C
G:q101   25.0
G:s102 198.8

Experiment Bruker_82, 1D 1H
G:q101-H   2.59

Experiment Bruker_74, 2D 13C-1H via onebond (HSQC)
G:q101-H - q101

Experiment Bruker_88, 2D 13C-1H via Jcoupling (HMBC)
G:q101-H - s102

Structure H, Unidentified hydrixylamine, Py ring unassigned

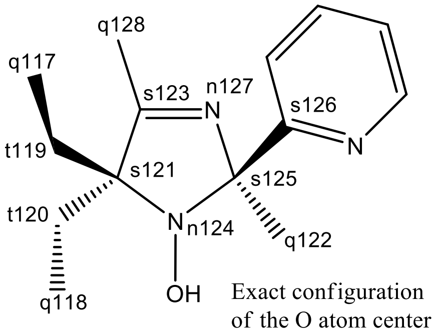

Exact configuration of the O atom center is not determined, C_{Py}, H_{Py}, N_{Py} not assigned

Experiment Bruker_71, 1D 13C
H:q117 8.4
H:q118 10.5
H:q122 24.8
H:q128 16.4
H:s121 80.4
H:s123 174.8
H:s125 95.2
H:s126 166.2
H:t119 30.2
H:t120 26.2

Experiment Bruker_82, 1D 1H
H:q117-H 0.55
H:q118-H 1.02
H:q122-H 1.88
H:q128-H 1.77
H:t119-a 1.26
H:t119-b 1.52
H:t120-a 1.46
H:t120-b 2.01

Experiment Bruker_91, 1D 15N
H:n124-N1 149.95
H:n127-N1 321.79

Experiment Bruker_74, 2D 13C-1H via onebond (HSQC)
H:q117-H - q117
H:q118-H - q118
H:q122-H - q122
H:q128-H - q128
H:t119-a - t119
H:t119-b - t119
H:t120-a - t120
H:t120-b - t120

Experiment Bruker_77, 2D 1H-13C via onebond (H-C correlation)
H:q117 - q117-H
H:q118 - q118-H
H:q122 - q122-H
H:q128 - q128-H

Experiment Bruker_73, 2D 1H-1H via Jcoupling (COSY)
H:q117-H - t119-a t119-b
H:q118-H - t120-a t120-b
H:t119-a - q117-H t119-b
H:t119-b - q117-H t119-a
H:t120-a - q118-H t120-b
H:t120-b - q118-H t120-a

Experiment Bruker_88, 2D 13C-1H via Jcoupling (HMBC)
H:q117-H - s121 t119
H:q118-H - s121 t120
H:q122-H - s125 s126
H:q128-H - s121 s123 s125(weak)
H:t119-a - q117 t120
H:t119-b - q117 s121 s123 t120
H:t120-a - q118 s121
H:t120-b - q118 s121 s123

Experiment Bruker_76, 2D 1H-1H via through-space (NOESY)
H:q118-H - q122-H
H:q122-H - q118-H

Experiment Bruker_86, 2D 15N-1H via Jcoupling
H:q122-H - n124-N1 n127-N1
H:q128-H - n127-N1
H:t119-a - n124-N1(weak)
H:t119-b - n124-N1
H:t120-a - n124-N1

Unidentified strange impuriries

Experiment Bruker_71, 1D 13C
I:d113 23.5
I:q112 7.6
I:s115 76.2
I:t114 33.7
I:t116 91.4

Experiment Bruker_82, 1D 1H
I:d113-H 1.94
I:q112-H 0.81
I:t114-a 1.27
I:t114-b 1.88
I:t116-a 4.16
I:t116-b 5.20

Experiment Bruker_91, 1D 15N
I:n111-N1 100.93

Experiment Bruker_74, 2D 13C-1H via onebond (HSQC)
I:d113-H - d113
I:q112-H - q112
I:t114-a - t114
I:t114-b - t114
I:t116-a - t116
I:t116-b - t116

Experiment Bruker_77, 2D 1H-13C via onebond (H-C correlation)
I:q112 - q112-H

Experiment Bruker_73, 2D 1H-1H via Jcoupling (COSY)
I:q112-H - t114-a t114-b
I:t114-a - q112-H t114-b
I:t114-b - q112-H t114-a
I:t116-a - t116-b(weak)
I:t116-b - t116-a(weak)

Experiment Bruker_88, 2D 13C-1H via Jcoupling (HMBC)
I:q112-H - s115 t114
I:t116-a - s115 t114
I:t116-b - s115 t114

Experiment Bruker_76, 2D 1H-1H via through-space (NOESY)
I:t116-a - t114-a?

Experiment Bruker_86, 2D 15N-1H via Jcoupling
I:d113-H - n111-N1
I:q112-H - n111-N1

The system has 2 distinct fragment(s)

Fragment 1:
I:q112
I:t114
I:s115
I:t116

Fragment 2:
I:d113

$^{13}C\{^{1}H\}$ NMR spectrum (150 MHz)

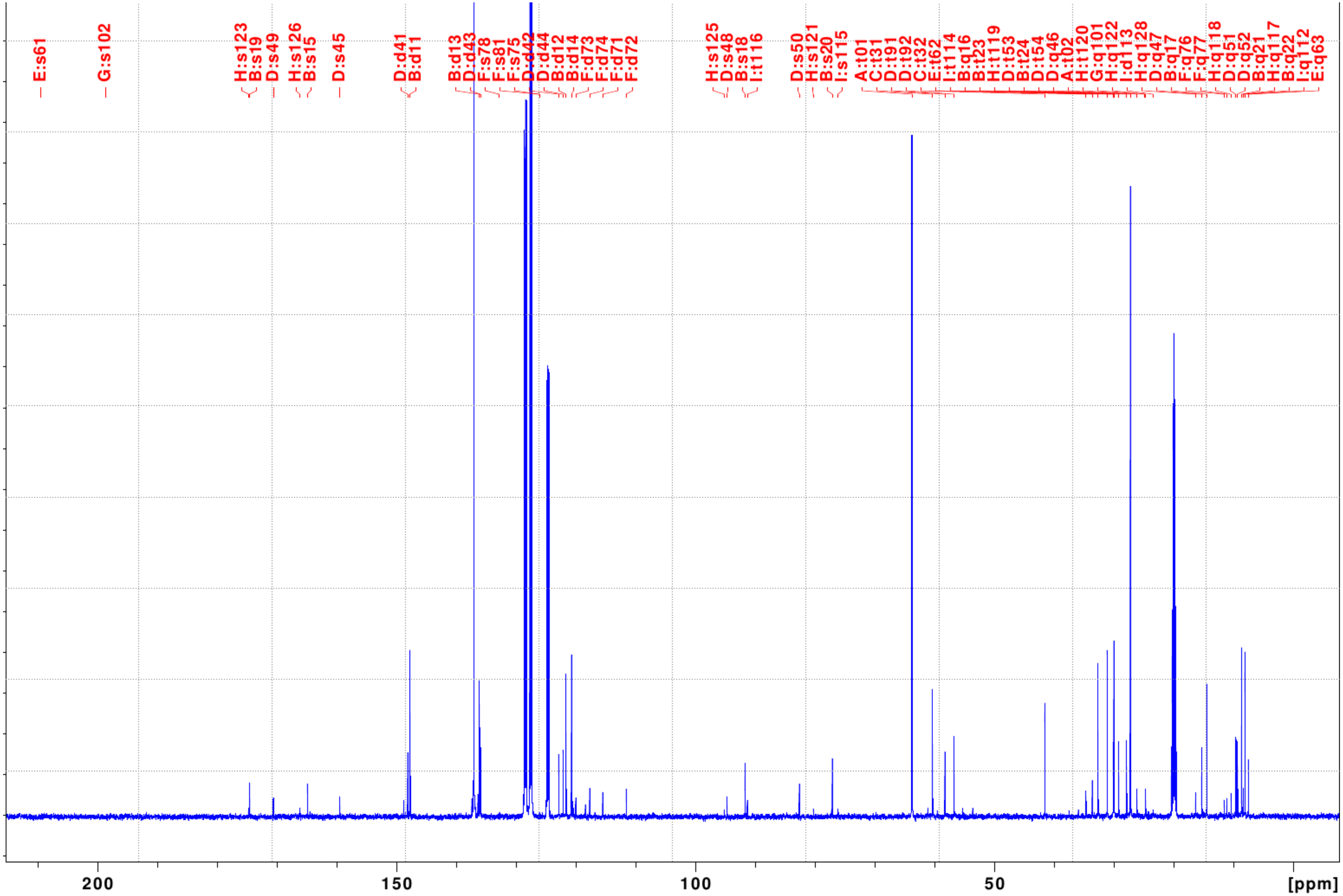

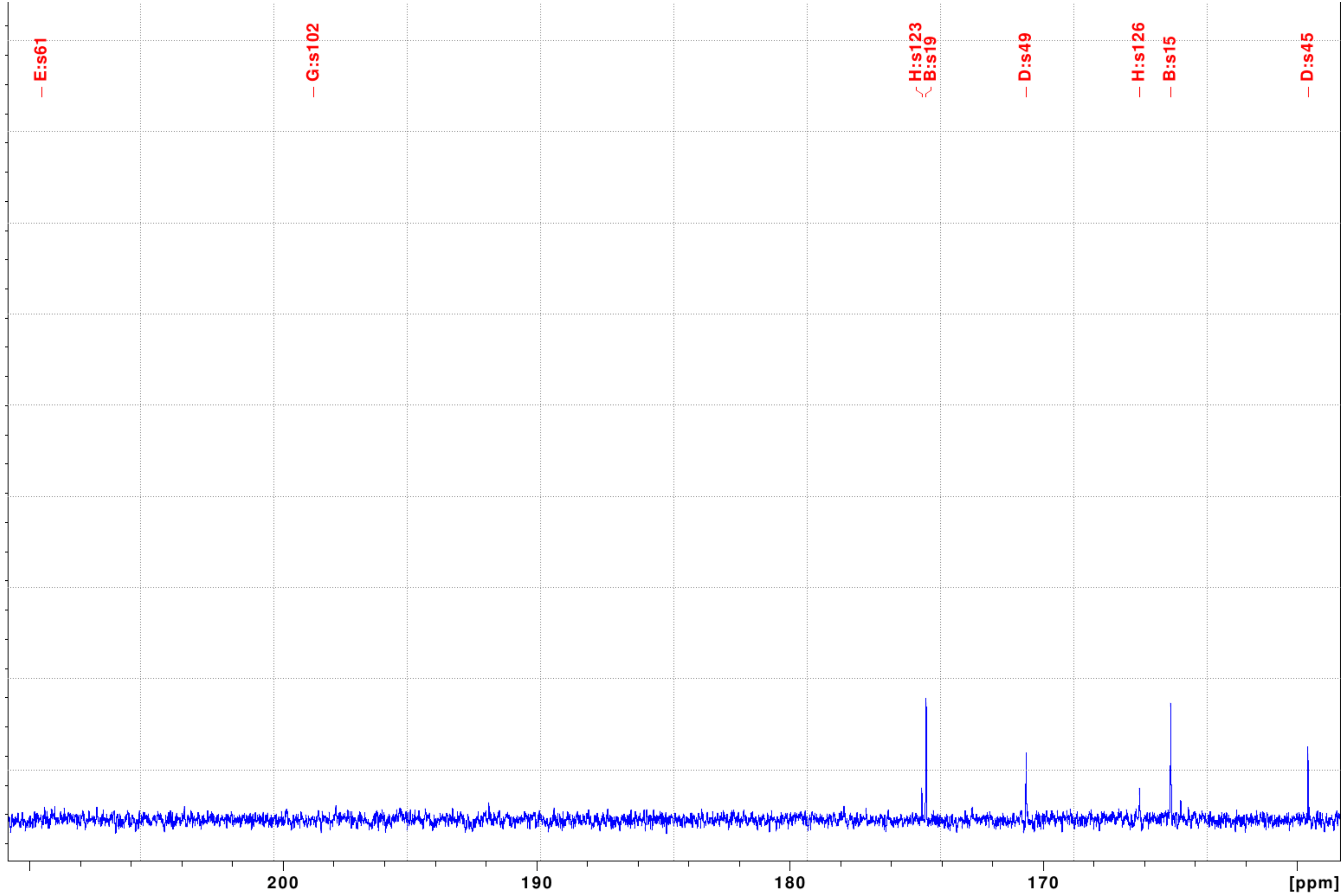

E:s61
G:s102
H:s123
B:s19
D:s49
H:s126
B:s15
D:s45
200
190
180
170
[ppm]

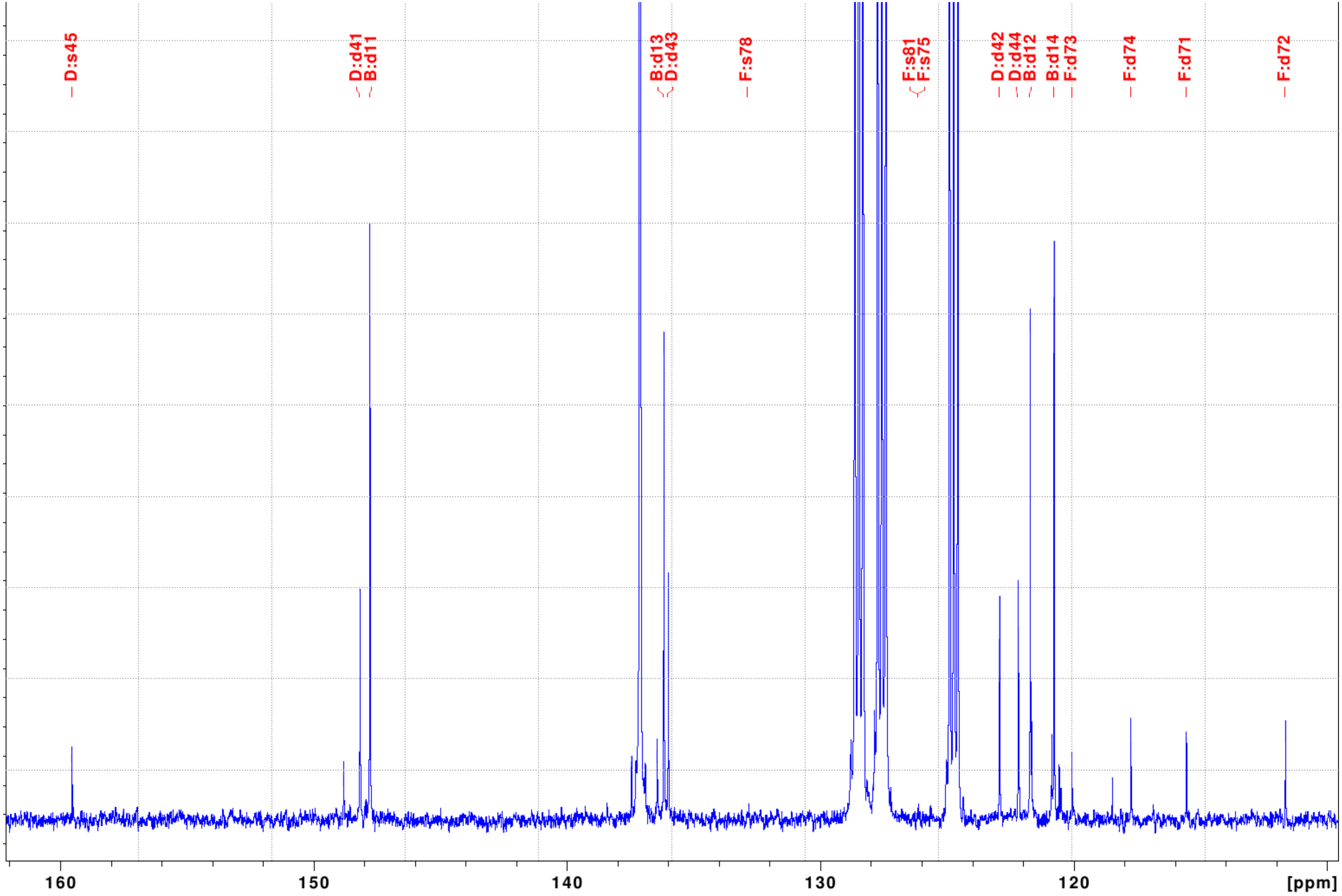

D:s45
D:d41
B:d11
B:d13
D:d43
F:s78
F:s81
F:s75
D:d42
D:d44
B:d12
B:d14
F:d73
F:d74
F:d71
F:d72
160
150
140
130
120
[ppm]

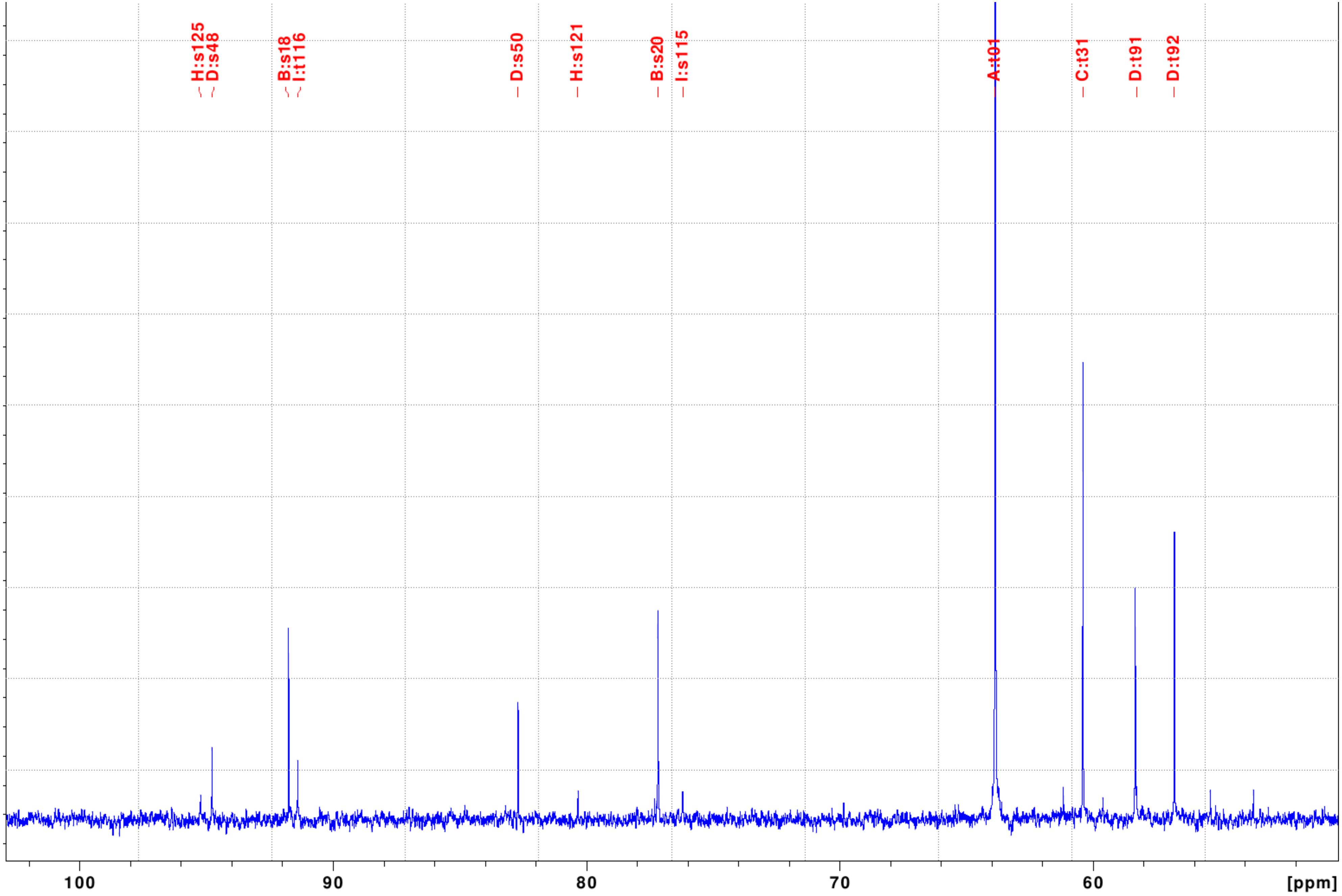
H:s125
D:s48
B:s18
I:t116
D:s50
H:s121
B:s20
I:s115
A:t01
C:t31
D:t91
D:t92
100
90
80
70
60
[ppm]

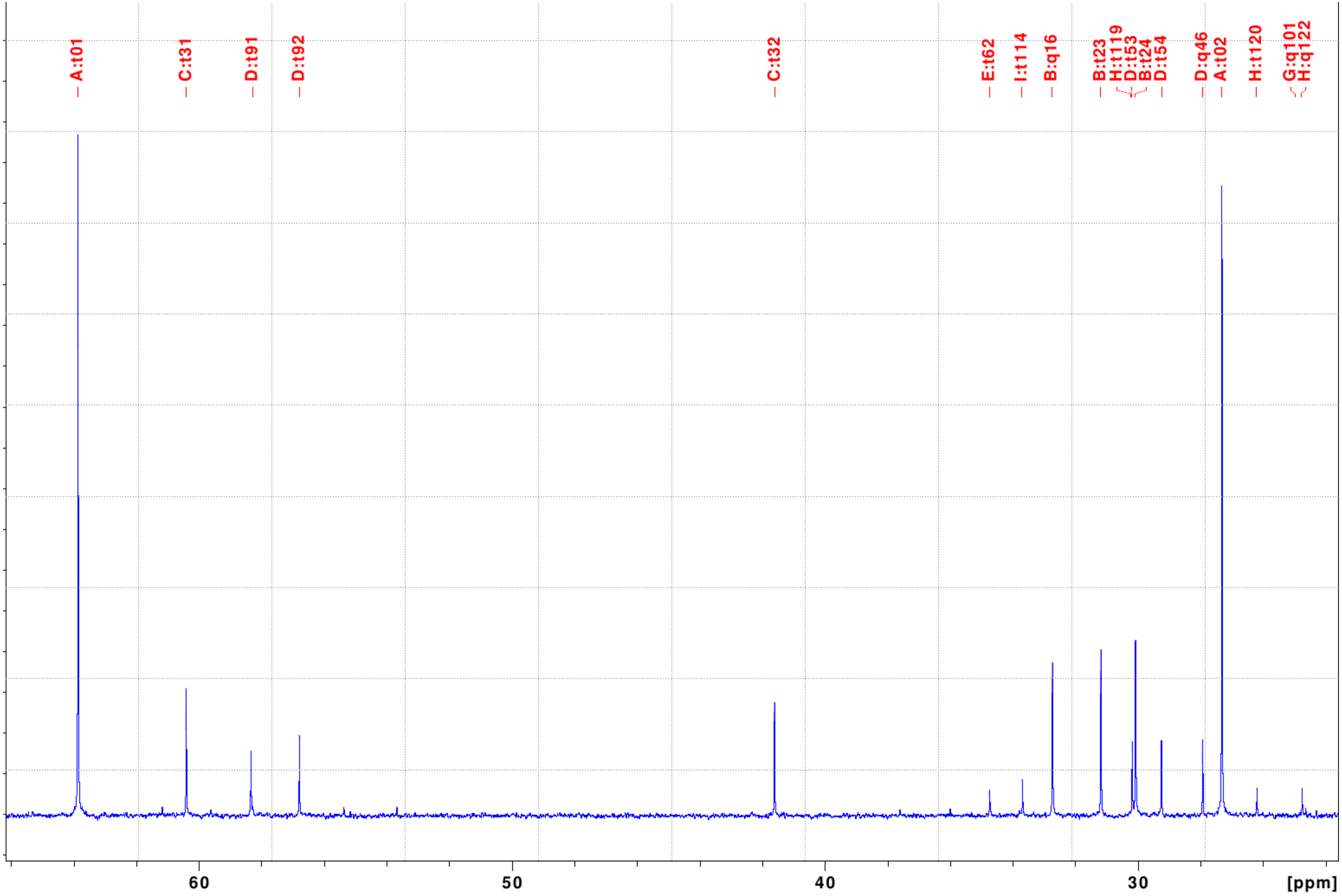

A:t01
C:t31
D:t91
D:t92
C:t32
E:t62
I:t114
B:q16
B:t23
H:t119
D:t53
B:t24
D:t54
D:q46
A:t02
H:t120
G:q101
H:q122
60
50
40
30
[ppm]

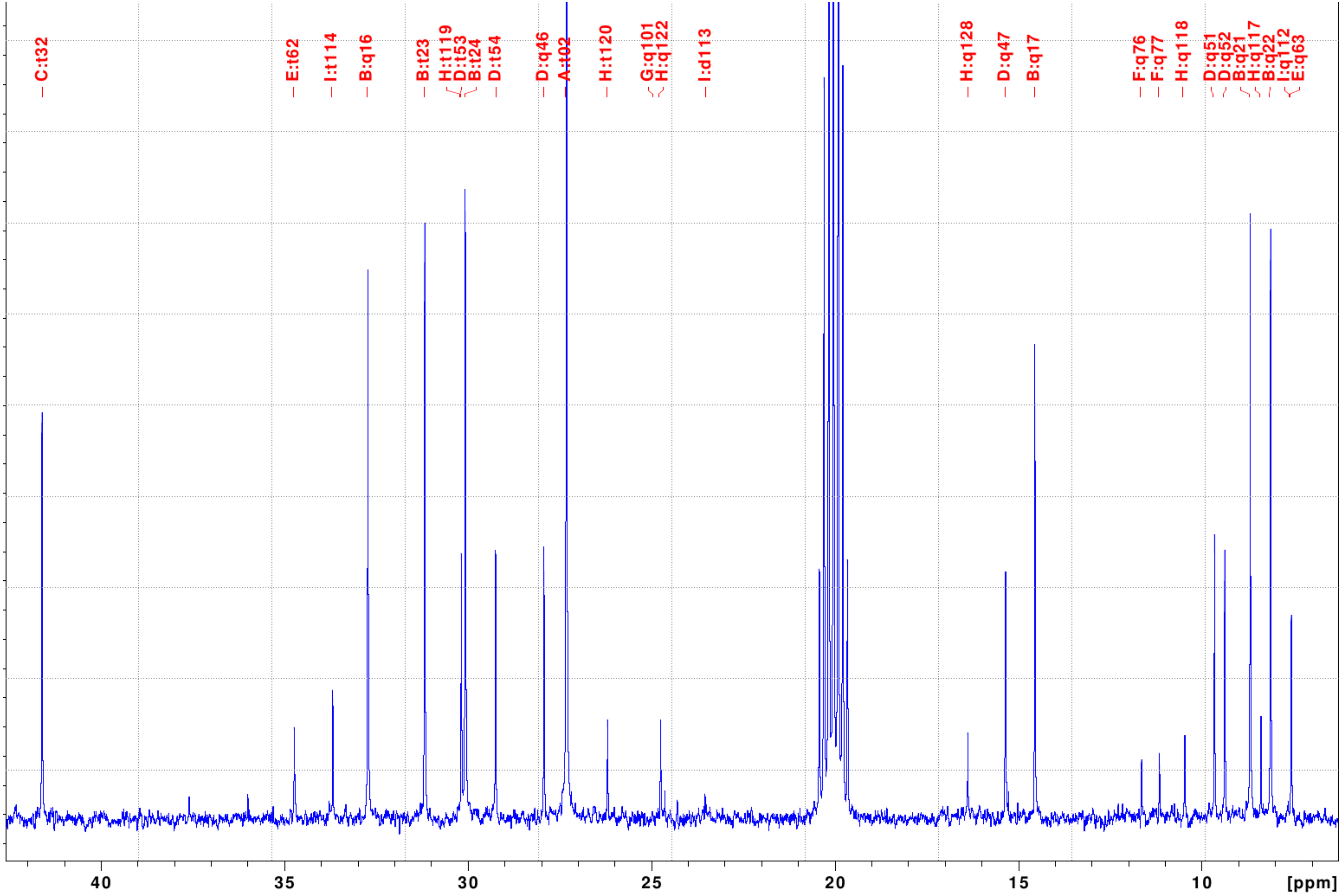

C:t32
E:t62
I:t114
B:q16
B:t23
H:t119
D:t53
B:t24
D:t54
D:q46
A:t02
H:t120
G:q101
H:q122
I:d113
H:q128
D:q47
B:q17
F:q76
F:q77
H:q118
D:q51
D:q52
B:q21
H:q117
B:q22
I:q112
E:q63
40
35
30
25
20
15
10
[ppm]

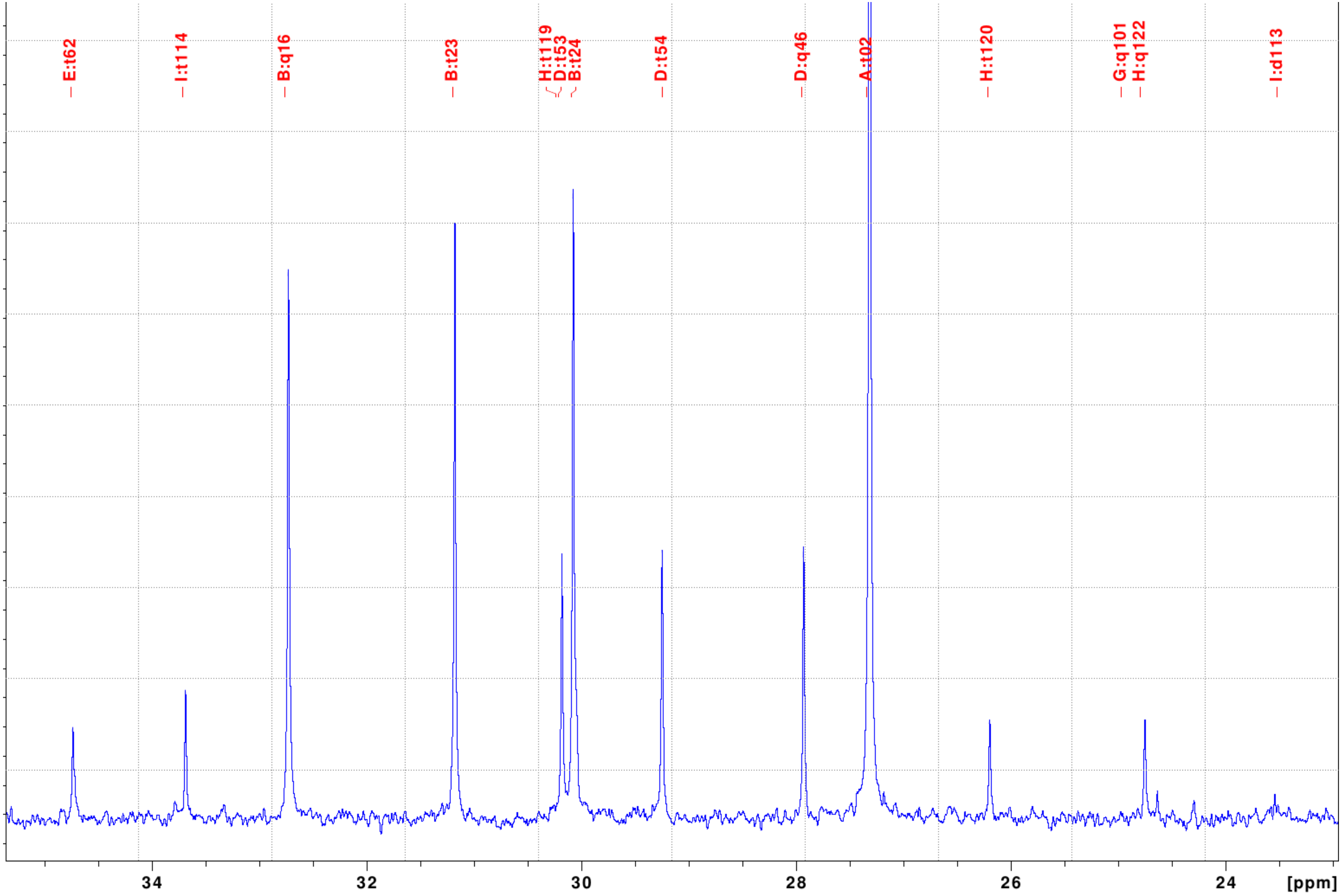

E:t62
I:t114
B:q16
B:t23
H:t119
D:t53
B:t24
D:t54
D:q46
A:t02
H:t120
G:q101
H:q122
I:d113
34
32
30
28
26
24
[ppm]

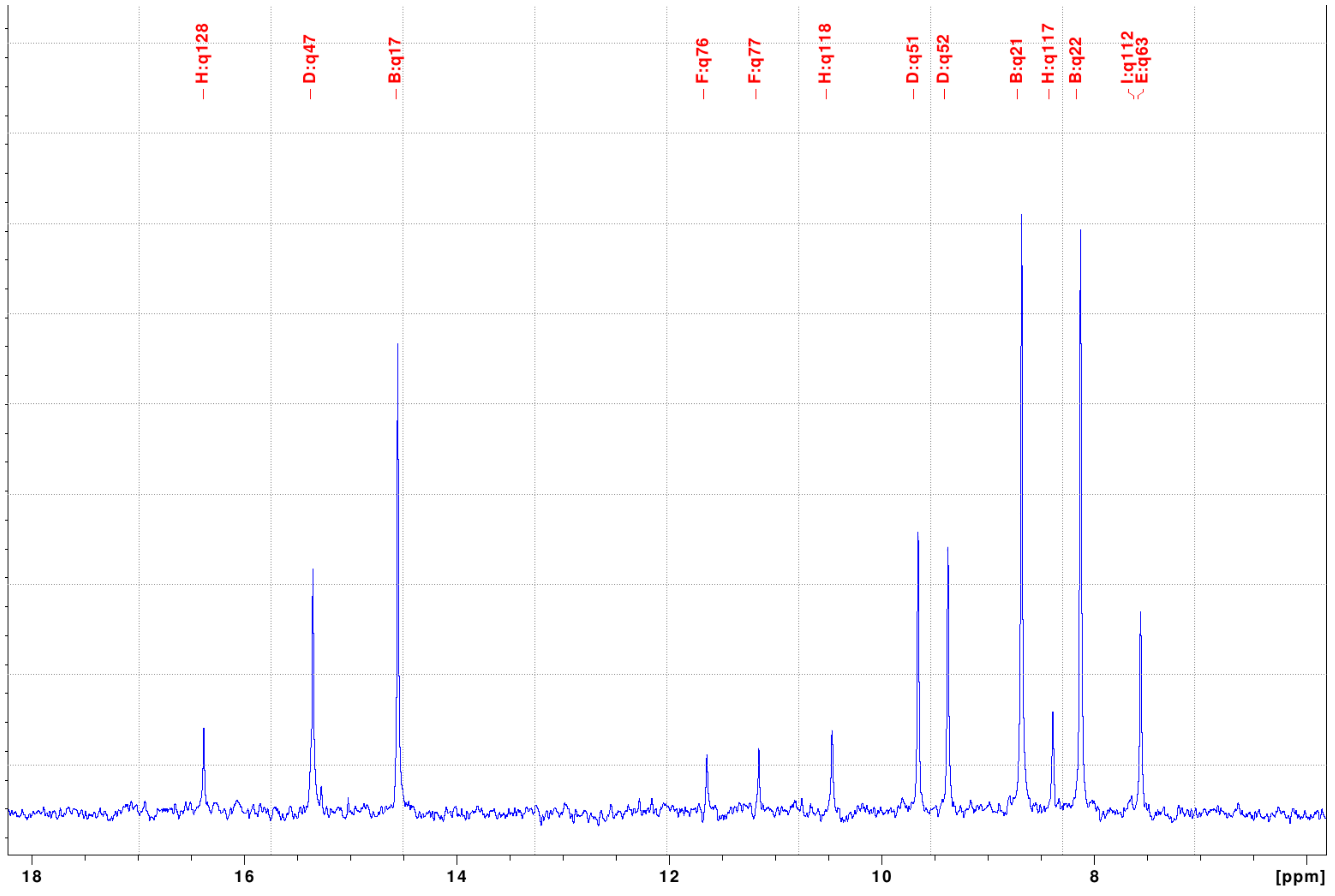
H:q128
D:q47
B:q17
F:q76
F:q77
H:q118
D:q51
D:q52
B:q21
H:q117
B:q22
I:q112
E:q63
18
16
14
12
10
8
[ppm]

^{1}H NMR spectrum (600 MHz)

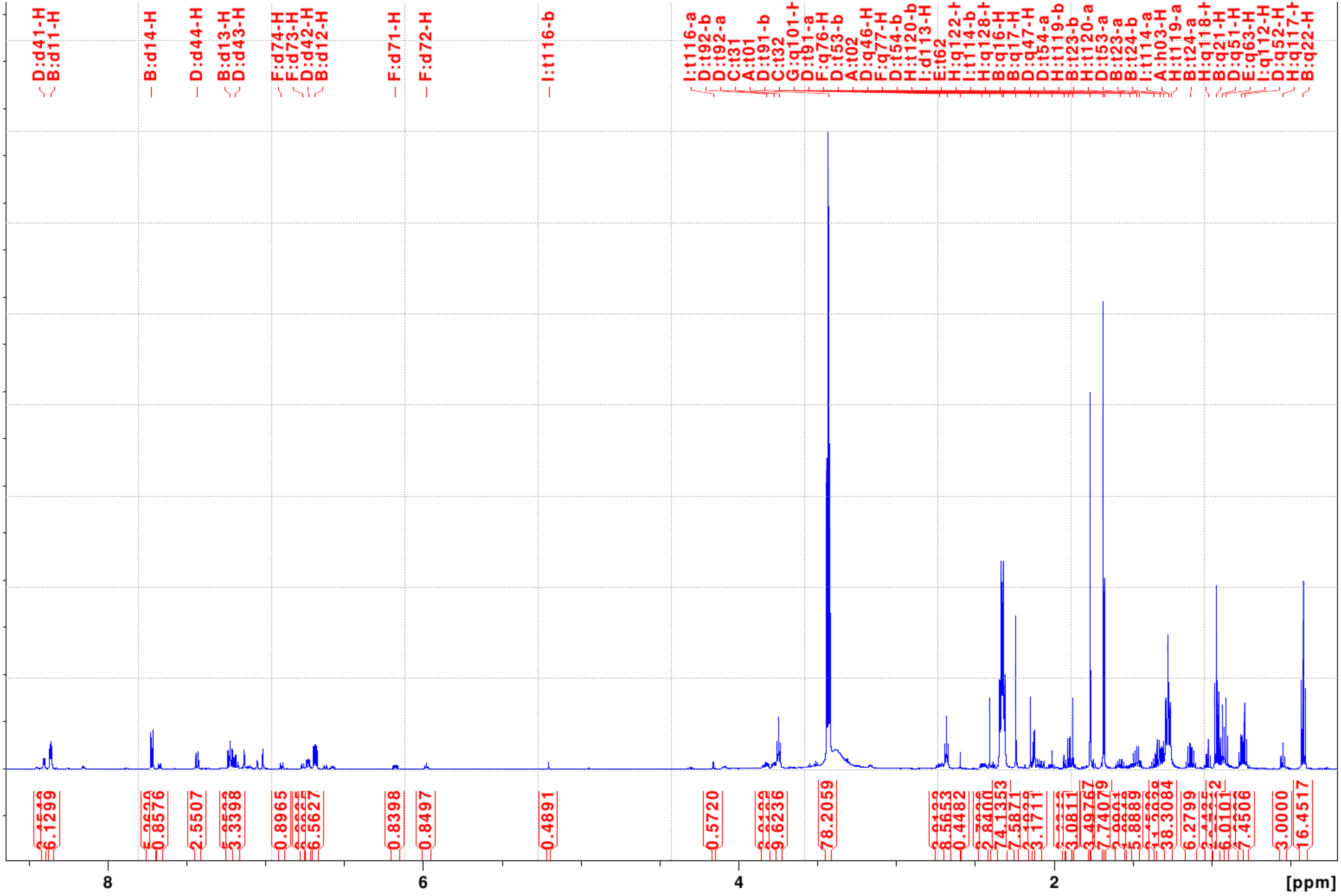

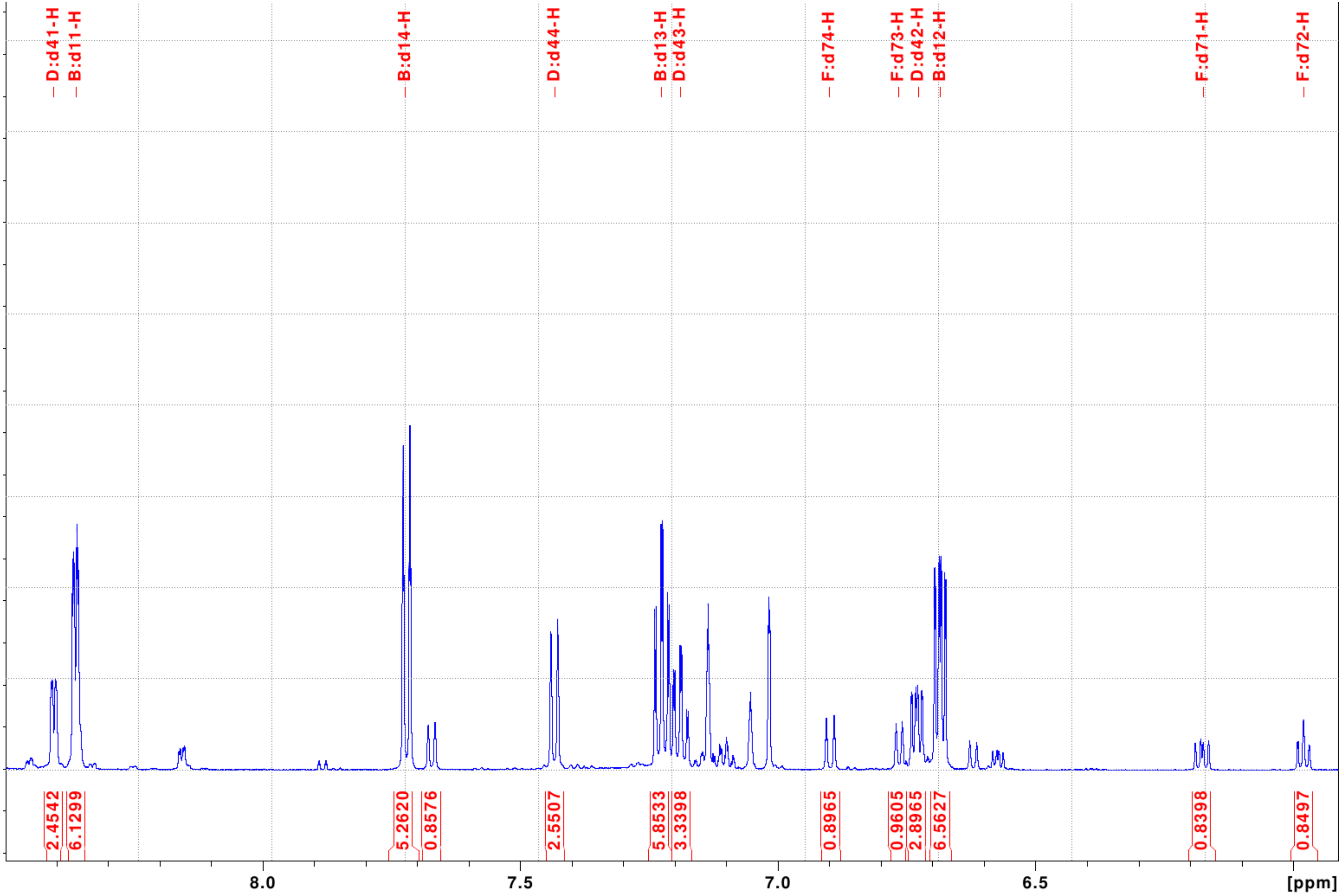

D:d41-H
B:d11-H
B:d14-H
D:d44-H
B:d13-H
D:d43-H
F:d74-H
F:d73-H
D:d42-H
B:d12-H
F:d71-H
F:d72-H
2.4542
6.1299
5.2620
0.8576
2.5507
5.8533
3.3398
0.8965
0.9605
2.8965
6.5627
0.8398
0.8497
8.0
7.5
7.0
6.5
[ppm]

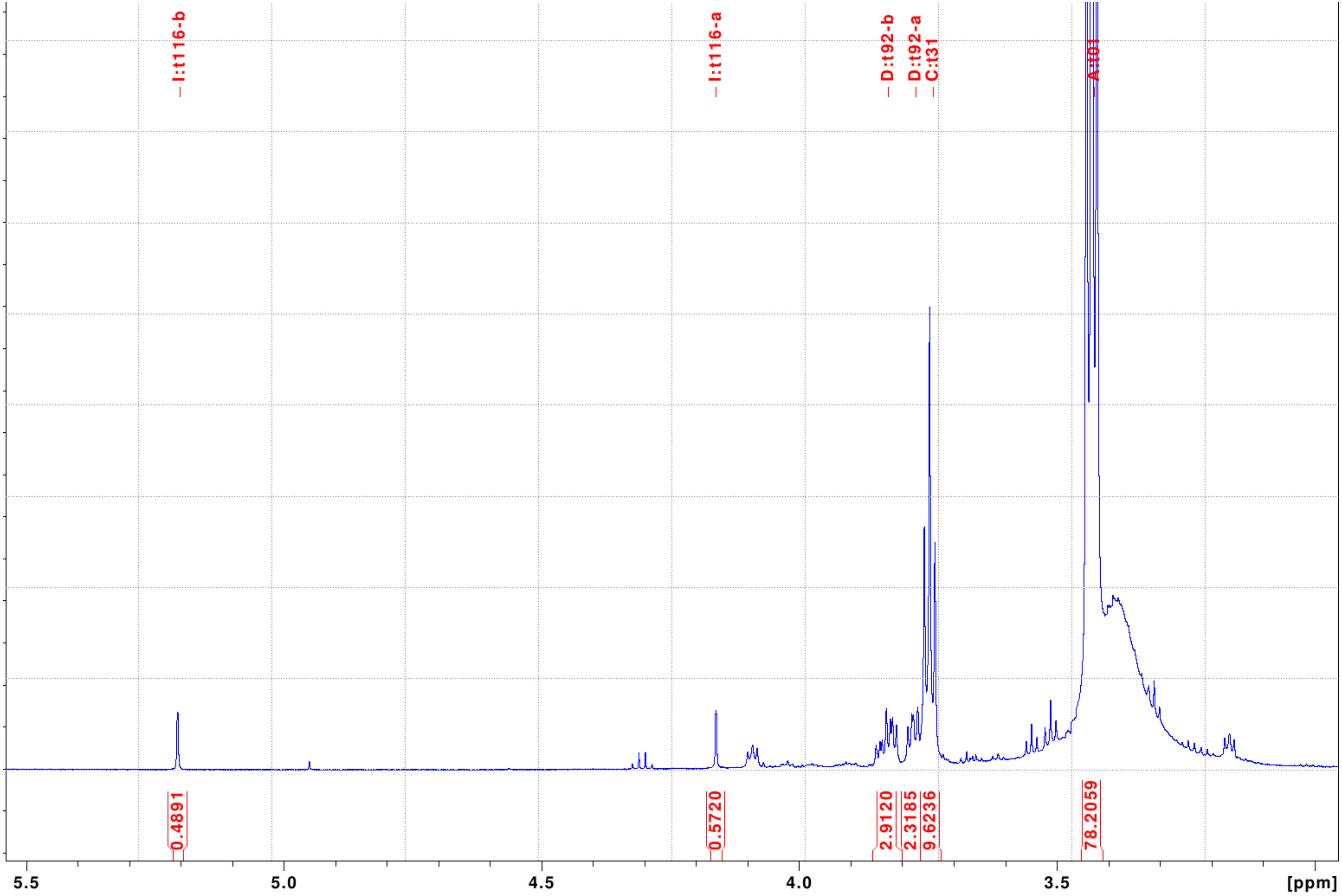

I:t116-b
I:t116-a
D:t92-b
D:t92-a
C:t31
A:t01
0.4891
0.5720
2.9120
2.3185
9.6236
78.2059
5.5
5.0
4.5
4.0
3.5
[ppm]

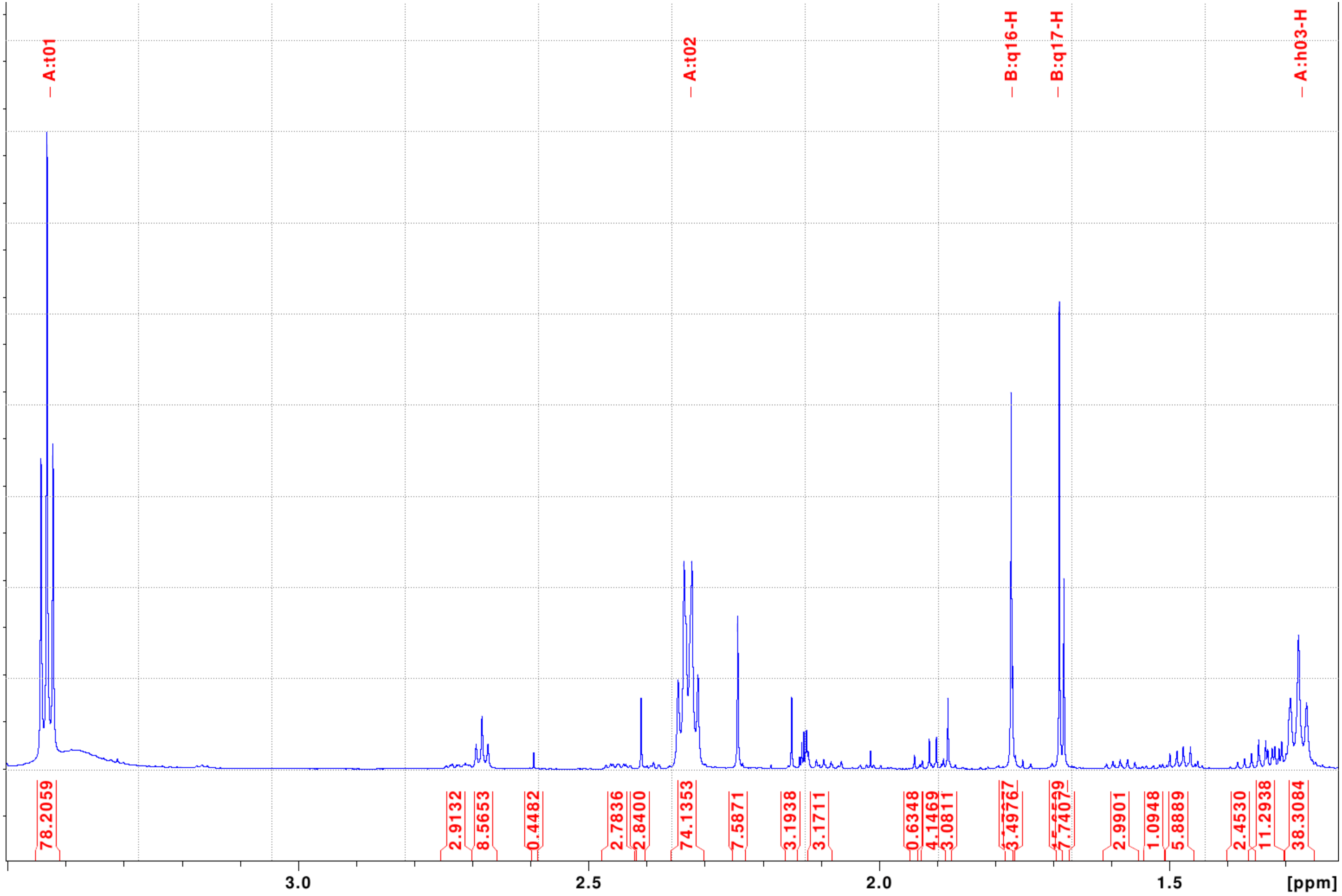
A:t01
A:t02
B:q16-H
B:q17-H
A:h03-H
78.2059
2.9132
8.5653
0.4482
2.7836
2.8400
74.1353
7.5871
3.1938
3.1711
0.6348
4.1469
3.0811
3.4976
7.7407
2.9901
1.0948
5.8889
2.4530
11.2938
38.3084
3.0
2.5
2.0
1.5
[ppm]

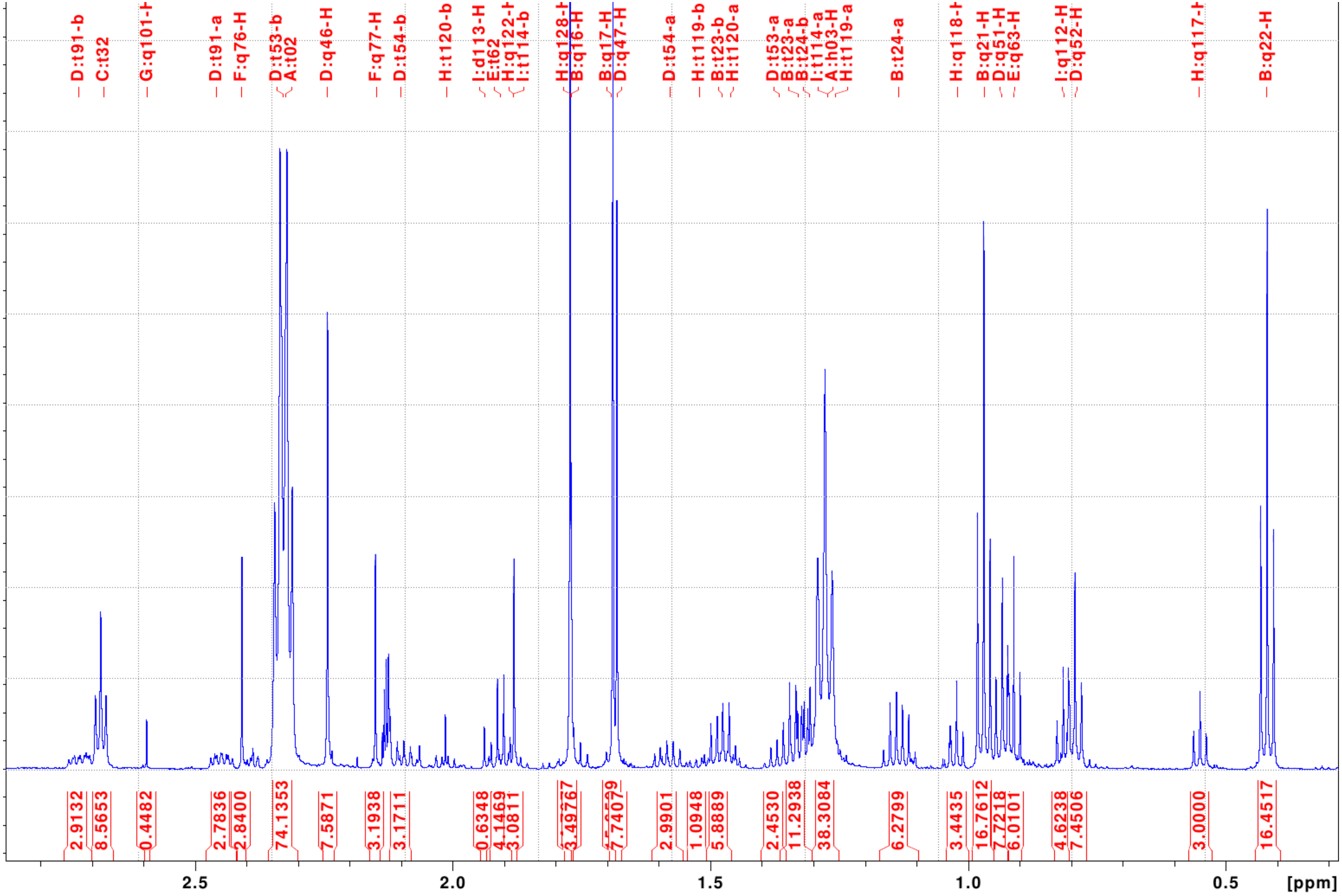

D:t91-b
C:t32
D:t91-a
F:q76-H
D:t53-b
A:t02
D:q46-H
F:q77-H
D:t54-b
H:t120-b
I:d113-H
E:t62
D:t54-a
H:t119-b
B:t23-b
H:t120-a
D:t53-a
B:t23-a
B:t24-b
I:t114-a
A:h03-H
H:t119-a
B:t24-a
B:q21-H
D:q51-H
E:q63-H
I:q112-H
D:q52-H
B:q22-H
2.9132
8.5653
0.4482
2.7836
2.8400
74.1353
7.5871
3.1938
3.1711
0.6348
3.4976
7.7407
2.9901
1.0948
5.8889
2.4530
11.2938
38.3084
6.2799
3.4435
16.7612
7.7218
6.0101
4.6238
7.4506
3.0000
16.4517
2.5
2.0
1.5
1.0
0.5
[ppm]

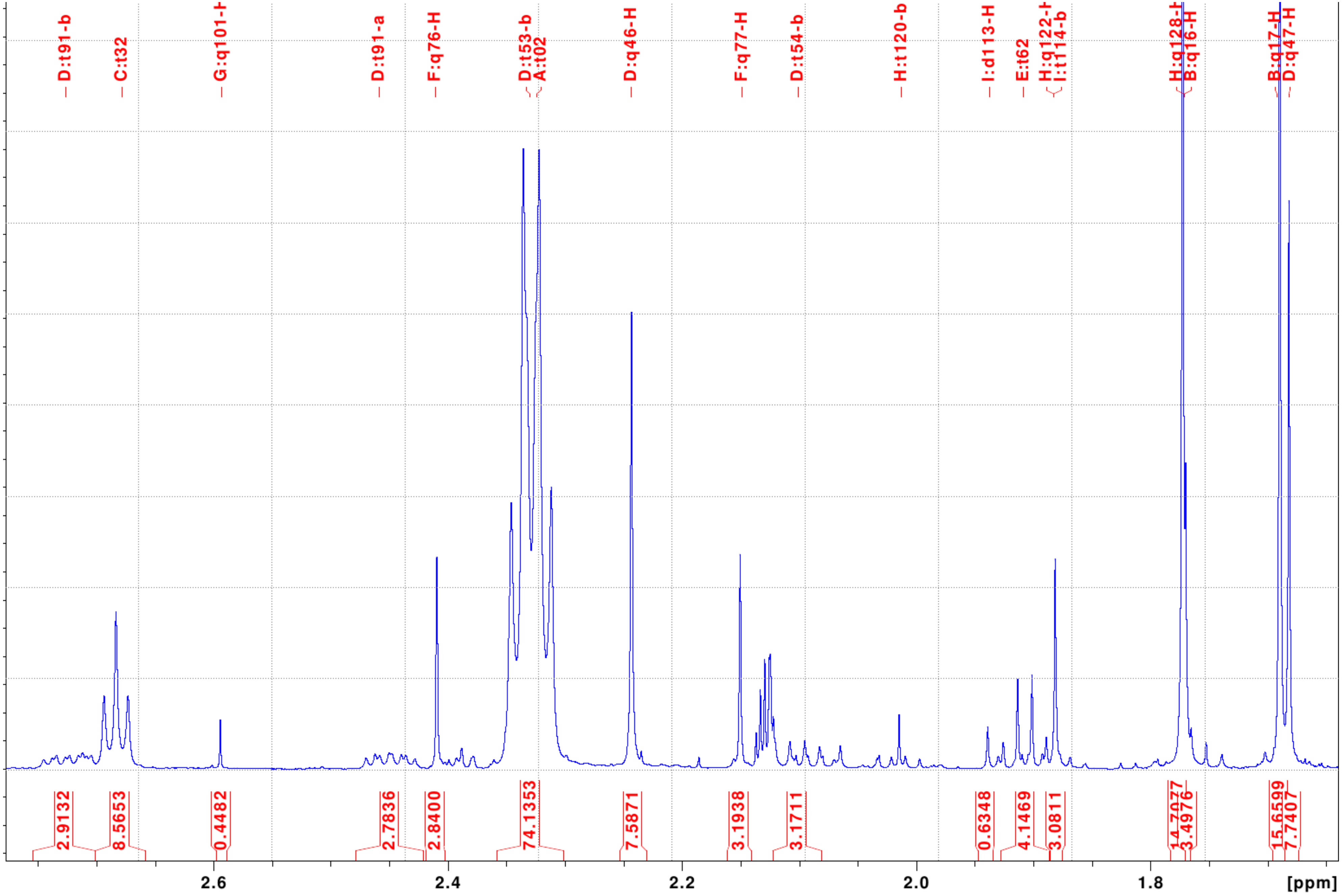
D:t91-b
C:t32
G:q101-H
D:t91-a
F:q76-H
D:t53-b
A:t02
D:q46-H
F:q77-H
D:t54-b
H:t120-b
I:d113-H
E:t62
H:q122-H
I:t114-b
H:q128-H
B:q16-H
B:q17-H
D:q47-H
2.9132
8.5653
0.4482
2.7836
2.8400
74.1353
7.5871
3.1938
3.1711
0.6348
4.1469
3.0811
14.7077
3.4976
15.6599
7.7407
2.6
2.4
2.2
2.0
1.8
[ppm]

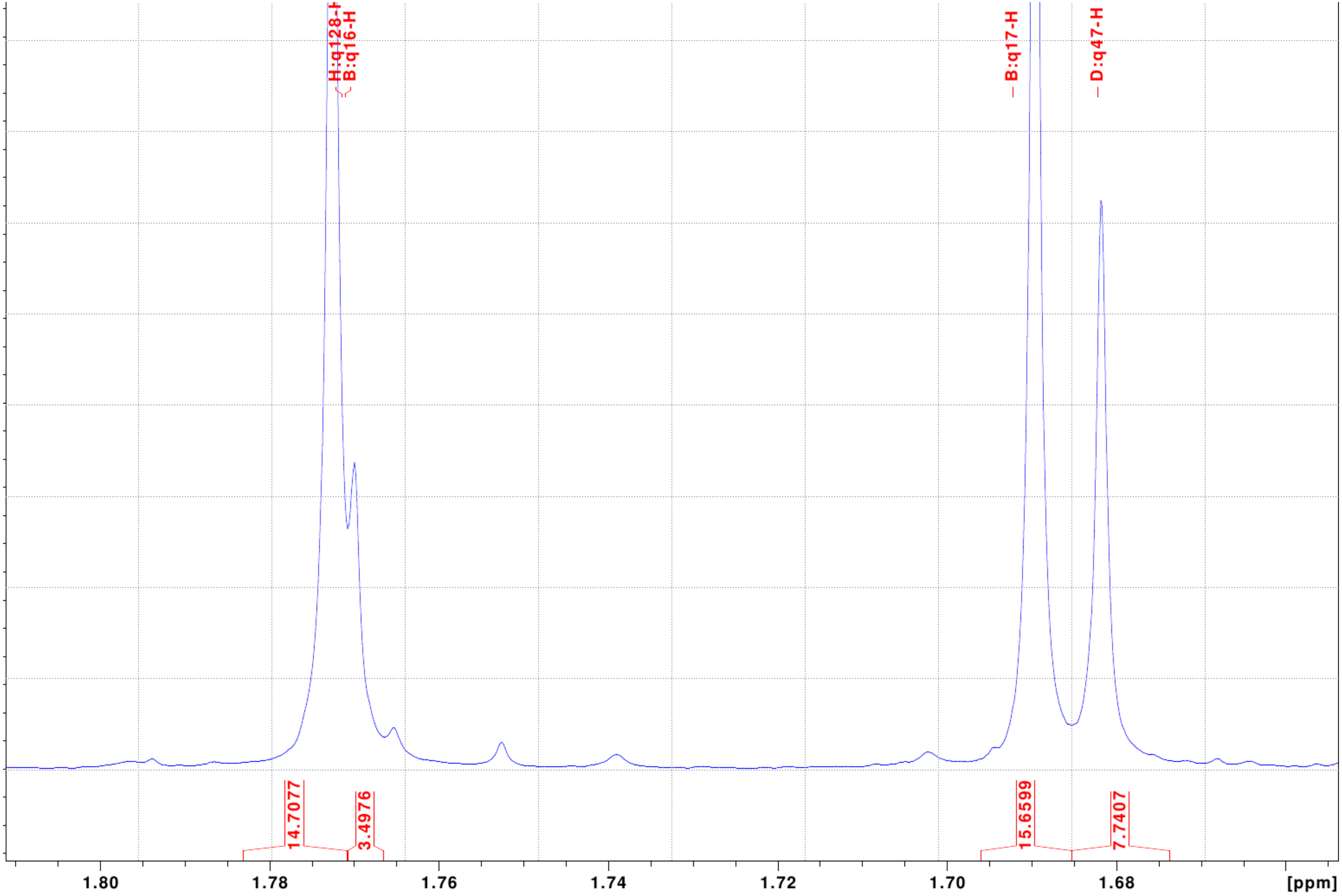
H:q128-H
B:q16-H
B:q17-H
D:q47-H
14.7077
3.4976
15.6599
7.7407
1.80
1.78
1.76
1.74
1.72
1.70
1.68
[ppm]

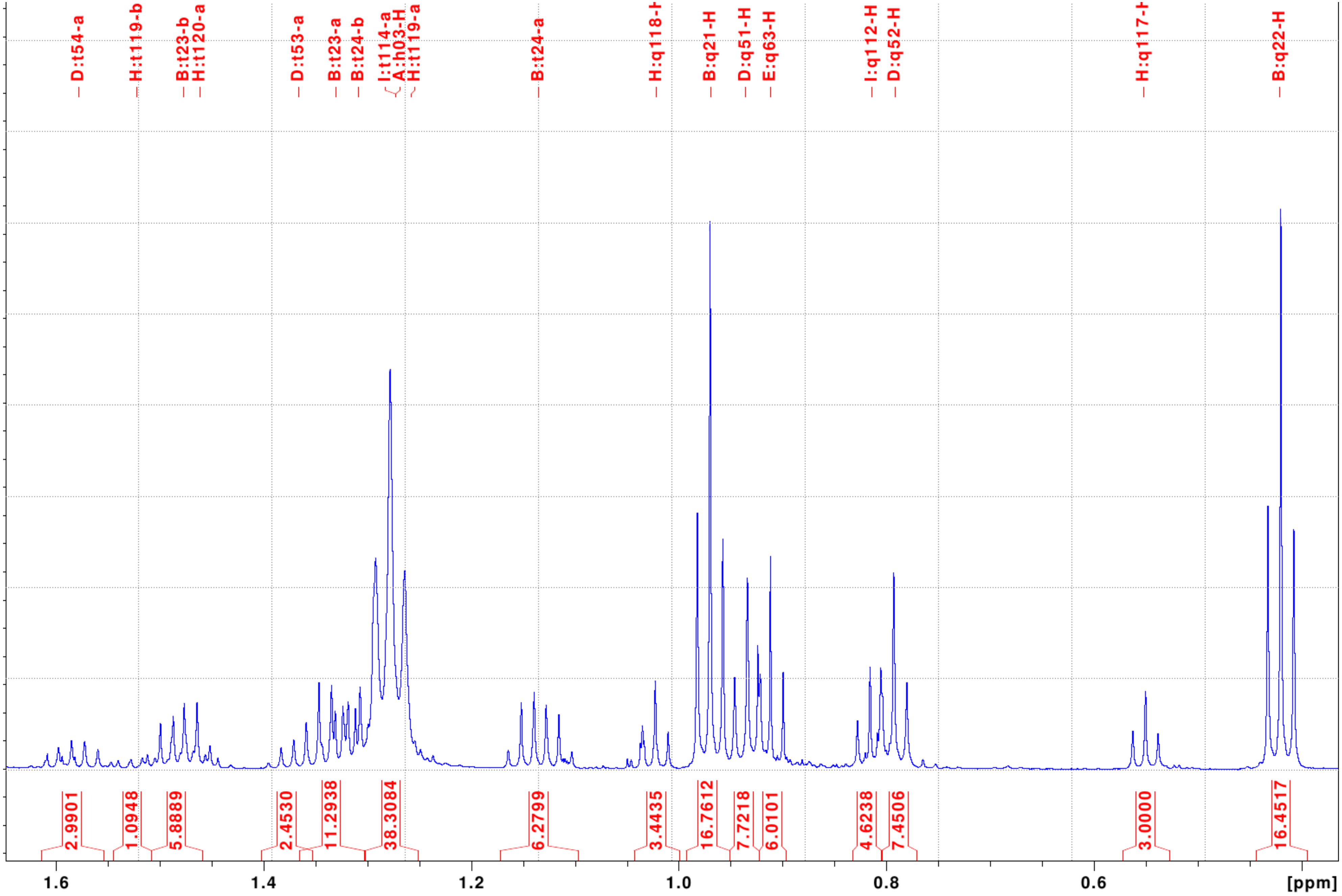
D:t54-a
H:t119-b
B:t23-b
H:t120-a
D:t53-a
B:t23-a
B:t24-b
I:t114-a
A:h03-H
H:t119-a
B:t24-a
H:q118-H
B:q21-H
D:q51-H
E:q63-H
I:q112-H
D:q52-H
H:q117-H
B:q22-H
2.9901
1.0948
5.8889
2.4530
11.2938
38.3084
6.2799
3.4435
16.7612
7.7218
6.0101
4.6238
7.4506
3.0000
16.4517
1.6
1.4
1.2
1.0
0.8
0.6
[ppm]

^{15}N NMR spectrum

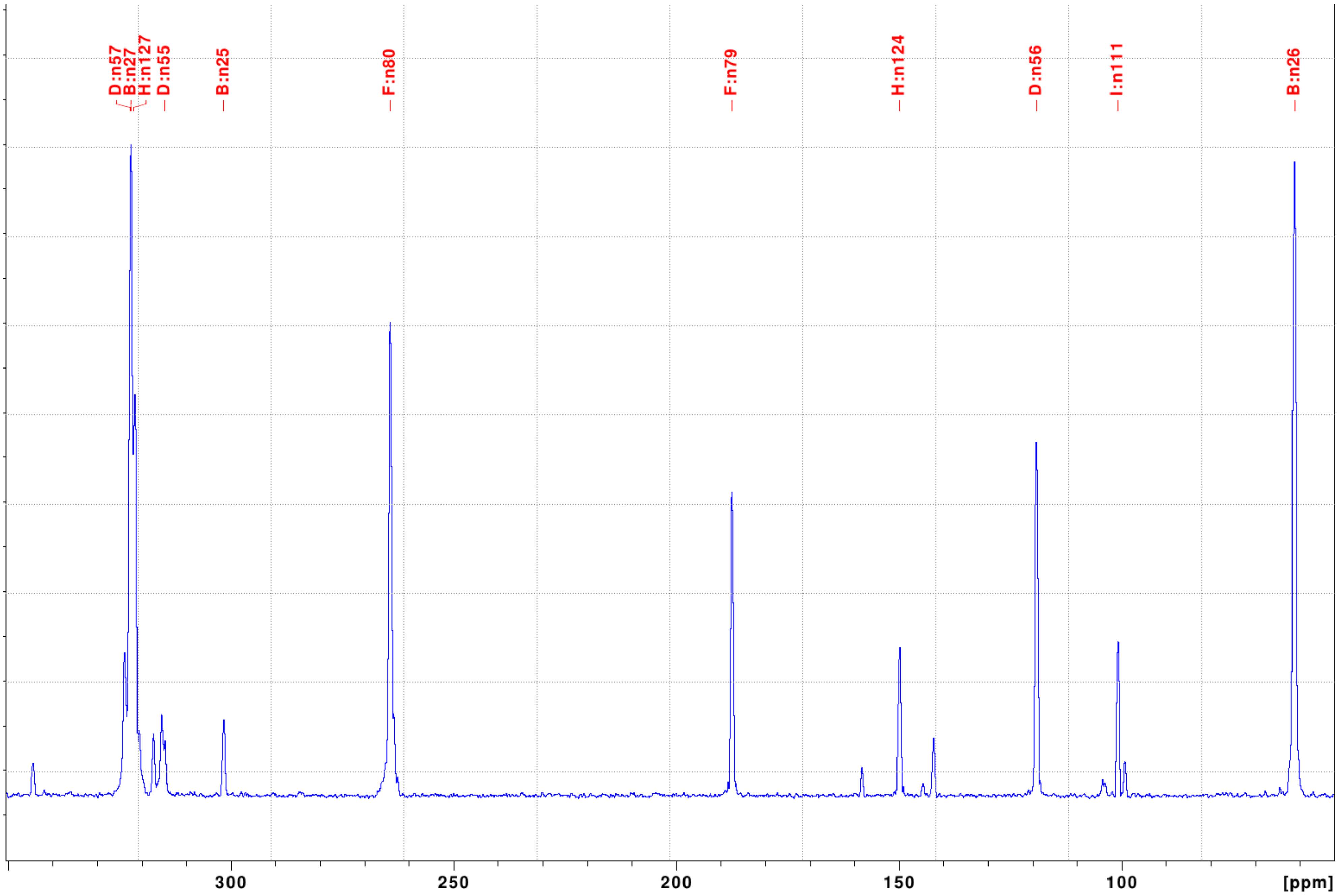

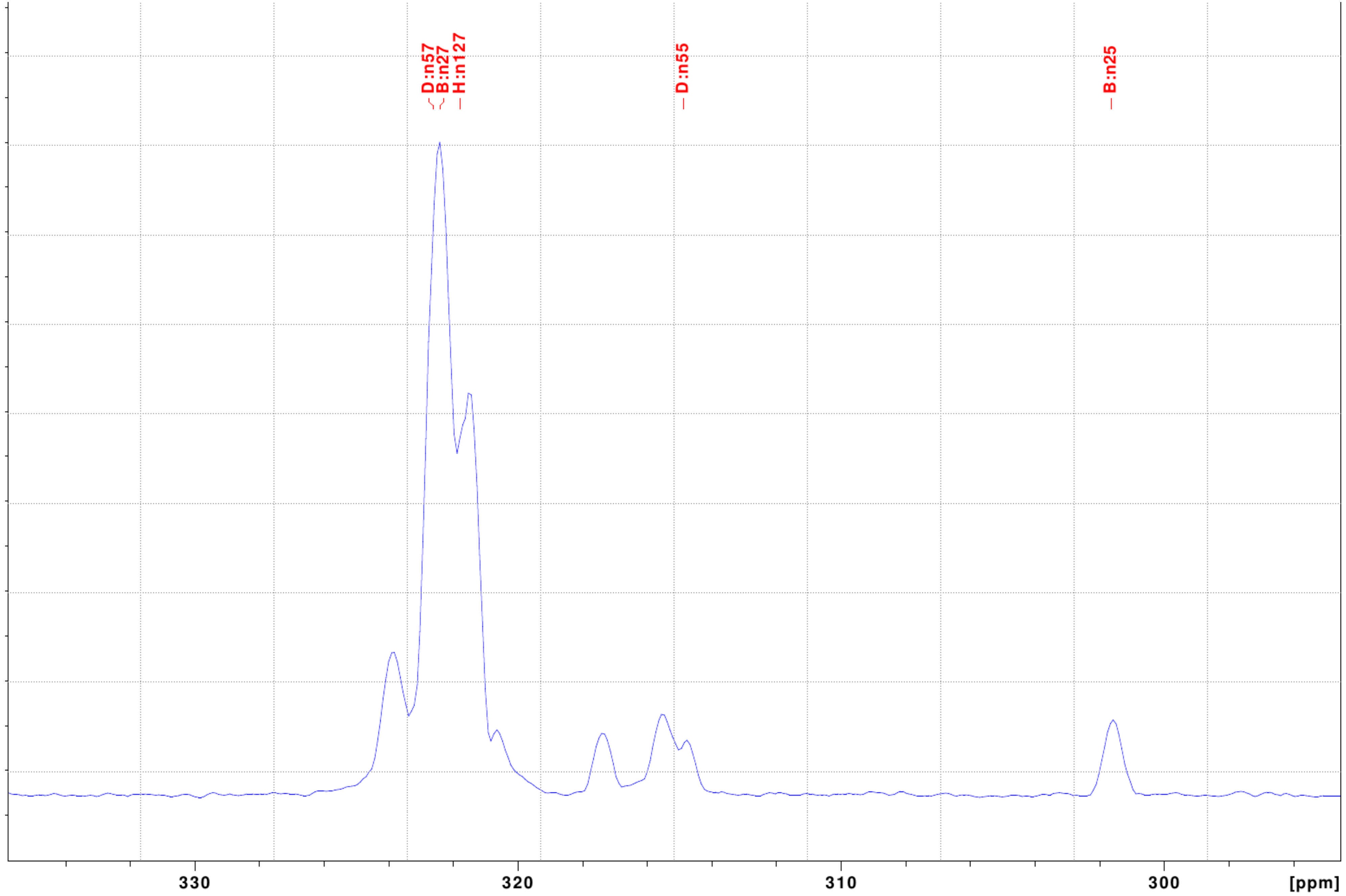
D:n57
B:n27
H:n127
D:n55
B:n25
330
320
310
300
[ppm]

Inversion of nitrogen, $2^{RS/SR}$i⇄$2^{RS/SR}$ (A⇄B) in $CDCl_3$

NOESY spectrum of A and B mixture at 38 °C
(d50-H,d20-H signals, τ = 0.1 s, see p. 181 for formula)

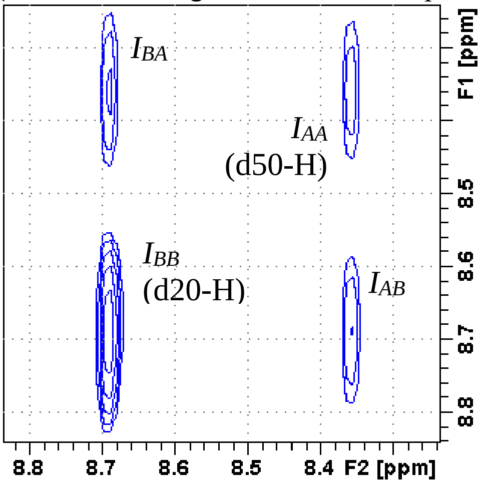

^{1}H DNMR spectra of A and B mixture
(d39-H,d09-H signals)

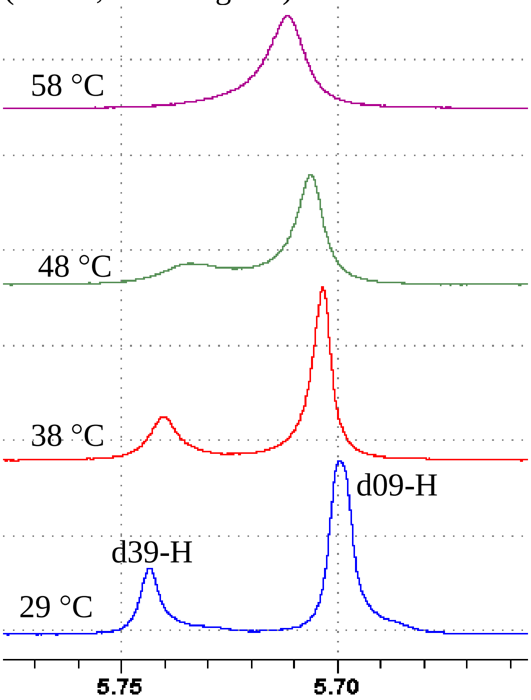

Line shape analysis: xsim
[ftp://nmr.nioch.nsc.ru/pub/nmr/]

Rate constants from NOESY and DNMR

°C	Method	k_{AB}, s^{-1}	ΔG#, kcal/mol	K = [B]/[A]	ΔG_0, kcal/mol
-8.1	NOESY	0.14	16.48	3.05	-0.59
4.6	NOESY	0.47	16.63	3.01	-0.61
16.9	NOESY	1.34	16.79	2.93	-0.62
28.7	NOESY	3.52	16.92	2.88	-0.63
38.4	NOESY	10.01	16.84	3.01	-0.68
28.6	DNMR	2.46	17.13	2.49	-0.55
38.3	DNMR	8.84	16.91	2.59	-0.59
48.1	DNMR	21.95	16.88	2.70	-0.63
57.7	DNMR	71.73	16.63	2.63	-0.63

Enthalpy and entropy of activation

Eyring equation
$\Delta G^{\#} = \Delta H^{\#} - T\Delta S^{\#} = -RT\,(\ln(k_{AB}/T) - \ln(æ/h))$
(transmission coeff. = 1, R = 1.987, ln(æ/h)=23.76)

$\Delta H^{\#}$ = 15.31±0.92 kcal/mol
$\Delta S^{\#}$ = -5.0±3.1 cal/mol/K
(1 sigma)

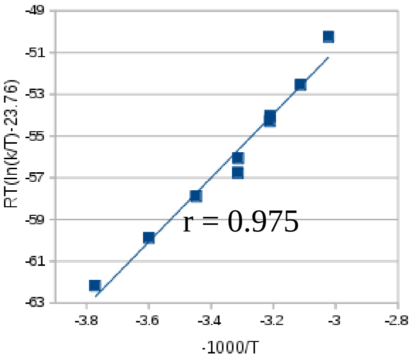

Enthalpy and entropy of the reaction

$\Delta G_0 = \Delta H_0 - T\Delta S_0 = -RT \ln K$

ΔH_0 = −0.41±0.18 kcal/mol
ΔS_0 = 0.69±0.61 cal/mol/K
(1 sigma)

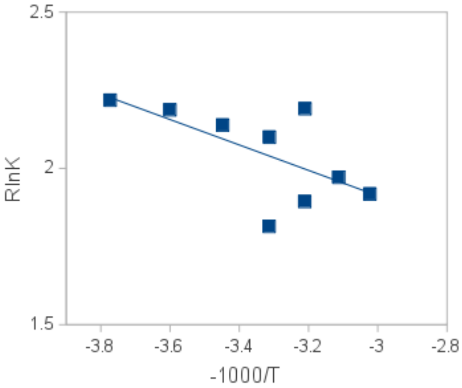

Epimerization of diastereomers $2^{RS/SR}$ (A) and $2^{RR/SS}$ (B) in DMSO-d_6

Rate constants from NOE at 117÷176 °C

Formula for rate constants from NOE (see http://nmr.nioch.nsc.ru/noekin/node5.html)

$$k_{AB}=\frac{K}{(K+1)\,\tau}\ln\frac{I_{AA}+KI_{BB}+(K+1)\,I_{cross}}{I_{AA}+KI_{BB}-(K+1)\,I_{cross}}$$

$$I_{cross} = (I_{AB}+I_{BA})/2$$

NOESY spectrum of A and B mixture at 156 °C
(CH region, τ = 1 s)

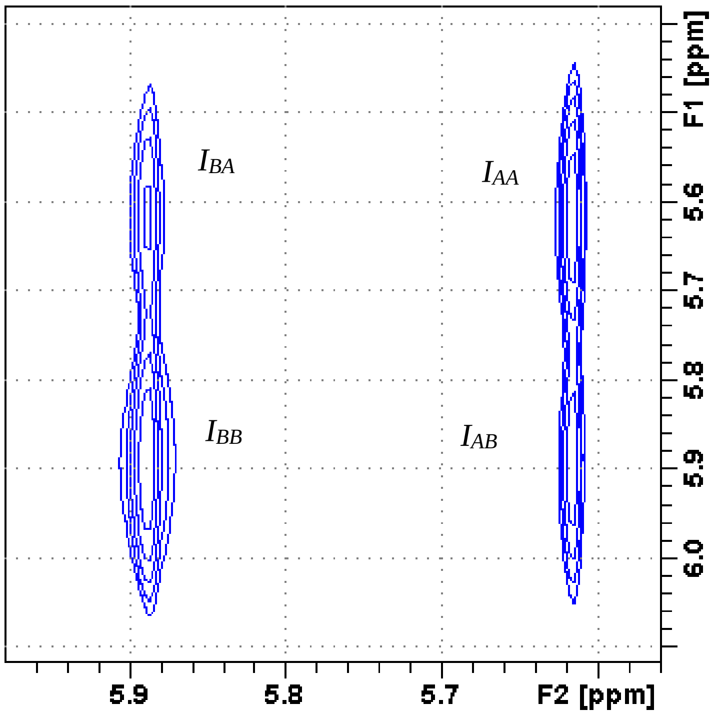

Rate and equilibrium constants

Temperature, °C	k_{AB}, s^{-1}	K [a]
175.7	2.5664	1.29
165.6	0.7966	1.32
156.2	0.5336	1.33
146.3	0.2043	1.30
136.6	0.0839	1.29
126.9	0.0393	1.28
117.3	0.0184	1.32
155.3 [b]	0.6026	1.32
145.8 [b]	0.2430	1.32
136.4 [b]	0.0952	1.27
127.1 [b]	0.0458	1.23
117.9 [b]	0.0133	1.17

[a] K values are taken from 1D spectra
[b] Concentration of (A+B) is 32 times less

Kinetics A⇄B ($2^{RS/SR}$⇄$2^{RR/SS}$) at 69 °C in DMSO-d_6

^{1}H NMR spectrum (CH region) at the end of kinetics (expno 78).

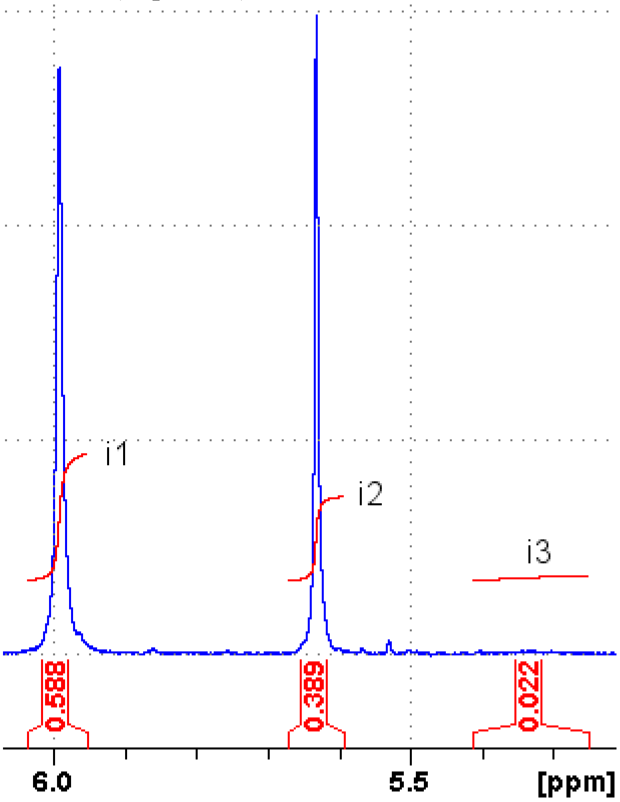

A⇄B $K=k_{AB}/k_{BA}$

[A] = i2/(i1+i2+i3), [B] = 1-[A], i3 = i1/25

kinetics_data.txt

expno	time,s	i1	i2
50	0	0.0303	0.9697
51	153	0.0423	0.9577
52	487	0.0586	0.9414
53	1124	0.0926	0.9074
54	1685	0.1244	0.8756
55	2277	0.1530	0.8470
56	3309	0.2020	0.7980
57	4531	0.2529	0.7471
58	5945	0.3058	0.6942
59	7549	0.3557	0.6443
60	9345	0.4027	0.5973
61	11332	0.4434	0.5566
62	13509	0.4803	0.5197
63	15878	0.5123	0.4877
64	18438	0.5377	0.4623
65	21188	0.5570	0.4430
66	24130	0.5721	0.4279
67	27263	0.5838	0.4162
68	30586	0.5933	0.4067
69	34101	0.6000	0.4000
70	37806	0.6043	0.3957
71	41703	0.6062	0.3938
72	45791	0.6086	0.3914
73	50069	0.6102	0.3898
74	54539	0.6111	0.3889
75	59201	0.6110	0.3890
76	64052	0.6120	0.3880
77	69095	0.6117	0.3883
78	74329	0.6126	0.3874

Output of our kinetic program

k_{AB} and K were used as fitted parameters (see next page for choice)

k_{AB} 6.638279e-05 *
K 1.588667e+00 *
SSD (sum of squares): 1.379229e-04
Standard deviation: 2.260145e-03
t_0 = -468 s (when [A]=1 and [B]=0)

Use of constant chi-square boundaries as confidence limits, nsigma=3
k_{AB} 6.638279e-05 ± 7.8e-07 (1.2%)
K 1.588667e+00 ± 1.4e-02 (0.9%)

Confidence limits by Monte Carlo simulation (bootstrap method)
200 synthetic data sets, nsigma = 3
k_{AB} 6.634291e-05 ± 7.7e-07 (1.2%)
K 1.590230e+00 ± 1.4e-02 (0.9%)

Kinetic curves

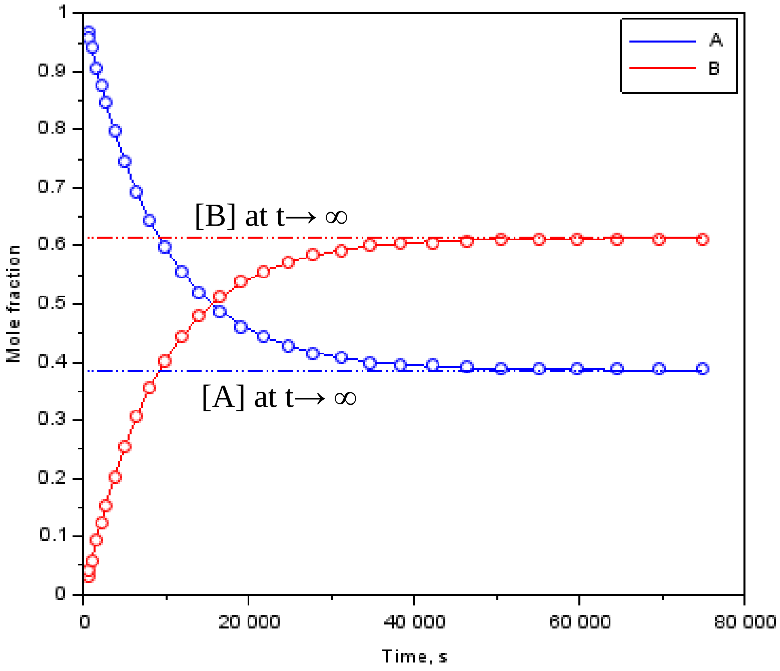

Kinetics A⇄B ($2^{RS/SR}$⇄$2^{RR/SS}$) at 69 °C in toluene-d_8

^{1}H NMR spectrum (CH region) at the end of kinetics (expno 175).

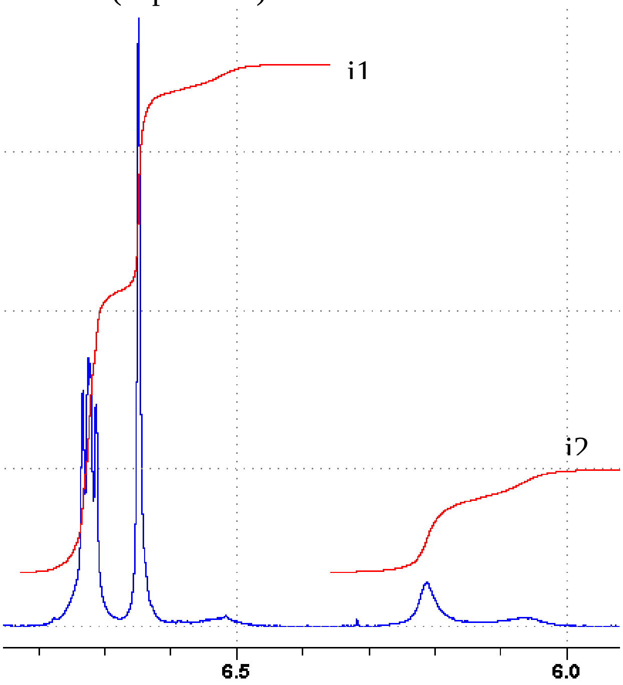

i1 = C_{py}-H(RR+RS) + C_{alk}-H(RR) + C_{py}(RS´)
i2 = C_{alk}-H(RS) + C_{alk}-H(RS´)
Normalization: i1 + i2 = 2

A⇄B K=k_{AB}/k_{BA}

[A] = i2, [B] = 1-i1

kin etics_data.txt

expno	time, s	[A]	[B]
142	0	0.8739	0.1261
143	867	0.8480	0.1520
144	1658	0.8221	0.1779
145	2391	0.8015	0.1985
146	3220	0.7788	0.2212
147	4147	0.7538	0.2462
148	5170	0.7274	0.2726
149	6291	0.7012	0.2988
150	7508	0.6717	0.3283
151	8823	0.6461	0.3539
152	10234	0.6200	0.3800
153	11743	0.5948	0.4052
154	13348	0.5674	0.4326
155	15051	0.5438	0.4562
156	16850	0.5190	0.4810
157	18747	0.4953	0.5047
158	20740	0.4731	0.5269
159	22831	0.4524	0.5476
160	25018	0.4331	0.5669
161	27303	0.4150	0.5850
162	29684	0.3975	0.6025
163	32163	0.3853	0.6147
164	34738	0.3746	0.6254
165	37411	0.3622	0.6378
166	40180	0.3535	0.6465
167	43047	0.3430	0.6570
168	46010	0.3306	0.6694
169	49071	0.3266	0.6734
170	52228	0.3219	0.6781
171	55484	0.3171	0.6829
172	58835	0.3165	0.6835
173	62284	0.3193	0.6807
174	65829	0.3246	0.6754
175	69472	0.3100	0.6900

Output of our kinetic program

k_{AB} and K were used as fitted parameters (see next page for choice)

k_{AB} 4.019785e-05 *
K 2.376697e+00 *
SSD (sum of squares): 4.788478e-04
Standard deviation: 3.868332e-03
t0 = -3457 s (when [A]=1 and [B]=0)

Use of constant chi-square boundaries as confidence limits, nsigma=3
k_{AB} 4.019785e-05 ± 7.5e-07 (1.9%)
K 2.376697e+00 ± 5.8e-02 (2.5%)

Confidence limits by Monte Carlo simulation (bootstrap method)
200 synthetic data sets, nsigma = 3
k_{AB} 4.013240e-05 ± 7.0e-07 (1.8%)
K 2.384368e+00 ± 5.8e-02 (2.4%)

Kinetic curves

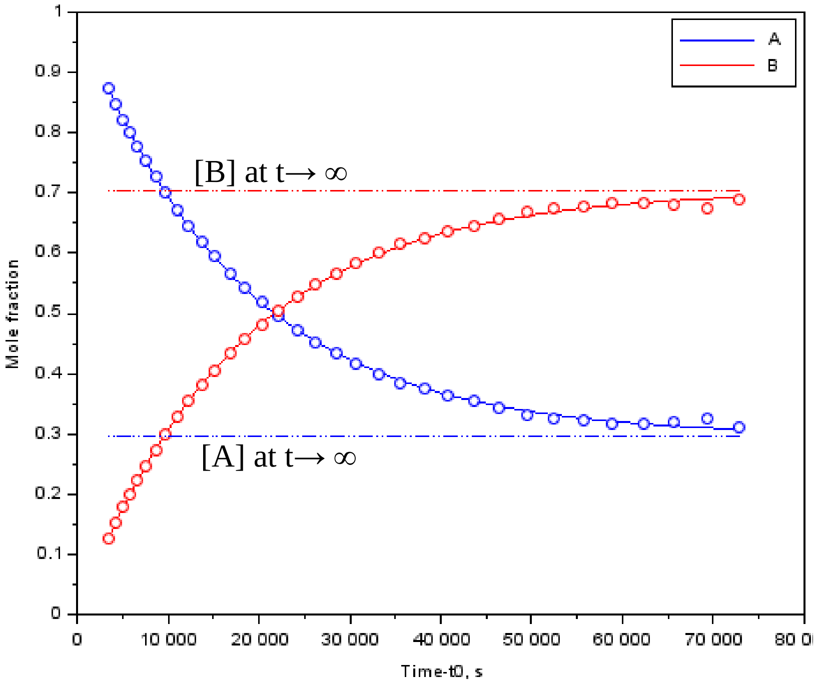

Choice of fitted parameters for A⇄B kinetics

Reversible reaction A⇄B can be characterized by three parameters: rate constants of direct and reverse reactions k_{AB} and k_{BA} and equilibrium constant K. Since $K=k_{AB}/k_{BA}$, only two of them are independent. If K is not known in advance, the fitting procedure should find one of three pairs of parameters (k_{AB} and k_{BA}), (k_{AB} and K) or (k_{BA} and K), and the third parameter is calculated from the two found. Naturally, the choice of a particular pair of fitting parameters does not affect the values of the parameters, but the errors in determining the parameters depend on it. We found that pair (k_{AB} and K) as fitting parameters for A⇄B gives minimal errors. Exactly the same conclusion was made for another reversible reaction [A.M. Genaev, H.S. Rzepa, A.V. Shernyukov, G.E. Salnikov, V.G. Shubin, *Org. Biomol. Chem.* **2019**, DOI: 10.1039/C9OB00607A].

Fitted and calculated parameters

Correlation of the fitted parameters by bootstrap method (200 synthetic data sets)

Fitted:
$k_{AB} = (6.638\pm0.078)\times10^{-5}\ s^{-1}$
$k_{BA} = (4.179\pm0.073)\times10^{-5}\ s^{-1}$

Calculated: [a]
$K = k_{AB}/k_{BA} = 1.589\pm0.033$

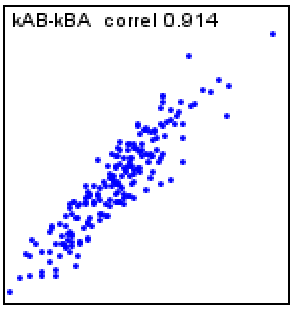

Fitted:
$k_{AB} = (6.638\pm0.078)\times10^{-5}\ s^{-1}$
$K = 1.589\pm0.014$

Calculated: [a]
$k_{BA} = k_{AB}/K = (4.179\pm0.061)\times10^{-5}\ s^{-1}$

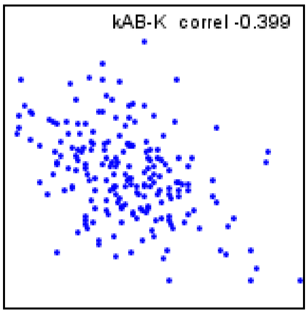

Fitted:
$k_{BA} = (4.179\pm0.073)\times10^{-5}\ s^{-1}$
$K = 1.589\pm0.014$

Calculated: [a]
$k_{AB} = k_{BA}*K = (6.638\pm0.130)\times10^{-5}\ s^{-1}$

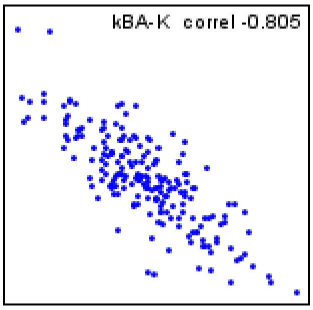

[a] by formula $(\sigma_f/f)^2 = (\sigma_x/x)^2 + (\sigma_y/y)^2$

Rate constant of direct reaction k_{AB} and equilibrium constant K are optimal fitting parameters for reversible reaction A⇄B.

Thermodynamic and activation parameters of **A→B** (**2**$^{RS/SR}$**→2**$^{RR/SS}$) reaction in DMSO-d_6

Enthalpy and entropy of reaction

$\Delta G_0 = \Delta H_0 - T\,\Delta S_0 = -RT\ lnK$

Temperature, °C	K [a]
156.0	1.31
146.3	1.29
136.7	1.29
127.0	1.27
117.4	1.32
175.3	1.32
175.3	1.29

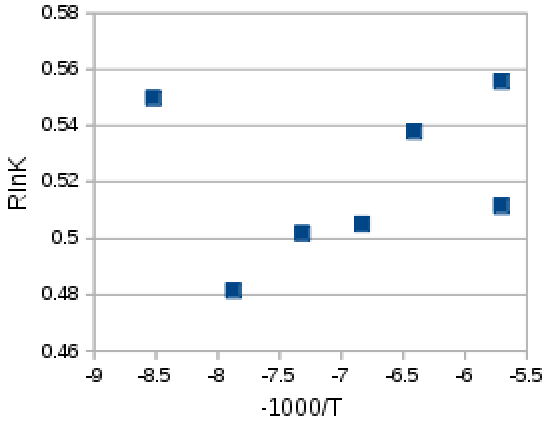

ΔH_0 = 0.005±0.011 kcal/mol
ΔS_0 = 0.558±0.079 cal/mol/K
(1 sigma)
Since the equilibrium constant is practically independent of temperature, the enthalpy of the reaction is close to zero.

Enthalpy and entropy of activation

Eyring equation
$\Delta G^{\#} = \Delta H^{\#} - T\,\Delta S^{\#} = -RT\ (ln(k_{AB}/T) - ln(æ/h))$
(transmission coeff. = 1, R = 1.987, ln(æ/h)=23.76)

Temperature, °C	k_{AB}, s^{-1}	$\Delta G^{\#}$, kcal/mol
175.7	2.5664	25.80
165.6	0.7966	26.22
156.2	0.5336	25.98
146.3	0.2043	26.17
136.6	0.0839	26.26
126.9	0.0393	26.23
117.3	0.0184	26.17
69.1 [a]	6.64E-05	26.67
155.3 [a]	0.6026	25.83
145.8 [a]	0.2430	25.99
136.4 [a]	0.0952	26.15
127.1 [a]	0.0458	26.12
117.9 [a]	0.0133	26.46

[a] Concentration of (A+B) is 32 times less

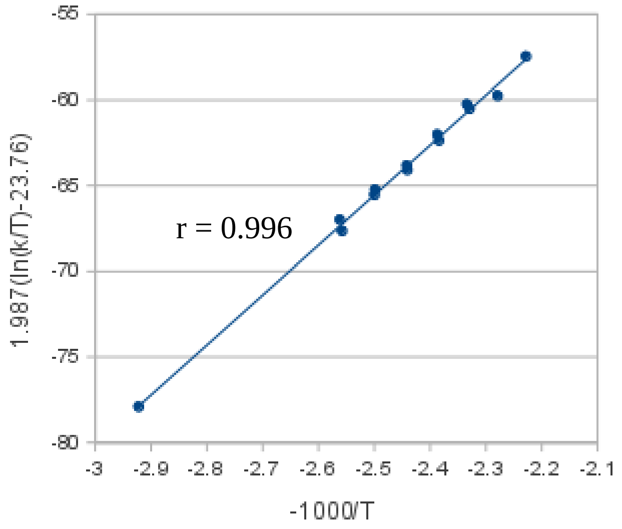

$\Delta H^{\#}$ = 29.18±0.54 kcal/mol
$\Delta S^{\#}$ = 7.4±1.3 cal/mol/K [b]
(1 sigma)

[b] Positive values suggest that entropy increases upon achieving the transition state, which often indicates a dissociative mechanism in which the activated complex is loosely bound and about to dissociate [https://en.wikipedia.org/wiki/Entropy_of_activation].

Kinetics A⇄B ($2^{RS/SR}$⇄$2^{RR/SS}$) at 74 °C in toluene-d_8

5.0 mg $2^{RS/SR}$ (473-2), 0.58 ml tol-d_8

^{1}H NMR spectrum (CH region, expno 353).

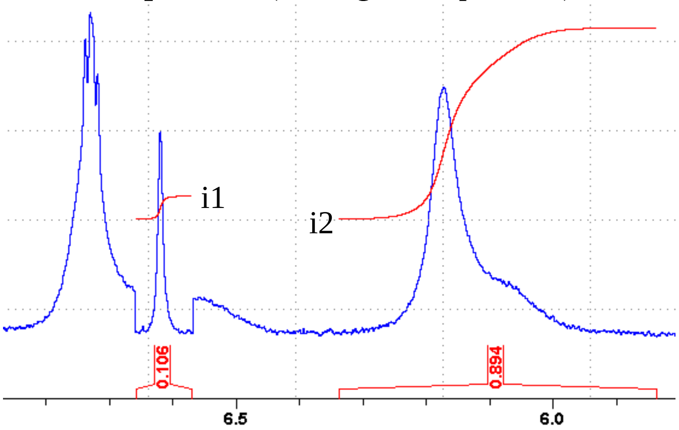

A⇄B $K = k_{AB}/k_{BA}$

[A] = i2/(i1+i2), [B] = 1-[A]

kinetics_data.txt

expno	time,s	i1	i2
351	0	0.0484	0.9516
352	202	0.0747	0.9253
353	586	0.1058	0.8942
354	865	0.1298	0.8702
355	1570	0.1939	0.8061
356	2402	0.2468	0.7532
357	3359	0.3051	0.6949
358	4442	0.3506	0.6494
359	5652	0.4112	0.5888
360	6987	0.4427	0.5573
361	8448	0.4928	0.5072
362	10036	0.5304	0.4696
363	11749	0.5411	0.4589
364	13588	0.5605	0.4395
365	15554	0.5892	0.4108
366	17645	0.5983	0.4017
367	19862	0.6109	0.3891
368	22206	0.6261	0.3739
369	24675	0.624	0.376
370	27270	0.6315	0.3685
371	29992	0.6324	0.3676
372	32839	0.6285	0.3715
373	35812	0.6394	0.3606
374	38912	0.6311	0.3689
375	42137	0.645	0.355
376	45488	0.6337	0.3663
377	48966	0.6345	0.3655
378	52569	0.6305	0.3695
379	56298	0.6346	0.3654
380	60153	0.6365	0.3635
381	64135	0.6388	0.3612
382	68243	0.6461	0.3539
383	72476	0.6427	0.3573
384	76836	0.633	0.367
385	81321	0.632	0.368
386	83053	0.636	0.364

Output of our kinetic program

k_{AB} and K were used as fitted parameters

kAB 1.052407e-04 *
K 1.744782e+00 *
SSD (sum of squares): 1.170408e-03
Standard deviation: 5.867177e-03
t0 = -478 s (when [A]=1 and [B]=0)

Use of constant chi-square boundaries as confidence limits, nsigma=3
kAB 1.052407e-04 +/- 3.3e-06 (3.1%)
K 1.744782e+00 +/- 3.0e-02 (1.7%)

Confidence limits by Monte Carlo simulation (montecarlo method)
200 synthetic data sets, nsigma = 3

kAB 1.053878e-04 +/- 3.4e-06 (3.2%)
K 1.743911e+00 +/- 3.1e-02 (1.8%)

Kinetic curves

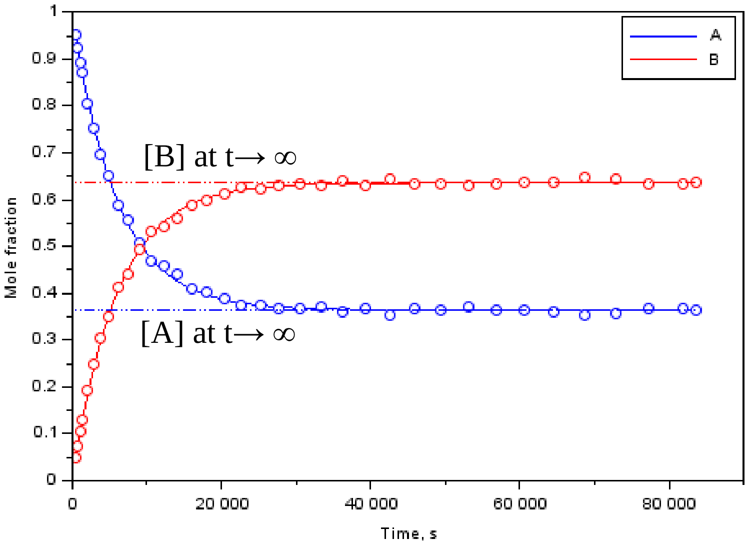

Kinetics 3 + TEMPO at 74 °C in toluene-d_8

6.7 mg **3**, 9.0 mg TEMPO (3 eq.), 0.50 ml tol-d_8

^{1}H NMR spectrum (part of aromatics, expno 205).

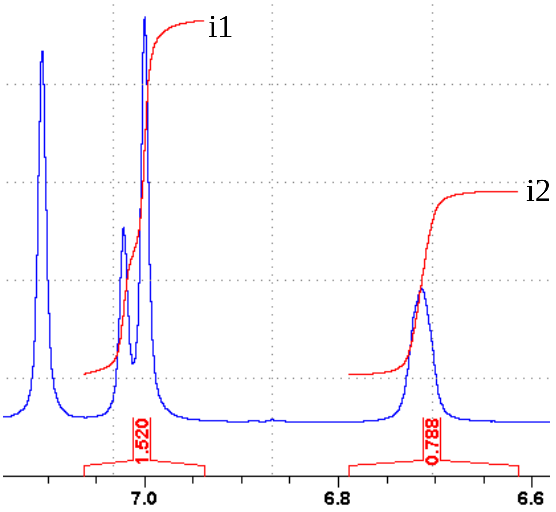

Integral i1 (residual o- and p-protons of tol-d_8) is taken as 1.52.

[**3**] = i2

[**3**] = [**3**]$_0$ exp(-kt)

Output of our kinetic program

k 3.282841e-06 *

SSD (sum of squares): 2.805781e-04
Standard deviation: 3.058203e-03
t0 = -2477 s

Use of constant chi-square boundaries as confidence limits, nsigma=3

k 3.282841e-06 +/- 5.6e-08 (1.7%)

Confidence limits by Monte Carlo simulation (montecarlo method)
200 synthetic data sets, nsigma = 3

k 3.287166e-06 +/- 5.8e-08 (1.8%)

kinetics_data.txt

expno	time,s	[**3**]
176	0	0.9919
177	115	0.9889
178	330	0.9906
179	436	0.9918
180	984	0.9914
181	1102	0.99
182	1416	0.9903
183	2457	0.9853
184	3691	0.9818
185	5119	0.9778
186	6742	0.9704
187	8558	0.9621
188	10568	0.9561
189	12773	0.947
190	15171	0.9447
191	17763	0.9329
192	20550	0.9224
193	23530	0.9148
194	26705	0.9041
195	30073	0.8952
196	33635	0.8855
197	37392	0.8739
198	41342	0.8633
199	45486	0.851
200	49825	0.84
201	54358	0.8291
202	59084	0.8181
203	64005	0.8055
204	69119	0.7927
205	72566	0.7876
206	72835	0.7882

Kinetic curves

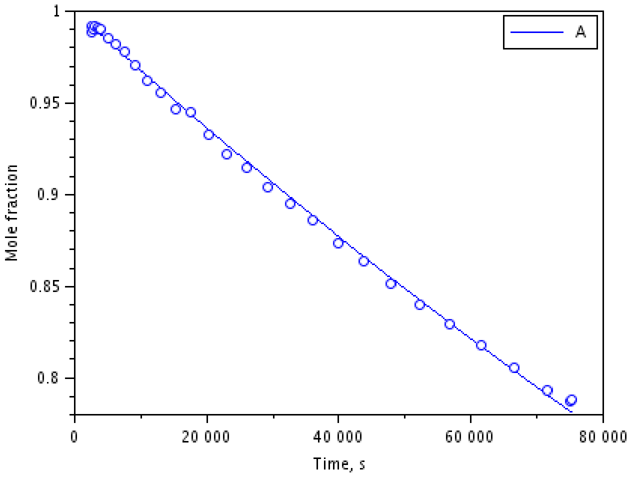

Kinetics 3 + TEMPO at 103 °C in toluene-d_8

6.7 mg **3**, 9.0 mg TEMPO (3 eq.), 0.50 ml tol-d_8

^{1}H NMR spectrum (part of aromatics, expno 221).

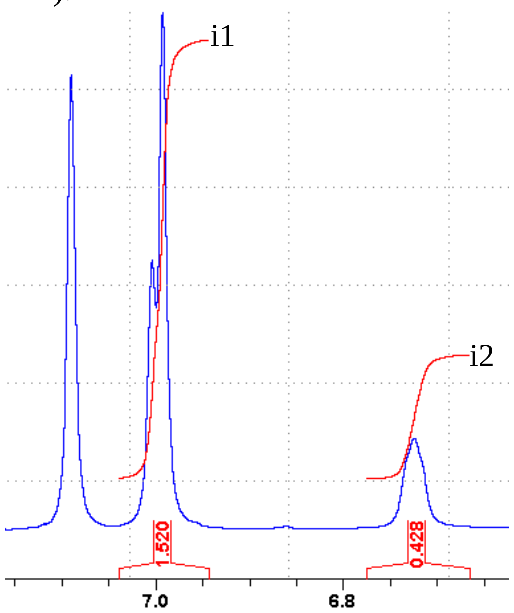

Integral i1 (residual o- and p-protons of tol-d_8) is taken as 1.52.

[**3**] = i2

[**3**] = [**3**]$_0$ exp(-kt)

kinetics_data.txt

expno	time,s	[**3**]
208	0	0.7728
209	290	0.7532
210	796	0.7159
211	1122	0.6904
212	1388	0.6771
213	2205	0.6443
214	3172	0.615
215	4287	0.5846
216	5552	0.5593
217	6965	0.5324
218	8528	0.5064
219	10239	0.4772
220	12100	0.4398
221	12752	0.4284
222	12848	0.4259

Output of our kinetic program

k 1e-04

t0 = -2577s

Kinetic curves

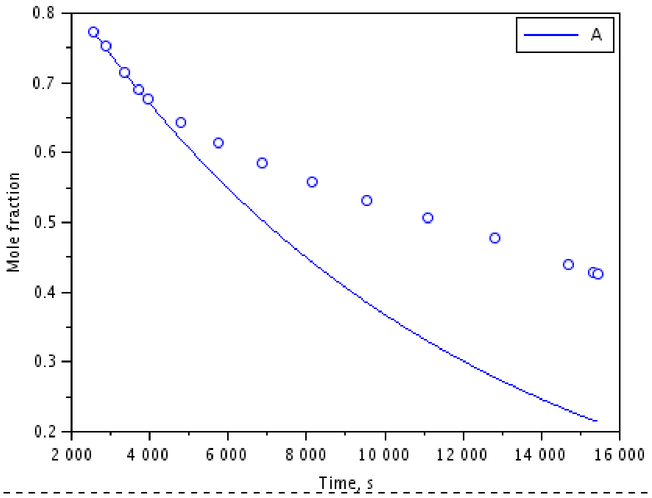

Kinetics $2^{RS/SR}$ + TEMPO at 74 °C in toluene-d_8

7.8 mg (0.019 mmol) $\mathbf{2}^{RS/SR}$ + 10.0 mg (0.064 mmol, 3.4 eq.) TEMPO + 0.56 mL toluene-d_8; initial concentration of $\mathbf{2}^{RS/SR}$ 0.033 mol/L; lmr-cherkas-473-2_06.2020

^{1}H NMR spectrum (expno 471).

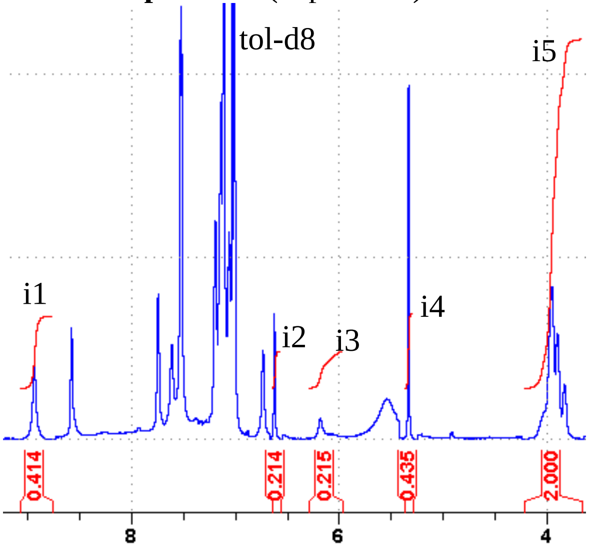

i5 is integral of all OCH_2
Normalization: i5 = 2

Kinetic model

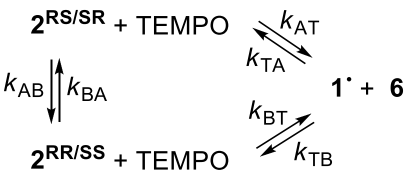

kin etics_data.txt

expno	time,s	i1 **1•**	i2 **2**$^{RR/SS}$	i3 **2**$^{RS/SR}$	i4 **6**
552	0	0.0386	0.0252	0.8568	0.0319
553	215	0.0741	0.0252	0.8285	0.0634
554	377	0.0977	0.0282	0.8084	0.0838
555	1121	0.193	0.023	0.7093	0.1722
556	1298	0.2105	0.0245	0.6959	0.1859
557	1852	0.2654	0.0263	0.6362	0.2375
558	2502	0.3181	0.0319	0.5844	0.2848
559	3249	0.3657	0.0401	0.5325	0.3305
560	4094	0.4045	0.0494	0.4851	0.3614
561	5035	0.4409	0.0577	0.4381	0.3904
562	6074	0.4619	0.0709	0.4002	0.4167
563	7209	0.4772	0.0871	0.3649	0.4319
564	8441	0.4912	0.1039	0.3395	0.4425
565	9771	0.4984	0.1236	0.3113	0.4476
566	11197	0.4958	0.1411	0.2919	0.4447
567	12721	0.5063	0.1596	0.2701	0.4498
568	14341	0.4983	0.1741	0.2548	0.4423
569	16058	0.4907	0.1872	0.2474	0.442
570	17873	0.488	0.2017	0.2288	0.438
571	19784	0.4808	0.2137	0.2154	0.4354
572	21793	0.48	0.2258	0.2109	0.4341
573	23898	0.4877	0.2308	0.2047	0.4307
574	26100	0.4813	0.2344	0.193	0.4246
575	28400	0.4733	0.241	0.1917	0.4253
576	30796	0.4744	0.2456	0.1888	0.4243
577	33290	0.4755	0.2534	0.1827	0.4235
578	35880	0.4716	0.2531	0.1829	0.4229
579	38569	0.4715	0.2571	0.1835	0.4202
580	41353	0.4686	0.2562	0.1773	0.4149
581	44234	0.469	0.2584	0.1739	0.4153
582	47213	0.4694	0.2568	0.1846	0.4208
583	50288	0.4695	0.2574	0.1799	0.4221
584	53461	0.4577	0.2607	0.1993	0.4172
586	56574	0.4522	0.2634	0.1939	0.4159

Signals of TEMPO are not inegrable,
[TEMPO] = C_{TEMPO} – [**6**] = 3.36 – i4

Output of our kinetic program

kAB 1.580127e-06 *
kBA 1.027455e-05 *
kAT 4.939388e-05 *
kTA 1.322692e-04 *
kBT 2.216252e-05 *
kTB 1.061606e-04 *
SSD (sum of squares): 4.682512e-02
Standard deviation: 1.689731e-02

Use of constant chi-square boundaries as confidence limits, nsigma=3
kAB 1.580127e-06 +/- >100%
kBA 1.027455e-05 +/- >100%
kAT 4.939388e-05 +/- 3.1e-06 (6.3%)
kTA 1.322692e-04 +/- 3.5e-05 (26.5%)
kBT 2.216252e-05 +/- 1.1e-05 (47.6%)
kTB 1.061606e-04 +/- 3.1e-05 (29.3%)

All constants are calculated in s^{-1} units. To obtain the correct kAT, kTA, kBT and kTB values (as for bimolecular reactions), they must be divided by the initial concentration of $\mathbf{2}^{RS/SR}$ (0.033 mol/L).

Kinetic curves

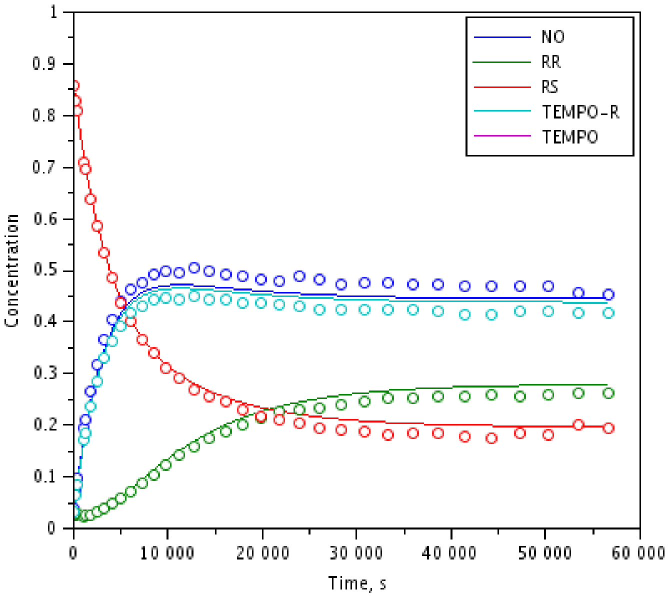

Kinetics $2^{RS/SR}$ + PhSH at 74 °C in toluene-d_8

5.7 mg $2^{RS/SR}$ (473-2), 0.115 ml PhSH, 0.50 ml tol-d_8

^{1}H NMR spectrum (region of CH protons, expno 435).

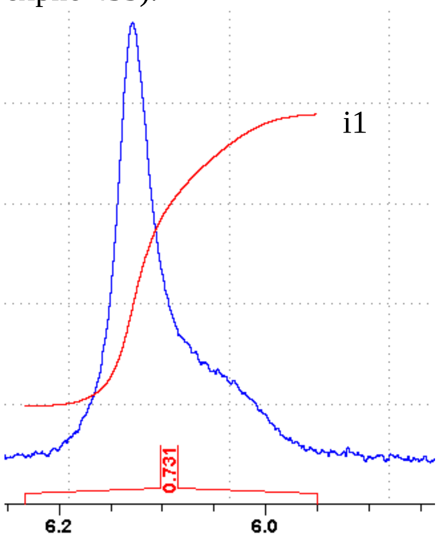

kinetics_data.txt

expno	time,s	RS
432	0	0.7992
433	101	0.7905
434	322	0.7642
435	516	0.731
436	1227	0.6259
437	1487	0.6058
438	2134	0.5881
439	2896	0.5207
440	3773	0.4364
441	4764	0.3753
442	5871	0.3075
443	7093	0.2585
444	8430	0.2044
445	9882	0.1513
446	11449	0.119
447	13131	0.0841
448	14928	0.0661
449	16839	0.0417
450	18866	0.0268
451	21008	0.0206
452	23265	0.0128
453	25637	0.0091

Integral of OCH_2 protons of reagents and products (3.78-4.16 ppm) is taken as 2.

[RS] = i1

$[RS] = [RS]_0 \exp(-kt)$

Output of our kinetic program

k 1.630616e-04 *

SSD (sum of squares): 2.986192e-03
Standard deviation: 1.192475e-02
t0 = -1375 s

Use of constant chi-square boundaries as confidence limits
nsigma=3

k 1.630616e-04 +/- 6.5e-06 (4.0%)

Confidence limits by Monte Carlo simulation (montecarlo method)
200 synthetic data sets, nsigma = 3

k 1.634367e-04 +/- 6.1e-06 (3.8%)

Kinetic curves

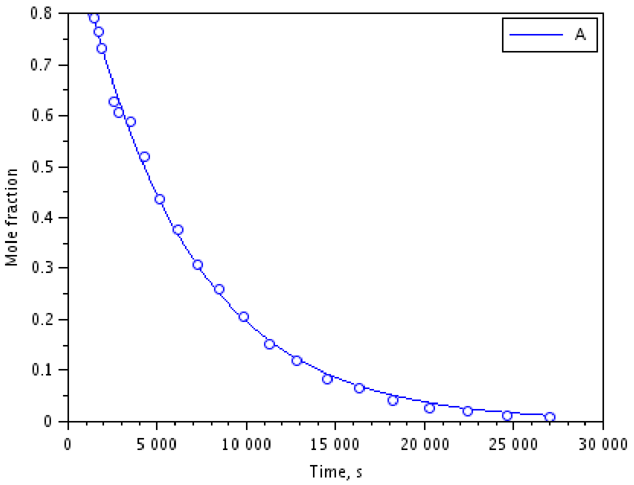

Kinetics $2^{RR/SS}$ + PhSH at 74 °C in toluene-d_8

7.1 mg $2^{RR/SS}$ (473-1), 0.115 ml PhSH, 0.50 ml tol-d_8

^{1}H NMR spectrum (region of α-pyridine protons, expno 191).

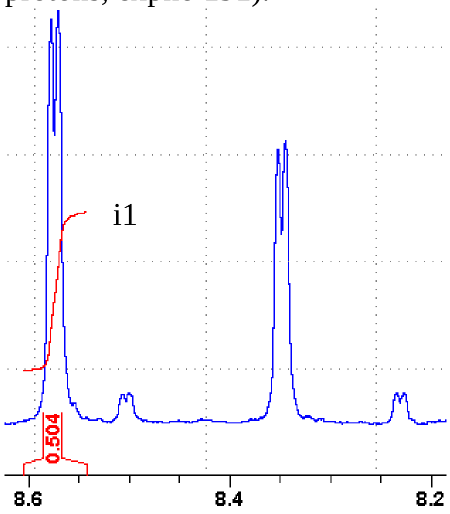

Integral of OCH_2 protons of reagents and products (3.76-4.12 ppm) is taken as 2.

[RR] = i1−0.015
(0.015 is related to an impurity under the main signal)

[RR] = $[RR]_0$ exp(-kt)

Output of our kinetic program

k 6.990292e-05 *

SSD (sum of squares): 1.214505e-03
Standard deviation: 6.706836e-03
t0 = -3719 s

Use of constant chi-square boundaries as confidence limits
nsigma=3

k 6.990292e-05 +/- 1.4e-06 (2.0%)

Confidence limits by Monte Carlo simulation (montecarlo method)
200 synthetic data sets, nsigma = 3

k 6.999264e-05 +/- 1.2e-06 (1.7%)

kinetics_data.txt

expno	time,s	[RR]
182	0	0.8288
183	154	0.7711
184	543	0.7509
185	728	0.7346
186	1433	0.7062
187	2264	0.6704
188	3221	0.6297
189	4304	0.5856
190	5513	0.5351
191	6847	0.4893
192	8308	0.4433
193	9896	0.401
194	11609	0.3526
195	13448	0.3094
196	15413	0.2697
197	17503	0.2308
198	19720	0.1921
199	22063	0.1624
200	24532	0.1324
201	27127	0.111
202	29848	0.0901
203	32695	0.0679
204	35667	0.0539
205	38766	0.0433
206	41991	0.0324
207	45342	0.0254
208	48819	0.0201
209	52422	0.0143
210	56151	0.0122

Kinetic curves

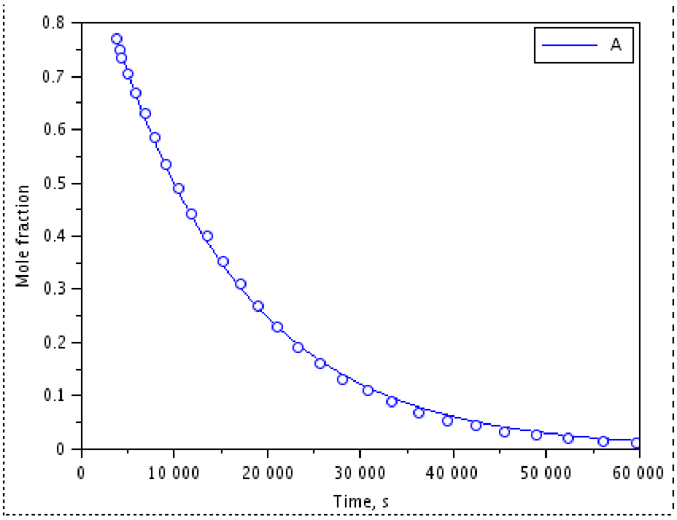

Kinetics 3 + PhSH at 74 °C in toluene-d_8

6.6 mg **3**, 0.115 ml PhSH, 0.50 ml tol-d_8

^{1}H NMR spectrum (region of α-pyridine protons, expno 191).

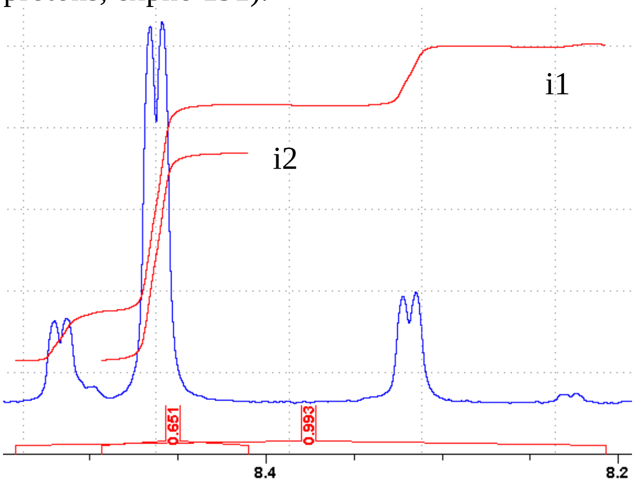

Integral (i1+i3) is taken as 1.
i3 is tiny integral of "heterocycle" at 5.89 ppm

[**3**] = i2(1+0.214)
(0.214 is related to N-invertomer at 8.52 ppm)

[**3**] = [**3**]$_0$ exp(-kt)

kinetics_data.txt

expno	time,s	[**3**]
269	0	0.9976652
270	187	1.000943
271	949	0.9953586
272	1195	1.0000932
273	1858	0.9956014
274	2637	0.9943874
275	3532	0.9905026
276	4543	0.9896528
277	5670	0.9811548
278	6913	0.9758132
279	8271	0.9786054
280	9746	0.969379
281	11337	0.9676794
282	13044	0.9618522
283	14867	0.9550538
284	16805	0.9465558
285	18860	0.9401216
286	21031	0.935994
287	23318	0.9257964
288	25721	0.9138992
289	28239	0.9021234
290	30874	0.8897406
291	33625	0.8812426
292	36492	0.8735944
293	39475	0.8606046
294	42574	0.8502856
295	45788	0.8416662
296	49119	0.8302546
297	52567	0.818843
298	56130	0.8062174
300	63604	0.7904354

Output of our kinetic program

k 3.623279e-06 *

SSD (sum of squares): 1.248389e-03
Standard deviation: 6.450810e-03
t0 = -645 s

Use of constant chi-square boundaries as confidence limits
nsigma=3

k 3.623279e-06 +/- 1.4e-07 (3.9%)

Confidence limits by Monte Carlo simulation (montecarlo method)
200 synthetic data sets, nsigma = 3

k 3.643395e-06 +/- 1.3e-07 (3.4%)

Kinetic curves

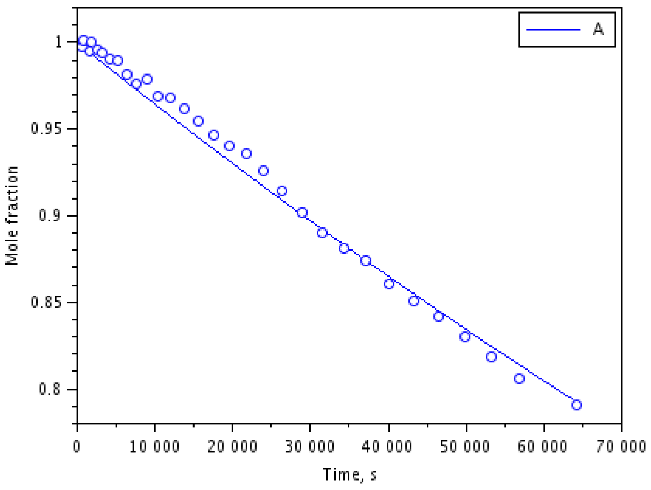

Kinetics 3 + PhSH at 103 °C in toluene-d_8

6.6 mg **3**, 0.115 ml PhSH, 0.50 ml tol-d_8

^{1}H NMR spectrum (region of α-pyridine protons, expno 191).

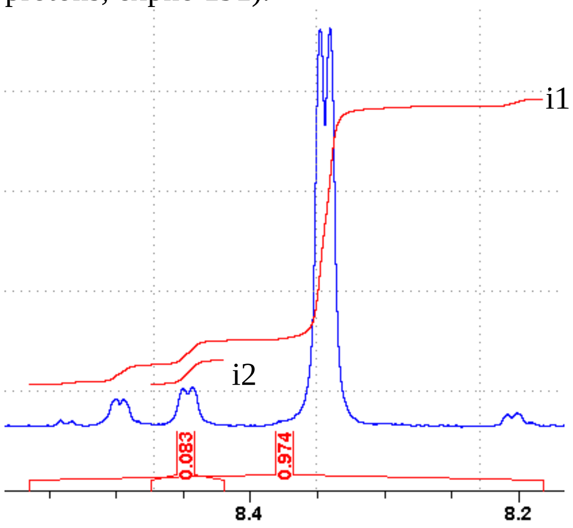

kinetics_data.txt

expno	time,s	[**3**]
303	0	0.691137
304	183	0.668505
305	598	0.606636
306	757	0.586464
307	1529	0.506391
308	2438	0.428901
309	3486	0.35301
310	4672	0.287574
311	5996	0.226689
312	7458	0.178473
313	9058	0.133824
314	10795	0.101844
315	12671	0.07257
316	14685	0.051291
317	16837	0.035178
318	19127	0.023739
319	21555	0.016974
320	24120	0.012423
321	25923	0.00984

Integral (i1+i3) is taken as 1.
i3 is tiny integral of "heterocycle" at 6.17 ppm

[**3**] = i2(1+0.23)
(0.23 is related to N-invertomer at 8.45 ppm)

[**3**] = $[\mathbf{3}]_0$ exp(-kt)

Output of our kinetic program

k 1.864765e-04 *

SSD (sum of squares): 1.034408e-03
Standard deviation: 7.580708e-03
t0 = -1981 s

Use of constant chi-square boundaries as confidence limits, nsigma=3

k 1.864765e-04 +/- 6.4e-06 (3.4%)

Confidence limits by Monte Carlo simulation (montecarlo method)
200 synthetic data sets, nsigma = 3

k 1.866560e-04 +/- 6.4e-06 (3.4%)

Kinetic curves

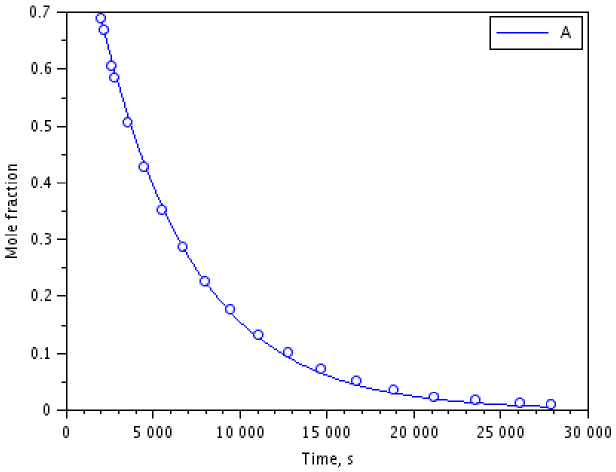

Kinetics 1• + PhSH at 27 °C in toluene-d_8

10 mg **1**•-radical, 0.115 ml PhSH, 0.50 ml tol-d_8

^{1}H NMR spectrum (region of α-pyridine protons, expno 131).

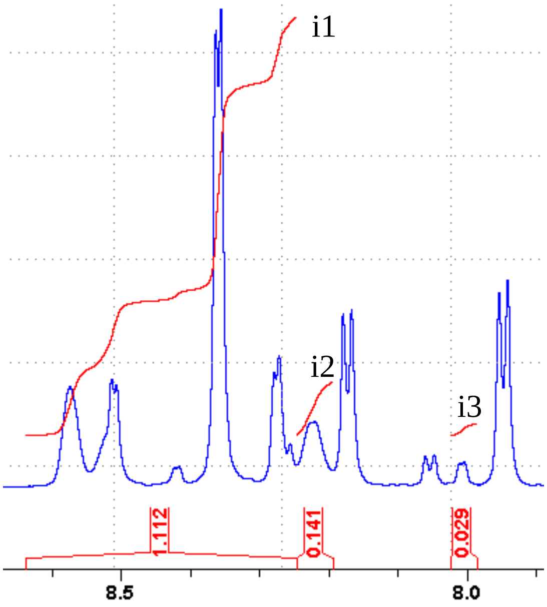

kinetics_data.txt

expno	time, s	i2
	0	1
130	258	0.4434
131	423	0.1408
132	585	0.056

Integral (i1-i2+i3) is taken as 1.

[**1**•] = i2

[**1**•] = [**1**•]$_0$ exp(-kt)

k 5.0e-03 +/- 1.8e-3

Kinetic curves

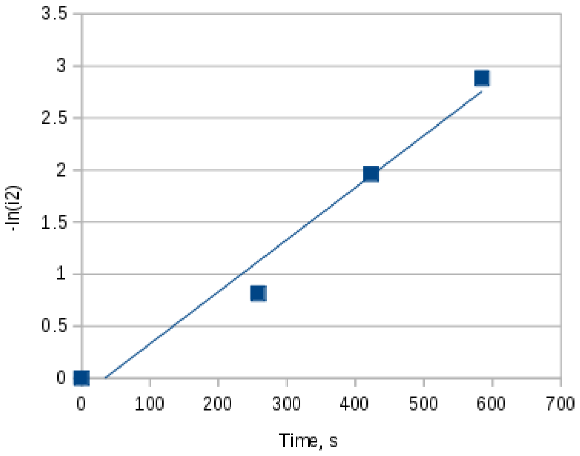

Kinetics 3 + BME at 69 °C in toluene-d_8

6.0 mg **3**, 0.080 ml BME, 0.50 ml tol-d_8

^{1}H NMR spectrum (region of α-pyridine protons, expno 191).

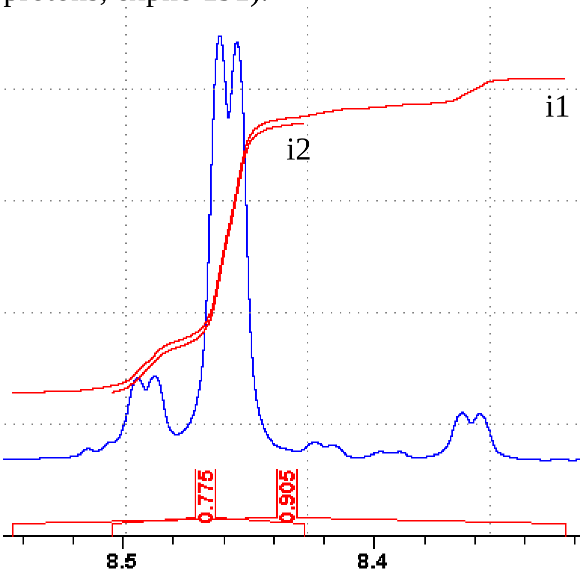

Integral (i1+i3) is taken as 1.
i3 is integral of "heterocycle" at 6.23 ppm

[**3**] = i2

[**3**] = [**3**]$_0$ exp(-kt)

kinetics_data.txt

expno	time,s	[3]
71	0	0.9647
72	369	0.9594
73	1162	0.9614
74	1890	0.9623
75	2715	0.9603
76	3637	0.951
77	4655	0.9511
78	5771	0.9485
79	6984	0.9433
80	8294	0.936
81	9701	0.9322
82	11204	0.9279
83	12805	0.9217
84	14503	0.9127
85	16298	0.9092
86	18190	0.9012
87	20179	0.893
88	22264	0.8887
89	24447	0.8805
90	26727	0.87
91	29104	0.8615
92	31578	0.855
93	34149	0.8427
94	36816	0.8347
95	39582	0.8264
96	42444	0.8162
97	45403	0.8038
98	48459	0.7938
99	51611	0.7844
100	54861	0.7752

Output of our kinetic program

k 4.060454e-06 *

SSD (sum of squares): 5.768436e-05
Standard deviation: 1.583672e-03
t0 = -12347 s

Use of constant chi-square boundaries as confidence limits, nsigma=3

k 4.060454e-06 +/- 4.5e-08 (1.1%)

Confidence limits by Monte Carlo simulation (montecarlo method)
200 synthetic data sets, nsigma = 3

k 4.066655e-06 +/- 4.2e-08 (1.0%)

Kinetic curves

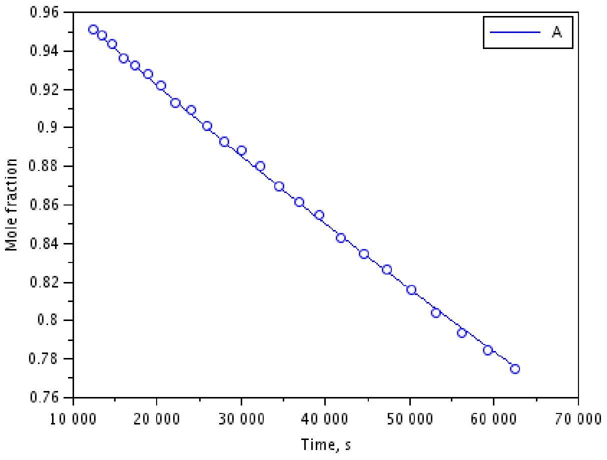

Kinetics 3 + BME at 103 °C in toluene-d_8

6.0 mg **3**, 0.080 ml BME, 0.50 ml tol-d_8

^{1}H NMR spectrum (region of α-pyridine protons, expno 109).

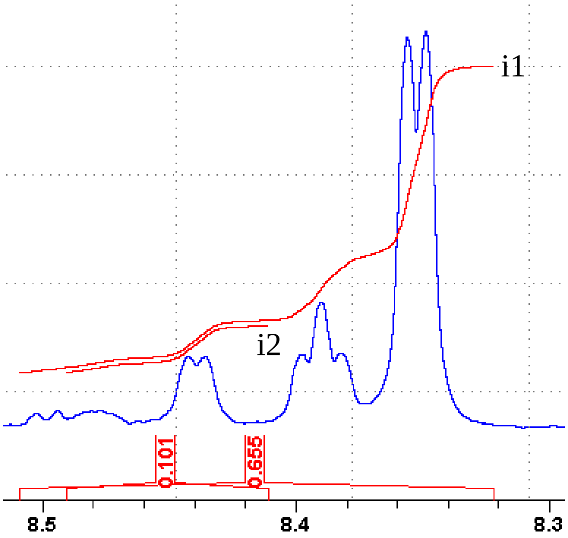

kinetics_data.txt

expno	time,s	[**3**]
101	0	0.6906
102	355	0.6161
103	960	0.5051
104	1671	0.403
105	2475	0.3109
106	3371	0.2384
107	4361	0.1826
108	5444	0.1365
109	6620	0.1009
110	7889	0.072
111	9250	0.0511
112	10705	0.0235
113	12253	0.0187
114	13894	0.0107
115	15628	0.006
116	17454	0.0034
117	19374	0.0019

Integral (i1+i3) is taken as 1.
i3 is integral of "heterocycle" at 6.22 ppm

[**3**] = i2

[**3**] = [**3**]$_0$ exp(-kt)

Output of our kinetic program

k 3.086419e-04 *

SSD (sum of squares): 7.880704e-04
Standard deviation: 7.018148e-03
t0 = -1199 s

Use of constant chi-square boundaries as confidence limits, nsigma=3

k 3.086419e-04 +/- 1.1e-05 (3.4%)

Confidence limits by Monte Carlo simulation (montecarlo method)
200 synthetic data sets, nsigma = 3

k 3.075865e-04 +/- 9.9e-06 (3.2%)

Kinetic curves

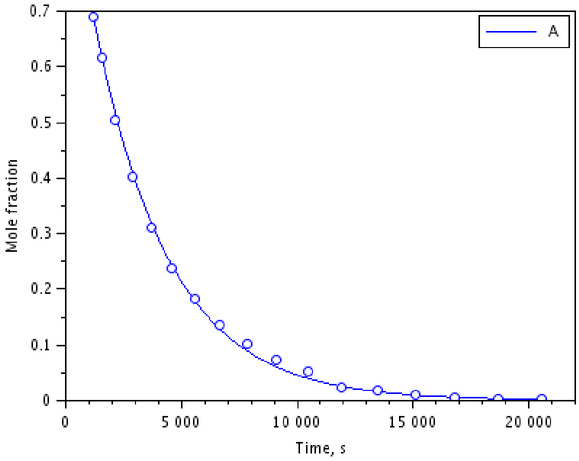

Kinetics $2^{RS/SR}$ (RS)+BME and $2^{RR/SS}$ (RR)+BME at 74 °C in toluene-d_8

5.0 mg RS (473-2) or RR (473-1), 80 mkl BME, 0.50 ml tol-d_8

RS → RR	kAB	
RR → RS	kBA	K = kAB/kBA
RS → P	kAP	
RR → P	kBP	kP = kAP/kBP

Fitting of both data sets together

kAB	8.292233e-05 +/- 5.8e-06 (7.0%)
K	1.329895e+00 +/- 1.2e-01 (8.9%)
kAP	8.270391e-05 +/- 6.1e-06 (7.3%)
kP	3.245143e+00 +/- 7.5e-01 (23.2%)

kinetics data **RS+BME**

time	RS	RR
800	0.7689	0.0435
1701	0.6401	0.083
2435	0.595	0.1154
3265	0.502	0.1363
4191	0.4333	0.1582
5215	0.3727	0.1772
6335	0.3198	0.1916
7553	0.2757	0.1984
8867	0.2477	0.21
10279	0.2118	0.2088
11787	0.1857	0.2094
13393	0.1651	0.2037
15095	0.1476	0.1985
16895	0.1318	0.1922
18791	0.1218	0.1809
20785	0.1101	0.1727
22875	0.1015	0.1605
25063	0.0929	0.1511
27347	0.0846	0.1387
29729	0.078	0.1291
32207	0.07	0.12
34783	0.0643	0.1099
37455	0.0576	0.0994
40225	0.0521	0.0877
43091	0.0453	0.0821
46056	0.0412	0.0713
49117	0.0356	0.0612

kinetics data **RR+BME**

time,s	RS	RR
400	0.0101	0.8927
570	0.0153	0.9095
1069	0.0378	0.8779
1293	0.0474	0.8506
1930	0.0670	0.8094
2680	0.0905	0.7610
3543	0.1078	0.7083
4518	0.1284	0.6499
5607	0.1451	0.6025
6809	0.1528	0.5479
8124	0.1609	0.5005
9551	0.1619	0.4507
11092	0.1657	0.4152
12746	0.1585	0.3750
14513	0.1585	0.3448
16393	0.1528	0.3137
18386	0.1422	0.2783
20491	0.1322	0.2586
22710	0.1250	0.2328
25042	0.1145	0.2232
27487	0.1063	0.1935
30044	0.0939	0.1777
32715	0.0891	0.1494
35499	0.0776	0.1398
38396	0.0714	0.1193
41406	0.0637	0.1044
44528	0.0527	0.0948
47764	0.0450	0.0872
51113	0.0369	0.0781
54576	0.0330	0.0575
58151	0.0292	0.0498
61839	0.0182	0.0412
65639	0.0148	0.0359
69553	0.0096	0.0216
73580	0.0038	0.0096
80053	0.0024	0.0072
80272	0.0000	0.0053

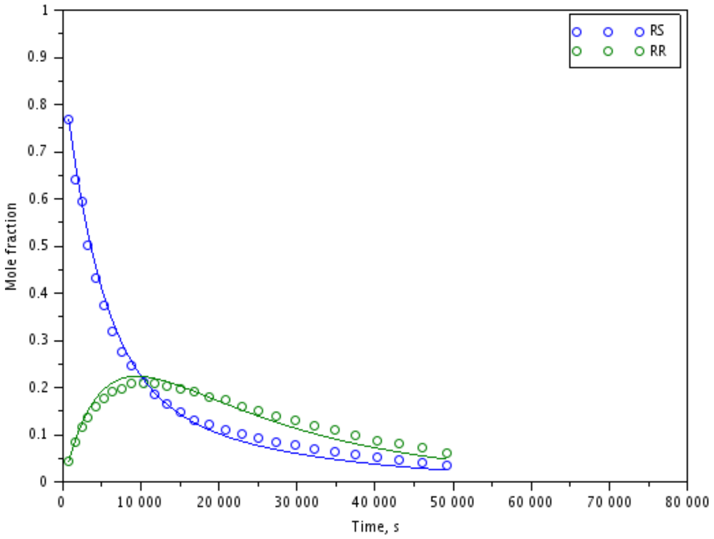

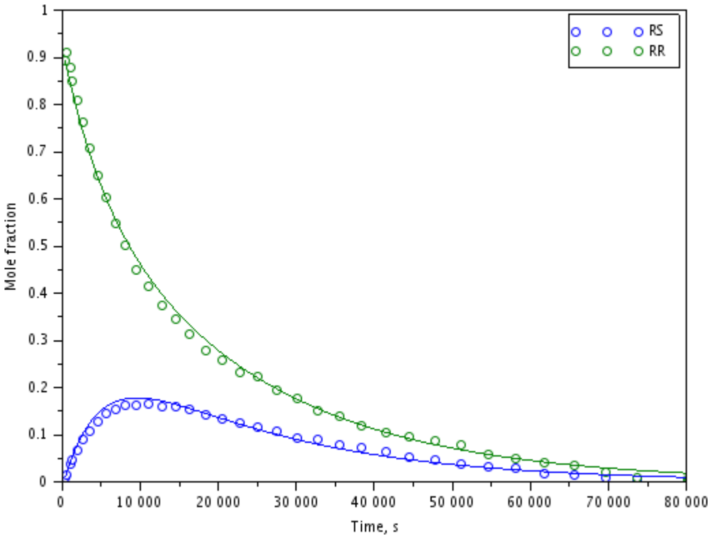

Kinetics 1• + BME at 74 °C in toluene-d_8

3.0 mg **1•**, 80 mkl BME, 0.50 ml tol-d_8

^{1}H NMR spectrum (expno 111).

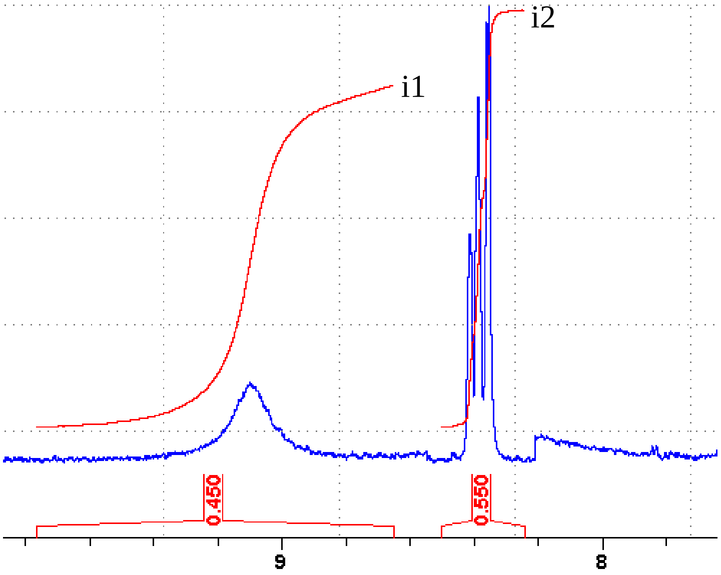

kinetics_data.txt

expno	time,s	i1	i2
111	0	0.4502	0.5498
112	387	0.1891	0.8109
113	689	0.0769	0.9231

i1 is 1H of **1•** radical
i2 is integral of α-pyridine protons of products.

[**1•**] = i1/(i1+i2),

d[**1•**]/dt = –k_{12}[**1•**]
d[**1•**]/dt = –k_{NO}[**NO˙**][BME]; [BME] = 1.943 mol/l
k_{NO} = k_{12}/[BME] = $(1.22\pm0.24)\cdot10^{-3}$ $s^{-1}mol^{-1}$

Output of our kinetic program

k12 2.373473e-03 *

SSD (sum of squares): 2.062912e-04
Standard deviation: 1.015606e-02

Use of constant chi-square boundaries as confidence limits, nsigma=3

k12 2.373473e-03 +/- 4.7e-04 (19.7%)

Confidence limits by Monte Carlo simulation
200 synthetic data sets, nsigma = 3

k12 2.373473e-03 +/- 3.3e-04 (14.1%)

Kinetic curves

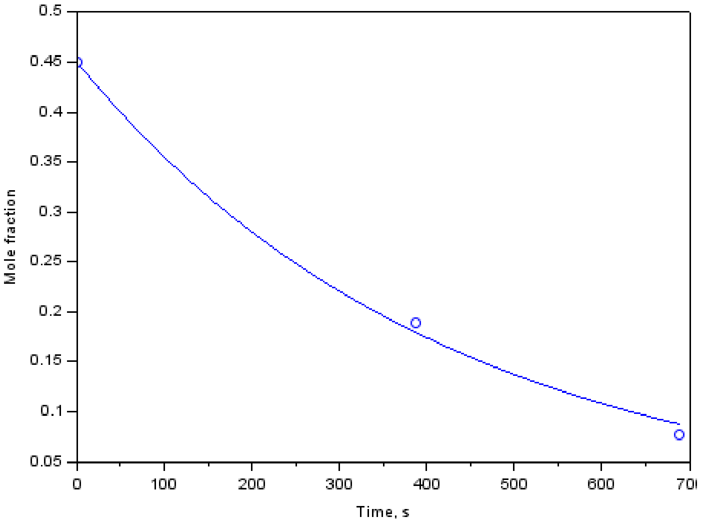

Kinetics of decomposition of alkoxyamine 2 in DMSO-d_6

$2^{RS/SR}$+$2^{RR/SS}$ mixture of diastereomers (473-mix and 427-2, the latter is at low concentration)

expno	time, s	i1 [a]	i2 [a]	Temp., °C	k, s^{-1}	ΔG$^{\#}$, kcal/mol	$t_{½}$, minutes
473-mix/41	1554723089	0.6324	0.4764	156.2	0.00016	32.9	70
473-mix/44	1554724552	0.5036	0.3686	156.2			
473-mix/45	1554724920	0.4750	0.3569	146.3	4.7E-05	33.1	244
473-mix/47	1554726110	0.4428	0.3436	146.3			
473-mix/57	1554782860	0.3000	0.2250	175.5	0.0017	32.3	6.7
473-mix/59	1554784110	0.0339	0.0261	175.5			
473-2/91	1556252119	0.0410	0.0308	155.9	0.00038	32.2	30
473-2/92	1556253147	0.0277	0.0211	155.9			
473-2/93	1556253959	0.0247	0.0189	146.4	9.6E-05	32.6	121
473-2/95	1556255044	0.0223	0.0170	146.4			

[a] i1 and i2 are C_{alk}–H integrals of $2^{RR/SS}$ and $2^{RS/SR}$ diastereomers correspondingly, DMSO-d_5 integral is taken as 1

SciLab script for A⇄B kinetics

```
clear();
funcprot(0);

// A ⇄ B
// t0 = 0
// A + B = 1
// A = A0, B = B0 at t = t0
// kAB is rate constant of A->B
// kBA is rate constant of B->A
// K is equilibrium constant, K = kAB/kBA, K = B/A at t->oo


// Experimental data (see p. 182, Error! Bookmark not defined., Error! Bookmark not defined.)

[M, text] = fscanfMat('kinetics_data.txt');
t_exp0 = M(:,2);
t_start = t_exp0(1);
t_end = t_exp0($);
t_exp = t_exp0-t_start;

A_exp = M(:,4);
B_exp = M(:,3);

for i=1:length(A_exp)
    y_exp(i) = A_exp(i)/(A_exp(i)+B_exp(i))
end

A0 = y_exp(1); // initial concentrations of the reagent A
B0 = 1 - A0;

reagent_name = ['A' 'B']

rate_start = [0.0001 1]
rate_name = ['kAB' 'K']
//rate_start = [0.0001 1]
//rate_name = ['kBA' 'K']
//rate_start = [0.0001 0.0001]
//rate_name = ['kAB' 'kBA']
rate_fix = [0 0]

// Errors handling:
// W.H. Press, B.P. Flannery, S.A. Teukolsky, W.T. Vetterling.
// Numerical Recipes in C. Cambridge University Press, Cambridge: 1988

// Constant chi-square (parameters variation): p.551
// Monte Carlo (bootstrap): p. 548
nsigma = 1; // 68.3%
//nsigma = 2; // 95.4%
nsigma = 3; // 99.7%
//hessian = 1;
variation = 1;
montecarlo = 200;

////////////////////////////////////////////////////////////////////////////////
m = length(y_exp)
N = length(t_exp) // Number of measurements
N = length(y_exp) // Number of measurements
Y_EXP = y_exp;
rate_n = length(rate_start)
rate_n_opt = 0
rate_opt_i = []
for i=1:rate_n
    if rate_fix(i)==0 then
        rate_n_opt = rate_n_opt + 1
        rate_opt_i = [rate_opt_i, i]
    end
end
M = rate_n_opt

global count;
count = 0;

function y = myModel(rate,t)
    kk = rate_start
    for i=1:length(rate_opt_i)
        kk(rate_opt_i(i)) = rate(i)
    end

    k = kk(1)
    //k = kk(1)*kk(2)
    K = kk(2)
    //K = kk(1)/kk(2)
```

```
    y = (A0+B0)/(K+1) + (K*A0-B0)/(K+1)*exp(-k*(1+1/K)*t)
endfunction

function f = myDifferences ( rate, m )
    global count;
    count = count + 1;
    // Returns the difference between the simulated differential
    // equation and the experimental data.
    y_calc=myModel(rate,t_exp)
    diffmat = y_calc - y_exp
    // Make a column vector
    f = diffmat(:)
endfunction

function [rate,SSD,diffopt]=mySolve(rate_start,rate_fix)
    rate0 = []
    rate_opt_i = []
    for i=1:rate_n
        if rate_fix(i)==0 then
           rate_opt_i = [rate_opt_i,i]
           rate0 = [rate0,rate_start(i)]
//           rate_opt_i($+1) = i
//           rate0($+1) = rate_start(i)
        end
    end
    //disp(rate_start)
    //disp(rate_opt_i)
    //disp(rate0)
    if length(rate_opt_i)==0 then
        rate1_nonfixed = []
        diffopt = myDifferences(rate0,m)
    else
        [rate1_nonfixed,diffopt]=lsqrsolve(rate0,myDifferences,m)
    end
    //disp(rate1_nonfixed)
    SSD = sum(diffopt.^2)
    rate = rate_start
    for i=1:length(rate_opt_i)
//        if rate1_nonfixed(i)<0 then
//            rate1_nonfixed(i) = 0
//        end
        rate(rate_opt_i(i)) = rate1_nonfixed(i)
    end
    //disp(rate)

endfunction

[rate1,SSD,diffopt] = mySolve(rate_start,rate_fix)

[my,ny] = size(y_exp)
if isdef("exp_err") then
//if isdef("exp_err") & exp_err ~= zeros(y_exp) then
    chi_square = sum(matrix(diffopt, my, ny).^2 ./ exp_err.^2)
else
   chi_square = N-M
end
//disp(chi_square)

STD = sqrt(SSD/(N-M))

for i=1:length(rate1)
    if rate_fix(i) == 0 then
        fff='  *'
    else
        fff=''
    end
    mprintf("%s\t%e%s\n",rate_name(i),rate1(i),fff)
end
mprintf("Irerations: %d\nSSD (sum of squares): %e\nStandard deviation: %e\n", count, SSD, STD);
k = rate1(1); K = rate1(2);
t0 = log(((A0+B0)-(A0+B0)/(K+1))/((K*A0-B0)/(K+1)))/(-k*(1+1/K));
mprintf("t0 = %.0f s\n", t0);

t_plot = t_exp(1):(t_exp($)-t_exp(1))/256:t_exp($);
rate_opt_i = 1:rate_n
t_calc_plot = t_plot-t0
t_exp_plot = t_exp-t0
y_calc_plot = myModel(rate1,t_plot);
for i=1:length(t_exp_plot)
    Aoo(i) = 1/(1+K)
    Boo(i) = K/(1+K)
end


f0=scf(0);
clf(f0)
plot(t_calc_plot',y_calc_plot','-b')
```

```
plot(t_calc_plot',(1-y_calc_plot)','-r')
plot(t_exp_plot,y_exp,'ob')
plot(t_exp_plot,1-y_exp,'or')
plot(t_exp_plot,Aoo,':b')
plot(t_exp_plot,Boo,':r')


xtitle('','Time-t0, s', 'Mole fraction')
legend(reagent_name, 1)

////////////////////////////////////////////////////////////////////////////////
//if isdef("hessian") & rate_n_opt>=1 & hessian>0 then
//    mprintf("\n%s\n", 'Use of Hessian matrix to obtain the standard deviations of the fitting parameters')
//    mprintf("nsigma=%d\n\n",nsigma)
//
//    if rate_n_opt<rate_n then
//        mprintf("Warning! There is fixed perameters. Bad results are expected.\n\n")
//    end
//
//    function ssd=return_ssd(rate)
//        y_calc=ode(C0',t0,t_exp,list(myModel,rate))
//        diffmat = (y_calc' - y_exp)/STD
//        ssd = sum(diffmat.^2)
//    endfunction
//    //ssd_ttt = return_ssd(rate1)
//
//    [J, H] = numderivative(return_ssd, rate1, [], [], "blockmat")
//    //disp(H)
//    H_inv = inv(H)
//    rate_std = sqrt(2*diag(H_inv)) * nsigma
//    for i=1:rate_n
//        if rate_fix(i) ~= 0 then
//            continue
//        end
//        mprintf("%s\t%e +/- %.1e (%.1f%%)\n",...
//          rate_name(i),rate1(i),rate_std(i),rate_std(i)/rate1(i)*100)
//    end
//
//end
////////////////////////////////////////////////////////////////////////////////

///////////////////////////////////////////////////////////////////////////////
if isdef("variation") & rate_n_opt>=1 & variation>0 then
    mprintf("\n%s\n", 'Use of constant chi-square boundaries as confidence limits')
    mprintf("nsigma=%d\n\n",nsigma)
    //target_SSD = (nsigma^2 + 1) * SSD  // doubling of SSD
    // delta_chi_square = 1 where chi_square = SSD/(SSD/(N-M))
    target_SSD = SSD + SSD/(N-M)*nsigma^2
    var_step = 0.001

    f1=scf(1);
    clf(f1);

    pos = 1
    better_solve = []
    prev_ssd = SSD
    for ind=1:rate_n
        if rate_fix(ind)~=0 then
            continue
        end
        var_mat = [SSD,rate1]
        rate_fix_var = rate_fix
        rate_fix_var(ind) = 1
        ssd = 0
        count_var = 1
        rate_var = rate1
        too_big_variation = %f

        while ssd < target_SSD
            if rate_var(ind) > rate1(ind)*2 then
                too_big_variation = %t
                break
            end
            rate_var(ind) = rate_var(ind)*(1+var_step*count_var)
            //disp(1+step*count_var)
            [rate_var,ssd]=mySolve(rate_var,rate_fix_var)
            var_mat = cat(1, var_mat,[ssd,rate_var])
            mprintf("%8.3f%8.3f\r", rate_var(ind)/rate1(ind), (ssd/SSD-1)*(N-M))
            if ssd<prev_ssd then
                better_solve = rate_var
                prev_ssd = ssd
            end
            //mprintf("+%.3f\r",ssd/SSD)
            //mprintf("%6d\r",count_var)
            count_var = count_var + 1
        end
        ssd = 0
```

```
        count_var = 1
        rate_var = rate1
        while ssd < target_SSD
            if too_big_variation then
                break
            end
            if rate_var(ind) < rate1(ind)/2 then
                break
            end
            rate_var(ind) = rate_var(ind)*(1-var_step*count_var)
            if rate_var(ind) < rate1(ind)*var_step then
                too_big_variation = %t
                break
            end
            //disp(1+step*count_var)
            [rate_var,ssd]=mySolve(rate_var,rate_fix_var)
            var_mat = cat(1, [ssd,rate_var], var_mat)
            mprintf("%8.3f%8.3f\r", rate_var(ind)/rate1(ind), (ssd/SSD-1)*(N-M))
            if ssd<prev_ssd then
                better_solve = rate_var
                prev_ssd = ssd
            end
            //mprintf("-%.3f\r",ssd/SSD)
            count_var = count_var + 1
        end
        //disp(var_mat)

        if too_big_variation then
            mprintf("%s\t%e +/- >100%%\n",rate_name(ind),rate1(ind))
        else
            rate_minus = var_mat(1,2:rate_n+1) - ...
              (var_mat(1,2:rate_n+1)-var_mat(2,2:rate_n+1)) * ...
              (var_mat(1,1)-target_SSD)/(var_mat(1,1)-var_mat(2,1))
            //disp(rate_minus)
            rate_plus = var_mat($,2:rate_n+1) - ...
              (var_mat($,2:rate_n+1)-var_mat($-1,2:rate_n+1)) * ...
              (var_mat($,1)-target_SSD)/(var_mat($,1)-var_mat($-1,1))
            //disp(rate_plus)
            rate_err = ((rate_plus-rate1)+(rate1-rate_minus))/2
            mprintf("%s\t%e +/- %.1e (%.1f%%)\n",...
              rate_name(ind),rate1(ind),rate_err(ind),rate_err(ind)/rate1(ind)*100)
            //disp(var_mat)
        end

        rrr_var_mat = var_mat(:,2:rate_n+1)
        for i=1:rate_n
            if rate_fix(i)==1 then
               rrr_var_mat(:,i) = ones(rrr_var_mat(:,i))
            elseif rate1(i)==0 then
               continue
            else
               rrr_var_mat(:,i) = rrr_var_mat(:,i)/rate1(i)
            end
        end
        //disp(rrr_var_mat)
        rrr_min = min(rrr_var_mat)
        rrr_max = max(rrr_var_mat)

        subplot(1,rate_n_opt,pos)
        plot(rrr_var_mat(:,ind),rrr_var_mat)
        dc=gca();
        dc.axes_visible =["off", "off"]
        dc.data_bounds=[rrr_min rrr_max rrr_min rrr_max]
        xtitle(rate_name(ind))
        pos = pos + 1
    end
//    subplot(1,rate_n_opt+1,pos)
    legend(rate_name)
    if ~isempty(better_solve) then
        mprintf("%s\n", "Warning! There is a better solution:")
        for i=1:length(better_solve)
            mprintf("%e ", better_solve(i))
        end
        mprintf("%s\n\n","Please, try it as a starting approximation...")
    end

end
/////////////////////////////////////////////////////////////////////////////

//////////////////////////////////////////////////////////////////////////////
if isdef("montecarlo") & rate_n_opt>=1 & montecarlo>0 then
    //mark_size=int(1+6/log(montecarlo))
    if montecarlo>300 then
        mark_size=2
    elseif montecarlo>100 then
        mark_size=3
    elseif montecarlo>30 then
```

```
        mark_size=4
    else
        mark_size=6
    end
    mprintf("\n%s\n", 'Confidence limits by Monte Carlo simulation (bootstrap method)');
    mprintf("%d synthetic data sets, nsigma = %d\n\n", montecarlo, nsigma);
    [my,ny] = size(y_exp)
    resid = matrix(diffopt, my, ny)

    mean_res = zeros(1,ny)
    //mean_res = mean(resid,'r')

    std_res = stdev(resid) //Use total chi-square for all experimental curves
    //std_res = stdev(resid,'r') //Use own chi-square for each experimental curve

    if length(std_res)==1 then  // if total chi-square
       mean_res = zeros(1,ny)
       std_res = ones(1,ny) * std_res
    end

    std_res = std_res * nsigma
    rate_bs = rate1

//    disp(rate_name)
//    disp(rate1')
    for i=1:montecarlo
        resid1 = []
        for j=1:ny
          resid1 = cat(2,resid1,grand(my, 1, "nor", mean_res(j), std_res(j)))
        end
        y_exp = Y_EXP + resid1
        [rrr,ssd,diffopt]=mySolve(rate1,rate_fix)
        //[rrr,diffopt]=lsqrsolve(rate1,myDifferences,m)
        //rate_bs($+1,:) = rrr'
        rate_bs = cat(1,rate_bs,rrr)
//        disp(rate_new')
        mprintf("\r%4d %8.3f", i, sqrt(ssd/SSD-1))
    end
    mprintf("\r%s",'')
    rate_std = stdev(rate_bs, 'r');
    //rate_av = rate1  //Use rates optimized from experimental data
    rate_av = mean(rate_bs, 'r')  //Use averaged rates from synthetic data sets
    for i=1:rate_n
        if rate_fix(i) ~= 0 then
            continue
        end
        mprintf("%s\t%e +/- %.1e (%.1f%%)\n",...
          rate_name(i),rate_av(i),rate_std(i),rate_std(i)/rate_av(i)*100)
    end

    // rrr_ - data of optimized parameters only (for plot)
    rrr_name = []
    rrr_bs = []
    rrr_n = rate_n_opt
    for i=1:rate_n
        if rate_fix(i)==0 then
            rrr_name = [rrr_name,rate_name(i)]
            rrr_bs = [rrr_bs,rate_bs(:,i)]
        end
    end

    if rrr_n > 1 then
        f2=scf(2);
        clf(f2);
        for i=2:rrr_n
            for j=1:i-1
                pos =((i-2)*(rrr_n-1)+j)
                //disp(pos)
                subplot(rrr_n-1,rrr_n-1,pos)
                R = correl(rrr_bs(:,j),rrr_bs(:,i))
                plot(rrr_bs(:,j),rrr_bs(:,i),'.')
                dc=gca();
                dc.axes_visible =["off", "off"]
                a=get("current_axes");
                p1=a.children.children(1);
                //set(p1,'mark_mode',"on");
                set(p1,'mark_size',mark_size);
                xtitle(rrr_name(j)+'-'+rrr_name(i)+'  correl '+msprintf("%.3f",R))
            end
        end
    end
end
////////////////////////////////////////////////////////////////////////////////
```

Quantum chemical calculations

The most stable conformers $2^{RS/SR}$, $2^{RS/SR}$i, $2^{RR/SS}$, $2^{RR/SS}$i

All conformers are available from site http://limor1.nioch.nsc.ru/quant/NO-inversion/

DFT/PBE/Λ1 geometry in XMol xyz format; 5th column is chemical shifts (ppm) calculated by DFT/PBE/Λ22
DFT energy and ZPE are in a.u., dipole moment is in Debyes, free energy (G) and Grimme D3 dispersion (Edisp) corrections are in kcal/mol

$2^{RS/SR}$ (RS.03 from the site)

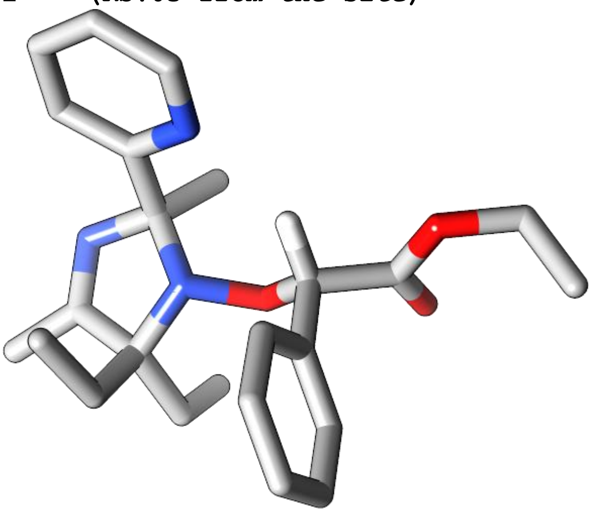

```
 61
Energy -1321.803719316  Dipole 2.34  ZPE 0.499257  G(298.15) 272.87  Edisp -54.06
C   2.01029000  -2.90379000  -2.99548000  128.2903
C   0.85125000  -2.17321000  -3.26726000  128.0069
C   2.38888000  -3.13810000  -1.66919000  128.5215
C   0.07493000  -1.67571000  -2.21717000  132.2861
C   1.61231000  -2.64148000  -0.62197000  128.9265
C   0.44936000  -1.90228000  -0.88774000  143.8755
C  -0.37355000  -1.32923000   0.25203000   84.7193
C  -0.78524000  -2.44739000   1.21285000  171.1947
O   0.36042000  -0.38364000   1.05630000  148.1969
O  -1.89027000  -3.06677000   0.72041000  177.0433
O  -0.21114000  -2.75061000   2.23294000  352.8400
N   0.44523000   0.86351000   0.33031000  187.7719
C  -2.34558000  -4.23261000   1.46595000   59.4671
C   1.85051000   1.37922000   0.28664000   85.4814
C  -0.41337000   1.93623000   0.95368000  104.5374
C  -1.65411000  -5.49736000   0.98050000    8.4728
C   1.63685000   2.81466000   0.74308000  178.1145
C   2.86456000   0.63725000   1.19637000   29.5349
C   2.37301000   1.31169000  -1.17670000   30.9492
N   0.44341000   3.11597000   1.09322000  346.7689
C  -1.58136000   2.26262000  -0.00015000  171.8189
C  -1.00674000   1.56740000   2.32216000   23.1632
C   2.74910000   3.81714000   0.78362000   13.1782
C   2.61595000   0.68844000   2.70713000    6.5408
C   1.49442000   2.03279000  -2.20033000    3.2524
N  -2.30927000   1.21666000  -0.42275000  321.2397
C  -1.88295000   3.58169000  -0.35823000  121.8898
C  -3.35088000   1.45892000  -1.22821000  149.6688
C  -2.96755000   3.82024000  -1.20228000  134.7777
C  -3.72211000   2.73647000  -1.65192000  121.5552
H   2.61910000  -3.29258000  -3.81621000    7.3947
H   0.55238000  -1.98498000  -4.30202000    7.3363
H   3.29251000  -3.71421000  -1.45193000    7.5150
H  -0.82768000  -1.09383000  -2.42448000    7.4908
H   1.89997000  -2.82496000   0.41780000    7.9417
H  -1.28003000  -0.83792000  -0.14202000    7.4743
```

H	-3.42928000	-4.26886000	1.28232000	3.5870
H	-2.15534000	-4.06279000	2.53680000	5.2489
H	-1.81596000	-5.64524000	-0.09792000	0.6944
H	-0.57283000	-5.44835000	1.17738000	1.1557
H	-2.06361000	-6.36866000	1.51767000	0.7616
H	2.89576000	-0.41140000	0.86098000	2.0632
H	3.85439000	1.06973000	0.96700000	1.3246
H	1.72531000	0.10183000	2.97120000	1.8995
H	2.47820000	1.72066000	3.07016000	0.6302
H	3.47782000	0.25932000	3.24358000	0.9929
H	2.44708000	0.24264000	-1.44069000	-0.1714
H	3.40075000	1.71648000	-1.19977000	1.0284
H	1.88877000	1.88664000	-3.21844000	-0.0813
H	0.46995000	1.63285000	-2.16290000	0.1740
H	1.44335000	3.11815000	-2.01029000	-0.3649
H	-1.69956000	0.72166000	2.22216000	1.9492
H	-1.55683000	2.44123000	2.70135000	1.5156
H	-0.21156000	1.30616000	3.03278000	1.6696
H	3.17989000	3.96310000	-0.22116000	1.6543
H	2.36728000	4.77677000	1.15811000	2.1226
H	3.56698000	3.47078000	1.43734000	1.7778
H	-1.25912000	4.38821000	0.03041000	7.9516
H	-3.92355000	0.58187000	-1.55447000	8.9709
H	-3.22022000	4.84034000	-1.50434000	7.5895
H	-4.58062000	2.87213000	-2.31393000	7.1929

2$^{RS/SR}$i (RSi.01 from the site)

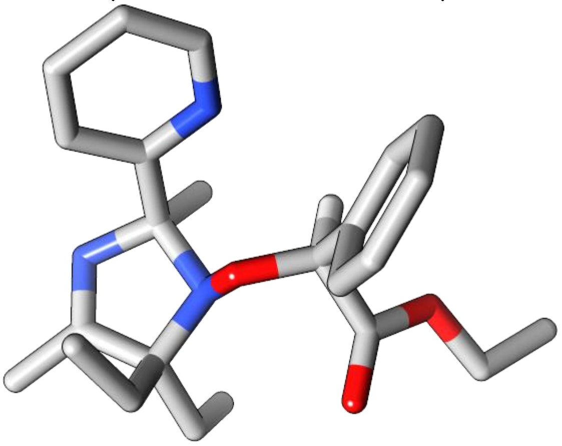

61

Energy -1321.80195945 Dipole 0.67 ZPE 0.499092 G(298.15) 273.07 Edisp -53.71

C	2.52295000	-4.21776000	1.44708000	128.4340
C	1.71634000	-3.45481000	2.29671000	128.0397
C	2.46159000	-4.01587000	0.06618000	127.5967
C	0.84902000	-2.49793000	1.76888000	128.8689
C	1.60130000	-3.05068000	-0.46184000	130.5121
C	0.78853000	-2.28655000	0.38381000	138.5949
C	-0.16014000	-1.25308000	-0.19266000	88.3913
C	-1.60766000	-1.72015000	0.03708000	177.1038
O	0.09877000	-0.01601000	0.46961000	141.7917
O	-2.04838000	-2.39577000	-1.05209000	182.7075
O	-2.24797000	-1.56109000	1.05258000	349.2959
N	-0.72785000	1.01281000	-0.14875000	209.1297
C	-3.37441000	-2.98281000	-0.92341000	62.5129
C	-1.37378000	1.87288000	0.90167000	87.1437
C	0.09727000	1.94732000	-1.04023000	104.6750
C	-3.66638000	-3.74243000	-2.20264000	8.1095
C	-0.97270000	3.26167000	0.42793000	180.2665
C	-0.89228000	1.60812000	2.35360000	29.4164
C	-2.91552000	1.66962000	0.87585000	29.6065
N	-0.17407000	3.30467000	-0.57082000	346.3789
C	1.60400000	1.65075000	-1.01411000	166.2290
C	-0.40729000	1.83033000	-2.48883000	27.0736
C	-1.47266000	4.50813000	1.09258000	13.3662
C	0.56337000	1.95758000	2.67658000	6.7038
C	-3.57913000	1.84579000	-0.49040000	4.9263

N	2.00563000	0.53750000	-1.65007000	326.8868
C	2.50124000	2.53165000	-0.40075000	123.4681
C	3.31442000	0.26083000	-1.66079000	149.2937
C	3.86395000	2.23695000	-0.43008000	133.8048
C	4.28521000	1.07118000	-1.06888000	121.4230
H	3.19949000	-4.96977000	1.86193000	7.3029
H	1.75992000	-3.61067000	3.37790000	7.2646
H	3.09107000	-4.60824000	-0.60323000	7.3984
H	0.20475000	-1.90879000	2.42537000	7.6920
H	1.55944000	-2.88131000	-1.54157000	7.6247
H	0.02441000	-1.16128000	-1.27526000	6.1599
H	-4.10275000	-2.17398000	-0.74842000	4.1983
H	-3.38643000	-3.64019000	-0.03855000	3.9463
H	-3.64665000	-3.07031000	-3.07409000	1.3979
H	-2.93071000	-4.54575000	-2.36070000	1.2099
H	-4.66765000	-4.19717000	-2.13837000	1.0741
H	-1.08231000	0.54247000	2.55566000	2.9938
H	-1.56619000	2.18158000	3.01403000	1.6149
H	1.25697000	1.35221000	2.07706000	0.6173
H	0.78411000	3.02194000	2.49117000	0.4589
H	0.76702000	1.75925000	3.74173000	0.7095
H	-3.10762000	0.65193000	1.25253000	2.4244
H	-3.35953000	2.37137000	1.60415000	1.6124
H	-3.17495000	1.11383000	-1.20593000	1.4099
H	-3.41457000	2.85468000	-0.90599000	0.4262
H	-4.66698000	1.68917000	-0.41196000	0.8130
H	0.07982000	2.59694000	-3.11074000	1.6166
H	-0.15375000	0.83717000	-2.88323000	2.9089
H	-1.49476000	1.98250000	-2.51740000	1.2924
H	-1.00283000	5.38640000	0.62906000	2.2879
H	-2.56795000	4.58998000	0.98944000	1.9137
H	-1.25186000	4.49811000	2.17285000	1.9378
H	2.12054000	3.43917000	0.06947000	7.5975
H	3.60971000	-0.66120000	-2.17638000	8.6699
H	4.58644000	2.91147000	0.03735000	7.4217
H	5.34113000	0.79484000	-1.11703000	6.8950

$2^{RR/SS}$ (RR.04 from the site)

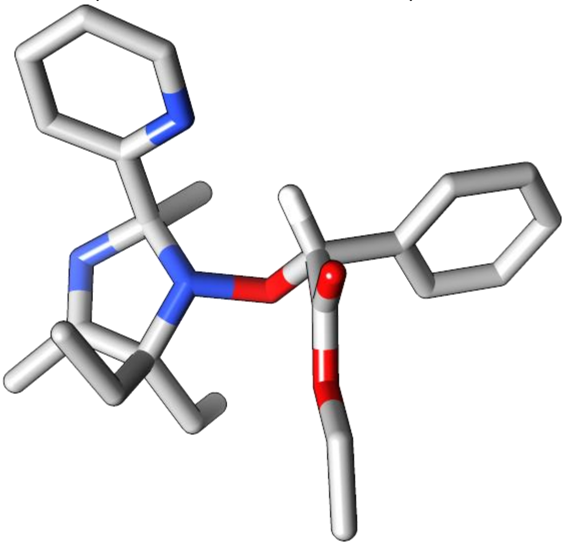

61

Energy -1321.803286988 Dipole 2.36 ZPE 0.499094 G(298.15) 272.26 Edisp -53.56

C	1.99470000	4.93210000	1.54099000	126.9569
C	2.64050000	4.37386000	0.43410000	127.6126
C	0.87137000	4.29829000	2.07683000	127.9646
C	2.16862000	3.18699000	-0.12772000	127.4938
C	0.39160000	3.11506000	1.51010000	125.0122
C	1.04112000	2.54750000	0.40647000	140.5863
C	0.53619000	1.27082000	-0.24326000	87.4709
C	-0.19653000	1.65080000	-1.54424000	174.4976
O	-0.26104000	0.55927000	0.70717000	124.0421
O	-1.50363000	1.94846000	-1.32348000	172.5210
O	0.35180000	1.73438000	-2.61891000	378.3402

N	-0.42496000	-0.80475000	0.23994000	198.4828
C	-2.22864000	2.41873000	-2.49510000	62.2458
C	-1.84895000	-1.25684000	0.35564000	86.2202
C	0.42131000	-1.77684000	1.03444000	105.3106
C	-3.63238000	2.78306000	-2.05218000	8.3008
C	-1.66968000	-2.58049000	1.08422000	177.7428
C	-2.43471000	-1.46731000	-1.07010000	32.9650
C	-2.78896000	-0.29567000	1.12910000	28.0701
N	-0.47475000	-2.85870000	1.44633000	347.2393
C	1.52112000	-2.34691000	0.11461000	170.8140
C	1.10073000	-1.17100000	2.27149000	22.9158
C	-2.81426000	-3.50841000	1.35428000	13.2637
C	-1.60129000	-2.37541000	-1.97608000	3.3088
C	-2.52046000	-0.12057000	2.62709000	7.2197
N	2.28310000	-1.44267000	-0.51931000	321.2659
C	1.72929000	-3.72507000	-0.00950000	121.5570
C	3.26262000	-1.88901000	-1.31451000	150.0551
C	2.75276000	-4.17864000	-0.84207000	134.6841
C	3.53866000	-3.24306000	-1.51487000	121.6240
H	2.36807000	5.85853000	1.98540000	7.3534
H	3.52171000	4.86198000	0.00931000	7.4384
H	0.36143000	4.72867000	2.94301000	7.4615
H	2.67116000	2.74949000	-0.99503000	7.7483
H	-0.48701000	2.61832000	1.92556000	7.7775
H	1.38934000	0.63850000	-0.54787000	7.9443
H	-2.22927000	1.62244000	-3.25801000	4.0485
H	-1.69093000	3.28276000	-2.91835000	3.9310
H	-4.20524000	3.15246000	-2.91765000	1.0689
H	-4.15834000	1.90904000	-1.63783000	1.3869
H	-3.61005000	3.57487000	-1.28798000	1.2427
H	-2.52430000	-0.46615000	-1.52440000	1.6448
H	-3.46157000	-1.86214000	-0.96864000	1.6483
H	-2.05245000	-2.44170000	-2.97891000	0.3571
H	-0.58174000	-1.97435000	-2.07747000	0.5408
H	-1.52458000	-3.39938000	-1.57333000	-0.1453
H	-2.73330000	0.67994000	0.62184000	3.1395
H	-3.81418000	-0.67858000	0.98164000	1.6266
H	-1.55521000	0.37688000	2.79625000	1.7482
H	-3.30820000	0.50363000	3.07964000	1.1877
H	-2.50820000	-1.08306000	3.16459000	0.8527
H	1.81501000	-0.39221000	1.97417000	1.7982
H	1.64078000	-1.97708000	2.78959000	1.4690
H	0.35547000	-0.74372000	2.95554000	1.5577
H	-3.24088000	-3.88066000	0.40734000	1.8537
H	-3.62812000	-2.99483000	1.89248000	1.9347
H	-2.46380000	-4.36253000	1.94978000	2.2671
H	1.08529000	-4.40880000	0.54582000	7.8880
H	3.86009000	-1.12219000	-1.82210000	8.9340
H	2.93337000	-5.25021000	-0.96358000	7.5655
H	4.34949000	-3.54951000	-2.17985000	7.1568

$2^{RR/SS}$i (RRi.01 from the site)

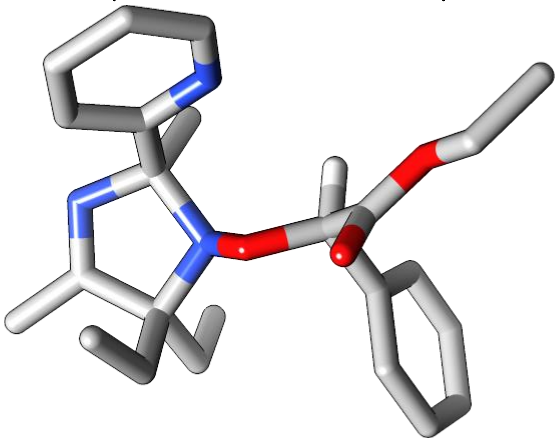

61
Energy -1321.799175743 Dipole 0.58 ZPE 0.498856 G(298.15) 272.50 Edisp -54.20

C	3.65502000	3.37369000	-0.12734000
C	3.06684000	3.01056000	-1.34089000
C	3.00655000	3.07510000	1.07580000
C	1.83680000	2.34756000	-1.35107000
C	1.77844000	2.41354000	1.06379000
C	1.18475000	2.03995000	-0.15171000
C	-0.13526000	1.29016000	-0.15962000
C	-1.18854000	2.08099000	0.62662000
O	-0.06955000	0.01120000	0.49224000
O	-1.81832000	2.94448000	-0.20919000
O	-1.40322000	1.98677000	1.81196000
N	0.68641000	-0.89581000	-0.34405000
C	-2.78166000	3.82716000	0.43122000
C	1.69457000	-1.64729000	0.47218000
C	-0.20115000	-1.93374000	-1.03263000
C	-3.35267000	4.73570000	-0.64007000
C	1.40761000	-3.07568000	0.03369000
C	1.54533000	-1.50059000	2.01176000
C	3.12665000	-1.18250000	0.08635000
N	0.39063000	-3.23474000	-0.72694000
C	-1.67180000	-1.87769000	-0.59693000
C	-0.13040000	-1.72081000	-2.55535000
C	2.24740000	-4.23272000	0.48161000
C	0.26598000	-2.05816000	2.64163000
C	3.46638000	-1.28102000	-1.40119000
N	-2.37089000	-0.80527000	-1.00584000
C	-2.25011000	-2.91731000	0.13864000
C	-3.66105000	-0.73080000	-0.66056000
C	-3.59923000	-2.83192000	0.48180000
C	-4.32480000	-1.71034000	0.08118000
H	4.61785000	3.89157000	-0.11756000
H	3.56956000	3.24013000	-2.28424000
H	3.46004000	3.36387000	2.02792000
H	1.37656000	2.05714000	-2.30012000
H	1.26060000	2.18377000	1.99969000
H	-0.50388000	1.17517000	-1.19099000
H	-3.56090000	3.21277000	0.91140000
H	-2.27002000	4.39536000	1.22561000
H	-3.85692000	4.15199000	-1.42537000
H	-2.56068000	5.34128000	-1.10643000
H	-4.08991000	5.41780000	-0.18763000
H	1.63356000	-0.42786000	2.24347000
H	2.42676000	-1.99419000	2.45765000
H	-0.61809000	-1.52003000	2.27360000
H	0.13487000	-3.13221000	2.42983000
H	0.30757000	-1.93840000	3.73652000
H	3.22611000	-0.13435000	0.41750000
H	3.84875000	-1.77136000	0.67990000
H	2.77606000	-0.65584000	-1.98620000
H	3.39452000	-2.31669000	-1.77464000
H	4.49292000	-0.92636000	-1.58595000
H	-0.64224000	-2.54944000	-3.06846000
H	0.91972000	-1.69181000	-2.87718000
H	-0.63265000	-0.77828000	-2.81290000
H	1.83094000	-5.16691000	0.08062000
H	3.28646000	-4.11953000	0.12905000
H	2.28605000	-4.29021000	1.58225000
H	-1.64118000	-3.78024000	0.41147000
H	-4.20118000	0.16107000	-1.00214000
H	-4.07724000	-3.63306000	1.05194000
H	-5.38322000	-1.59653000	0.32728000

Transition state of $2^{RS/SR}$-$2^{RS/SR}$i NO inversion

```
(RS.01-RSi.01 from the site)
61
 Energy -1321.777795957  Dipole 1.325796  ZPE 0.497975  G(298.15) 272.77  Edisp -53.10
 C    1.72391280  -4.83135814   2.05057899
 C    0.52972430  -4.26726418   2.50898293
 C    2.36259770  -4.29079247   0.93185424
 C   -0.02598647  -3.17068368   1.84898809
 C    1.81097573  -3.18812727   0.27588201
 C    0.61359898  -2.62148005   0.72821806
 C    0.01779671  -1.41467887   0.01988377
 C   -1.39256761  -1.79302476  -0.46478693
 O    0.02545935  -0.36551753   0.98785334
 O   -1.30027728  -2.41963864  -1.66656605
 O   -2.42768103  -1.61527117   0.13703409
 N    0.04517142   1.01570452   0.51664678
 C   -2.55312879  -2.93425121  -2.19953961
 C   -0.73087567   1.89272343   1.40200156
 C    0.13086829   1.54539828  -0.86461421
 C   -2.24106236  -3.63966424  -3.50491412
 C   -0.73827170   3.11689133   0.51036833
 C    0.05636138   2.12142873   2.72834888
 C   -2.16761399   1.41751497   1.78157647
 N   -0.28072508   2.94381137  -0.67547529
 C    1.60595962   1.50592791  -1.32564379
 C   -0.76121462   0.96199060  -1.98141543
 C   -1.24791764   4.44565749   0.97241648
 C    1.49751996   2.59578339   2.53894565
 C   -3.25782727   1.49245716   0.70826167
 N    2.09657291   0.29573648  -1.63694432
 C    2.38149102   2.66970985  -1.40293492
 C    3.38135820   0.20799427  -2.00457801
 C    3.71480236   2.56689533  -1.79451198
 C    4.23464024   1.30727884  -2.09879505
 H    2.15660977  -5.69312755   2.56582330
 H    0.02617925  -4.68799502   3.38343596
 H    3.29776897  -4.72704437   0.57072585
 H   -0.96538926  -2.73163845   2.19497258
 H    2.31248190  -2.75458672  -0.59370676
 H    0.66155627  -1.15924800  -0.83996773
 H   -3.25096603  -2.09256617  -2.34208565
 H   -3.00035014  -3.61568112  -1.45721138
 H   -1.79481494  -2.94452975  -4.23249331
 H   -1.54347730  -4.47551900  -3.34328765
 H   -3.17145983  -4.04227772  -3.93597321
 H    0.05046912   1.15689766   3.26531947
 H   -0.50979118   2.83501031   3.35240071
 H    2.03826285   1.90244725   1.87788902
 H    1.54430789   3.60046360   2.08541341
 H    2.02047418   2.64126185   3.50747152
 H   -2.06304946   0.37298789   2.11702815
 H   -2.47884488   2.01158436   2.65837624
 H   -3.09234955   0.74814627  -0.08123292
 H   -3.33152269   2.49029102   0.24555636
 H   -4.23564621   1.26092443   1.16095350
 H   -0.72532402   1.66247086  -2.83021093
 H   -0.39948818  -0.01511700  -2.32378138
 H   -1.80151230   0.88368734  -1.63830138
 H   -1.21360710   5.16397763   0.14159913
 H   -2.28264023   4.36247827   1.34415860
 H   -0.63595842   4.82559424   1.80792026
 H    1.92028755   3.63012659  -1.16915709
 H    3.74931106  -0.79730843  -2.24441567
 H    4.34076611   3.46059429  -1.86590919
 H    5.27405532   1.17654372  -2.40843143
```

The most stable conformers 3, 3i

```
R-005 from the site
63
 Energy -1248.063095065  Dipole 1.322763  ZPE 0.528159  G(298) 293.58  Edisp -55.44
 C   -0.25853699  -3.20477667   0.87663651
 C   -1.10783672  -2.51410871  -0.17971095
 N   -0.47207773  -1.15383868  -0.19963520
 C    0.50926559  -1.10227560   0.94888017
 N    0.61287797  -2.46954727   1.45614760
 C   -0.92541670  -3.20325303  -1.56299111
 C    0.52290885  -3.29653032  -2.04593341
 C   -2.61669619  -2.54333794   0.17574993
 C   -3.01140599  -2.06761368   1.57667521
 C    1.86609888  -0.64347650   0.39225512
 C    0.10953277  -0.17391586   2.10473646
 N    1.83067439   0.48343753  -0.33300285
 C    2.97822544   0.93712115  -0.84965686
 C    4.20959817   0.30341992  -0.66672855
 C    4.24179749  -0.86087537   0.10211515
 C    3.05076787  -1.34380813   0.64564645
 O   -1.46261319  -0.11042046  -0.13996163
 C   -1.41513041   0.84331994  -1.24614674
 C   -0.88905340   0.27262802  -2.55456901
 C   -2.88558566   1.27933661  -1.38196760
 C   -0.62078575   2.14399252  -0.90379692
 O   -0.00285698   2.78310731  -1.72834603
 O   -0.86976932   2.52529902   0.37091631
 C   -0.40465473   3.84664892   0.87309066
 C   -1.07346719   4.96530377   0.06814005
 C    1.12259499   3.93589446   0.82357288
 C   -0.90232029   3.85301728   2.32024578
 C   -0.42001957  -4.65557784   1.21553606
 H   -1.52832480  -2.63701105  -2.29357875
 H   -1.37456025  -4.21114359  -1.50944057
 H    0.56676636  -3.71778897  -3.06283931
 H    1.13279722  -3.93860660  -1.38889393
 H    0.98536617  -2.29837115  -2.06104196
 H   -3.14279936  -1.93642988  -0.57887477
 H   -2.95100813  -3.58546343   0.02945909
 H   -4.08524304  -2.24835259   1.74751493
 H   -2.82363244  -0.99046072   1.68340464
 H   -2.45405947  -2.59902708   2.36587324
 H   -0.82026774  -0.52599272   2.57271233
 H   -0.03684807   0.84514398   1.72950975
 H    0.91296737  -0.19173652   2.85604037
 H    2.90487298   1.85604392  -1.44421997
 H    5.11625608   0.71573928  -1.11604370
 H    5.18356057  -1.38910692   0.27521260
 H    3.01252381  -2.24889579   1.25425042
 H   -0.90248316   1.06500932  -3.31503652
 H    0.14142069  -0.07992153  -2.42880777
 H   -1.52552791  -0.56020648  -2.88735137
 H   -2.97049020   2.12172070  -2.08605794
 H   -3.27357610   1.59331862  -0.40225840
 H   -3.48824289   0.43896718  -1.75979720
 H   -2.16956315   4.86118722   0.10535264
 H   -0.80542268   5.94000568   0.50654561
 H   -0.74235858   4.94227201  -0.97847980
 H    1.44570926   4.86529432   1.31996107
 H    1.47627428   3.94030269  -0.21525552
 H    1.57448588   3.08211591   1.35070950
 H   -0.63749944   4.80793914   2.80016145
 H   -1.99634004   3.73477705   2.35385263
 H   -0.44248954   3.03390420   2.89430026
 H    0.28483557  -4.92600869   2.01372854
 H   -1.44864519  -4.86943716   1.55126410
 H   -0.23073594  -5.28982194   0.33334817
```

```
Ri-001 from the site
63
 Energy -1248.064165692  Dipole 1.518523  ZPE 0.528617  G(298) 293.66  Edisp -54.59
 C   -2.43810709  -1.85098213   0.13487173
 C   -1.86775045  -0.52351168  -0.32992037
 N   -0.54146855  -0.51257443   0.38791682
 C   -0.38237782  -1.89792456   1.03291595
 N   -1.65073182  -2.59289992   0.81707806
 C   -1.78845993  -0.46527402  -1.88016387
 C   -1.17568339  -1.67455561  -2.59082248
 C   -2.73591526   0.67551737   0.14638848
 C   -2.89087592   0.80960565   1.66182964
 C    0.77951457  -2.73600590   0.46865955
 C   -0.19783153  -1.74718992   2.55254151
 N    2.02127697  -2.39279034   0.84952732
 C    3.04620131  -3.12344632   0.39520825
 C    2.89964952  -4.23379161  -0.43799888
 C    1.60741289  -4.60379425  -0.80975215
 C    0.53211239  -3.84532013  -0.34908784
 O    0.54455984  -0.21763052  -0.52761638
 C    1.31635111   0.94043978  -0.13635379
 C    1.78568933   0.89159433   1.31696959
 C    2.51357598   0.87763732  -1.10184678
 C    0.55400713   2.25165308  -0.48660832
 O   -0.23974820   2.35638577  -1.39966368
 O    0.96419810   3.26084448   0.31277802
 C    0.47921539   4.65455491   0.10526186
 C   -1.03666965   4.71863158   0.30938545
 C    0.89990345   5.15310262  -1.27955002
 C    1.20736746   5.42700559   1.20684551
 C   -3.83240754  -2.28583172  -0.20173321
 H   -1.22177562   0.44444840  -2.12747070
 H   -2.82175831  -0.31025776  -2.23818317
 H   -1.20655435  -1.51806828  -3.68178966
 H   -1.72029571  -2.60939643  -2.37649349
 H   -0.12582947  -1.81221490  -2.29672318
 H   -2.28065615   1.58835211  -0.26985859
 H   -3.72951851   0.58029201  -0.32682418
 H   -3.51228942   1.68483868   1.91104096
 H   -1.90568336   0.93616320   2.13534428
 H   -3.36852767  -0.07921192   2.10844871
 H   -0.99338384  -1.10530638   2.95650881
 H    0.78486505  -1.31399559   2.77362102
 H   -0.26476342  -2.73927610   3.02445579
 H    4.04514704  -2.81017153   0.72389991
 H    3.77495253  -4.79612736  -0.77196050
 H    1.43626841  -5.47632718  -1.44604638
 H   -0.49735602  -4.10910103  -0.59241289
 H    2.26508931  -0.08464952   1.48377897
 H    2.49746004   1.70415479   1.51338924
 H    0.93397056   0.99463342   2.00260901
 H    3.15892388   1.75769779  -0.96348335
 H    2.15669860   0.85013625  -2.14220873
 H    3.09324499  -0.03373063  -0.89281207
 H   -1.30854980   4.29227510   1.28784414
 H   -1.36103832   5.77155855   0.28980114
 H   -1.56164956   4.16894468  -0.48237136
 H    0.62739230   6.21585669  -1.38112638
 H    0.39925603   4.57967676  -2.07058949
 H    1.99102581   5.06565963  -1.40282290
 H    0.93010355   6.49152006   1.16207421
 H    0.93533417   5.03466223   2.19871320
 H    2.29801696   5.34360009   1.08245984
 H   -3.99984001  -3.30478968   0.17355842
 H   -4.57278553  -1.60825805   0.25612031
 H   -4.00355679  -2.26005400  -1.29065240
g
```

Transition state of 3-3i NO inversion

```
022-045 from the site
63
 Energy -1248.032024765  Dipole 1.698988  ZPE 0.527445  G(298.15) 293.34  Edisp -54.07
 C   -4.91331781  -0.06160180  -1.98146283
 C   -4.65922674   0.90609114  -1.00538264
 C   -3.53517991   0.76238755  -0.19771788
 C   -2.69904149  -0.34491868  -0.38838276
 N   -2.93568041  -1.27602298  -1.32146920
 C   -4.02163677  -1.12684792  -2.09508534
 C   -1.47073647  -0.53874908   0.52920690
 N   -1.98401172  -0.79595410   1.89367304
 C   -1.37959862  -0.03559817   2.73376905
 C   -0.32203462   0.88786671   2.14249814
 N   -0.74046408   0.72188949   0.73927443
 C   -1.69515737  -0.10888415   4.19623583
 C   -0.44439228   2.36739688   2.58417702
 C   -1.76081601   3.05629498   2.22326804
 C    1.11917652   0.36297051   2.44445670
 C    1.75347509   0.73109893   3.79303669
 O    0.00861100   1.53158020  -0.19444288
 C    0.67195166   1.04149915  -1.39748638
 C    1.41211790   2.32361920  -1.83397896
 C   -0.66308078  -1.79205432   0.13368678
 C    1.80452404   0.00659678  -1.14984951
 O    1.79553511  -0.94984522  -2.10425793
 C    2.89401489  -1.95824156  -2.19339281
 C    2.47746144  -2.79544616  -3.40362133
 O    2.62949823   0.11601247  -0.26697694
 H   -0.82037816  -0.44978303   4.77395723
 H   -2.52291471  -0.81567154   4.34846183
 H   -1.97782393   0.88031522   4.59297869
 H   -0.28299415   2.41349893   3.67392143
 H    0.39378181   2.91603492   2.11993059
 H    1.75196627   0.74662169   1.62909991
 H    1.11486761  -0.73496789   2.33797539
 H   -1.78274998   4.07718300   2.63796554
 H   -1.87391981   3.12685872   1.13183186
 H   -2.63194119   2.51343707   2.62835199
 H    2.74169597   0.24919822   3.87467024
 H    1.91218608   1.81580211   3.89319207
 H    1.15665511   0.39737513   4.65732869
 H   -1.31594936  -2.67223862   0.20390470
 H   -0.28272924  -1.73404582  -0.89289417
 H    0.17932442  -1.90745877   0.82916666
 C   -0.33168025   0.64390556  -2.48405679
 C    4.22349661  -1.24533573  -2.45339630
 C    2.93625326  -2.81619485  -0.92664113
 H    3.22020410  -3.58890279  -3.57926803
 H    1.49699573  -3.26480834  -3.23052113
 H    2.41378449  -2.16884106  -4.30653426
 H   -5.33065417   1.75951625  -0.87711423
 H   -3.28421249   1.48975106   0.57444584
 H   -4.18501469  -1.90433641  -2.85172280
 H   -5.78184058   0.00724901  -2.64104578
 H    1.93309492   2.15487151  -2.78853503
 H    0.67591094   3.13064155  -1.96486853
 H    2.14285448   2.61035704  -1.06534358
 H    0.18108607   0.54354571  -3.45130613
 H   -0.84943237  -0.30007823  -2.27713753
 H   -1.08538263   1.44249669  -2.56151395
 H    5.00937328  -1.99789790  -2.62611506
 H    4.15049823  -0.61312723  -3.35247380
 H    4.50989243  -0.62365181  -1.59487301
 H    3.69162118  -3.60800897  -1.05617838
 H    3.20130715  -2.20848598  -0.05197624
 H    1.96077678  -3.29596423  -0.75344198
```

DFT "transition state" of $2^{RS/SR}$-$2^{RR/SS}$ epimerization

```
61
 Energy -1321.752224637  Dipole 6.472457  ZPE 0.495279  G(298.15) 269.07
 C   -2.26739980  -4.38591591   2.65388665
 C   -1.78502500  -3.07057451   2.56903475
 C   -2.32957336  -5.16774507   1.49196686
 C   -1.36983430  -2.55204252   1.35347713
 C   -1.92522803  -4.66015617   0.26513555
 C   -1.43102403  -3.32430113   0.15005228
 C   -1.00664477  -2.70163815  -1.05281611
 C   -0.89514670  -3.20907454  -2.39920067
 O   -0.45506504   0.88800725   1.29848547
 O   -1.29906223  -4.51835624  -2.56355354
 O   -0.47317427  -2.55305063  -3.35294128
 N    0.17882162   1.94799966   1.07496916
 C   -1.18352405  -5.02379330  -3.91205880
 C    1.07127110   2.59608563   2.07796296
 C   -0.02558381   2.85533711  -0.11722351
 C   -1.67195269  -6.46148326  -3.90735810
 C    1.25307260   3.93763937   1.39202790
 C    2.37661657   1.77601246   2.25451661
 C    0.34211412   2.67855012   3.44351452
 N    0.67032680   4.07494207   0.25656317
 C    0.60238930   2.22270782  -1.37642127
 C   -1.52405780   3.07512259  -0.34583623
 C    2.06009937   5.03513115   2.00903581
 C    3.24376065   1.62508922   1.00368455
 C   -0.97070997   3.46423014   3.44789552
 N    0.13671129   1.01470337  -1.71134350
 C    1.56634405   2.91303077  -2.11901866
 C    0.63502537   0.43192844  -2.81371530
 C    2.07037576   2.30501152  -3.26943293
 C    1.60065790   1.04042420  -3.62237551
 H   -2.58615064  -4.79744804   3.61500972
 H   -1.72773477  -2.45144859   3.46875222
 H   -2.70548044  -6.19340086   1.54926442
 H   -0.98054879  -1.53107429   1.29637997
 H   -1.98628954  -5.26953964  -0.63546489
 H   -0.70689749  -1.65173083  -0.97572429
 H   -0.13279276  -4.95139364  -4.24112143
 H   -1.78061984  -4.39241110  -4.59192823
 H   -1.06407499  -7.08247391  -3.23104946
 H   -2.72366921  -6.51973005  -3.58635023
 H   -1.59682317  -6.88084092  -4.92339920
 H    2.06111107   0.78058837   2.61407948
 H    2.95411278   2.24311016   3.07108953
 H    2.70457556   1.09835446   0.20223166
 H    3.57115069   2.59972770   0.60666127
 H    4.14571497   1.04047874   1.24339005
 H    0.14895827   1.63538264   3.74688378
 H    1.05278011   3.10478415   4.17147496
 H   -1.72634025   2.97283581   2.81651441
 H   -0.83931746   4.49799145   3.08868649
 H   -1.37502462   3.51322779   4.47105143
 H   -1.66465686   3.71663265  -1.22696501
 H   -1.98305632   2.09304593  -0.52586505
 H   -1.98263822   3.55547633   0.53054136
 H    2.04713133   5.91583757   1.35257464
 H    1.65837428   5.31119861   2.99849238
 H    3.10298347   4.71046501   2.16466994
 H    1.88922950   3.90445170  -1.79777662
 H    0.24455051  -0.56690948  -3.05753364
 H    2.81891422   2.81654062  -3.88085382
 H    1.96794794   0.52445019  -4.51262933
```

The "transition state" is taken in quotation marks because its structure has a significant contribution of a singlet biradical state, for which the DFT method is unsuitable. Indeed, N_{FOD} value for the DFT/PBE/Λ1 calculated **TS** is 1.83, thus falling under point c) of the Grimme's rules of thumb: "significant and delocalized ρ^{FOD}: use multi-reference methods" [Neese, F. *The ORCA program system. Wiley Interdisciplinary Reviews: Computational Molecular Science*, **2012**, *2*, 73–78; Grimme, S.; Hansen, A. A Practicable Real-Space Measure and Visualization of Static Electron-Correlation Effects. *Angew. Chem. Int. Ed.* **2015**, *54*, 12308–12313].

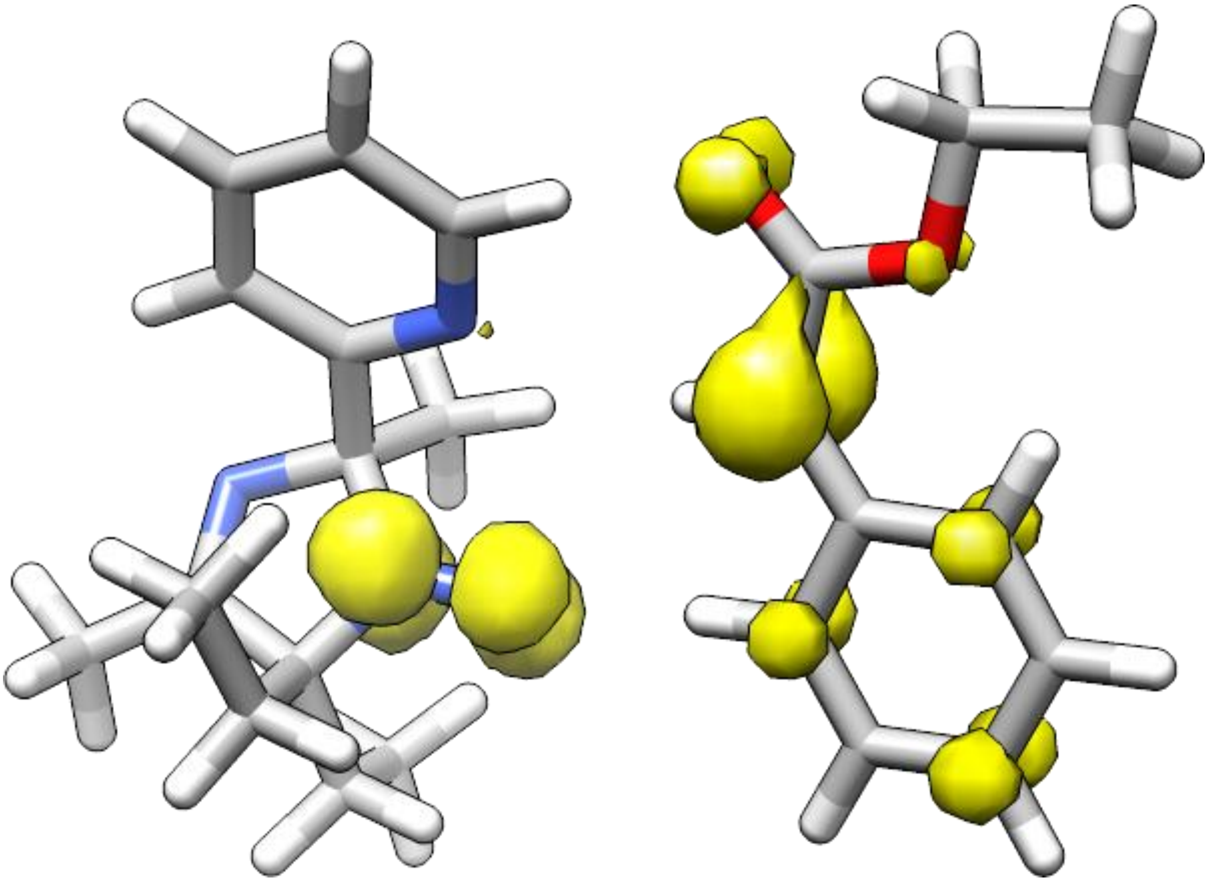

FOD plot at σ = 0.005 e/Bohr3 (TPSS/def2-TZVP (T = 5000 K) level); FOD depicted in yellow; DFT/PBE/Λ1 optimized geometry

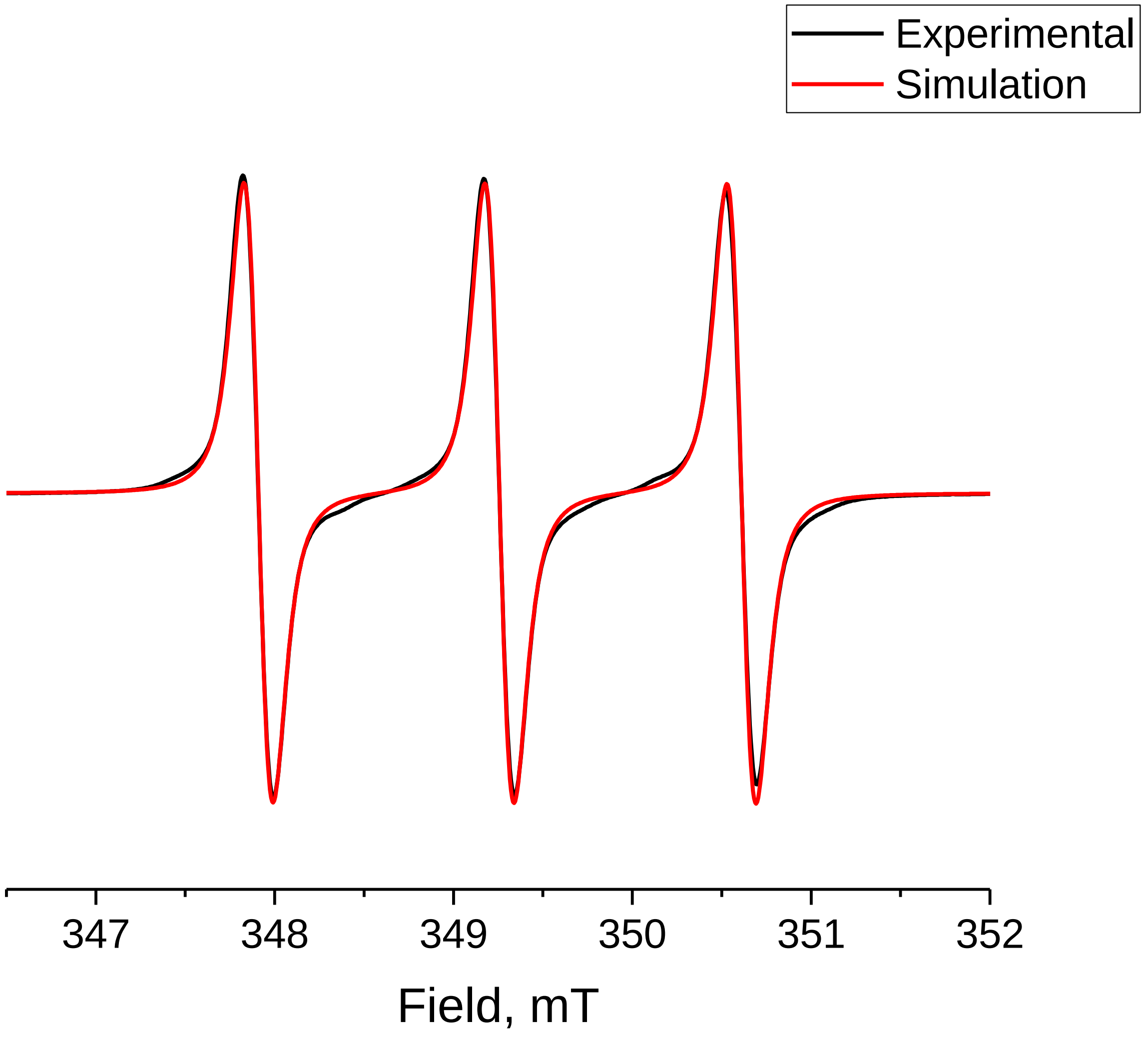

EPR spectrum of radical 1•; g = 2.00608, nitrogen hyperfine coupling constant is 1.351 mT. The EPR spectrum of **1•** was recording at the following parameters: microwave power 2 mW, resolution – 1024 points, number of scans 24, conversion time 20.74 ms, modulation amplitude 0.5 G, time constant 20.48 ms.
